# Logic models to predict continuous outputs based on binary inputs with an application to personalized cancer therapy

**DOI:** 10.1038/srep36812

**Published:** 2016-11-23

**Authors:** Theo A. Knijnenburg, Gunnar W. Klau, Francesco Iorio, Mathew J. Garnett, Ultan McDermott, Ilya Shmulevich, Lodewyk F. A. Wessels

**Affiliations:** 1 Institute for Systems Biology, Seattle, US; 2 Centrum Wiskunde & Informatica, Amsterdam, The Netherlands; 3 European Molecular Biology Laboratory - European Bioinformatics Institute, UK; 4 Wellcome Trust Sanger Institute, UK; 5 Netherlands Cancer Institute, Amsterdam, and The Faculty of EEMCS, Delft University of Technology, Delft, The Netherlands

## Abstract

Mining large datasets using machine learning approaches often leads to models that are hard to interpret and not amenable to the generation of hypotheses that can be experimentally tested. We present ‘Logic Optimization for Binary Input to Continuous Output’ (LOBICO), a computational approach that infers small and easily interpretable logic models of binary input features that explain a continuous output variable. Applying LOBICO to a large cancer cell line panel, we find that logic combinations of multiple mutations are more predictive of drug response than single gene predictors. Importantly, we show that the use of the continuous information leads to robust and more accurate logic models. LOBICO implements the ability to uncover logic models around predefined operating points in terms of sensitivity and specificity. As such, it represents an important step towards practical application of interpretable logic models.

Regression and classification models are important tools for researchers in various fields. The application of these many-to-one mapping models is two-fold. First, they can be used for prediction. The output value or class of a (new) case can be predicted by applying the inferred mapping to the input variables of the case. Second, they inform us about the relationship between the input and the output. They specify how the input variables are (mathematically) interacting with each other to produce the output variable. The usefulness of the second application is, however, limited by the power of the human intellect. We suggest that the interpretation of these many-to-one mapping models is of utmost, yet undervalued, importance in many research fields.

This also holds for computational biology, where a multitude of molecular and genomic data is frequently used to explain or predict a biological or clinical phenotype. Single predictor models are generally not accurate enough, reflecting the importance of acknowledging the interaction between biological components. On the other hand, machine learning approaches, such as Elastic Net^1^ and Random Forests^2^ produce complex multi-predictor models that are hard to interpret and not amenable to the generation of hypotheses that can be experimentally tested. As a consequence, such models are not likely to further our understanding of biology. There is an urgent need for approaches that build small, interpretable, yet accurate models that capture the interplay between biological components and explain the phenotype of interest.

In this study, we have developed such a modeling framework to explain drug response of cancer cell lines using gene mutation data. Our approach, ‘Logic Optimization for Binary Input to Continuous Output’ (LOBICO) infers small and easily interpretable logic models of gene mutations (binary input variables) that explain the observed sensitivity to anticancer drugs in the cell lines (continuous output).

The contributions of our approach are three-fold: First, the continuous information of the output variable is retained in the logic mapping. The output variable is binarized, which facilitates its interpretation, yet the distances of the continuous values to the binarization threshold are used in the inference. Second, LOBICO provides the user with the option to include constraints on the model performance that allows the identification of logic models around operating points predefined in terms of sensitivity and specificity. This enables tailoring of the model to, for example, clinical applications where the severity of diseases or side effects of the treatment dictate a desired level of specificity or sensitivity. Third, the logic mapping is formulated as an integer linear programming problem (ILP). This means that advanced ILP solvers can be used to find an optimal logic mapping fast enough to apply LOBICO to large and complex datasets without the need to tune parameters.

Our work is similar in spirit to logic regression (LR)^3^,^4^, sparse combinatorial inference (SCI)^5^, Markov logic networks^6^,^7^, combinatorial association logic (CAL)^8^, CellNetOptimizer^9^ and genetic programming for association studies (GPAS)^10^, which all employ combinatorial logic to explicitly incorporate interactions in their models. The most important aspect in which LOBICO differentiates itself from these approaches is by its direct emphasis on interpretability. This is in contrast with the linearly weighted sums of logic functions as inferred by LR or the posterior probabilities of predictors in the model averaged across an ensemble of many solutions as inferred by SCI. Graphical models, such as Bayesian networks^11^ and Markov random fields^12^ also facilitate interpretation, although due to their probabilistic nature they do not lend themselves to standard formal reasoning as well as logic models do. MOCA (Multivariate Organization of Combinatorial Alterations)^13^ deserves special attention as it has also been applied to predict drug response by inferring logic combinations of genomic input features. The most important differences with our work are: (1) MOCA employs a heuristic, sub-optimal progressive selection of features to infer logic formulas, and (2) MOCA uses discretized drug response values and discards the information in the continuous values that LOBICO uses in its model inference. Moreover, LOBICO includes constraints on statistical performance criteria, such as a minimum specificity, which is a novel feature not available in any other approach.

Here, we demonstrate LOBICO by application to a large cancer cell line panel, where the goal is to explain drug response based on binary mutation data of a set of genes^14^. We investigate whether logic models perform better than single-gene predictors, and put genes that co-occur in logic models in the context of known cancer pathways. We assess whether using continuous output values provides benefit in terms of robustness and performance above the use of binarized data, which is usually the starting point for logical analysis of data^15^. We also provide a comparison with Elastic Net, Random Forests and LR. Finally, we explore the power and flexibility of the ILP formulation by inferring logic models around different operating points in the receiver-operator-characteristic (ROC) space. We show that the ability to find robust and interpretable models with a predefined balance between false positives and false negatives is an important step towards practical application of these models.

## Results

### Sensitivity to anticancer drugs as a logic combination of gene mutations

We used LOBICO to find the logic combinations of mutations that best explain the response of cancer cell lines to anticancer drugs. This analysis was based on a panel of 714 cancer cell lines from more than 50 tumor types for which the binary mutation status of 54 known cancer genes was obtained^14^. A gene was called mutated when it had a point mutation, a small insertion or deletion as determined by capillary sequencing, or when it was highly amplified or homozygously deleted based on copy number arrays. Additionally, we included the presence or absence of 6 known oncogenic gene fusions across these cell lines, resulting in a binary data matrix with a total of 60 features. The cell lines in the panel were screened against 142 anticancer drugs. The half-maximal inhibitory concentrations at 72 hours (IC50s) obtained in this screen were used to represent the drug response and served as the continuous output variables. LOBICO was applied to the IC50s of each drug separately.

**Figure 1:**
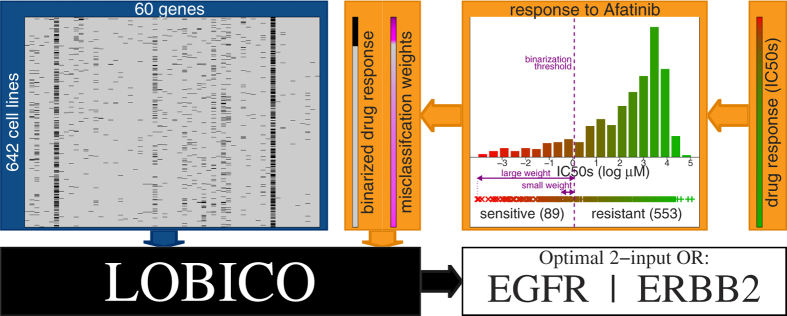
Workflow of LOBICO. LOBICO has two main inputs: (1) a binary matrix of samples by features (depicted in the blue box). Here, the binary matrix contains the mutation status of 60 cancer genes measured across 642 cancer cell lines. (2) a continuous vector with a value for each of the samples (depicted in the orange boxes). In this case, the vector contains the IC50 of each cell line in response to Afatinib, an EGFR/ERBB2 inhibitor. The continuous vector is transformed into a binary vector and a sample-specific weight vector using a binarization scheme. Particularly, the IC50s are binarized using a threshold leading to a set of sensitive and a set of resistant cell lines. The distances of the original IC50s to the binarization threshold are represented in the weight vector, which is normalized per class. Then, LOBICO finds the optimal logic model of features (gene mutations) that minimizes the total weight of misclassified samples (cell lines). In this case, the optimal 2-input OR logic formula is ‘EGFR OR ERBB2’ (depicted in the white box).

Figure 1 provides a schematic overview of the application of LOBICO to this dataset using the IC50s of the EGFR/ERBB2 inhibitor Afatinib as the output variable. For Afatinib, IC50s were obtained for 642 of the 714 cell lines. Although LOBICO uses the continuous values, it is also necessary to define a binarization threshold for the output variable. This threshold is used to divide the cell lines into two classes; the sensitive cell lines and the resistant cell lines. LOBICO finds an optimal logic function of binary mutation features that minimizes the error, which is defined as the sum of the weighted misclassified cell lines. A misclassification occurs when a sensitive cell line is predicted by the model to be resistant or vice versa. The weight is proportional to the distance to the binarization threshold. Consequently, misclassification of cell lines close to the binarization threshold does not considerably affect the optimization criterion, whereas there is a large penalty for misclassifying cell lines that are extremely sensitive or resistant to the drug. By default, the total weight associated with samples of each class is normalized in order to balance class importance. This is especially important for unbalanced classes as is often the case for the cell line panel, where we observed that, for most drugs, the bulk of cell lines are not affected, and only a small percentage (5–15% typically) end up in the class of sensitive cell lines. The optimization procedure is formulated as an integer linear programming (ILP) problem, which provides a practically efficient way to find optimal solutions. See Methods Section for more details.

### Drug response is explained by multiple mutations

LOBICO finds logic models that are described in the disjunctive normal form (DNF), a standard notation in which every logic function can be expressed. The DNF is parameterized by two parameters: *K*, the number of disjuncts, and *M*, the number of terms per disjunct. We applied LOBICO with eight different parameter settings, i.e. from simple single predictor models (*K* = 1, *M* = 1) to more complex multi-predictor models (*K* > 1, *M* > 1). See Table 1 for examples of the eight models. A stratified 10-fold cross-validation (CV) strategy was employed to select the appropriate model complexity, i.e. the one with the lowest CV error.

**Figure 2:**
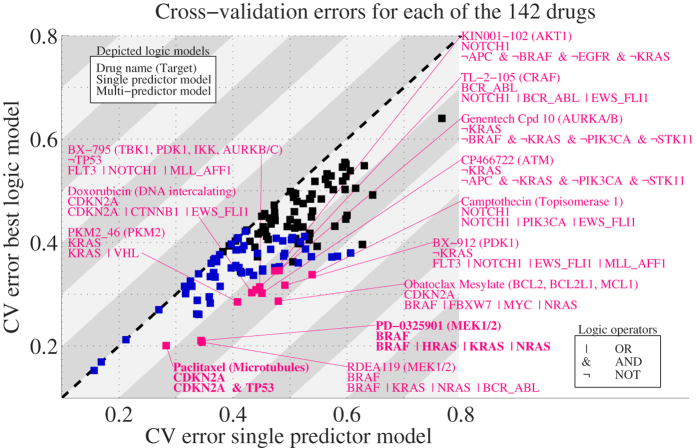
Multi-predictor models outperform single predictor models. Scatter plot with the 10-fold cross-validation (CV) error for single predictor models (x-axis) and the best (lowest CV error) multi-predictor model (y-axis). Each point represents one of the 142 drugs. Statistically significant models are highlighted in blue. Multi-predictor models that have a CV error lower than 0.35 and at least a 25% improvement upon the single predictor model are highlighted in magenta. The two examples discussed in the text are highlighted in bold typeface.

Application across all 142 drugs revealed that the large majority of drugs (85%) are best explained by multi-predictor models (Table 1, Supplementary Data 1 and 2). This number remains high (81%) if we only consider the 72 drugs for which the CV error is statistically significant at a FDR < 1% and p < 0.01. In many cases the multi-predictor model selected by CV was substantially better than the single predictor model (Fig. 2, Supplementary Figure 1). For example, LOBICO inferred a 4-input OR model for the MEK1/2 inhibitor PD−0325901, which had a CV error of 0.21 while the best single input model only reached 0.34. The 4-input OR model included the genes BRAF, NRAS, KRAS and HRAS. Thus, cell lines with a BRAF mutation or a mutation in NRAS, KRAS or HRAS (members of the RAS protein family) are predicted to be sensitive to this drug. Since BRAF and RAS are directly upstream of MEK in the MEK-ERK pathway, this association is easily understood from pathway biology. In general, OR models will increase the number of cell lines (or patient population) that are predicted to respond to a drug. On the other hand, AND models refine the predictions. For example, LOBICO inferred that cell lines with a mutation in both tumor suppressors TP53 and CDKN2A specifically respond to the microtubule inhibitor Paclitaxel (Taxol). These results strengthen the notion that gene mutations should not be considered in isolation, but in relationship to one another, if they are to be used as prognostic biomarkers or predictors of therapy response.

We validated these models using an independent drug sensitivity dataset from the Cancer Therapeutics Response Portal (CTRP, 2^nd^ version)^16^. Our analyses were restricted to those compounds (n = 47) overlapping with our own study. We explored two scenarios. In Scenario 1 we trained LOBICO models on our data using the 344 cell lines overlapping with CTRP and validated on the CTRP drug response measurements of these 344 cell lines. In Scenario 2 we trained LOBICO models on the 377 cell lines that are unique to our dataset and then validated on the independent set of 344 CTRP cell lines. See Supplementary Figure 2 for an overview of these datasets. The results of these validation analyses are presented in Table 2. When training (using our data) and testing (using CTRP drug response) on the same cell lines (Scenario 1), we validated 5 of the 18 models (28%) that were statistically significant in the training phase, including both single and multi-input models (Supplementary Figures 3 and 4). Importantly, 4 of the 5 best performing models on our cell line panel were validated on CTRP with considerable statistical significance. Using the independent set of cell lines as a validation cohort (Scenario 2) we found that the two best performing models on GDSC validated with high statistical significance in CTRP (Supplementary Figure 5). Yet, many other strong models on GDSC, i.e. those with high statistical significance, did not lead to a significant prediction of drug response in CTRP (Supplementary Figure 6). Still, 5 of the 25 models (20%) that were statistically significant in the training phase also showed statistical significance in the CTRP validation cohort. Similar to Scenario 1, we found that in many cases multi-predictor models outperformed single predictor models also on the validation cohorts. Overall, strong logic models validate in CTRP, whereas this is not necessarily the case for weaker, yet still statistically significant models, and generalization of these models to different cell line panels should be approached with caution. See Supplementary Note 1 for details.

### Use of continuous output yields more robust and accurate models

After LOBICO was successfully employed to infer sensible models of drug response, we set out to quantify the contribution of the use of the sample-specific weights. In other words, is it advantageous to use the distances from the continuous IC50s to the binarization threshold as sample-specific misclassification penalties? Or, would simply using binarized data as is done in standard logical data analysis^15^ as well as in a recent application to cancer cell line drug screening data^13^ be equally informative?

To this end, we analyzed logic models under slightly different binarization thresholds. Specifically, we shifted the default threshold (*t* = 0.05) slightly downwards (*t* = 0.03) and upwards (*t* = 0.07) leading to, on average, 24% less and 22% more sensitive cell lines, respectively. As such, these parameter changes lead to a small (not insignificant, yet also not substantial) change in the assignment of cases (sensitive cell lines) and controls (resistant cell lines). See Methods Section for details. Then, we ran LOBICO across these binarization thresholds with and without the sample-specific weights. The logic models for a drug were inferred using the model complexity (defined by *K* and *M*) selected by CV for that drug in the standard setting, i.e. with the sample-specific weights and *t* = 0.05. We conjectured that small changes in the binarization thresholds should only moderately affect the inferred logic models, i.e. the models should be robust against these small changes.

**Figure 3:**
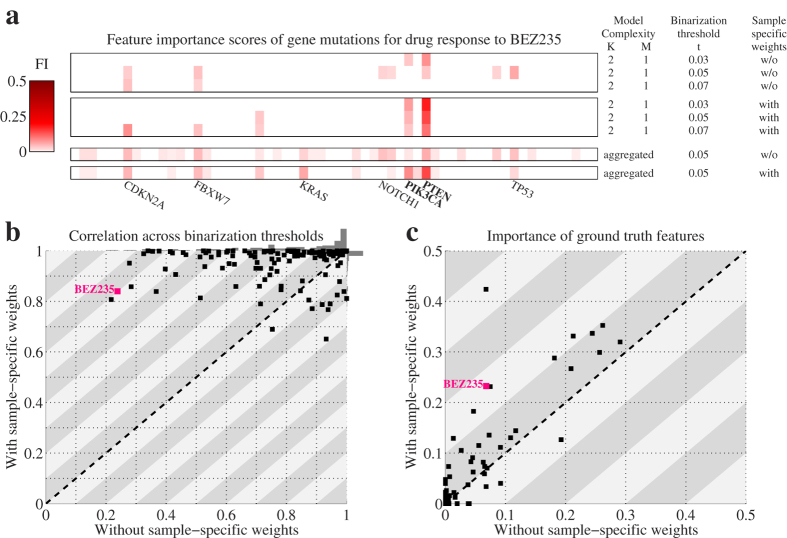
LOBICO’s use of continuous output leads to robust and accurate models. (**a**) Heatmaps depicting the feature importance (FI) scores across the 60 gene mutations for the logic models inferred to explain the drug response to the PI3K/mTOR inhibitor BEZ235. The upper heatmap represents FI scores for the 2-input OR model (*K* = 2, *M* = 1) using three different binarization thresholds for logic models with binarized output, i.e. not using the sample-specific weights. The middle of the three heatmaps represents the same FI scores, but for logic models with continuous output, i.e. using the sample-specific weights. The bottom two heatmaps depict FI scores aggregated across all model complexities, using the standard binarization threshold (*t* = 0.05), for both the logic models with and without the sample-specific weights. The labels of the gene mutations with a large FI in any of these heatmaps are printed below. The ‘ground truth’ features, i.e. the expected or annotated targets of this drug, PTEN and PIK3CA, are printed in bold. (**b**) Scatter plot with the average Pearson correlation coefficients of the similarity of FI scores across the binarization thresholds for inferred logic models without (x-axis) and with (y-axis) the sample-specific weights. Each point represents one of the 142 drugs. The correlation scores are computed using the model-complexity-specific FI scores. The grey bars on top and to the right of the scatter plot represent histograms of these correlation scores for models without and with the sample-specific weights, respectively. (**c**) Scatter plot with the importance of the ground truth features for inferred logic models without (x-axis) and with (y-axis) the sample-specific weights. Each point represents one of the 49 drugs, for which ground truth features were available. The importance scores of the ground truth features were derived from aggregated FI scores.

In order to evaluate the robustness of the logic models we examined the feature importance (FI) scores of these models across the binarization thresholds. Specifically, we used the correlation of FI scores across the binarization thresholds as a measure of robustness, where the degree of change in the FI scores is inversely proportional to the robustness of the model. The FI score for a feature is defined as the increase in error when the feature is left out of the inferred logic model (Equation 9). The FI scores are not only based on the optimal logic model, but also on other good (suboptimal) logic models that are identified by the ILP solver. (See Methods Section for details.) Across the 72 statistically significant models we found that most features are essential, i.e. have a high FI score, for a specific drug. However, BRAF, CDKN2A and TP53 play an important role in explaining the drug response for many of the drugs in our panel (Supplementary Figure 7). As an example of FI scores, Fig. 3a depicts the FI scores of the 60 gene mutation features for the PI3K/mTOR inhibitor BEZ235. From this panel it is clear that two features, PTEN and PI3KCA, are important in explaining BEZ235 response. More importantly, when sample-specific weights are employed, the FI remains robust across different values of the discretization threshold.

Also across the remaining drugs the use of the continuous output via sample-specific weights resulted in a substantially smaller variation in the FI scores across the binarization thresholds (Fig. 3b). Particularly, the FI scores for the logic models across the three thresholds had an average Pearson correlation coefficient larger than 0.75 for all but two drugs (99% of the drugs), and 106 drugs (75% of the drugs) had a correlation larger than 0.95. This was in stark contrast to the logic models based without the sample-specific weights, where 93 drugs (65%) had a correlation larger than 0.75 and only 44 (31%) had a correlation larger than 0.95. From this observation we conclude that the use of the sample-specific weights based on the continuous output makes the inferred logic models less sensitive to small changes in the dataset, i.e. more robust.

We also assessed the robustness of the logic models across the CV training folds. Here, we observed a similar pattern, although the difference between using and not using sample-specific weights was less pronounced (Supplementary Note 2 and Supplementary Figure 8). Logic models inferred from the randomly sampled subsets of cell lines, i.e. the CV folds, showed more variability in feature importance scores than the models inferred with slight changes in the binarization threshold. This is not surprising, since the in- or exclusion of samples, especially those far away from the binarization threshold, can have a large effect on the optimization function (Equation 1) and thus the inferred optimal logic model. One such example in our dataset is represented by the drug Bicalutamide, which has the poorest CV performance (point in the top right in Fig. 2). Here, the cell line HT-3 has an extremely low IC50, much lower than all other sensitive cell lines (see Supplementary Data 1). This results in a disproportionally large weight for this sample (its weight is 0.21), whereas the total weight of all 657 cell lines, for which IC50s have been obtained, is 1. This analysis hints at an important weakness of our approach: Outliers, i.e. samples with erroneously large or small output values, have a large effect on the inferred logic model. In general, it is thus very important that outliers are detected and removed before performing a LOBICO analysis.

Studying the robustness across different CV folds led us to investigate the number of cell lines needed to effectively run LOBICO. We implemented a subsampling strategy that showed a substantial influence on performance and robustness. Specifically, whereas for 51% of the drugs LOBICO inferred logic models that are robust and predictive when using all cell lines, this number decreased to 25% of the drugs when using 50% of the cell lines (around 300 cell lines). With a subsampling frequency of 10% (around 60 cell lines) only 18% of the drugs had a robust and predictive model (Supplementary Note 3 and Supplementary Figure 9). The balance between the sensitive and the resistant class as well as the classification performance are important factors in determining whether LOBICO can be effectively run for a smaller cell line panel.

Next, we employed a ‘ground truth’ mapping between drugs and mutations to gauge whether the use of continuous output variables led to more accurate models. This mapping, which is based on the annotation of the drug targets and expert knowledge, links 49 drugs to one or more of the 60 gene mutation features (Supplementary Data 2). For each of these 49 drugs, we analyzed the FI scores of the ground truth features. In this case, we used ‘aggregated’ FI scores, which were not just based on the optimal and suboptimal models from the model complexity with the lowest CV error, but also on the other model complexities that have a CV error equal or smaller than the CV error for the single predictor model (*K* = 1, *M* = 1). These aggregated FI scores provide a more comprehensive landscape of the importance of gene mutations in explaining drug response (Fig. 3a).

For 19 of the 49 drugs, the total aggregated FI scores of the ground truth features were not larger than 0.05, both for the logic models with as well as without the sample-specific weights (Fig. 3c). In these cases, the ground truth features did not explain the observed variation in drug response. For 26 of the remaining 30 drugs, the logic models with sample specific weights had larger FI scores for the ground truth features versus only 4 for the logic models without sample specific weights. Moreover, for 7 drugs, the use of the sample specific weights increased the importance of the ground truth features by at least 0.1. These 7 drugs included the PI3K/mTOR inhibitor BEZ235, 2 BRAF inhibitors and 4 small molecule inhibitors that target PDGFRA and KIT mutants, two of which also target FLT3 mutants, according to the ground truth mapping. Amongst the sensitive cell lines for these drugs, those with the smallest IC50s, i.e. the most sensitive ones, indeed harbored mutations in the ground truth features. This explains the increased accuracy of the logic models with sample-specific weights of retrieving the ground truth features.

The use of sample-specific weights is a prominent feature of LOBICO that sets it apart from traditional methods that use binarized data. The results presented in this section demonstrate that the use of the continuous output leads to more robust and accurate logic models.

### LOBICO finds logic models around a user-defined operating point

A binary classifier can make two kinds of mistakes: false positives (Type I errors) and false negatives (Type II errors). The trade-off between the false positive rate (FPR, 1-specificity) and false negative rate (FNR, 1-sensitivity) defines the operating point of the classifier. The practical application of a binary classifier in any domain is determined by its operating point, the prevalence of positives in the population of interest and the costs, financial or otherwise, that are associated with false positives, false negatives and the test itself.

For example, many medical screening tests, which are relatively inexpensive and generally non-invasive, have high sensitivity and are useful for ‘ruling out’ patients that test negative. For example, a mammogram to screen for breast cancer has a sensitivity of 80% and a specificity of 90%^17^. (It should be noted that periodic screening amounts to a large total number of false positives as evidenced by the fact that half of the women screened in the US will receive a false positive mammogram in any 10-year period^18^).

The implementation of LOBICO as an integer programming problem allows one to add constraints to ensure that solutions meet predefined statistical performance criteria, such as a minimum specificity. We have applied lower bounds on the sensitivity and specificity by adding additional constraints to the ILP (see Equations 10 and 11). If a solution is found, the corresponding logic model is guaranteed to meet these constraints. By setting various lower bounds on the sensitivity and specificity we can probe the receiver-operator-characteristic (ROC) space. In that way we can uncover logic models possibly employing different features at different operating points. Note that this analysis is quite different from the classical ROC analysis, where the same model is evaluated with different thresholds on the output parameter.

For the 25 drugs with the lowest CV error in the original analysis, we ran LOBICO with an array of sensitivity and specificity constraints covering the complete ROC space in intervals of 0.05. The optimal solution for each combination of sensitivity and specificity was again determined by CV. Note that the inclusion of the constraints could lead to different model complexities having the smallest CV error. The Pareto front of solutions is formed by the logic models that perform best in terms of the tradeoff between sensitivity and specificity. This front is equivalent to the ROC curve, although the model at each operating point is different. We observed a large variation in the logic models across this curve, both in terms of the genes that were present in the models as well as the model complexity (Supplementary Data 3).

**Figure 4:**
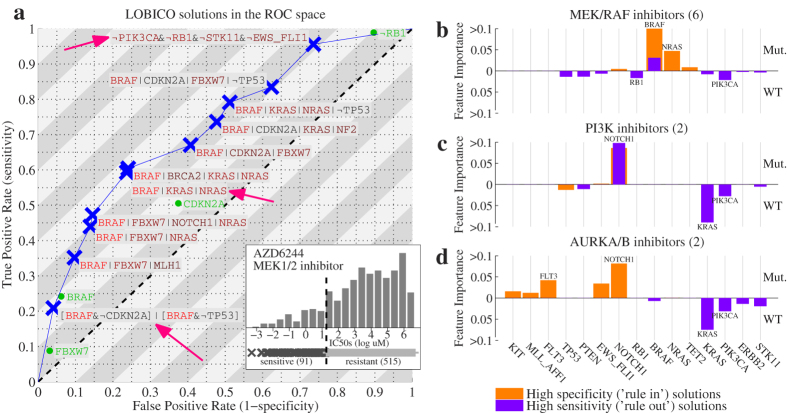
LOBICO finds solutions at different operating points. (**a**) ROC space with LOBICO solutions to explain drug sensitivity to the MEK1/2 inhibitor AZD6244. Blue crosses indicate the TPR and FPR at which the solution was found. The logic formula of the solutions is printed next to the blue crosses. The color of the genes in a formula indicate their FI. Colors range from black (moderately important) to bright red (highly important). For comparison, the best single predictor solutions are visualized in green. Pink arrows point to solutions discussed in the text. The inlay depicts the histogram of IC50s for AZD6244 together with the binarization threshold, which divides the cell lines into 91 cell lines that are sensitive to AZD6244 and 515 that are resistant. (**b**) Average FI scores for a group of 6 MEK/RAF inhibitors (including AZD6244), for high specificity solutions (orange) and high sensitivity solutions (magenta). High specificity solutions were defined as solutions with FPR < 10%. Conversely, high sensitivity solutions were defined as solutions with TPR > 90%. The FI scores of all solutions on the Pareto front (ROC curve) that met these respective criteria across the six drugs were averaged. We distinguished between positive terms, indicating mutations (Mut.) and negated terms, indicating wild-type (WT). The two genes with the highest average FI score as mutants were printed at the top of their FI bar. The two genes with the highest average FI score as wild-types were printed at the bottom of their FI bar. (**c**,**d**) Similar to (**b**), but for a group of two PI3K inhibitors and a group of two AURKA/B inhibitors, respectively.

Figure 4a depicts the logic models in the ROC space for a single drug: MEK inhibitor AZD6244 (brand name Selumetinib), which is currently in clinical trials. Mutations in the gene BRAF clearly play an important role in explaining the drug response in the cell line panel, since BRAF is found in almost all solutions in the ROC space. The most specific solution near the bottom-left of the curve (FPR < 5%) states that BRAF mutants that neither have a CDKN2A nor a TP53 mutation are sensitive to this drug. This high specificity solution can be termed a ‘rule in’ solution, because due to the low FPR, cell lines (or, potentially in the future, patients) that carry a BRAF mutation and are CDKN2A or TP53 wild-type are very likely to respond to this drug. However, this solution explains only slightly more than 20% of the sensitive cell lines (true positive rate (TPR) ≈ 20%). Around the middle of the ROC curve, we observed a 3-input OR solution consisting of BRAF, KRAS and NRAS, the latter two being part of the RAS protein family, which is directly upstream of BRAF, MEK and ERK in the MAPK pathway. This solution has a TPR slightly below 60% and a FPR < 30%. At the top-right of the ROC curve, LOBICO provided a high sensitivity solution (TPR > 95%) that did not contain BRAF. This 4-input AND model predicts that cell lines that are wild-type (WT) for PIK3CA, RB1, STK11 mutations and that do not contain the EWS-FLI1 gene fusion, will respond to the drug. Another way to interpret this solution is that cell lines that have a mutation in either PIK3CA, RB1, STK11 or that contain the EWS-FLI1 gene fusion are resistant to the drug with a very high degree of certainty (FPR < 5% for identifying resistant cell lines). This high sensitivity solution can be termed a ‘rule out’ solution, as cell lines (or potentially patients) that satisfy this rule will most likely not respond to this drug.

Across the 25 drugs, there were 6 inhibitors of MEK or RAF, 2 inhibitors of Phosphoinositide 3-kinase (PI3K) and 2 inhibitors of Aurora kinase (AURK). The logic models across the ROC space displayed high similarity within these three groups (Supplementary Figure 10). Figure 4b–d show the average FI scores of the high-specificity (‘rule in’) solutions and the high-sensitivity (‘rule out’) solutions. As already observed with the MEK-inhibitor AZD6244, mutations in BRAF and NRAS are indicative of drug response with high specificity for the MEK/RAF inhibitors. Conversely, the high importance scores for wild-type RB1 and PIK3CA in the high sensitivity solutions indicate that RB1 and PIK3CA mutants are resistant to these inhibitors. These observations fit within the current ideas on oncogenic signaling, as mutations in the tumor suppressor RB1 and oncogene PIK3CA can lead to uncontrolled cell growth by activating pathways other than the MAPK pathway that these inhibitors are targeting^19^,^20^. For the PI3K and AURK inhibitors we observed that cell lines with a mutation in the transmembrane receptor NOTCH1 are responsive with high specificity. KRAS and, noteworthy, PIK3CA mutants remain resistant to these drugs.

The ability to uncover logic models around predefined operating points is an important requirement for practical application of such models. The implementation of additional constraints in the ILP formulation of LOBICO enabled us to systematically probe the ROC space and uncover logic models for different operating points of interest. To the best of our knowledge, LOBICO is the first method to provide this important capability.

## Discussion

Generally speaking, classification and regression approaches are primarily focused on prediction performance. Today’s state-of-the-art methods use complicated computational frameworks and data transformations to optimize how well the model fits the data. These models have little bearing on the mental model of the person using the approach. The interpretation of the model, if possible at all, is typically limited to a ranked list of important features.

Here, we have presented LOBICO, which, although also optimizing data fit, was developed specifically to produce models that are intuitively understandable. It is important to recognize that performance and interpretation form a trade-off; by constraining a model to be simplistic enough to enable interpretation, one loses the freedom that a complex model can be used to achieve a better representation of the data. LOBICO generates small and robust logic models of binary input features that explain a continuous output.

These models, which fit with standard formal reasoning, allow a researcher to easily assimilate the model with his or her domain. We suggest that the use of interpretable models is crucial in any scientific discipline, where researchers, not machines, generate hypotheses, gain novel insights and decide on further experimentation.

We have demonstrated the application of LOBICO on a large cancer cell line panel by linking logic combinations of gene mutations to drug response. These logic models are easily integrated into current thinking based around stratifying patient populations using individual and combinations of gene mutations. LOBICO is, however, a general framework that can be applied to all research questions that can be described as a mapping from binary input features to a continuous output variable. For example, we successfully applied LOBICO to a cross of two natural yeast strains, the offspring of which were phenotyped for sporulation efficiency (Supplementary Note 4 and Supplementary Figure 11). Importantly, although most (biological) measurements are not binary, they are often amenable to binarization or, at least, a binary interpretation. The use of binary variables allows for standard formal reasoning. That is, if the relation between variables is described using logic, it can be easily understood and reasoned with. An argument against such binarization is the loss of information. In the models that we propose the continuous information of the phenotype variable is retained in the form of sample-specific weights, which, as we have shown, leads to more robust models. This is achieved by splitting the continuous phenotype into a binary vector and a vector with sample-specific weights. We envision that LOBICO has many applications, both within biology as well as in other domains. An important consideration for applying LOBICO, however, is that the dataset at hand has the statistical power that enables one to detect logical relationships that explain the phenotype of interest. For example, most genome-wide association studies of complex traits lack compelling statistical support for epistatic interactions, or the relative magnitude of statistical epistasis is small^21^.

There are certain considerations about the applicability of LOBICO. First, LOBICO solves an NP-hard problem (Supplementary Note 5). We observed that the ILP solver can quickly traverse the search space of logic combinations when searching for small logic models. When looking for large models, on the other hand, the search space quickly explodes and it becomes prohibitive to find an optimal solution. However, we generally restrict LOBICO from inferring large models as they tend to overfit and are less interpretable.

We note that logic regression (LR) implements another search strategy based on simulated annealing, which also achieved a good performance on our dataset (Supplementary Note 6). In comparison, LR cannot guarantee that the obtained solution is optimal nor can it incorporate statistical performance constraints, which is the most relevant scenario for practical applications.

Second, the number of features and correlation structure within the feature space have a large impact on the feasibility of finding an optimal solution within a reasonable time. Large numbers of features drastically increase the search space. Hence, feature selection may be a prudent preprocessing step. When preselecting features, it would no longer be guaranteed that the optimal logic model is found. However, experiments on the cancer cell line panel show that features employed by the optimal logic model have high importance scores both in linear and non-linear regression models (Supplementary Note 7 and Supplementary Figure 12). Thus, these methods might be useful for feature selection when LOBICO is to be applied to datasets with a prohibitively large number of features. Alternatively, expert knowledge can be used to preselect a smaller set of features. An advantage of the latter approach is that these features make sense to the domain experts and will lead to more easily interpretable models.

Correlated features are problematic, not only for LOBICO, but for most (sparse) linear or non-linear classification and regression models. Highly correlated features are effectively interchangeable. In practice, this means that only one feature is selected or that the importance in the model is spread across these features. An additional disadvantage for LOBICO is that the ILP solver will spend a long time deciding which of the correlated features should be part of the optimal solution. Also, from the perspective of interpretation, correlated features can lead to ambiguity and should preferably be dealt with prior to performing a LOBICO analysis.

The implementation of LOBICO as an ILP framework offers substantial advantages and possibilities. Using sophisticated ILP solvers, LOBICO traverses the huge search space of logic combinations fast enough for application to large datasets involving multiple model complexities and cross-validation. An argument against logical analysis is the loss of information when binarizing continuous data. The ILP formulation allows LOBICO to retain the continuous information in the form of sample-specific weights, which, as an added benefit, leads to robust models. Further, constraints guaranteeing predefined statistical performance criteria can be easily incorporated into the ILP framework, giving the logic models direct practical applicability.

A straightforward extension includes adjusting the ILP formulation to prioritize or even require certain features to be part of the inferred logic model. Also, certain combinations of features can easily be allowed or disallowed in the model. Furthermore, biological networks can be captured by ILP constraints, possibly reducing the search space and leading to logic models that reflect network biology. Another avenue for feature engineering for logic models based on cancer mutations would be the inclusion of mutually exclusive or co-occurring mutations^22^,^23^ as single features. These ideas will be explored in future research.

## Methods and Materials

### Cancer cell line dataset

We used a slightly expanded version of a previously published cancer cell line dataset^14^. Specifically, our dataset contained 714 cell lines and 142 drugs instead of 639 and 130, respectively, in the original publication. Although capillary sequencing was performed for 64 genes, our dataset only included the 54 genes for which at least one mutation or copy number aberration was found. A gene was called mutated when it had a point mutation, a small insertion or deletion as determined by capillary sequencing, or when it was highly amplified or homozygously deleted based on copy number arrays. This strategy of lumping together these genomic aberrations to derive mutated genes is identical to the original work^14^. Additionally, we included 6 known oncogenic gene fusions, resulting in 60 mutation features. The drug screening dataset is incomplete, i.e. not all 142 drugs have been screened across all 714 cell lines. In total 81,700 IC50s were measured and 19,688 (19%) were missing values. For the large majority of drugs, IC50s were obtained for between 600 to 700 cell lines. Since we applied LOBICO to each drug separately, cell lines that lack an IC50 were not used. We did not impute missing IC50s. In this work, IC50s were recorded as the natural logarithm of the half-maximal inhibitory μM concentration. Complete data and information on cell lines, the binary mutation matrix, drugs and IC50s are found in Supplementary Data 2 and 4. Additional information on these data is found in^14^,^24^ and on the Genomics of Drugs Sensitivity in Cancer webpages (http://www.cancerrxgene.org/).

### Binarization thresholds of the IC50s

The binarization threshold for each of the drugs was automatically determined using a heuristic outlier procedure, which consists of four steps:

1. *Upsampling*. For a drug, we gathered the IC50s and their confidence intervals of all (*n*) cell lines that were screened for that drug. Then, for each cell line, we took the IC50 and the confidence interval and used this to define a normal distribution. Specifically, the mean of this normal distribution was the IC50 and the standard deviation was the average difference between the IC50 and the lower and upper bound of the confidence interval. We sampled 1,000 data points from this normal distribution and repeated that for all cell lines, leading to 1,000 · *n* data points in total.
2. *Density estimation*. We performed kernel density estimation on the 1,000 · *n* data points using as kernel a normal distribution with bandwidth (standard deviation) 0.5. This kernel density estimate, ***f***, is defined on the interval from the minimum value of the 1,000 · *n* data points, *f*^min^, to the maximum value, *f*^max^, and was normalized such that 
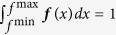
.
3. *Modeling the population of resistant cell lines*. The population of resistant cell lines was modeled as a normal distribution. We used the mode (highest point) in ***f*** as the mean, *μ*, of this distribution. This choice was based on the expectation and previous observation that the large majority of cell lines is resistant to a drug^14^. To compute the standard deviation of this distribution, *σ*, we first computed the parameter *θ*, which marks the divide between sensitive and resistant cell lines. *θ* was computed using the following rules.
  - *θ* is the maximum value, where the derivative ***f***′ is zero, i.e. ***f***′(*θ*) = 0, under the constraints that *θ* < *μ, **f***(*θ*) < 0.8 · ***f***(*μ*) and 
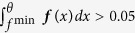
.
  - If no such value exists, *θ* is the maximum value, where the second derivative ***f***′′ is zero, i.e. ***f***′′(*θ*) = 0, as well as ***f***′′′(*θ*) > 0, again under the constraints that *θ* < *μ, **f***(*θ*) < 0.8 · ***f***(*μ*) and 
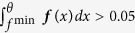
.
  - If no such value exists, *θ* was set to *f*^min^.If *θ* is found under rule i., this indicates bimodality in ***f***, i.e. there is another peak in the distribution to the left of *μ*, which represents the distribution of sensitive cell lines. Similarly, if *θ* is found under rule ii., there is a marked change in the slope of ***f***, which points to the distribution of sensitive cell lines. The standard deviation, *σ*, is computed as the median distance of all data points within the interval [*θ μ*]from *μ*. Finally, the population of resistant cell lines is represented by the Normal distribution *N*(*μ, σ*^2^), which we denote by ***g***.
4. *Evaluating the cumulative normal distribution to find the binarization threshold.* The binarization threshold, *b*, is controlled by parameter *t*. Specifically, *b* is chosen such that the cumulative normal distribution function at *b* equals *t*, i.e. 
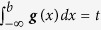
. The default setting for *t* is 0.05. Cell lines with IC50s smaller than *b* are called sensitive, whereas cell lines with IC50s larger than or equal to *b* are called resistant.

Supplementary Figures 13, 14 and 15 provide a visual description of the four-step-procedure to binarize IC50s for three different drugs, where rule i., ii., and iii. were used to find *θ*, respectively. Binarization thresholds (for *t* = 0.05) are found in Supplementary Data 2.

### LOBICO

The goal of LOBICO (Logic Optimization for Binary Input to Continuous Output) is to construct a Boolean logic function that optimally maps the binary variables of an input dataset to a continuous output variable. LOBICO is an extension to the work of Kamath *et al*.^25^, which describes a way to solve the Boolean Function Synthesis Problem (BFSP) using integer linear programming (ILP). See Supplementary Note 5 for a description of the BFSP. Similarly to the BFSP, LOBICO is an NP-hard problem (also in Supplementary Note 5).

LOBICO has three main inputs:

1. **X**, a *N* × *P* binary matrix with *N* samples characterized by *P* binary features *x*_1_, *x*_2_, …, *x*_*P*_. The *P* columns of **X** are denoted as **x**_**1**_, **x**_2_, …, **x**_*P*_.
2. **y**, a *N* × 1 binary vector, which is the binarized version of the continuous output variable.
3. **w**, a *N* × 1 continuous vector with weights for each of the *N* samples.

LOBICO finds an optimal logic function 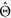 that minimizes the weighted sum of incorrectly inferred samples:


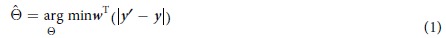


where, **y**′ = Θ(**X**) is a binary vector with the inferred binary labels. The logic functions inferred by LOBICO are in disjunctive normal form (DNF), a generalized logic notation also known as the sum-of-products expression. The complexity of a DNF is determined by two parameters: *K*, the number of disjunctive terms and *M*, the maximum number of selected features per disjunctive term.

### ILP formulation of LOBICO

The ILP formulation to find an optimal 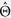 given **X**, **y**, **w**, *K* and *M* employs three variables: First, selection variables are introduced to determine which variable is part of a disjunctive term.


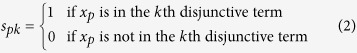



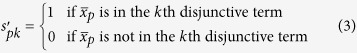


**Figure 5:**
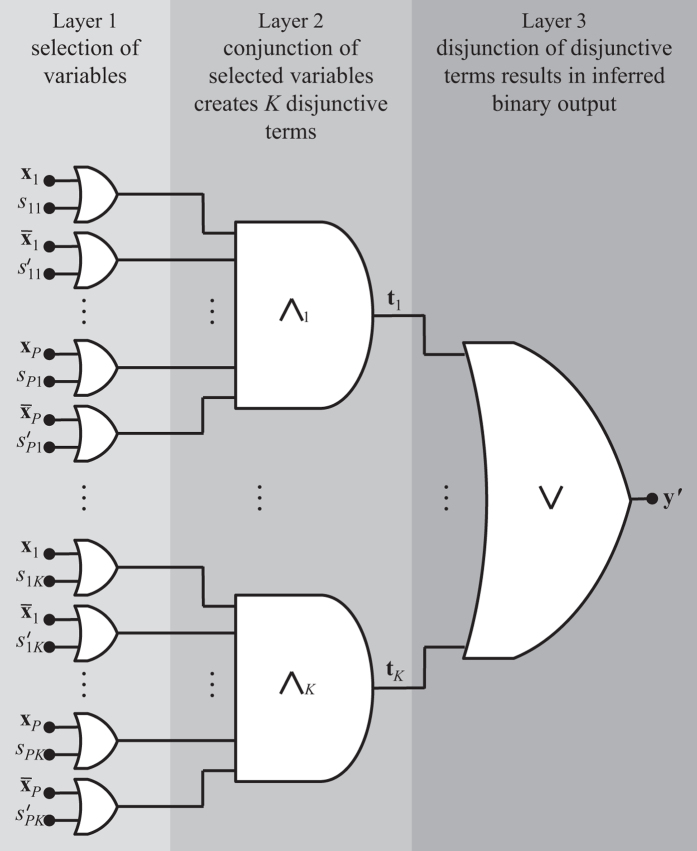
3-layer Boolean circuit representing the structure of the LOBICO ILP formulation. In Layer 1 variables *s*_11_, …, *s*_*PK*_ are used to select the inputs (*x*_1_, *x*_2_, …, *x*_*P*_) that are combined using a conjunction (AND gate) to create the *K* disjunctive terms in Layer 2. These disjunctive terms (the outputs of the AND gates) are represented by variables **t**_1_, …, **t**_*K*_. In Layer 3 the disjunctive terms are combined using a disjunction (OR gate) resulting in the inferred binary output variable **y**′. This figure is adapted from Figure 2.1 in Kamath *et al*.^25^.

Second, auxiliary variables **t**_1_, …, **t**_*K*_, which are vectors of length *N*, are used to represent the disjunctive terms. Third, the disjunctive terms are combined in the final disjunction resulting in the inferred binary output variable **y**′. Figure 5 presents a graphical overview of the variables used in the ILP formulation.

The complete ILP formulation is given below.


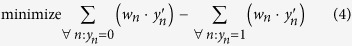


subject to


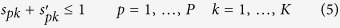



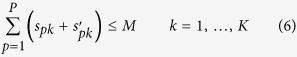



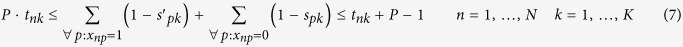



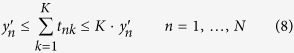


Following is a brief interpretation of the equations. The objective function in Equation 4 is the ILP formulation of Equation 1 and thus represents the weighted sum of incorrectly inferred samples, which should be minimized. The constraints in Equation 5 ensure that *x*_*p*_ and its negation 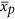 are not simultaneously part of the same disjunctive term. The constraints in Equation 6 ensure that the total number of selected features in a disjunctive term does not exceed *M*. Equation 7 encodes the AND-gates that define the *K* disjunctive terms. The output of the AND-gate *t*_*nk*_ is only 1 for those samples, where the binary data in **X** agrees with all selected features, i.e. 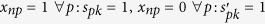. In that case, the two summations in the middle part of the equation add up to *P*, thereby constraining *t*_*nk*_ to 1. Equation 8 encodes the OR-gate, which combines the *K* disjunctive terms in the inferred binary output variable **y**′.

### Feature importance scores

Feature importance (FI) scores are based on the activity measure of variables in Boolean networks^26^,^27^. The importance score of feature *a* is defined as:


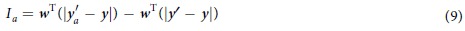


It represents the increase in the weighted sum of incorrectly inferred samples (Equation 1), henceforth called error, when comparing the optimal solution 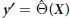, resulting in the error **w**^T^(|**y**′ − **y**|), with 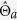, the optimal solution where feature *a* is left out of the model, resulting in the error 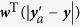.

In practice, leaving feature *a* out of the DNF is achieved by either setting all values of *a* to 1 in the case that feature *a* is part of a disjunctive term with at least one other feature, or setting all values of *a* to 0 in the case that feature *a* is the only feature in the disjunctive term. Features that are not part of the model (DNF) receive an importance score of 0.

See “Application to the cancer cell line panel” below, where we explain how we aggregated feature importance scores across suboptimal solutions, model complexities and CV folds to quantify the importance of gene mutations in explaining drug sensitivity.

### Sensitivity and specificity constraints

The ILP formulation, which includes both the actual output (**y**) and inferred output (**y**′), enables the straightforward implementation of constraints, which guarantee that the ILP solution meets certain performance statistics (provided that a solution actually exists). Specifically, we implemented constraints on minimum sensitivity (also called true positive rate or recall), *TPR*^min^, and minimum specificity, (also called true negative rate or one minus the false positive rate), *TNR*^min^, as follows:


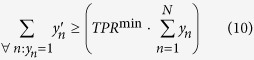



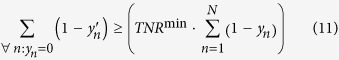


Note that in Equation 10 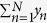 is the number of positives and 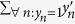 is the number of true positives. In Equation 11 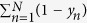 is the number of negatives and 
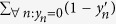
 is the number of true negatives.

Using the notion of sample-specific weights (as represented by **w**), we defined ‘continuous’ versions of constraints on the sensitivity and specificity as follows:


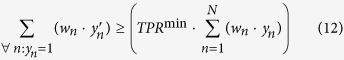



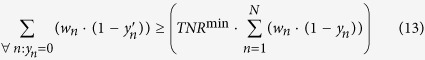


Visualizations of LOBICO solutions in the ROC space using the constraints on the ‘continuous’ sensitivity and specificity are found in Supplementary Data 3.

### Application to the cancer cell line panel

#### LOBICO inputs

LOBICO was applied to each drug in the cancer cell line panel separately. The three main inputs for each LOBICO analysis were:

1. **X**, the *N* × *P* binary mutation matrix with *N* cell lines and *P* = 60 binary gene mutations. *N* differs per drug as not all drugs have been screened across all cell lines. For the large majority of drugs, *N* is between 600 and 700.
2. **y**, the *N* × 1 binary vector indicating whether a cell line is sensitive (1) or resistant (0) to the drug. This vector is obtained by binarizing the continuous IC50s using the binarization threshold, which is determined as explained above.
3. **w**, a *N* × 1 continuous vector with weights for each of the *N* cell lines. **w** is simply the absolute difference between the IC50s and the binarization threshold, normalized per class:

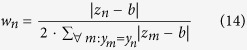


here, *z*_*n*_ is the continuous IC50 of cell line *n* and *b* the binarization threshold for the drug. The normalization ensures that both classes (sensitive and resistant) have the same total weight, i.e. 0.5. Note that 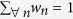. In “Use of continuous output yields more robust and accurate models”, where we compared to the setup in which we did not use the sample-specific weights, 
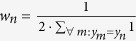
.

#### Statistical performance constraints

For the analyses described in “LOBICO finds logic models around a user-defined operating point”, we systematically varied *TPR*^min^ (Equation 10) and *TNR*^min^ (Equation 11). For all other analyses in the paper, we did not employ the statistical performance constraints.

#### Model complexities

We applied LOBICO with eight different DNF complexities, i.e. we used all combinations of natural numbers *K* and *M* provided that *K* · *M* ≤ 4 (see Table 1).

#### Cross-validation

For each LOBICO analysis in the paper, a stratified 10-fold cross-validation (CV) strategy was employed.

#### Permutation test

We implemented a straightforward statistical test to decide whether an inferred logic model performed significantly better than random. First, we took the inferred class labels (sensitive (1) and resistant (0)) of the inferred logic model when applied to the test folds in the CV. Here, the model complexity of the employed logic model was the optimal complexity according to CV. The binary vector was permuted 1,000,000 times and the error (i.e the sum of the weighted misclassified cell lines) associated with each permutation was computed and recorded. The permuted errors, the mean of which is 0.5, were compared to the original CV error. The permutation test P-value was computed using EPEPT (Enhanced P-value Estimation for Permutation Tests)^28^,^29^. This was done for all 142 logic models. We derived q-values for each of the 142 P-values. A logic model was called statistically significant when both its p-value and q-value (FDR) were smaller than 0.01.

#### Feature importance scores

We computed two types of FI scores for the analysis of the drug response models:

1. *Model-complexity-specific FI scores*. The model-complexity-specific (MCS) importance of feature *f* is the non-zero average of the FI scores of feature *f, I*_*f*_ (Equation 9), obtained from the optimal solution and suboptimal logic models (if any) for particular model complexity (*K* and *M*). Often the suboptimal solutions differ only slightly in error with respect to the optimal model, but contain different features. (See “Solving the ILP using CPLEX” later in this section that explains how suboptimal solutions are found.) This means that the MCS FI score of feature *f* is the mean FI of *f* across all “good” logic models that contain feature *f*. Features that are neither part of the optimal logic model nor part of any suboptimal logic model get a score of zero. In this work, the sample-specific weights were normalized such that errors are between 0 and 1, with random prediction resulting in an expected error of 0.5 (Equation 14). Hence, FI scores are between 0 and 0.5, although the large majority (about 90%) of the non-zero FI scores are smaller than 0.05, and only a small portion (about 5%) are larger than 0.1. The MCS FI scores were used in the robustness analysis (Fig. 3b and Supplementary Figure 8) and the ROC analysis (Fig. 4). They are displayed in Supplementary Data 1 and the upper part of Fig. 3a.
2. *Aggregated FI scores*. The aggregated importance of feature *f* is the non-zero average of the MCS FI scores of all model complexities that have a CV error equal or smaller than the CV error for the single predictor model (*K* = 1 and *M* = 1). Calculating the aggregated FI score involves averaging across the logic models based on all samples as well as the logic models based on the CV training folds. Hence, the aggregated FI scores provide a more comprehensive view of the importance of features in explaining the variation in the output variable. The aggregated FI scores were used in the ground truth analysis (Fig. 3c). They are displayed in Supplementary Data 2 and the lower part of Fig. 3a.

### Solving the ILP using CPLEX

The ILP problems were solved using IBM ILOG CPLEX Optimization Studio V12.4, which is freely available for academic use. Importantly, ILP solvers guarantee optimal solutions (within a numerically small tolerance). Besides the optimal solution, we collected suboptimal solutions using CPLEX’s solution pool. Specifically, up to 25 solutions with a relative gap smaller than 0.1 were gathered using the default pool replacement strategy of replacing the least diverse solutions. We employed a time limit of 1 hour (3600 seconds) per ILP. Less than 1% of the ILP runs did not find the guaranteed optimal solution within this time limit (See Supplementary Figure 16). These solutions were all for 2 × 2 models. In these cases we used the best solutions found thus far. All other parameters were set to their default values.

### LOBICO code availability

LOBICO is implemented in MATLAB and Python and is available through https://github.com/tknijnen/LOBICO.

## Additional Information

**How to cite this article**: Knijnenburg, T. A. *et al*. Logic models to predict continuous outputs based on binary inputs with an application to personalized cancer therapy. *Sci. Rep.* **6**, 36812; doi: 10.1038/srep36812 (2016).

**Publisher's note:** Springer Nature remains neutral with regard to jurisdictional claims in published maps and institutional affiliations.

## Supplementary Material

Supplementary Information

Supplementary Dataset 1

Supplementary Dataset 2

Supplementary Dataset 3

Supplementary Dataset 4

## Figures and Tables

**Table 1:** Overview of the optimal logic model complexity as determined by cross-validation across the set of 142 drugs.

Model complexity			Number of drugs best explained by indicated model complexity (%)	Example			
*K*	*M*	Description		Drug name	Drug target	Optimal logic model	Page in SD1
1	1	Single predictor	21 (15%)	PLX4720	RAF	BRAF	41
1	2	2-input AND	11 (8%)	Paclitaxel	Microtubules	CDKN2A & TP53	64
1	3	3-input AND	9 (6%)	Cytarabine	DNA synthesis	CDKN2A & ¬EGFR & ¬SMAD4	14
1	4	4-input AND	31 (22%)	KIN001-102	Akt1	¬APC & ¬BRAF & ¬EGFR & ¬KRAS	135
2	1	2-input OR	12 (8%)	BEZ235	PI3K, MTORC	PIK3CA | PTEN	55
3	1	3-input OR	17 (12%)	AZD6244	MEK 1/2	BRAF | KRAS | NRAS	60
4	1	4-input OR	23 (16%)	Afatinib	EGFR, ERBB2	EGFR | ERBB2 | JAK2 | SMAD4	39
2	2	2-by-2	18 (13%)	JQ12	HDAC	(CDKN2A & ¬SMAD4) | (¬KRAS & ¬TP53)	90

For each of the 8 model complexities (columns 1–3), this table states the number of drugs best explained by the indicated model complexity (column 4), as well an example of one drug, including drug name (column 5), drug target (column 6), the optimal logic formula (column 7) and the page in Supplementary Data 1 with the standard LOBICO visualization of the inferred logic models for the indicated drug (column 8).

**Table 2:** Overview of the CTRP validation analyses.

Row		Scenario	1		2	
		Training dataset	GDSC344		GDSC370	
		Validation dataset	CTRP344		CTRP344	
			#	%	#	%
1	Training	drugs (models)	37		46	
2		MP models	24	65	34	74
3		predictive models	18	49	25	54
4		predictive MP models	11	61	21	84
5	Validation	drugs (models)	37	100	39	85
6		MP models	19	51	12	31
7		validated predictive models	5	28	5	20
8		validated predictive MP models	4	80	3	60

LOBICO models were trained on 344 cell lines in our data (GDSC344) and validated on the same cell lines in CTRP (CTRP344) (Scenario 1, same cell lines for training and validation), and trained on 370 cell lines unique to our dataset (GDSC370) and validated on CTRP344 (Scenario 2, different cell lines for training and validation). Rows 1 to 4 list results on the training datasets. Specifically, Row 1 lists the number of drugs (or models) that were run on the GDSC dataset. Row 2 lists, of the models in Row 1, the number and percentage for which a multi-predictor model was better than a single predictor model. Row 3 lists, of all models in Row 1, the number and percentage of ‘predictive models’, i.e. the models that showed significant differences in drug response values (IC50s) for the cell lines predicted to be sensitive and resistant. Row 4 lists, of the predictive models, the number and percentage comprising multi-predictor models. Rows 5 to 8 list results on the validation dataset. Specifically, Row 5 lists, of the models run on the training data, the number and percentage that could be run on CTRP. Row 6 lists of the models listed in Row 5, the number and percentage for which a multi-predictor model was better than a single predictor model. Row 7 lists, of the predictive models on the GDSC training data, the number and percentage of ‘validated’ models, i.e. the models that showed significant differences of drug response values (AUCs for CTRP) for the cell lines predicted to be sensitive and resistant. Finally, Row 8 lists, of the validated models, the number and percentage of multi-predictor models.

MP: multi-predictor.
