# Supplementary material for: Logic models to predict continuous outputs based on binary inputs with an application to personalized cancer therapy
Source: Sci Rep. 2016 Nov 23;6:36812. doi: 10.1038/srep36812 (PMC5120272; doi:10.1038/srep36812)
Supplement: Supplementary Information [file srep36812-s1.pdf]

Logic models to predict continuous outputs based on binary inputs with an application to personalized cancer therapy - SUPPLEMENT

Authors

Theo A. Knijnenburg, Institute for Systems Biology, Seattle, US

Gunnar W. Klau, Centrum Wiskunde & Informatica, Amsterdam, The Netherlands

Francesco Iorio, European Molecular Biology Laboratory - European Bioinformatics Institute, UK

Mathew J. Garnett, Wellcome Trust Sanger Institute, UK

Ultan McDermott, Wellcome Trust Sanger Institute, UK

Ilya Shmulevich, Institute for Systems Biology, Seattle, US

Lodewyk F.A. Wessels, Netherlands Cancer Institute, Amsterdam, and The Faculty of EEMCS, Delft University of Technology, Delft, The Netherlands

Abstract

Mining large datasets using machine learning approaches often leads to models that are hard to interpret and not amenable to the generation of hypotheses that can be experimentally tested. We present 'Logic Optimization for Binary Input to Continuous Output' (LOBICO), a computational approach that infers small and easily interpretable logic models of binary input features that explain a continuous output variable. Applying LOBICO to a large cancer cell line panel, we find that logic combinations of multiple mutations are more predictive of drug response than single gene predictors. Importantly, we show that the use of the continuous information leads to robust and more accurate logic models. LOBICO implements the ability to uncover logic models around predefined operating points in terms of sensitivity and specificity. As such, it represents an important step towards practical application of interpretable logic models.

Supplementary Information

Supplementary Note 1 – Validation on the CTRP dataset

We aimed to validate the logic models inferred by LOBICO on our cell line panel by applying these logic models to the drug response data of another cell line panel: the Cancer Therapeutic Response Portal version 2 (CTRP) [1].

CTRP data was obtained from the supplementary information files of the corresponding main publication, available online on the Cancer Discovery journal web-site at: http://cancerdiscovery.aacrjournals.org/content/early/2015/10/14/2159-8290.CD-15-0235/suppl/DC1 (Supplemental Tables S1 – S7, file: 145780_2_supp_3058746_nrhtdz.xlsx). From these files, identifiers of screened cell lines and compounds were extracted and mapped to the cell lines and compound identifiers of our study (from now called GDSC for Genomics of Drug Sensitivity in Cancer). In total, there were 47 overlapping drugs between GDSC and CTRP. For CTRP, the drug response indicator is the AUC, i.e. the area under the drug/cell-line dose response curve. IC50 values were not available for the CTRP study.

Across GDSC and CTRP there are 344 cell lines available in both panels, and 370 cell lines only available in GDSC. We explored two scenarios that both involved a training cohort and a validation cohort: 1) **Train:GDSC344-Validate:CTRP344** LOBICO models were trained on GDSC data of the 344 cell lines (GDSC344) and validated on CTRP data of the same set of 344 cell lines (CTRP344), and 2) **Train:GDSC370-Validate:CTRP344** LOBICO models were trained on GDSC data of the 370 cell lines (GDSC370) and validated on CTRP data of the independent set of 344 cell lines (CTRP344). See **Supplementary Figure 2** for an overview of these datasets.

For each of the two scenarios, we ran LOBICO across the 47 compounds on GDSC using the same settings as for the original analysis. For each drug we selected the best model according to cross-validation (CV) and applied that model to the cell lines dividing them into a group that is predicted to be sensitive and a group that is predicted to be resistant. Then, we performed a t-test comparing these two groups both for the GDSC IC50s as well as for the CTRP AUCs. We also performed a t-test for the best single predictor model. The t-tests were only performed when the groups consisted of at least 5 cell lines. This led to 37 and 39 drugs for scenarios 1 (**Train:GDSC344-Validate:CTRP344)** and scenario 2 (**Train:GDSC370-Validate:CTRP344**), respectively, that we could test within this framework.

For scenario 1 (**Train:GDSC344-Validate:CTRP344)** the best performing models on GDSC validated on CTRP with high statistical significance (**Supplementary Figure 3**). Overall, the p-values for the t-tests on the GDSC IC50s and CTRP AUCs for the 37 models were substantially correlated (**Supplementary Figure 4,** Pearson correlation: 0.94, p = 2.17×10^{-18} , Spearman correlation: 0.31, p = 0.06). Note that lower Spearman correlation indicates that the correlation is mostly driven by the strong models in GDSC and also showed strong validation in CTRP.

Selection of significant t-test p-values using a strict p-value threshold of 0.01/37 (a Bonferroni corrected p-value of 0.01) showed significant overlap between GDSC and CTRP (Fisher exact test, p = 1.3×10^{-3}).

Using a somewhat loose threshold of 1/37 (a family-wise error rate of 1) we identified that 18 of the 37 (49%) drugs led to statistically significant models in the GDSC training cohort, i.e. for these models the cell lines predicted to be sensitive and resistant showed differential drug response using the t-test. 5 of these 18 models (28%) also showed statistical significance in the CTRP344 validation cohort. Importantly, we found that in many cases multi-predictor models outperformed single predictor models (65% for the training cohort and 51% for the validation cohort). See **Table 2** for on overview.

For scenario 2 (**Train:GDSC370-Validate:CTRP344**) we found that the two best performing models on GDSC validated with high statistical significance in CTRP (**Supplementary Figure 5**). Yet, many other good models on GDSC did not lead to a strong prediction of drug response in CTRP; this was the case both for the multi-predictor models and single predictor models inferred from GDSC370. Overall, the p-values for the t-tests on the GDSC IC50s and CTRP AUCs for the 39 models were correlated (**Supplementary Figure 6,** Pearson correlation: 0.84, p = 3.3×10^{-18}). This correlation is mostly driven by the top 2 as evidenced by the much lower Spearman correlation of 0.17, p = 0.31.

Related to this, selection of significant t-test p-values using the strict p-value threshold of 0.01/39 (a Bonferroni corrected p-value of 0.01) showed only a moderately significant overlap between GDSC and CTRP (Fisher exact test, p = 0.06).

Using the more loose threshold of 1/46 for the training cohort and 1/39 for the validation cohort (a family-wise error rate of 1) we identified that 25 of the 46 (54%) drugs led to statistically significant models in the GDSC training cohort, i.e. for these models the cell lines predicted to be sensitive and resistant showed differential drug response using the

t-test. 5 of these 25 models (20%) also showed statistical significance in the CTRP344 validation cohort. Again, we found that in many cases multi-predictor models outperformed single predictor models (74% for the training cohort and 31% for the validation cohort). Of the 5 validated models, 3 were multi-predictor models. Again, see **Table 2** for on overview.

A comparison of scenario 1 and 2 shows that for the independent validation cohort (scenario 2), fewer of the multi-predictor models inferred on the training cohort are more predictive than the best single predictor model (31% in scenario 2 vs. 51% in scenario 1, and 60% for statistically significant models in scenario 2 vs. 80% in scenario 1). This is an indication that, for a considerable number of some drugs, the multi-predictor LOBICO models inferred on one set of cell lines do not generalize to another set of cell lines. Yet, overall these results are encouraging, also given that the sets of 370 and 344 cell lines are substantially different in terms of tissue types and mutation landscape (**Supplementary Figure 2**).

Supplementary Note 2 – Robustness across CV folds

We investigated the robustness of the logic models across the ten CV training folds for each of the 142 drugs. The logic models for a drug were inferred using the model complexity (defined by K and M) selected by CV for that drug in the standard setting, i.e. with the sample-specific weights and t=0.05. The use of the continuous output resulted in a smaller variation in the FI scores across the CV folds (**Supplementary Figure 8a**). Particularly, the FI scores for the logic models across the CV folds had an average Pearson correlation coefficient larger than 0.75 for 113 drugs (80%), and 40 drugs (28%) had a correlation larger than 0.95. In contrast, for the logic models based on binarized data, there were 89 drugs (63%) had a correlation larger than 0.75 and 35 (25%) had a correlation larger than 0.95.

In comparison to changing the binarization threshold (**Figure 3a**), we observed that logic models inferred from the randomly sampled subsets, i.e. the CV folds, showed more variability in the FI scores, and thus a smaller correlation amongst the CV folds. We hypothesized that the inclusion or exclusion of samples, especially those far away from the binarization threshold, can have a large effect on the optimization function (Equation 1), and therefore a large effect on the inferred optimal logic model and the resulting FI scores. To test this hypothesis, we compared the similarity between CV folds with the similarity of the

FI scores derived from the logic models trained on these CV folds. Specifically, for each of the 142 drugs separately, we computed:

1. for each pair of the CV training folds, say a and b, the similarity between the CV folds a and b in the following manner:
    a. We took $\mathbf{w}$, the $N \times 1$ continuous vector with weights for each of the N samples. $\mathbf{w}$ is the absolute difference between the IC50s and the binarization threshold, normalized per class (Equation 14).
    b. We created $\mathbf{w}^a$ and $\mathbf{w}^b$, where $\mathbf{w}^a$ is identical to $\mathbf{w}$, except that all samples that are not part of the training set of a are replaced by 0, and similarly for $\mathbf{w}^b$.
    c. As a metric of the similarility between CV folds a and b, we computed the Pearson correlation coefficient between vectors $\mathbf{w}^a$ and $\mathbf{w}^b$.
2. for each pair of the CV training folds, as a metric of the similarility of the FI scores between the two members a and b, the Pearson correlation coefficient between the FI score vectors derived from the logic models trained on CV folds a and b.

With the 142 drugs and 10-fold CV strategy, this resulted in $142 \times \binom{10}{2} = 6390$ pairwise correlation scores for the similarity in the weight vectors and 6390 pairwise correlation scores for the similarity in the FI scores. We observed a clear relationship between these correlation scores (**Supplementary Figure 8b**). Particularly, pairs of CV folds with a small correlation between the weight vectors often had a small correlation between the FI scores. We observed that in about 5% of the cases the correlation between the weight vectors was quite low, i.e. the correlation coefficient was smaller than 0.7. These are cases, where the two CV folds include (and exclude) different samples with extreme IC50s, i.e. those far away from the binarization threshold. These ‘important’ samples have large weights in the weight vector, and when set to 0 in one of the folds, but not the other, lead to the low correlation scores between the folds. It is thus not surprising that the logic models inferred on these distinct CV folds lead to different FI scores.

This analysis confirmed our hypothesis that the larger variation in FI scores observed across CV folds is due to the inclusion or exclusion of samples with large weights, i.e. those far away from the binarization threshold.

Supplementary Note 3 – Subsampling analysis

Specifically, we randomly sampled from all 714 cell lines 90% to 1% of the cell lines in 13 steps, i.e. 90%, 80%, 70%, 60%, 50%, 40%, 30%, 20%, 15%, 10%, 5%, 2%, 1%, and repeated this 10 times. Then, for each case we ran LOBICO across all 142 drugs using the original settings. We analyzed the CV errors and the FI scores across the repeats and compared the results to the setting when we use all (100%) of the cell lines. Based on the CV errors, FI scores and the permutation test (Methods section), we employed the following 5 criteria to identify 'robust' and 'predictive' models. All criteria must be met in order to call a model robust and predictive.

1. The number of sensitive cell lines must be larger than 10 – This is prerequisite for running LOBICO using 10-fold CV and is also the minimum number of cell lines in the positive (sensitive class). In our cell line panel, for most drugs, the bulk of cell lines are not affected, and only a small percentage (5-15% typically) end up in the class of sensitive cell lines. When subsampling to 50% (~300 cell lines) still 135 (95%) of the models can be run. When sampling 20% and 10% of the cell lines (~125 and 65 cell lines resp.) LOBICO models can only be run for 77 (54%) and 23 (16%) of the models. See **Supplementary Figure 9a**.
2. The CV error must be smaller than 0.4 – The statistical cutoff of FDR<1% and p<0.01 that we used to identify statistically significant logic models using the permutation test coincided with a CV error of approximately 0.4 (see **Figure 2**). Therefore, we used this cutoff to identify predictive models. Without subsampling, i.e. using all cell lines, 72 (51%) of the models are predictive. This percentage remains more or less constant when subsampling. See **Supplementary Figure 9b**.
3. The error across the complete dataset must be smaller than 0.4 – The optimal logic model for each of the subsampling rates and repeats was applied to all cell lines after which the error across the complete cohort was computed. We required this error to be smaller than 0.4 in which case the logic model inferred

using a subset of the samples generalizes over the complete dataset. We observed that the although CV-error remains constant, the error on the complete dataset increases with a smaller subsampling frequency. This is an indication that when using a smaller set of cell lines the inferred logic models do a good job of explaining the drug response for those cell lines, but these models do not generalize across a larger panel. For example, when sampling 10% of the cell lines (~65 cell lines) the percentage of predictive models drops to ~30%. See **Supplementary Figure 9c**.

4. <u>The Pearson correlation of FI scores amongst the CV folds must be higher than 0.7</u> – We observed that the Pearson correlation coefficients of the similarity of FI scores across the 10 CV folds on the complete dataset were higher than 0.7 for most drugs (**Supplementary Figure 8**). Therefore, we used this cutoff to identify robust models in the subsampling analysis. Without subsampling, i.e. using all cell lines, 124 (87%) of the models are robust according to that definition. This percentage remains more or less constant when subsampling. See **Supplementary Figure 9d**.
5. <u>The Pearson correlation of FI scores between the subsampled and complete dataset must be higher than 0.7</u> – The FI scores obtained for the logic models for each of the subsampling rates and repeats were correlated with the FI scores from the logic models inferred across the complete cohort. We required this correlation to be larger than 0.7 in which case the logic models inferred using a subset of the samples are highly similar to the original logic model based on all samples. We observed that correlation of these FI scores decreased quickly with a smaller subsampling frequency. This is an indication that when using a smaller set of cell lines the inferred logic models are different from the original model. For example, when sampling 50% and 20% of the cell lines (~300 and 125 cell lines resp.) 55 (41%) and 32 (28%) logic models met this criterion. See **Supplementary Figure 9e**.

Overall, we observed that subsampling had a substantial influence on performance and robustness as defined by our criteria. Specifically, whereas for 51% of the drugs LOBICO inferred logic models that are robust and predictive when using all cell lines, this number decreased to 25% of the drugs when using 50% of the cell lines (around 300 cell lines). With

a subsampling frequency of 10% (around 60 cell lines) only 4 (18%) of the drugs had a robust and predictive model. Perhaps not surprising, these 4 drugs were amongst the ones with the lowest CV-error on the complete dataset.

In conclusion, LOBICO can also effectively be run on smaller sets of cell lines, but in our panel there was only a relatively small number of drugs for which robust and predictive models were found with much smaller sets of cell lines. The subsampling analysis led to two important insights: First, it is important that the classes in the dataset are not strongly unbalanced; if one of the two classes is too small this leads to non-robust models or even the inability to run LOBICO using CV. Second, only for the highly predictive models, i.e. those with a small CV error, did we find robust and predictive models when using a small set of cell lines. Thus, for smaller sets of cell lines strong effect sizes are necessary to reach significance. This is something that can potentially be tested with univariate tests before running LOBICO.

Supplementary Note 4 – LOBICO on a yeast cross phenotyped for sporulation efficiency

We re-analyzed the genetic linking map of a cross of two natural yeast strains, a strain isolated from the bark of an oak tree that sporulates at 99% efficiency, and a strain originating from a wine barrel that sporulates at only 3.5% [2]. The genetic linkage map consists of 225 loci genotyped in 374 segregants. For each of the 374 recombinant offspring, the sporulation efficiency was measured as a percentage between 0 and 100. Gerke *et al.* [2] used composite interval mapping based on a stepwise regression model to find loci that significantly cosegregated with variation in sporulation efficiency, leading to 5 significant loci, L7-9, L10-14, L13-6, L7-17 and L11-2 (Table 1 in [2]). Next, a second stepwise regression was used to select significant predictors from the five loci and all 2 and 3-way interaction terms involving these five loci. The final model included three significant 2-way interaction effects and one 3-way interaction effect. All these interactions were comprised of combinations of the three most significant individual loci, i.e. L7-9, L10-14 and L13-6 (Table S2 in [2]).

We applied LOBICO to this dataset to evaluate which (logical) interaction effects would be uncovered. The genotype information was straightforwardly transformed into binary predictor variables: Alleles from the oak strain (wine strain) were set to 1 (0),

resulting in a truth table with $n = 225$ loci and $p = 374$ segregants. The sporulation phenotype data was binarized by applying a threshold of 50%. Samples were weighted using the distance to this threshold. No specificity and sensitivity constraints were applied. We employed the eight model complexities also used for the cell line panel analysis, i.e. all combinations of K and M with $K \cdot M \leq 4$.

The largest single effect found in Gerke *et al.*, loci L7-9, is also the best single predictor uncovered by LOBICO (**Supplementary Figure 11**). The two-input AND model found by LOBICO consisted of loci L7-9 and L10-14. This interaction, which is also one of the 2-way interaction effects found in Gerke *et al.* has a much higher specificity and precision than the single locus model, although a smaller recall. Many of the offspring with the highest sporulation efficiency have both the L7-9 and the L10-14 locus from the oak strain. The best model according to CV is a 2-by-2 model, which contains the same three loci as in the interaction effects found in Gerke *et al.*, i.e. L7-9, L10-14 and L13-6. (Actually, the LOBICO 2-by-2 model contained L13-7 instead of L13-6; they are highly correlated. The fourth feature in the 2-by-2 is L7-11, which is highly correlated to L7-9.) Thus, LOBICO finds interactions between the same three loci as the regression model employed by Gerke *et al.*.

It is important to point out that LOBICO uncovered these interactions using the complete dataset of 225 loci, and not by first filtering on individual features as was done in Gerke *et al.*. Surely, the (biological) interpretation of the logic model and the additive linear model is quite different. We would argue that the logic model is more intuitive and sensible than the linear model.

Supplementary Note 5 – Explanation of the Boolean Function Synthesis Problem and proof that LOBICO is NP-complete

The Boolean Function Synthesis Problem (BFSP) is a particular type of Boolean Satisfiability Problem, where the goal is to find an algebraic sum-of-products expression for an incompletely specified Boolean function $\Phi:\{0,1\}^n \rightarrow \{0,1\}$. The sum-of-products expression is also called a disjunctive normal form (DNF), i.e. a disjunction of conjunctions. Each Boolean function can be expression in DNF. An element of the domain of Φ is called a minterm of Φ. The set of minterms for which Φ evaluates to 1 (resp. 0) is called the $\mathrm{ON\text{-}set}$ (resp. $\mathrm{OFF\text{-}set}$). An incompletely specified Boolean function is one for which

$|\mathrm{ON\text{-}set}| + |\mathrm{OFF\text{-}set}| < 2^n$. **Supplementary Figure 17** displays an incompletely specified Boolean function with $n = 10$ input variables, $\boldsymbol{x}_1, \boldsymbol{x}_2, ..., \boldsymbol{x}_{10}$ and an output variable $\boldsymbol{y}$.

The number of rows in the Boolean truth table is given by p ($p = |\mathrm{ON\text{-}set}| + |\mathrm{OFF\text{-}set}|$), and is 40 in this case ($40 << 2^{10}$). Note that in most biology applications, Φ is incompletely specified. The sought after algebraic expression is a Boolean DNF expression that evaluates to 1 for all minterms in the $\mathrm{ON\text{-}set}$ ($\mathrm{OFF\text{-}set}$) and to 0 for all minterms in the $\mathrm{OFF\text{-}set}$. Formally, the problem is as follows: Given an $\mathrm{ON\text{-}set}$ and an $\mathrm{OFF\text{-}set}$ of minterms that characterize a Boolean function Φ, find a DNF of Φ with maximally K disjunctive terms having each maximally M variables. The corresponding decision problem is NP-complete [3].

The decision version of LOBICO is as follows: Given inputs $\mathbf{X}$, $\mathbf{y}$, $\mathbf{w}$, K, M and a parameter L, does there exist a logic function $\hat{\Theta}$ expressed in DNF(K, M), i.e. a DNF having at most K disjunctive terms and M literals, such that the weighted sum of incorrectly inferred samples as described in Equation 1 is less than or equal to ε? Cleary, the problem is in NP. It is easily shown that this problem is also NP-complete by the following polynomial-time reduction from BFSP: Given an instance of BFSP we construct an instance for LOBICO by deriving $\mathbf{X}$ from the minterms, $\mathbf{y}$ from the $\mathrm{ON\text{-}set}$ and $\mathrm{OFF\text{-}set}$ and by setting $\mathbf{w}$ to **1**, i.e. $w_n = 1 \quad \forall n$. Now, BFSP can be satisfied if and only if LOBICO has a solution with an error of $\varepsilon = 0$. Since the BFSP decision problem is NP-complete, the LOBICO decision problem is also NP-complete.

Supplementary Note 6 – Comparison with logic regression

Logic regression (LR) [4,5] is a generalized regression methodology that can be applied to data with binary predictors, although continuous predictors are also allowed. The goal of LR is to find linearly weighted logic combinations of the original predictors that explain a continuous response variable or class label. We configured the implementation of LR, i.e. the R-package 'logreg', such that it infers logic models with a predefined model complexity. Specifically, logreg has a scoring function for classification using sample-specific weights, which we used to give it the same objective function as LOBICO (Equation 1). Also, logreg

can be configured to output a single logic model (a tree) with predefined logical operators and size. (See below for experimental details.)

We ran LR for each of the 142 drugs in the cancer cell line panel using the model complexity (defined by *K* and *M*) selected by CV for the associated drug when using LOBICO. LR was run on the same computers (Intel(R) Xeon(R) CPU, E5645, 2.40GHz, 6 cores) as LOBICO and was given the same amount of CPU time (**Supplementary Figure 18a**). Then, we evaluated the logic formulas inferred by LR. Specifically, we looked at the Jaccard similarity of the selected predictors in the inferred LOBICO and LR models. (In computing the Jaccard similarity negated terms, e.g. ¬TP53, are treated as separate predictors from their positive equivalents.) For models with *K*=1 and/or *M*=1, a Jaccard similarity of 1 indicates that the exact same logic formula was found. For *K*=2 and *M*=2 (the 2x2 models) this is not necessarily the case, but we were not able to restrict logreg to output a DNF with *K*=2 and *M*=2 anyway. For example, for the drug 'MG-132' LOBICO inferred the 2x2 '(¬MYC & RB1) | (¬PIK3CA & ¬TP53)', whereas LR inferred '(((¬TP53) or (RB1 or NOTCH1)) and (¬PIK3CA))'. The LR model is clearly not a DNF with *K*=2 and *M*=2.

Overall, LR found the same (optimal) logic formulas as LOBICO (**Supplementary Figure 18b**). The main exception is the 2x2 model (*K*=2, *M*=2), but this is because of the reason mentioned above. For the 4-input OR models (*K*=4, *M*=1) we observed four cases where the logic formulas differed between LOBICO and LR. Upon further inspection, we found that in these cases the formula inferred by LR had the same (optimal) error as the LOBICO solution. These LR solutions were present in LOBICO's solution pool, i.e. they were part of the set of (sub-)optimal solutions output by LOBICO. (See experimental details below.)

In conclusion, when logreg parameters are properly set, LR can find the optimal solution when given the same amount of time that was necessary for LOBICO to find the optimal solution on the cancer cell line dataset. Potentially, LR finds this solution faster than LOBICO on this dataset. It is however important to point out that LR cannot guarantee that the obtained solution is optimal, and it is known that ILP solvers spend a long time proving that the found solution is indeed optimal. In future work, we will investigate the performance of LR and LOBICO on other (larger) datasets, and assess how the two methods can be used in parallel to find optimal solutions faster. For example, we will investigate whether LR can be used to identify initial starting models for LOBICO.

Importantly, LR cannot incorporate statistical performance constraints, such as sensitivity and specificity (Equations 10 - 13), which we assert is the preferred and, in practice, most relevant scenario for LOBICO inferences. Additionally, in contrast to LR, LOBICO can output the pool of (sub-)optimal solutions, which we used to measure feature importance.

Experimental details: LR was run using the R-packing logreg (version 1.5.8). We used simulated annealing as this search algorithm gave the best results. We followed the logreg's documentation to set the upper and lower temperature of the annealing chain based on experiments with the cancer cell line panel. The number of iterations was set, such that the total CPU time spent on solving the problem was comparable to the CPU time that LOBICO needed to find the optimal solution (**Supplementary Figure 18a**). The simulated annealing parameters were set as follows (R-code):

```
myanneal <- logreg.anneal.control(start = 1, end = -5, iter = T*200000, update = T*20000)
```

To infer a LR model with the same model complexity as LOBICO, we made sure that for 2-, 3- and 4-input AND models only AND operators were allowed. Similarly, for 2-, 3- and 4-input OR models only OR operators were allowed. For 2x2 models we allowed both AND and OR operators. The parameters of the logic 'tree' shape were set as follows (R-code):

```
if (K>M) mytreecontrol <- logreg.tree.control(opers=3) else mytreecontrol <- logreg.tree.control(opers=2)
if (K==2&M==2) mytreecontrol <- logreg.tree.control(opers=1)
```

LR was run to output one logic tree (`ntrees=1`), where the maximum number of leaves was set to K x M (`nleaves=K*M`). In the R-code below `Y` is $\mathbf{y}$, `X` is $\mathbf{X}$ and `W` is $\mathbf{w}$ as used in the **Methods Section** and Equation 1. LR was run as follows (R-code):

```
q<-logreg(resp=Y, bin=X, wgt=W, type=1, select=1, ntrees=1, nleaves=K*M,anneal.control = myanneal, tree.control = mytreecontrol)
```

Supplementary Note 7 – Comparison with sparse linear regression and Random Forests

We compared the LOBICO models obtained on the cancer cell line panel with Elastic Net [6], a sparse linear regression model, and with Random Forests regression [7], a non-linear regression model. Specifically, for the 25 drugs with the lowest CV error in the original analysis, we compared the model-specific FI scores (of the model complexity selected by CV)

with the regression weights inferred by Elastic Net (EN) and the importance scores inferred by Random Forests (RF).

We observed a large concordance between LOBICO's FI scores and the EN regression weights (**Supplementary Figure 12a**). In the EN models, the large majority (63%) of all regression weights across the 60 features and 25 drugs were 0. Importantly, all of the important features according to LOBICO (FI>0.05) had a non-zero regression weight in EN. Moreover, the smallest EN regression weight for which the corresponding LOBICO FI was larger than 0.05, was 0.2752 (blue line in **Supplementary Figure 12a**), which was in the tail of the EN weights.

Similarly for RF, we observed a high degree of correlation between LOBICO's FI scores and the RF importance scores (**Supplementary Figure 12b**). The important features according to LOBICO (FI>0.05) also had a high RF importance score. The smallest RF importance score for which the corresponding LOBICO FI was larger than 0.05, was 0.014, which marked the 82% percentile of the RF importance scores.

We note that it is not possible to do a direct comparison in terms of predictive performance, because LOBICO and EN/RF use different error measures. That is, LOBICO's error is the weighted sum of incorrectly inferred samples, where the weight is the distance from a cell line's IC50 to the discretization boundary - an L1 norm for misclassified samples. This error is different from the L2 norm (least squares) or L1 norm across all samples used in (RF) regression models. At the same time, LOBICO's goal is not to be better than other methods in terms of prediction performance, but instead to create interpretable models with good performance.

Experimental details: For EN, we used the MATLAB 'lasso' function with an alpha (mix between L2 and L1 penalty) of 0.5, 10-fold CV and sample weights $\mathbf{w}$, the $N \times 1$ continuous vector with weights for each of the N samples (Equation 14). For RF, we employed the Random Forests implementation for MATLAB v0.02 downloaded from http://code.google.com/p/randomforest-matlab/. The RF regression models were run with 1000 trees each and default settings for the other parameters were used. The reported importance scores represent the mean decrease in accuracy. To accommodate the different sample weights, we created (for each drug) a dataset of 10,000 samples, which were randomly drawn with replacement from the original dataset, where the probability of being drawn was proportional to the sample weights in $\mathbf{w}$.

Citations

1 Seashore-Ludlow, B. *et al.* Harnessing connectivity in a large-scale small-molecule sensitivity dataset. *Cancer discovery* **5**, 1210-1223 (2015).

2 Gerke, J., Lorenz, K. & Cohen, B. Genetic interactions between transcription factors cause natural variation in yeast. *Science* **323**, 498 (2009).

3 Gimpel, J. F. A method of producing a Boolean function having an arbitrarily prescribed prime implicant table. *Electronic Computers, IEEE Transactions on*, 485-488 (1965).

4 Ruczinski, I., Kooperberg, C. & LeBlanc, M. Logic regression. *Journal of Computational and Graphical Statistics* **12**, 475-511 (2003).

5 Kooperberg, C. & Ruczinski, I. Identifying interacting SNPs using Monte Carlo logic regression. *Genetic epidemiology* **28**, 157-170 (2005).

6 Zou, H. & Hastie, T. Regularization and variable selection via the elastic net. *Journal of the Royal Statistical Society: Series B (Statistical Methodology)* **67**, 301-320 (2005).

7 Breiman, L. Random forests. *Machine learning* **45**, 5-32 (2001).

Supplementary Figures

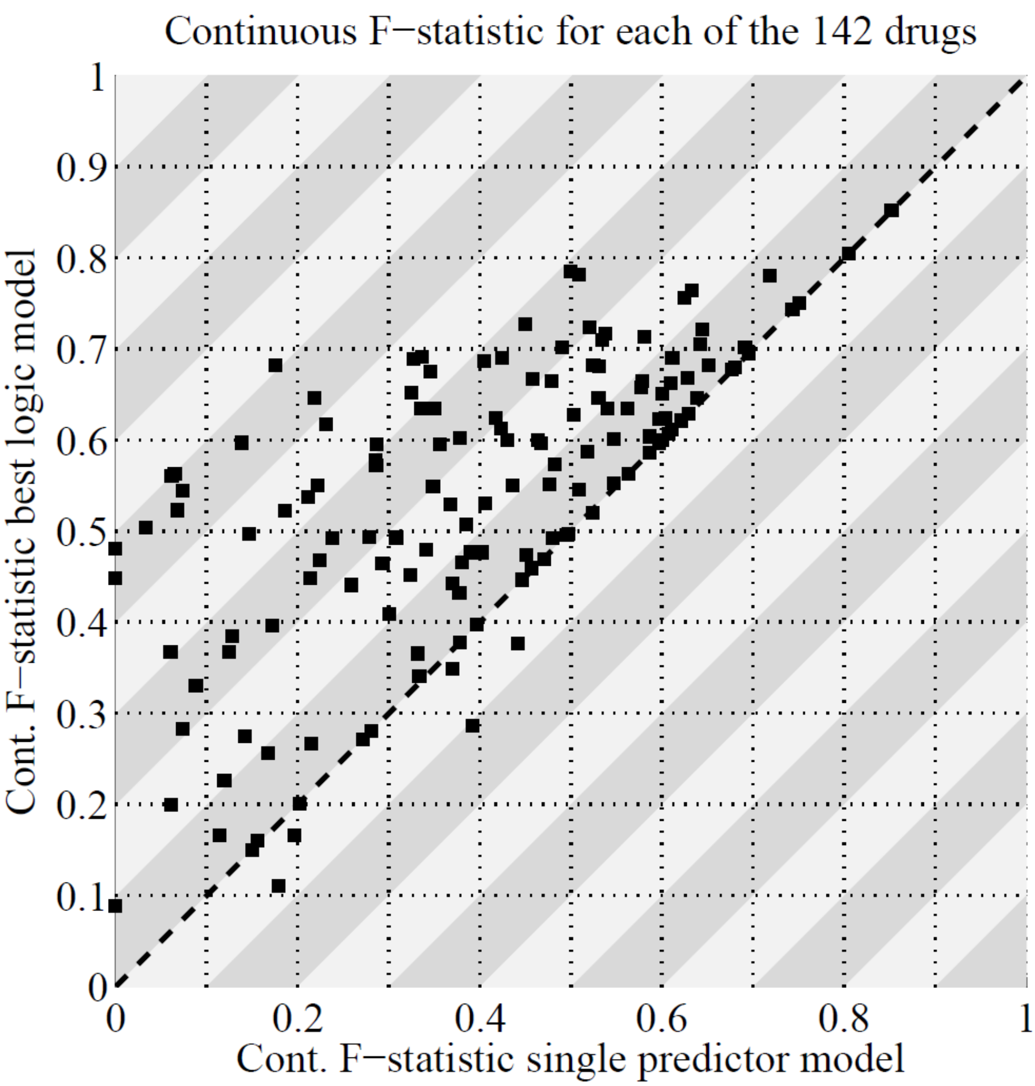

Supplementary Figure 1 | Multi-predictor models outperform single predictor models
Scatter plot with the continuous F-statistic for single predictor models (x-axis) and the best (lowest CV error) multi-predictor model (y-axis). Each point represents one of the 142 drugs. The continuous F-statistic is the defined as the harmonic mean of the continuous recall and continuous precision. By analogy to Equations 12 and 13, the continuous recall and continuous precision are defined as $\mathrm{R_c} = \frac{\sum_{\forall n: y_n = 1} (w_n \cdot y'_n)}{\sum_{n=1}^{N} (w_n \cdot y_n)}$ and $\mathrm{P_c} = \frac{\sum_{\forall n: y_n = 1} (w_n \cdot y'_n)}{\sum_{n=1}^{N} (w_n \cdot y'_n)}$. The continuous F-statistic uses the sample-specific weights that LOBICO uses in its optimization and is therefore a better performance measure than the standard F-statistic. Similarly, to the CV error depicted in **Figure 2**, the continuous F-statistic was computed on the inferred class labels of the samples in the test sets.

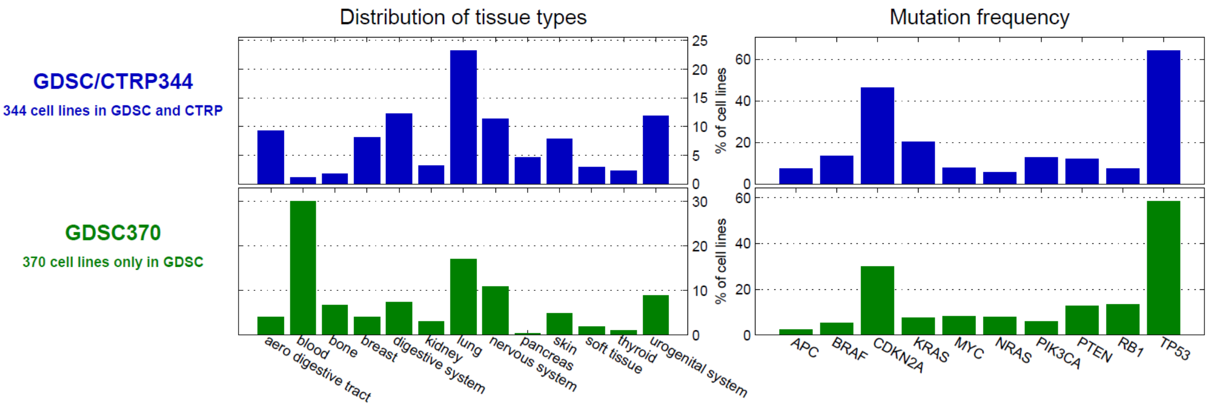

Supplementary Figure 2 | Overview of the training and validation cohorts of the GDSC and CTRP

Bar graphs showing the distribution of tissue types **(left)** and mutation frequency of the ten most frequently mutated genes **(right)** for the 344 cell lines available in both the GDSC and CTRP (GDSC/CTRP344) **(top)** and for the 370 cell lines only available in GDSC (GDSC370) **(bottom)**.

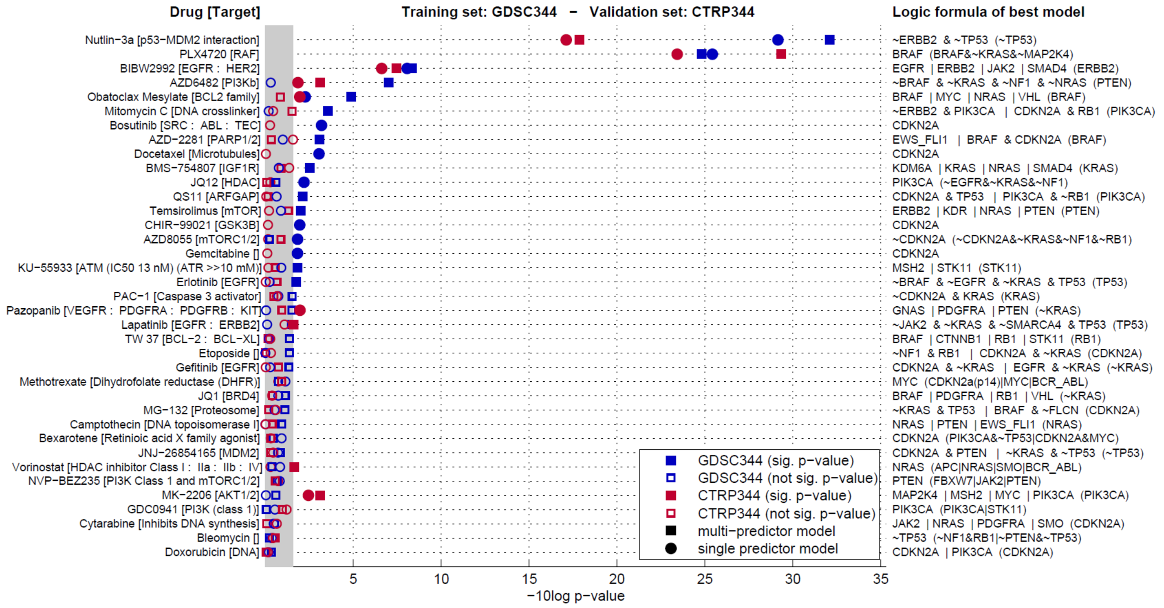

Supplementary Figure 3 | Ordered t-test p-values for GDSC and CTRP for scenario 1 - Train:GDSC344-Validate:CTRP344

LOBICO models were trained on GDSC data of the 344 cell lines (GDSC344) and validated on CTRP data of the same set of 344 cell lines (CTRP344). The scatter plot depicts the -log10 p-values for t-tests that quantify the difference between cell lines predicted to be sensitive and resistant according to LOBICO. In case the best model is a multi-predictor model, the -log10 p-value for the best single predictor model is also depicted, and the single-predictor formula is stated in parentheses behind the formula of the multi-predictor model. In case the single-predictor model led to a lower p-value, yet according to CV the multi-predictor was better, the formula of the multi-predictor model is stated between parantheses. The 37 drugs are sorted based on the t-test p-value derived from the GDSC344 IC50s. P-values are considered significant at p<0.027 (1/37).

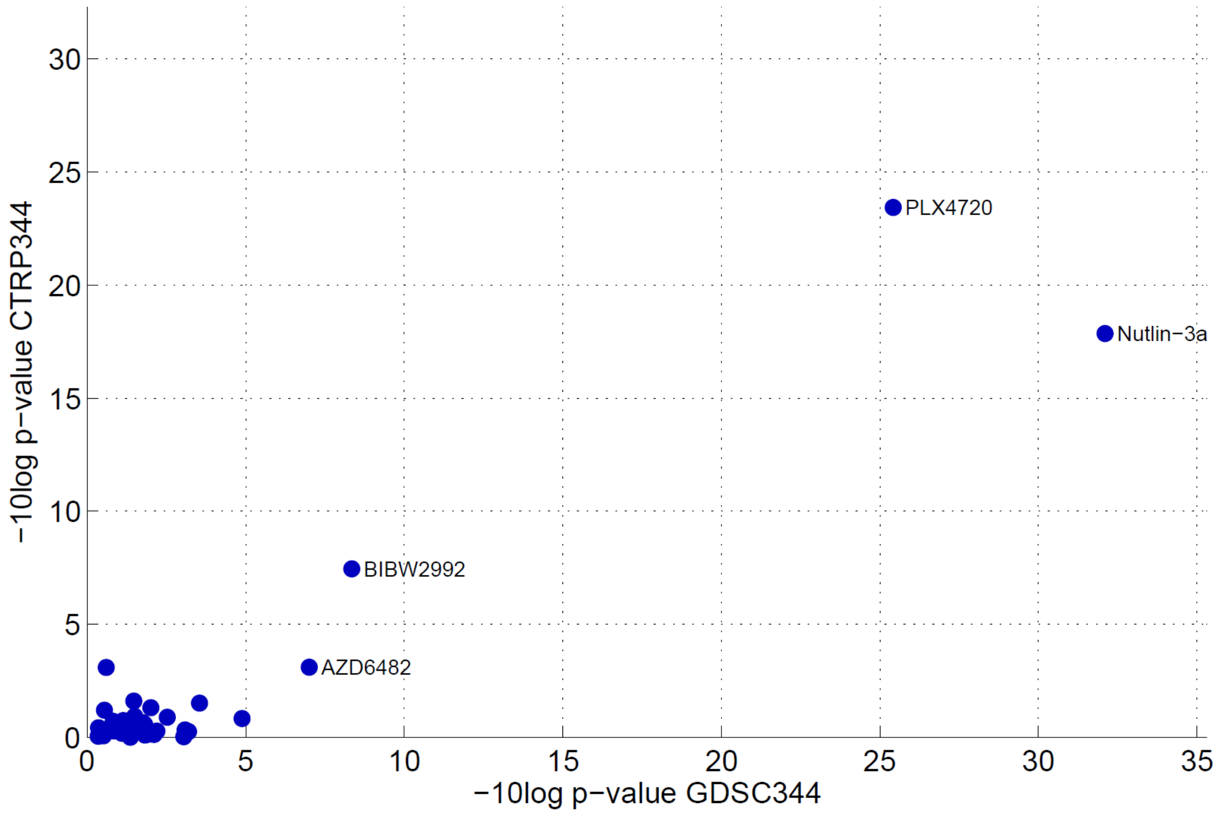

Supplementary Figure 4 | Comparison of t-test p-values for GDSC and CTRP for scenario 1 - Train:GDSC344-Validate:CTRP344

LOBICO models were trained on GDSC data of the 370 cell lines (GDSC344) and validated on CTRP data of the same set of 344 cell lines (CTRP344). The scatter plot depicts the -log10 p-values for t-tests that quantify the difference between cell lines predicted to be sensitive and resistant according to LOBICO. The x-axis depicts p-values for the difference between these two groups based on the IC50s within GDSC344. The y-axis depicts p-values for the difference between these two groups based on the AUCs within CTRP344. Drugs with a p-value lower than 10^{-5} are annotated.

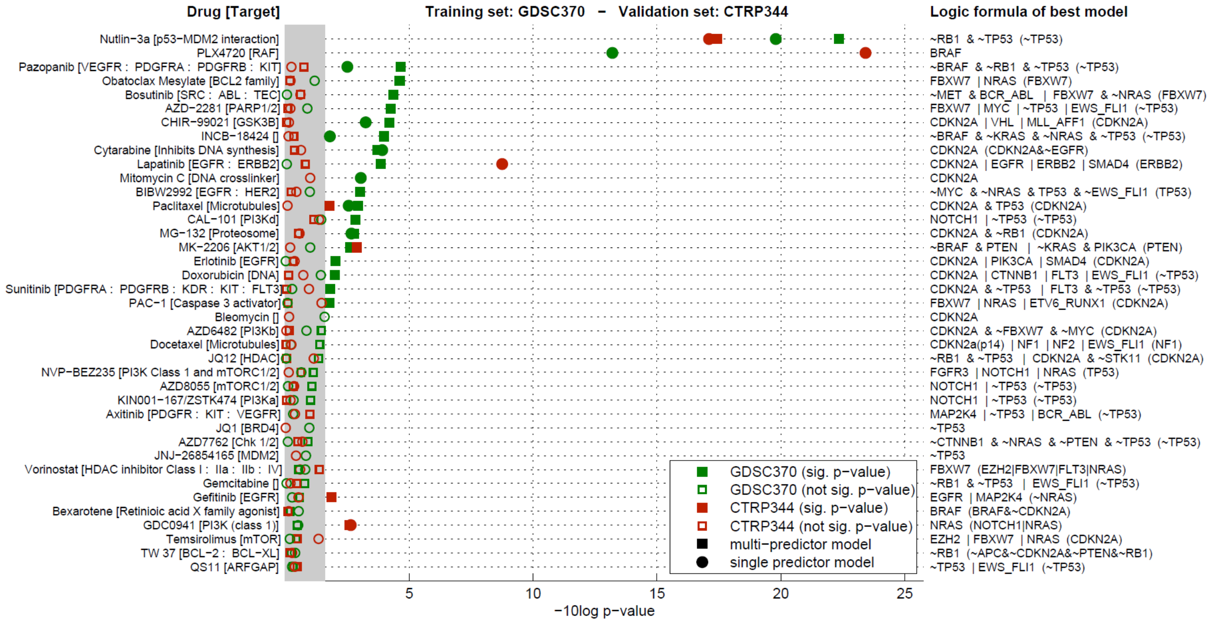

Supplementary Figure 5 | Ordered t-test p-values for GDSC and CTRP for scenario 2 - Train:GDSC370-Validate:CTRP344

LOBICO models were trained on GDSC data of the 370 cell lines (GDSC370) and validated on CTRP data of the independent set of 344 cell lines (CTRP344). The scatter plot depicts the -log10 p-values for t-tests that quantify the difference between cell lines predicted to be sensitive and resistant according to LOBICO. In case the best model is a multi-predictor model, the -log10 p-value for the best single predictor model is also depicted, and the single-predictor formula is stated in parentheses behind the formula of the multi-predictor model. In case the single-predictor model led to a lower p-value, yet according to CV the multi-predictor was better, the formula of the multi-predictor model is stated between parantheses. The 39 drugs are sorted based on the t-test p-value derived from the GDSC370 IC50s. P-values are considered significant at $p<0.026$ (1/39).

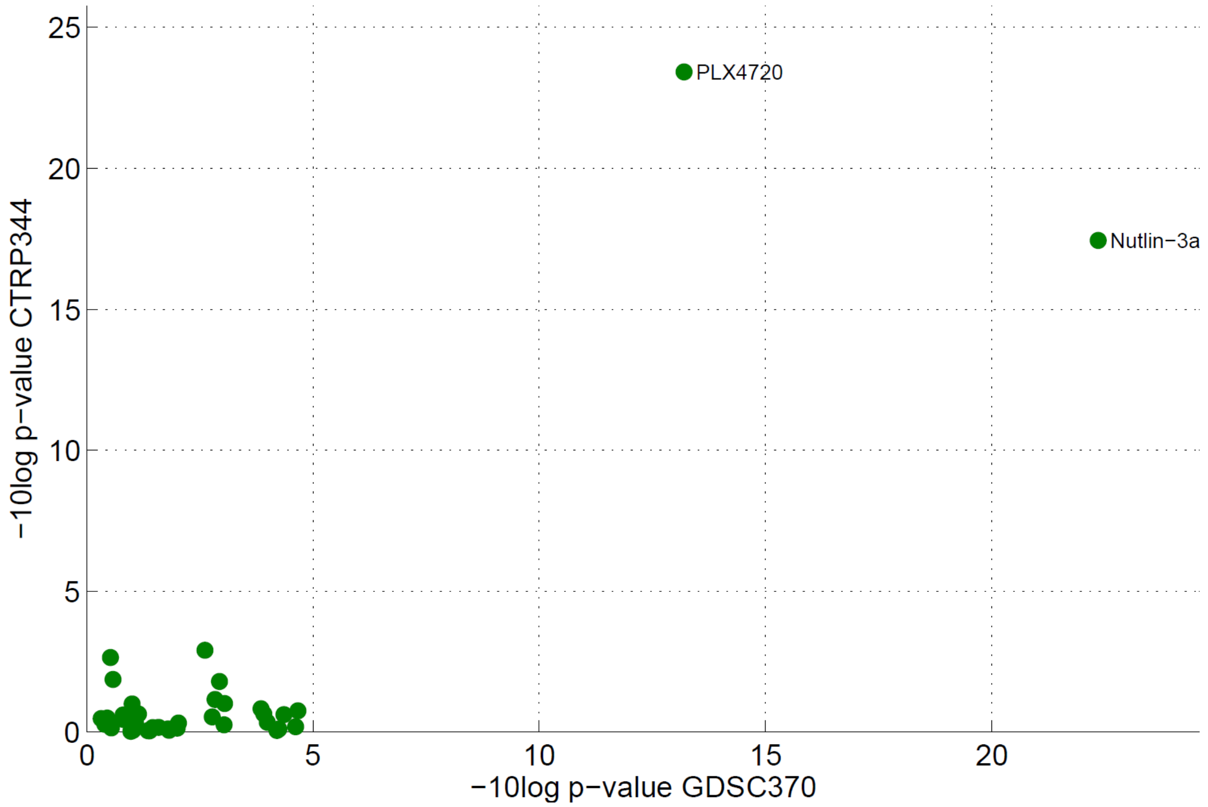

Supplementary Figure 6 | Comparison of t-test p-values for GDSC and CTRP for scenario 2 - Train:GDSC370-Validate:CTRP344

LOBICO models were trained on GDSC data of the 370 cell lines (GDSC370) and validated on CTRP data of the independent set of 344 cell lines (CTRP344). The x-axis depicts p-values for the difference between these two groups based on the IC50s within GDSC370. The y-axis depicts p-values for the difference between these two groups based on the AUCs within CTRP344. Drugs with a p-value lower than 10^{-5} are annotated.

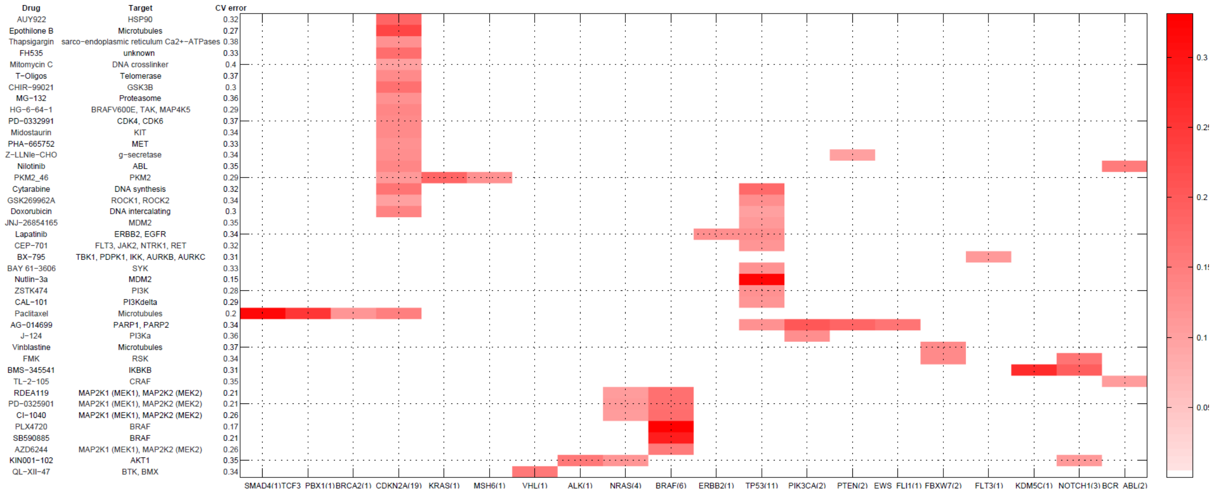

Supplementary Figure 7 | Essential gene mutation features for explaining drug response. Of the 72 statistically significant logic models at FDR<1% and p<0.01, we selected those for which at least one gene mutation feature had a FI score of 0.1 or higher. The statistical cutoff of FDR<1% and p<0.01 that we used to identify statistically significant logic models coincided with a CV error of approximately 0.4 (see **Figure 2**). Since the FI score for a feature is the increase in error when the feature is left out of the inferred logic model, and the randomly expected CV error is 0.5, we classified gene mutation features with a FI score larger than 0.1 as 'essential' features for explaining the drug response. This heatmap visualizes the FI score of those features with the drugs on the rows and the features on the columns. The number in parentheses behind the gene labels indicates the number of drugs for which these genes had an FI score larger than 0.1. These results show that most gene mutations are essential for not more than one drug, but that BRAF (6), CDKN2A (19) and TP53 (11) play an important role for many of the drugs in our panel.

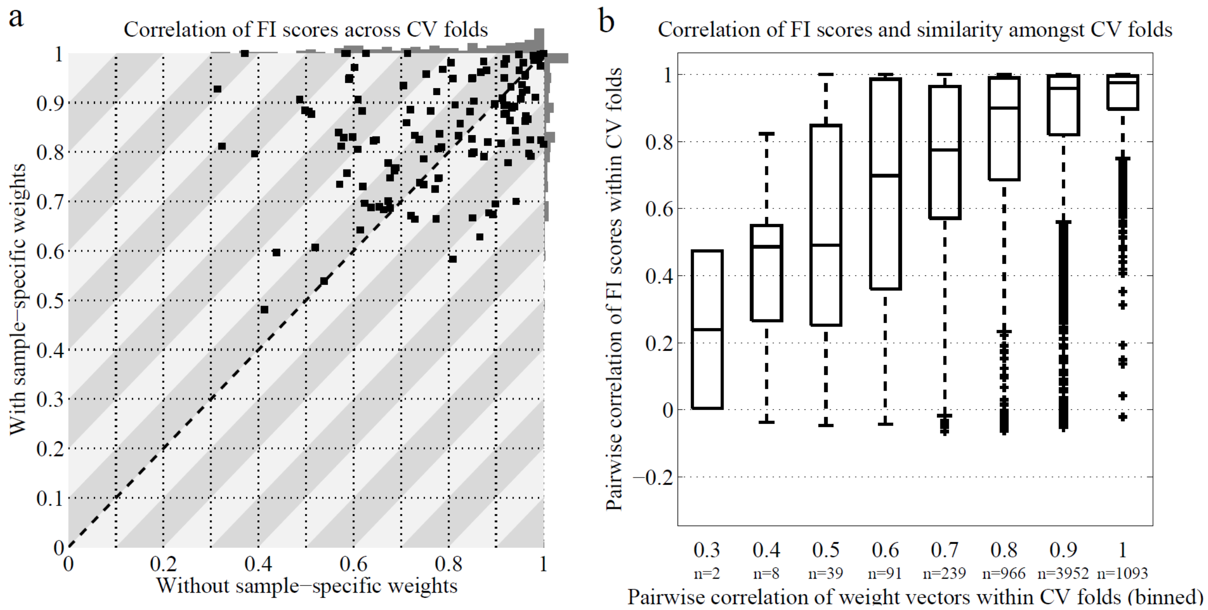

Supplementary Figure 8 | Robustness across CV folds

a) Scatter plot with the average Pearson correlation coefficients of the similarity of FI scores across the 10 CV folds for inferred logic models without (x-axis) and with (y-axis) the sample-specific weights. Each point represents one of the 142 drugs. The correlation scores are computed using the model-complexity-specific FI scores. The grey bars on top and to the right of the scatter plot represent histograms of these correlation scores for models without and with the sample-specific weights, respectively. **b)** Boxplot comparing the pairwise correlation of weight vectors between CV folds (x-axis) with the pairwise correlations of FI scores between the same CV folds (y-axis). The pairwise correlation of weight vectors were binned by rounding the correlation to the nearest decimal. The number of correlations per box is indicated below the box.

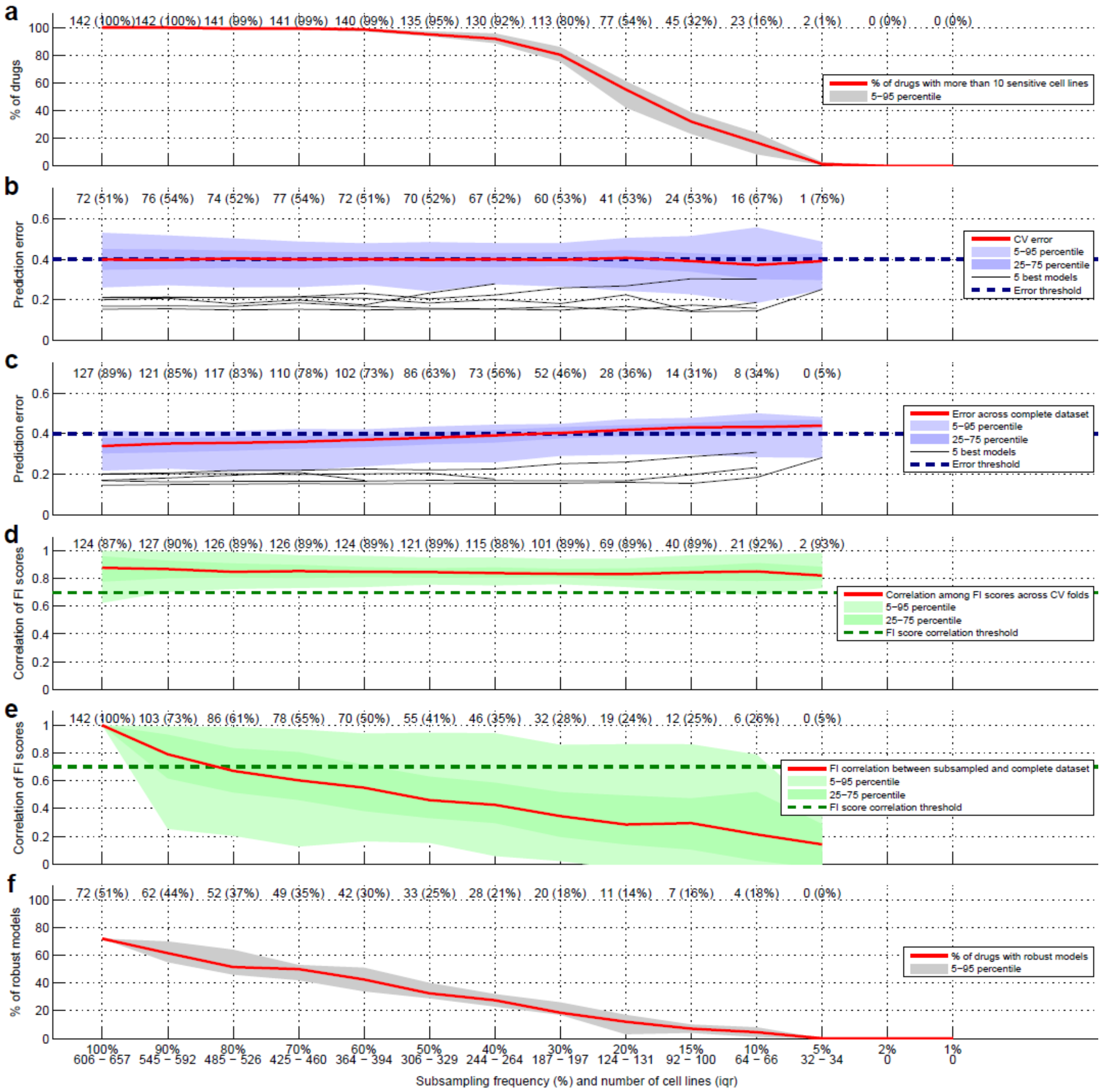

Supplementary Figure 9 | Subsampling analysis to identify robust logic models with fewer cell lines

LOBICO models were run on randomly selected sets of cell lines for an array of subsampling frequencies (x-axis). The subsampling frequency and interquartile range of the number cell lines associated with the subsampling frequency is found in the bottom of the figure. From top to bottom: **a)** The percentage of drugs for which LOBICO models could be run, i.e. for which the number of sensitive cell lines was 10 or greater. Percentile values show variation across the repeats. Absolute numbers and percentages are stated in the top of the plot. **b)** The CV-error. Percentile values show variation across the repeats and across drugs. Average number and percentage of drugs with a CV-error lower than the threshold of 0.4 are stated in the top of the plot. Note that the percentages are based on the number of drugs that can be run for a particular subsampling frequency (a), not based on all 142 drugs. This is also the case for c), d), e) and f). **c)** The error across the complete dataset based on the logic models inferred from the subsampled datasets. Percentile values show variation across the repeats and across drugs. Average number and percentage of drugs with an error lower than the threshold of 0.4 are stated in the top of the plot. **d)** The Pearson correlation among the FI

scores across the CV folds. Percentile values show variation across the repeats and across drugs. Average number and percentage of drugs with a FI score correlation larger than the threshold of 0.7 are stated in the top of the plot. **e)** The Pearson correlation between the FI scores of the logic models from the subsampled datasets and the complete dataset. Percentile values show variation across the repeats and across drugs. Average number and percentage of drugs with a FI score correlation higher than the threshold of 0.7 are stated in the top of the plot. **f)** The percentage of robust and predictive models, i.e. those that meet all criteria. Percentile values show variation across the repeats. Absolute numbers and percentages are stated in the top of the plot.

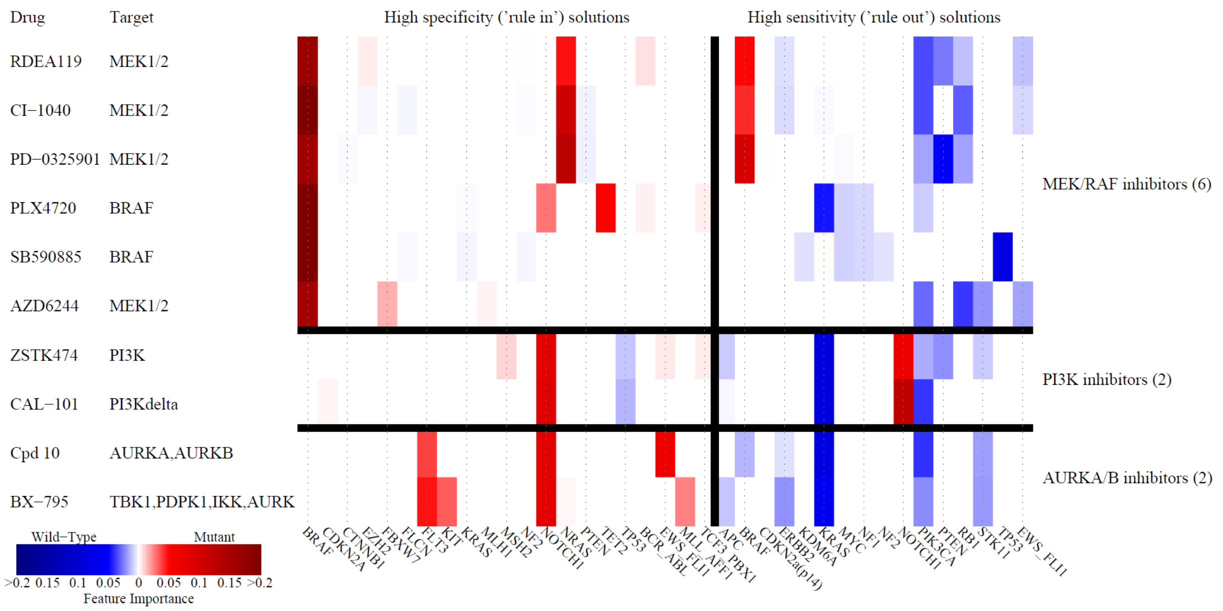

Supplementary Figure 10 | Feature importance scores for 'rule in' and 'rule out' solutions
FI scores of 6 MEK/RAF, 2 PI3K and 2 AURKA/B inhibitors (rows) for high specificity ('rule in') solutions (left) and high sensitivity ('rule out') solutions (right). High specificity solutions were defined as solutions with FPR<10%. Conversely, high sensitivity solutions were defined as solutions with TPR>90%. We distinguished between positive terms, indicating mutations (red) and negated terms, indicating wild-type (blue).

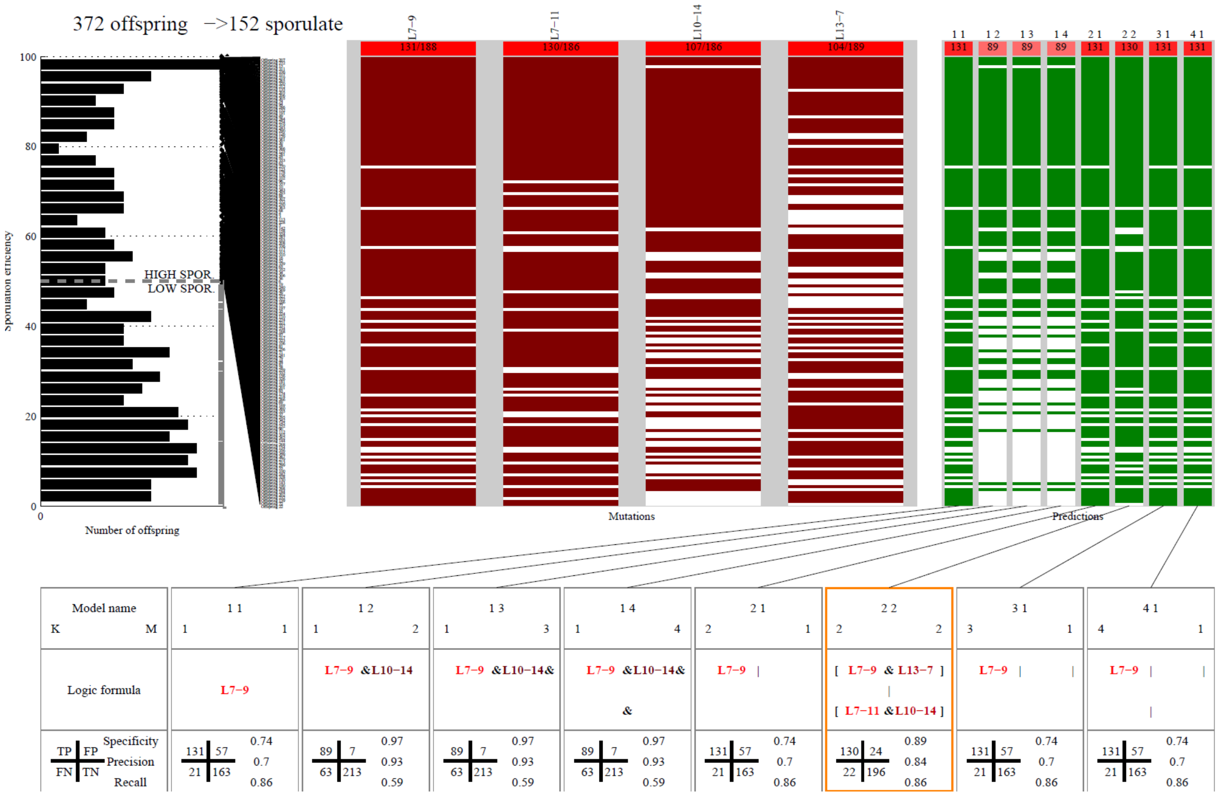

Supplementary Figure 11 | LOBICO results of the yeast cross phenotyped for sporulation efficiency

Standard LOBICO visualization of the uncovered logic models for the yeast cross dataset (**Supplementary Note 4**). See **Supplementary Data 1** for an explanation of the visualization.

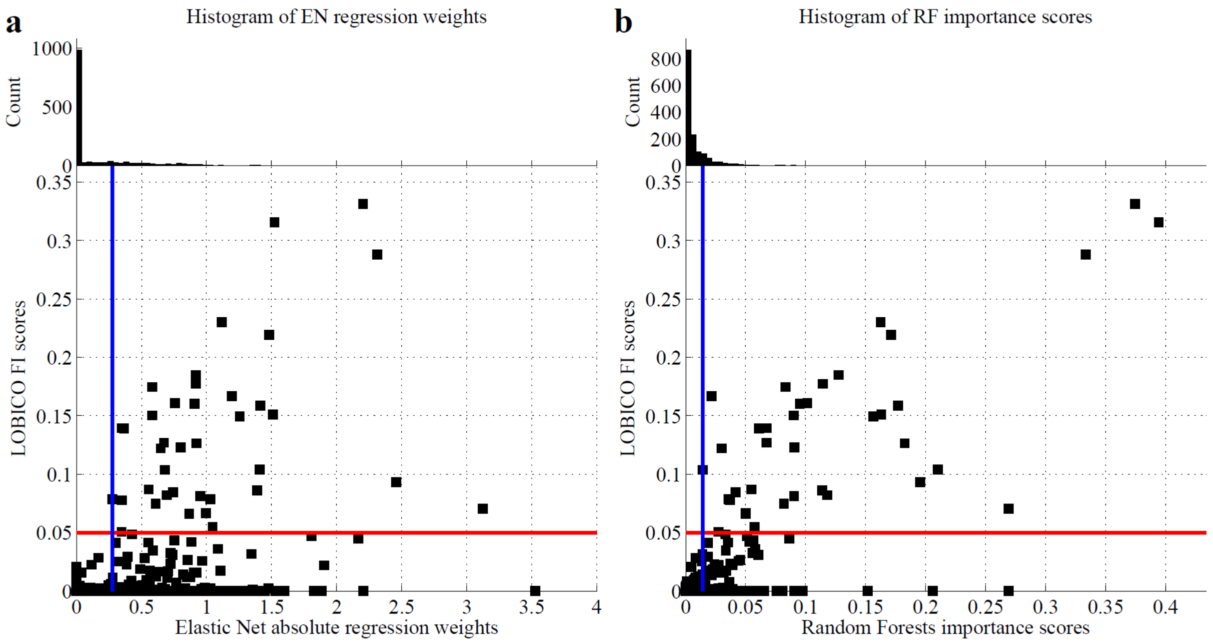

Supplementary Figure 12 | Comparison of feature importance scores between LOBICO, Elastic Net and Random Forests

a) Scatter plot comparing LOBICO's FI scores with EN's absolute regression weights. These scores and weights are derived from inferred models of the 25 drugs with the lowest CV error in the LOBICO analysis (**Supplementary Note 6**). The red line depicts the FI score of 0.05; features with a FI>0.05 are considered important predictors. The blue depicts the minimal EN regression weight for which the corresponding LOBICO FI was larger than 0.05. **b)** Similar to **a)**, except LOBICO's FI scores are compared to RF's importance scores.

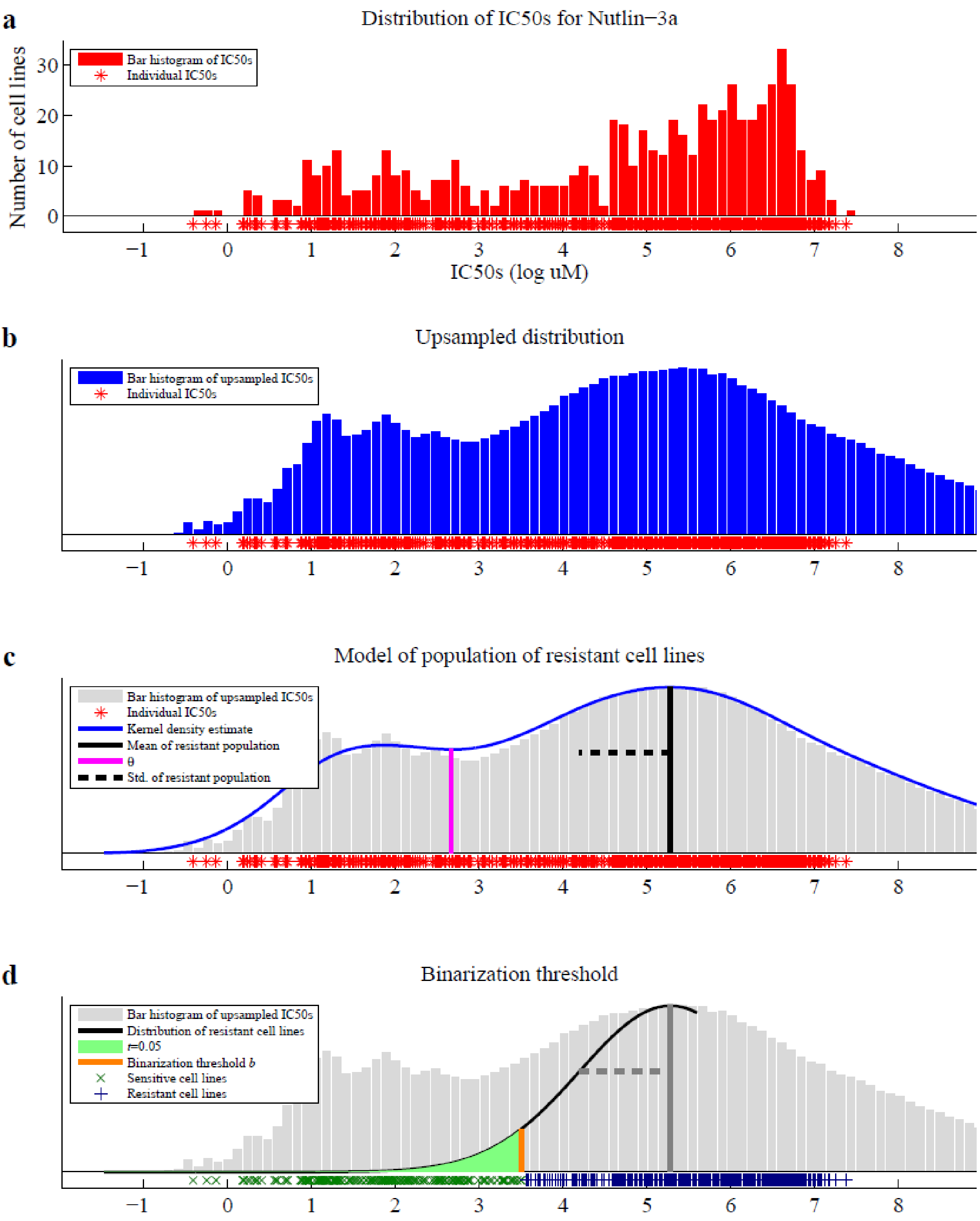

Supplementary Figure 13 | Four-step-procedure to binarize IC50s for Nutlin-3a

a) Histogram plot for the distribution of IC50s for the drug Nutlin−3a. **b)** Histogram plot for the upsampled distribution **c)** Visualization of an empirical model (obtained through density estimation) of the upsampled IC50s (depicted in blue). θ was computed using rule i. (See **Methods Section** for details.) **d)** Visualization of the model of resistant cell lines (depicted in black), from which the binarization threshold b (depicted in orange) is derived.

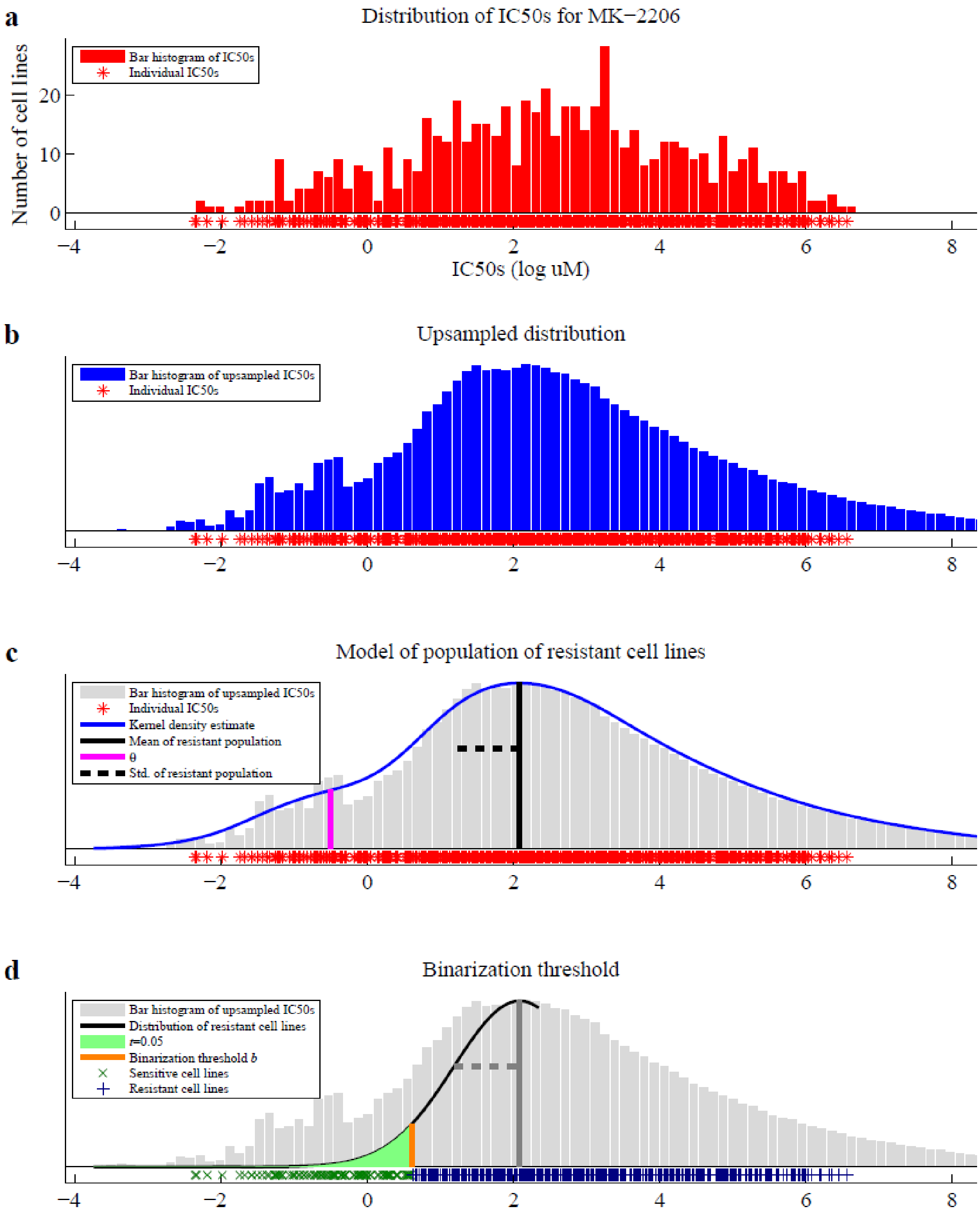

Supplementary Figure 14 | Four-step-procedure to binarize IC50s for MK−2206
Similar to **Supplementary Figure 13**, except showing the procedure for drug MK−2206, and the use of rule ii to find θ.

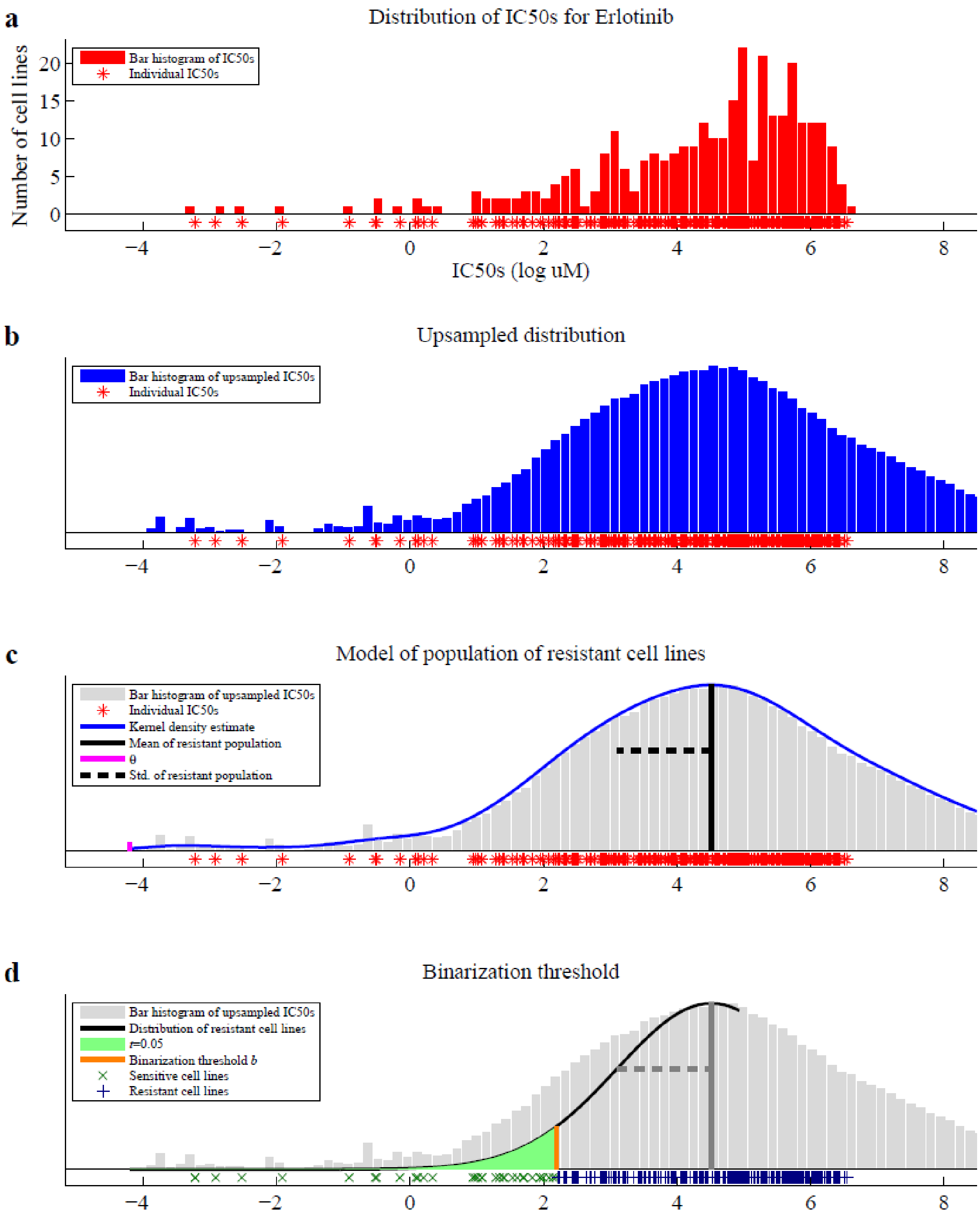

Supplementary Figure 15 | Four-step-procedure to binarize IC50s for Erlotinib
Similar to **Supplementary Figure 13**, except showing the procedure for drug Erlotinib, and the use of rule iii to find θ.

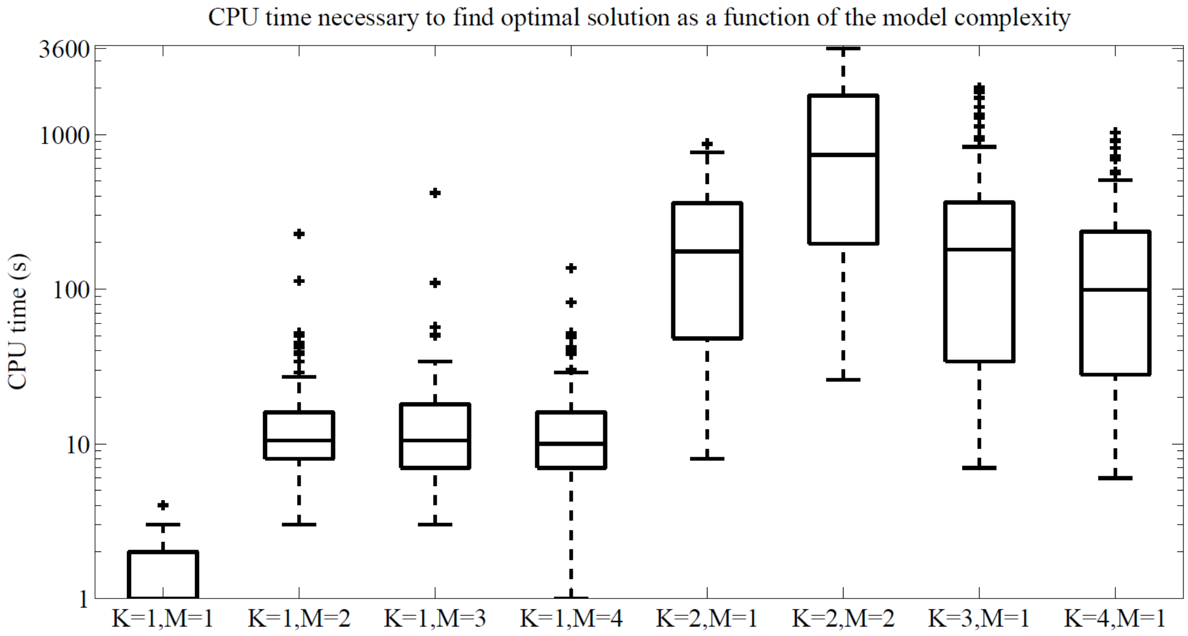

Supplementary Figure 16 | Time needed to find optimal solution

Boxplot of CPU time (y-axis) necessary to find the optimal solution as a function of the model complexity (x-axis). Each box is comprised of 142 values, i.e. the time necessary for CPLEX to find the optimal solution with the indicated model complexity for each of the 142 drugs. These experiments were performed on a computer cluster, where each ILP was run on one node (Intel(R) Xeon(R) CPU, E5645, 2.40GHz, 6 cores) at a time.

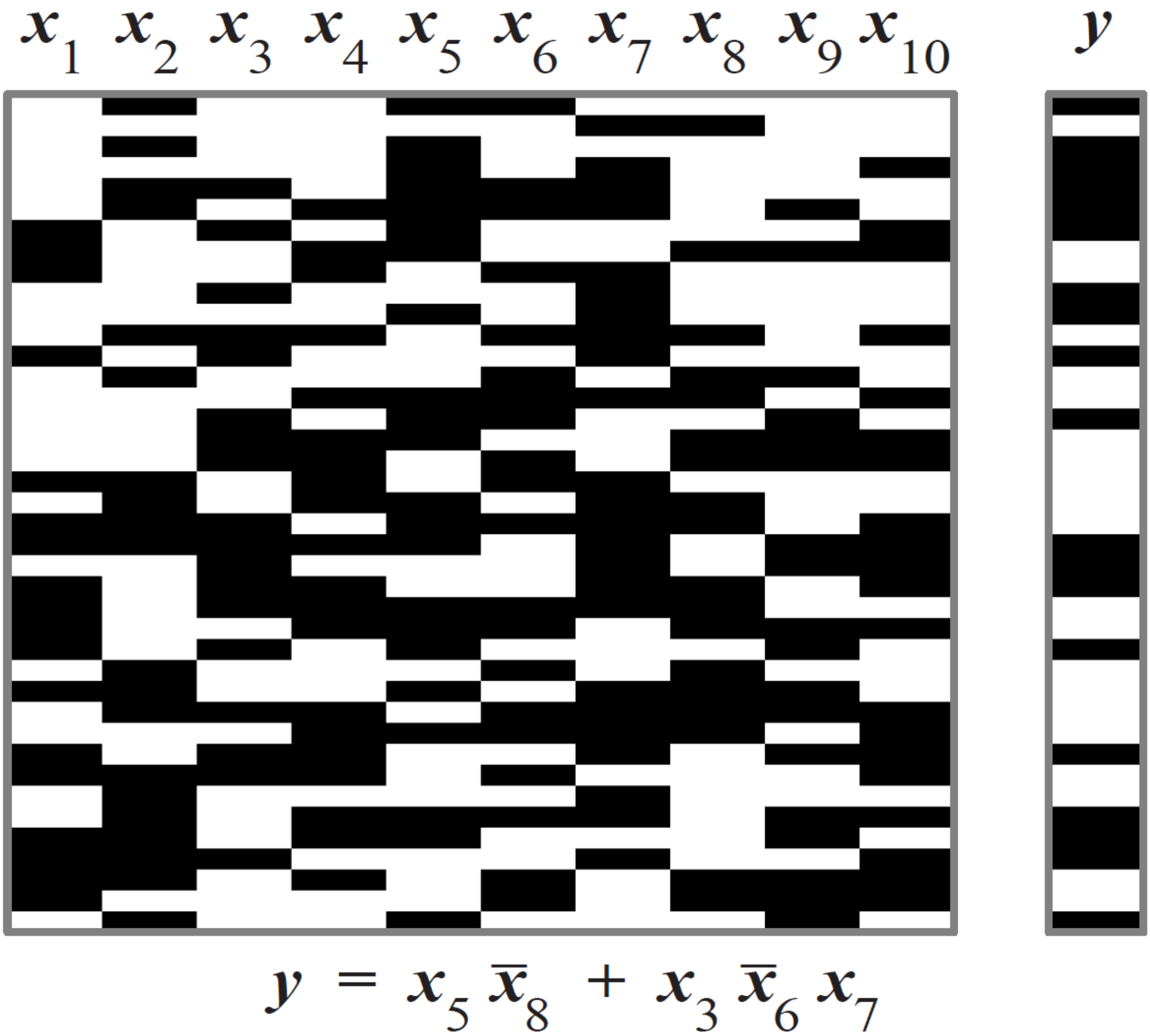

Supplementary Figure 17 | Example Boolean truth table

Boolean truth table (black=1, white=0) with 10 input variables $x_1, x_2, ..., x_{10}$ and output variable y. The DNF expression with $K = 2$ and $M = 3$ for the truth table depicted is below the table.

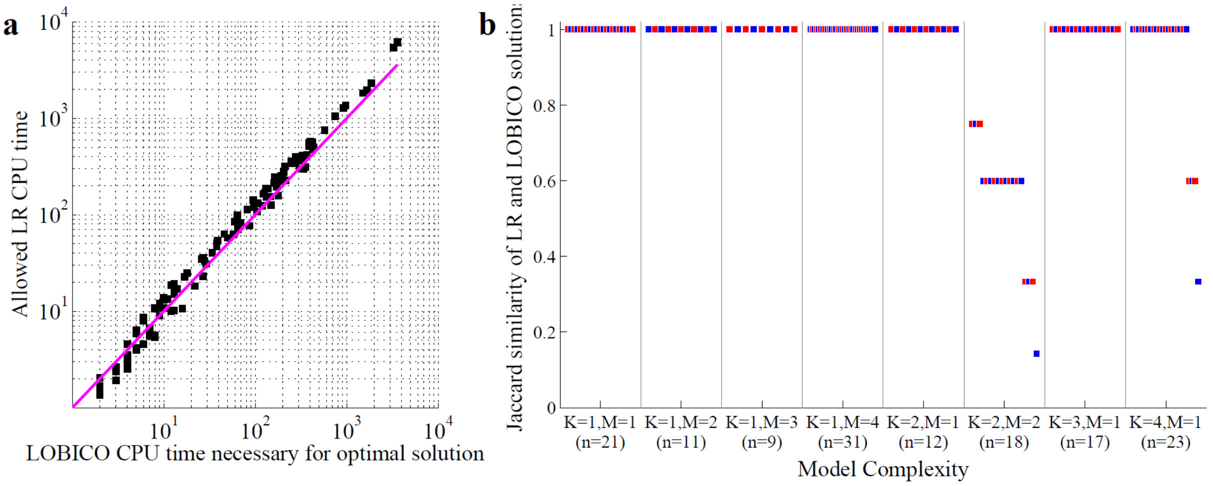

Supplementary Figure 18| Comparison of solution time and uncovered logic models between LOBICO and logic regression

a) Scatter plot comparing the CPU time needed for LOBICO to find the optimal solution (x-axis) and the CPU time given to LR to find the best solution (y-axis). Each of the 142 drugs is represented by a point. The magenta line is y=x. **b)** Plot of the Jaccard similarity between the LR and LOBICO solutions. Each of the 142 drugs is represented by a point. The points are alternately colored in blue and red for visibility. The drugs are grouped based on the model complexity (x-axis) for which the LOBICO and LR models were inferred.

Supplementary Data Explanation

Supplementary Data 1 | LOBICO visualization of the inferred logic models for all 142 drugs
PDF with a visualization of the LOBICO results for each drug (pages 8-149). The first 7 pages provide a visual explanation of the visualization.

Supplementary Data 2 | Drug information
Tab separated Excel table containing information about the 142 drugs. Specifically, (from left to right in the table), information about the drugs (ID, name and target), binarization thresholds, ground truth mapping to the gene mutation features, model performance statistics of the inferred logic models, and aggregated feature importance scores for the gene mutation features in the inferred logic models.

Supplementary Data 3 | 25 ROC models visual
PDF with visualizations of LOBICO solutions in the ROC space for each of 25 drugs with the lowest CV error in the original analysis. Blue crosses indicate the true positive rate (TPR) and false positive rate (FPR) at which the solution was found. The logic formula of the solutions is printed next to the blue crosses. The color of the genes in a formula indicate their FI. Colors range from black (moderately important) to bright red (highly important). For comparison, the best single predictor solutions are visualized in green. The inlay depicts the histogram of IC50s of the drug together with the binarization threshold.
The PDF consists of 50 pages; each of the 25 drugs is represented by two visualizations: one using the standard (binary) definitions sensitivity (or TPR) and specificity (or 1-FPR), and one using the weighted definitions of sensitivity and specificity (see Equations 12 and 13). These visualizations are similar to **Figure 4a**. The visualizations are generated automatically; text strings are partially overlapping in some cases.

Supplementary Data 4 | Cell line information
Tab separated Excel table containing information about the 714 cell lines. Specifically, (from left to right in the table), information about the cell lines (ID, name and description), the binary mutation status of the 60 gene mutation features, and the IC50s for each of the 142 drugs.

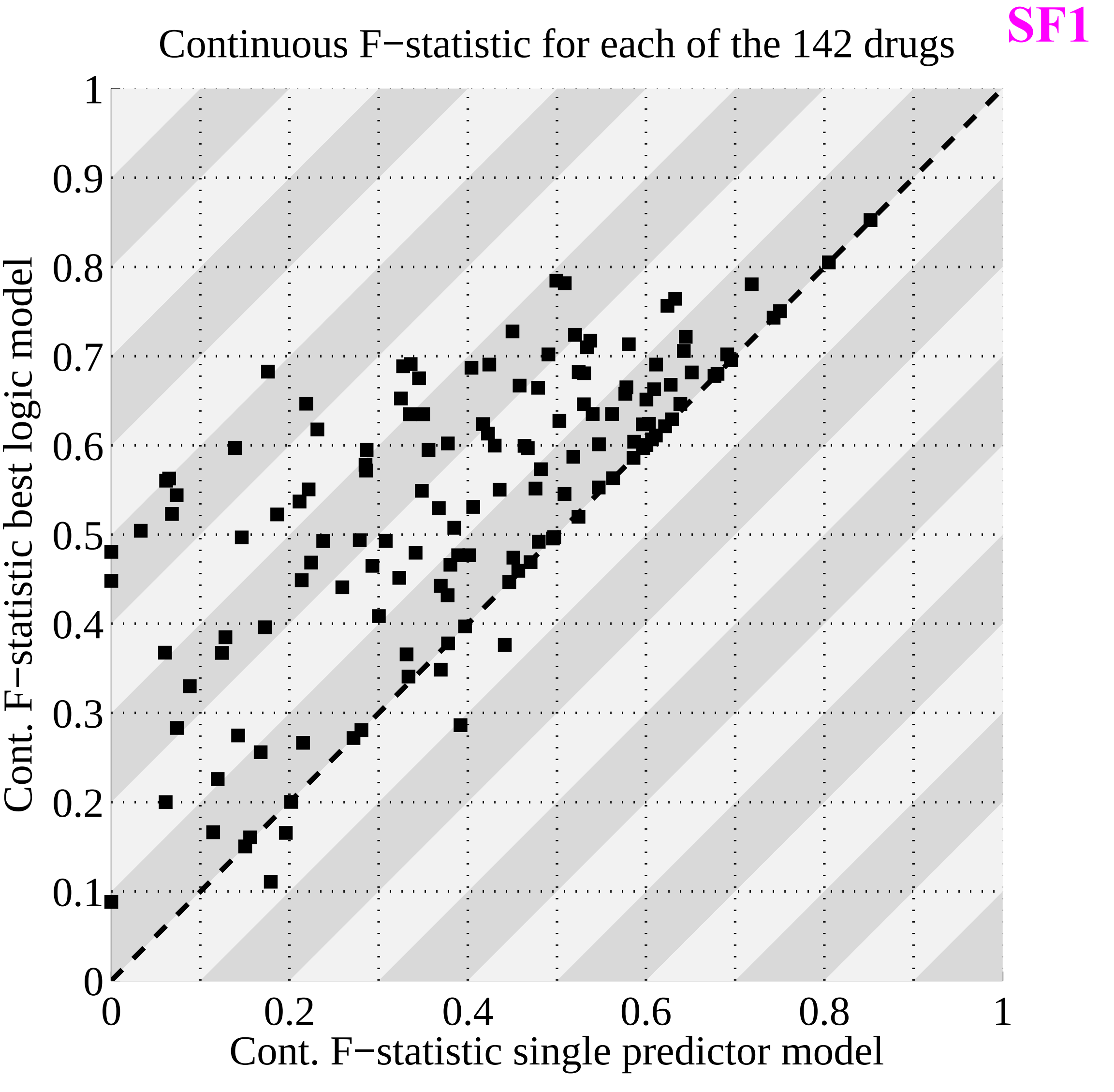
SF1
Continuous F−statistic for each of the 142 drugs
Cont. F−statistic best logic model
Cont. F−statistic single predictor model
0
0.1
0.2
0.3
0.4
0.5
0.6
0.7
0.8
0.9
1
0
0.2
0.4
0.6
0.8
1

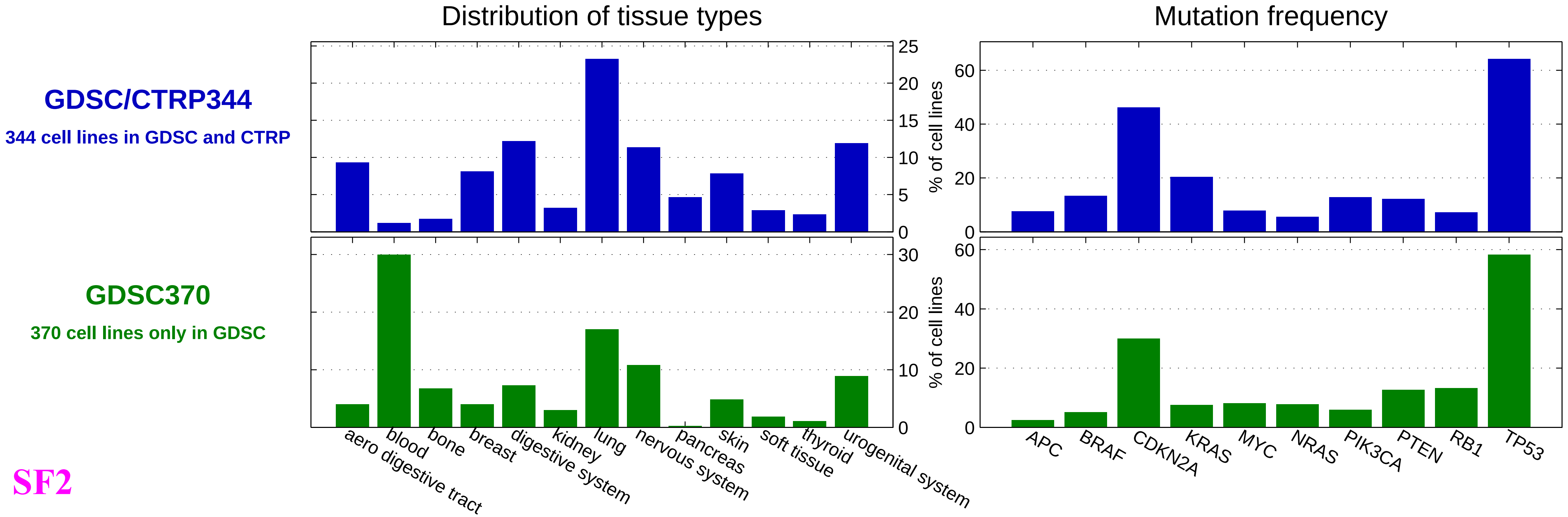

Distribution of tissue types
Mutation frequency
GDSC/CTRP344
344 cell lines in GDSC and CTRP
GDSC370
370 cell lines only in GDSC
% of cell lines
% of cell lines
0
5
10
15
20
25
0
10
20
30
0
20
40
60
0
20
40
60
aero digestive tract
blood
bone
breast
digestive system
kidney
lung
nervous system
pancreas
skin
soft tissue
thyroid
urogenital system
APC
BRAF
CDKN2A
KRAS
MYC
NRAS
PIK3CA
PTEN
RB1
TP53

SF2

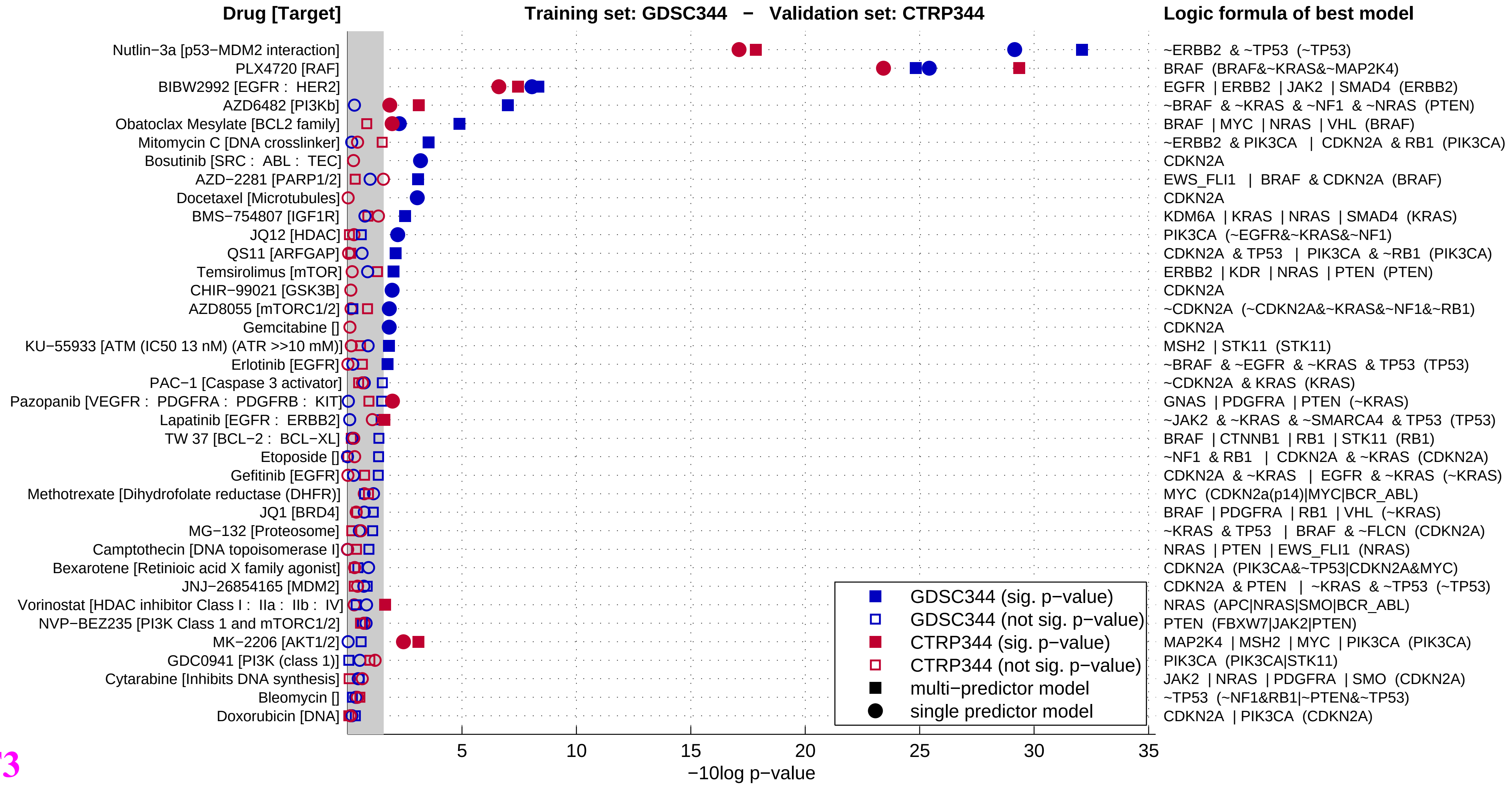
Drug [Target]
Training set: GDSC344 – Validation set: CTRP344
Logic formula of best model
Nutlin−3a [p53−MDM2 interaction]
PLX4720 [RAF]
BIBW2992 [EGFR : HER2]
AZD6482 [PI3Kb]
Obatoclax Mesylate [BCL2 family]
Mitomycin C [DNA crosslinker]
Bosutinib [SRC : ABL : TEC]
AZD−2281 [PARP1/2]
Docetaxel [Microtubules]
BMS−754807 [IGF1R]
JQ12 [HDAC]
QS11 [ARFGAP]
Temsirolimus [mTOR]
CHIR−99021 [GSK3B]
AZD8055 [mTORC1/2]
Gemcitabine []
KU−55933 [ATM (IC50 13 nM) (ATR >>10 mM)]
Erlotinib [EGFR]
PAC−1 [Caspase 3 activator]
Pazopanib [VEGFR : PDGFRA : PDGFRB : KIT]
Lapatinib [EGFR : ERBB2]
TW 37 [BCL−2 : BCL−XL]
Etoposide []
Gefitinib [EGFR]
Methotrexate [Dihydrofolate reductase (DHFR)]
JQ1 [BRD4]
MG−132 [Proteosome]
Camptothecin [DNA topoisomerase I]
Bexarotene [Retinioic acid X family agonist]
JNJ−26854165 [MDM2]
Vorinostat [HDAC inhibitor Class I : IIa : IIb : IV]
NVP−BEZ235 [PI3K Class 1 and mTORC1/2]
MK−2206 [AKT1/2]
GDC0941 [PI3K (class 1)]
Cytarabine [Inhibits DNA synthesis]
Bleomycin []
Doxorubicin [DNA]
~ERBB2 & ~TP53 (~TP53)
BRAF (BRAF&~KRAS&~MAP2K4)
EGFR | ERBB2 | JAK2 | SMAD4 (ERBB2)
~BRAF & ~KRAS & ~NF1 & ~NRAS (PTEN)
BRAF | MYC | NRAS | VHL (BRAF)
~ERBB2 & PIK3CA | CDKN2A & RB1 (PIK3CA)
CDKN2A
EWS_FLI1 | BRAF & CDKN2A (BRAF)
CDKN2A
KDM6A | KRAS | NRAS | SMAD4 (KRAS)
PIK3CA (~EGFR&~KRAS&~NF1)
CDKN2A & TP53 | PIK3CA & ~RB1 (PIK3CA)
ERBB2 | KDR | NRAS | PTEN (PTEN)
CDKN2A
~CDKN2A (~CDKN2A&~KRAS&~NF1&~RB1)
CDKN2A
MSH2 | STK11 (STK11)
~BRAF & ~EGFR & ~KRAS & TP53 (TP53)
~CDKN2A & KRAS (KRAS)
GNAS | PDGFRA | PTEN (~KRAS)
~JAK2 & ~KRAS & ~SMARCA4 & TP53 (TP53)
BRAF | CTNNB1 | RB1 | STK11 (RB1)
~NF1 & RB1 | CDKN2A & ~KRAS (CDKN2A)
CDKN2A & ~KRAS | EGFR & ~KRAS (~KRAS)
MYC (CDKN2a(p14)|MYC|BCR_ABL)
BRAF | PDGFRA | RB1 | VHL (~KRAS)
~KRAS & TP53 | BRAF & ~FLCN (CDKN2A)
NRAS | PTEN | EWS_FLI1 (NRAS)
CDKN2A (PIK3CA&~TP53|CDKN2A&MYC)
CDKN2A & PTEN | ~KRAS & ~TP53 (~TP53)
NRAS (APC|NRAS|SMO|BCR_ABL)
PTEN (FBXW7|JAK2|PTEN)
MAP2K4 | MSH2 | MYC | PIK3CA (PIK3CA)
PIK3CA (PIK3CA|STK11)
JAK2 | NRAS | PDGFRA | SMO (CDKN2A)
~TP53 (~NF1&RB1|~PTEN&~TP53)
CDKN2A | PIK3CA (CDKN2A)
GDSC344 (sig. p−value)
GDSC344 (not sig. p−value)
CTRP344 (sig. p−value)
CTRP344 (not sig. p−value)
multi−predictor model
single predictor model
5
10
15
20
25
30
35
−10log p−value

SF3

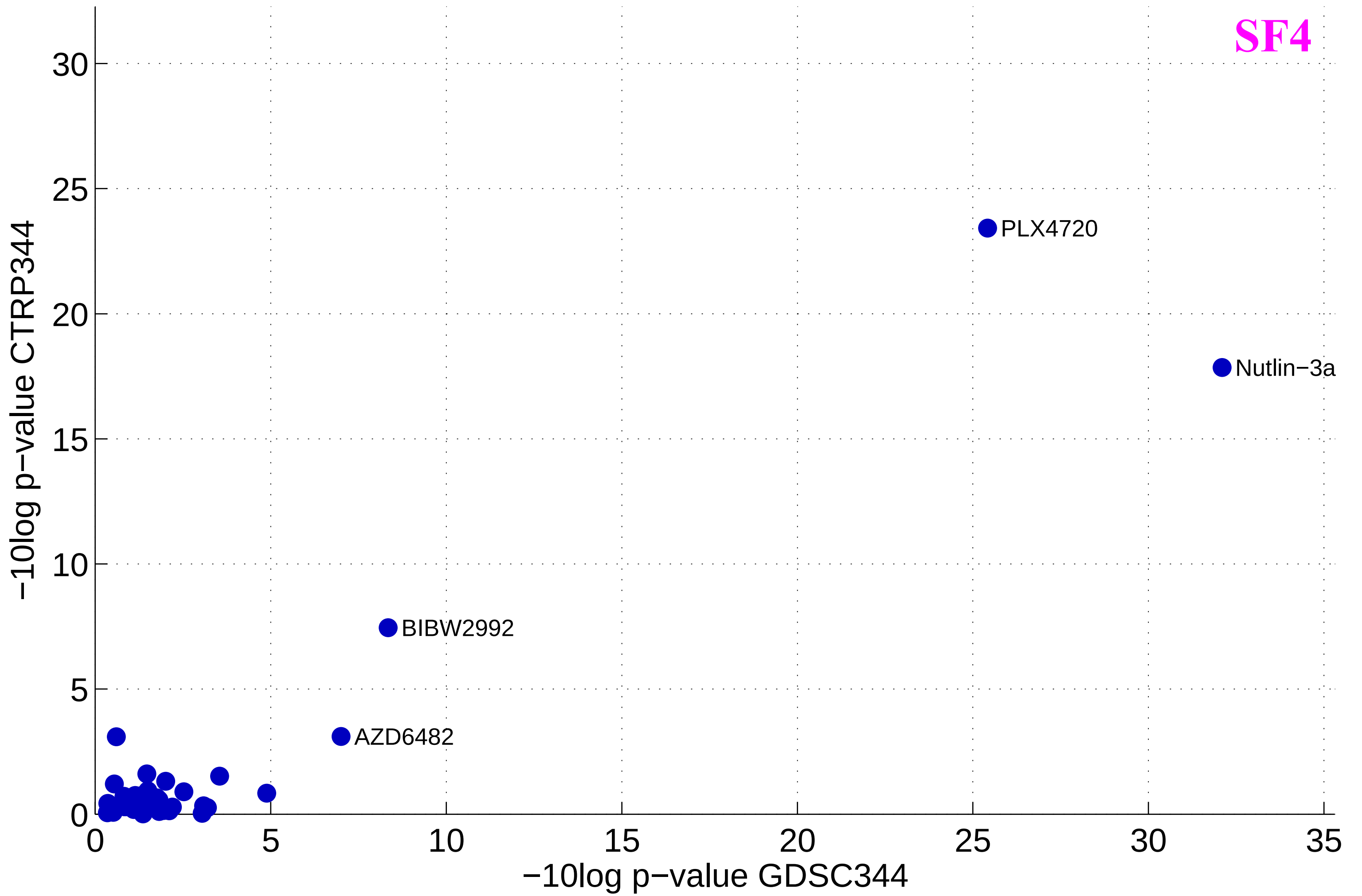
SF4
PLX4720
Nutlin−3a
BIBW2992
AZD6482
−10log p−value CTRP344
−10log p−value GDSC344
0
5
10
15
20
25
30
35

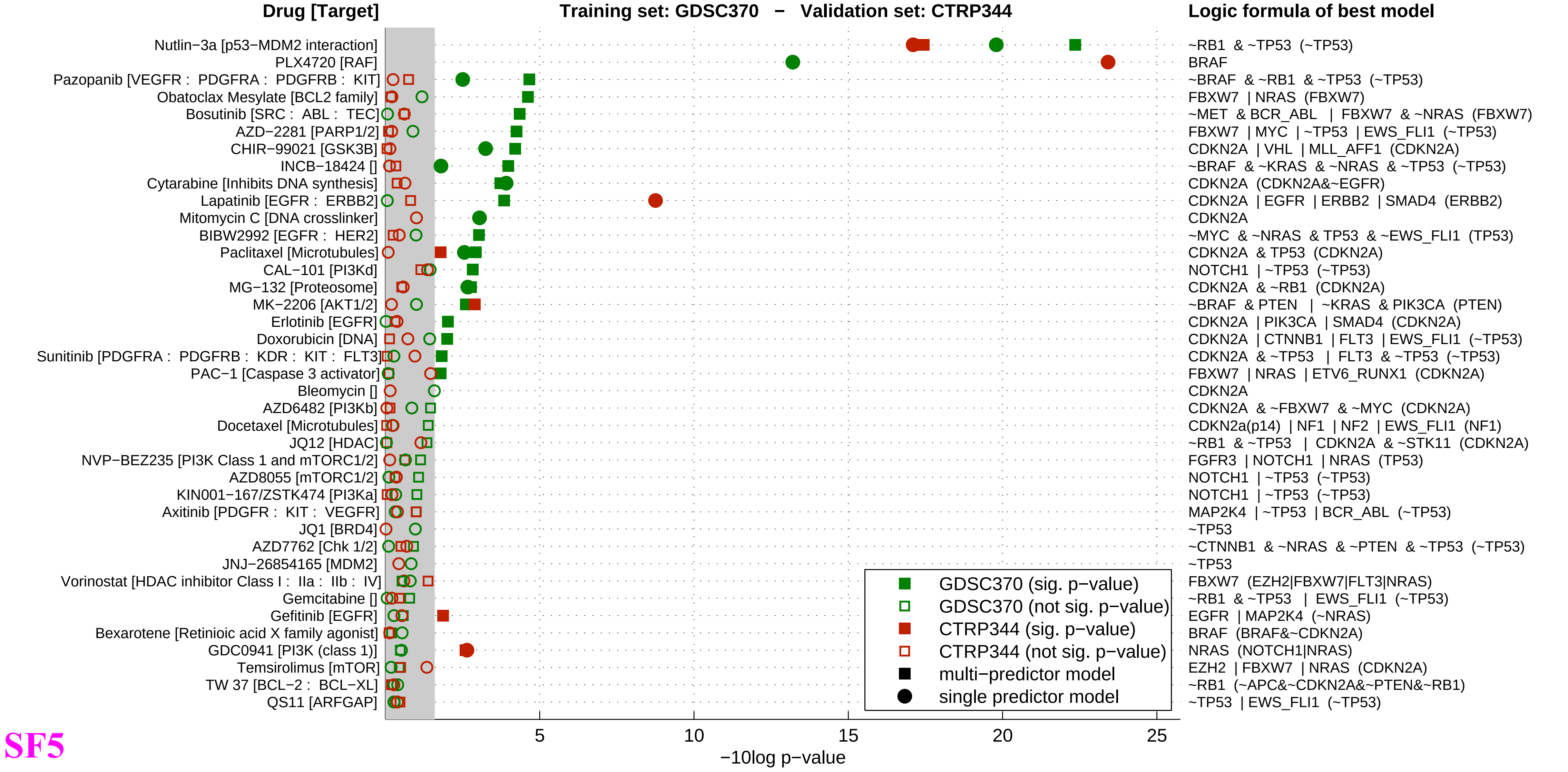
Drug [Target]
Training set: GDSC370 – Validation set: CTRP344
Logic formula of best model
Nutlin−3a [p53−MDM2 interaction]
PLX4720 [RAF]
Pazopanib [VEGFR : PDGFRA : PDGFRB : KIT]
Obatoclax Mesylate [BCL2 family]
Bosutinib [SRC : ABL : TEC]
AZD−2281 [PARP1/2]
CHIR−99021 [GSK3B]
INCB−18424 []
Cytarabine [Inhibits DNA synthesis]
Lapatinib [EGFR : ERBB2]
Mitomycin C [DNA crosslinker]
BIBW2992 [EGFR : HER2]
Paclitaxel [Microtubules]
CAL−101 [PI3Kd]
MG−132 [Proteosome]
MK−2206 [AKT1/2]
Erlotinib [EGFR]
Doxorubicin [DNA]
Sunitinib [PDGFRA : PDGFRB : KDR : KIT : FLT3]
PAC−1 [Caspase 3 activator]
Bleomycin []
AZD6482 [PI3Kb]
Docetaxel [Microtubules]
JQ12 [HDAC]
NVP−BEZ235 [PI3K Class 1 and mTORC1/2]
AZD8055 [mTORC1/2]
KIN001−167/ZSTK474 [PI3Ka]
Axitinib [PDGFR : KIT : VEGFR]
JQ1 [BRD4]
AZD7762 [Chk 1/2]
JNJ−26854165 [MDM2]
Vorinostat [HDAC inhibitor Class I : IIa : IIb : IV]
Gemcitabine []
Gefitinib [EGFR]
Bexarotene [Retinioic acid X family agonist]
GDC0941 [PI3K (class 1)]
Temsirolimus [mTOR]
TW 37 [BCL−2 : BCL−XL]
QS11 [ARFGAP]
~RB1 & ~TP53 (~TP53)
BRAF
~BRAF & ~RB1 & ~TP53 (~TP53)
FBXW7 | NRAS (FBXW7)
~MET & BCR_ABL | FBXW7 & ~NRAS (FBXW7)
FBXW7 | MYC | ~TP53 | EWS_FLI1 (~TP53)
CDKN2A | VHL | MLL_AFF1 (CDKN2A)
~BRAF & ~KRAS & ~NRAS & ~TP53 (~TP53)
CDKN2A (CDKN2A&~EGFR)
CDKN2A | EGFR | ERBB2 | SMAD4 (ERBB2)
CDKN2A
~MYC & ~NRAS & TP53 & ~EWS_FLI1 (TP53)
CDKN2A & TP53 (CDKN2A)
NOTCH1 | ~TP53 (~TP53)
CDKN2A & ~RB1 (CDKN2A)
~BRAF & PTEN | ~KRAS & PIK3CA (PTEN)
CDKN2A | PIK3CA | SMAD4 (CDKN2A)
CDKN2A | CTNNB1 | FLT3 | EWS_FLI1 (~TP53)
CDKN2A & ~TP53 | FLT3 & ~TP53 (~TP53)
FBXW7 | NRAS | ETV6_RUNX1 (CDKN2A)
CDKN2A
CDKN2A & ~FBXW7 & ~MYC (CDKN2A)
CDKN2a(p14) | NF1 | NF2 | EWS_FLI1 (NF1)
~RB1 & ~TP53 | CDKN2A & ~STK11 (CDKN2A)
FGFR3 | NOTCH1 | NRAS (TP53)
NOTCH1 | ~TP53 (~TP53)
NOTCH1 | ~TP53 (~TP53)
MAP2K4 | ~TP53 | BCR_ABL (~TP53)
~TP53
~CTNNB1 & ~NRAS & ~PTEN & ~TP53 (~TP53)
~TP53
FBXW7 (EZH2|FBXW7|FLT3|NRAS)
~RB1 & ~TP53 | EWS_FLI1 (~TP53)
EGFR | MAP2K4 (~NRAS)
BRAF (BRAF&~CDKN2A)
NRAS (NOTCH1|NRAS)
EZH2 | FBXW7 | NRAS (CDKN2A)
~RB1 (~APC&~CDKN2A&~PTEN&~RB1)
~TP53 | EWS_FLI1 (~TP53)
GDSC370 (sig. p−value)
GDSC370 (not sig. p−value)
CTRP344 (sig. p−value)
CTRP344 (not sig. p−value)
multi−predictor model
single predictor model
5
10
15
20
25
−10log p−value
SF5

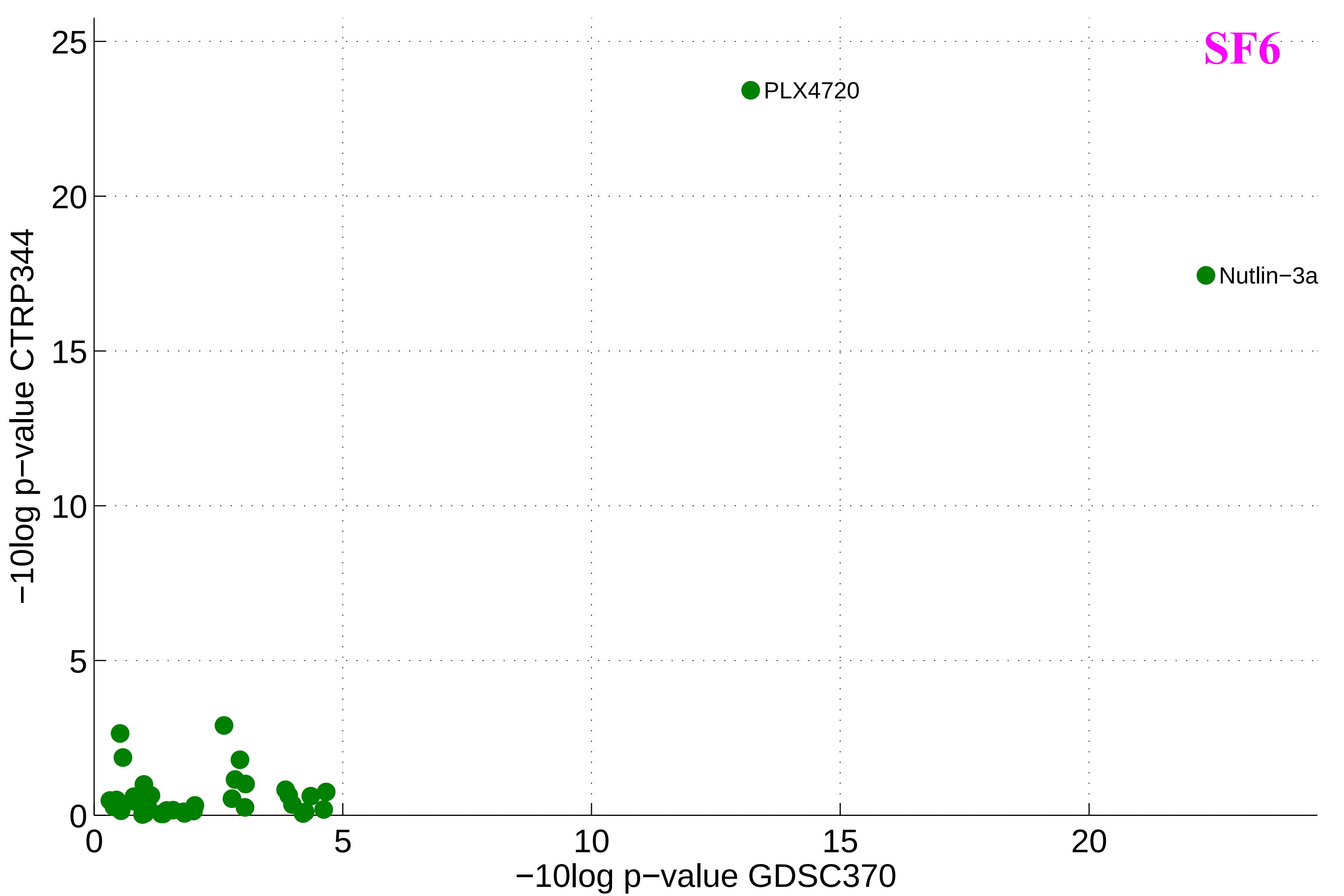

SF6
PLX4720
Nutlin−3a
25
20
15
10
5
0
0
5
10
15
20
−10log p−value CTRP344
−10log p−value GDSC370

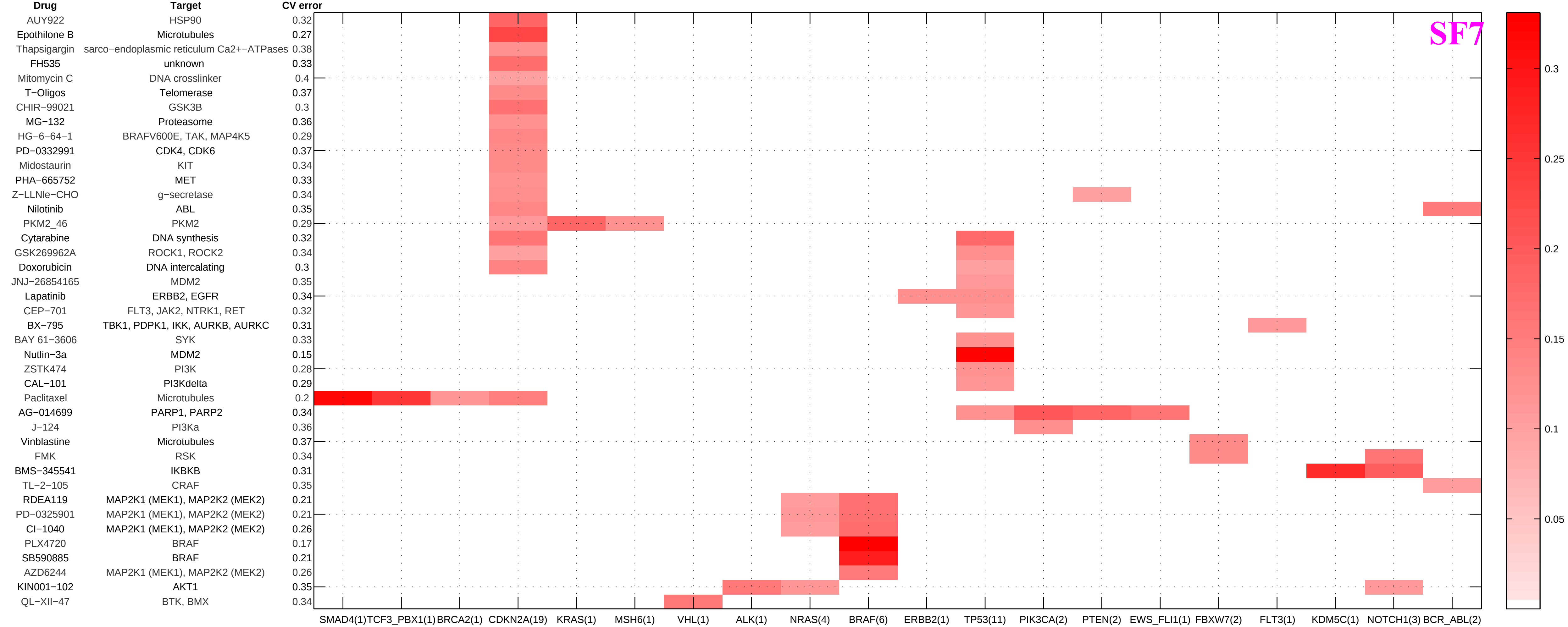

Drug
Target
CV error
AUY922 HSP90 0.32
Epothilone B Microtubules 0.27
Thapsigargin sarco−endoplasmic reticulum Ca2+−ATPases 0.38
FH535 unknown 0.33
Mitomycin C DNA crosslinker 0.4
T−Oligos Telomerase 0.37
CHIR−99021 GSK3B 0.3
MG−132 Proteasome 0.36
HG−6−64−1 BRAFV600E, TAK, MAP4K5 0.29
PD−0332991 CDK4, CDK6 0.37
Midostaurin KIT 0.34
PHA−665752 MET 0.33
Z−LLNle−CHO g−secretase 0.34
Nilotinib ABL 0.35
PKM2_46 PKM2 0.29
Cytarabine DNA synthesis 0.32
GSK269962A ROCK1, ROCK2 0.34
Doxorubicin DNA intercalating 0.3
JNJ−26854165 MDM2 0.35
Lapatinib ERBB2, EGFR 0.34
CEP−701 FLT3, JAK2, NTRK1, RET 0.32
BX−795 TBK1, PDPK1, IKK, AURKB, AURKC 0.31
BAY 61−3606 SYK 0.33
Nutlin−3a MDM2 0.15
ZSTK474 PI3K 0.28
CAL−101 PI3Kdelta 0.29
Paclitaxel Microtubules 0.2
AG−014699 PARP1, PARP2 0.34
J−124 PI3Ka 0.36
Vinblastine Microtubules 0.37
FMK RSK 0.34
BMS−345541 IKBKB 0.31
TL−2−105 CRAF 0.35
RDEA119 MAP2K1 (MEK1), MAP2K2 (MEK2) 0.21
PD−0325901 MAP2K1 (MEK1), MAP2K2 (MEK2) 0.21
CI−1040 MAP2K1 (MEK1), MAP2K2 (MEK2) 0.26
PLX4720 BRAF 0.17
SB590885 BRAF 0.21
AZD6244 MAP2K1 (MEK1), MAP2K2 (MEK2) 0.26
KIN001−102 AKT1 0.35
QL−XII−47 BTK, BMX 0.34
SMAD4(1) TCF3_PBX1(1) BRCA2(1) CDKN2A(19) KRAS(1) MSH6(1) VHL(1) ALK(1) NRAS(4) BRAF(6) ERBB2(1) TP53(11) PIK3CA(2) PTEN(2) EWS_FLI1(1) FBXW7(2) FLT3(1) KDM5C(1) NOTCH1(3) BCR_ABL(2)
SF7
0.3
0.25
0.2
0.15
0.1
0.05

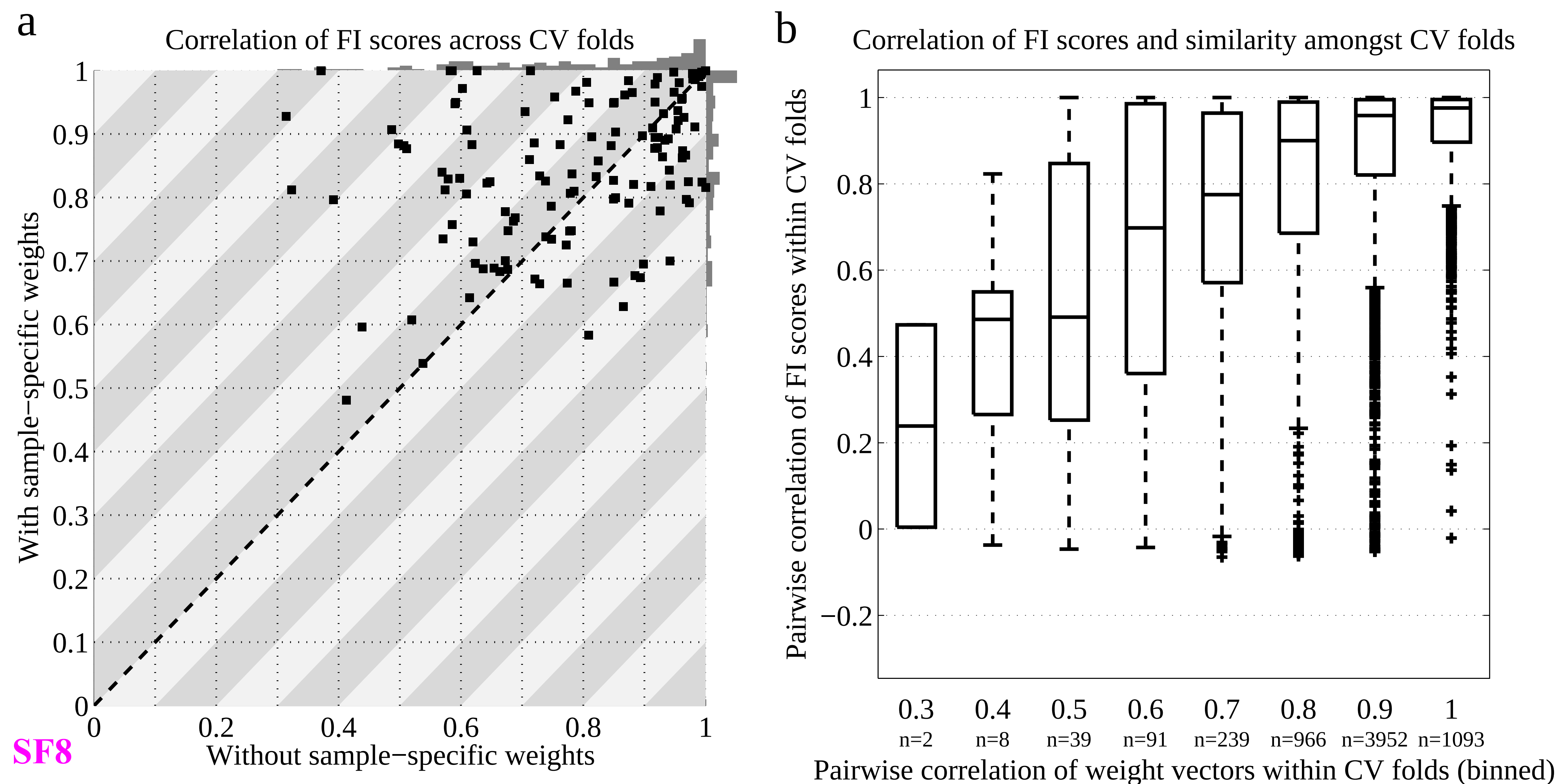
a
Correlation of FI scores across CV folds
With sample−specific weights
Without sample−specific weights
0
0.1
0.2
0.3
0.4
0.5
0.6
0.7
0.8
0.9
1
0
0.2
0.4
0.6
0.8
1
b
Correlation of FI scores and similarity amongst CV folds
Pairwise correlation of FI scores within CV folds
−0.2
0
0.2
0.4
0.6
0.8
1
0.3
n=2
0.4
n=8
0.5
n=39
0.6
n=91
0.7
n=239
0.8
n=966
0.9
n=3952
1
n=1093
Pairwise correlation of weight vectors within CV folds (binned)
SF8

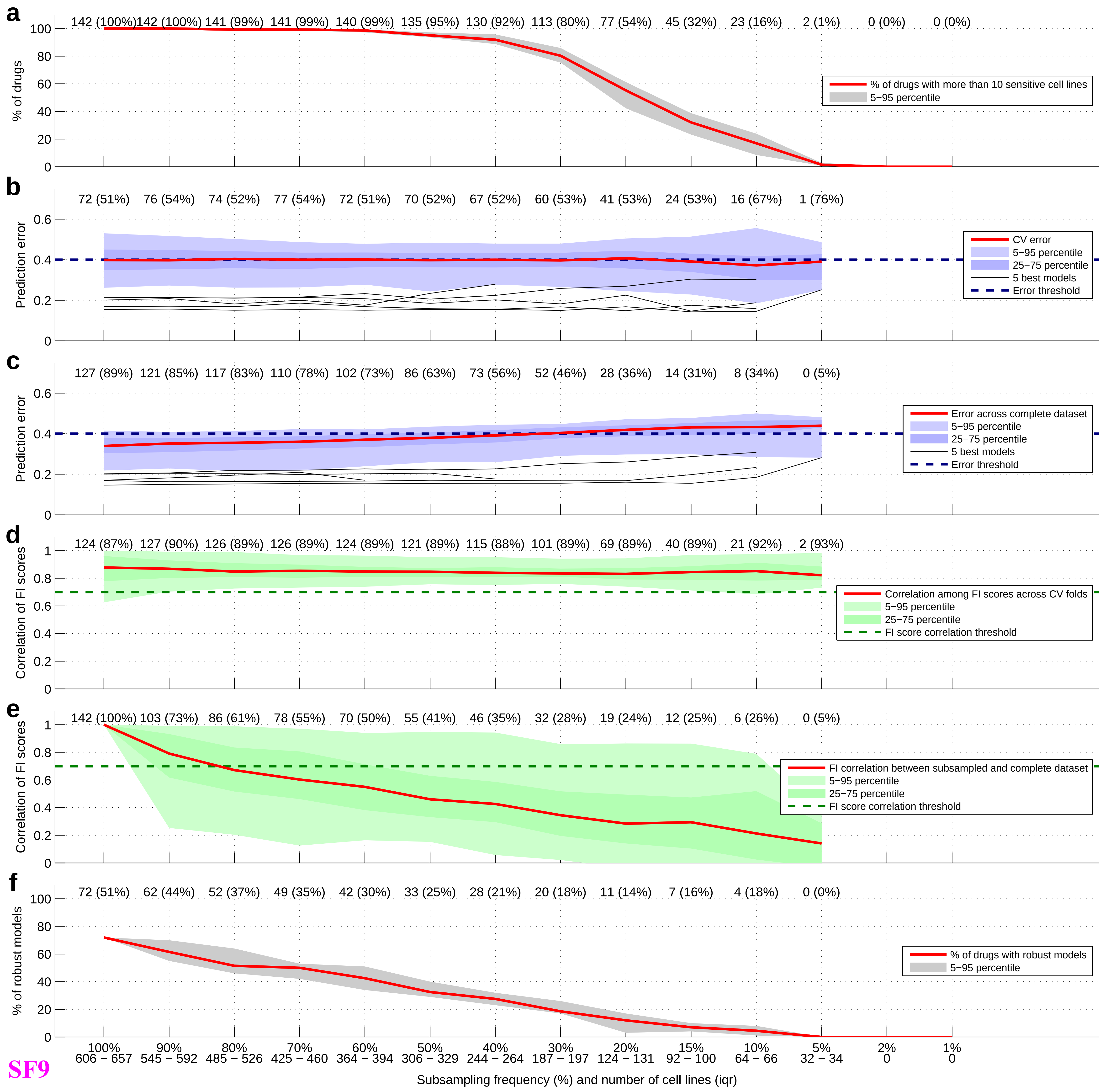

a
% of drugs
142 (100%) 142 (100%) 141 (99%) 141 (99%) 140 (99%) 135 (95%) 130 (92%) 113 (80%) 77 (54%) 45 (32%) 23 (16%) 2 (1%) 0 (0%) 0 (0%)
% of drugs with more than 10 sensitive cell lines
5−95 percentile
b
Prediction error
72 (51%) 76 (54%) 74 (52%) 77 (54%) 72 (51%) 70 (52%) 67 (52%) 60 (53%) 41 (53%) 24 (53%) 16 (67%) 1 (76%)
CV error
5−95 percentile
25−75 percentile
5 best models
Error threshold
c
Prediction error
127 (89%) 121 (85%) 117 (83%) 110 (78%) 102 (73%) 86 (63%) 73 (56%) 52 (46%) 28 (36%) 14 (31%) 8 (34%) 0 (5%)
Error across complete dataset
5−95 percentile
25−75 percentile
5 best models
Error threshold
d
Correlation of FI scores
124 (87%) 127 (90%) 126 (89%) 126 (89%) 124 (89%) 121 (89%) 115 (88%) 101 (89%) 69 (89%) 40 (89%) 21 (92%) 2 (93%)
Correlation among FI scores across CV folds
5−95 percentile
25−75 percentile
FI score correlation threshold
e
Correlation of FI scores
142 (100%) 103 (73%) 86 (61%) 78 (55%) 70 (50%) 55 (41%) 46 (35%) 32 (28%) 19 (24%) 12 (25%) 6 (26%) 0 (5%)
FI correlation between subsampled and complete dataset
5−95 percentile
25−75 percentile
FI score correlation threshold
f
% of robust models
72 (51%) 62 (44%) 52 (37%) 49 (35%) 42 (30%) 33 (25%) 28 (21%) 20 (18%) 11 (14%) 7 (16%) 4 (18%) 0 (0%)
% of drugs with robust models
5−95 percentile
100% 606 − 657
90% 545 − 592
80% 485 − 526
70% 425 − 460
60% 364 − 394
50% 306 − 329
40% 244 − 264
30% 187 − 197
20% 124 − 131
15% 92 − 100
10% 64 − 66
5% 32 − 34
2% 0
1% 0
Subsampling frequency (%) and number of cell lines (iqr)

SF9

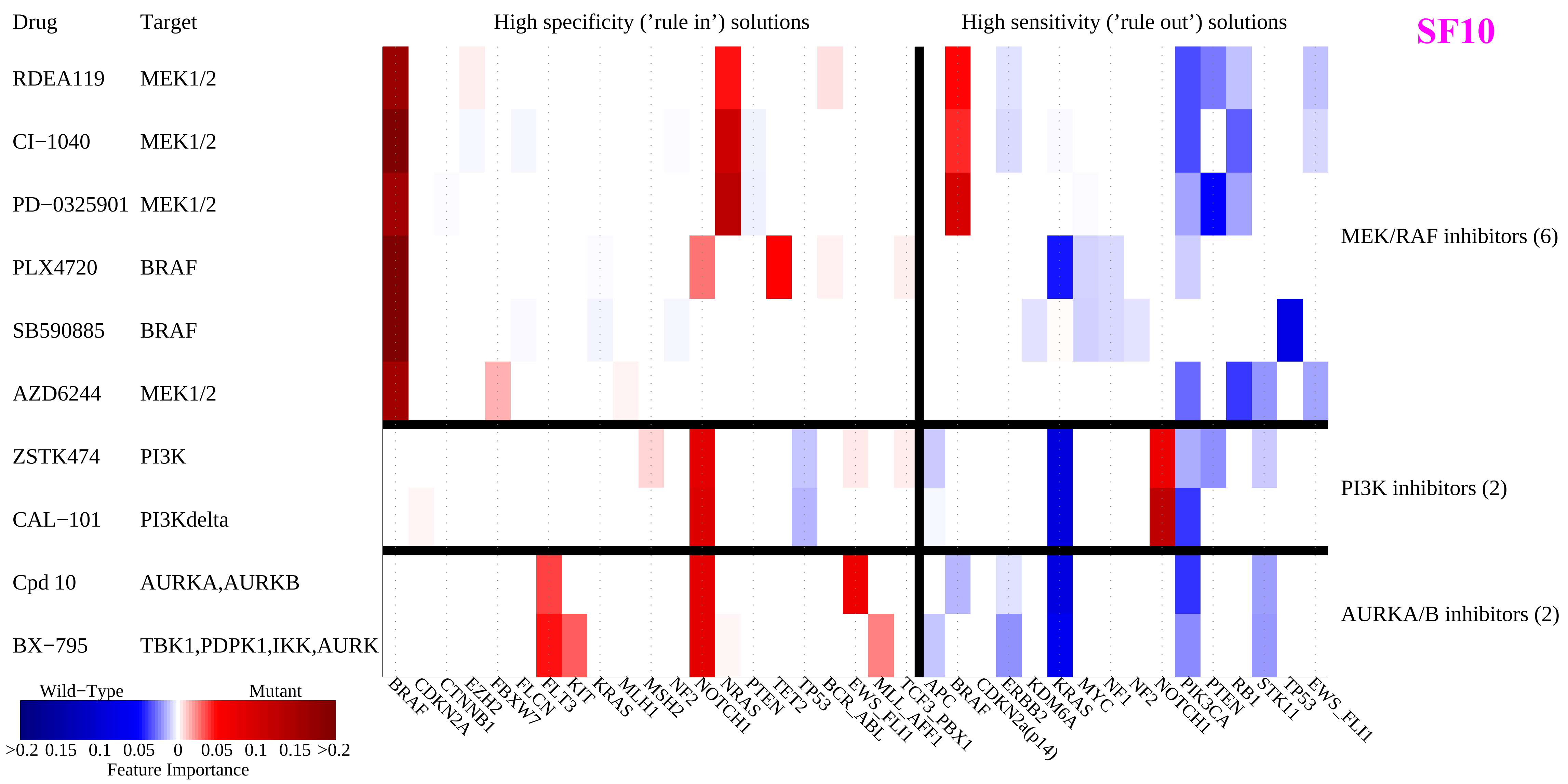
SF10
Drug
Target
High specificity ('rule in') solutions
High sensitivity ('rule out') solutions
RDEA119 MEK1/2
CI−1040 MEK1/2
PD−0325901 MEK1/2
PLX4720 BRAF
SB590885 BRAF
AZD6244 MEK1/2
ZSTK474 PI3K
CAL−101 PI3Kdelta
Cpd 10 AURKA,AURKB
BX−795 TBK1,PDPK1,IKK,AURK
MEK/RAF inhibitors (6)
PI3K inhibitors (2)
AURKA/B inhibitors (2)
BRAF CDKN2A CTNNB1 EZH2 FBXW7 FLCN FLT3 KIT KRAS MLH1 MSH2 NF2 NOTCH1 NRAS PTEN TET2 TP53 BCR_ABL EWS_FLI1 MLL_AFF1 TCF3_PBX1
APC BRAF CDKN2a(p14) ERBB2 KDM6A KRAS MYC NF1 NF2 NOTCH1 PIK3CA PTEN RB1 STK11 TP53 EWS_FLI1
Wild−Type
Mutant
>0.2 0.15 0.1 0.05 0 0.05 0.1 0.15 >0.2
Feature Importance

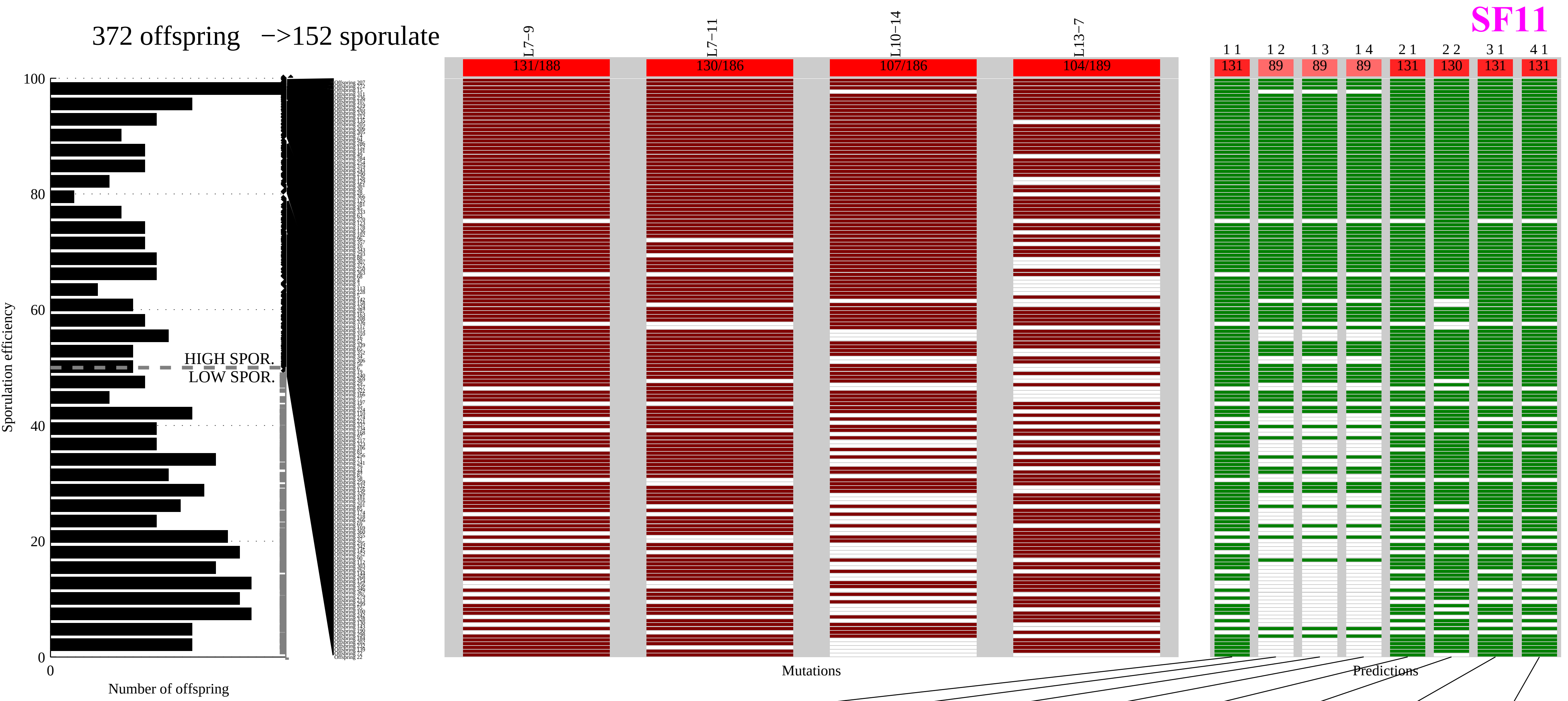

Model name (K, M)	1 1	1 2	1 3	1 4	2 1	2 2	3 1	4 1
K / M	1 / 1	1 / 2	1 / 3	1 / 4	2 / 1	2 / 2	3 / 1	4 / 1
Logic formula	**L7−9**	**L7−9 &L10−14**	**L7−9 &L10−14&**	**L7−9 &L10−14&** **&**	**L7−9 |**	**[L7−9 & L13−7] | [L7−11 &L10−14]**	**L7−9 | |**	**L7−9 | | |**
TP FP / FN TN	131 57 / 21 163	89 7 / 63 213	89 7 / 63 213	89 7 / 63 213	131 57 / 21 163	130 24 / 22 196	131 57 / 21 163	131 57 / 21 163
Specificity	0.74	0.97	0.97	0.97	0.74	0.89	0.74	0.74
Precision	0.7	0.93	0.93	0.93	0.7	0.84	0.7	0.7
Recall	0.86	0.59	0.59	0.59	0.86	0.86	0.86	0.86

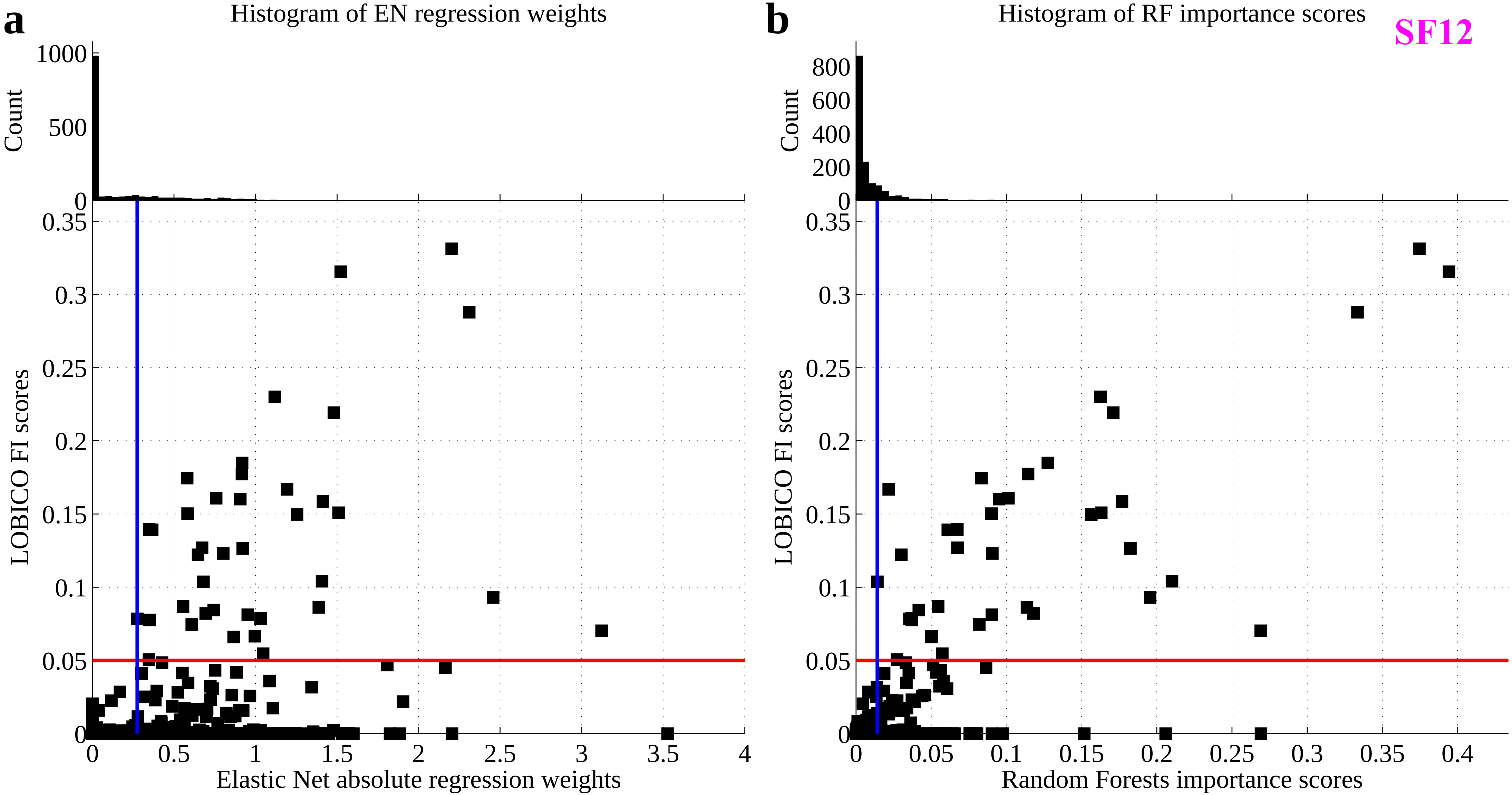

a
Histogram of EN regression weights
Count
1000
500
0
LOBICO FI scores
0.35
0.3
0.25
0.2
0.15
0.1
0.05
0
0.5
1
1.5
2
2.5
3
3.5
4
Elastic Net absolute regression weights
b
Histogram of RF importance scores
SF12
800
600
400
200
0.05
0.1
0.15
0.2
0.25
0.3
0.35
0.4
Random Forests importance scores

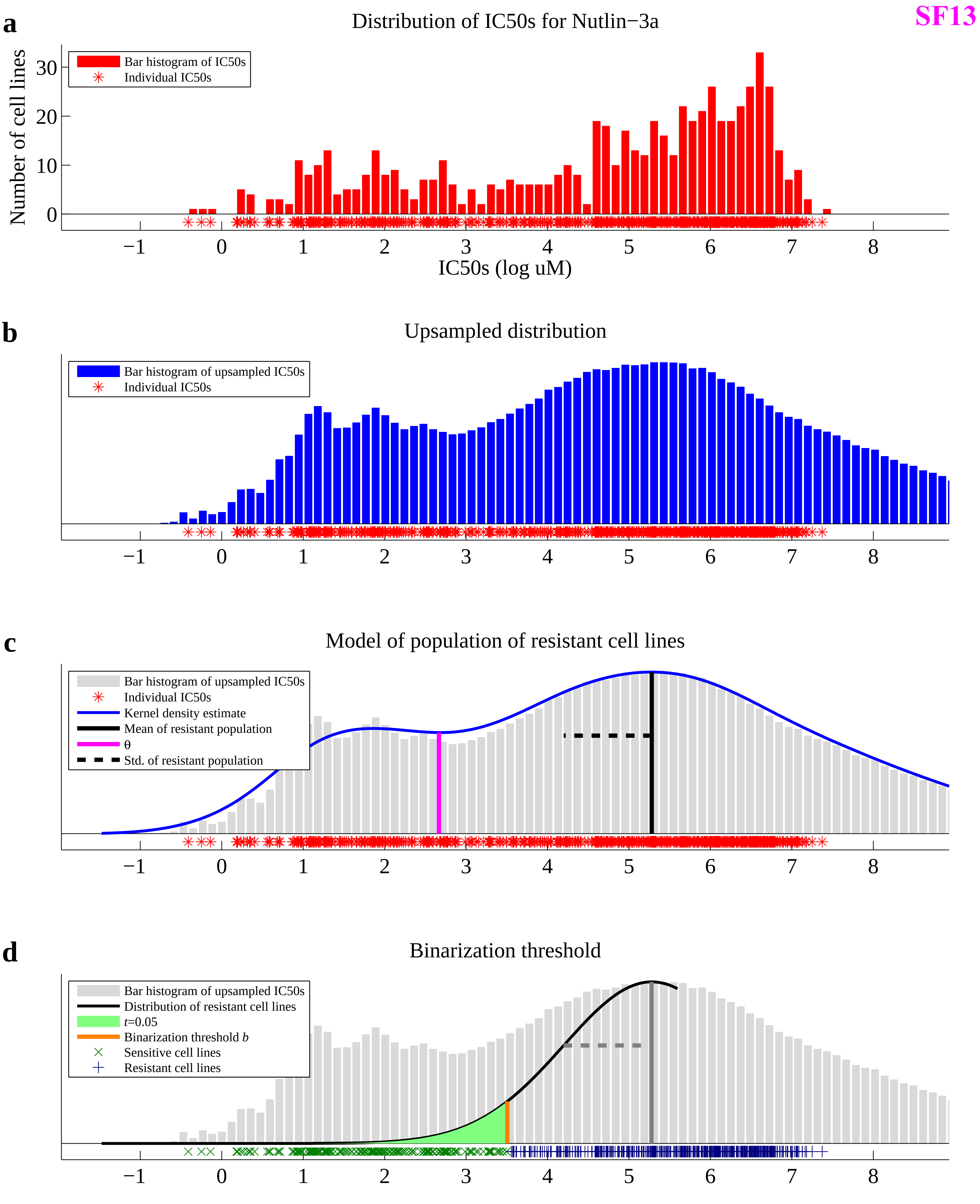
SF13
a
Distribution of IC50s for Nutlin−3a
Bar histogram of IC50s
Individual IC50s
Number of cell lines
30
20
10
0
−1
0
1
2
3
4
5
6
7
8
IC50s (log uM)
b
Upsampled distribution
Bar histogram of upsampled IC50s
Individual IC50s
c
Model of population of resistant cell lines
Bar histogram of upsampled IC50s
Individual IC50s
Kernel density estimate
Mean of resistant population
θ
Std. of resistant population
d
Binarization threshold
Bar histogram of upsampled IC50s
Distribution of resistant cell lines
t=0.05
Binarization threshold b
Sensitive cell lines
Resistant cell lines

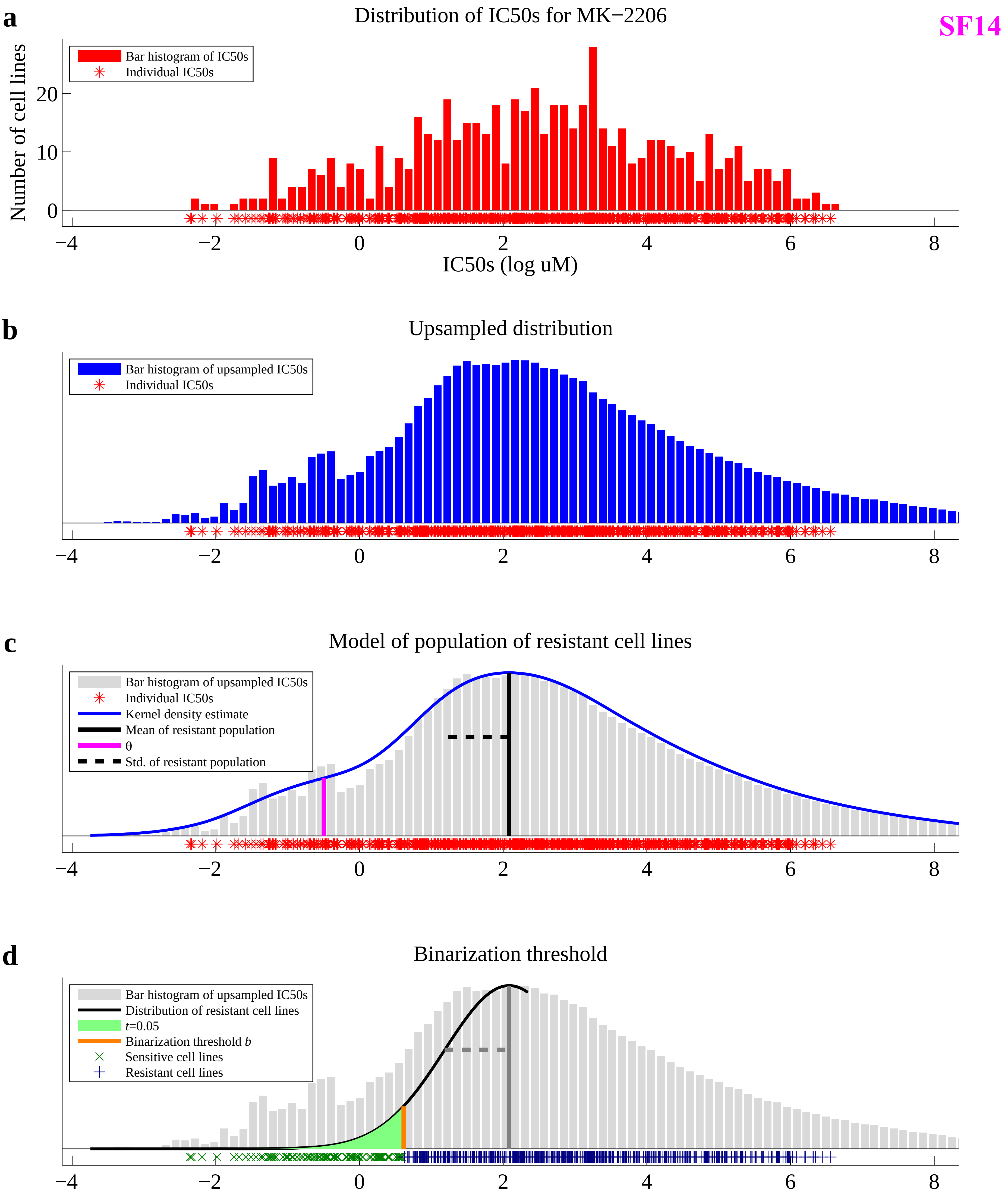

a
Distribution of IC50s for MK−2206
SF14
Number of cell lines
Bar histogram of IC50s
Individual IC50s
IC50s (log uM)
b
Upsampled distribution
Bar histogram of upsampled IC50s
Individual IC50s
c
Model of population of resistant cell lines
Bar histogram of upsampled IC50s
Individual IC50s
Kernel density estimate
Mean of resistant population
θ
Std. of resistant population
d
Binarization threshold
Bar histogram of upsampled IC50s
Distribution of resistant cell lines
t=0.05
Binarization threshold b
Sensitive cell lines
Resistant cell lines

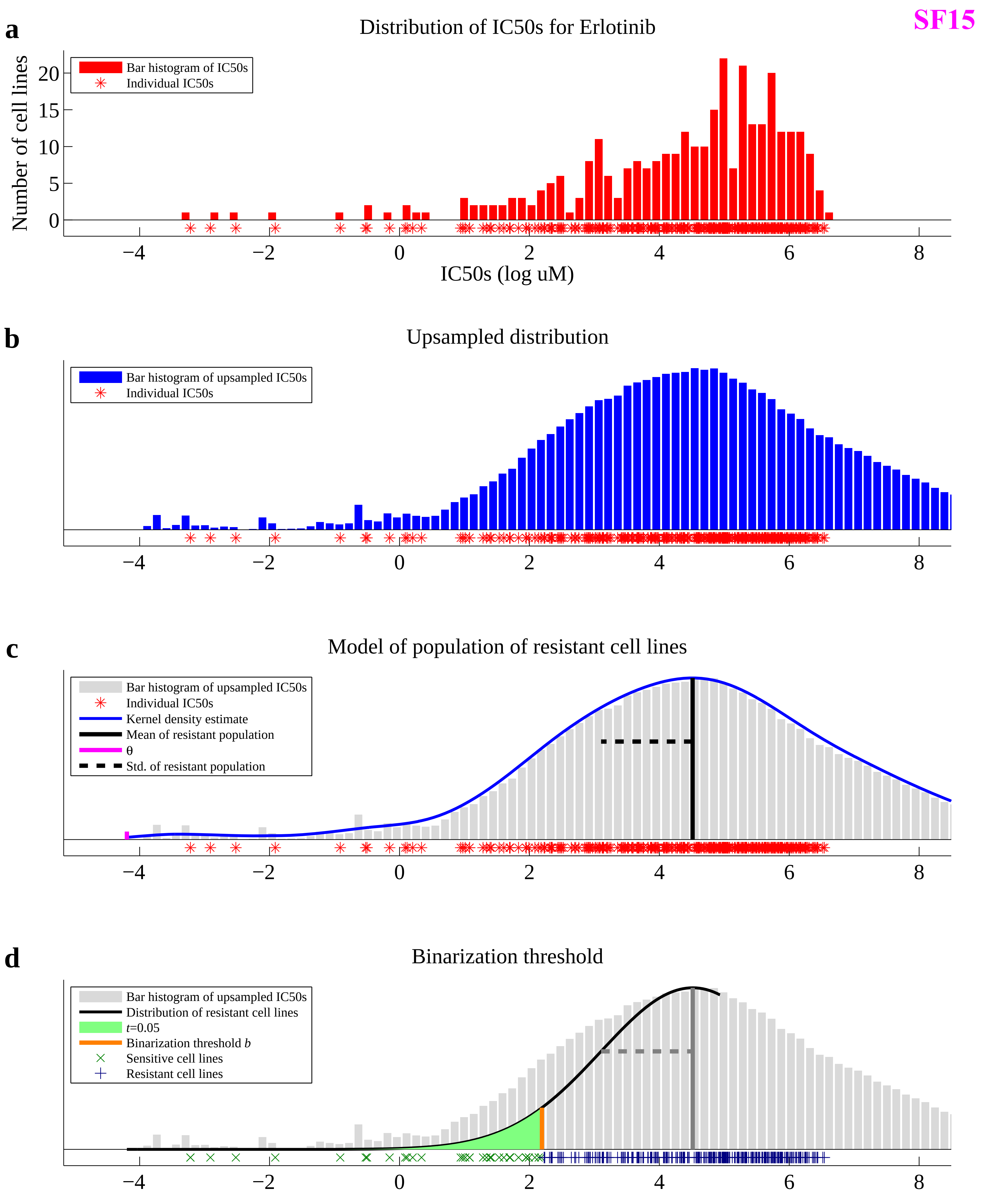
SF15
a
Distribution of IC50s for Erlotinib
Bar histogram of IC50s
Individual IC50s
Number of cell lines
IC50s (log uM)
b
Upsampled distribution
Bar histogram of upsampled IC50s
Individual IC50s
c
Model of population of resistant cell lines
Bar histogram of upsampled IC50s
Individual IC50s
Kernel density estimate
Mean of resistant population
θ
Std. of resistant population
d
Binarization threshold
Bar histogram of upsampled IC50s
Distribution of resistant cell lines
t=0.05
Binarization threshold b
Sensitive cell lines
Resistant cell lines

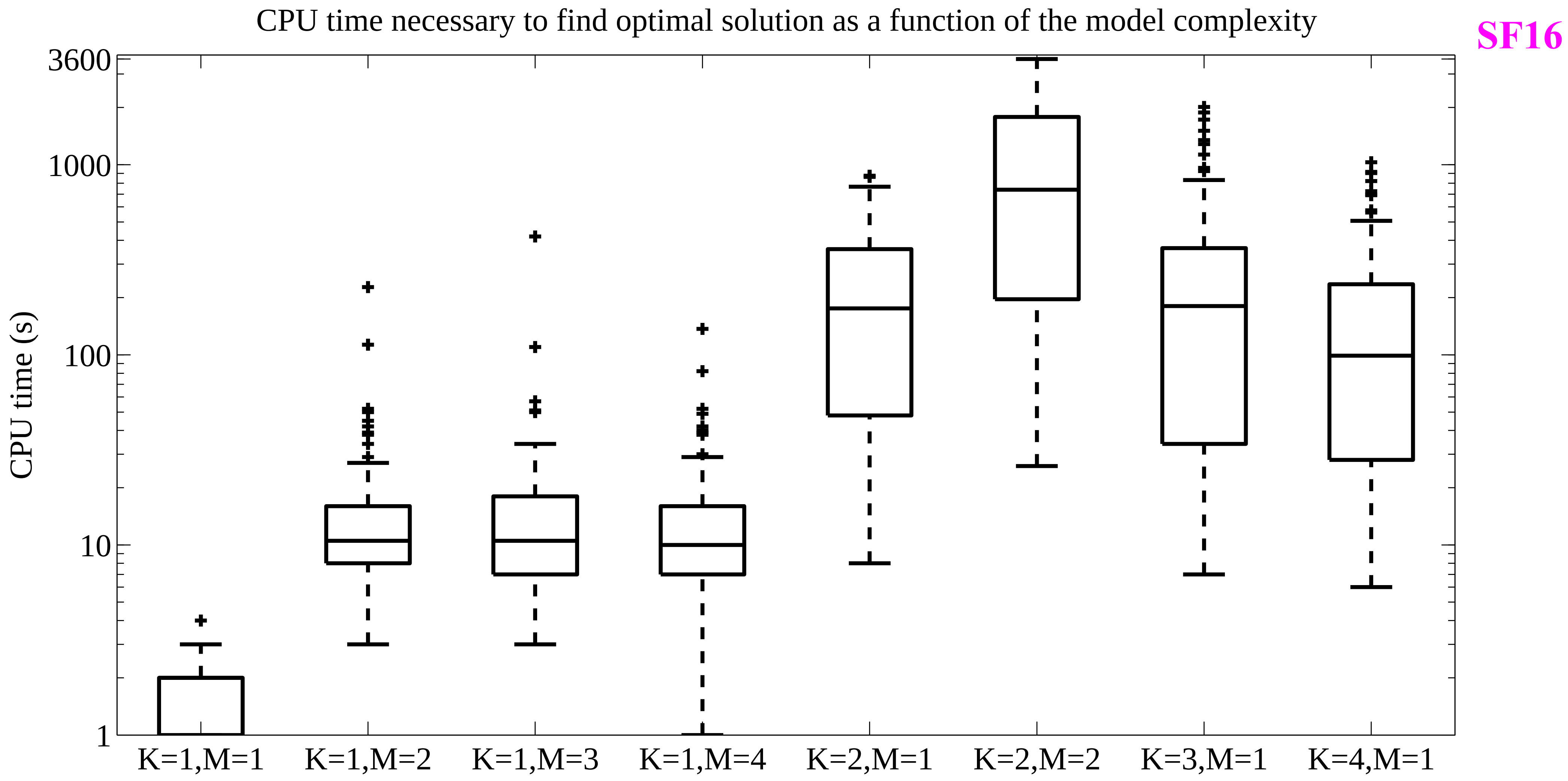
CPU time necessary to find optimal solution as a function of the model complexity
SF16
CPU time (s)
3600
1000
100
10
1
K=1,M=1
K=1,M=2
K=1,M=3
K=1,M=4
K=2,M=1
K=2,M=2
K=3,M=1
K=4,M=1

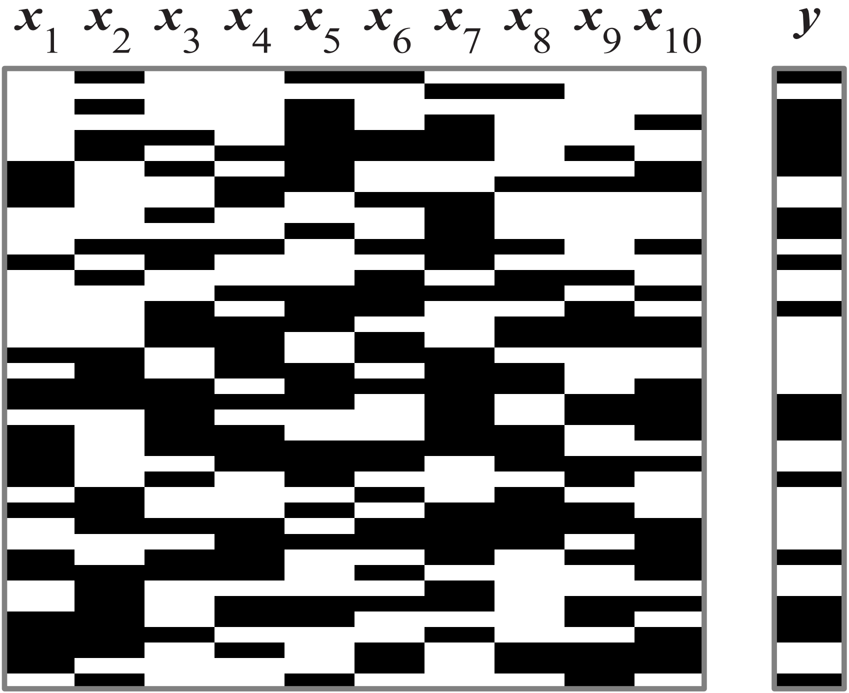

SF17 $y = x_5 \bar{x}_8 + x_3 \bar{x}_6 x_7$

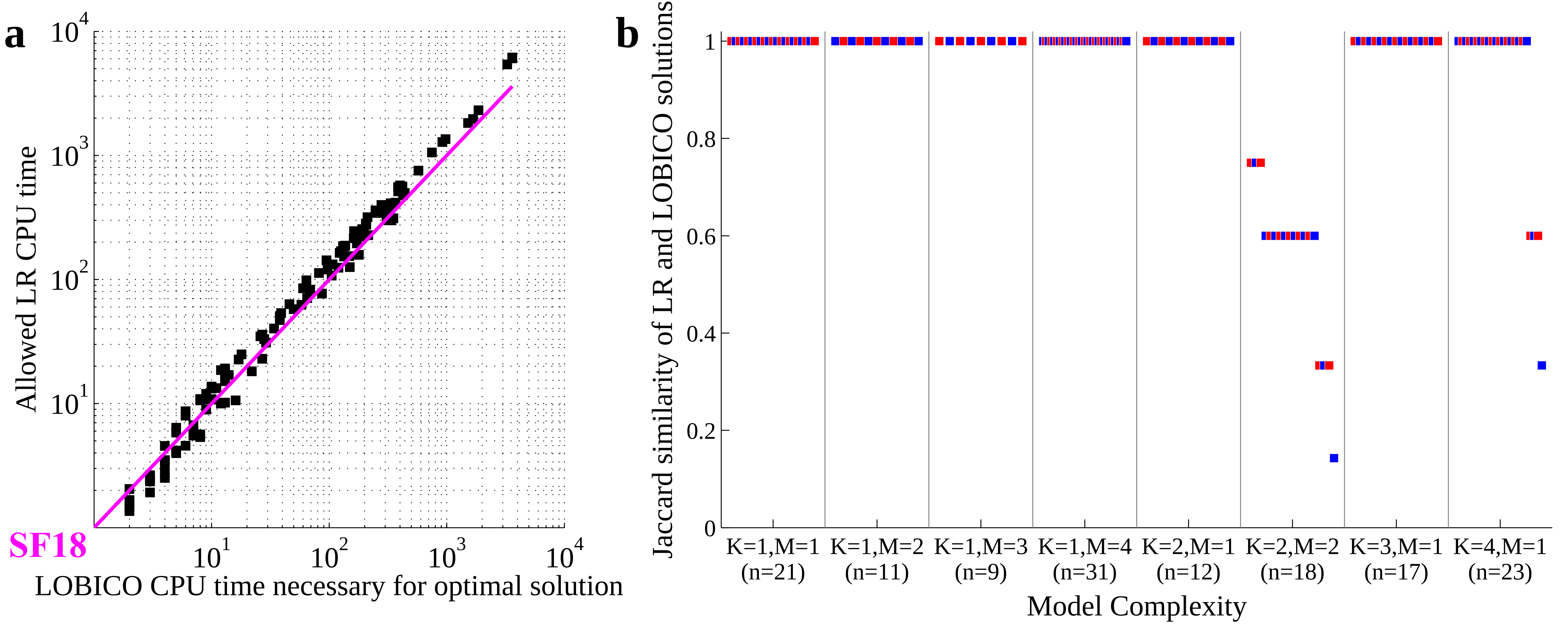
a
Allowed LR CPU time
LOBICO CPU time necessary for optimal solution
SF18
b
Jaccard similarity of LR and LOBICO solutions
Model Complexity
K=1,M=1 (n=21)
K=1,M=2 (n=11)
K=1,M=3 (n=9)
K=1,M=4 (n=31)
K=2,M=1 (n=12)
K=2,M=2 (n=18)
K=3,M=1 (n=17)
K=4,M=1 (n=23)
