# Supplementary material for: Logic models to predict continuous outputs based on binary inputs with an application to personalized cancer therapy
Source: Sci Rep. 2016 Nov 23;6:36812. doi: 10.1038/srep36812 (PMC5120272; doi:10.1038/srep36812)
Supplement: Supplementary Dataset 1 [file srep36812-s2.zip › SD1.pdf]

Visual explanation of the logic models for a single drug inferred by LOBICO

ID:1 Erlotinib –> EGFR

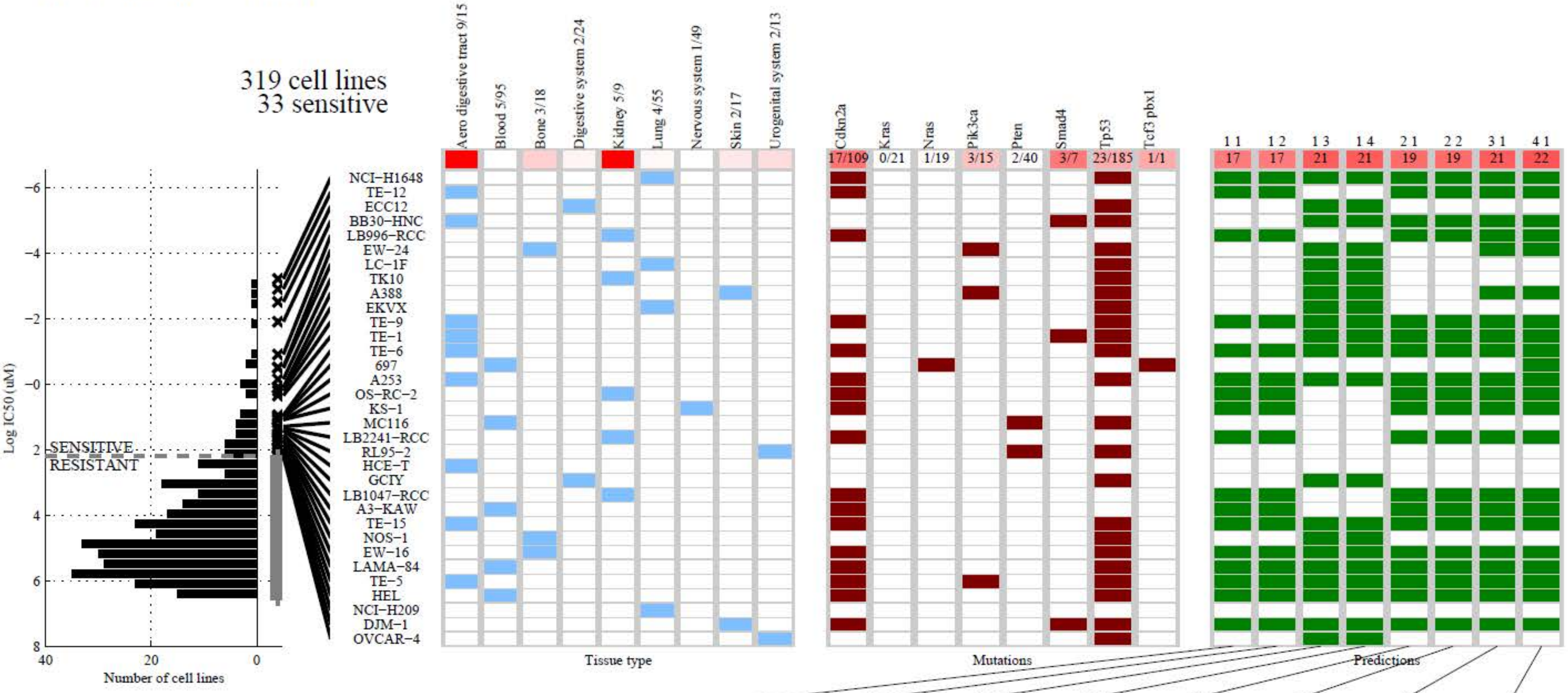

Model name	1 1	1 2	1 3	1 4	2 1	2 2	3 1	4 1
K / M	1 / 1	1 / 2	1 / 3	1 / 4	2 / 1	2 / 2	3 / 1	4 / 1
Logic formula	CDKN2	CDKN2&¬PTEN	¬KRAS&¬PTEN& TP53	¬KRAS&¬NRAS& ¬PTEN& TP53	CDKN2 | SMAD4	[¬KRAS&SMAD4] | [CDKN2&¬PTEN]	CDKN2 | PIK3C | SMAD4	CDKN2 | PIK3C | SMAD4 | TCF3
TP / FP	17 / 92	17 / 75	21 / 120	21 / 113	19 / 95	19 / 77	21 / 104	22 / 104
FN / TN	16 / 194	16 / 211	12 / 166	12 / 173	14 / 191	14 / 209	12 / 182	11 / 182
Specificity	0.68	0.74	0.58	0.6	0.67	0.73	0.64	0.64
Precision	0.16	0.18	0.15	0.16	0.17	0.2	0.17	0.17
Recall	0.52	0.52	0.64	0.64	0.58	0.58	0.64	0.67

Information about the drug and the cell lines sensitive to the drug

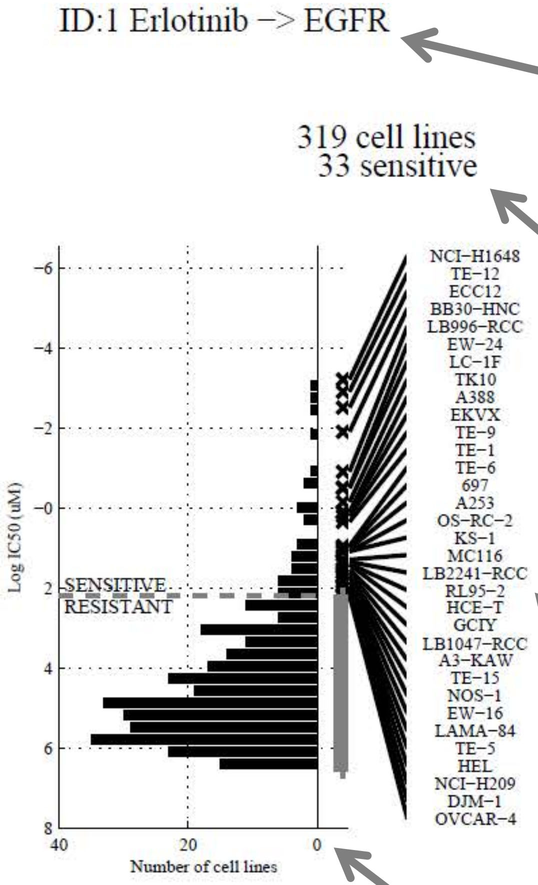

Drug ID followed by the name of the drug (if available) and, to the right of '->' the target(s) of the drug (if available).

Number of cell lines for which drug response values (IC50s) are available, and below, the number of cell lines that are sensitive to the drug.

Names of the sensitive cell lines with the most sensitive (lowest IC50) at the top.

Histogram of all (log transformed) IC50s. The grey dashed line represents the threshold used to decide whether cell lines are sensitive or resistant.

Tissue of origin of the sensitive cell lines

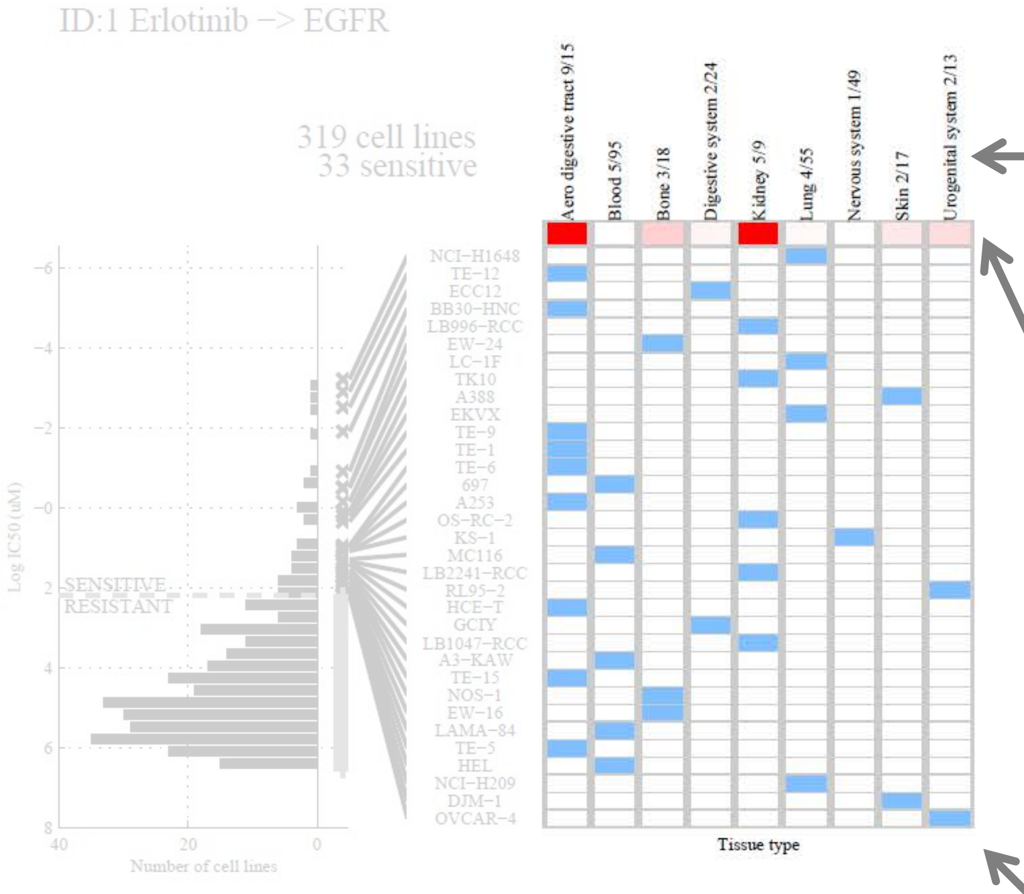

Tissue type labels followed by '*x*/*y*', where *x* is the number of sensitive cell lines with the indicated tissue type, and *y* is the total number of cell lines from that tissue type

White to red heatmap indicating whether cell lines from the indicated tissue type are enriched within the group of sensitive cell lines. Bright red indicates strong enrichment. White indicates no enrichment.

Example: Of the 33 sensitive cell lines, 5 are 'kidney' lines (5th column), which is a significant enrichment given that there are 9 'kidney' lines in the total set of 319 cell lines.

Binary heatmap indicating for each of the sensitive cell lines the tissue of origin (indicated by light blue).

Example: The most sensitive cell line (NCI-H1648, first row) is a 'lung' cell line.

Mutation status of the sensitive cell lines

Labels of the genomic features used in one or more of the inferred logic models

White to red heatmap indicating whether cell lines that have a mutation of the indicated genomic feature are enriched within the group of sensitive cell lines. Bright red indicates strong enrichment. White indicates no enrichment. The text in the box is '*x*/*y*', where *x* is the number of mutated sensitive cell lines, and *y* is the total number of cell lines with a mutation of that feature.

Example: Of the 33 sensitive cell lines, 3 have a 'Smad4' mutation (6th column), which is a significant enrichment given that there are 7 lines with a 'Smad4' mutation in the total set of 319 cell lines.

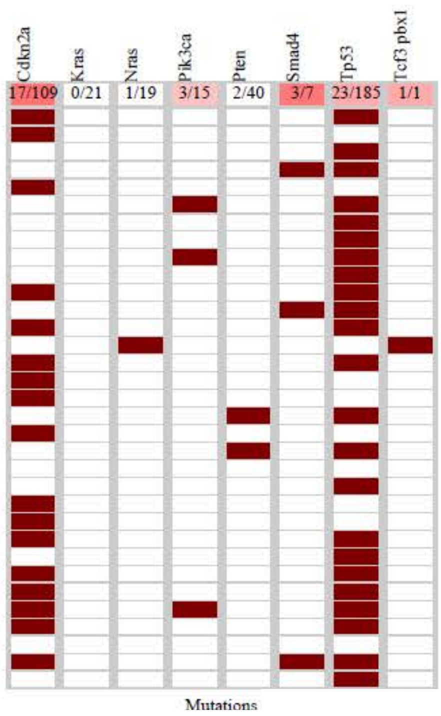

Binary heatmap showing for each of the sensitive cell lines whether they have a mutation of the indicated feature (dark red).

Correctly and incorrectly predicted sensitive cell lines

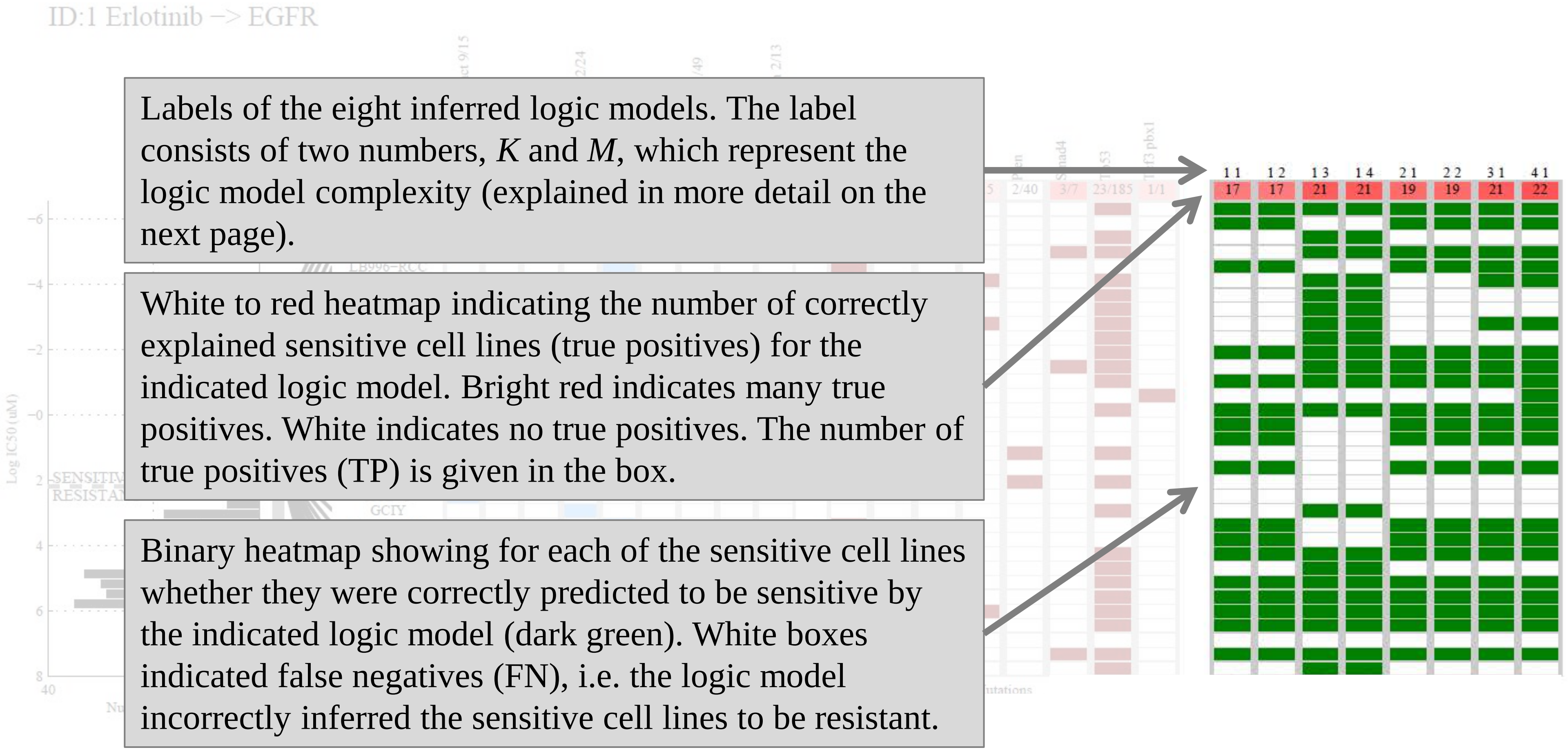

Labels of the eight inferred logic models. The label consists of two numbers, *K* and *M*, which represent the logic model complexity (explained in more detail on the next page).

White to red heatmap indicating the number of correctly explained sensitive cell lines (true positives) for the indicated logic model. Bright red indicates many true positives. White indicates no true positives. The number of true positives (TP) is given in the box.

Binary heatmap showing for each of the sensitive cell lines whether they were correctly predicted to be sensitive by the indicated logic model (dark green). White boxes indicated false negatives (FN), i.e. the logic model incorrectly inferred the sensitive cell lines to be resistant.

Formulas and performance statistics for the logic models

ID:1 Erlotinib –> EGFR

‘Explanation box’ for the eight logic model boxes to the right including:
- Model name and complexity
- The logic formula of genomic features to predict drug sensitivity
- Contingency table and performance statistics

Boxes with the formula and performance statistics for each of the eight logic models. The top part of the box indicates the model complexity (as defined by *K* and *M*), i.e. the type of logic model, from single predictor model to multi-input AND and OR models. The middle part lists the logic formula. &, | and ¬ indicate AND, OR and NOT, respectively. The color of a genomic feature indicates its importance with bright red meaning very important and black not important. The lower part gives several performance criteria.

The logic model with the best performance according to cross-validation is highlighted in orange.

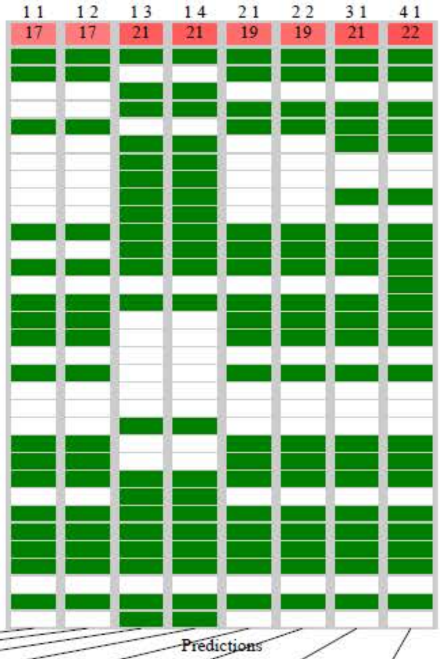

	Single predictor	2-input AND	3-input AND	4-input AND	2-input OR	2-by-2	3-input OR	4-input OR
Model name<br>K M	1 1<br>1 1	1 2<br>1 2	1 3<br>1 3	1 4<br>1 4	2 1<br>2 1	2 2<br>2 2	3 1<br>3 1	4 1<br>4 1
Logic formula	CDKN2	CDKN2&¬PTEN	¬KRAS&¬PTEN& TP53	¬KRAS&¬NRAS& ¬PTEN& TP53	CDKN2 | SMAD4	[¬KRAS&SMAD4] | [CDKN2&¬PTEN]	CDKN2 | PIK3C | SMAD4	CDKN2 | PIK3C | SMAD4 | TCF3
TP FP / FN TN	17 92 / 16 194	17 75 / 16 211	21 120 / 12 166	21 113 / 12 173	19 95 / 14 191	19 77 / 14 209	21 104 / 12 182	22 104 / 11 182
Specificity	0.68	0.74	0.58	0.6	0.67	0.73	0.64	0.64
Precision	0.16	0.18	0.15	0.16	0.17	0.2	0.17	0.17
Recall	0.52	0.52	0.64	0.64	0.58	0.58	0.64	0.67

Complete visualization of the logic models for a single drug inferred by LOBICO

ID:1 Erlotinib –> EGFR

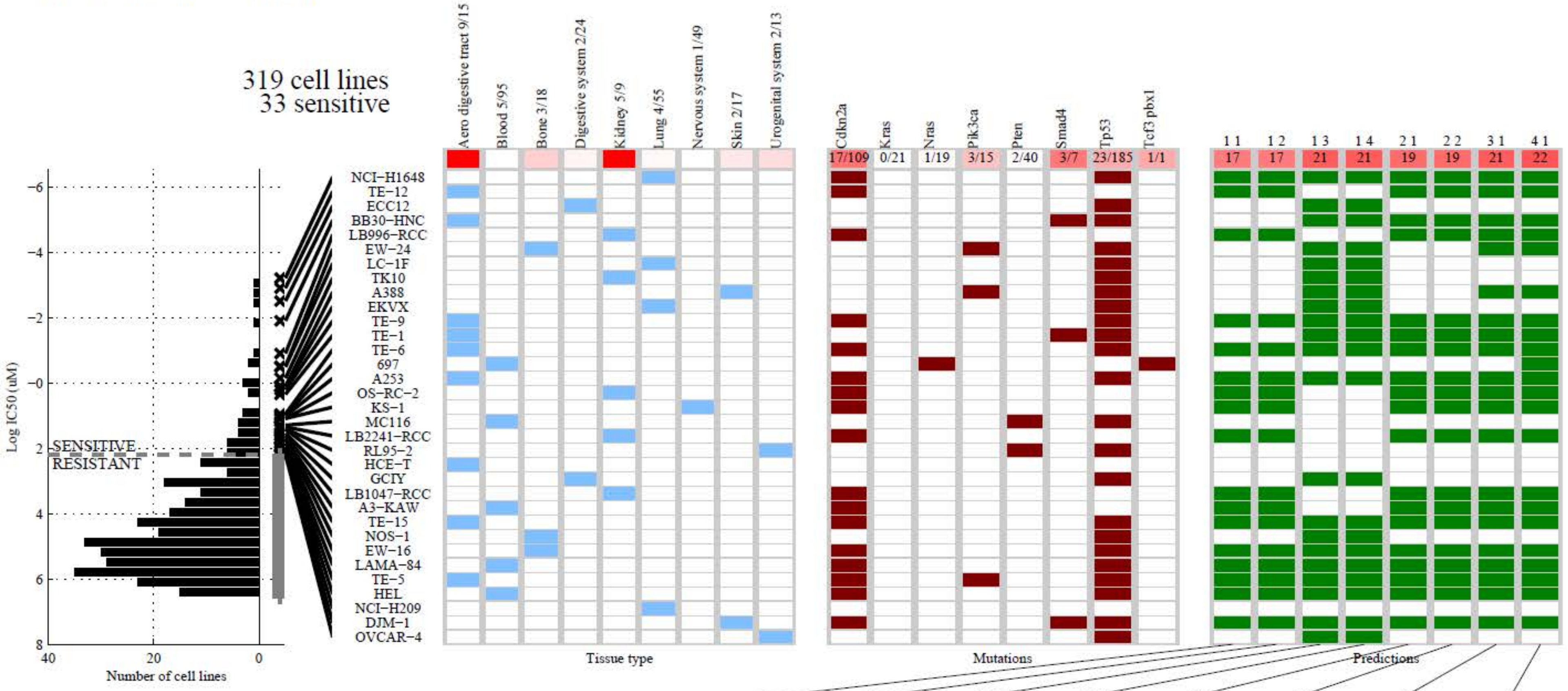

<table>
<tr><td>Model name
K M</td><td>1 1
1 1</td><td>1 2
1 2</td><td>1 3
1 3</td><td>1 4
1 4</td><td>2 1
2 1</td><td>2 2
2 2</td><td>3 1
3 1</td><td>4 1
4 1</td></tr>
<tr><td>Logic formula</td><td>CDKN2</td><td>CDKN2&¬PTEN</td><td>¬KRAS&¬PTEN& TP53</td><td>¬KRAS&¬NRAS& ¬PTEN& TP53</td><td>CDKN2 | SMAD4</td><td>[¬KRAS&SMAD4] | [CDKN2&¬PTEN]</td><td>CDKN2 | PIK3C | SMAD4</td><td>CDKN2 | PIK3C | SMAD4 | TCF3</td></tr>
<tr><td>TP FP / FN TN
Specificity
Precision
Recall</td><td>17 92 / 16 194
0.68
0.16
0.52</td><td>17 75 / 16 211
0.74
0.18
0.52</td><td>21 120 / 12 166
0.58
0.15
0.64</td><td>21 113 / 12 173
0.6
0.16
0.64</td><td>19 95 / 14 191
0.67
0.17
0.58</td><td>19 77 / 14 209
0.73
0.2
0.58</td><td>21 104 / 12 182
0.64
0.17
0.64</td><td>22 104 / 11 182
0.64
0.17
0.67</td></tr>
</table>

ID:1 Erlotinib −> EGFR

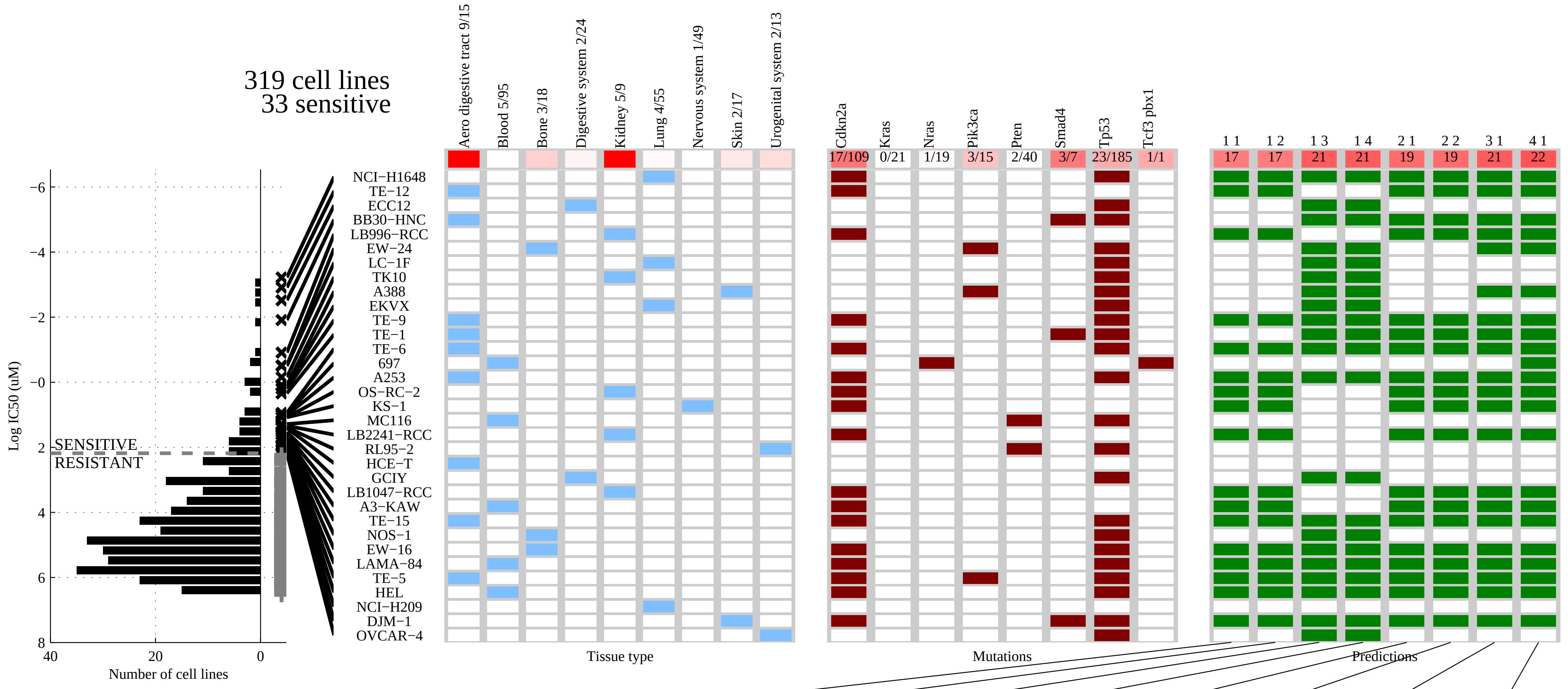

Model name	1 1	1 2	1 3	1 4	2 1	2 2	3 1	4 1
K / M	1 / 1	1 / 2	1 / 3	1 / 4	2 / 1	2 / 2	3 / 1	4 / 1
Logic formula	CDKN2	CDKN2&¬PTEN	¬KRAS&¬PTEN& TP53	¬KRAS&¬NRAS& ¬PTEN& TP53	CDKN2 | SMAD4	[¬KRAS&SMAD4] | [CDKN2&¬PTEN]	CDKN2 | PIK3C | SMAD4	CDKN2 | PIK3C | SMAD4 | TCF3
TP FP / FN TN	17 92 / 16 194	17 75 / 16 211	21 120 / 12 166	21 113 / 12 173	19 95 / 14 191	19 77 / 14 209	21 104 / 12 182	22 104 / 11 182
Specificity	0.68	0.74	0.58	0.6	0.67	0.73	0.64	0.64
Precision	0.16	0.18	0.15	0.16	0.17	0.2	0.17	0.17
Recall	0.52	0.52	0.64	0.64	0.58	0.58	0.64	0.67

ID:1000 Metformin −> AMPK agonist

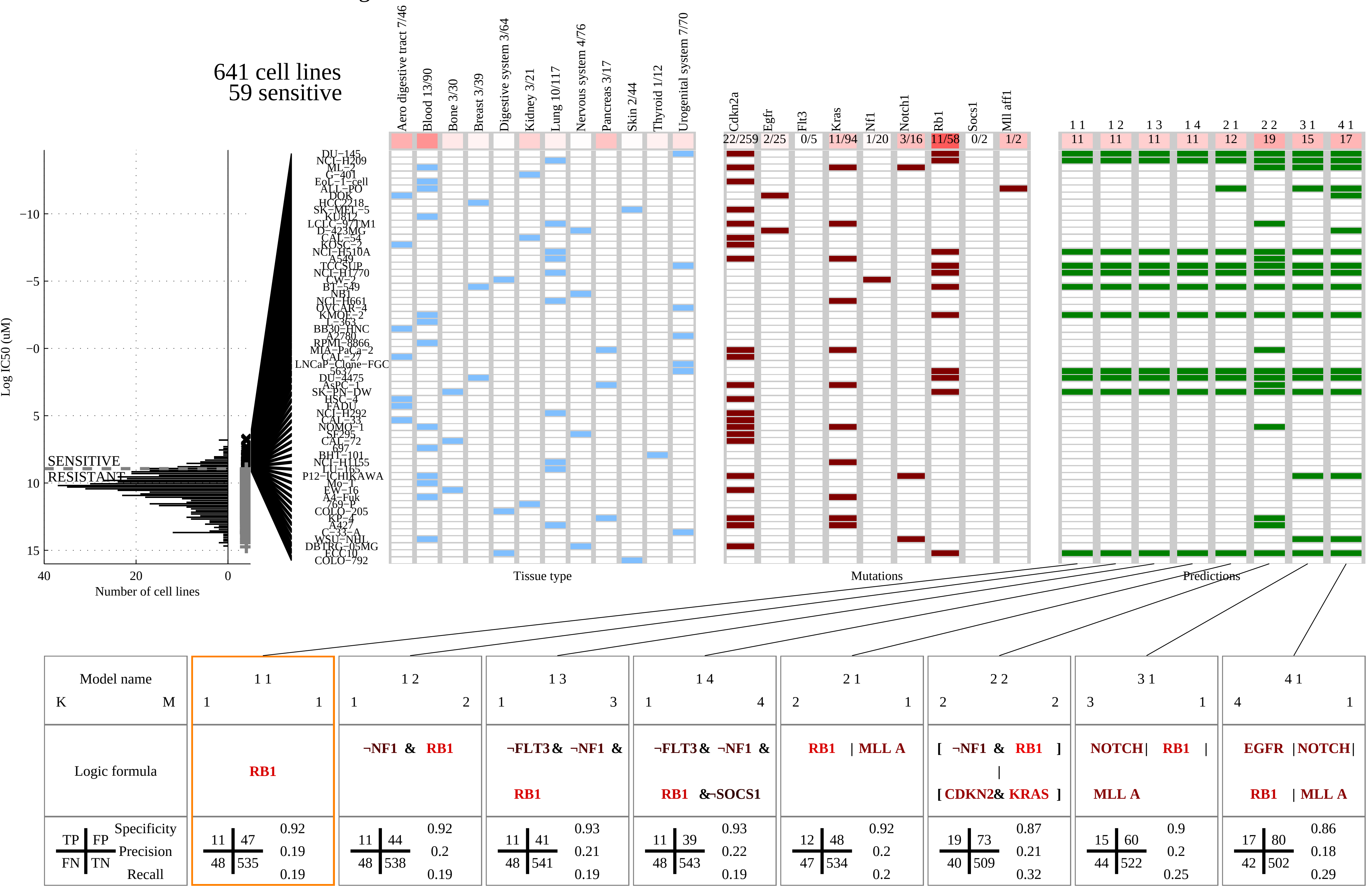

ID:1001 AICAR –> AAPK1 (AMPK) agonist

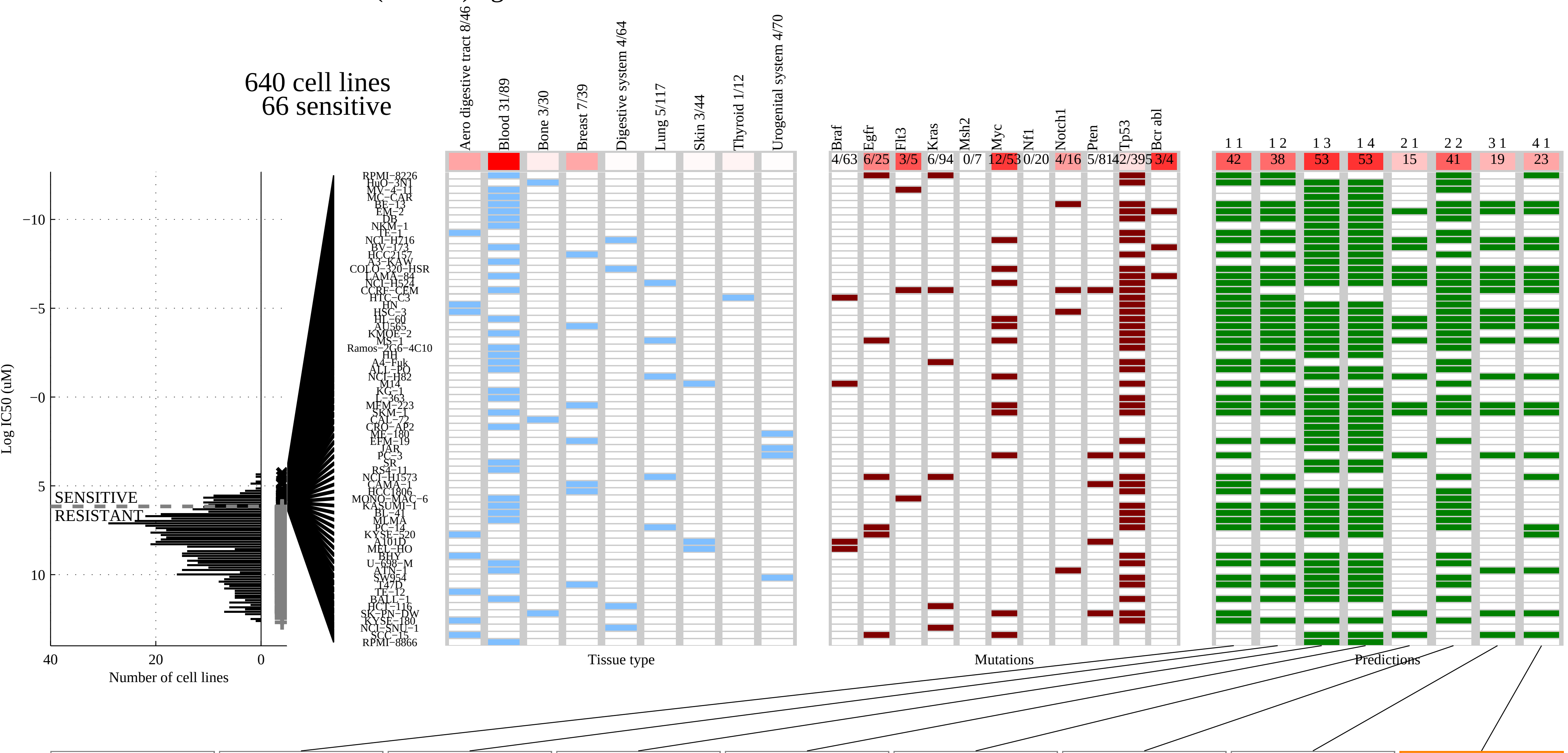

Model name<br>K M	1 1<br>1 1	1 2<br>1 2	1 3<br>1 3	1 4<br>1 4	2 1<br>2 1	2 2<br>2 2	3 1<br>3 1	4 1<br>4 1
Logic formula	TP53	¬PTEN & TP53	¬BRAF & ¬KRAS & ¬PTEN	¬BRAF & ¬KRAS & ¬NF1 & ¬PTEN	MYC | BCR A	[¬PTEN & TP53] | [FLT3 & ¬MSH2]	MYC | NOTCH | BCR A	EGFR | MYC | NOTCH | BCR A
TP FP<br>FN TN	42 353<br>24 221	38 299<br>28 275	53 370<br>13 204	53 358<br>13 216	15 42<br>51 532	41 299<br>25 275	19 53<br>47 521	23 68<br>43 506
Specificity	0.39	0.48	0.36	0.38	0.93	0.48	0.91	0.88
Precision	0.11	0.11	0.13	0.13	0.26	0.12	0.26	0.25
Recall	0.64	0.58	0.8	0.8	0.23	0.62	0.29	0.35

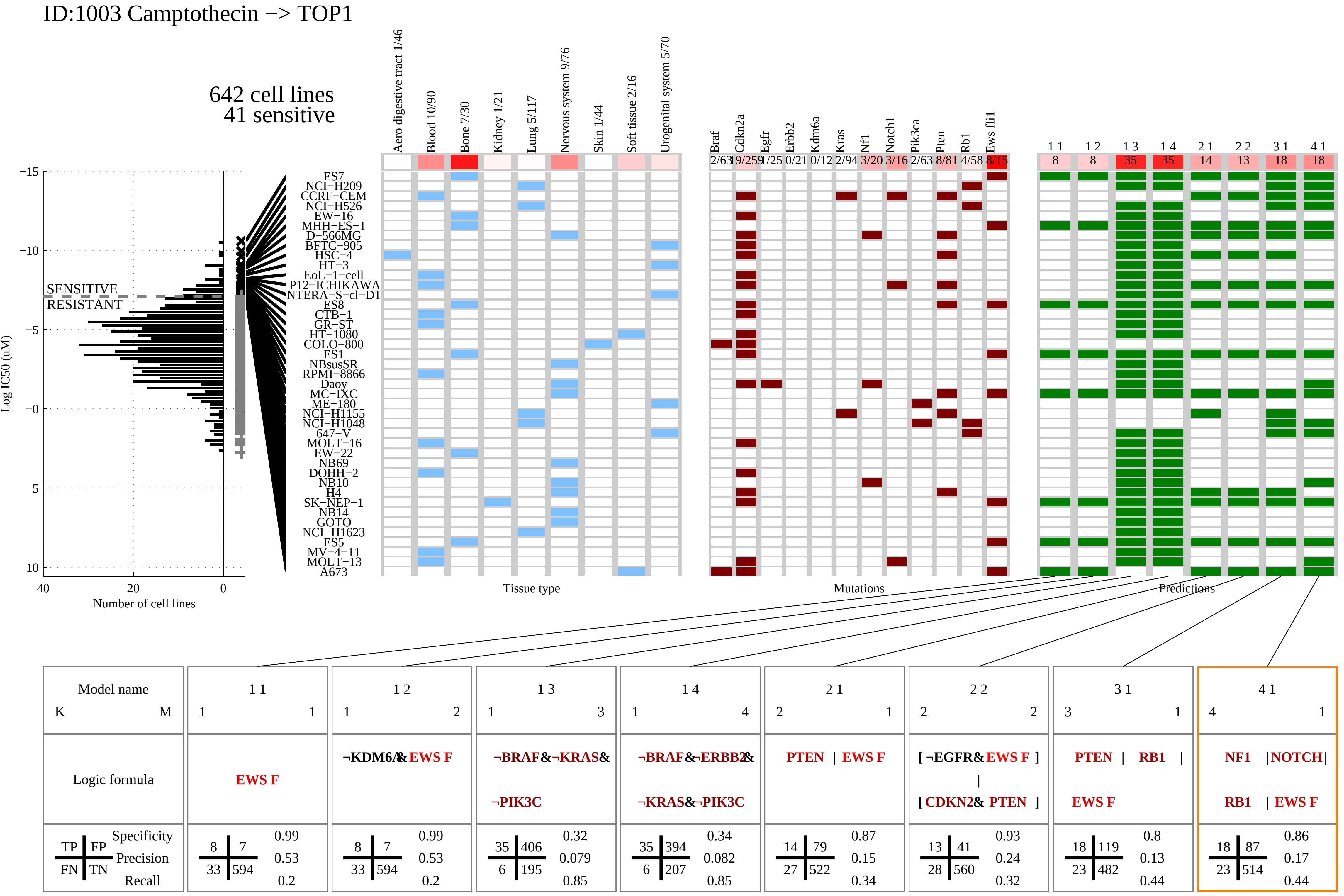
ID:1003 Camptothecin −> TOP1
642 cell lines
41 sensitive
Aero digestive tract 1/46
Blood 10/90
Bone 7/30
Kidney 1/21
Lung 5/117
Nervous system 9/76
Skin 1/44
Soft tissue 2/16
Urogenital system 5/70
Braf 2/63
Cdkn2a 19/259
Egfr 1/25
Erbb2 0/21
Kdm6a 0/12
Kras 2/94
Nf1 3/20
Notch1 3/16
Pik3ca 2/63
Pten 8/81
Rb1 4/58
Ews fli1 8/15
1 1 8
1 2 8
1 3 35
1 4 35
2 1 14
2 2 13
3 1 18
4 1 18
ES7
NCI−H209
CCRF−CEM
NCI−H526
EW−16
MHH−ES−1
D−566MG
BFTC−905
HSC−4
HT−3
EoL−1−cell
P12−ICHIKAWA
NTERA−S−cl−D1
ES8
CTB−1
GR−ST
HT−1080
COLO−800
ES1
NBsusSR
RPMI−8866
Daoy
MC−IXC
ME−180
NCI−H1155
NCI−H1048
647−V
MOLT−16
EW−22
NB69
DOHH−2
NB10
H4
SK−NEP−1
NB14
GOTO
NCI−H1623
ES5
MV−4−11
MOLT−13
A673
SENSITIVE
RESISTANT
Log IC50 (uM)
Number of cell lines
Tissue type
Mutations
Predictions
Model name
K
M
Logic formula
TP FP FN TN
Specificity
Precision
Recall
1 1
EWS F
8 7 33 594
0.99 0.53 0.2
1 2
¬KDM6A & EWS F
8 7 33 594
0.99 0.53 0.2
1 3
¬BRAF & ¬KRAS & ¬PIK3C
35 406 6 195
0.32 0.079 0.85
1 4
¬BRAF & ¬ERBB2 & ¬KRAS & ¬PIK3C
35 394 6 207
0.34 0.082 0.85
2 1
PTEN | EWS F
14 79 27 522
0.87 0.15 0.34
2 2
[¬EGFR & EWS F] | [CDKN2 & PTEN]
13 41 28 560
0.93 0.24 0.32
3 1
PTEN | RB1 | EWS F
18 119 23 482
0.8 0.13 0.44
4 1
NF1 | NOTCH | RB1 | EWS F
18 87 23 514
0.86 0.17 0.44

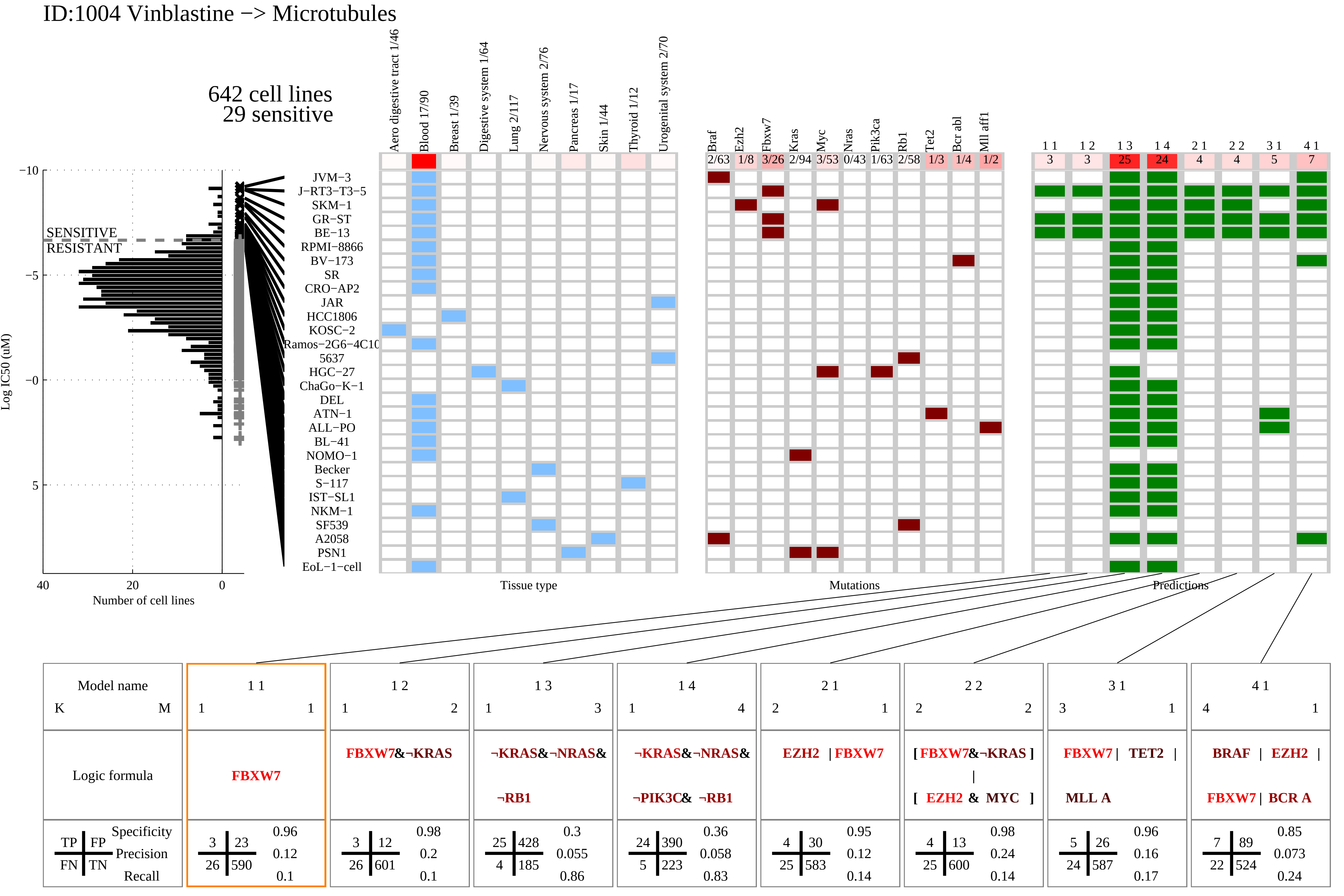

ID:1004 Vinblastine −> Microtubules
642 cell lines
29 sensitive
SENSITIVE
RESISTANT
Log IC50 (uM)
Number of cell lines
Aero digestive tract 1/46
Blood 17/90
Breast 1/39
Digestive system 1/64
Lung 2/117
Nervous system 2/76
Pancreas 1/17
Skin 1/44
Thyroid 1/12
Urogenital system 2/70
Braf 2/63
Ezh2 1/8
Fbxw7 3/26
Kras 2/94
Myc 3/53
Nras 0/43
Pik3ca 1/63
Rb1 2/58
Tet2 1/3
Bcr abl 1/4
Mll aff1 1/2
JVM−3
J−RT3−T3−5
SKM−1
GR−ST
BE−13
RPMI−8866
BV−173
SR
CRO−AP2
JAR
HCC1806
KOSC−2
Ramos−2G6−4C10
5637
HGC−27
ChaGo−K−1
DEL
ATN−1
ALL−PO
BL−41
NOMO−1
Becker
S−117
IST−SL1
NKM−1
SF539
A2058
PSN1
EoL−1−cell
Tissue type
Mutations
Predictions
Model name K M
Logic formula
TP FP FN TN
Specificity Precision Recall
1 1: FBXW7; 3 23 26 590; 0.96 0.12 0.1
1 2: FBXW7&¬KRAS; 3 12 26 601; 0.98 0.2 0.1
1 3: ¬KRAS&¬NRAS& ¬RB1; 25 428 4 185; 0.3 0.055 0.86
1 4: ¬KRAS&¬NRAS& ¬PIK3C& ¬RB1; 24 390 5 223; 0.36 0.058 0.83
2 1: EZH2 | FBXW7; 4 30 25 583; 0.95 0.12 0.14
2 2: [FBXW7&¬KRAS] | [EZH2 & MYC]; 4 13 25 600; 0.98 0.24 0.14
3 1: FBXW7 | TET2 | MLL A; 5 26 24 587; 0.96 0.16 0.17
4 1: BRAF | EZH2 | FBXW7 | BCR A; 7 89 22 524; 0.85 0.073 0.24

ID:1005 Cisplatin –> DNA crosslinker

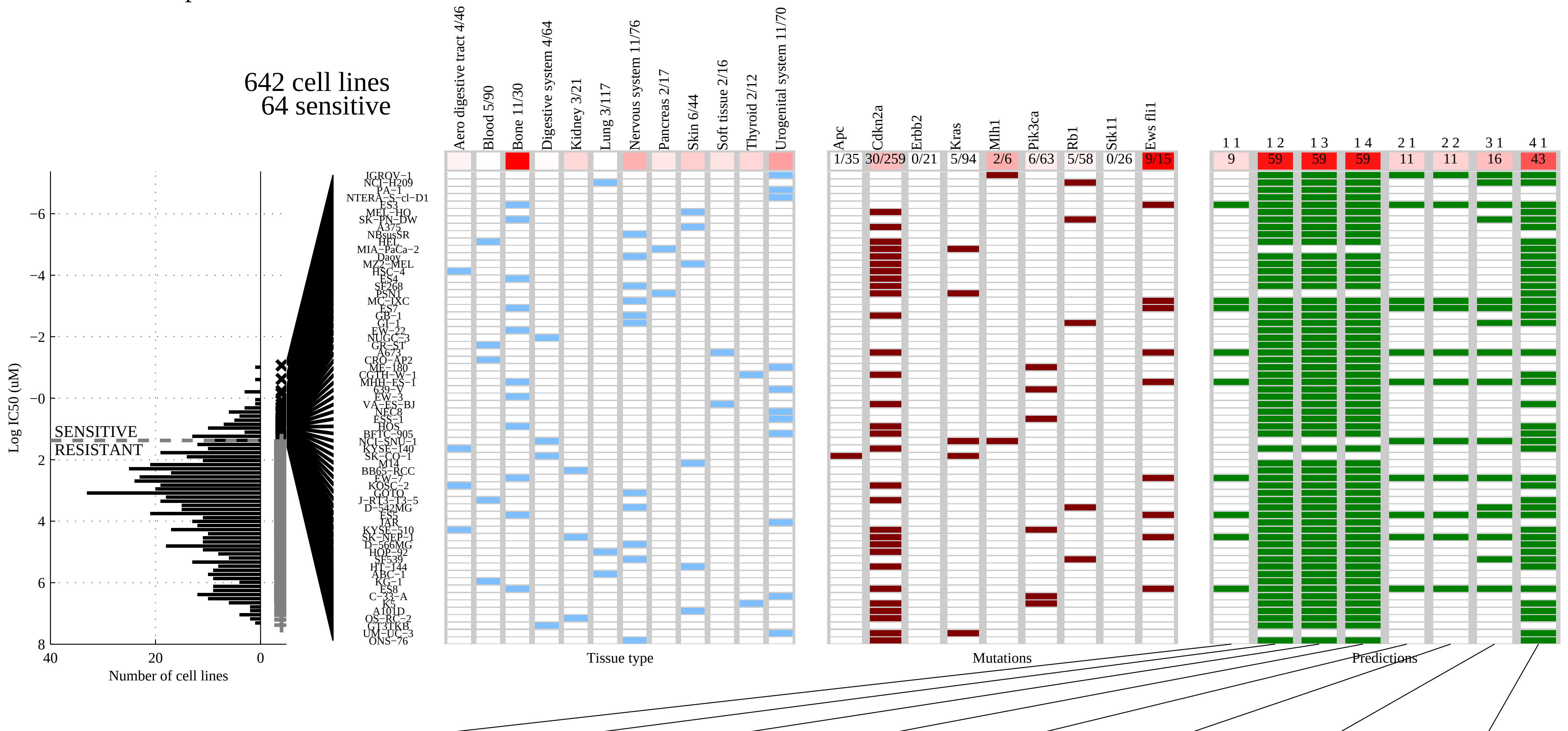

Model name (K, M)	Logic formula	TP	FP	FN	TN	Specificity	Precision	Recall
1 1 (1, 1)	EWS F	9	6	55	572	0.99	0.6	0.14
1 2 (1, 2)	¬ERBB2&¬KRAS	59	470	5	108	0.19	0.11	0.92
1 3 (1, 3)	¬ERBB2&¬KRAS& ¬STK11	59	453	5	125	0.22	0.12	0.92
1 4 (1, 4)	¬APC &¬ERBB2& ¬KRAS&¬STK11	59	437	5	141	0.24	0.12	0.92
2 1 (2, 1)	MLH1 | EWS F	11	10	53	568	0.98	0.52	0.17
2 2 (2, 2)	[MLH1 &¬PIK3C] | [EWS F &]	11	8	53	570	0.99	0.58	0.17
3 1 (3, 1)	MLH1 | RB1 | EWS F	16	63	48	515	0.89	0.2	0.25
4 1 (4, 1)	CDKN2 | MLH1 | RB1 | EWS F	43	280	21	298	0.52	0.13	0.67

ID:1006 Cytarabine −> DNA synthesis

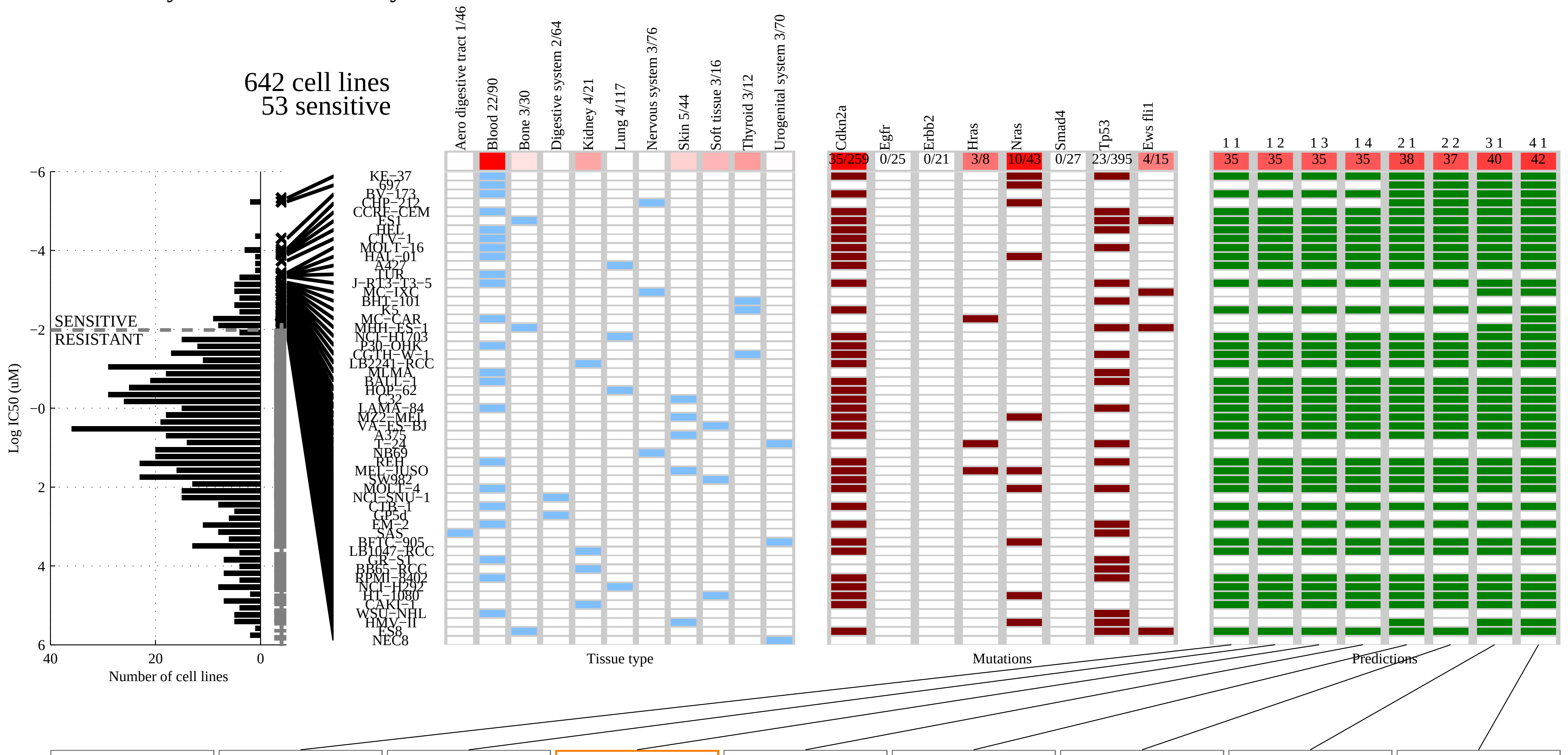

<table>
<tr><td>Model name
K M</td><td>1 1
1 1</td><td>1 2
1 2</td><td>1 3
1 3</td><td>1 4
1 4</td><td>2 1
2 1</td><td>2 2
2 2</td><td>3 1
3 1</td><td>4 1
4 1</td></tr>
<tr><td>Logic formula</td><td>CDKN2</td><td>CDKN2&¬SMAD4</td><td>CDKN2&¬EGFR&¬SMAD4</td><td>CDKN2&¬EGFR&¬ERBB2&¬SMAD4</td><td>CDKN2 | NRAS</td><td>[NRAS & ¬TP53] | [CDKN2&¬SMAD4]</td><td>CDKN2 | NRAS | EWS F</td><td>CDKN2 | HRAS | NRAS | EWS F</td></tr>
<tr><td>TP FP / FN TN</td><td>35 224 / 18 365</td><td>35 209 / 18 380</td><td>35 197 / 18 392</td><td>35 192 / 18 397</td><td>38 239 / 15 350</td><td>37 216 / 16 373</td><td>40 247 / 13 342</td><td>42 249 / 11 340</td></tr>
<tr><td>Specificity</td><td>0.62</td><td>0.65</td><td>0.67</td><td>0.67</td><td>0.59</td><td>0.63</td><td>0.58</td><td>0.58</td></tr>
<tr><td>Precision</td><td>0.14</td><td>0.14</td><td>0.15</td><td>0.15</td><td>0.14</td><td>0.15</td><td>0.14</td><td>0.14</td></tr>
<tr><td>Recall</td><td>0.66</td><td>0.66</td><td>0.66</td><td>0.66</td><td>0.72</td><td>0.7</td><td>0.75</td><td>0.79</td></tr>
</table>

ID:1007 Docetaxel −> Microtubules

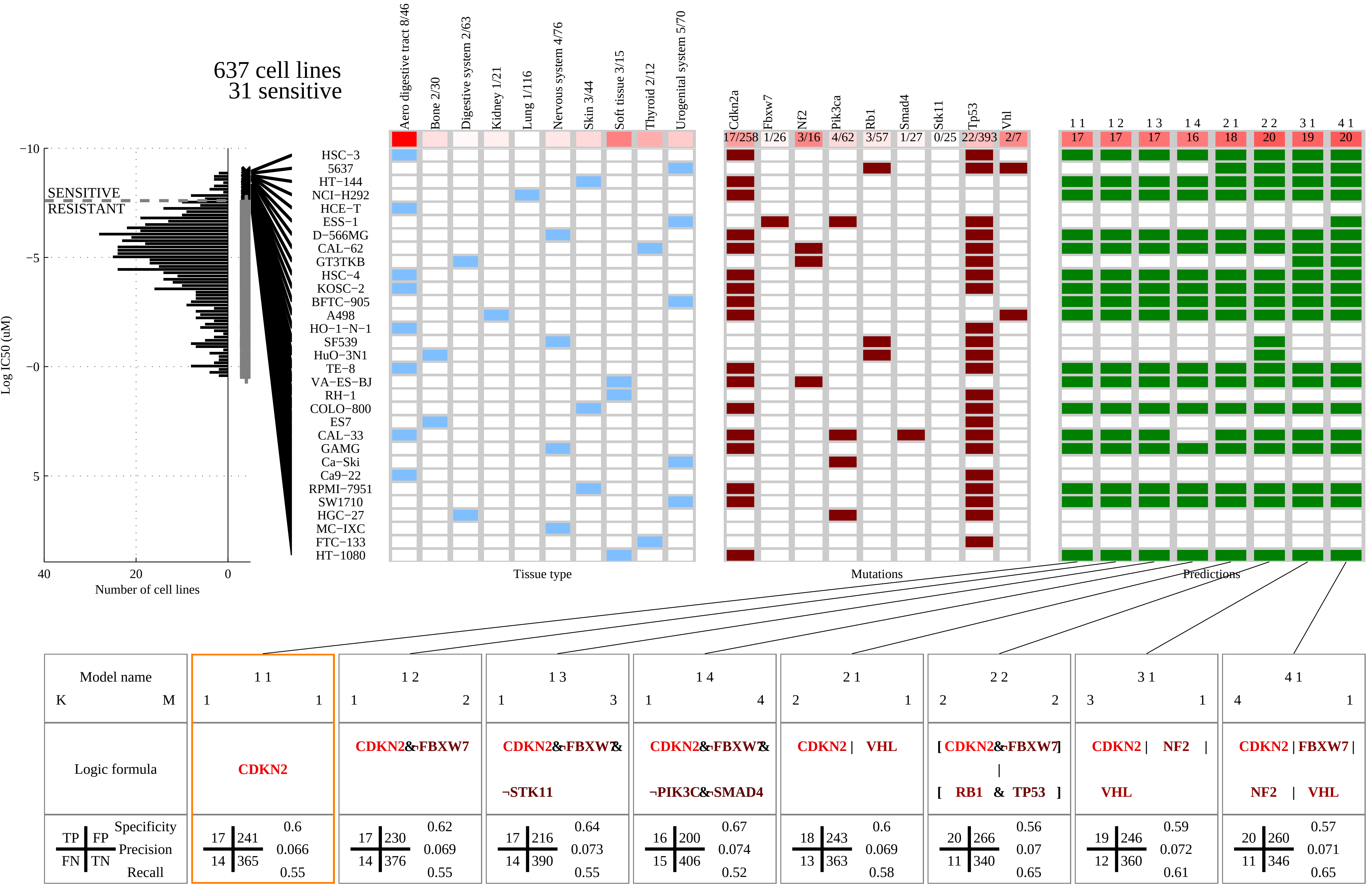

ID:1008 Methotrexate −> Dihydrofolate reductase (DHFR)

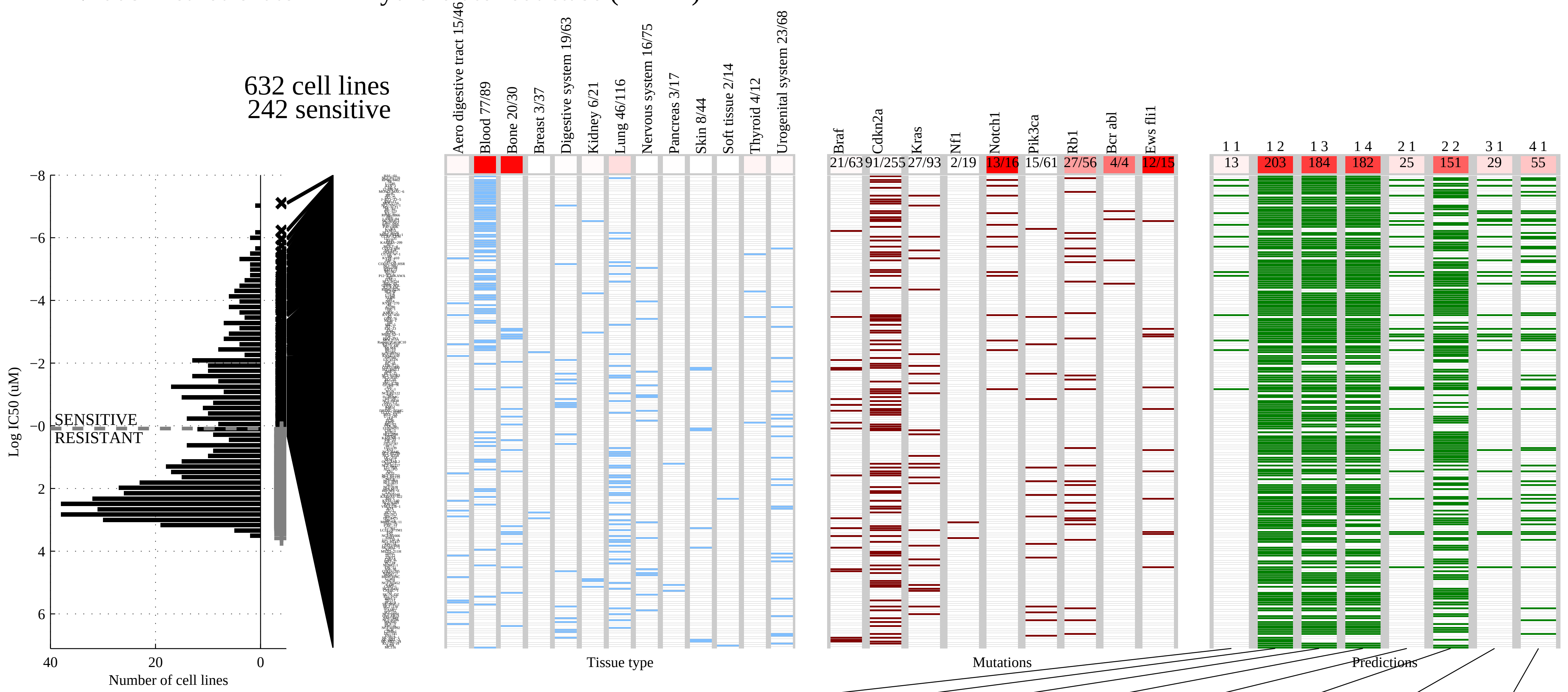

Model name (K, M)	Logic formula	TP	FP	FN	TN	Specificity	Precision	Recall
1 1 (1, 1)	NOTCH	13	3	229	387	0.99	0.81	0.054
1 2 (1, 2)	¬KRAS&¬PIK3C	203	287	39	103	0.26	0.41	0.84
1 3 (1, 3)	¬BRAF&¬KRAS&¬PIK3C	184	249	58	141	0.36	0.42	0.76
1 4 (1, 4)	¬BRAF&¬KRAS&¬NF1 &¬PIK3C	182	235	60	155	0.4	0.44	0.75
2 1 (2, 1)	NOTCH | EWS F	25	6	217	384	0.98	0.81	0.1
2 2 (2, 2)	[¬CDKN2&¬PIK3C] | [NOTCH&¬PIK3C]	151	195	91	195	0.5	0.44	0.62
3 1 (3, 1)	NOTCH | BCR A | EWS F	29	6	213	384	0.98	0.83	0.12
4 1 (4, 1)	NOTCH | RB1 | BCR A | EWS F	55	35	187	355	0.91	0.61	0.23

ID:1009 ATRA −> Retinoic acid and retinoid X receptor agonist

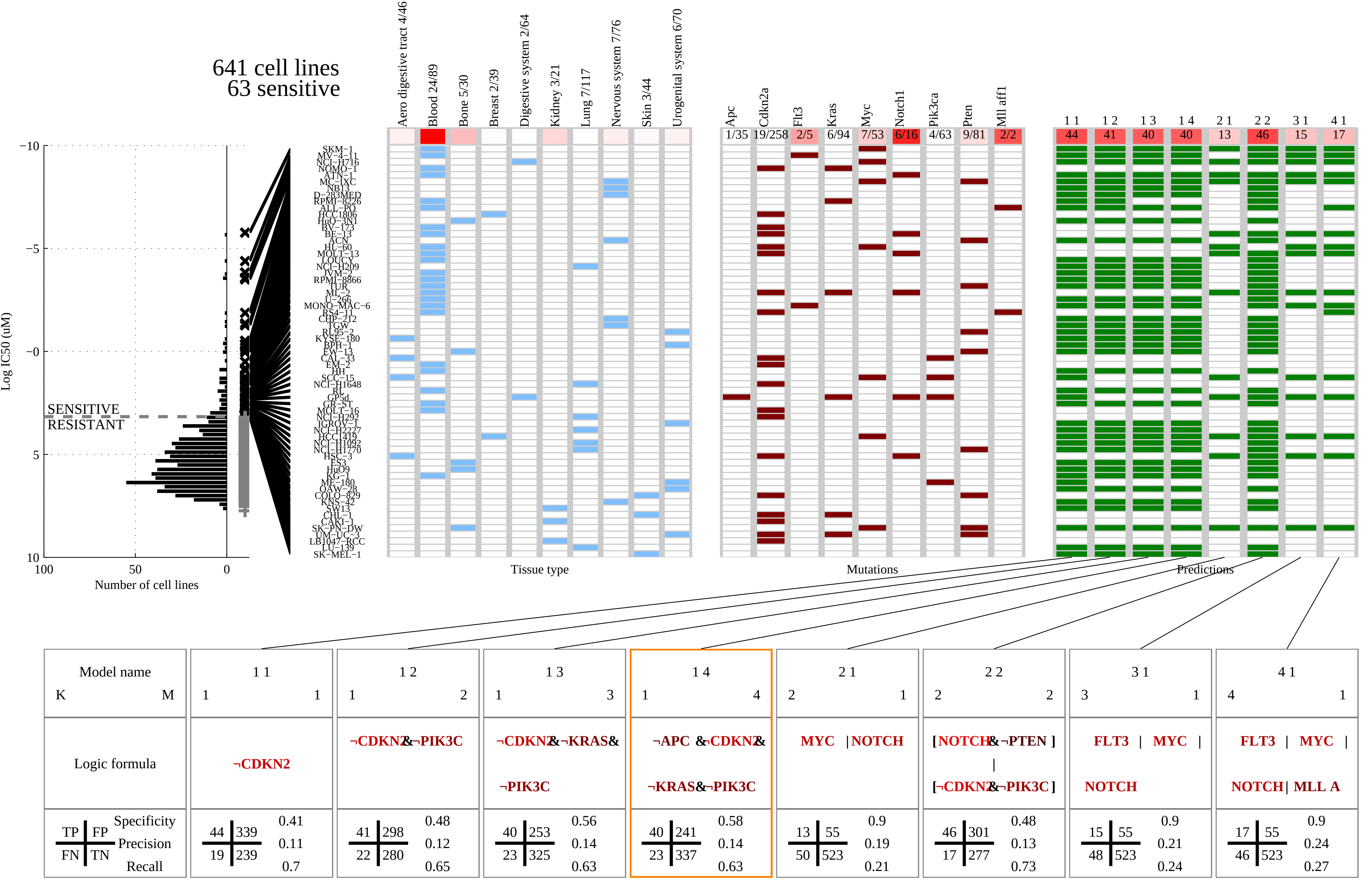

ID:1010 Gefitinib –> EGFR

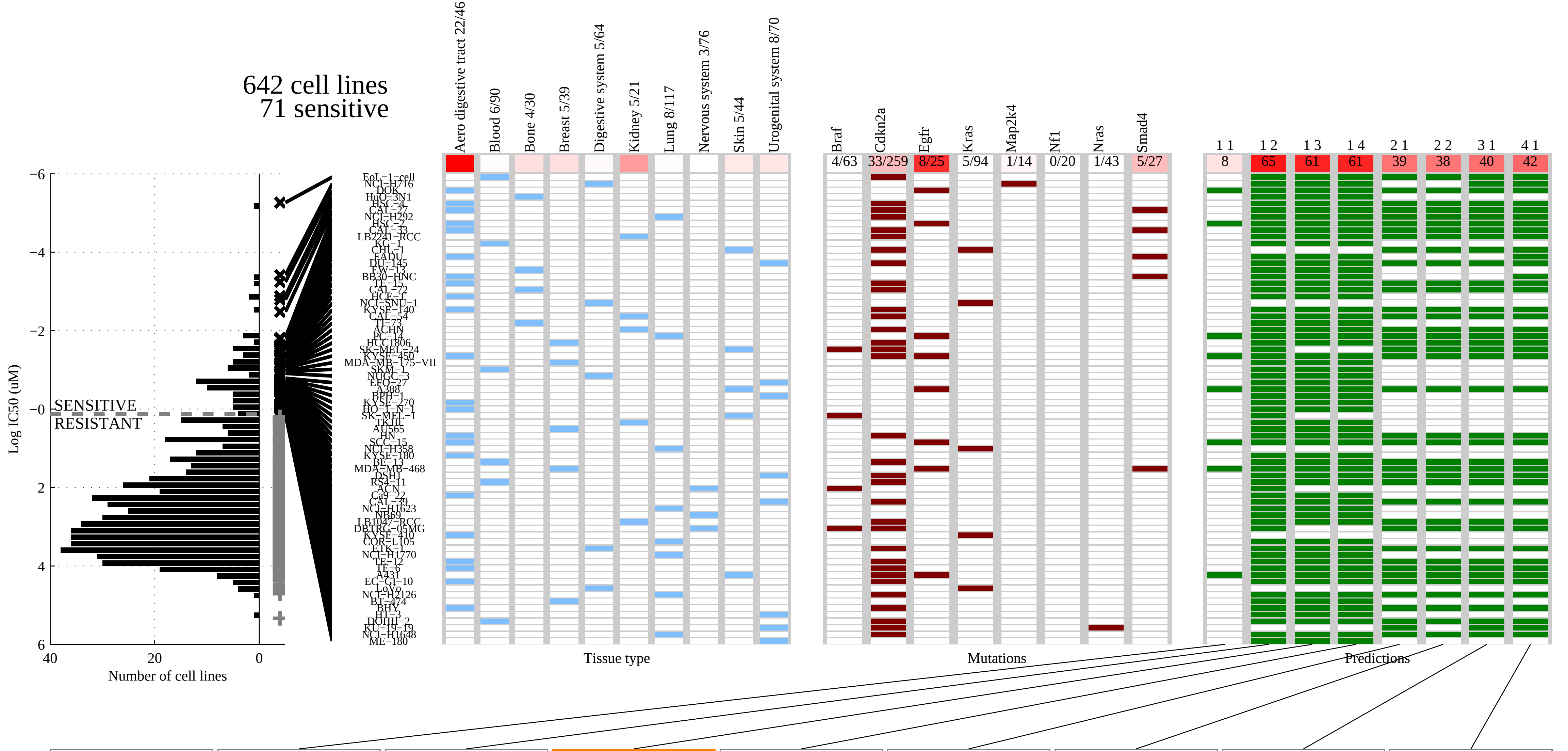

Model name	1 1	1 2	1 3	1 4	2 1	2 2	3 1	4 1
K M	1 1	1 2	1 3	1 4	2 1	2 2	3 1	4 1
Logic formula	EGFR	¬KRAS&¬NRAS	¬BRAF&¬KRAS& ¬NRAS	¬BRAF&¬KRAS& ¬NF1 &¬NRAS	CDKN2 | EGFR	[CDKN2&¬NRAS] | [EGFR &¬KRAS]	CDKN2 | EGFR | MAP2K	CDKN2 | EGFR | MAP2K | SMAD4
TP FP	8 17	65 441	61 387	61 370	39 233	38 207	40 241	42 250
FN TN	63 554	6 130	10 184	10 201	32 338	33 364	31 330	29 321
Specificity	0.97	0.23	0.32	0.35	0.59	0.64	0.58	0.56
Precision	0.32	0.13	0.14	0.14	0.14	0.16	0.14	0.14
Recall	0.11	0.92	0.86	0.86	0.55	0.54	0.56	0.59

ID:1011 ABT−263 −> BCL2, BCL2L1, BCL2L2

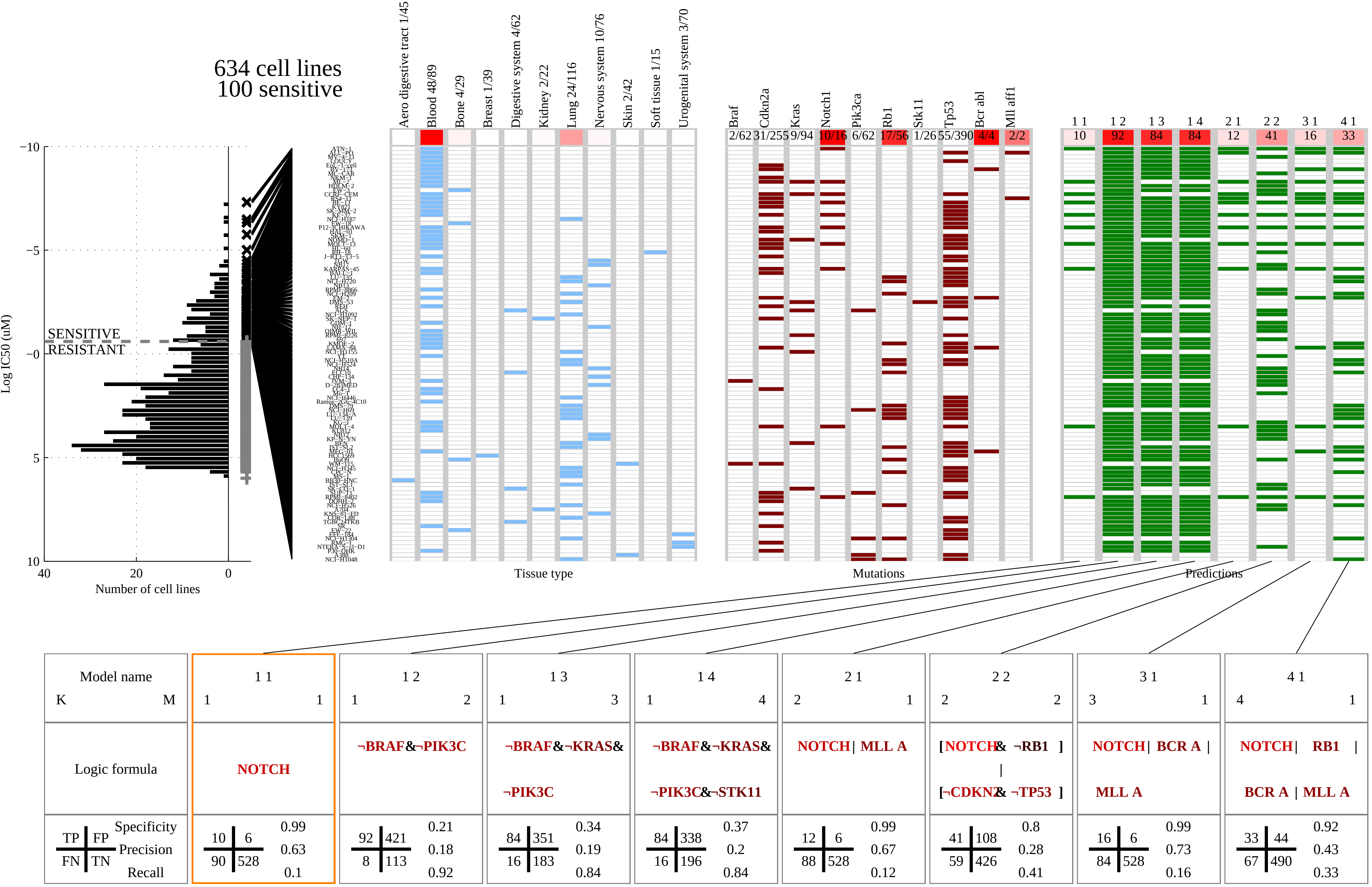

ID:1012 Vorinostat −> HDAC inhibitor Class I, IIa, IIb, IV

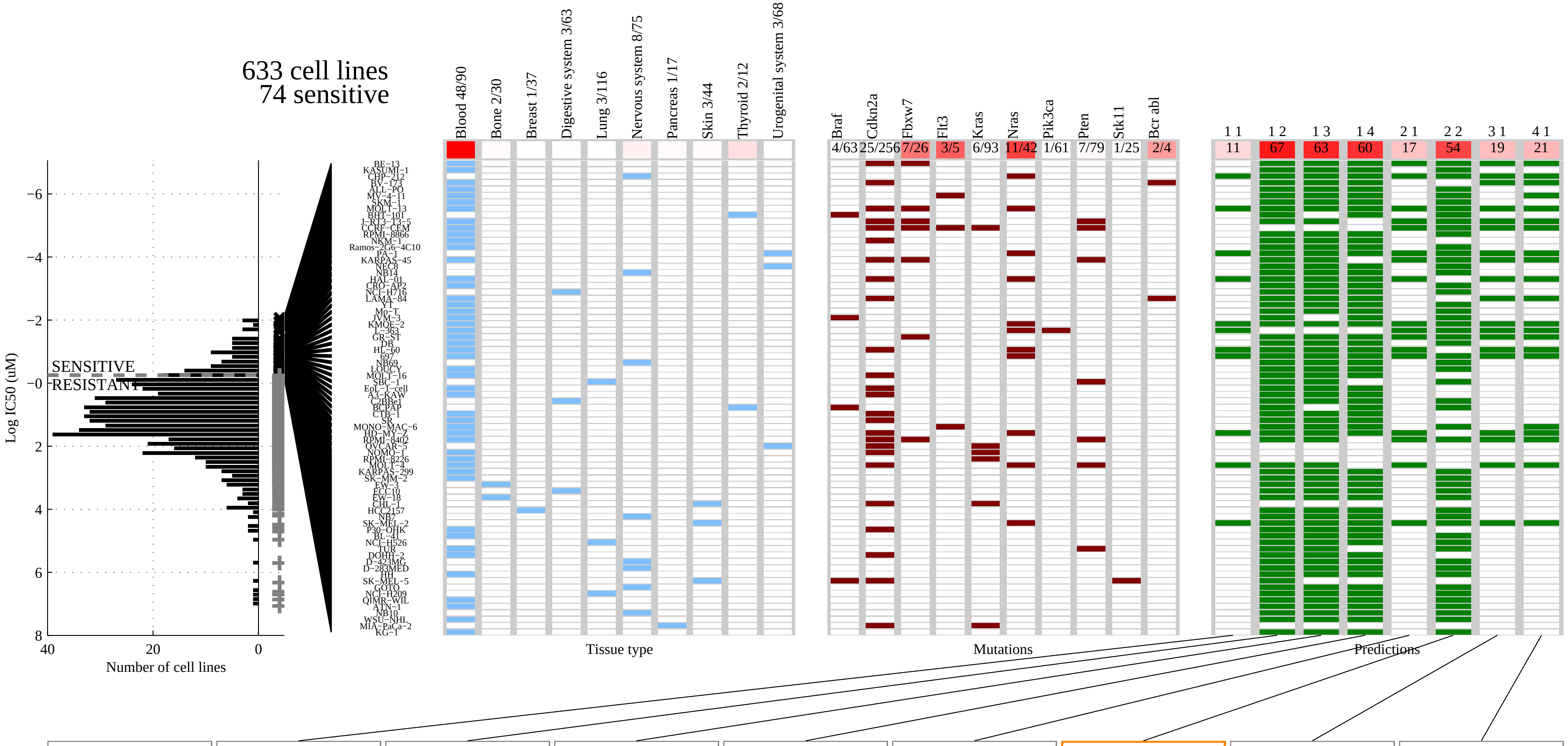

Model name (K, M)	1 1 (1, 1)	1 2 (1, 2)	1 3 (1, 3)	1 4 (1, 4)	2 1 (2, 1)	2 2 (2, 2)	3 1 (3, 1)	4 1 (4, 1)
Logic formula	NRAS	¬KRAS&¬PIK3C	¬BRAF&¬KRAS&¬PIK3C	¬KRAS&¬PIK3C&¬PTEN&¬STK11	FBXW7 | NRAS	[¬CDKN2&¬KRAS] | [CDKN2&FBXW7]	FBXW7 | NRAS | BCR A	FBXW7 | FLT3 | NRAS | BCR A
TP	11	67	63	60	17	54	19	21
FP	31	424	371	343	48	279	50	50
FN	63	7	11	14	57	20	55	53
TN	528	135	188	216	511	280	509	509
Specificity	0.94	0.24	0.34	0.39	0.91	0.5	0.91	0.91
Precision	0.26	0.14	0.15	0.15	0.26	0.16	0.28	0.3
Recall	0.15	0.91	0.85	0.81	0.23	0.73	0.26	0.28

ID:1013 Nilotinib –> ABL

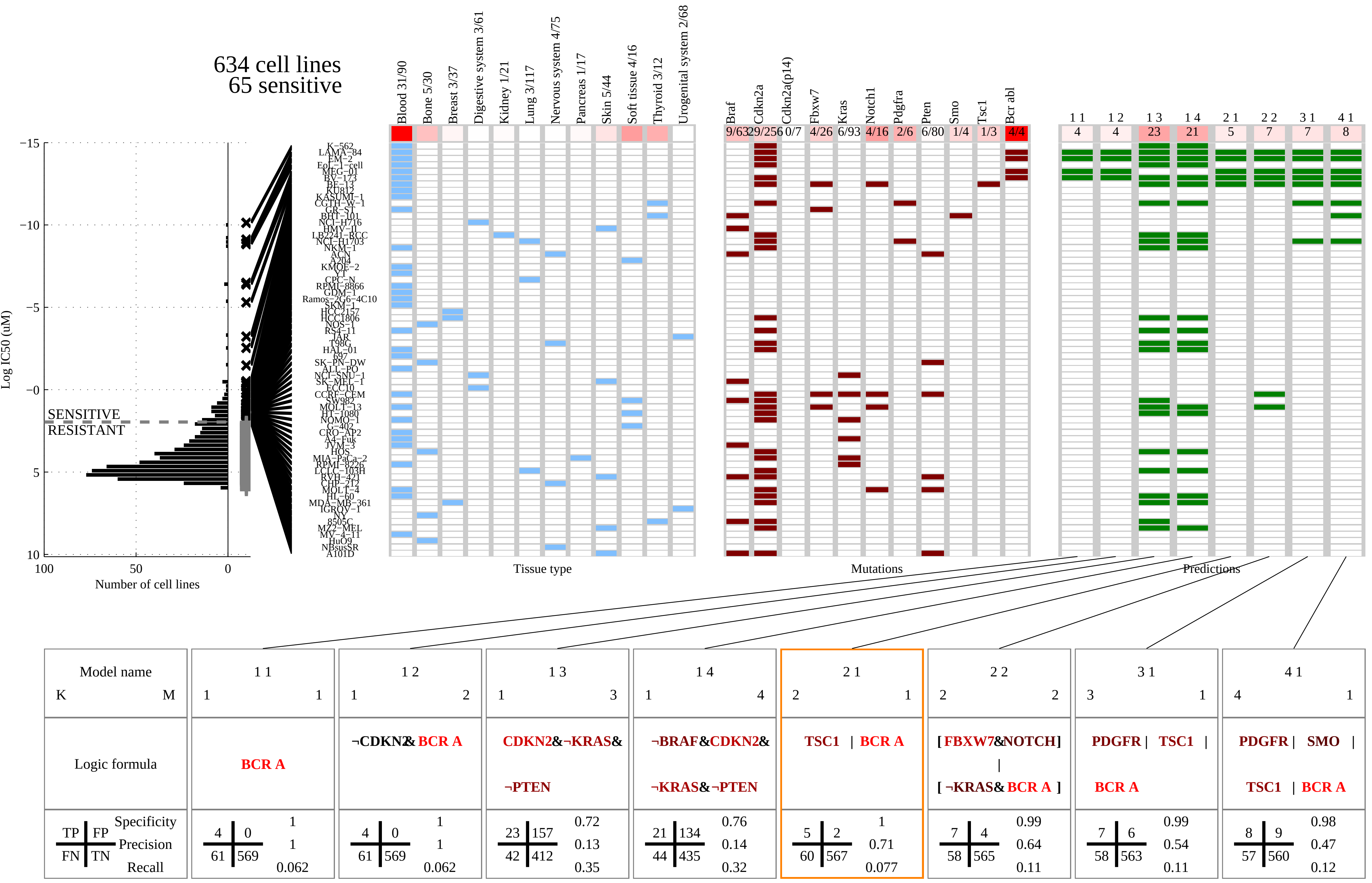

ID:1014 RDEA119 −> MAP2K1 (MEK1), MAP2K2 (MEK2)

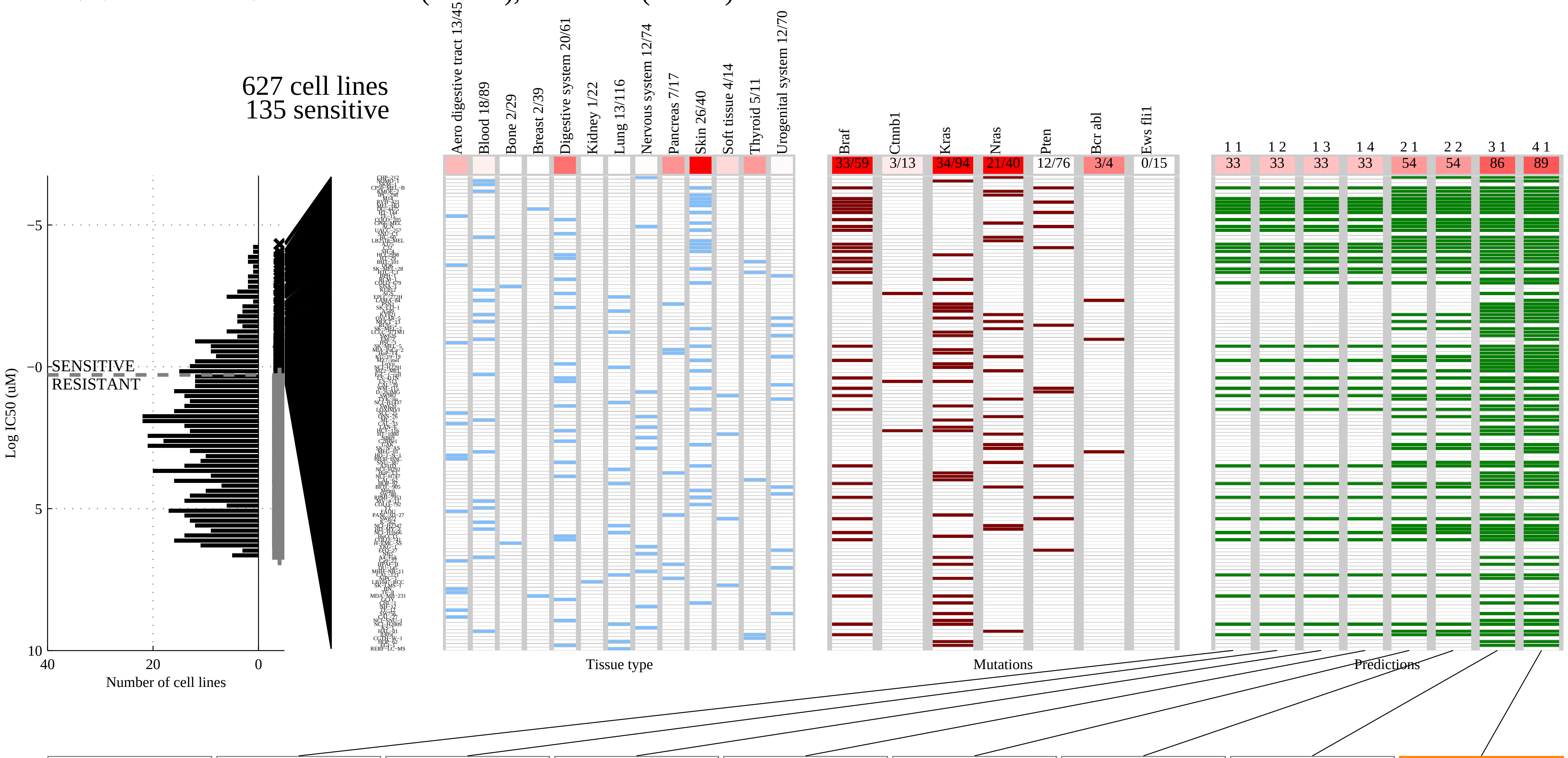

Model name (K, M)	1 1 (1, 1)	1 2 (1, 2)	1 3 (1, 3)	1 4 (1, 4)	2 1 (2, 1)	2 2 (2, 2)	3 1 (3, 1)	4 1 (4, 1)
Logic formula	**BRAF**	**BRAF** &¬**NRAS**	**BRAF** &¬CTNNB& ¬NRAS	**BRAF** &¬CTNNB& ¬NRAS&¬EWS F	**BRAF** | **NRAS**	[**NRAS** &¬PTEN] | [**BRAF** &CTNNB]	**BRAF** | **KRAS** | **NRAS**	**BRAF** | **KRAS** | **NRAS** | **BCR A**
TP | FP / FN | TN	33 | 26 / 102 | 466	33 | 24 / 102 | 468	33 | 23 / 102 | 469	33 | 22 / 102 | 470	54 | 43 / 81 | 449	54 | 38 / 81 | 454	86 | 102 / 49 | 390	89 | 103 / 46 | 389
Specificity	0.95	0.95	0.95	0.96	0.91	0.92	0.79	0.79
Precision	0.56	0.58	0.59	0.6	0.56	0.59	0.46	0.46
Recall	0.24	0.24	0.24	0.24	0.4	0.4	0.64	0.66

ID:1015 CI−1040 −> MAP2K1 (MEK1), MAP2K2 (MEK2)

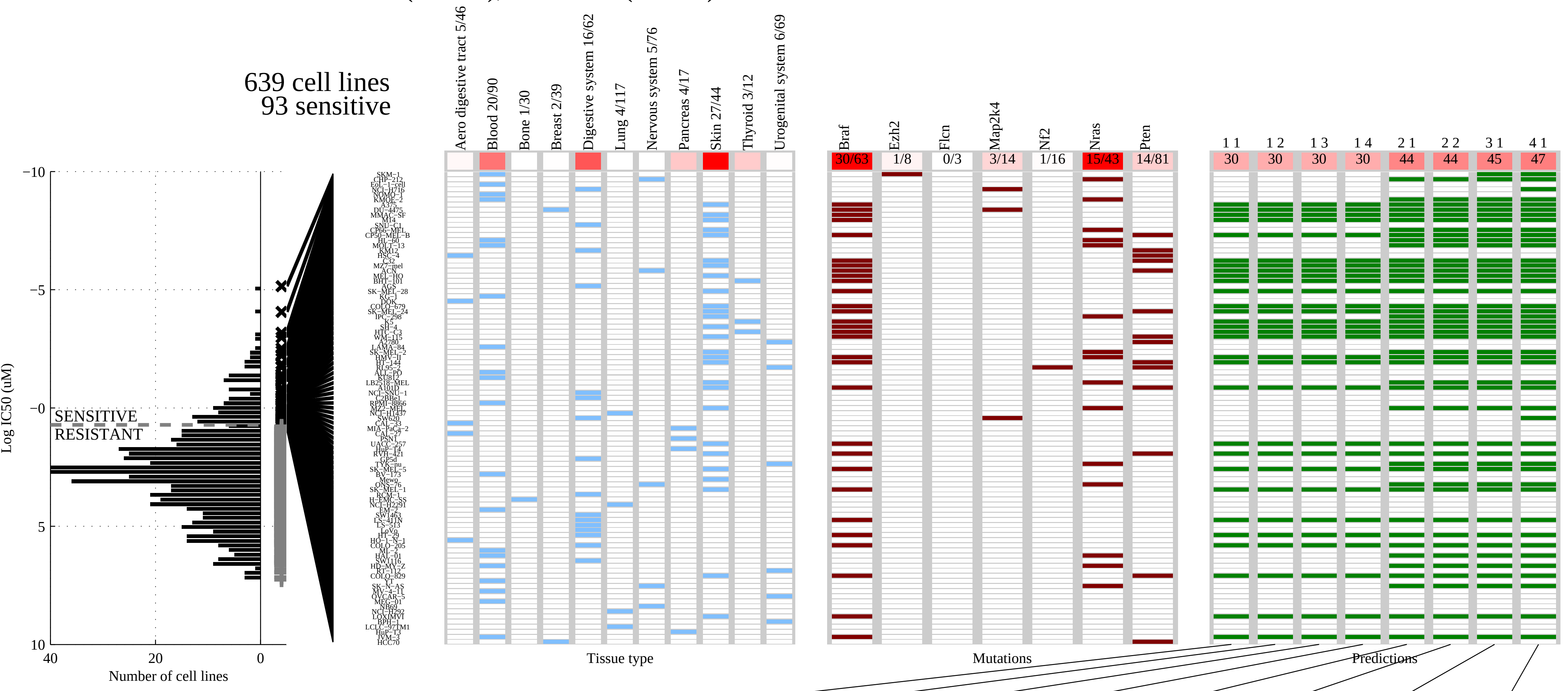

<table>
<tr><td>Model name
K M</td><td>1 1
1 1</td><td>1 2
1 2</td><td>1 3
1 3</td><td>1 4
1 4</td><td>2 1
2 1</td><td>2 2
2 2</td><td>3 1
3 1</td><td>4 1
4 1</td></tr>
<tr><td>Logic formula</td><td>BRAF</td><td>BRAF &¬EZH2</td><td>BRAF &¬EZH2& ¬FLCN</td><td>BRAF &¬EZH2& ¬FLCN& ¬NF2</td><td>BRAF | NRAS</td><td>[NRAS &¬PTEN] | [BRAF &¬FLCN]</td><td>BRAF | EZH2 | NRAS</td><td>BRAF | EZH2 | MAP2K | NRAS</td></tr>
<tr><td>TP FP / FN TN
Specificity
Precision
Recall</td><td>30 33 / 63 513
0.94
0.48
0.32</td><td>30 29 / 63 517
0.95
0.51
0.32</td><td>30 28 / 63 518
0.95
0.52
0.32</td><td>30 25 / 63 521
0.95
0.55
0.32</td><td>44 59 / 49 487
0.89
0.43
0.47</td><td>44 54 / 49 492
0.9
0.45
0.47</td><td>45 62 / 48 484
0.89
0.42
0.48</td><td>47 71 / 46 475
0.87
0.4
0.51</td></tr>
</table>

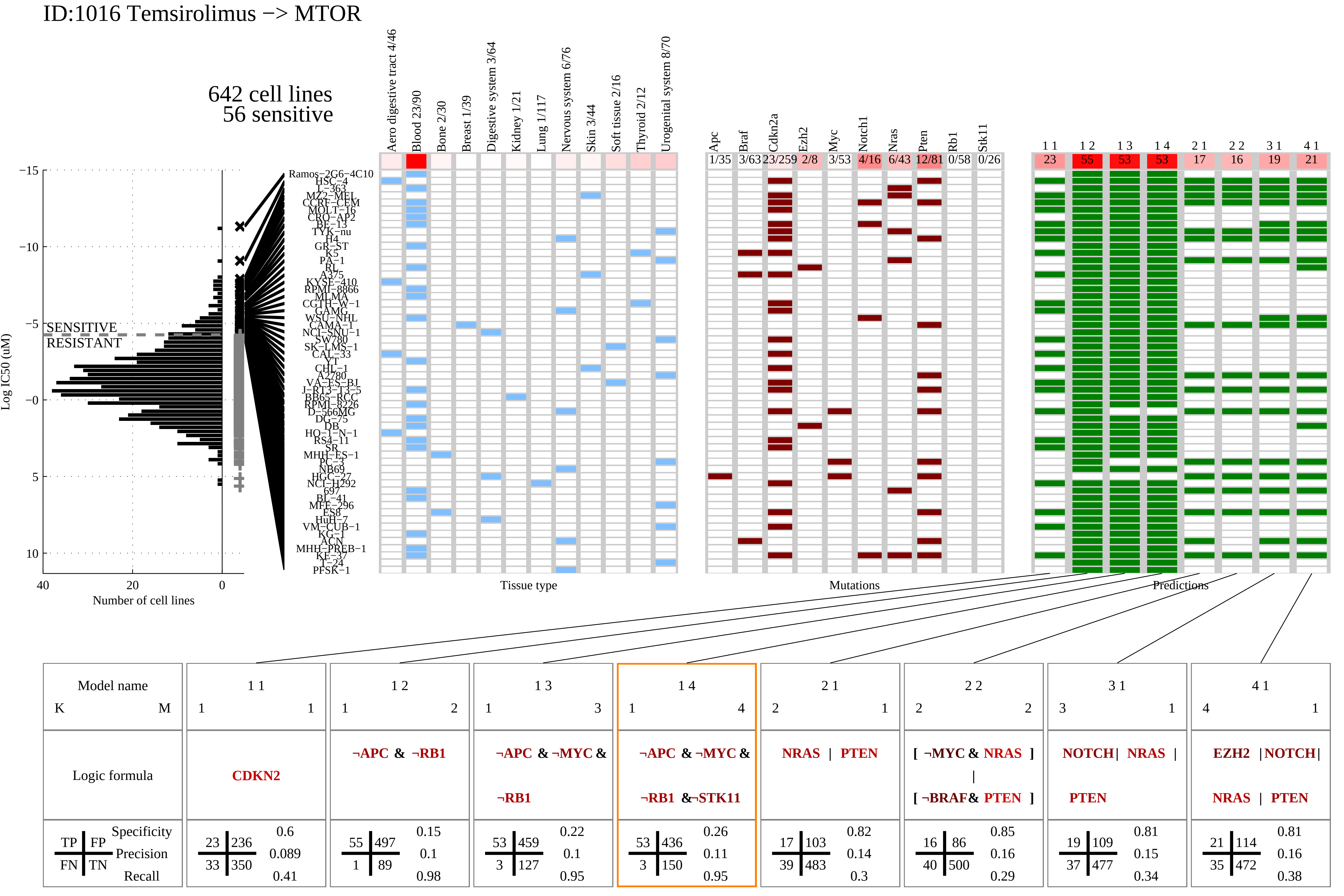

ID:1016 Temsirolimus −> MTOR
642 cell lines
56 sensitive
Aero digestive tract 4/46
Blood 23/90
Bone 2/30
Breast 1/39
Digestive system 3/64
Kidney 1/21
Lung 1/117
Nervous system 6/76
Skin 3/44
Soft tissue 2/16
Thyroid 2/12
Urogenital system 8/70
Apc 1/35
Braf 3/63
Cdkn2a 23/259
Ezh2 2/8
Myc 3/53
Notch1 4/16
Nras 6/43
Pten 12/81
Rb1 0/58
Stk11 0/26
1 1: 23
1 2: 55
1 3: 53
1 4: 53
2 1: 17
2 2: 16
3 1: 19
4 1: 21
SENSITIVE
RESISTANT
Log IC50 (uM)
Number of cell lines
Tissue type
Mutations
Predictions
Model name K M
Logic formula
TP FP FN TN
Specificity Precision Recall
1 1 (K 1, M 1): CDKN2 — TP 23, FP 236, FN 33, TN 350; 0.6, 0.089, 0.41
1 2 (K 1, M 2): ¬APC & ¬RB1 — TP 55, FP 497, FN 1, TN 89; 0.15, 0.1, 0.98
1 3 (K 1, M 3): ¬APC & ¬MYC & ¬RB1 — TP 53, FP 459, FN 3, TN 127; 0.22, 0.1, 0.95
1 4 (K 1, M 4): ¬APC & ¬MYC & ¬RB1 & ¬STK11 — TP 53, FP 436, FN 3, TN 150; 0.26, 0.11, 0.95
2 1 (K 2, M 1): NRAS | PTEN — TP 17, FP 103, FN 39, TN 483; 0.82, 0.14, 0.3
2 2 (K 2, M 2): [¬MYC & NRAS] | [¬BRAF & PTEN] — TP 16, FP 86, FN 40, TN 500; 0.85, 0.16, 0.29
3 1 (K 3, M 1): NOTCH | NRAS | PTEN — TP 19, FP 109, FN 37, TN 477; 0.81, 0.15, 0.34
4 1 (K 4, M 1): EZH2 | NOTCH | NRAS | PTEN — TP 21, FP 114, FN 35, TN 472; 0.81, 0.16, 0.38

ID:1017 Olaparib –> PARP1, PARP2

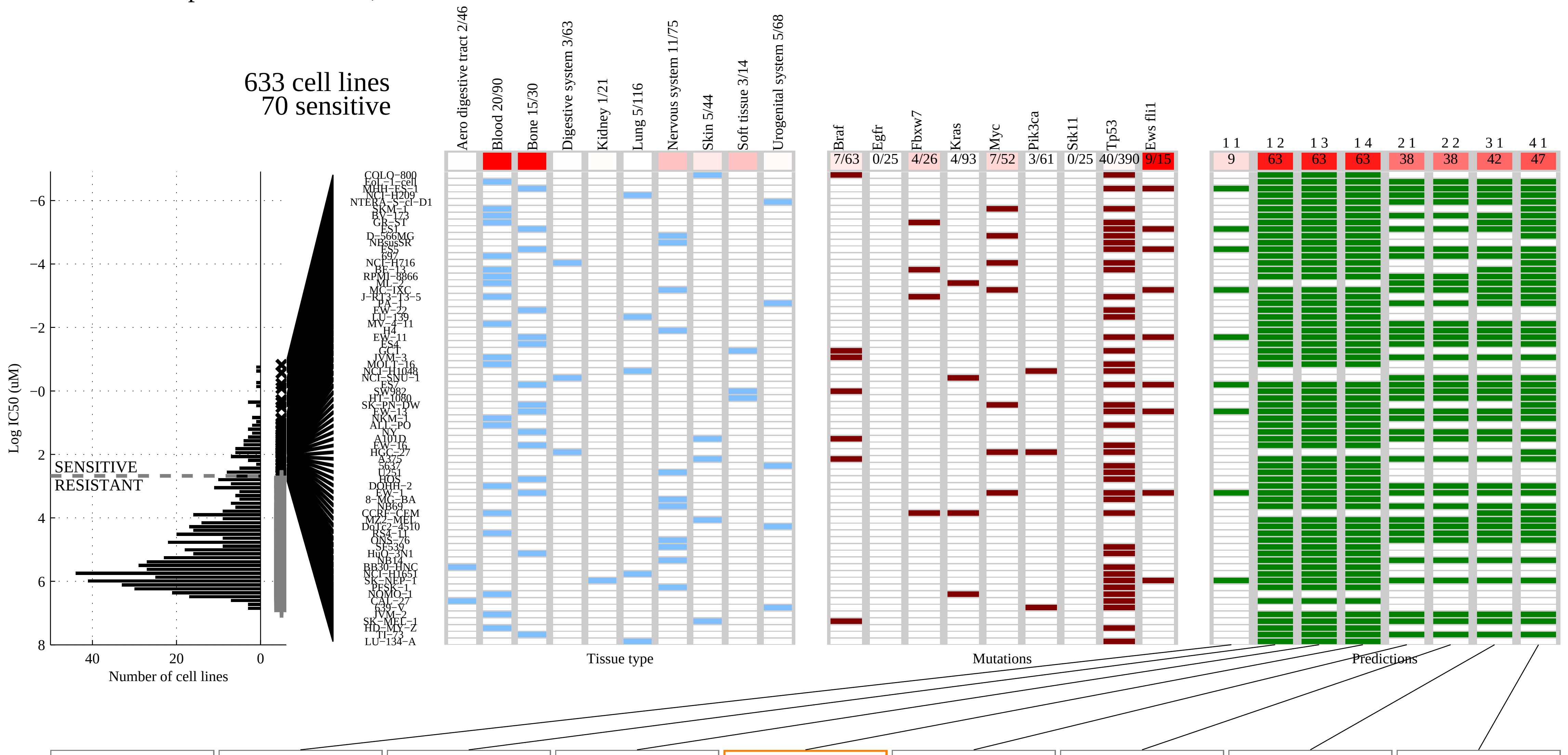

Model name	1 1	1 2	1 3	1 4	2 1	2 2	3 1	4 1
K / M	1 / 1	1 / 2	1 / 3	1 / 4	2 / 1	2 / 2	3 / 1	4 / 1
Logic formula	**EWS F**	**¬KRAS&¬PIK3C**	**¬KRAS&¬PIK3C& ¬STK11**	**¬EGFR&¬KRAS& ¬PIK3C&¬STK11**	**¬TP53 | EWS F**	**[¬BRAF& EWS F] | [¬PIK3C& ¬TP53]**	**FBXW7 | ¬TP53 | EWS F**	**FBXW7 | MYC | ¬TP53 | EWS F**
TP FP / FN TN	9 6 / 61 557	63 428 / 7 135	63 411 / 7 152	63 394 / 7 169	38 217 / 32 346	38 193 / 32 370	42 234 / 28 329	47 268 / 23 295
Specificity	0.99	0.24	0.27	0.3	0.61	0.66	0.58	0.52
Precision	0.6	0.13	0.13	0.14	0.15	0.16	0.15	0.15
Recall	0.13	0.9	0.9	0.9	0.54	0.54	0.6	0.67

ID:1018 ABT−888 −> PARP1, PARP2

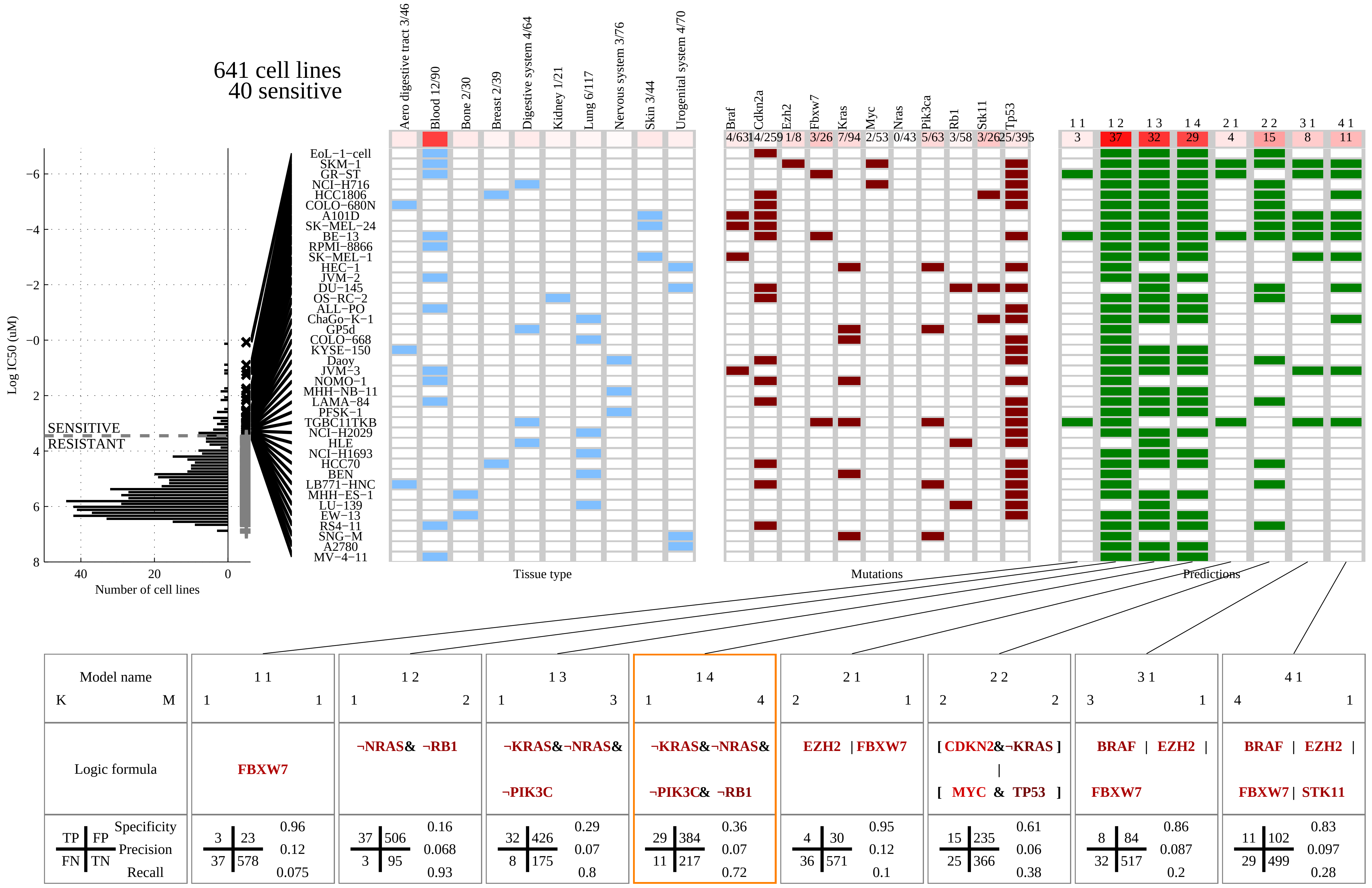

ID:1019 Bosutinib –> SRC, ABL, TEC

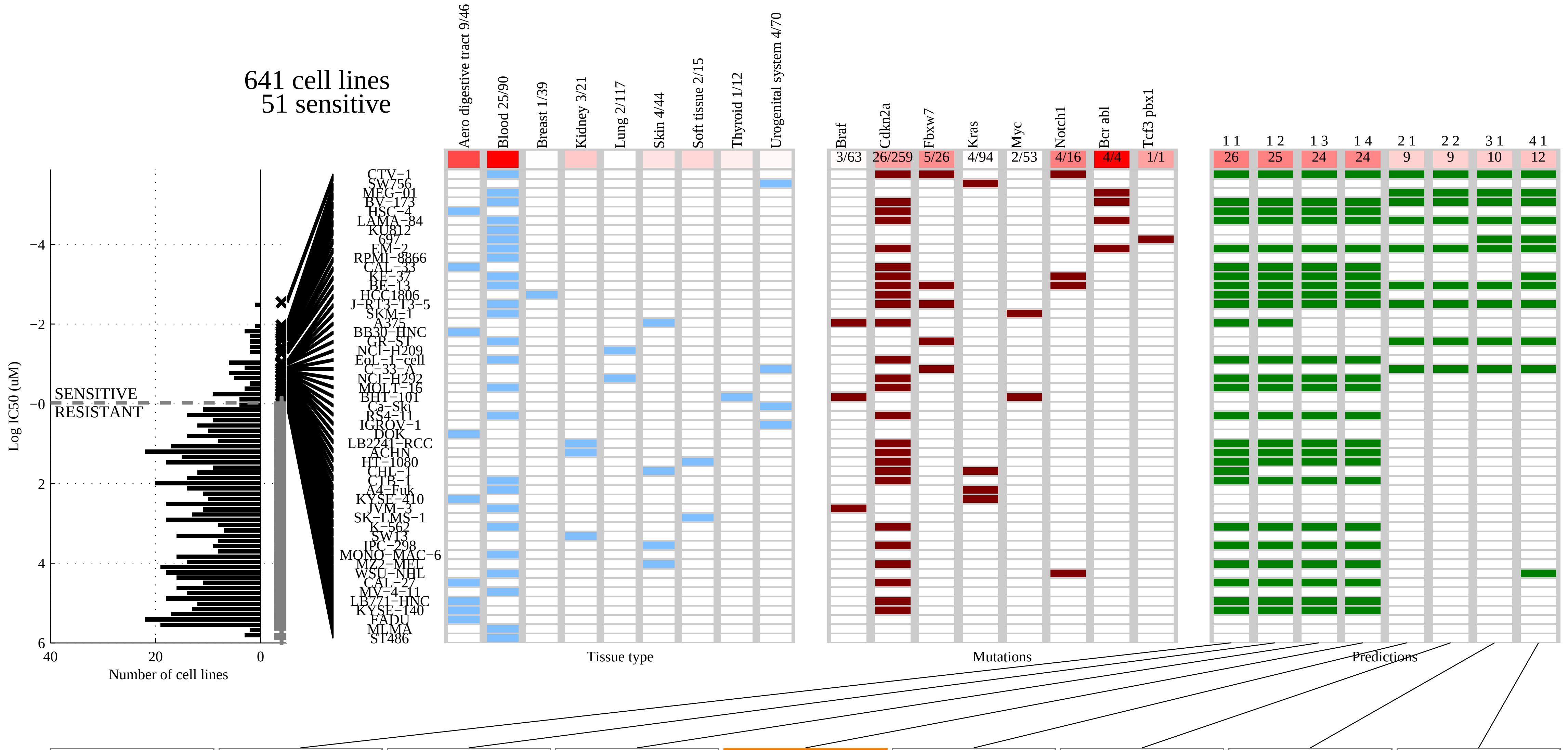

Model name	1 1	1 2	1 3	1 4	2 1	2 2	3 1	4 1
K / M	1 / 1	1 / 2	1 / 3	1 / 4	2 / 1	2 / 2	3 / 1	4 / 1
Logic formula	CDKN2	CDKN2&¬KRAS	¬BRAF&CDKN2&¬KRAS	¬BRAF&CDKN2&¬KRAS&¬MYC	FBXW7 | BCR A	[BCR A&] | [FBXW7&¬KRAS]	FBXW7 | BCR A | TCF3	FBXW7 | NOTCH | BCR A | TCF3
TP / FP	26 / 233	25 / 195	24 / 158	24 / 144	9 / 21	9 / 10	10 / 21	12 / 28
FN / TN	25 / 357	26 / 395	27 / 432	27 / 446	42 / 569	42 / 580	41 / 569	39 / 562
Specificity	0.61	0.67	0.73	0.76	0.96	0.98	0.96	0.95
Precision	0.1	0.11	0.13	0.14	0.3	0.47	0.32	0.3
Recall	0.51	0.49	0.47	0.47	0.18	0.18	0.2	0.24

ID:1020 Lenalidomide −> TNFA

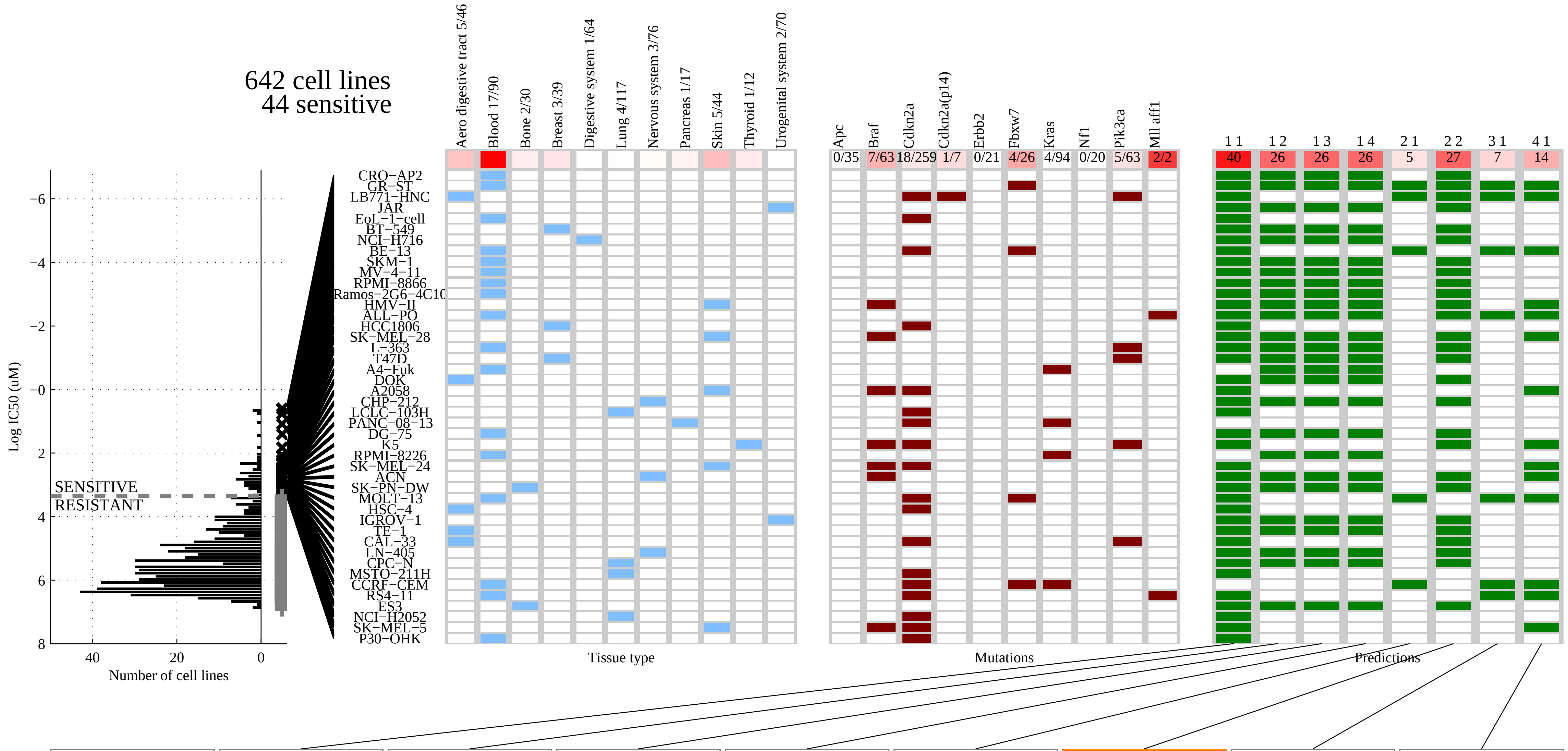

Model name<br>K M	1 1<br>1 1	1 2<br>1 2	1 3<br>1 3	1 4<br>1 4	2 1<br>2 1	2 2<br>2 2	3 1<br>3 1	4 1<br>4 1
Logic formula	¬KRAS	¬APC &¬CDKN2	¬APC &¬CDKN2&<br>¬NF1	¬APC &¬CDKN2&<br>¬ERBB2& ¬NF1	CDKN2 | FBXW7	[¬KRAS& PIK3C]<br>| [¬CDKN2&¬KRAS]	CDKN2 | FBXW7 | MLL A	BRAF | CDKN2 | FBXW7 | MLL A
TP FP / FN TN	40 508 / 4 90	26 324 / 18 274	26 310 / 18 288	26 295 / 18 303	5 27 / 39 571	27 316 / 17 282	7 27 / 37 571	14 81 / 30 517
Specificity	0.15	0.46	0.48	0.51	0.95	0.47	0.95	0.86
Precision	0.073	0.074	0.077	0.081	0.16	0.079	0.21	0.15
Recall	0.91	0.59	0.59	0.59	0.11	0.61	0.16	0.32

ID:1021 Axitinib −> PDGFR, KIT, VEGFR

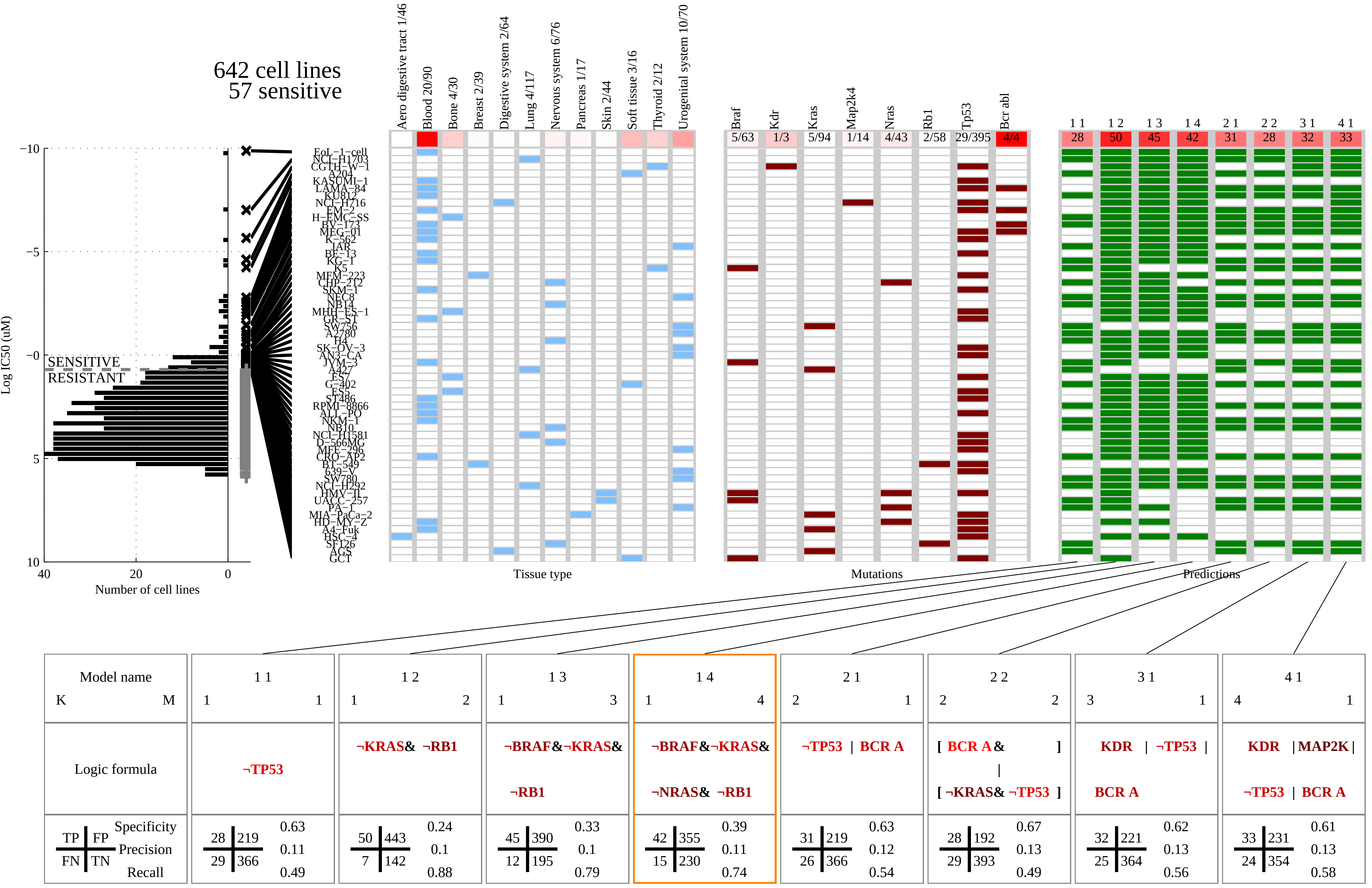

ID:1022 AZD7762 –> CHEK1, CHEK2

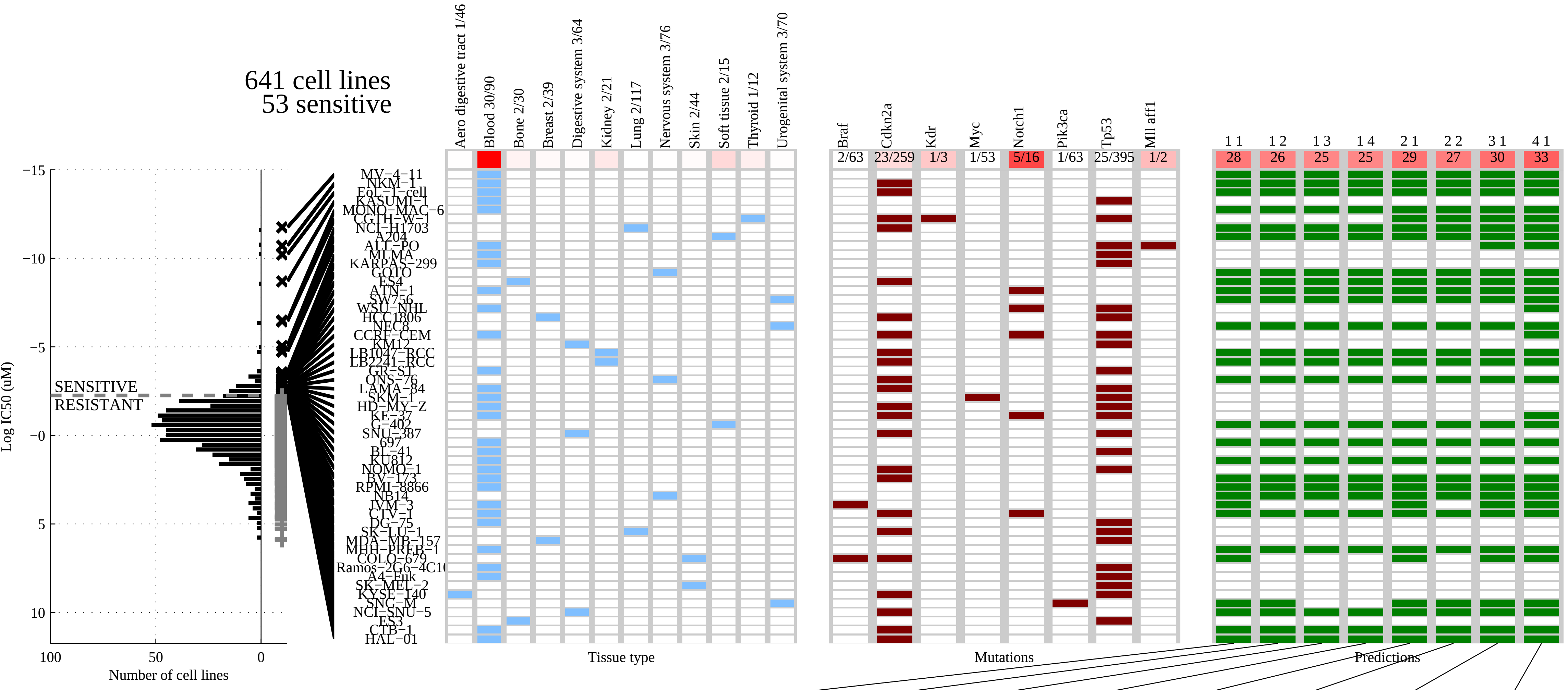

<table>
<tr><td>Model name
K M</td><td>1 1
1 1</td><td>1 2
1 2</td><td>1 3
1 3</td><td>1 4
1 4</td><td>2 1
2 1</td><td>2 2
2 2</td><td>3 1
3 1</td><td>4 1
4 1</td></tr>
<tr><td>Logic formula</td><td>¬TP53</td><td>¬BRAF & ¬TP53</td><td>¬BRAF & ¬PIK3C & ¬TP53</td><td>¬BRAF & ¬MYC & ¬PIK3C & ¬TP53</td><td>KDR | ¬TP53</td><td>[CDKN2 & KDR] | [¬BRAF & ¬TP53]</td><td>KDR | ¬TP53 | MLL A</td><td>KDR | NOTCH | ¬TP53 | MLL A</td></tr>
<tr><td>TP FP / FN TN
Specificity
Precision
Recall</td><td>28 218 / 25 370
0.63
0.11
0.53</td><td>26 188 / 27 400
0.68
0.12
0.49</td><td>25 167 / 28 421
0.72
0.13
0.47</td><td>25 156 / 28 432
0.73
0.14
0.47</td><td>29 220 / 24 368
0.63
0.12
0.55</td><td>27 188 / 26 400
0.68
0.13
0.51</td><td>30 220 / 23 368
0.63
0.12
0.57</td><td>33 229 / 20 359
0.61
0.13
0.62</td></tr>
</table>

ID:1023 GW 441756 –> NTRK1

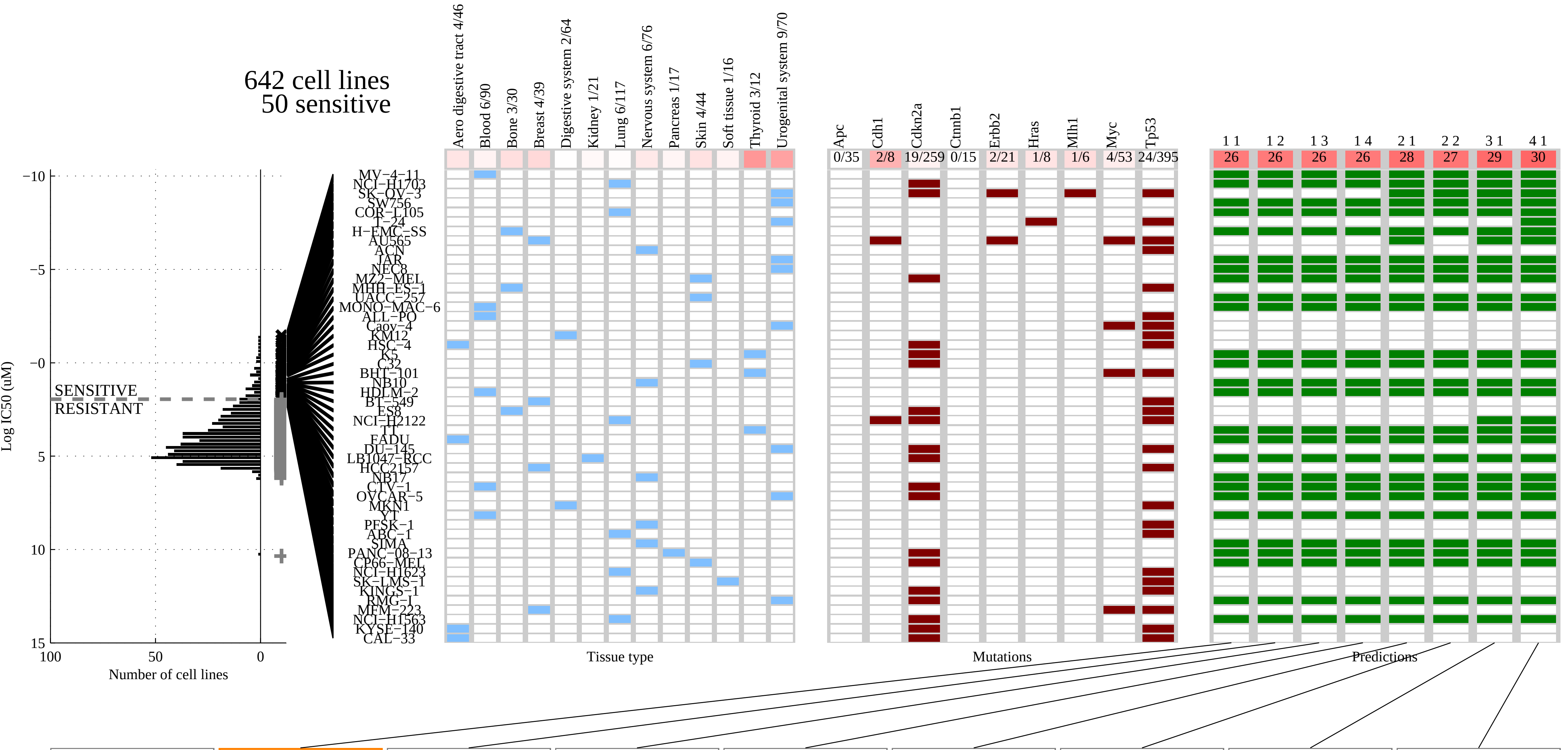

Model name	1 1	1 2	1 3	1 4	2 1	2 2	3 1	4 1
K / M	1 / 1	1 / 2	1 / 3	1 / 4	2 / 1	2 / 2	3 / 1	4 / 1
Logic formula	¬TP53	¬CTNNB& ¬TP53	¬CTNNB& ¬MYC & ¬TP53	¬APC &¬CTNNB& ¬MYC & ¬TP53	ERBB2 | ¬TP53	[CDKN2& MLH1] | [¬CTNNB& ¬TP53]	CDH1 | MLH1 | ¬TP53	CDH1 | HRAS | MLH1 | ¬TP53
TP / FP	26 / 221	26 / 207	26 / 194	26 / 185	28 / 236	27 / 208	29 / 223	30 / 227
FN / TN	24 / 371	24 / 385	24 / 398	24 / 407	22 / 356	23 / 384	21 / 369	20 / 365
Specificity	0.63	0.65	0.67	0.69	0.6	0.65	0.62	0.62
Precision	0.11	0.11	0.12	0.12	0.11	0.11	0.12	0.12
Recall	0.52	0.52	0.52	0.52	0.56	0.54	0.58	0.6

ID:1024 CEP−701 −> FLT3, JAK2, NTRK1, RET

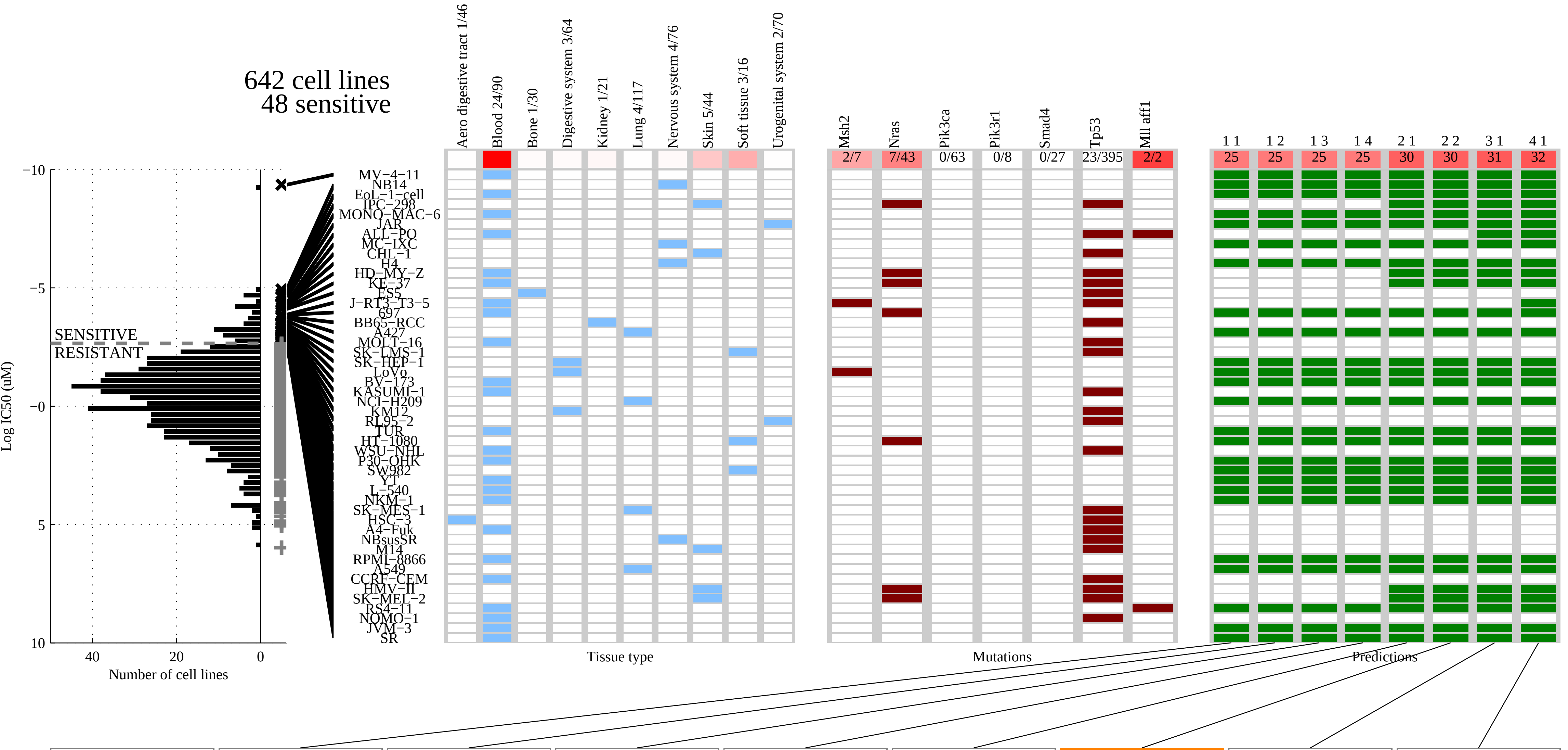

Model name	1 1	1 2	1 3	1 4	2 1	2 2	3 1	4 1
K M	1 1	1 2	1 3	1 4	2 1	2 2	3 1	4 1
Logic formula	¬TP53	¬PIK3C& ¬TP53	¬PIK3C&¬SMAD4& ¬TP53	¬PIK3C&¬PIK3R& ¬SMAD4& ¬TP53	NRAS | ¬TP53	[NRAS &¬PIK3C] | [¬PIK3C& ¬TP53]	NRAS | ¬TP53 | MLL A	MSH2 | NRAS | ¬TP53 | MLL A
TP FP / FN TN	25 222 / 23 372	25 198 / 23 396	25 191 / 23 403	25 187 / 23 407	30 239 / 18 355	30 214 / 18 380	31 239 / 17 355	32 241 / 16 353
Specificity	0.63	0.67	0.68	0.69	0.6	0.64	0.6	0.59
Precision	0.1	0.11	0.12	0.12	0.11	0.12	0.11	0.12
Recall	0.52	0.52	0.52	0.52	0.63	0.63	0.65	0.67

ID:1025 SB 216763 −> GSK3A, GSK3B

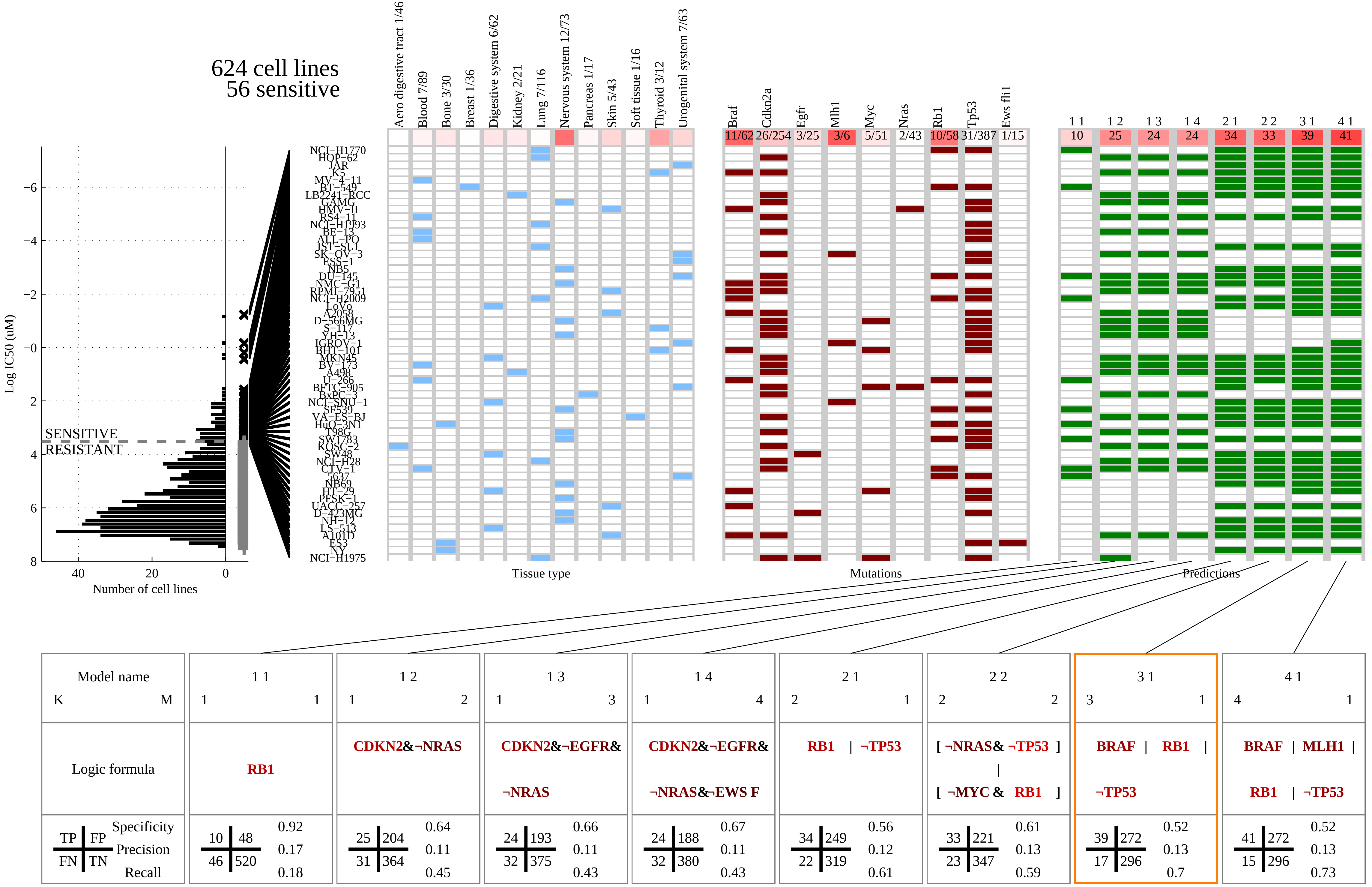

ID:1026 17−AAG −> HSP90

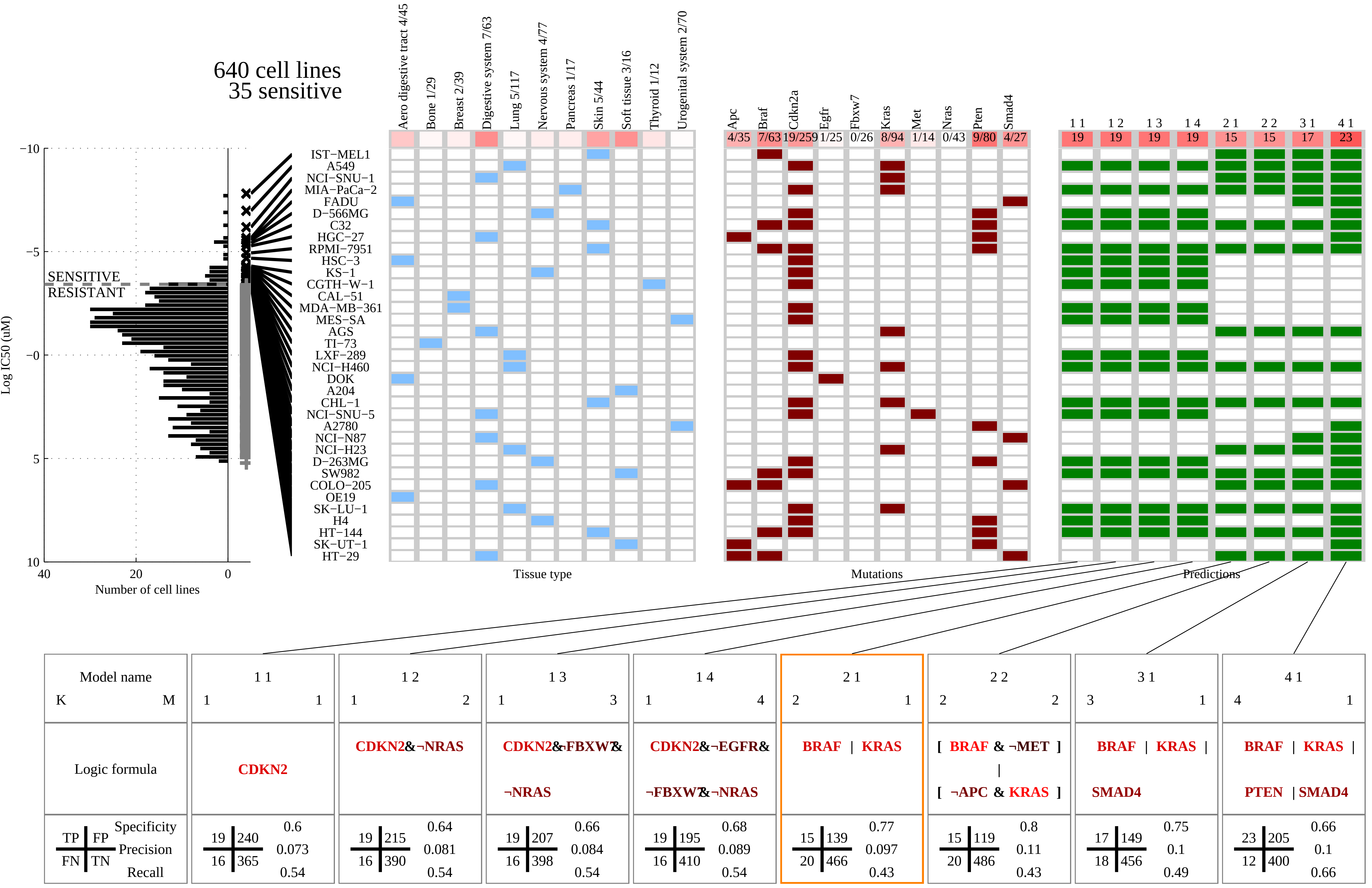

ID:1028 VX−702 -> p38

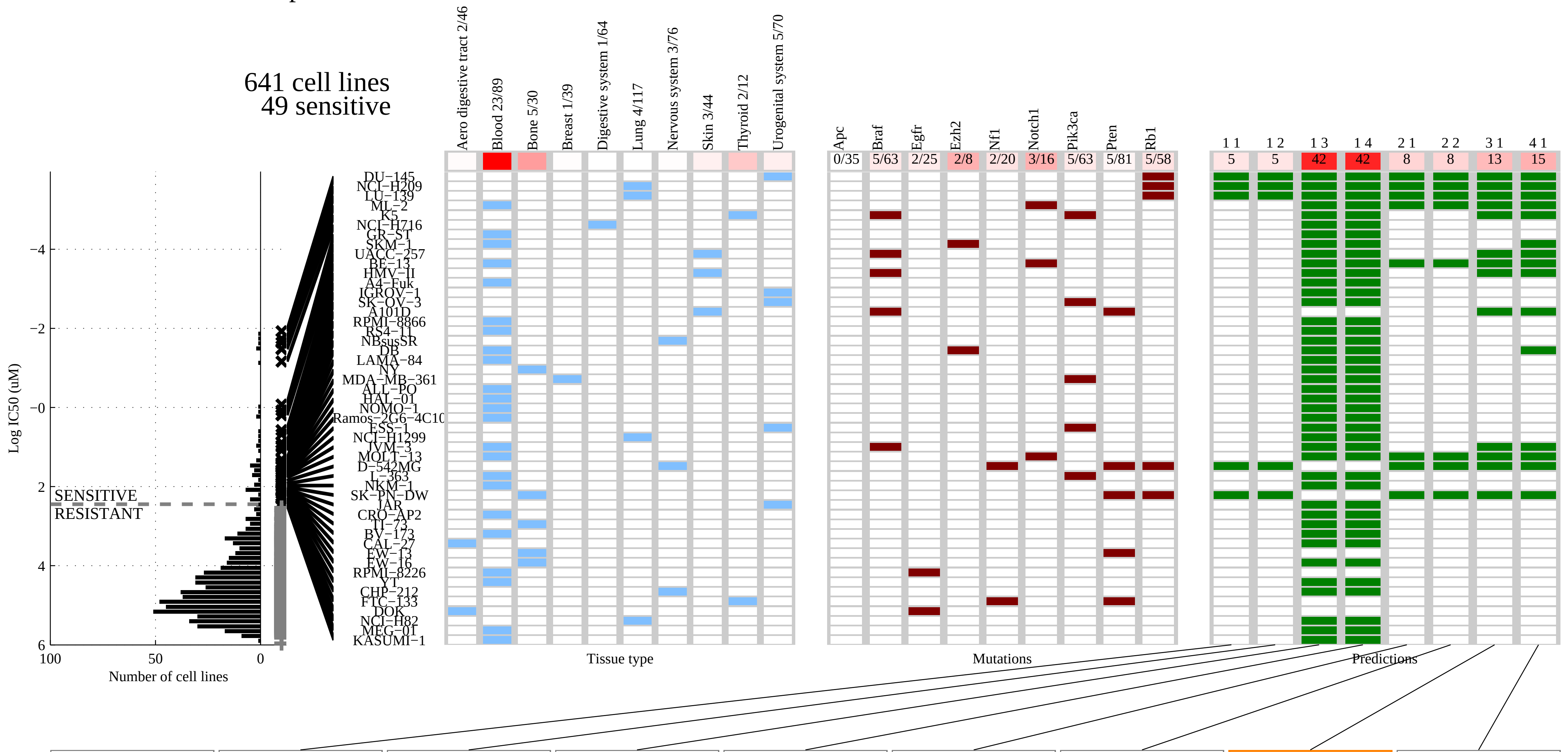

Model name	1 1	1 2	1 3	1 4	2 1	2 2	3 1	4 1
K M	1 1	1 2	1 3	1 4	2 1	2 2	3 1	4 1
Logic formula	RB1	¬PIK3C& RB1	¬APC &¬EGFR& ¬PTEN	¬APC &¬EGFR& ¬NF1 &¬PTEN	NOTCH| RB1	[¬PIK3C& RB1] | [NOTCH&¬PTEN]	BRAF |NOTCH| RB1	BRAF | EZH2 | NOTCH| RB1
TP	5	5	42	42	8	8	13	15
FP	53	45	461	448	65	51	119	121
FN	44	44	7	7	41	41	36	34
TN	539	547	131	144	527	541	473	471
Specificity	0.91	0.92	0.22	0.24	0.89	0.91	0.8	0.8
Precision	0.086	0.1	0.083	0.086	0.11	0.14	0.098	0.11
Recall	0.1	0.1	0.86	0.86	0.16	0.16	0.27	0.31

ID:1029 AMG−706 −> VEGFR, RET, c−KIT, PDGFR

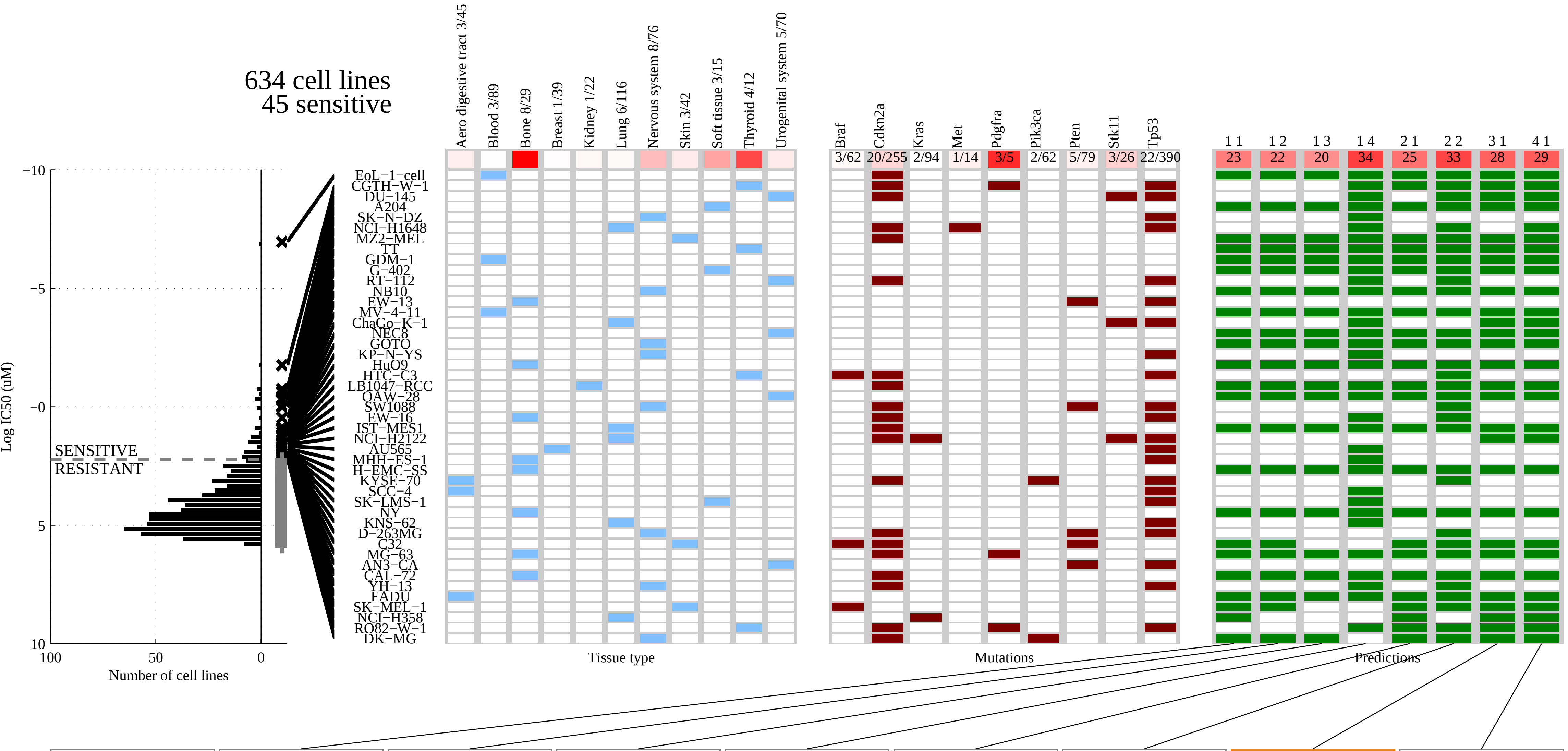

<table>
<tr><td>Model name
K M</td><td>1 1
1 1</td><td>1 2
1 2</td><td>1 3
1 3</td><td>1 4
1 4</td><td>2 1
2 1</td><td>2 2
2 2</td><td>3 1
3 1</td><td>4 1
4 1</td></tr>
<tr><td>Logic formula</td><td>¬TP53</td><td>¬KRAS& ¬TP53</td><td>¬BRAF&¬KRAS&
¬TP53</td><td>¬BRAF&¬KRAS&
¬PIK3C&¬PTEN</td><td>PDGFR | ¬TP53</td><td>[¬KRAS& ¬TP53]
|
[CDKN2&¬KRAS]</td><td>PDGFR | STK11 |
¬TP53</td><td>MET | PDGFR |
STK11 | ¬TP53</td></tr>
<tr><td>TP FP / FN TN</td><td>23 221 / 22 368</td><td>22 192 / 23 397</td><td>20 163 / 25 426</td><td>34 344 / 11 245</td><td>25 223 / 20 366</td><td>33 305 / 12 284</td><td>28 237 / 17 352</td><td>29 246 / 16 343</td></tr>
<tr><td>Specificity</td><td>0.62</td><td>0.67</td><td>0.72</td><td>0.42</td><td>0.62</td><td>0.48</td><td>0.6</td><td>0.58</td></tr>
<tr><td>Precision</td><td>0.094</td><td>0.1</td><td>0.11</td><td>0.09</td><td>0.1</td><td>0.098</td><td>0.11</td><td>0.11</td></tr>
<tr><td>Recall</td><td>0.51</td><td>0.49</td><td>0.44</td><td>0.76</td><td>0.56</td><td>0.73</td><td>0.62</td><td>0.64</td></tr>
</table>

ID:1030 KU−55933 −> ATM

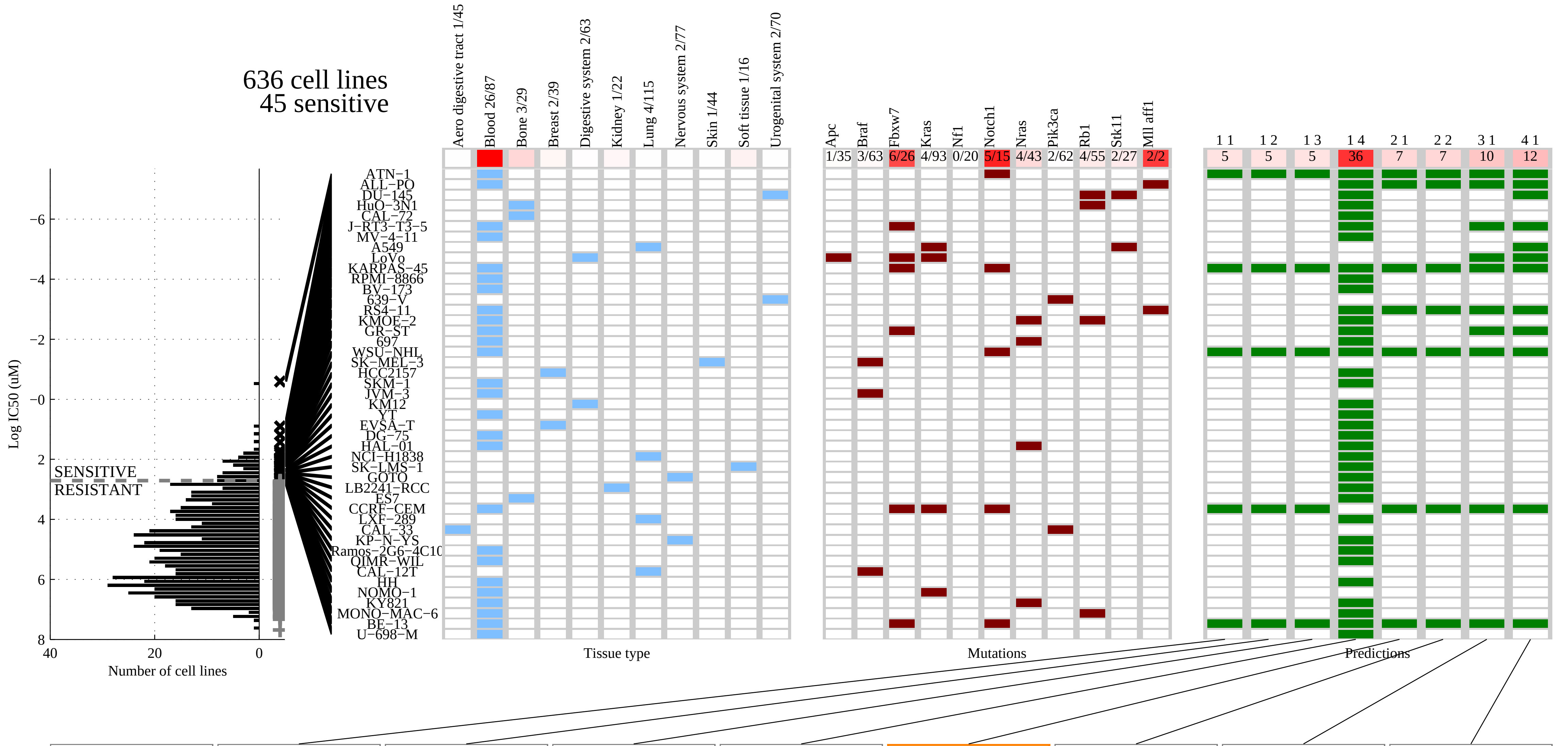

Model name	1 1	1 2	1 3	1 4	2 1	2 2	3 1	4 1
K / M	1 / 1	1 / 2	1 / 3	1 / 4	2 / 1	2 / 2	3 / 1	4 / 1
Logic formula	NOTCH	NOTCH&¬NRAS	¬APC &NOTCH& ¬NRAS	¬BRAF&¬KRAS& ¬NF1 &¬PIK3C	NOTCH | MLL A	[¬RB1 &MLL A] | [NOTCH&¬NRAS]	FBXW7 | NOTCH | MLL A	FBXW7 | NOTCH | STK11 | MLL A
TP	5	5	5	36	7	7	10	12
FP	10	5	4	384	10	5	26	51
FN	40	40	40	9	38	38	35	33
TN	581	586	587	207	581	586	565	540
Specificity	0.98	0.99	0.99	0.35	0.98	0.99	0.96	0.91
Precision	0.33	0.5	0.56	0.086	0.41	0.58	0.28	0.19
Recall	0.11	0.11	0.11	0.8	0.16	0.16	0.22	0.27

ID:1031 Elesclomol -> HSP70

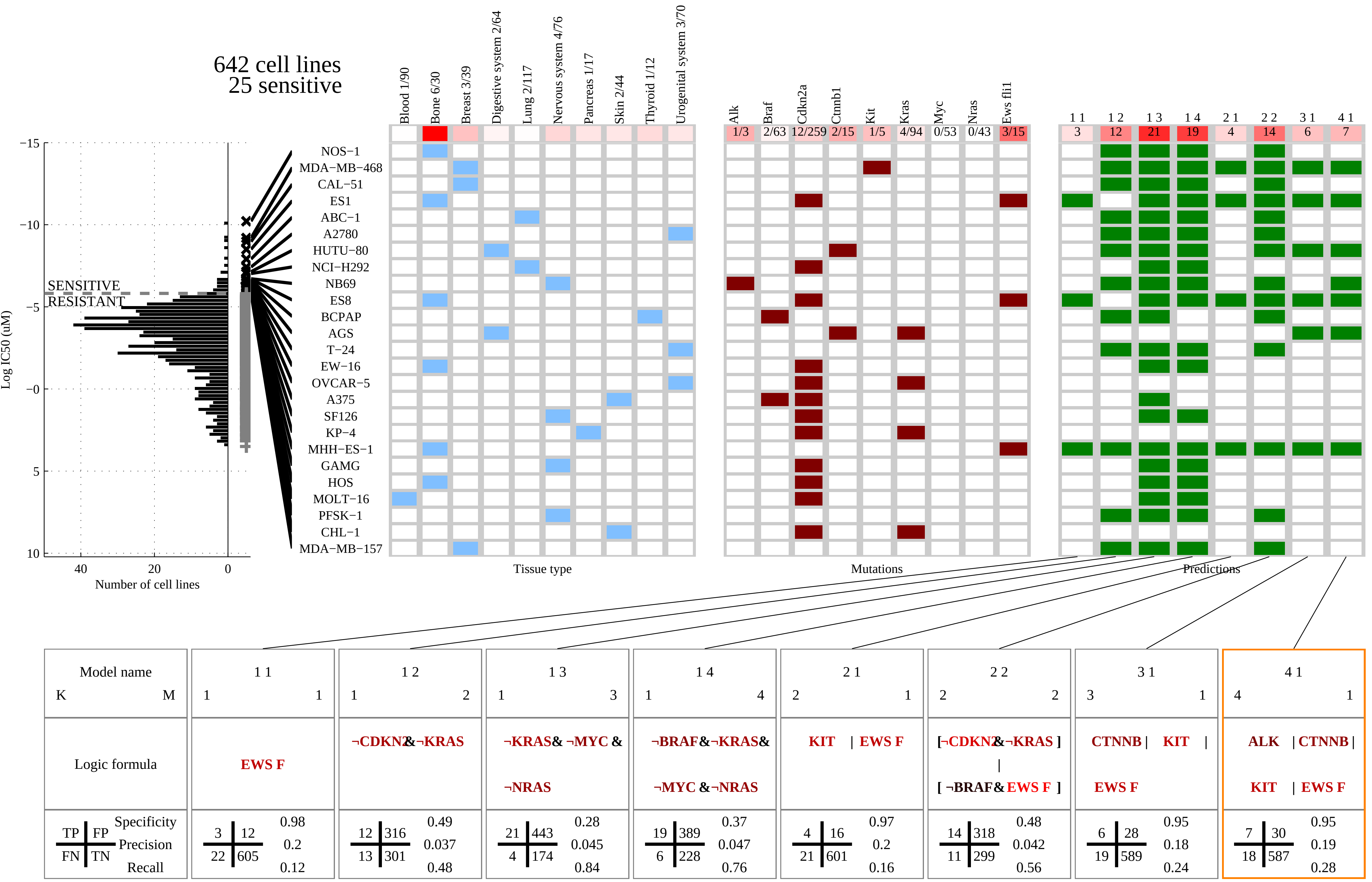

ID:1032 Afatinib −> ERBB2, EGFR

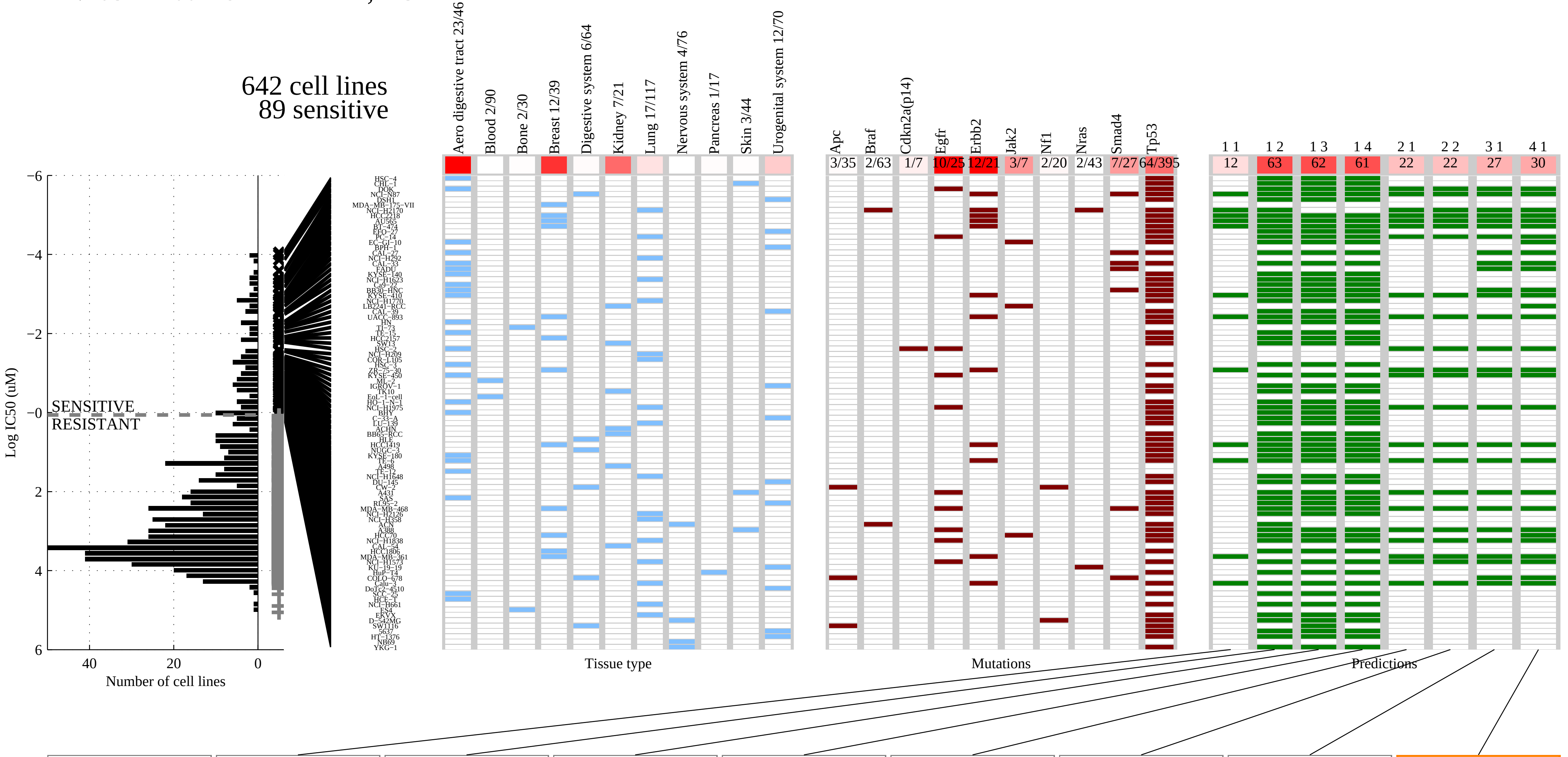

Model name (K, M)	1 1 (1, 1)	1 2 (1, 2)	1 3 (1, 3)	1 4 (1, 4)	2 1 (2, 1)	2 2 (2, 2)	3 1 (3, 1)	4 1 (4, 1)
Logic formula	ERBB2	¬APC & TP53	¬BRAF&¬NRAS& TP53	¬APC &¬BRAF& ¬NRAS& TP53	EGFR | ERBB2	[¬CDKN2&ERBB2] | [EGFR & ¬NF1]	EGFR | ERBB2 | SMAD4	EGFR | ERBB2 | JAK2 | SMAD4
TP / FP	12 / 9	63 / 306	62 / 283	61 / 262	22 / 24	22 / 22	27 / 44	30 / 46
FN / TN	77 / 544	26 / 247	27 / 270	28 / 291	67 / 529	67 / 531	62 / 509	59 / 507
Specificity	0.98	0.45	0.49	0.53	0.96	0.96	0.92	0.92
Precision	0.57	0.17	0.18	0.19	0.48	0.5	0.38	0.39
Recall	0.13	0.71	0.7	0.69	0.25	0.25	0.3	0.34

ID:1033 Vismodegib −> SMO

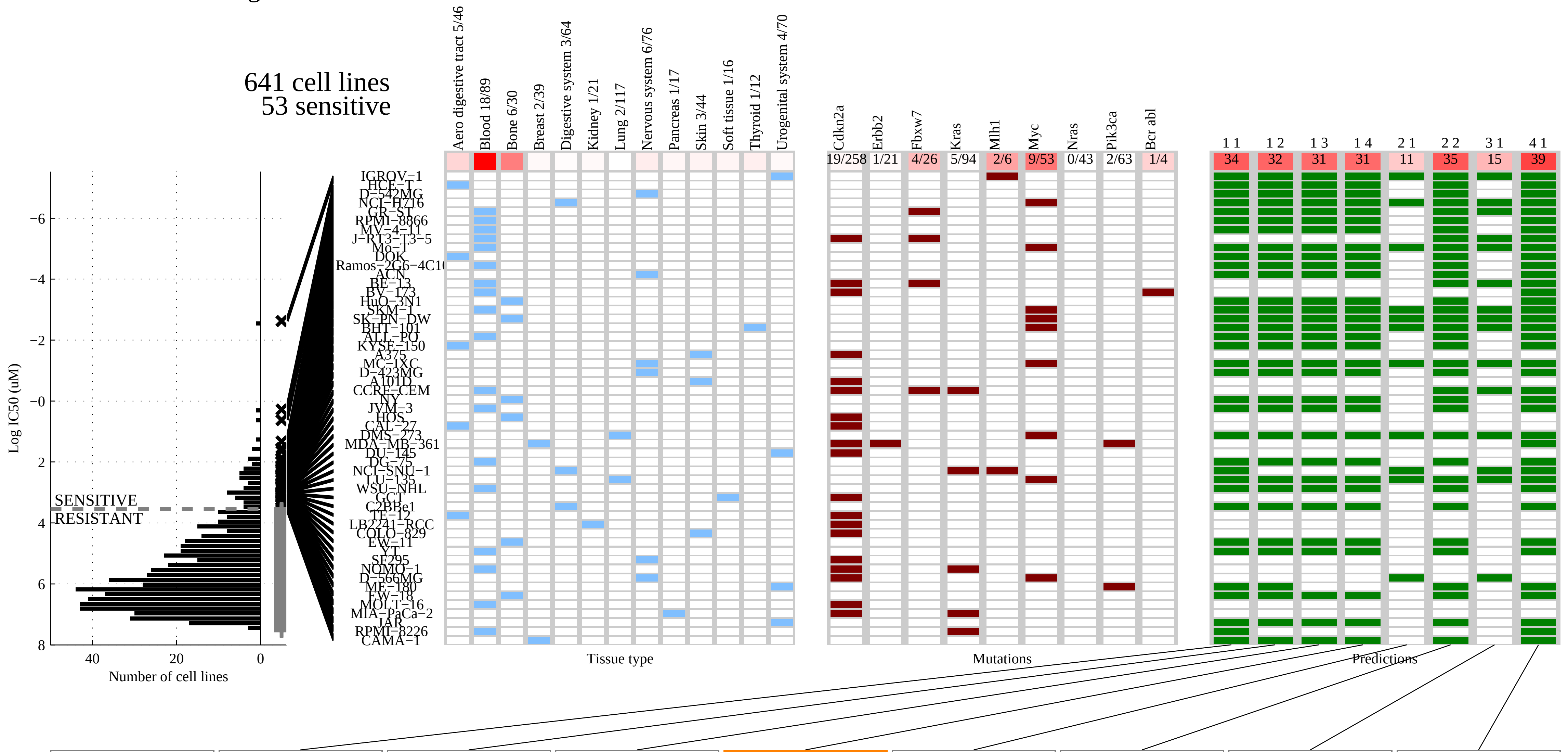

Model name	1 1	1 2	1 3	1 4	2 1	2 2	3 1	4 1
K / M	1 / 1	1 / 2	1 / 3	1 / 4	2 / 1	2 / 2	3 / 1	4 / 1
Logic formula	**¬CDKN2**	**¬CDKN2&¬KRAS**	**¬CDKN2&¬KRAS& ¬PIK3C**	**¬CDKN2&¬KRAS& ¬NRAS&¬PIK3C**	**MLH1 | MYC**	**[CDKN2&FBXW7] | [¬CDKN2&¬KRAS]**	**FBXW7 | MLH1 | MYC**	**¬CDKN2 | ERBB2 | FBXW7 | BCR A**
TP / FP	34 / 349	32 / 296	31 / 262	31 / 246	11 / 48	35 / 304	15 / 70	39 / 363
FN / TN	19 / 239	21 / 292	22 / 326	22 / 342	42 / 540	18 / 284	38 / 518	14 / 225
Specificity	0.41	0.5	0.55	0.58	0.92	0.48	0.88	0.38
Precision	0.089	0.098	0.11	0.11	0.19	0.1	0.18	0.097
Recall	0.64	0.6	0.58	0.58	0.21	0.66	0.28	0.74

ID:1036 PLX4720 –> BRAF

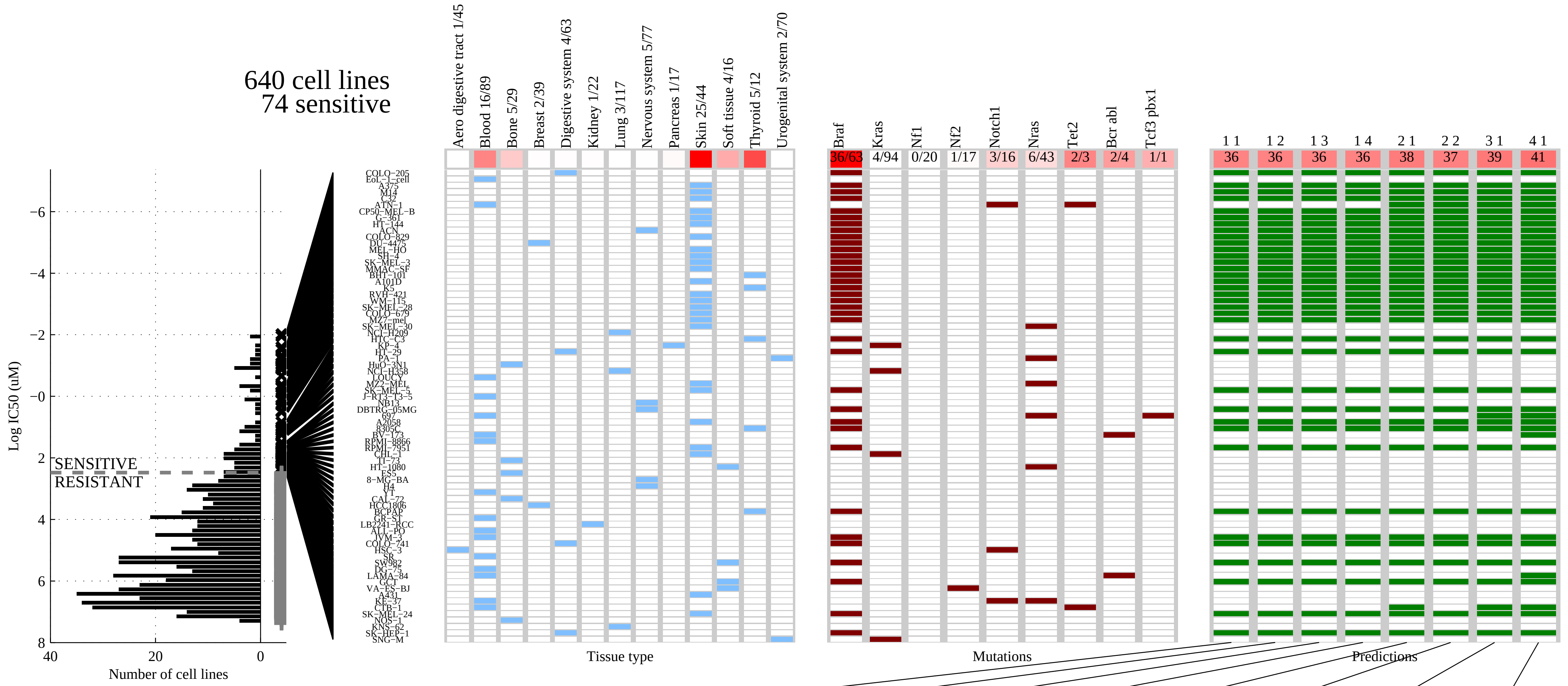

Model name	1 1	1 2	1 3	1 4	2 1	2 2	3 1	4 1
K	1	1	1	1	2	2	3	4
M	1	2	3	4	1	2	1	1
Logic formula	BRAF	BRAF &¬KRAS	BRAF & ¬NF2 & ¬NRAS	BRAF & ¬NF1 & ¬NF2 &¬NRAS	BRAF | TET2	[NOTCH1& TET2] | [BRAF &¬KRAS]	BRAF | TET2 | TCF3	BRAF | TET2 | BCR A | TCF3
TP	36	36	36	36	38	37	39	41
FP	27	24	21	19	28	24	28	30
FN	38	38	38	38	36	37	35	33
TN	539	542	545	547	538	542	538	536
Specificity	0.95	0.96	0.96	0.97	0.95	0.96	0.95	0.95
Precision	0.57	0.6	0.63	0.65	0.58	0.61	0.58	0.58
Recall	0.49	0.49	0.49	0.49	0.51	0.5	0.53	0.55

ID:1037 BX−795 −> TBK1, PDPK1, IKK, AURKB, AURKC

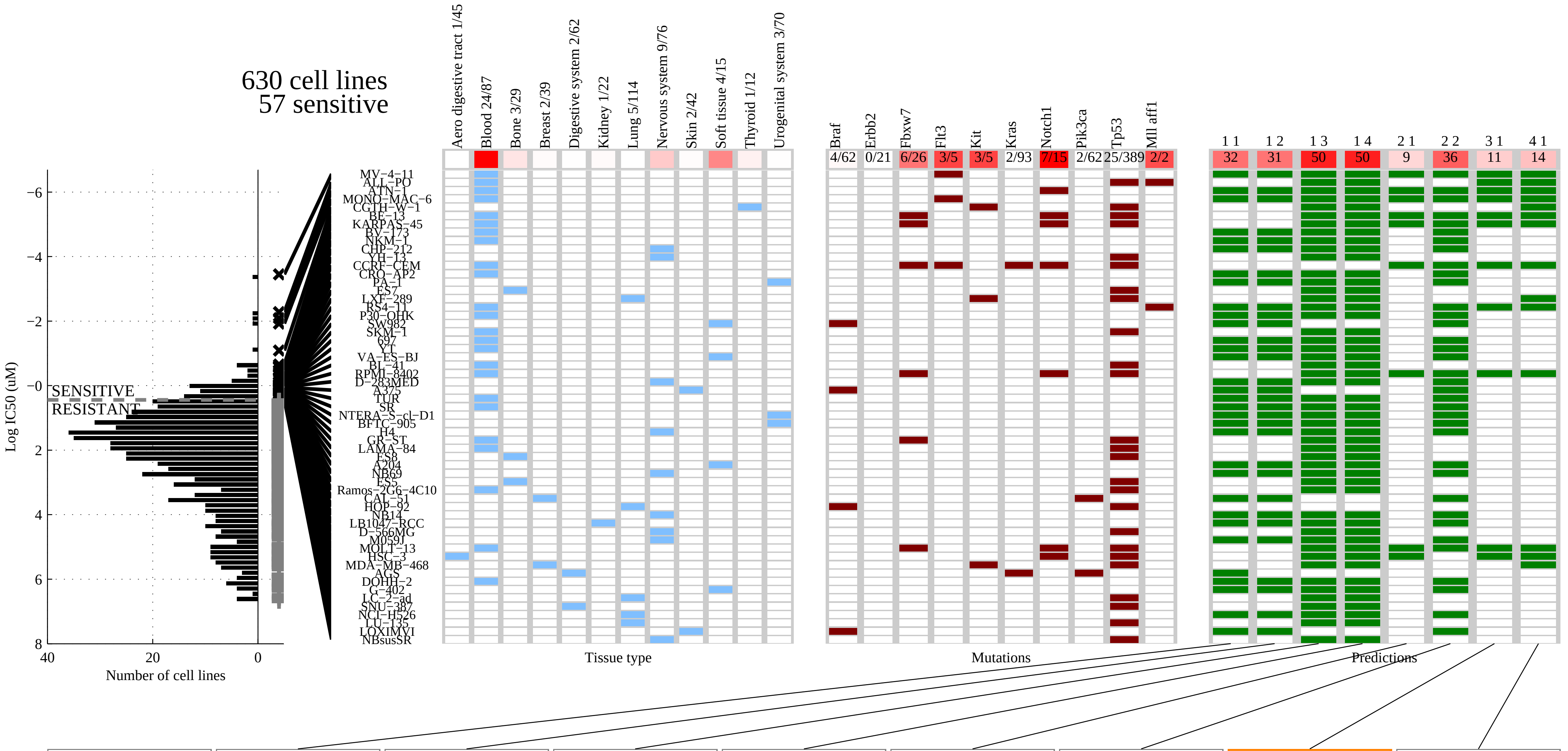

Model name	1 1	1 2	1 3	1 4	2 1	2 2	3 1	4 1
K / M	1 / 1	1 / 2	1 / 3	1 / 4	2 / 1	2 / 2	3 / 1	4 / 1
Logic formula	¬TP53	¬KRAS& ¬TP53	¬BRAF&¬KRAS& ¬PIK3C	¬BRAF&¬ERBB2& ¬KRAS&¬PIK3C	FLT3 | NOTCH	[FBXW7&NOTCH] | [¬KRAS& ¬TP53]	FLT3 | NOTCH | MLL A	FLT3 | KIT | NOTCH | MLL A
TP FP / FN TN	32 209 / 25 364	31 181 / 26 392	50 382 / 7 191	50 370 / 7 203	9 9 / 48 564	36 182 / 21 391	11 9 / 46 564	14 11 / 43 562
Specificity	0.64	0.68	0.33	0.35	0.98	0.68	0.98	0.98
Precision	0.13	0.15	0.12	0.12	0.5	0.17	0.55	0.56
Recall	0.56	0.54	0.88	0.88	0.16	0.63	0.19	0.25

ID:1038 NU−7441 −> PRKDC (DNAPK)

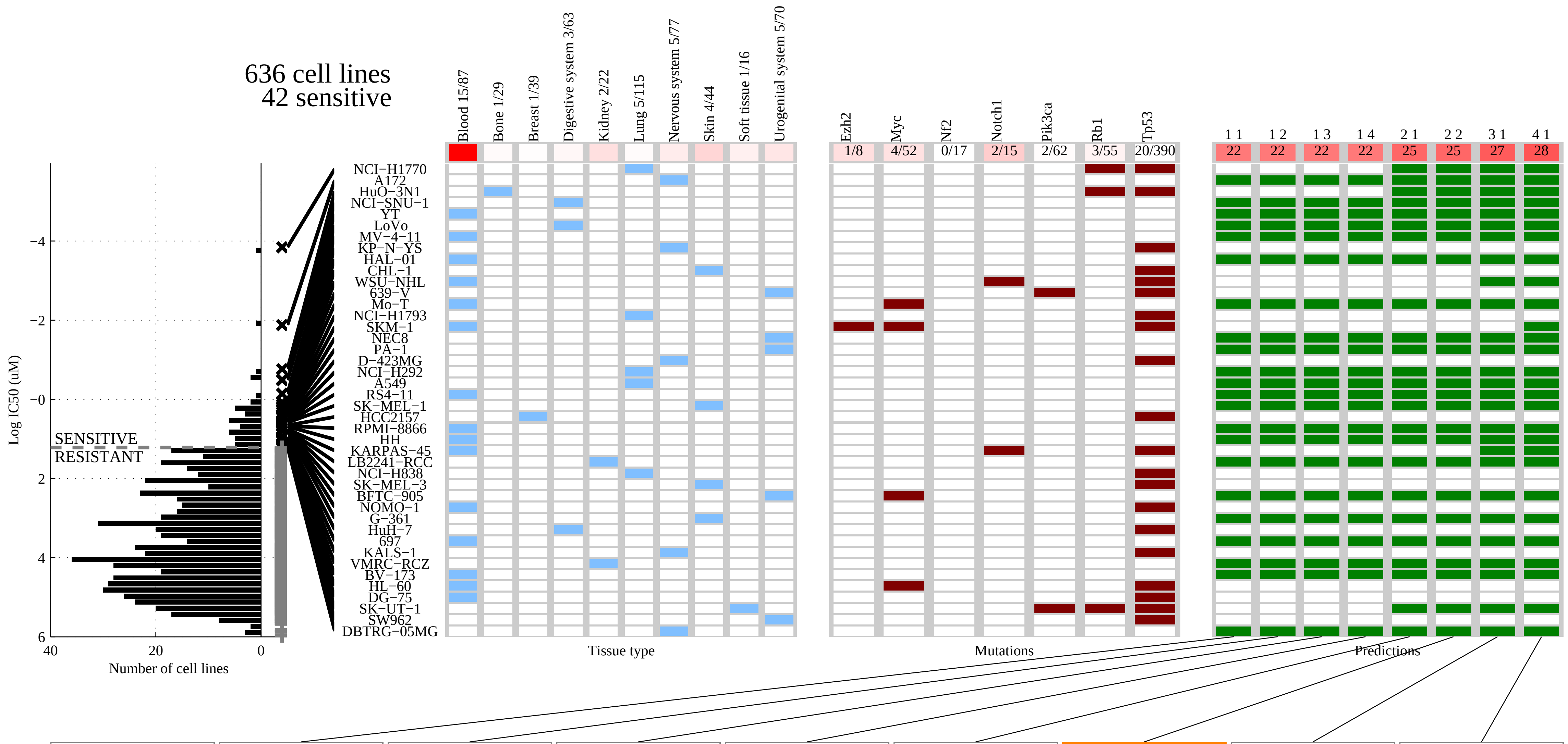

Model name	1 1	1 2	1 3	1 4	2 1	2 2	3 1	4 1
K / M	1 / 1	1 / 2	1 / 3	1 / 4	2 / 1	2 / 2	3 / 1	4 / 1
Logic formula	¬TP53	¬PIK3C& ¬TP53	¬PIK3C& ¬RB1 & ¬TP53	¬NF2 &¬PIK3C& ¬RB1 & ¬TP53	RB1 | ¬TP53	[¬MYC & RB1] | [¬PIK3C& ¬TP53]	NOTCH| RB1 | ¬TP53	EZH2 |NOTCH| RB1 | ¬TP53
TP FP	22 224	22 200	22 190	22 182	25 265	25 234	27 275	28 279
FN TN	20 370	20 394	20 404	20 412	17 329	17 360	15 319	14 315
Specificity	0.62	0.66	0.68	0.69	0.55	0.61	0.54	0.53
Precision	0.089	0.099	0.1	0.11	0.086	0.097	0.089	0.091
Recall	0.52	0.52	0.52	0.52	0.6	0.6	0.64	0.67

ID:1039 SL 0101−1 −> RSK, AURKB, PIM3

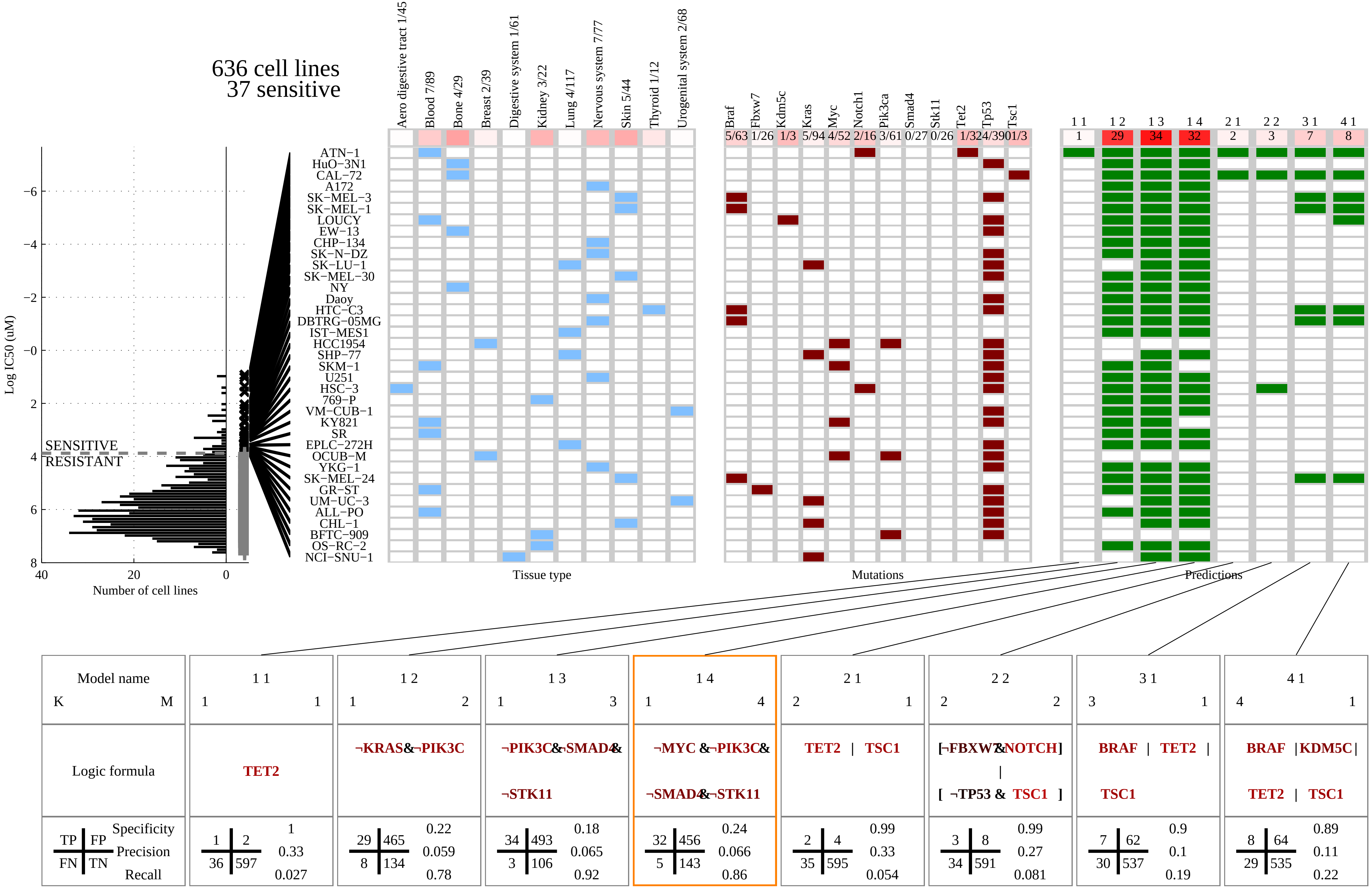

ID:1040 BI−D1870 −> RSK1/2/3/5 : PLK1 : AURKB

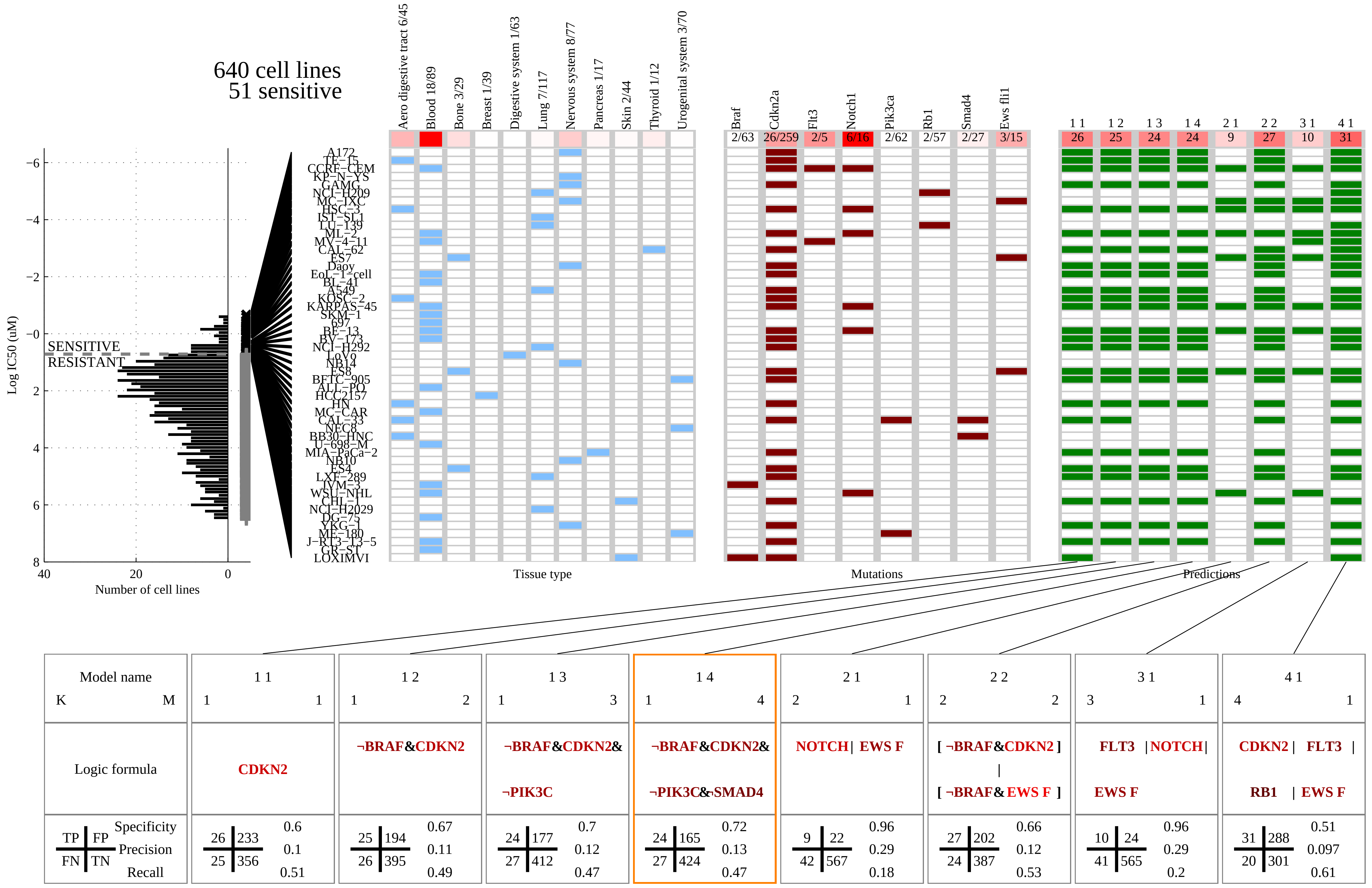

ID:1042 BIRB 0796 -> p38, JNK2

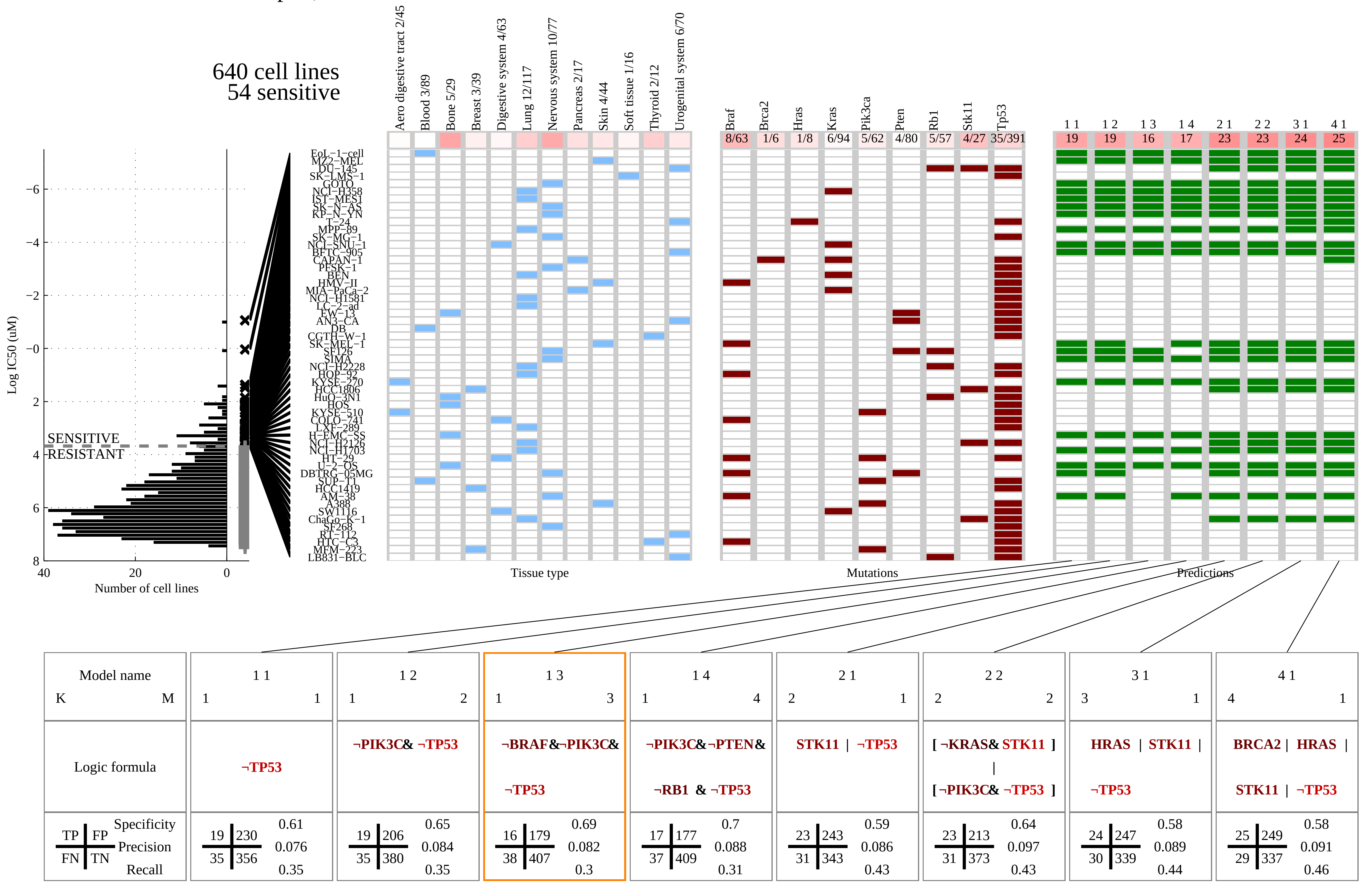

ID:1043 JNK Inhibitor VIII -> JNK

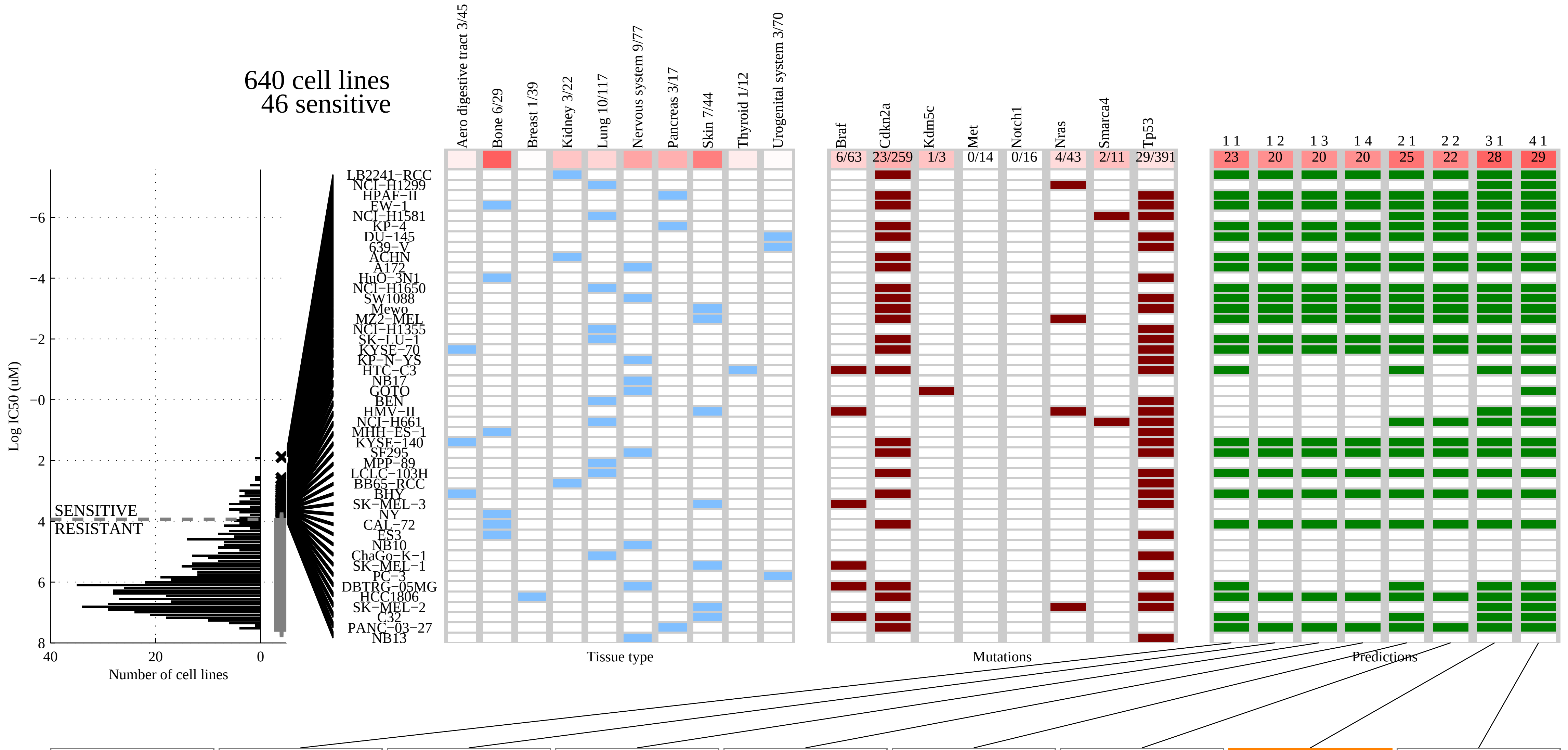

Model name	1 1	1 2	1 3	1 4	2 1	2 2	3 1	4 1
K / M	1 / 1	1 / 2	1 / 3	1 / 4	2 / 1	2 / 2	3 / 1	4 / 1
Logic formula	CDKN2	¬BRAF&CDKN2	¬BRAF&CDKN2& ¬NOTCH	¬BRAF&CDKN2& ¬MET &NOTCH	CDKN2 | SMARC	[SMARC& TP53] | [¬BRAF&CDKN2]	CDKN2 | NRAS | SMARC	CDKN2 | KDM5C | NRAS | SMARC
TP / FP	23 / 236	20 / 199	20 / 186	20 / 179	25 / 243	22 / 205	28 / 258	29 / 259
FN / TN	23 / 358	26 / 395	26 / 408	26 / 415	21 / 351	24 / 389	18 / 336	17 / 335
Specificity	0.6	0.66	0.69	0.7	0.59	0.65	0.57	0.56
Precision	0.089	0.091	0.097	0.1	0.093	0.097	0.098	0.1
Recall	0.5	0.43	0.43	0.43	0.54	0.48	0.61	0.63

ID:1046 681640 –> WEE1, CHEK1

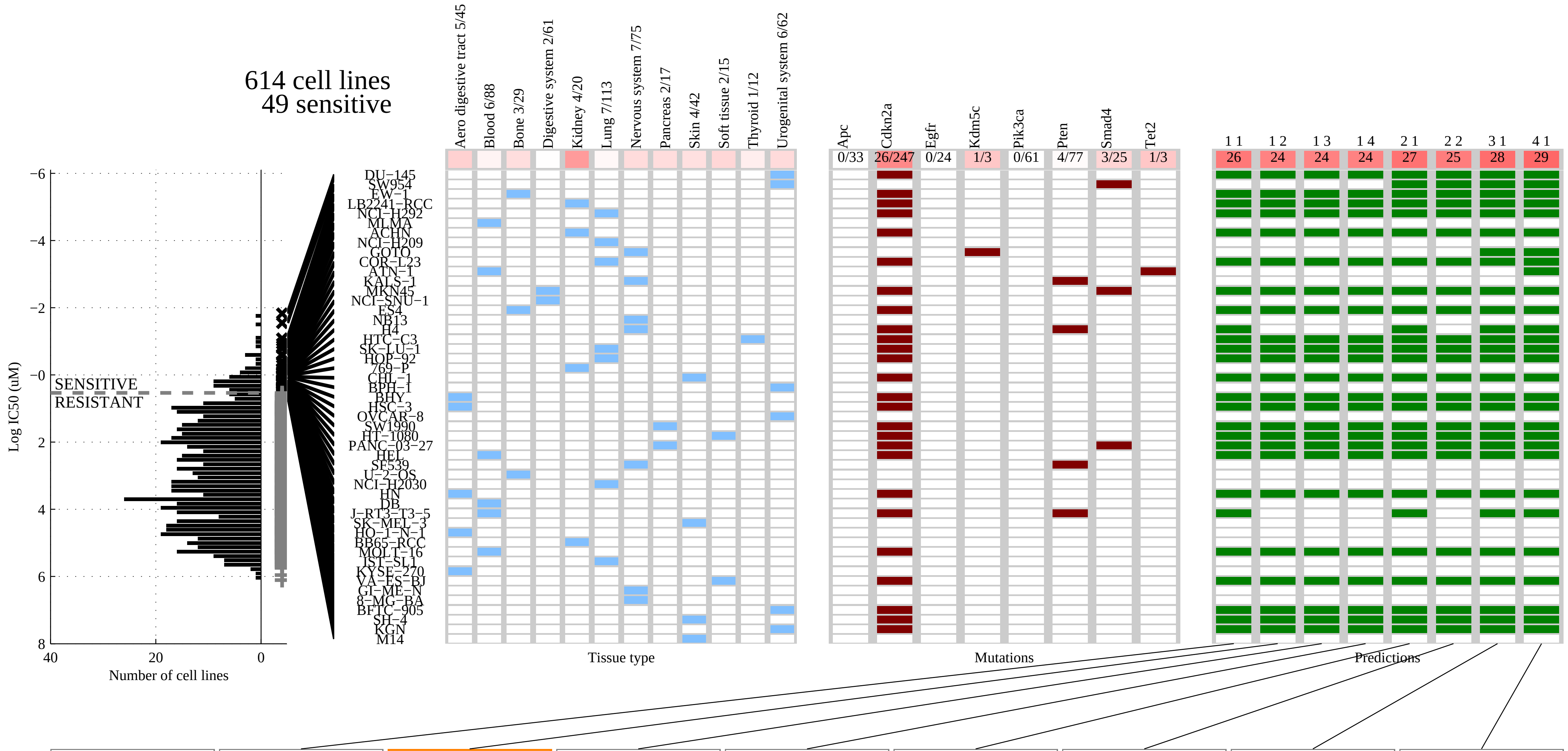

Model name	K	M	Logic formula	TP	FP	FN	TN	Specificity	Precision	Recall
1 1	1	1	**CDKN2**	26	221	23	344	0.61	0.11	0.53
1 2	1	2	**CDKN2&¬PTEN**	24	185	25	380	0.67	0.11	0.49
1 3	1	3	**CDKN2&¬PIK3C&¬PTEN**	24	166	25	399	0.71	0.13	0.49
1 4	1	4	**CDKN2&¬EGFR&¬PIK3C&¬PTEN**	24	157	25	408	0.72	0.13	0.49
2 1	2	1	**CDKN2 | SMAD4**	27	230	22	335	0.59	0.11	0.55
2 2	2	2	**[CDKN2&¬PTEN] | [¬APC &SMAD4]**	25	191	24	374	0.66	0.12	0.51
3 1	3	1	**CDKN2 | KDM5C | SMAD4**	28	231	21	334	0.59	0.11	0.57
4 1	4	1	**CDKN2 | KDM5C | SMAD4 | TET2**	29	232	20	333	0.59	0.11	0.59

ID:1047 Nutlin−3a −> MDM2

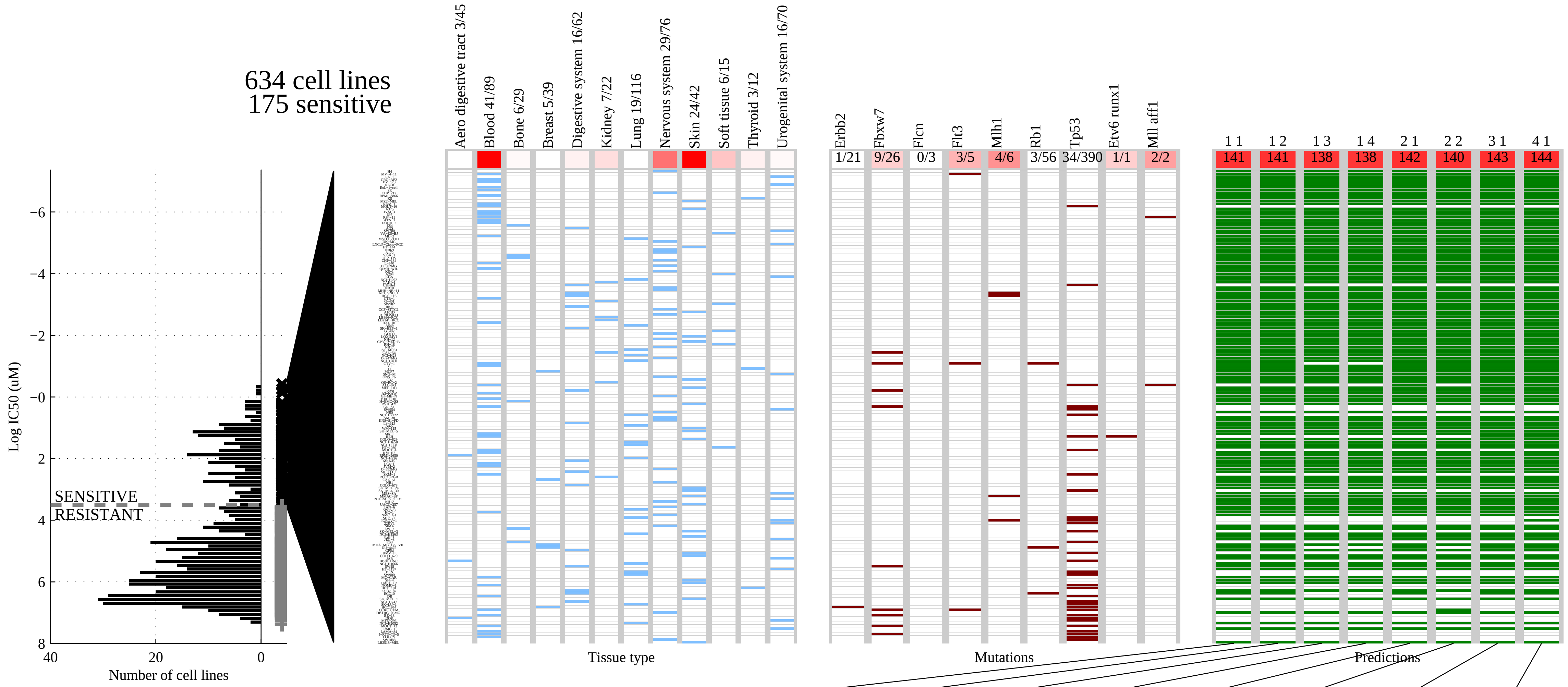

Model name (K, M)	1 1 (K 1, M 1)	1 2 (K 1, M 2)	1 3 (K 1, M 3)	1 4 (K 1, M 4)	2 1 (K 2, M 1)	2 2 (K 2, M 2)	3 1 (K 3, M 1)	4 1 (K 4, M 1)
Logic formula	¬TP53	¬ERBB2 & ¬TP53	¬ERBB2 & ¬RB1 & ¬TP53	¬ERBB2 & ¬FLCN & ¬RB1 & ¬TP53	¬TP53 | MLL A	[¬RB1 & ¬TP53] | [FBXW7 & FLT3]	¬TP53 | ETV6 | MLL A	MLH1 | ¬TP53 | ETV6 | MLL A
TP	141	141	138	138	142	140	143	144
FP	103	99	90	88	103	94	103	104
FN	34	34	37	37	33	35	32	31
TN	356	360	369	371	356	365	356	355
Specificity	0.78	0.78	0.8	0.81	0.78	0.8	0.78	0.77
Precision	0.58	0.59	0.61	0.61	0.58	0.6	0.58	0.58
Recall	0.81	0.81	0.79	0.79	0.81	0.8	0.82	0.82

ID:1049 PD−173074 −> FGFR1, FGFR3

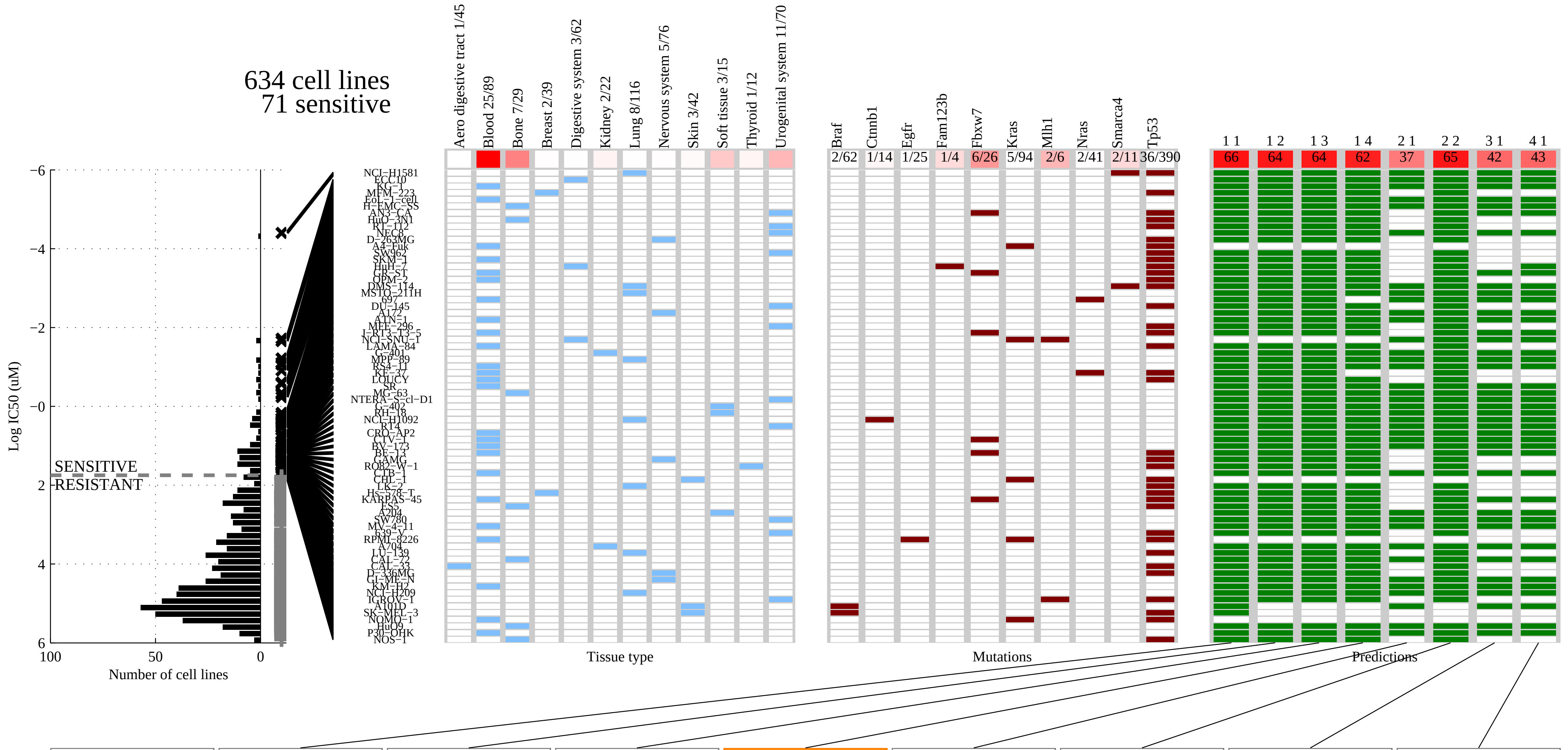

Model name	1 1	1 2	1 3	1 4	2 1	2 2	3 1	4 1
K / M	1 / 1	1 / 2	1 / 3	1 / 4	2 / 1	2 / 2	3 / 1	4 / 1
Logic formula	¬KRAS	¬BRAF&¬KRAS	¬BRAF&¬EGFR& ¬KRAS	¬BRAF&¬EGFR& ¬KRAS&¬NRAS	SMARC | ¬TP53	[¬CTNNB& MLH1] | [¬BRAF&¬KRAS]	FBXW7 | SMARC | ¬TP53	FAM12 | FBXW7 | SMARC | ¬TP53
TP FP / FN TN	66 474 / 5 89	64 417 / 7 146	64 396 / 7 167	62 360 / 9 203	37 215 / 34 348	65 417 / 6 146	42 231 / 29 332	43 232 / 28 331
Specificity	0.16	0.26	0.3	0.36	0.62	0.26	0.59	0.59
Precision	0.12	0.13	0.14	0.15	0.15	0.13	0.15	0.16
Recall	0.93	0.9	0.9	0.87	0.52	0.92	0.59	0.61

ID:1050 ZM−447439 -> AURKB

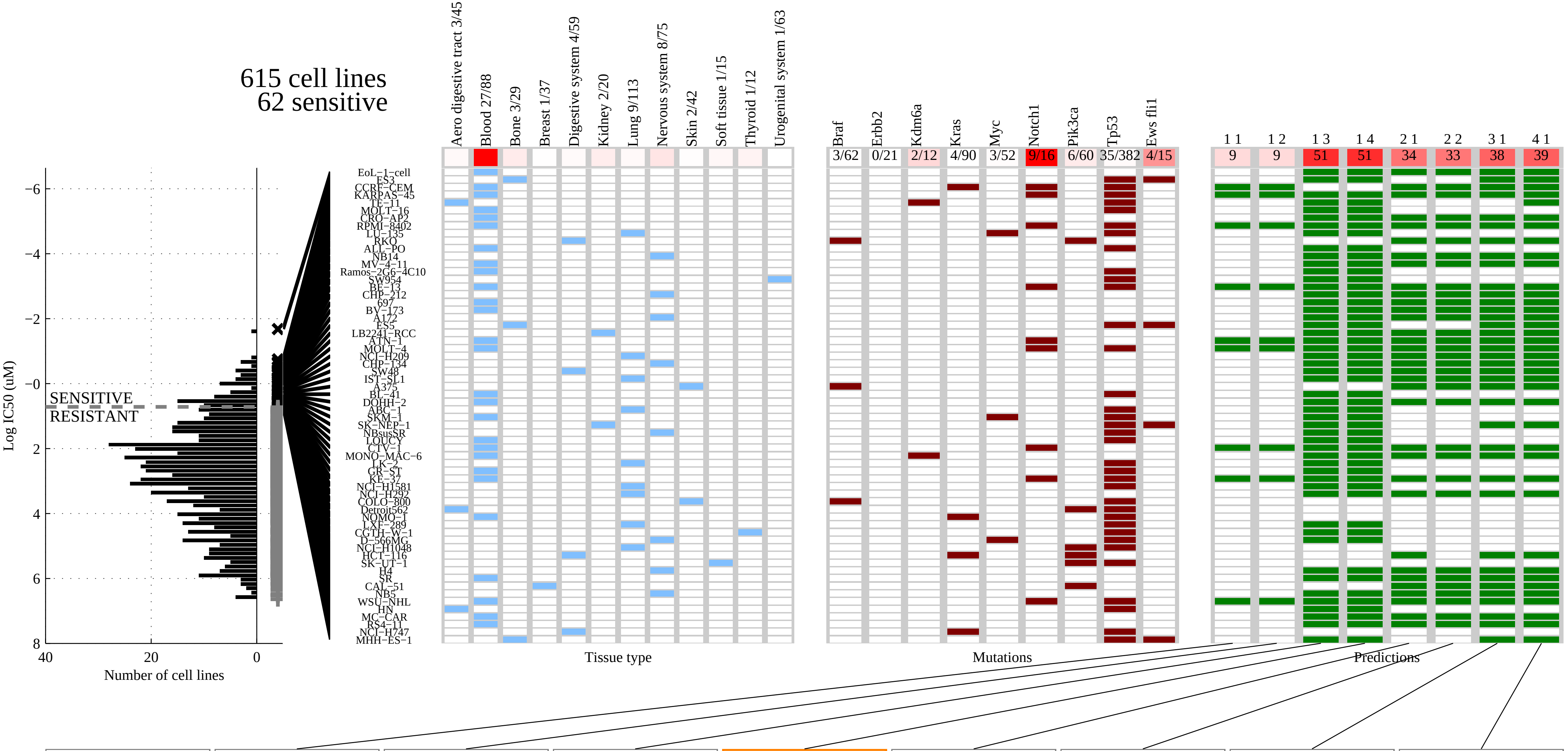

Model name (K, M)	1 1	1 2	1 3	1 4	2 1	2 2	3 1	4 1
K / M	1 / 1	1 / 2	1 / 3	1 / 4	2 / 1	2 / 2	3 / 1	4 / 1
Logic formula	NOTCH	¬MYC & NOTCH	¬BRAF & ¬KRAS & ¬PIK3C	¬BRAF & ¬ERBB2 & ¬KRAS & ¬PIK3C	NOTCH | ¬TP53	[NOTCH & TP53] | [¬KRAS & ¬TP53]	NOTCH | ¬TP53 | EWS F	KDM6A | NOTCH | ¬TP53 | EWS F
TP	9	9	51	51	34	33	38	39
FP	7	6	371	359	211	185	219	225
FN	53	53	11	11	28	29	24	23
TN	546	547	182	194	342	368	334	328
Specificity	0.99	0.99	0.33	0.35	0.62	0.67	0.6	0.59
Precision	0.56	0.6	0.12	0.12	0.14	0.15	0.15	0.15
Recall	0.15	0.15	0.82	0.82	0.55	0.53	0.61	0.63

ID:1052 RO−3306 −> CDK1

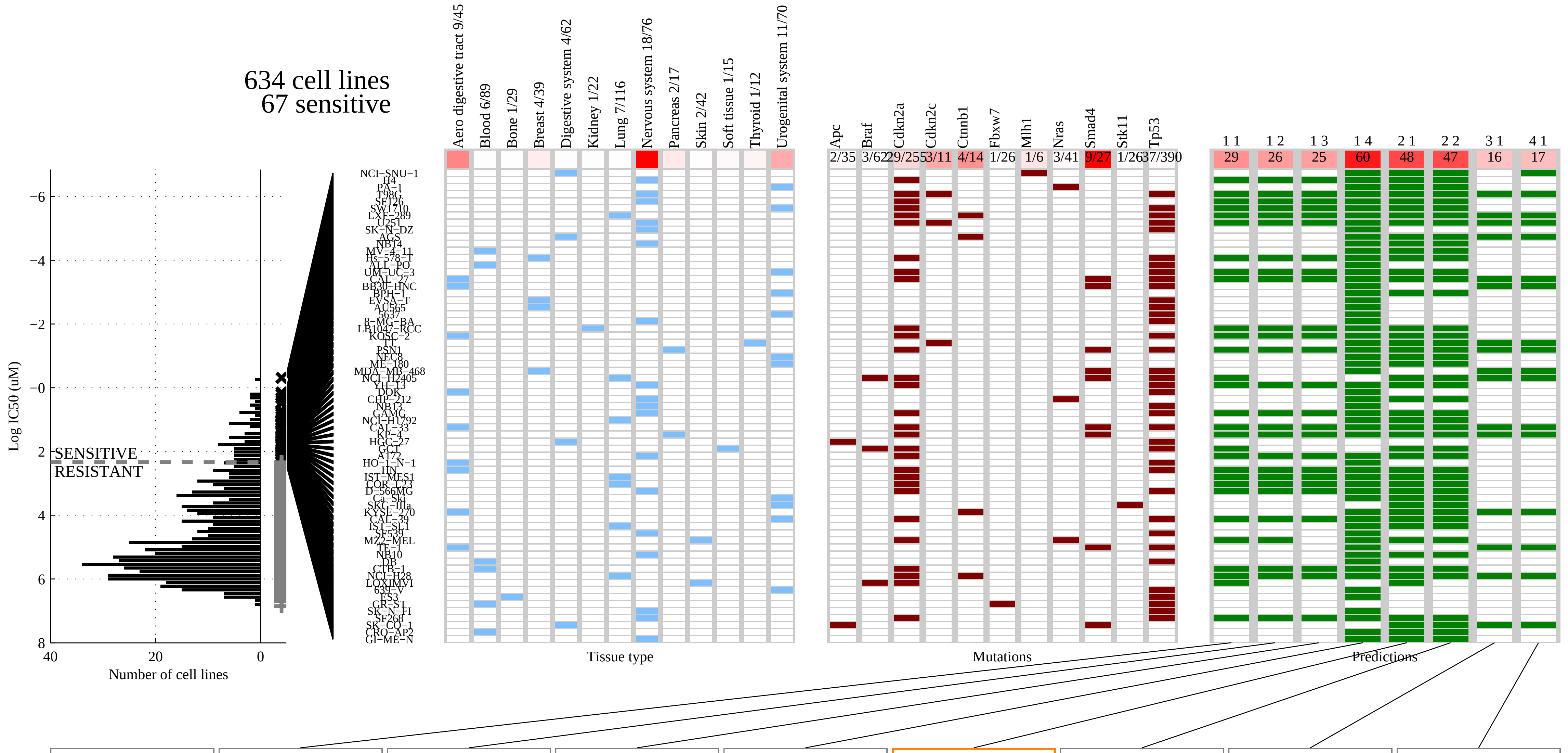

Model name	1 1	1 2	1 3	1 4	2 1	2 2	3 1	4 1
K / M	1 / 1	1 / 2	1 / 3	1 / 4	2 / 1	2 / 2	3 / 1	4 / 1
Logic formula	CDKN2	¬BRAF&CDKN2	¬BRAF&CDKN2& ¬NRAS	¬APC &¬BRAF& ¬FBXW7&¬STK11	CDKN2 | ¬TP53	[¬BRAF& ¬TP53] | [CDKN2& TP53]	CDKN2 | CTNNB | SMAD4	CDKN2 | CTNNB | MLH1 | SMAD4
TP FP / FN TN	29 226 / 38 341	26 190 / 41 377	25 169 / 42 398	60 440 / 7 127	48 343 / 19 224	47 313 / 20 254	16 35 / 51 532	17 38 / 50 529
Specificity	0.6	0.66	0.7	0.22	0.4	0.45	0.94	0.93
Precision	0.11	0.12	0.13	0.12	0.12	0.13	0.31	0.31
Recall	0.43	0.39	0.37	0.9	0.72	0.7	0.24	0.25

ID:1053 MK−2206 −> AKT1, AKT2

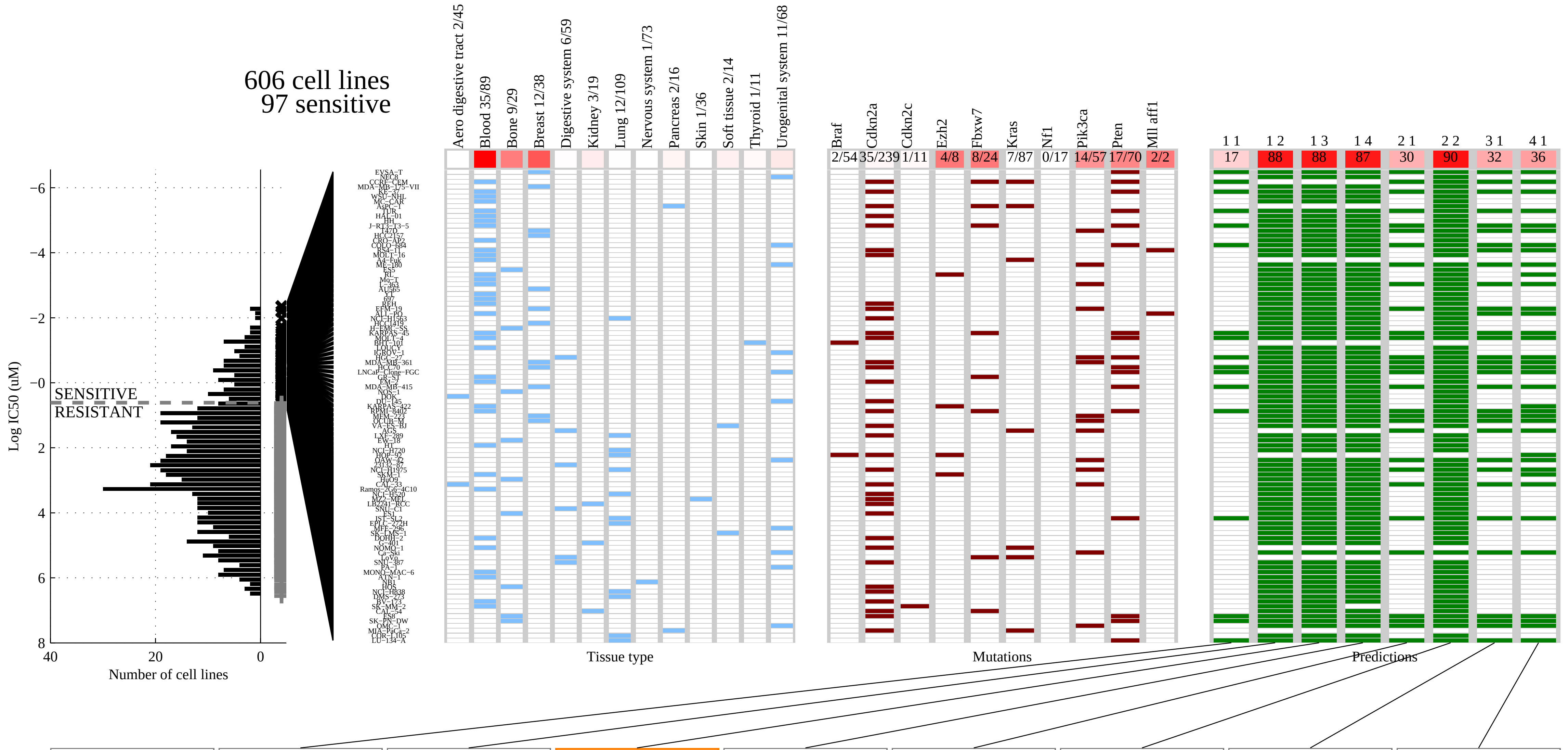

Model name (K, M)	1 1	1 2	1 3	1 4	2 1	2 2	3 1	4 1
K / M	1 / 1	1 / 2	1 / 3	1 / 4	2 / 1	2 / 2	3 / 1	4 / 1
Logic formula	PTEN	¬BRAF&¬KRAS	¬BRAF&¬KRAS& ¬NF1	¬BRAF&¬CDKN2& ¬KRAS& ¬NF1	PIK3C | PTEN	[¬BRAF&¬KRAS] | [CDKN2&FBXW7]	PIK3C | PTEN | MLL A	EZH2 | PIK3C | PTEN | MLL A
TP	17	88	88	87	30	90	32	36
FP	53	380	365	356	94	380	94	98
FN	80	9	9	10	67	7	65	61
TN	456	129	144	153	415	129	415	411
Specificity	0.9	0.25	0.28	0.3	0.82	0.25	0.82	0.81
Precision	0.24	0.19	0.19	0.2	0.24	0.19	0.25	0.27
Recall	0.18	0.91	0.91	0.9	0.31	0.93	0.33	0.37

ID:1054 PD−0332991 −> CDK4, CDK6

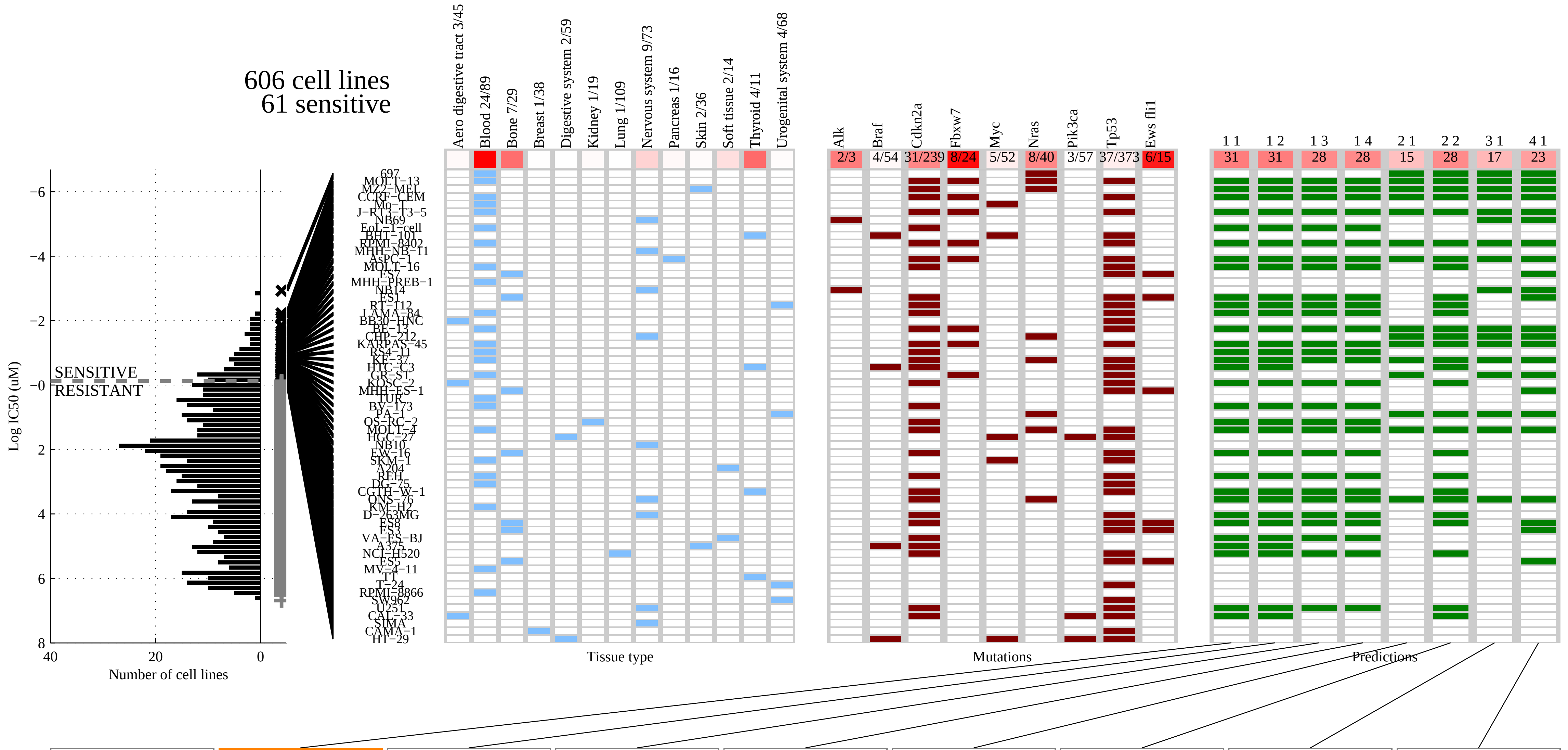

Model name	1 1	1 2	1 3	1 4	2 1	2 2	3 1	4 1
K / M	1 / 1	1 / 2	1 / 3	1 / 4	2 / 1	2 / 2	3 / 1	4 / 1
Logic formula	**CDKN2**	**CDKN2& ¬MYC**	**¬BRAF&CDKN2& ¬PIK3C**	**¬BRAF&CDKN2& ¬MYC &¬PIK3C**	**FBXW7 | NRAS**	**[CDKN2& TP53] | [NRAS & ¬TP53]**	**ALK | FBXW7 | NRAS**	**ALK | FBXW7 | NRAS | EWS F**
TP FP	31 208	31 189	28 159	28 144	15 46	28 131	17 47	23 56
FN TN	30 337	30 356	33 386	33 401	46 499	33 414	44 498	38 489
Specificity	0.62	0.65	0.71	0.74	0.92	0.76	0.91	0.9
Precision	0.13	0.14	0.15	0.16	0.25	0.18	0.27	0.29
Recall	0.51	0.51	0.46	0.46	0.25	0.46	0.28	0.38

ID:1057 NVP−BEZ235 −> PI3K (Class 1) and MTORC1/2

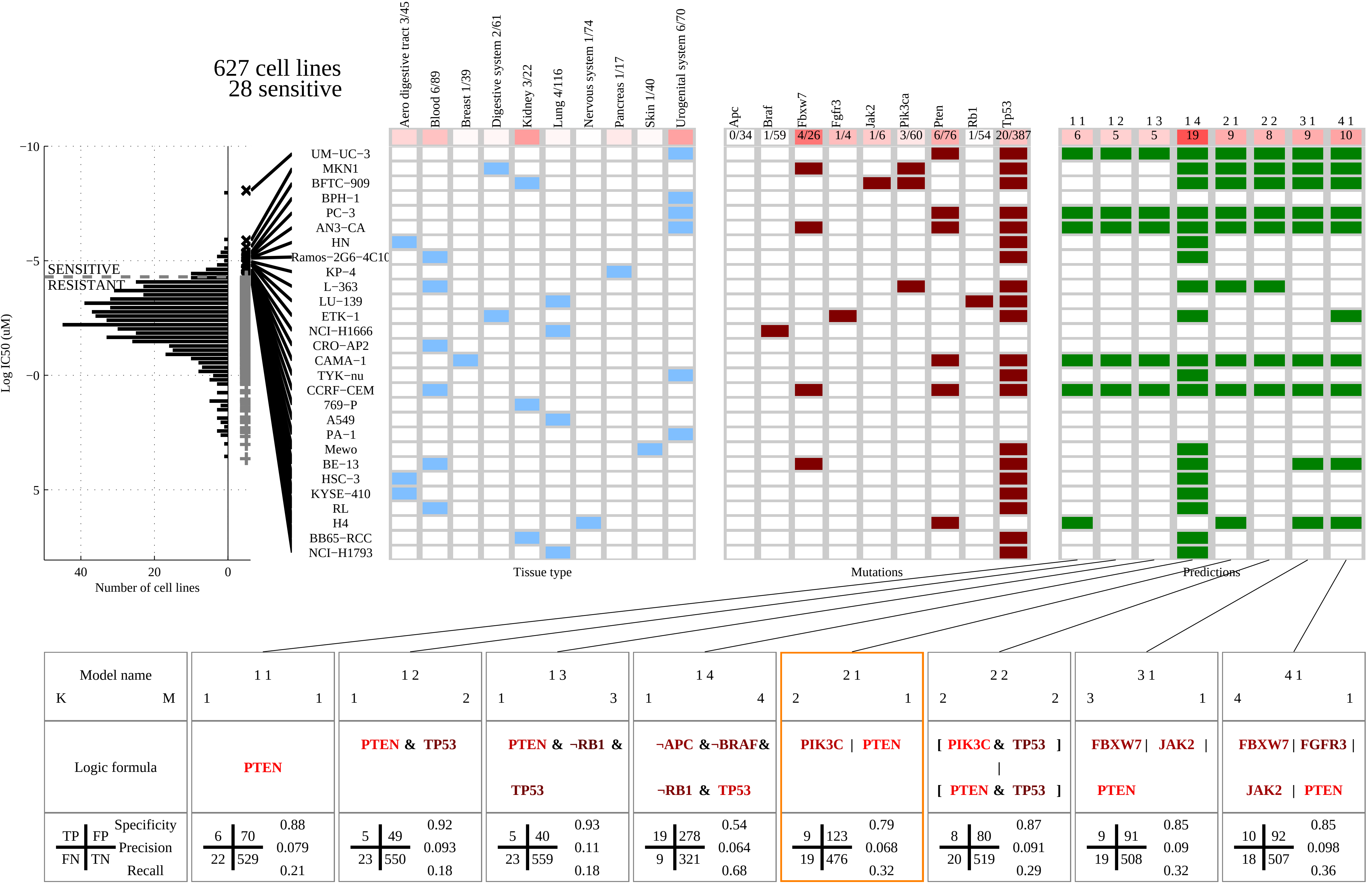

ID:1058 GDC0941 −> PI3K (class 1)

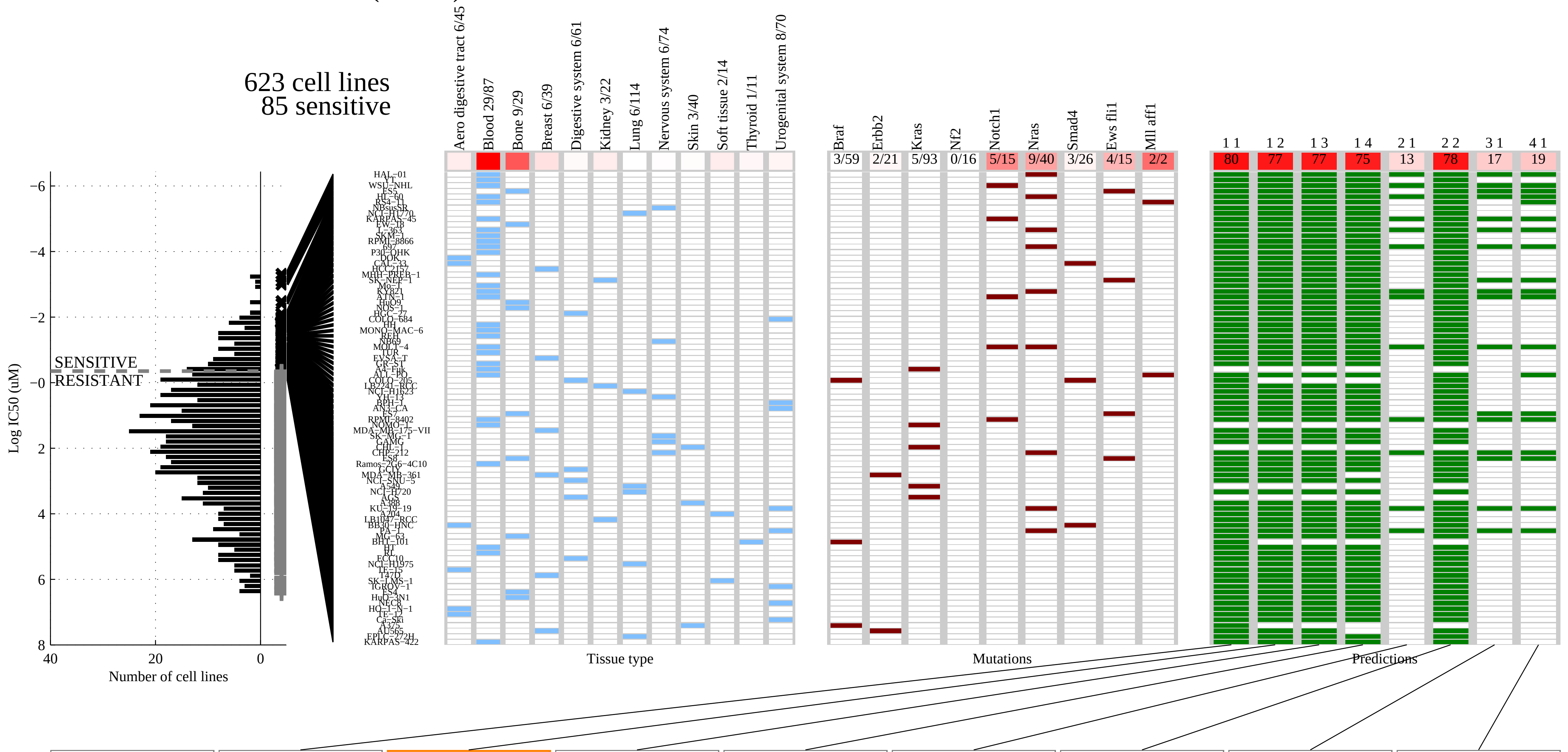

Model name (K, M)	1 1 (1, 1)	1 2 (1, 2)	1 3 (1, 3)	1 4 (1, 4)	2 1 (2, 1)	2 2 (2, 2)	3 1 (3, 1)	4 1 (4, 1)
Logic formula	¬KRAS	¬BRAF&¬KRAS	¬BRAF&¬KRAS& ¬NF2	¬BRAF&¬ERBB2& ¬KRAS& ¬NF2	NOTCH| NRAS	[¬BRAF&¬KRAS] | [BRAF &SMAD4]	NOTCH| NRAS | EWS F	NOTCH| NRAS | EWS F | MLL A
TP / FP	80 / 450	77 / 397	77 / 386	75 / 369	13 / 37	78 / 399	17 / 48	19 / 48
FN / TN	5 / 88	8 / 141	8 / 152	10 / 169	72 / 501	7 / 139	68 / 490	66 / 490
Specificity	0.16	0.26	0.28	0.31	0.93	0.26	0.91	0.91
Precision	0.15	0.16	0.17	0.17	0.26	0.16	0.26	0.28
Recall	0.94	0.91	0.91	0.88	0.15	0.92	0.2	0.22

ID:1059 AZD8055 −> MTORC1/2

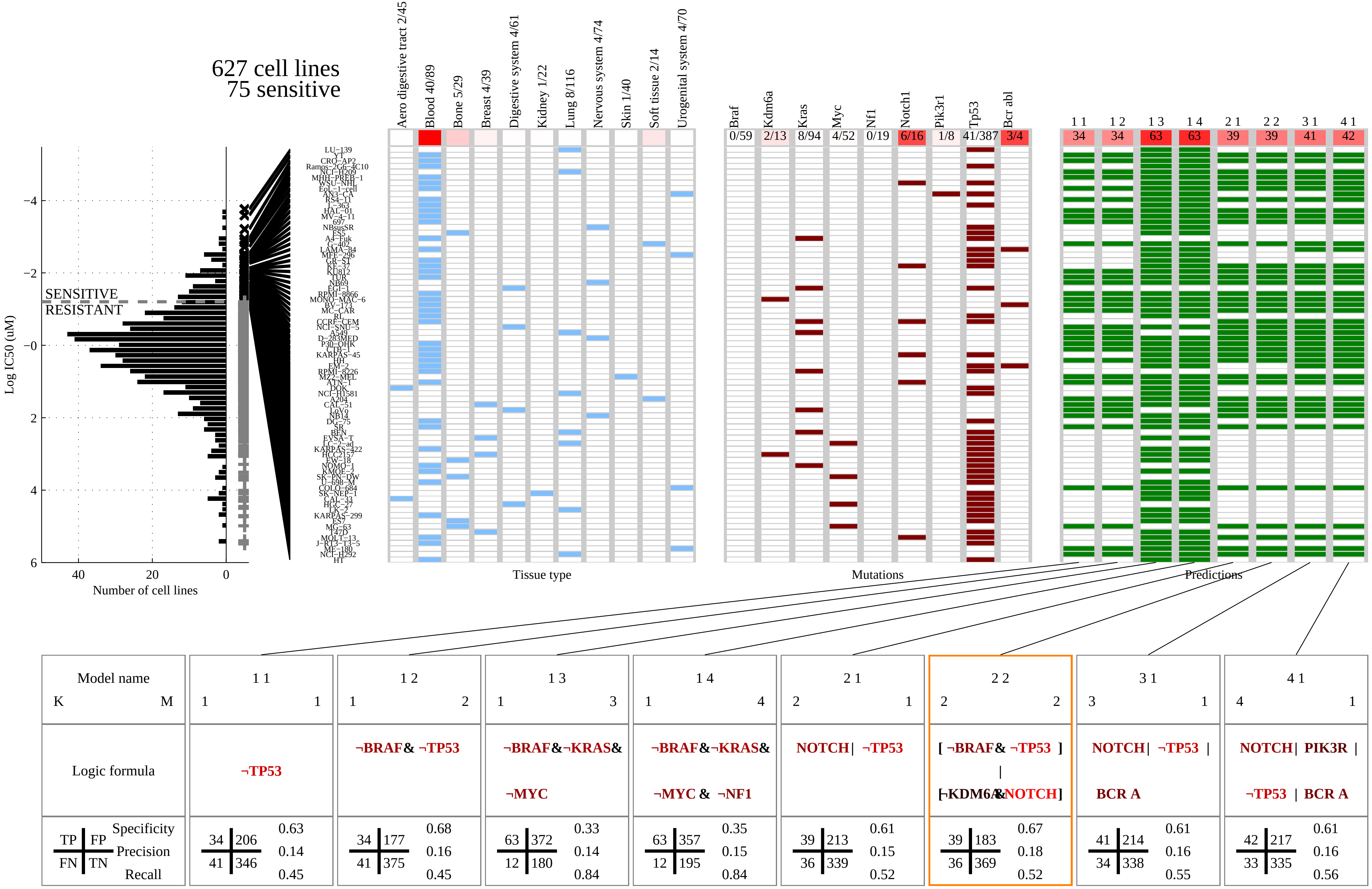

ID:1060 PD−0325901 -> MAP2K1 (MEK1), MAP2K2 (MEK2)

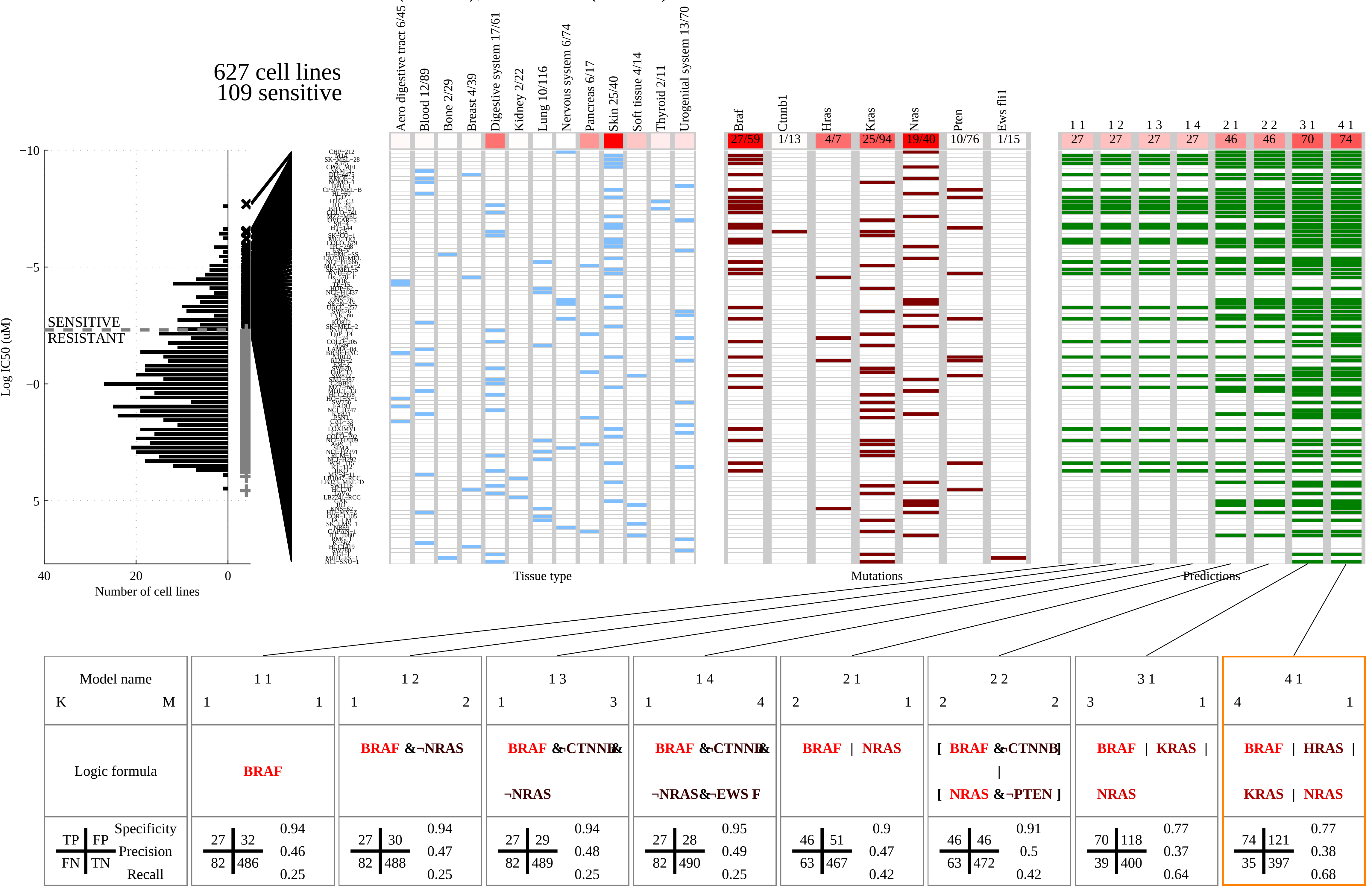

ID:1061 SB590885 –> BRAF

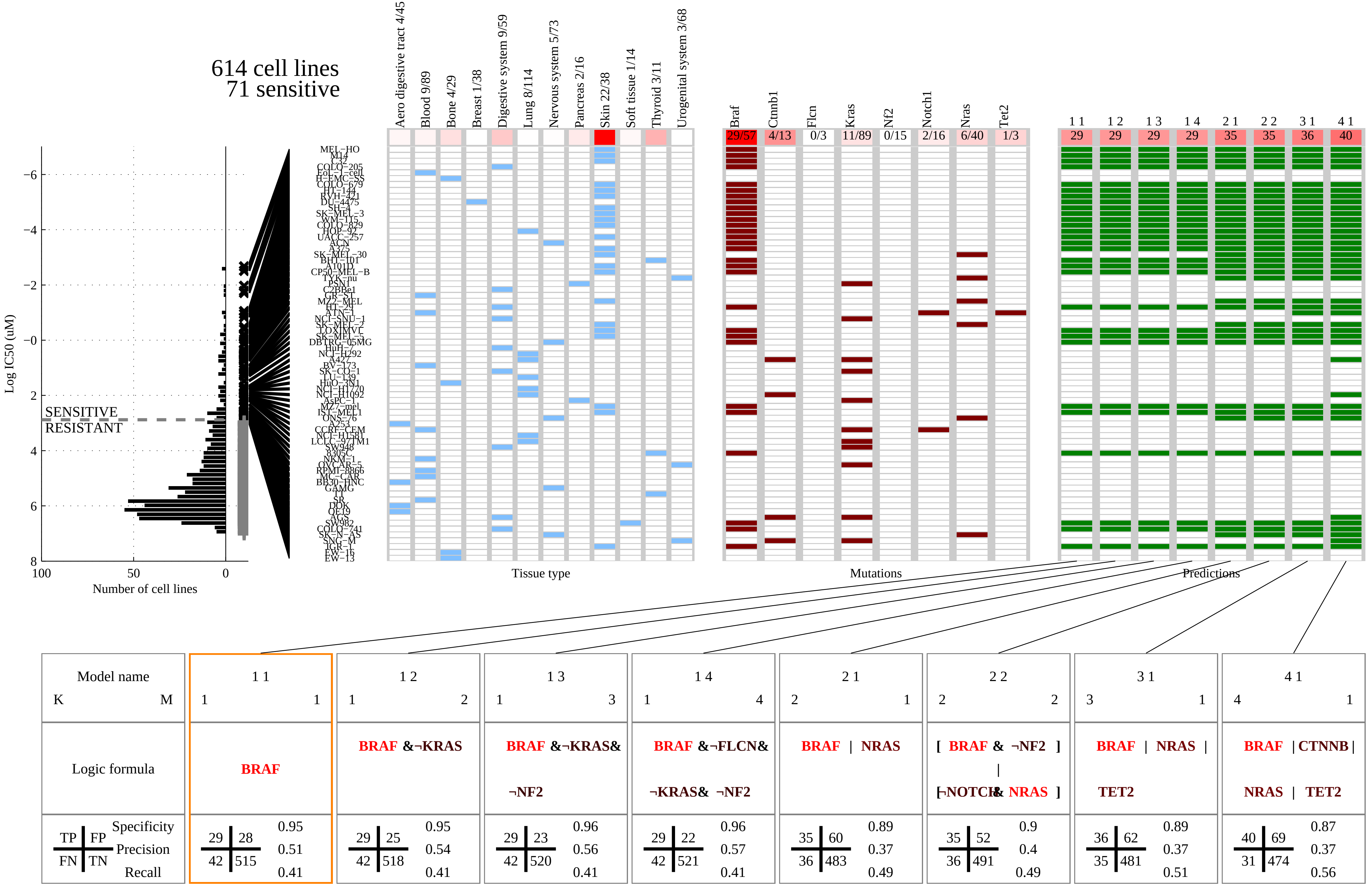

ID:1062 AZD6244 −> MAP2K1 (MEK1), MAP2K2 (MEK2)

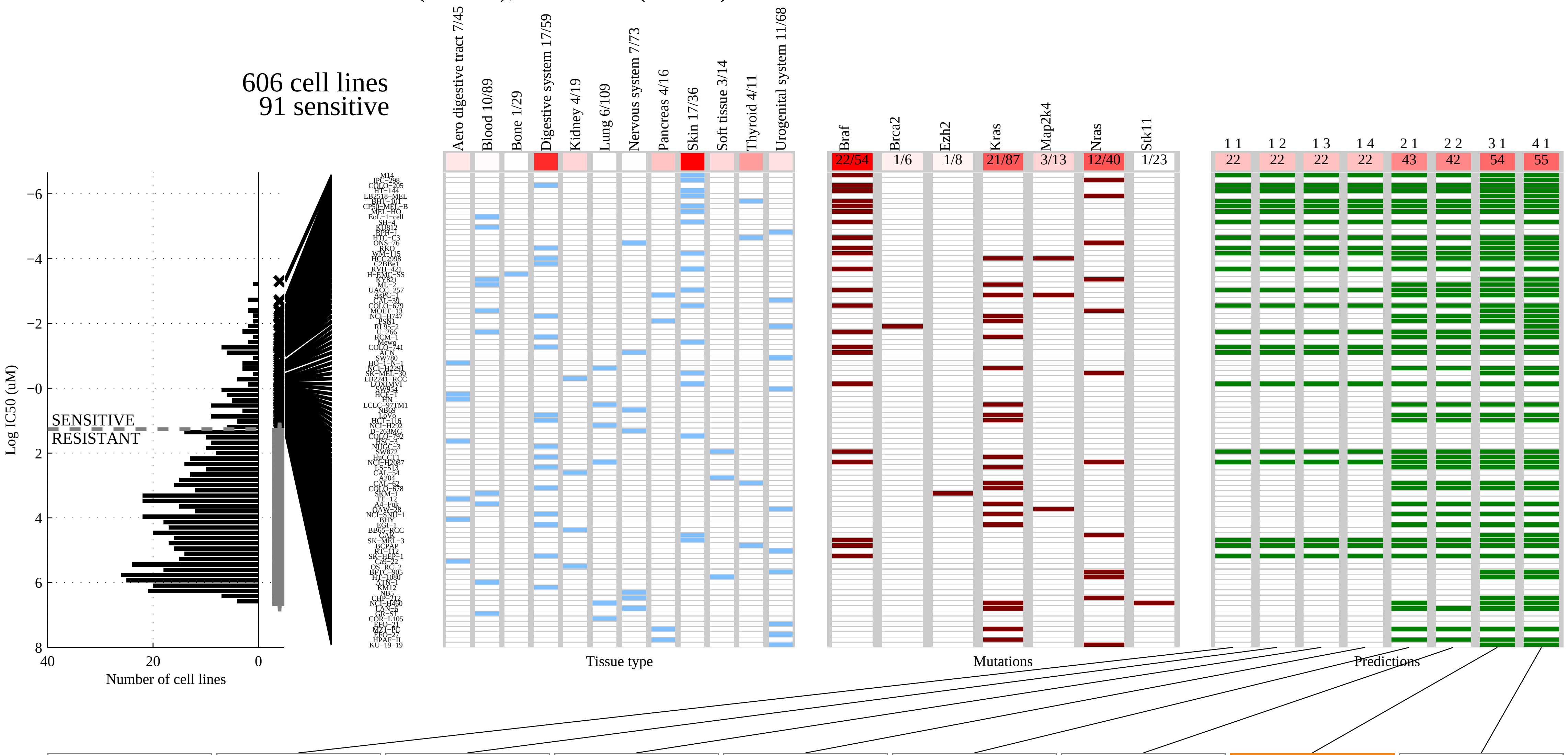

Model name (K, M)	1 1 (1, 1)	1 2 (1, 2)	1 3 (1, 3)	1 4 (1, 4)	2 1 (2, 1)	2 2 (2, 2)	3 1 (3, 1)	4 1 (4, 1)
Logic formula	BRAF	BRAF &¬STK11	BRAF &MAP2K& ¬STK11	BRAF &¬EZH2& ¬MAP2K&¬STK11	BRAF | KRAS	[KRAS &¬STK11] | [BRAF &¬STK11]	BRAF | KRAS | NRAS	BRAF | BRCA2 | KRAS | NRAS
TP FP / FN TN	22 32 / 69 483	22 29 / 69 486	22 26 / 69 489	22 22 / 69 493	43 95 / 48 420	42 85 / 49 430	54 122 / 37 393	55 124 / 36 391
Specificity	0.94	0.94	0.95	0.96	0.82	0.83	0.76	0.76
Precision	0.41	0.43	0.46	0.5	0.31	0.33	0.31	0.31
Recall	0.24	0.24	0.24	0.24	0.47	0.46	0.59	0.6

ID:1067 CCT007093 –> PPM1D

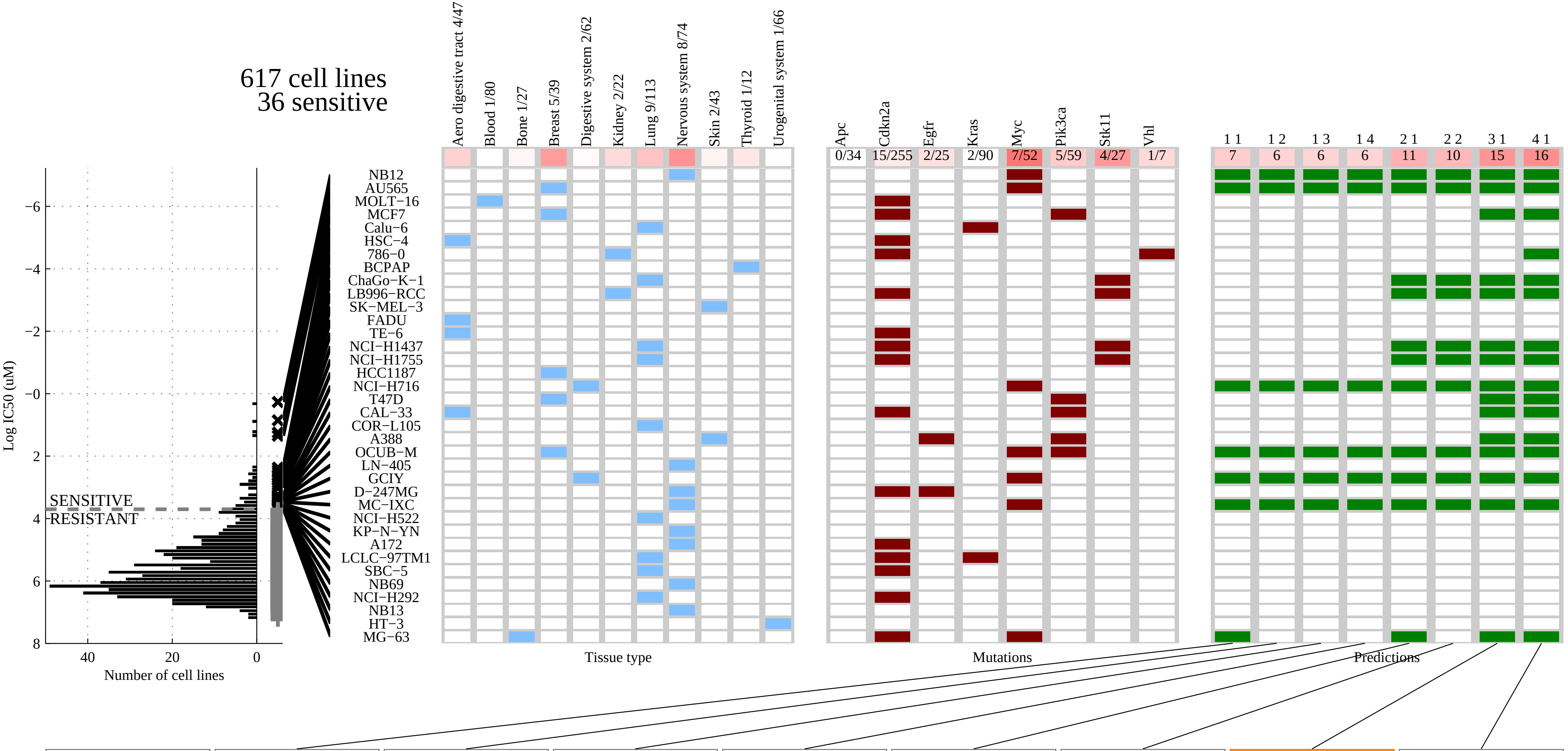

Model name	1 1	1 2	1 3	1 4	2 1	2 2	3 1	4 1
K / M	1 / 1	1 / 2	1 / 3	1 / 4	2 / 1	2 / 2	3 / 1	4 / 1
Logic formula	MYC	¬CDKN2& MYC	¬APC &¬CDKN2& MYC	¬APC &¬CDKN2& ¬EGFR& MYC	MYC | STK11	[¬KRAS& STK11] | [¬CDKN2& MYC]	MYC | PIK3C | STK11	MYC | PIK3C | STK11 | VHL
TP / FP	7 / 45	6 / 27	6 / 22	6 / 20	11 / 66	10 / 41	15 / 111	16 / 117
FN / TN	29 / 536	30 / 554	30 / 559	30 / 561	25 / 515	26 / 540	21 / 470	20 / 464
Specificity	0.92	0.95	0.96	0.97	0.89	0.93	0.81	0.8
Precision	0.13	0.18	0.21	0.23	0.14	0.2	0.12	0.12
Recall	0.19	0.17	0.17	0.17	0.31	0.28	0.42	0.44

ID:1069 EHT 1864 –> Rac GTPases

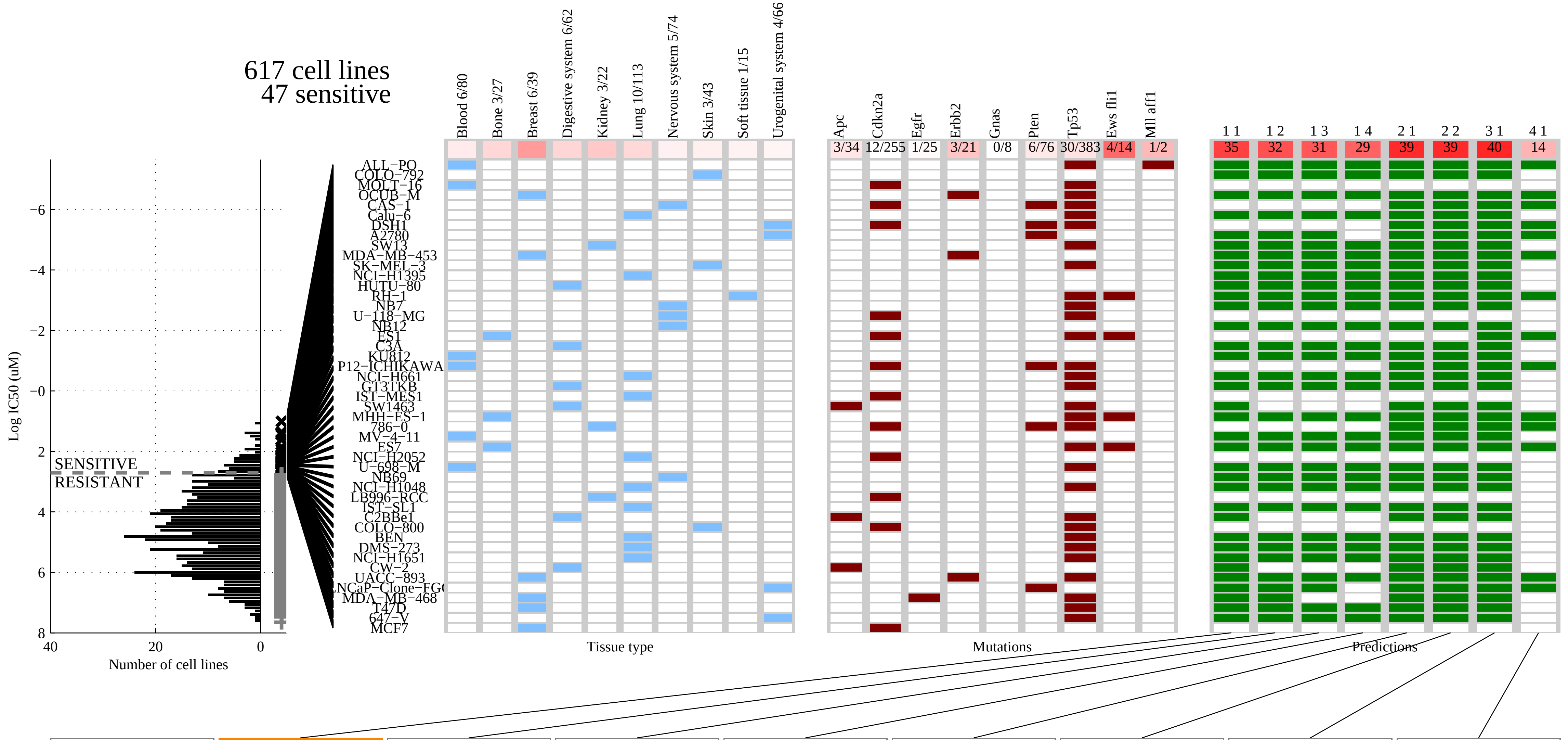

Model name	1 1	1 2	1 3	1 4	2 1	2 2	3 1	4 1
K / M	1 / 1	1 / 2	1 / 3	1 / 4	2 / 1	2 / 2	3 / 1	4 / 1
Logic formula	¬CDKN2	¬APC &¬CDKN2	¬APC &¬CDKN2& ¬EGFR	¬APC &¬CDKN2& ¬EGFR&¬PTEN	¬CDKN2| PTEN	[¬CDKN2&¬GNAS] | [PTEN & TP53]	¬CDKN2| PTEN | EWS F	ERBB2 | PTEN | EWS F | MLL A
TP / FP	35 / 327	32 / 298	31 / 286	29 / 253	39 / 362	39 / 342	40 / 365	14 / 97
FN / TN	12 / 243	15 / 272	16 / 284	18 / 317	8 / 208	8 / 228	7 / 205	33 / 473
Specificity	0.43	0.48	0.5	0.56	0.36	0.4	0.36	0.83
Precision	0.097	0.097	0.098	0.1	0.097	0.1	0.099	0.13
Recall	0.74	0.68	0.66	0.62	0.83	0.83	0.85	0.3

ID:1072 BMS−708163 −> g−secretase

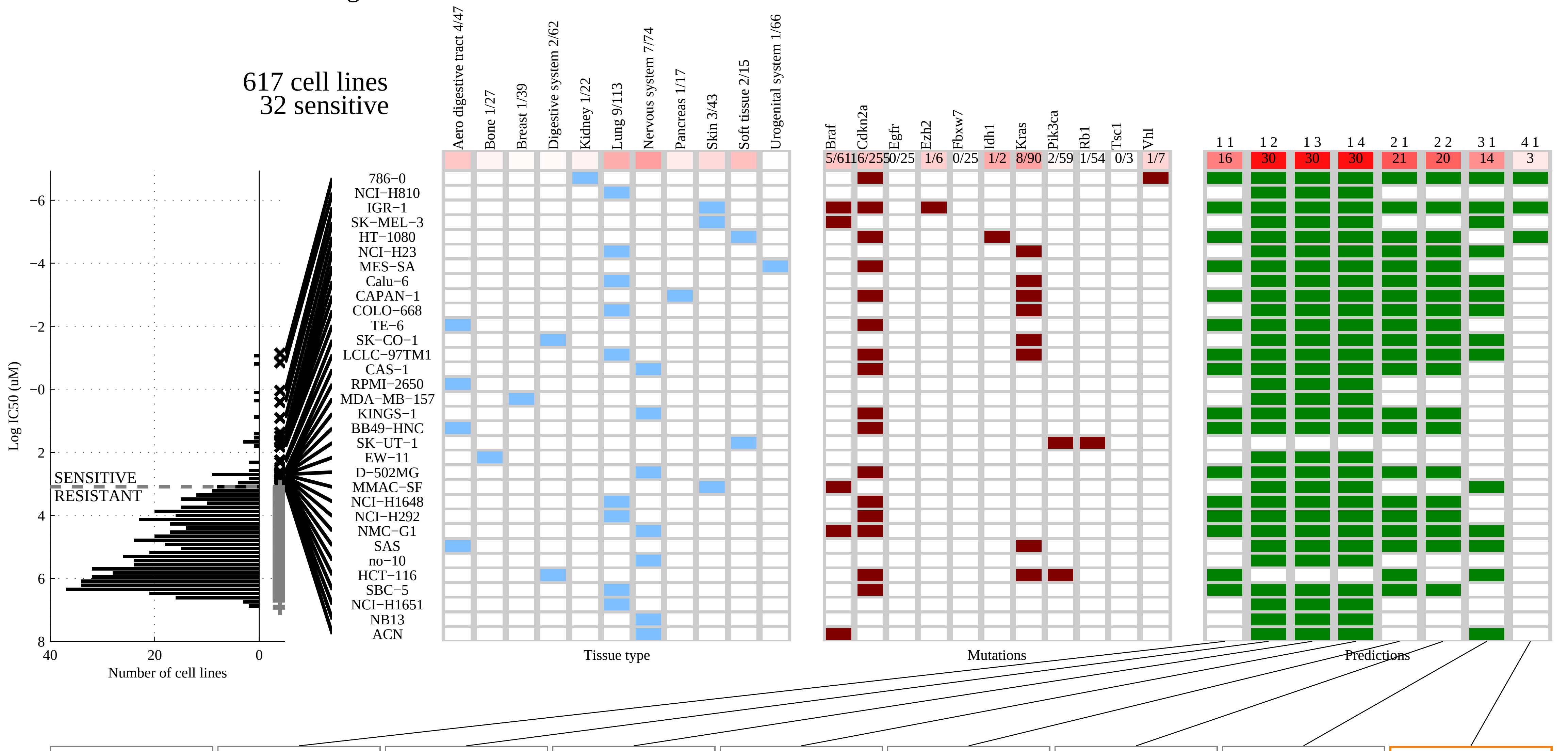

Model name (K, M)	1 1 (K=1, M=1)	1 2 (K=1, M=2)	1 3 (K=1, M=3)	1 4 (K=1, M=4)	2 1 (K=2, M=1)	2 2 (K=2, M=2)	3 1 (K=3, M=1)	4 1 (K=4, M=1)
Logic formula	CDKN2	¬PIK3C& ¬RB1	¬FBXW7&¬PIK3C& ¬RB1	¬EGFR&¬FBXW7& ¬PIK3C& ¬RB1	CDKN2 | KRAS	[CDKN2&¬PIK3C] | [KRAS &¬PIK3C]	BRAF | KRAS | VHL	EZH2 | IDH1 | TSC1 | VHL
TP	16	30	30	30	21	20	14	3
FP	239	482	461	444	286	261	141	15
FN	16	2	2	2	11	12	18	29
TN	346	103	124	141	299	324	444	570
Specificity	0.59	0.18	0.21	0.24	0.51	0.55	0.76	0.97
Precision	0.063	0.059	0.061	0.063	0.068	0.071	0.09	0.17
Recall	0.5	0.94	0.94	0.94	0.66	0.63	0.44	0.094

ID:11 Paclitaxel –> Microtubules

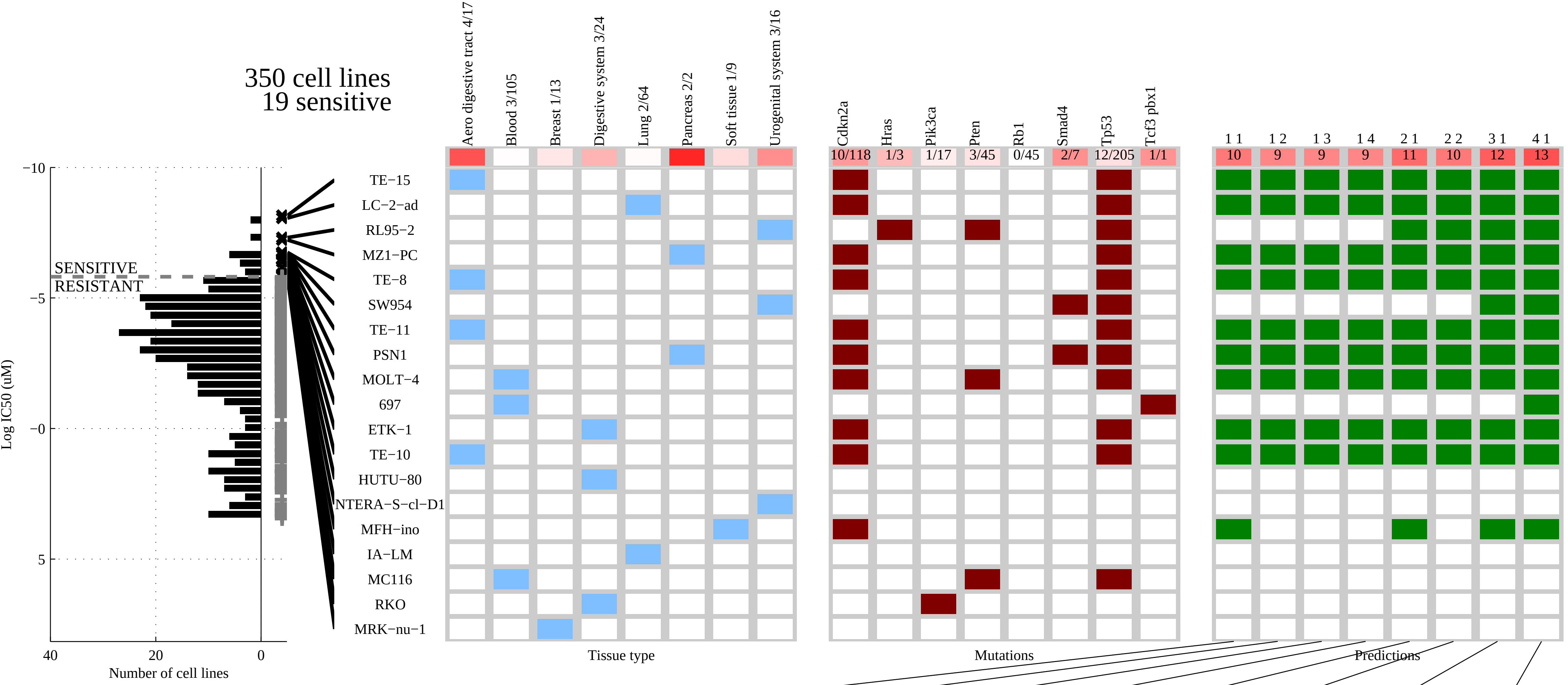

<table>
<tr><td>Model name</td><td>1 1</td><td>1 2</td><td>1 3</td><td>1 4</td><td>2 1</td><td>2 2</td><td>3 1</td><td>4 1</td></tr>
<tr><td>K</td><td>1</td><td>1</td><td>1</td><td>1</td><td>2</td><td>2</td><td>3</td><td>4</td></tr>
<tr><td>M</td><td>1</td><td>2</td><td>3</td><td>4</td><td>1</td><td>2</td><td>1</td><td>1</td></tr>
<tr><td>Logic formula</td><td>CDKN2</td><td>CDKN2& TP53</td><td>CDKN2& ¬RB1 & TP53</td><td>CDKN2&¬PIK3C& ¬RB1 & TP53</td><td>CDKN2 | HRAS</td><td>[HRAS & PTEN] | [CDKN2& TP53]</td><td>CDKN2 | HRAS | SMAD4</td><td>CDKN2 | HRAS | SMAD4 | TCF3</td></tr>
<tr><td>TP</td><td>10</td><td>9</td><td>9</td><td>9</td><td>11</td><td>10</td><td>12</td><td>13</td></tr>
<tr><td>FP</td><td>108</td><td>51</td><td>47</td><td>44</td><td>109</td><td>51</td><td>113</td><td>113</td></tr>
<tr><td>FN</td><td>9</td><td>10</td><td>10</td><td>10</td><td>8</td><td>9</td><td>7</td><td>6</td></tr>
<tr><td>TN</td><td>223</td><td>280</td><td>284</td><td>287</td><td>222</td><td>280</td><td>218</td><td>218</td></tr>
<tr><td>Specificity</td><td>0.67</td><td>0.85</td><td>0.86</td><td>0.87</td><td>0.67</td><td>0.85</td><td>0.66</td><td>0.66</td></tr>
<tr><td>Precision</td><td>0.085</td><td>0.15</td><td>0.16</td><td>0.17</td><td>0.092</td><td>0.16</td><td>0.096</td><td>0.1</td></tr>
<tr><td>Recall</td><td>0.53</td><td>0.47</td><td>0.47</td><td>0.47</td><td>0.58</td><td>0.53</td><td>0.63</td><td>0.68</td></tr>
</table>

ID:1129 PF−4708671 −> RPS6KB1 (p70S6KA)

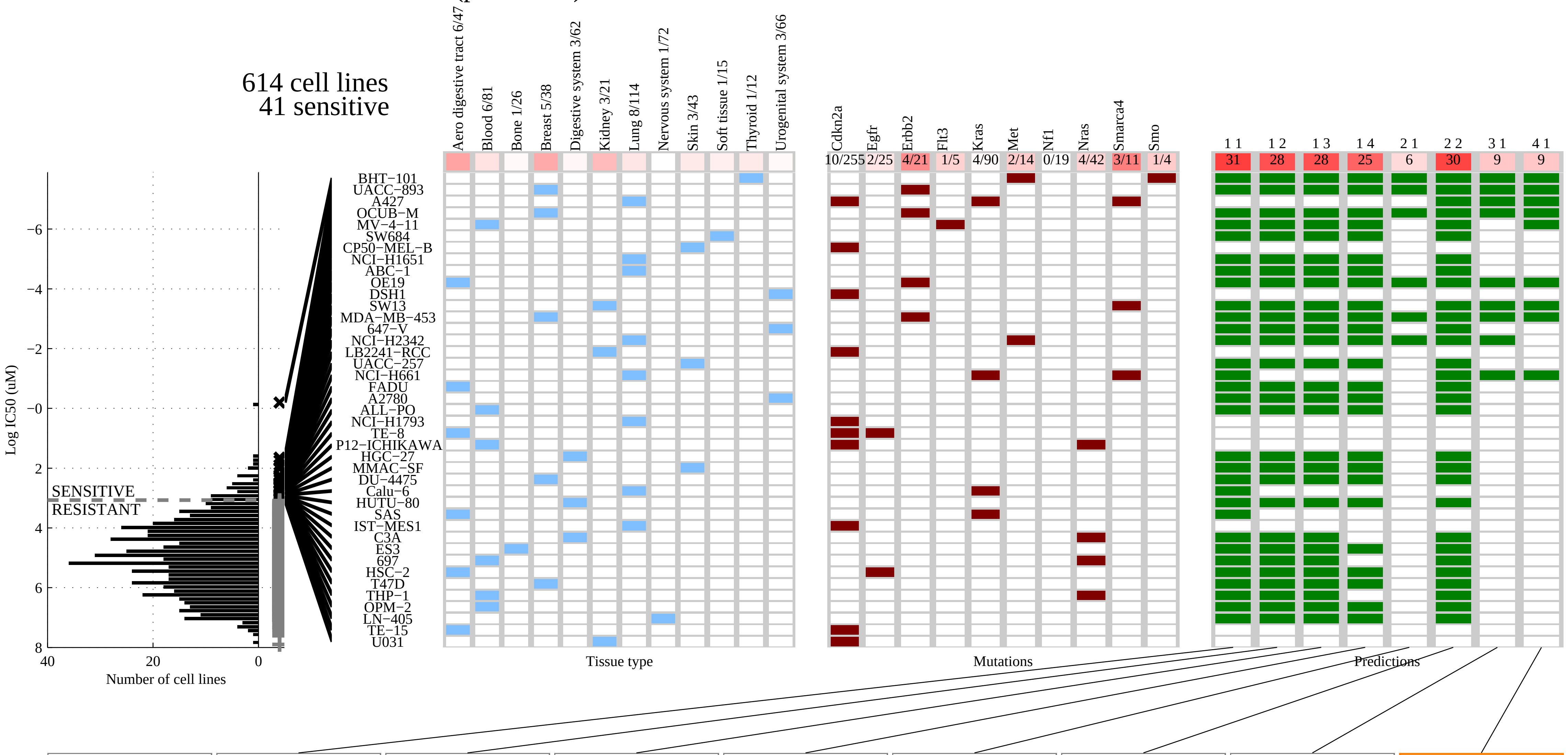

Model name (K, M)	1 1	1 2	1 3	1 4	2 1	2 2	3 1	4 1
K / M	1 / 1	1 / 2	1 / 3	1 / 4	2 / 1	2 / 2	3 / 1	4 / 1
Logic formula	¬CDKN2	¬CDKN2&¬KRAS	¬CDKN2&¬KRAS& ¬NF1	¬CDKN2&¬KRAS& ¬NF1 &¬NRAS	ERBB2 | MET	[¬CDKN2&¬KRAS] | [¬EGFR&SMARC]	ERBB2 | MET | SMARC	ERBB2 | FLT3 | SMARC | SMO
TP	31	28	28	25	6	30	9	9
FP	328	279	266	251	27	281	34	30
FN	10	13	13	16	35	11	32	32
TN	245	294	307	322	546	292	539	543
Specificity	0.43	0.51	0.54	0.56	0.95	0.51	0.94	0.95
Precision	0.086	0.091	0.095	0.091	0.18	0.096	0.21	0.23
Recall	0.76	0.68	0.68	0.61	0.15	0.73	0.22	0.22

ID:1133 JNJ−26854165 −> MDM2

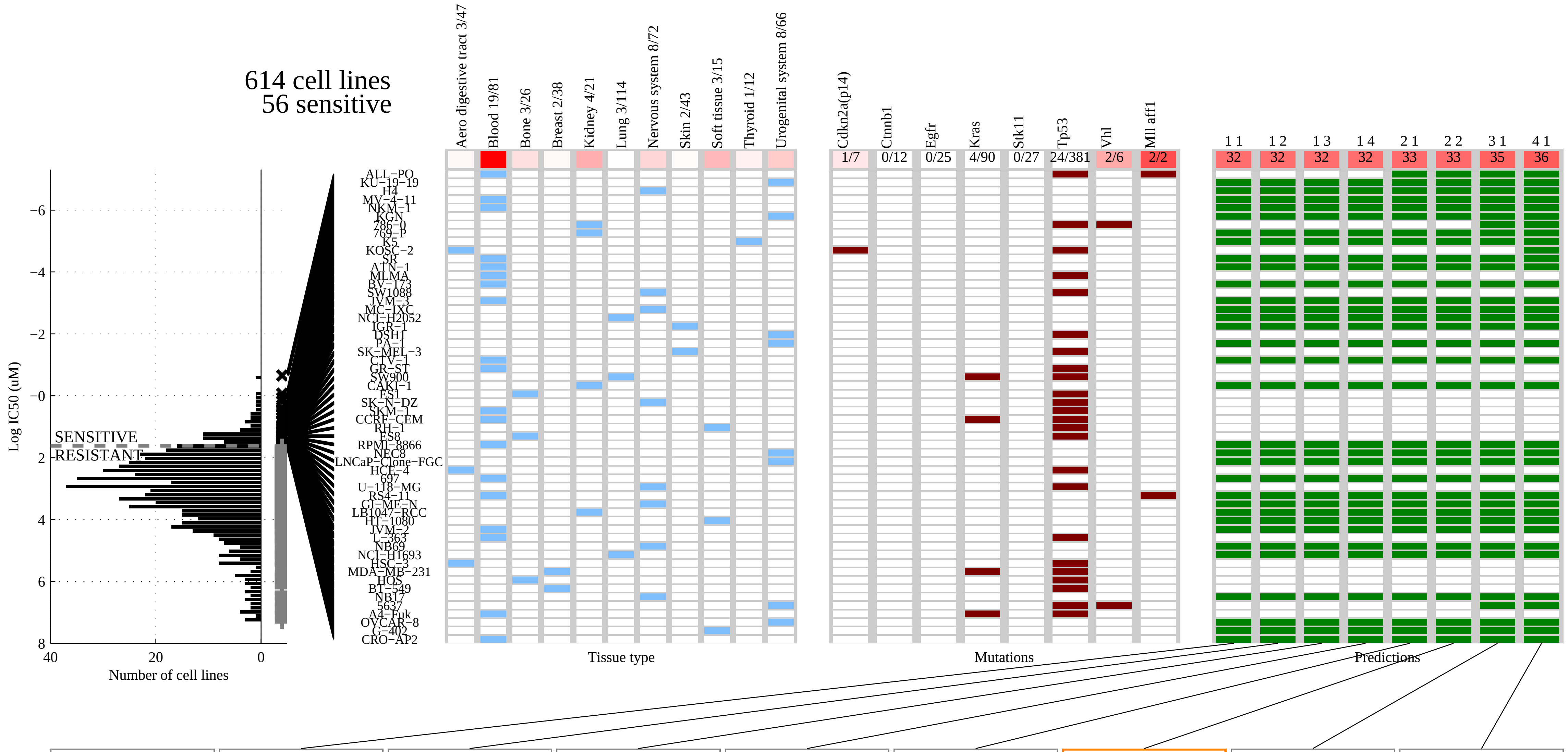

Model name	1 1	1 2	1 3	1 4	2 1	2 2	3 1	4 1
K	1	1	1	1	2	2	3	4
M	1	2	3	4	1	2	1	1
Logic formula	¬TP53	¬KRAS& ¬TP53	¬EGFR&¬KRAS& ¬TP53	¬EGFR&¬KRAS& ¬STK11& ¬TP53	¬TP53 | MLL A	[¬CTNNB1& MLL A] | [¬KRAS& ¬TP53]	¬TP53 | VHL | MLL A	CDKN2 | ¬TP53 | VHL | MLL A
TP	32	32	32	32	33	33	35	36
FP	201	174	167	160	201	174	202	205
FN	24	24	24	24	23	23	21	20
TN	357	384	391	398	357	384	356	353
Specificity	0.64	0.69	0.7	0.71	0.64	0.69	0.64	0.63
Precision	0.14	0.16	0.16	0.17	0.14	0.16	0.15	0.15
Recall	0.57	0.57	0.57	0.57	0.59	0.59	0.63	0.64

ID:1149 TW 37 –> BCL2, BCL2L1

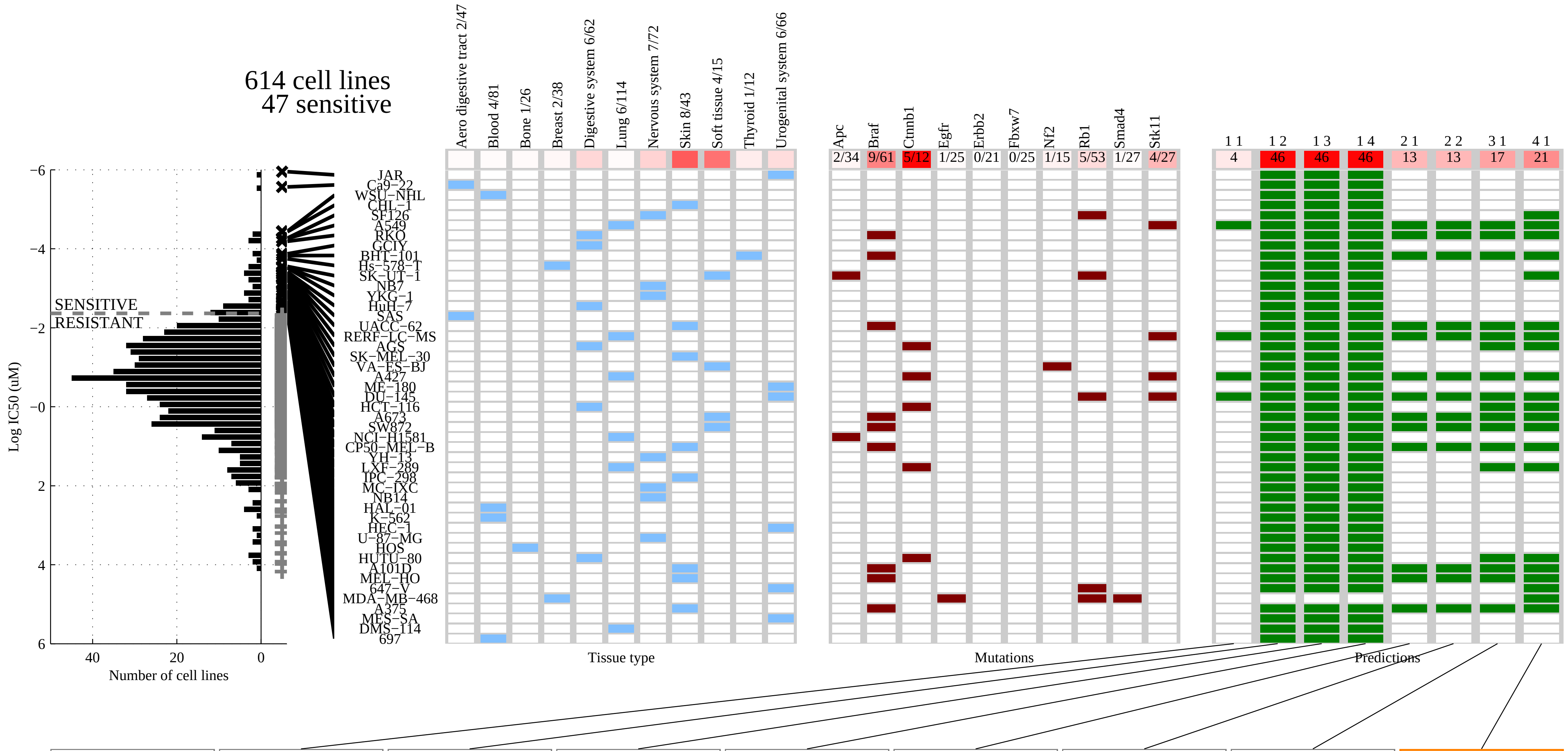

Model name (K, M)	1 1 (1, 1)	1 2 (1, 2)	1 3 (1, 3)	1 4 (1, 4)	2 1 (2, 1)	2 2 (2, 2)	3 1 (3, 1)	4 1 (4, 1)
Logic formula	STK11	¬ERBB2&¬SMAD4	¬ERBB2&¬FBXW7& ¬SMAD4	¬EGFR&¬ERBB2& ¬FBXW7&¬SMAD4	BRAF | STK11	[¬APC & BRAF] | [¬NF2 & STK11]	BRAF | CTNNB | STK11	BRAF | CTNNB | RB1 | STK11
TP	4	46	46	46	13	13	17	21
FP	23	521	496	473	70	63	76	121
FN	43	1	1	1	34	34	30	26
TN	544	46	71	94	497	504	491	446
Specificity	0.96	0.081	0.13	0.17	0.88	0.89	0.87	0.79
Precision	0.15	0.081	0.085	0.089	0.16	0.17	0.18	0.15
Recall	0.085	0.98	0.98	0.98	0.28	0.28	0.36	0.45

ID:1170 CCT018159 −> HSP90

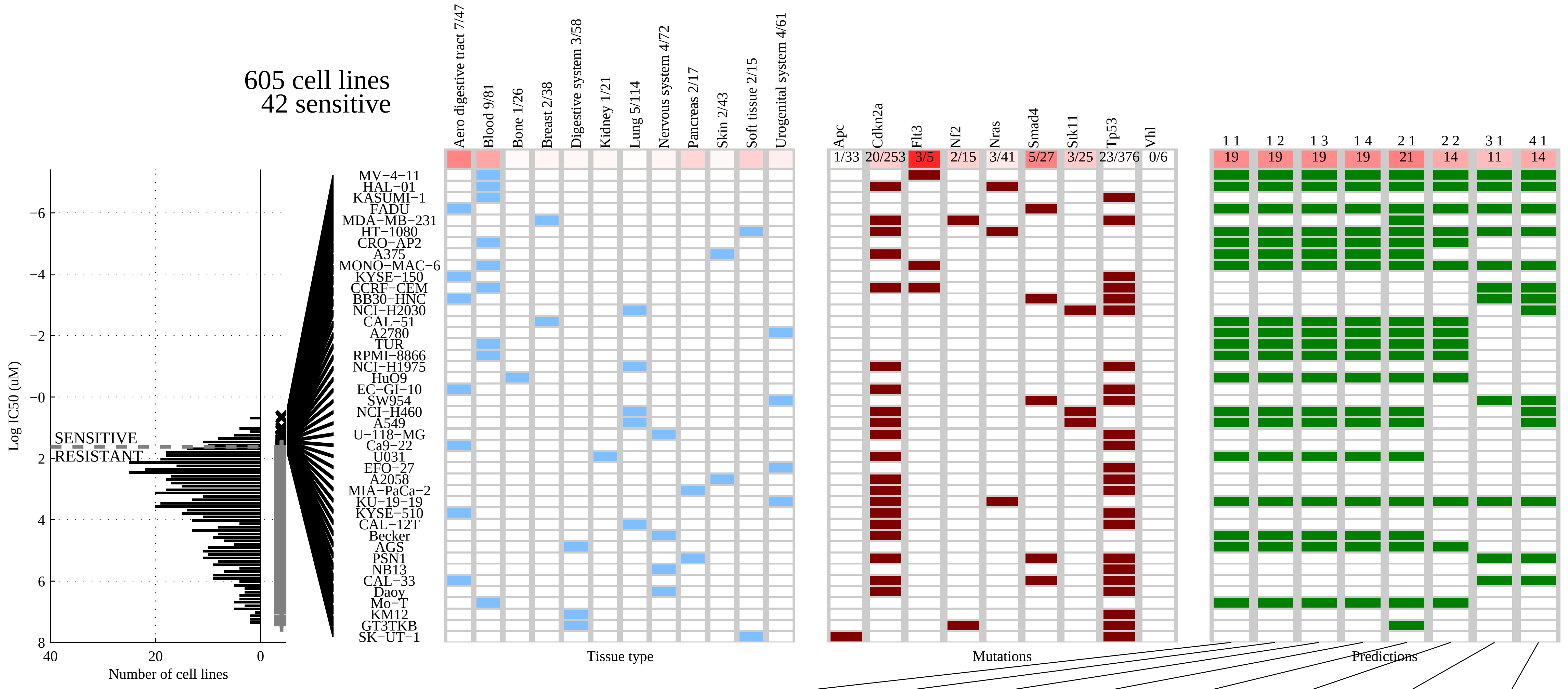

Model name	1 1	1 2	1 3	1 4	2 1	2 2	3 1	4 1
K / M	1 / 1	1 / 2	1 / 3	1 / 4	2 / 1	2 / 2	3 / 1	4 / 1
Logic formula	¬TP53	¬APC & ¬TP53	¬APC & ¬NF2 & ¬TP53	¬APC & ¬NF2 & ¬TP53 & ¬VHL	NF2 | ¬TP53	[¬CDKN2A & ¬TP53] | [NRAS & ¬TP53]	FLT3 | NRAS | SMAD4	FLT3 | NRAS | SMAD4 | STK11
TP / FP	19 / 210	19 / 201	19 / 193	19 / 190	21 / 215	14 / 118	11 / 61	14 / 82
FN / TN	23 / 353	23 / 362	23 / 370	23 / 373	21 / 348	28 / 445	31 / 502	28 / 481
Specificity	0.63	0.64	0.66	0.66	0.62	0.79	0.89	0.85
Precision	0.083	0.086	0.09	0.091	0.089	0.11	0.15	0.15
Recall	0.45	0.45	0.45	0.45	0.5	0.33	0.26	0.33

ID:1175 AG−014699 −> PARP1, PARP2

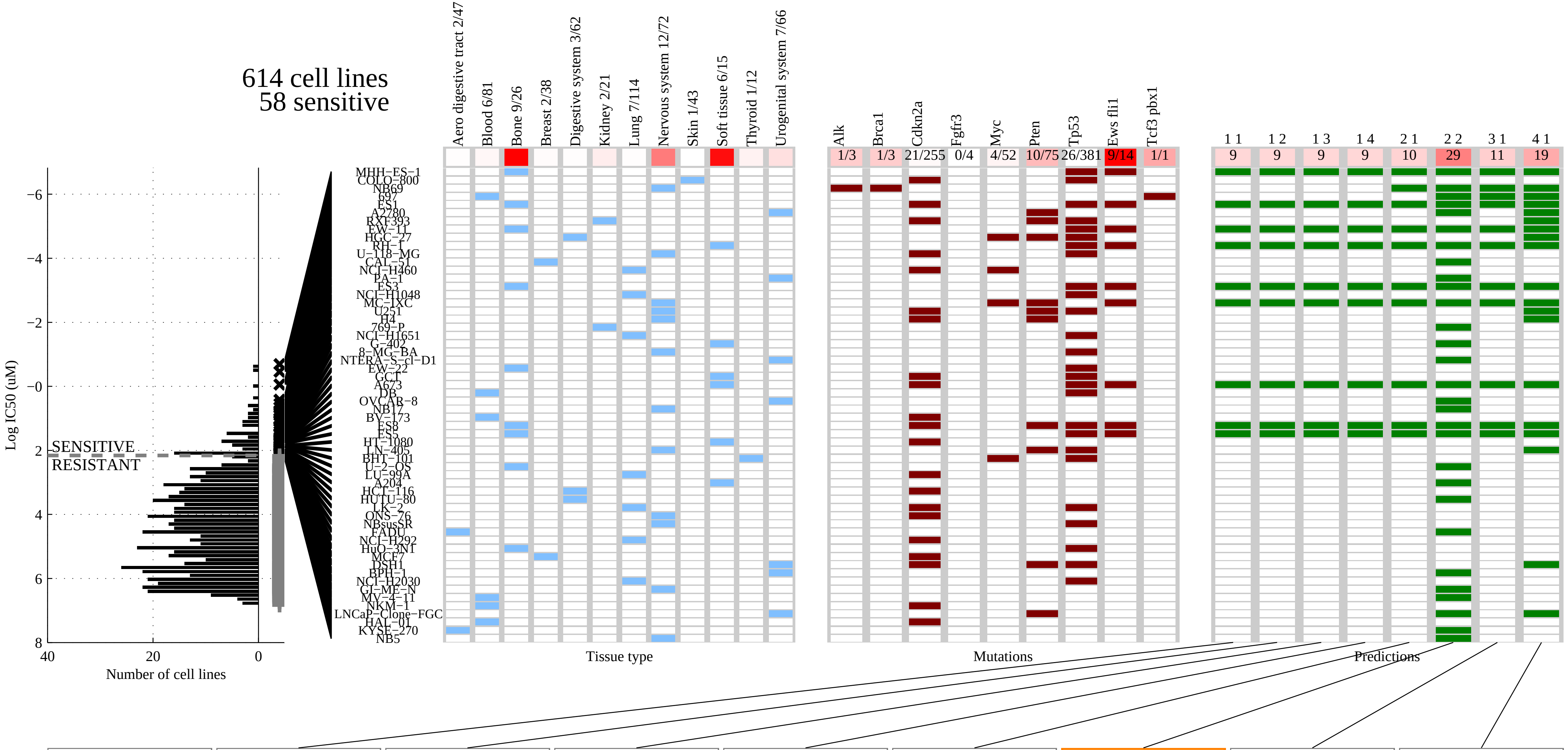

Model name (K / M)	1 1 (1 / 1)	1 2 (1 / 2)	1 3 (1 / 3)	1 4 (1 / 4)	2 1 (2 / 1)	2 2 (2 / 2)	3 1 (3 / 1)	4 1 (4 / 1)
Logic formula	**EWS F**	**EWS F &**	**EWS F & &**	**¬FGFR3 & EWS F & &**	**ALK | EWS F**	**[¬MYC & EWS F] | [¬CDKN2 & ¬TP53]**	**ALK | EWS F | TCF3**	**BRCA1 | PTEN | EWS F | TCF3**
TP / FP	9 / 5	9 / 5	9 / 5	9 / 5	10 / 7	29 / 106	11 / 7	19 / 71
FN / TN	49 / 551	49 / 551	49 / 551	49 / 551	48 / 549	29 / 450	47 / 549	39 / 485
Specificity	0.99	0.99	0.99	0.99	0.99	0.81	0.99	0.87
Precision	0.64	0.64	0.64	0.64	0.59	0.21	0.61	0.21
Recall	0.16	0.16	0.16	0.16	0.17	0.5	0.19	0.33

ID:119 Lapatinib −> ERBB2, EGFR

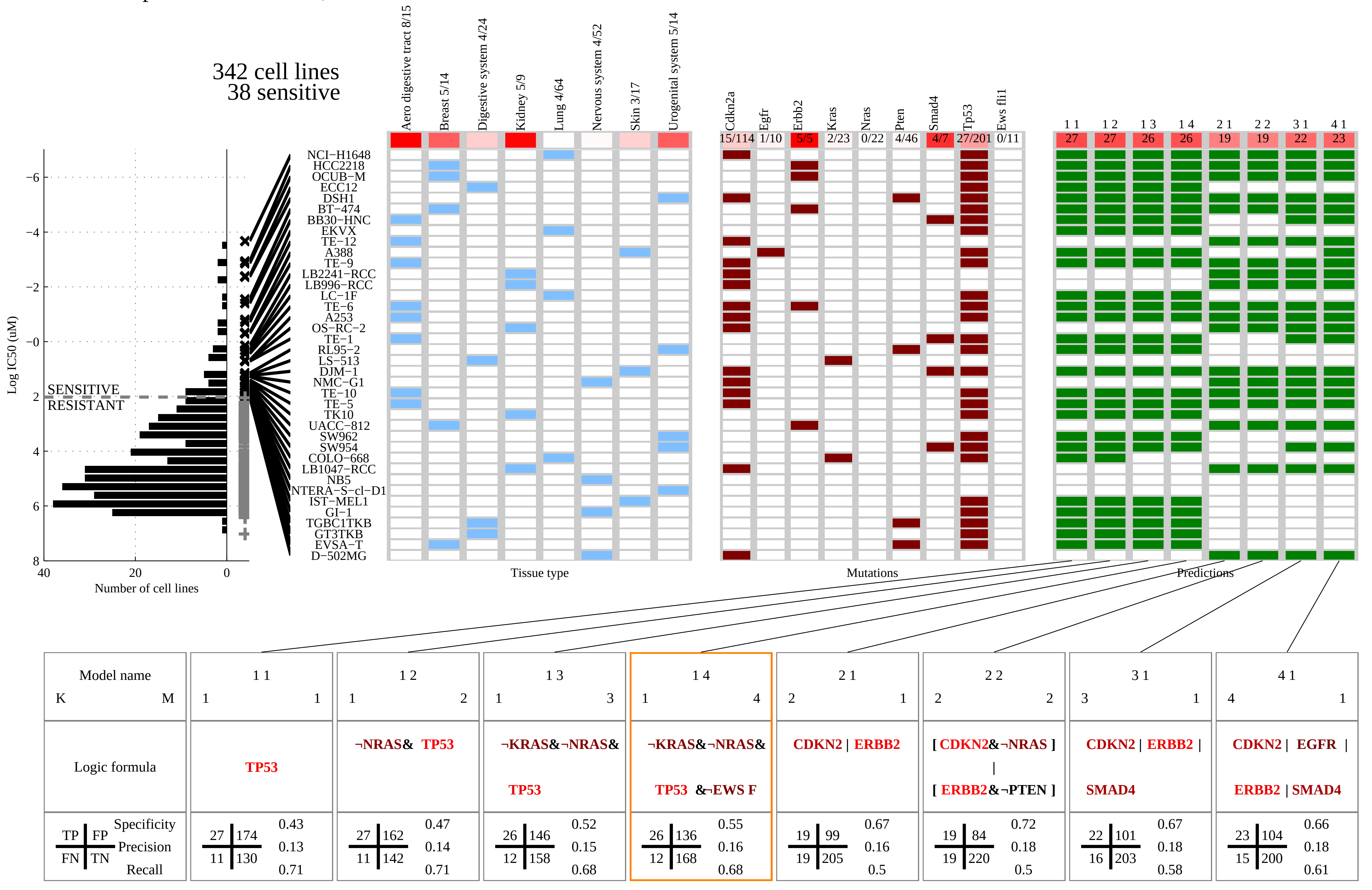

ID:127 GSK269962A –> ROCK1, ROCK2

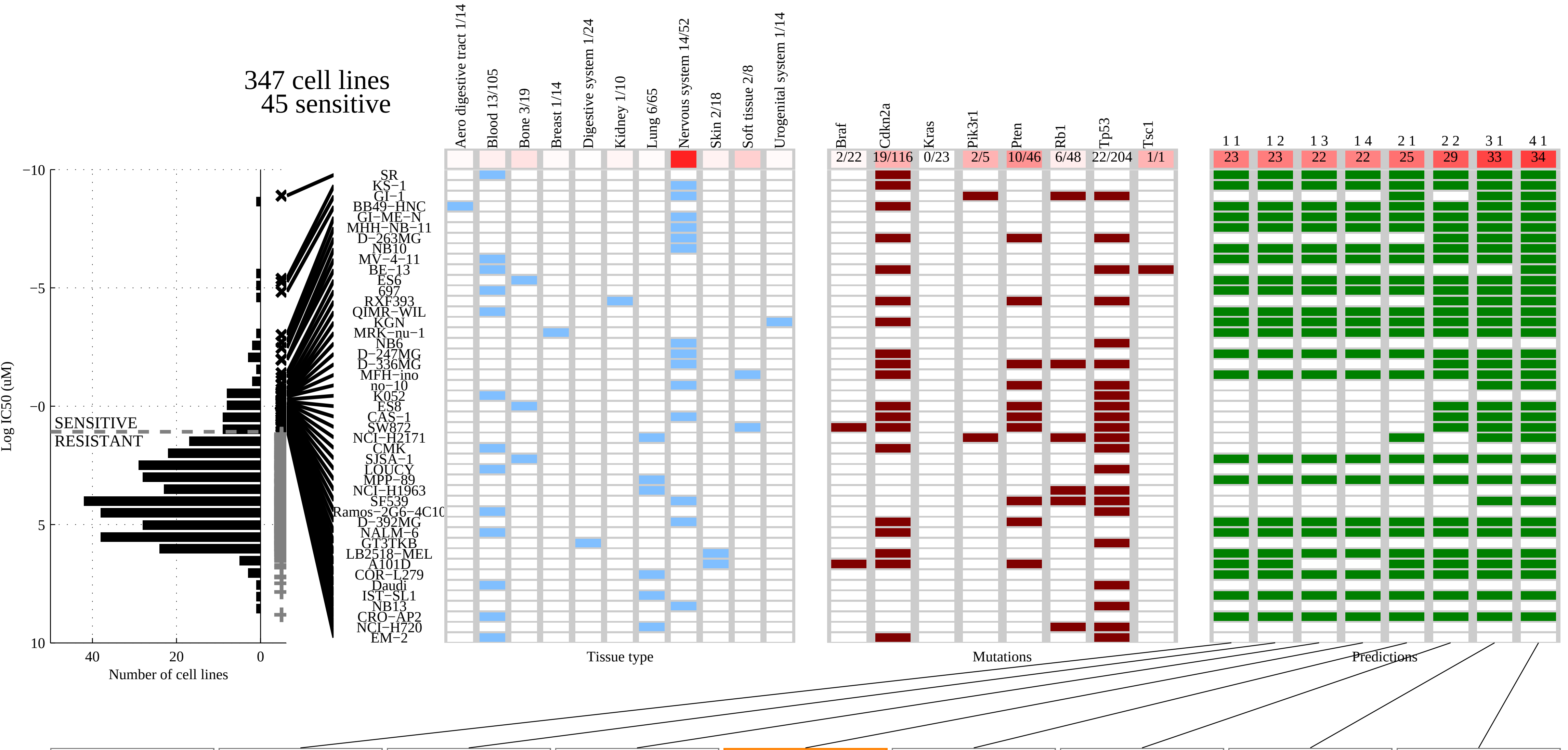

Model name (K, M)	1 1 (K 1, M 1)	1 2 (K 1, M 2)	1 3 (K 1, M 3)	1 4 (K 1, M 4)	2 1 (K 2, M 1)	2 2 (K 2, M 2)	3 1 (K 3, M 1)	4 1 (K 4, M 1)
Logic formula	¬TP53	¬RB1 & ¬TP53	¬BRAF& ¬RB1 & ¬TP53	¬BRAF&¬KRAS& ¬RB1 & ¬TP53	PIK3R | ¬TP53	[CDKN2& PTEN] | [¬BRAF& ¬TP53]	PIK3R | PTEN | ¬TP53	PIK3R | PTEN | ¬TP53 | TSC1
TP	23	23	22	22	25	29	33	34
FP	120	108	93	88	120	113	148	148
FN	22	22	23	23	20	16	12	11
TN	182	194	209	214	182	189	154	154
Specificity	0.6	0.64	0.69	0.71	0.6	0.63	0.51	0.51
Precision	0.16	0.18	0.19	0.2	0.17	0.2	0.18	0.19
Recall	0.51	0.51	0.49	0.49	0.56	0.64	0.73	0.76

ID:130 T−Oligos −> Telomerase

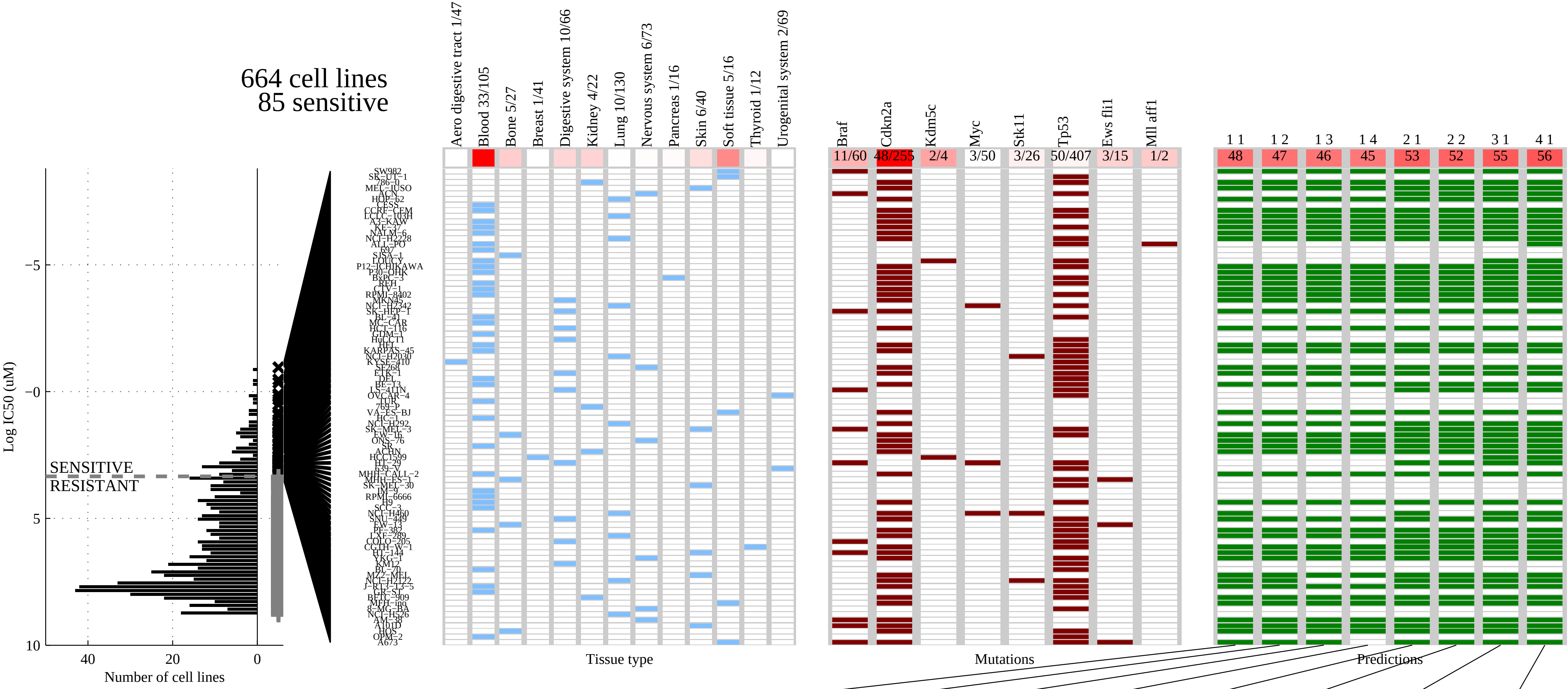

Model name (K, M)	1 1 (1, 1)	1 2 (1, 2)	1 3 (1, 3)	1 4 (1, 4)	2 1 (2, 1)	2 2 (2, 2)	3 1 (3, 1)	4 1 (4, 1)
Logic formula	CDKN2	CDKN2& ¬MYC	CDKN2& ¬MYC & ¬STK11	CDKN2& ¬MYC & ¬STK11&¬EWS F	BRAF | CDKN2	[BRAF & TP53] | [CDKN2& ¬MYC]	BRAF | CDKN2 | KDM5C	BRAF | CDKN2 | KDM5C | MLL A
TP	48	47	46	45	53	52	55	56
FP	207	190	178	175	225	201	226	226
FN	37	38	39	40	32	33	30	29
TN	372	389	401	404	354	378	353	353
Specificity	0.64	0.67	0.69	0.7	0.61	0.65	0.61	0.61
Precision	0.19	0.2	0.21	0.2	0.19	0.21	0.2	0.2
Recall	0.56	0.55	0.54	0.53	0.62	0.61	0.65	0.66

ID:132 J−124 −> PI3Ka

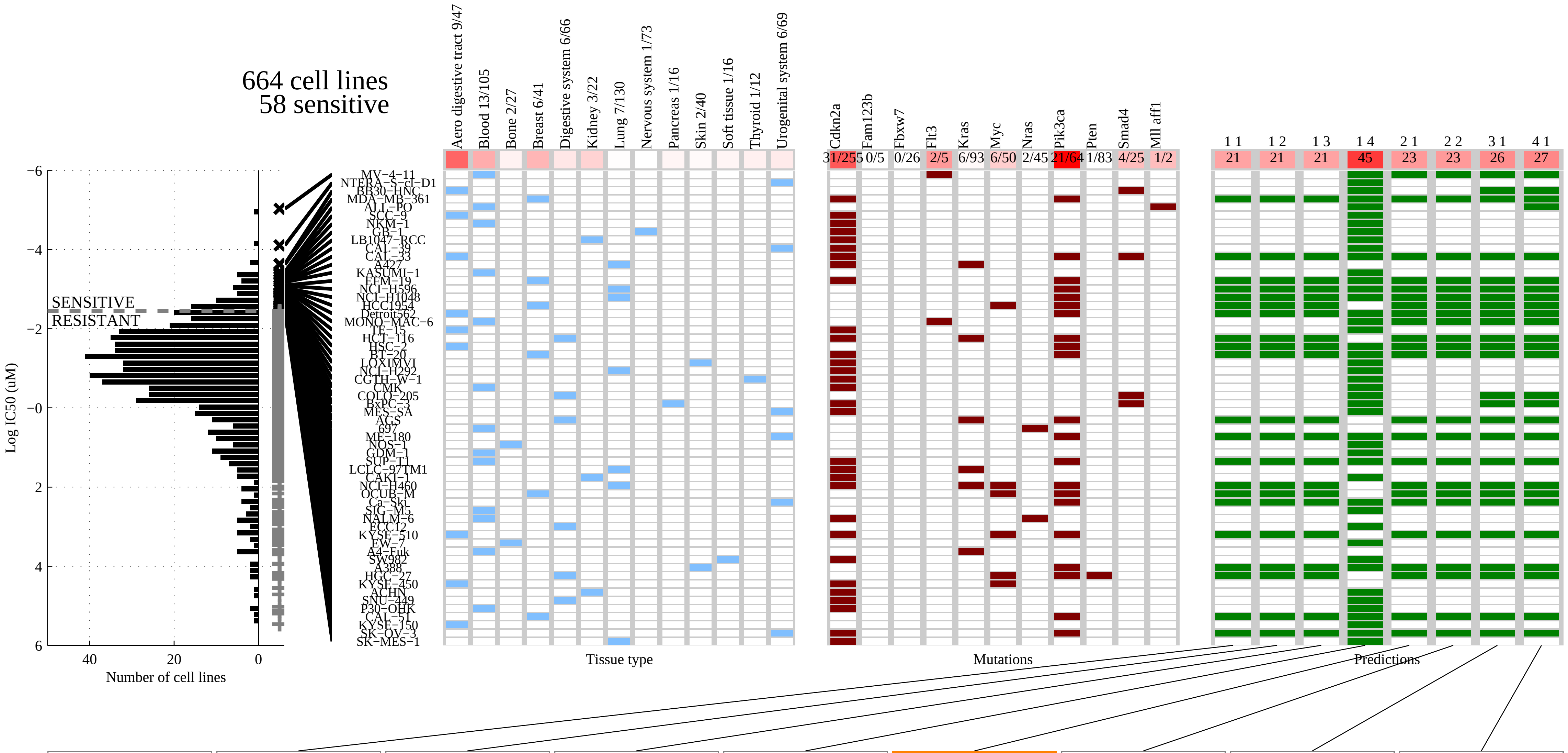

Model name (K, M)	1 1 (1, 1)	1 2 (1, 2)	1 3 (1, 3)	1 4 (1, 4)	2 1 (2, 1)	2 2 (2, 2)	3 1 (3, 1)	4 1 (4, 1)
Logic formula	PIK3C	¬FBXW7& PIK3C	¬FAM12&¬FBXW7& PIK3C	¬KRAS& ¬MYC & ¬NRAS&¬PTEN	FLT3 | PIK3C	[¬FBXW7& PIK3C] | [¬CDKN2& FLT3]	FLT3 | PIK3C | SMAD4	FLT3 | PIK3C | SMAD4 | MLL A
TP / FP	21 / 43	21 / 39	21 / 37	45 / 371	23 / 46	23 / 39	26 / 65	27 / 66
FN / TN	37 / 563	37 / 567	37 / 569	13 / 235	35 / 560	35 / 567	32 / 541	31 / 540
Specificity	0.93	0.94	0.94	0.39	0.92	0.94	0.89	0.89
Precision	0.33	0.35	0.36	0.11	0.33	0.37	0.29	0.29
Recall	0.36	0.36	0.36	0.78	0.4	0.4	0.45	0.47

ID:133 Doxorubicin –> DNA intercalating

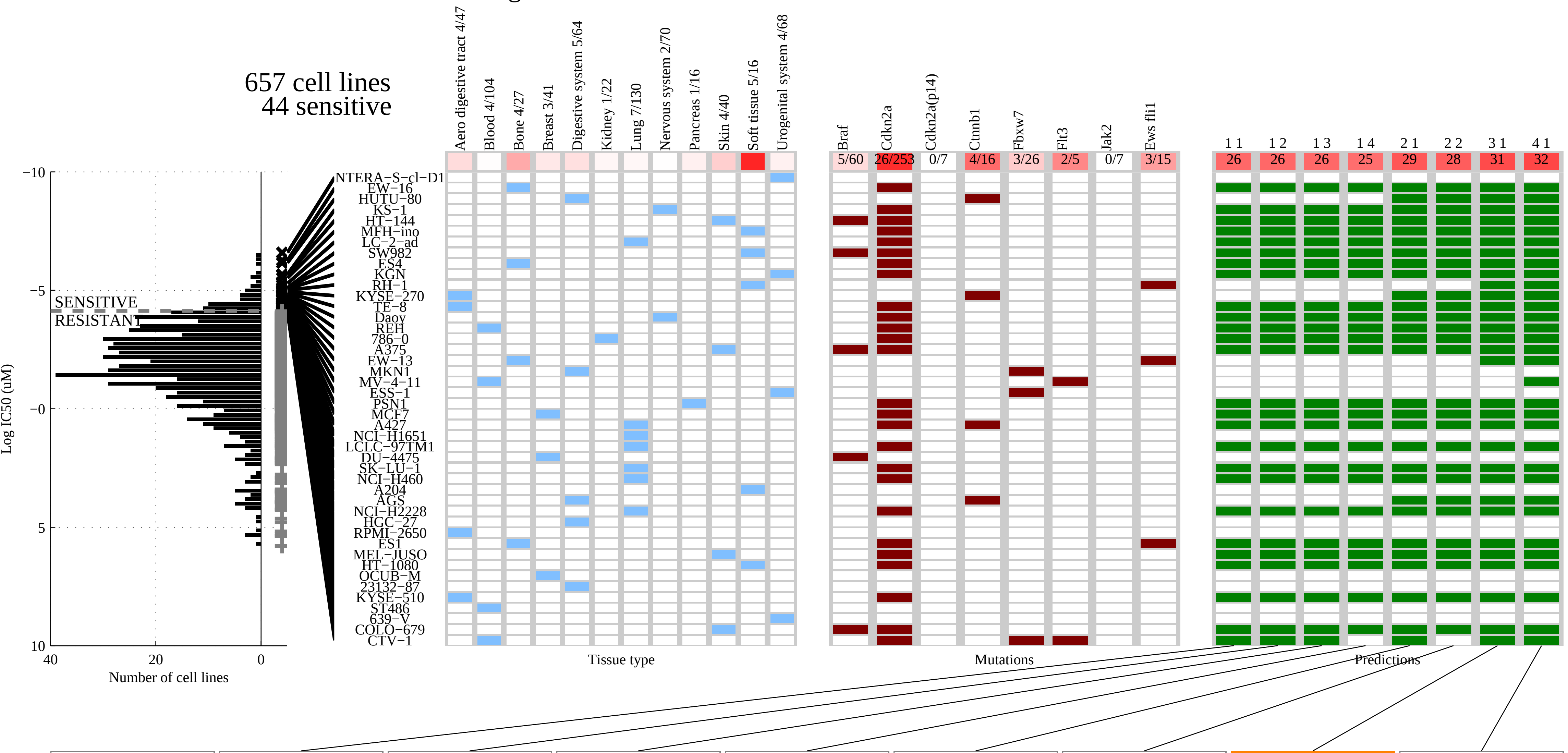

Model name	1 1	1 2	1 3	1 4	2 1	2 2	3 1	4 1
K / M	1 / 1	1 / 2	1 / 3	1 / 4	2 / 1	2 / 2	3 / 1	4 / 1
Logic formula	CDKN2	CDKN2&¬CDKN2	CDKN2&¬CDKN2& ¬JAK2	CDKN2&¬CDKN2& ¬FBXW7& ¬JAK2	CDKN2 | CTNNB	[¬BRAF&CTNNB] | [CDKN2&¬FBXW7]	CDKN2 | CTNNB | EWS F	CDKN2 | CTNNB | FLT3 | EWS F
TP / FP	26 / 227	26 / 221	26 / 215	25 / 207	29 / 235	28 / 224	31 / 243	32 / 244
FN / TN	18 / 386	18 / 392	18 / 398	19 / 406	15 / 378	16 / 389	13 / 370	12 / 369
Specificity	0.63	0.64	0.65	0.66	0.62	0.63	0.6	0.6
Precision	0.1	0.11	0.11	0.11	0.11	0.11	0.11	0.12
Recall	0.59	0.59	0.59	0.57	0.66	0.64	0.7	0.73

ID:134 Etoposide −> TOP2

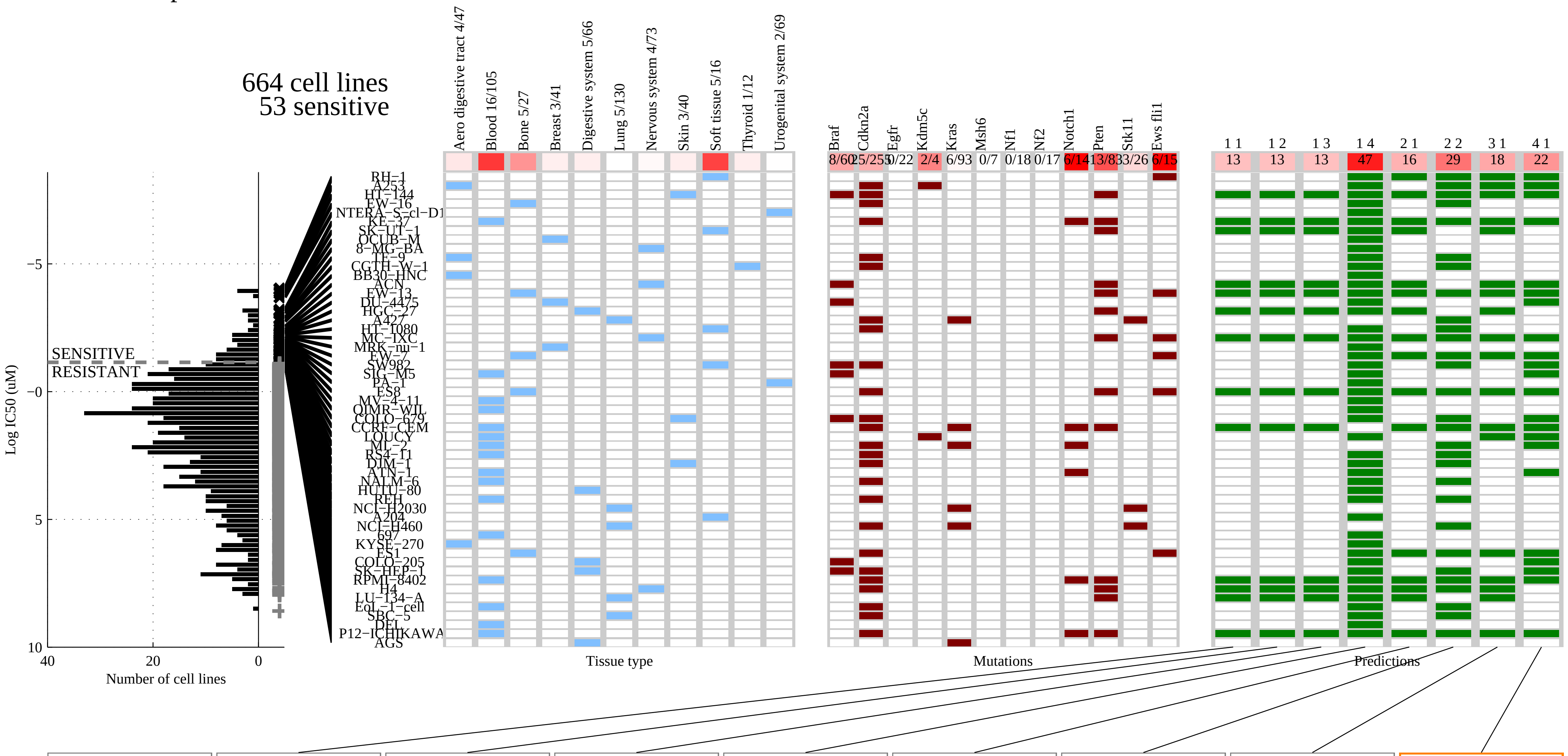

Model name	1 1	1 2	1 3	1 4	2 1	2 2	3 1	4 1
K	1	1	1	1	2	2	3	4
M	1	2	3	4	1	2	1	1
Logic formula	PTEN	¬NF1 & PTEN	¬MSH6 & ¬NF1 & PTEN	¬EGFR & ¬KRAS & ¬NF1 & ¬STK11	PTEN | EWS F	[¬KDM5C & EWS F] | [CDKN2 & ¬NF2]	KDM5C | PTEN | EWS F	BRAF | KDM5C | NOTCH | EWS F
TP	13	13	13	47	16	29	18	22
FP	70	66	63	471	79	223	81	70
FN	40	40	40	6	37	24	35	31
TN	541	545	548	140	532	388	530	541
Specificity	0.89	0.89	0.9	0.23	0.87	0.64	0.87	0.89
Precision	0.16	0.16	0.17	0.091	0.17	0.12	0.18	0.24
Recall	0.25	0.25	0.25	0.89	0.3	0.55	0.34	0.42

ID:135 Gemcitabine –> DNA replication

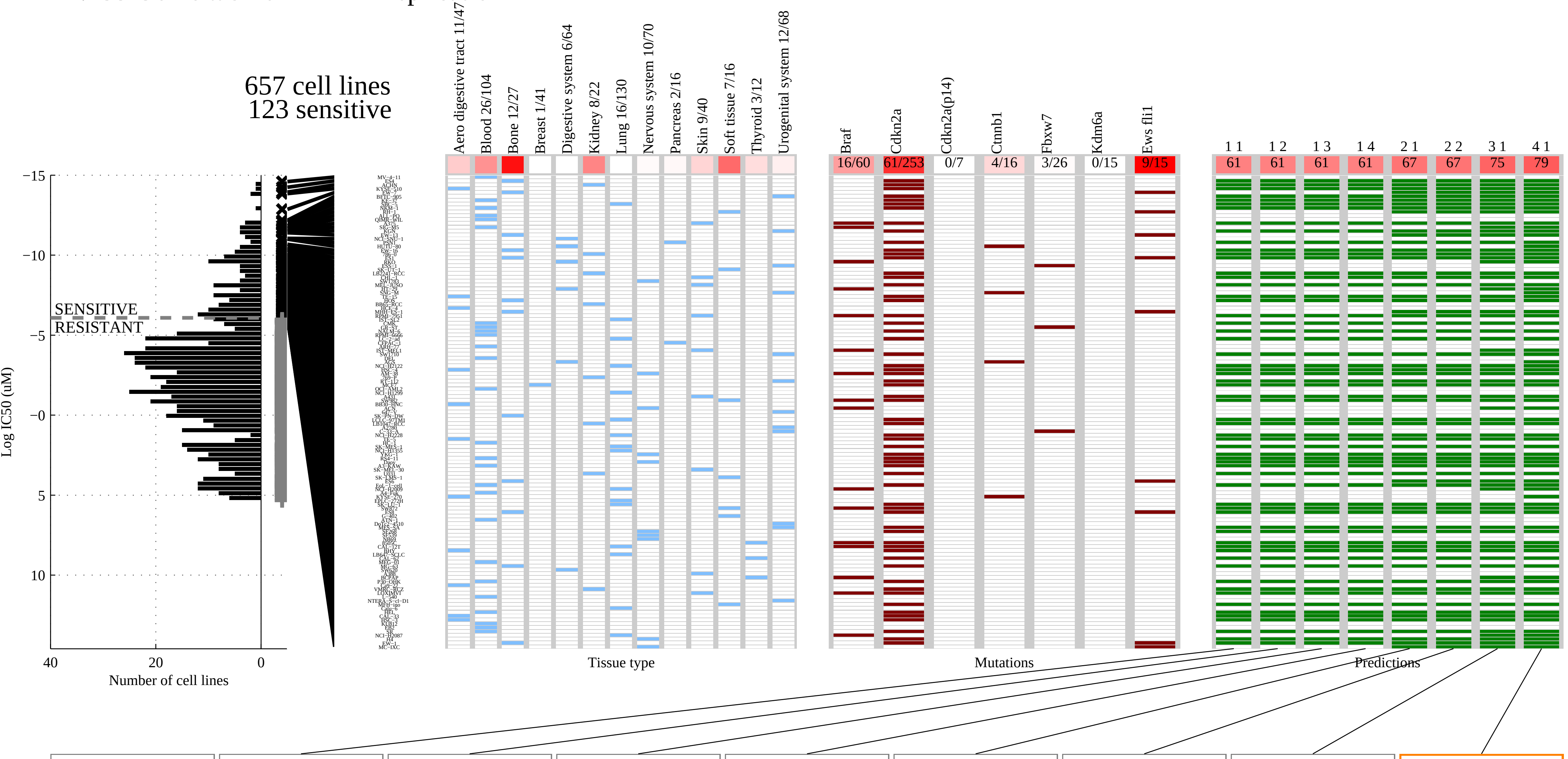

Model name<br>K M	1 1<br>1 1	1 2<br>1 2	1 3<br>1 3	1 4<br>1 4	2 1<br>2 1	2 2<br>2 2	3 1<br>3 1	4 1<br>4 1
Logic formula	**CDKN2**	**CDKN2&¬FBXW7**	**CDKN2&¬CDKN2&**<br>**¬FBXW7**	**CDKN2&¬CDKN2&**<br>**¬FBXW7&KDM6A**	**CDKN2 | EWS F**	**[EWS F &]**<br>**|**<br>**[CDKN2&¬FBXW7]**	**BRAF | CDKN2 |**<br>**EWS F**	**BRAF | CDKN2 |**<br>**CTNNB | EWS F**
TP FP / FN TN	61 192 / 62 342	61 182 / 62 352	61 177 / 62 357	61 171 / 62 363	67 196 / 56 338	67 186 / 56 348	75 211 / 48 323	79 216 / 44 318
Specificity	0.64	0.66	0.67	0.68	0.63	0.65	0.6	0.6
Precision	0.24	0.25	0.26	0.26	0.25	0.26	0.26	0.27
Recall	0.5	0.5	0.5	0.5	0.54	0.54	0.61	0.64

ID:136 Mitomycin C –> DNA crosslinker

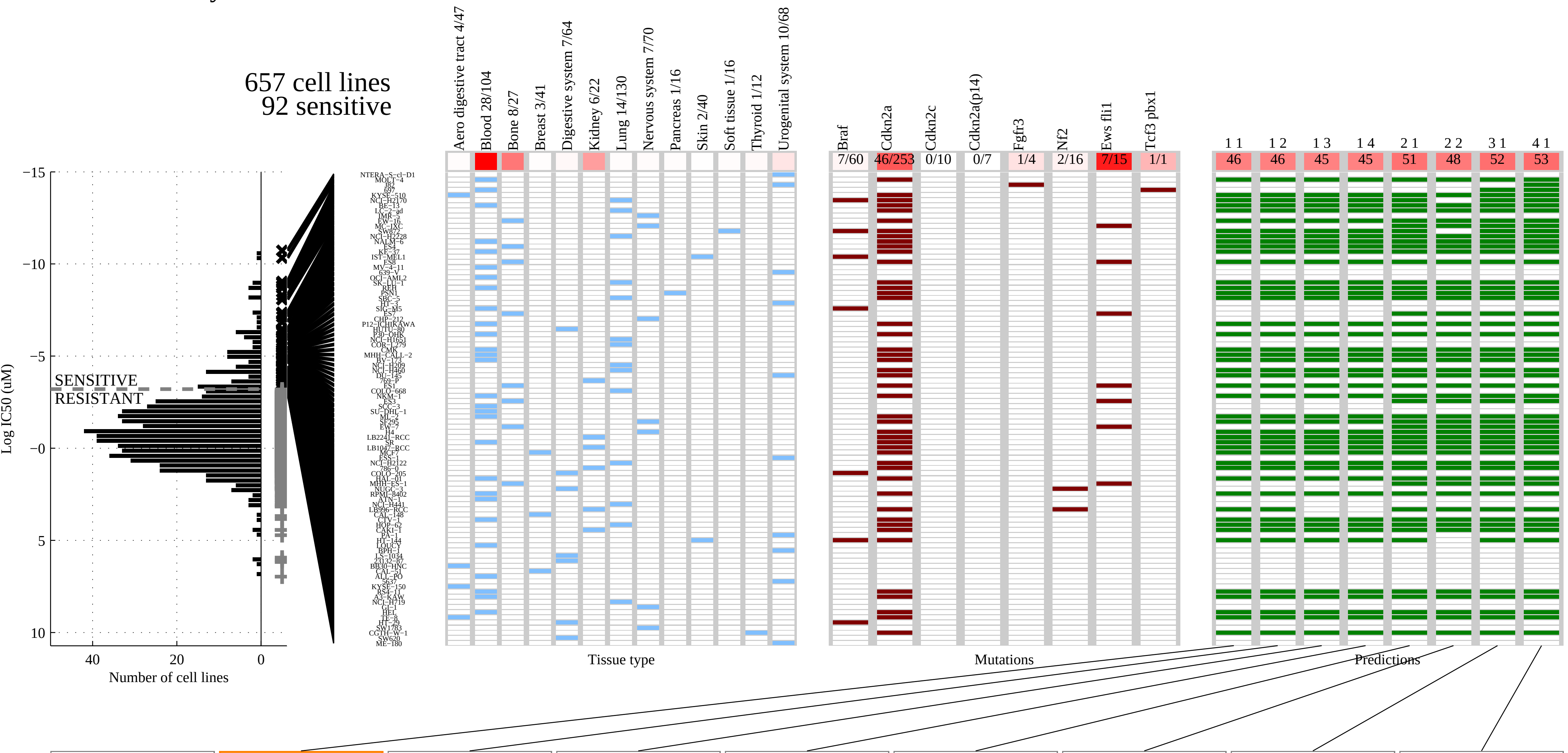

Model name	1 1	1 2	1 3	1 4	2 1	2 2	3 1	4 1
K / M	1 / 1	1 / 2	1 / 3	1 / 4	2 / 1	2 / 2	3 / 1	4 / 1
Logic formula	**CDKN2**	**CDKN2&¬CDKN2**	**CDKN2&¬CDKN2& ¬NF2**	**CDKN2&¬CDKN2& ¬CDKN2& ¬NF2**	**CDKN2 | EWS F**	**[¬CDKN2& EWS F] | [¬BRAF&CDKN2]**	**CDKN2 | EWS F | TCF3**	**CDKN2 | FGFR3 | EWS F | TCF3**
TP FP	46 207	46 201	45 189	45 183	51 212	48 178	52 212	53 214
FN TN	46 358	46 364	47 376	47 382	41 353	44 387	40 353	39 351
Specificity	0.63	0.64	0.67	0.68	0.62	0.68	0.62	0.62
Precision	0.18	0.19	0.19	0.2	0.19	0.21	0.2	0.2
Recall	0.5	0.5	0.49	0.49	0.55	0.52	0.57	0.58

ID:140 Vinorelbine −> Microtubules

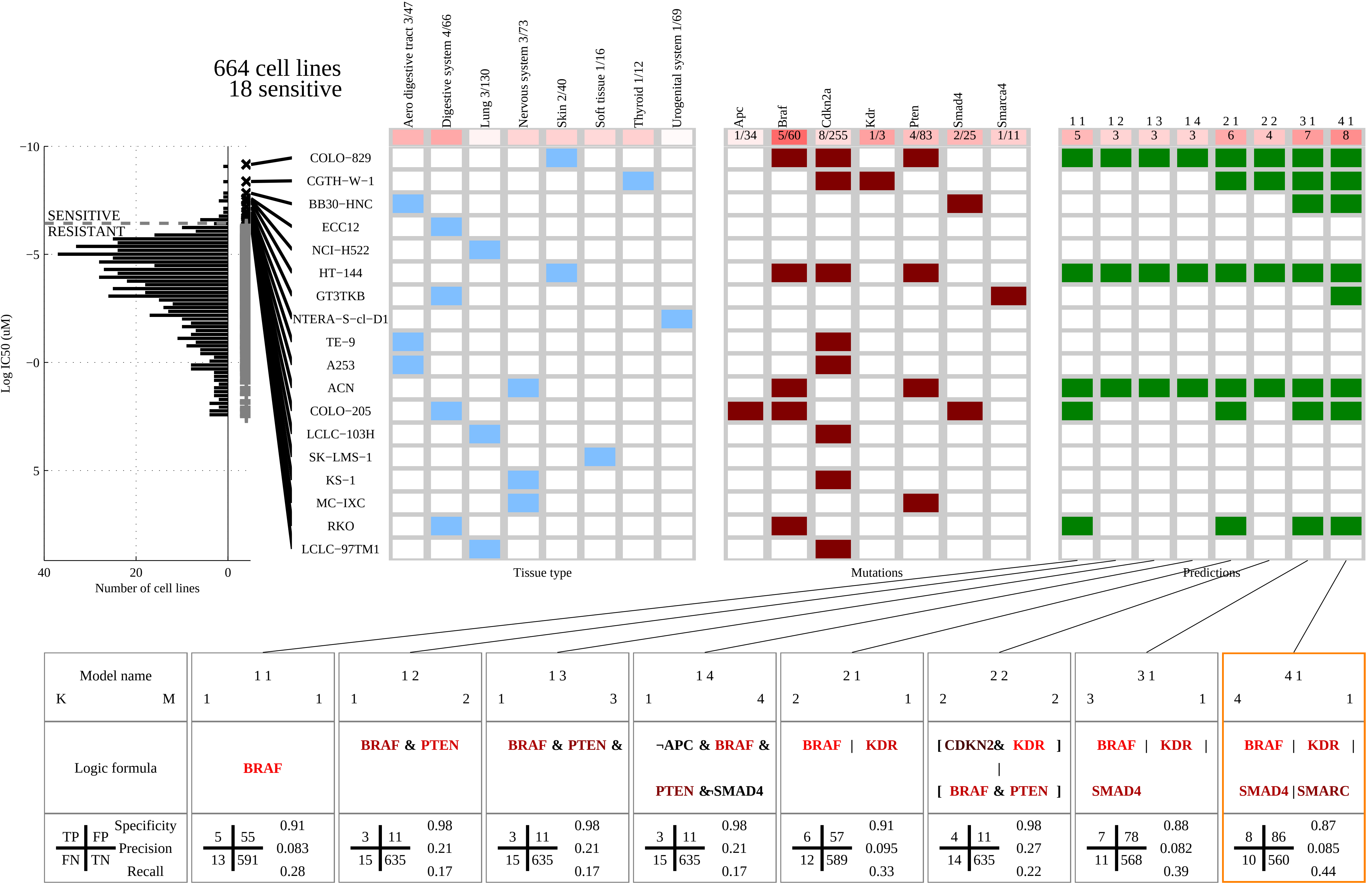

ID:146 A−769662 −> AMPK

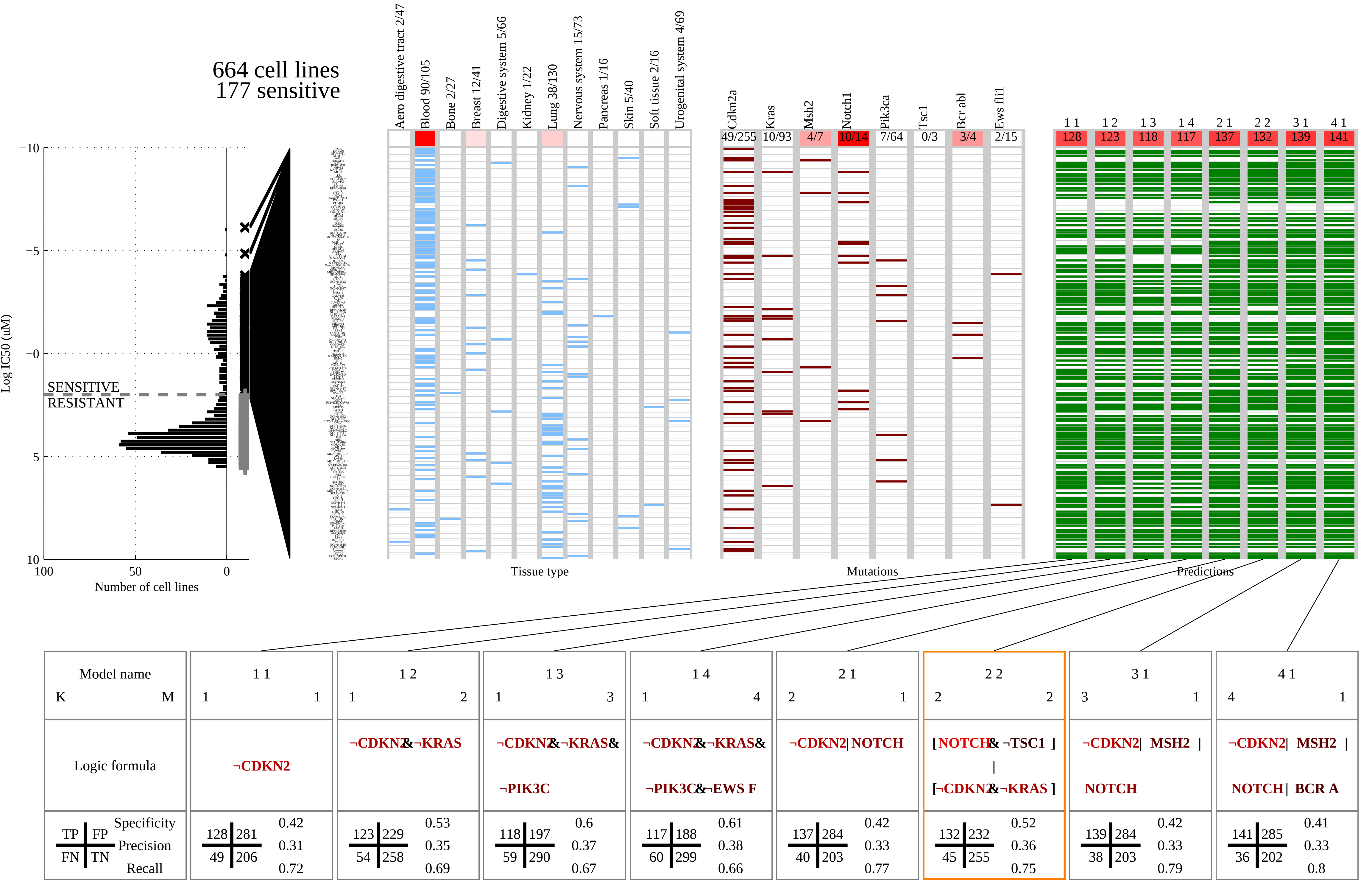

ID:147 NSC−87877 -> PTPN6 (SHP−1), PTPN11 (SHP−2)

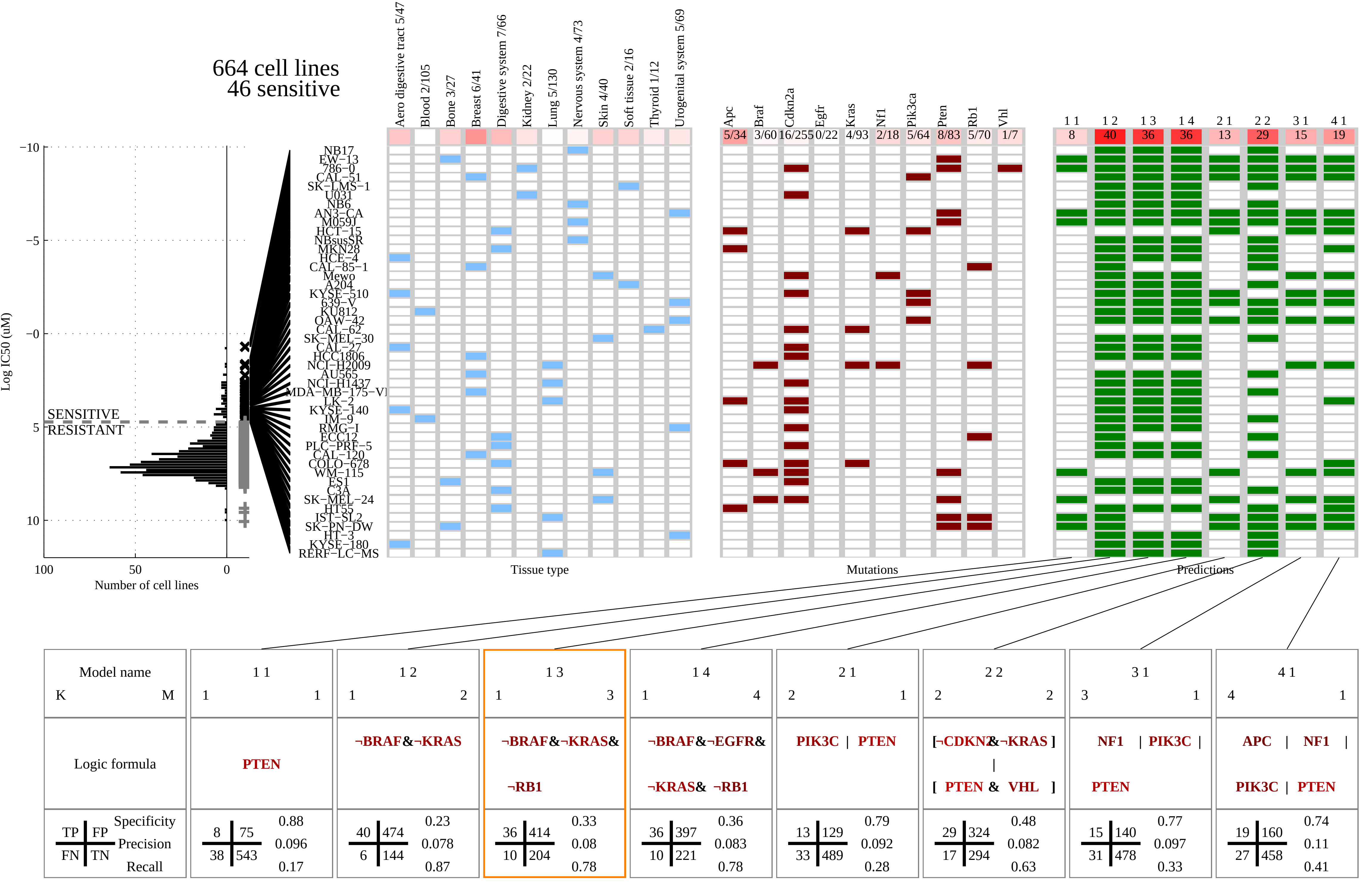

ID:150 Bicalutamide −> ANDR (androgen receptor)

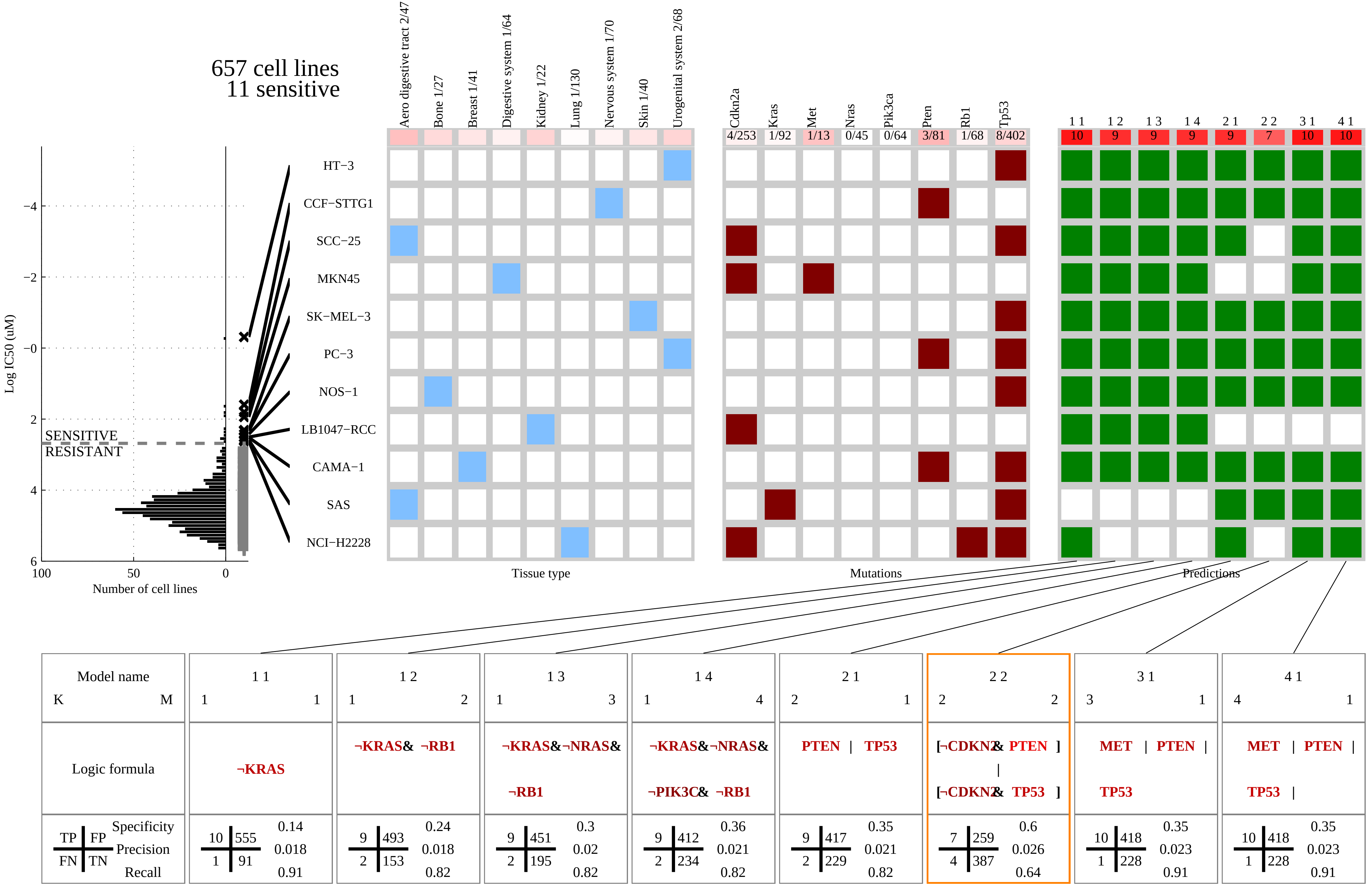

ID:151 QS11 −> ARFGAP

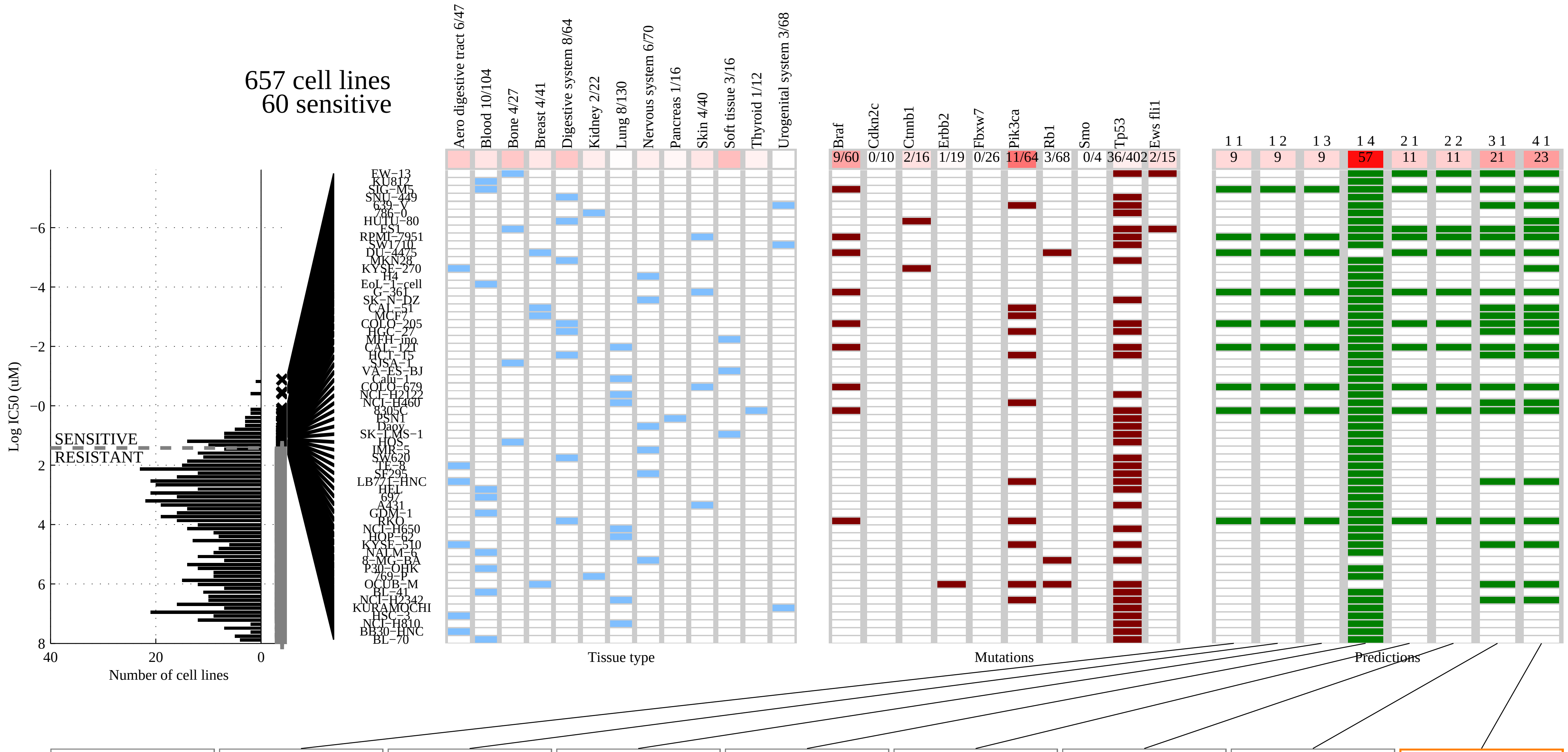

Model name	1 1	1 2	1 3	1 4	2 1	2 2	3 1	4 1
K / M	1 / 1	1 / 2	1 / 3	1 / 4	2 / 1	2 / 2	3 / 1	4 / 1
Logic formula	BRAF	BRAF & ¬SMO	BRAF & ¬CTNNB & ¬SMO	¬CDKN2 & ¬ERBB2 & ¬FBXW7 & ¬RB1	BRAF | EWS F	[BRAF & ¬SMO] | [TP53 & EWS F]	BRAF | PIK3C | EWS F	BRAF | CTNNB | PIK3C | EWS F
TP / FP	9 / 51	9 / 47	9 / 45	57 / 480	11 / 63	11 / 56	21 / 114	23 / 122
FN / TN	51 / 546	51 / 550	51 / 552	3 / 117	49 / 534	49 / 541	39 / 483	37 / 475
Specificity	0.91	0.92	0.92	0.2	0.89	0.91	0.81	0.8
Precision	0.15	0.16	0.17	0.11	0.15	0.16	0.16	0.16
Recall	0.15	0.15	0.15	0.95	0.18	0.18	0.35	0.38

ID:152 CP466722 −> ATM

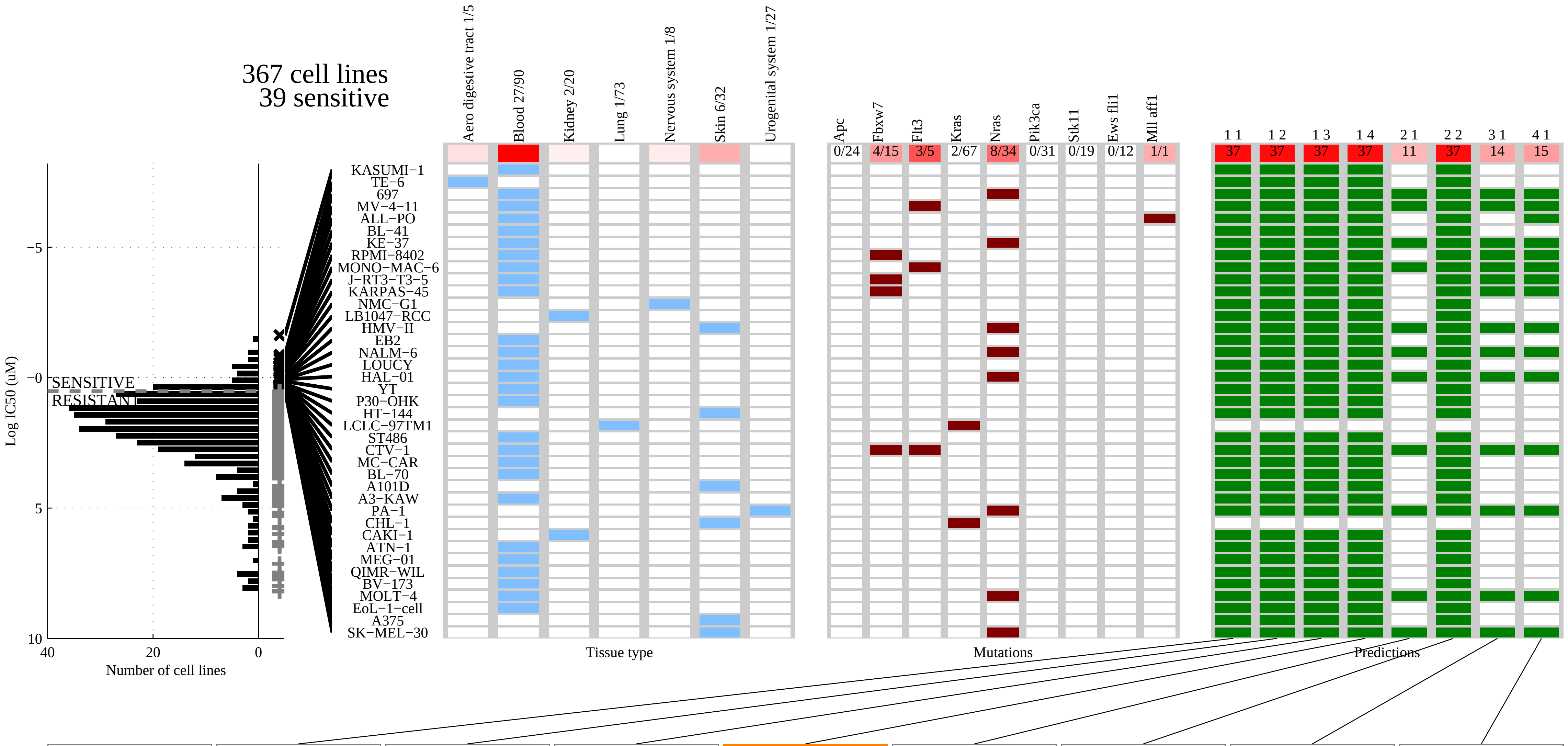

<table>
<tr><th>Model name
K M</th><th>1 1
1 1</th><th>1 2
1 2</th><th>1 3
1 3</th><th>1 4
1 4</th><th>2 1
2 1</th><th>2 2
2 2</th><th>3 1
3 1</th><th>4 1
4 1</th></tr>
<tr><td>Logic formula</td><td>¬KRAS</td><td>¬KRAS&¬PIK3C</td><td>¬KRAS&¬PIK3C& ¬STK11</td><td>¬APC &¬KRAS& ¬PIK3C&¬STK11</td><td>FLT3 | NRAS</td><td>[FLT3 & EWS F] | [¬KRAS&¬PIK3C]</td><td>FBXW7 | FLT3 | NRAS</td><td>FBXW7 | FLT3 | NRAS | MLL A</td></tr>
<tr><td>TP FP / FN TN</td><td>37 263 / 2 65</td><td>37 239 / 2 89</td><td>37 228 / 2 100</td><td>37 218 / 2 110</td><td>11 27 / 28 301</td><td>37 239 / 2 89</td><td>14 35 / 25 293</td><td>15 35 / 24 293</td></tr>
<tr><td>Specificity</td><td>0.2</td><td>0.27</td><td>0.3</td><td>0.34</td><td>0.92</td><td>0.27</td><td>0.89</td><td>0.89</td></tr>
<tr><td>Precision</td><td>0.12</td><td>0.13</td><td>0.14</td><td>0.15</td><td>0.29</td><td>0.13</td><td>0.29</td><td>0.3</td></tr>
<tr><td>Recall</td><td>0.95</td><td>0.95</td><td>0.95</td><td>0.95</td><td>0.28</td><td>0.95</td><td>0.36</td><td>0.38</td></tr>
</table>

ID:153 Midostaurin −> KIT

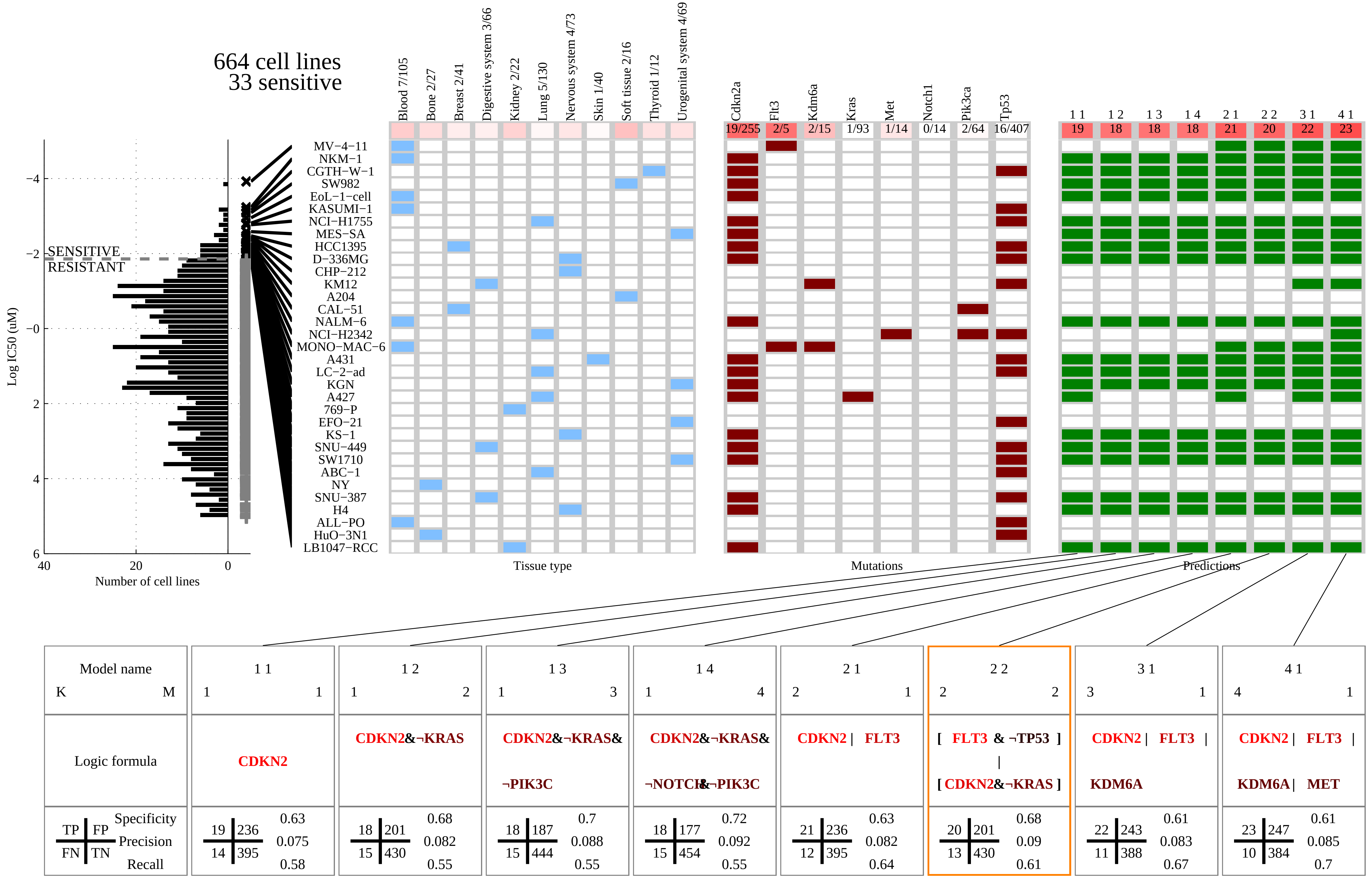

ID:154 CHIR−99021 −> GSK3B

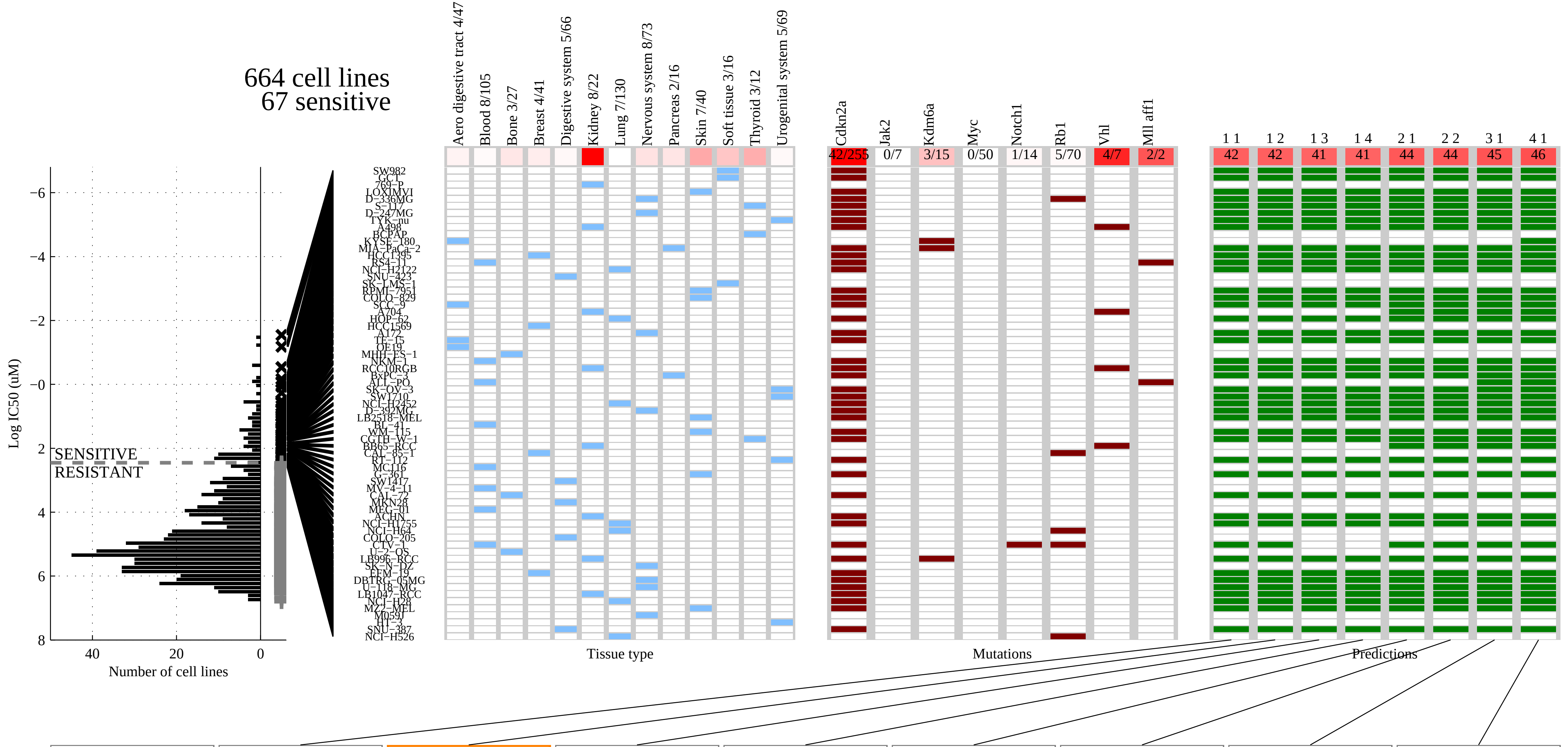

Model name	1 1	1 2	1 3	1 4	2 1	2 2	3 1	4 1
K / M	1 / 1	1 / 2	1 / 3	1 / 4	2 / 1	2 / 2	3 / 1	4 / 1
Logic formula	CDKN2	CDKN2& ¬MYC	CDKN2& ¬MYC & ¬NOTCH	CDKN2& ¬JAK2 & ¬MYC &NOTCH	CDKN2 | VHL	[CDKN2& ¬MYC] | [¬RB1 & VHL]	CDKN2 | VHL | MLL A	CDKN2 |KDM6A| VHL | MLL A
TP FP / FN TN	42 213 / 25 384	42 195 / 25 402	41 185 / 26 412	41 179 / 26 418	44 214 / 23 383	44 195 / 23 402	45 214 / 22 383	46 222 / 21 375
Specificity	0.64	0.67	0.69	0.7	0.64	0.67	0.64	0.63
Precision	0.16	0.18	0.18	0.19	0.17	0.18	0.17	0.17
Recall	0.63	0.63	0.61	0.61	0.66	0.66	0.67	0.69

ID:156 AZD6482 −> PI3Kbeta

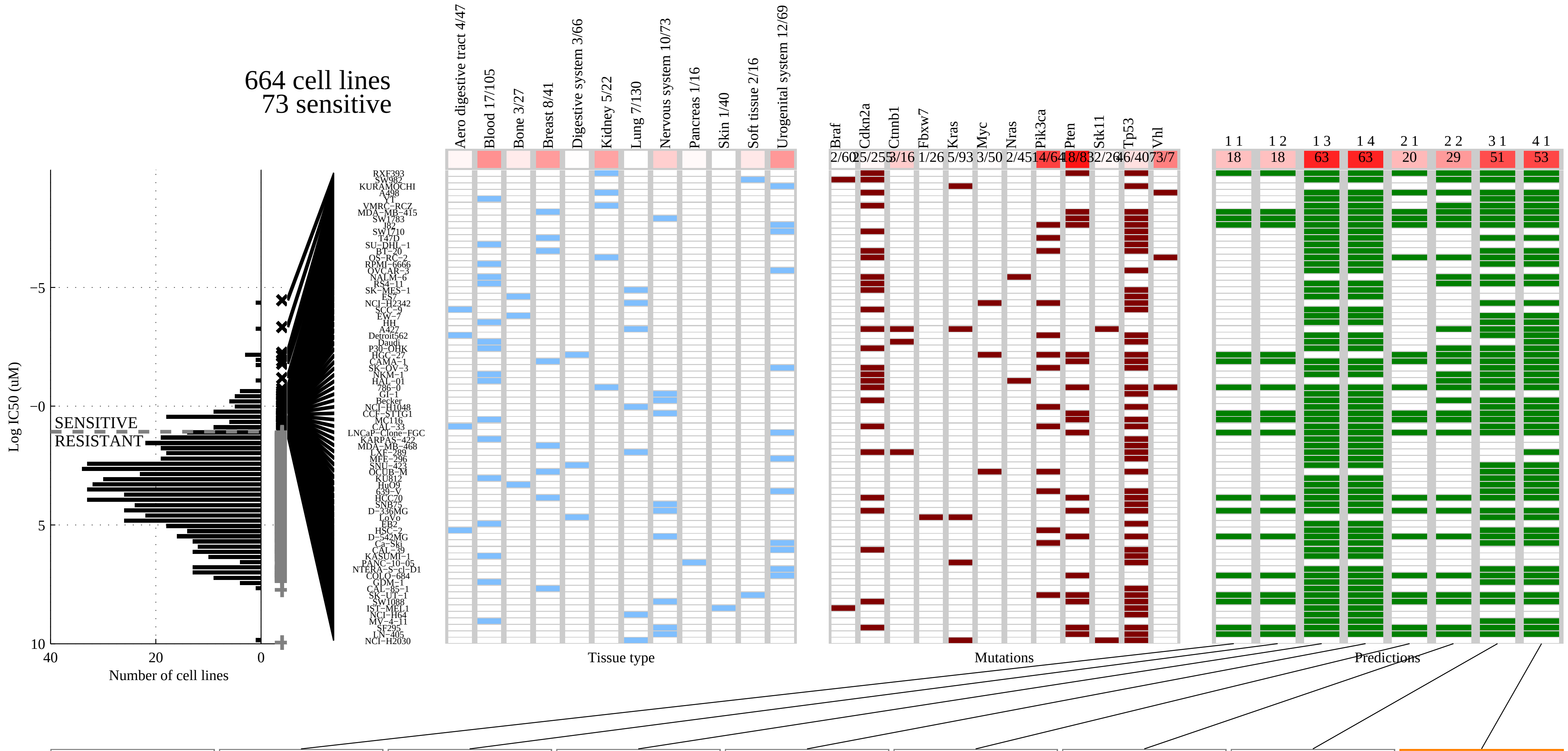

Model name (K, M)	1 1 (1, 1)	1 2 (1, 2)	1 3 (1, 3)	1 4 (1, 4)	2 1 (2, 1)	2 2 (2, 2)	3 1 (3, 1)	4 1 (4, 1)
Logic formula	PTEN	¬BRAF& PTEN	¬KRAS& ¬MYC & ¬NRAS	¬KRAS& ¬MYC & ¬NRAS&¬STK11	PTEN | VHL	[¬FBXW7& PTEN] | [CDKN2& ¬TP53]	PIK3C | PTEN | ¬TP53	CTNNB | PIK3C | PTEN | ¬TP53
TP	18	18	63	63	20	29	51	53
FP	65	51	424	406	69	140	301	301
FN	55	55	10	10	53	44	22	20
TN	526	540	167	185	522	451	290	290
Specificity	0.89	0.91	0.28	0.31	0.88	0.76	0.49	0.49
Precision	0.22	0.26	0.13	0.13	0.22	0.17	0.14	0.15
Recall	0.25	0.25	0.86	0.86	0.27	0.4	0.7	0.73

ID:157 JNK−9L −> JNK

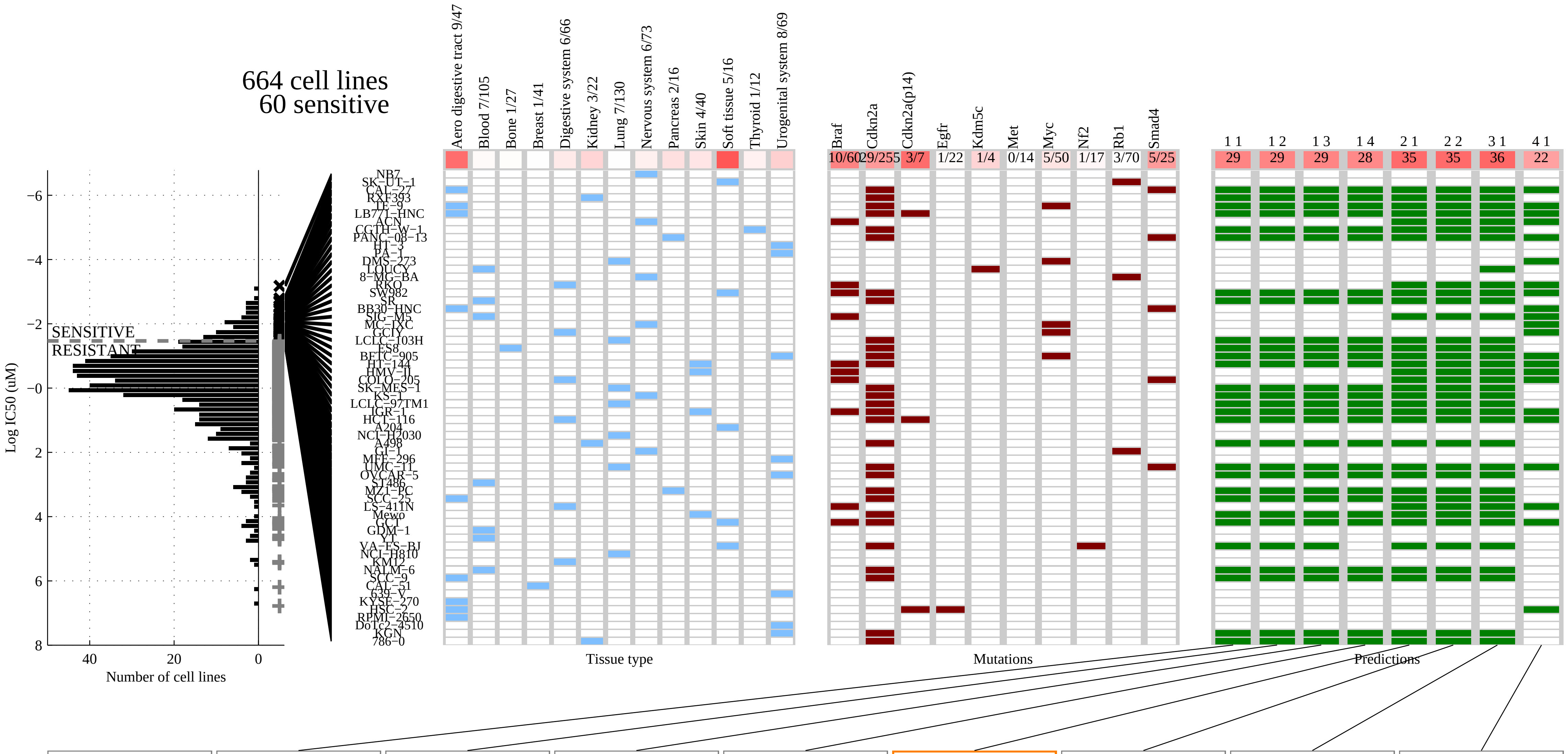

Model name (K, M)	1 1 (1, 1)	1 2 (1, 2)	1 3 (1, 3)	1 4 (1, 4)	2 1 (2, 1)	2 2 (2, 2)	3 1 (3, 1)	4 1 (4, 1)
Logic formula	**CDKN2**	**CDKN2& ¬RB1**	**CDKN2& ¬MET & ¬RB1**	**CDKN2& ¬MET & ¬NF2 & ¬RB1**	**BRAF | CDKN2**	**[CDKN2&¬EGFR] | [BRAF &¬EGFR]**	**BRAF | CDKN2 | KDM5C**	**BRAF | CDKN2 | MYC | SMAD4**
TP / FP	29 / 226	29 / 216	29 / 208	28 / 196	35 / 243	35 / 232	36 / 245	22 / 112
FN / TN	31 / 378	31 / 388	31 / 396	32 / 408	25 / 361	25 / 372	24 / 359	38 / 492
Specificity	0.63	0.64	0.66	0.68	0.6	0.62	0.59	0.81
Precision	0.11	0.12	0.12	0.13	0.13	0.13	0.13	0.16
Recall	0.48	0.48	0.48	0.47	0.58	0.58	0.6	0.37

ID:159 HG−6−64−1 −> BRAFV600E, TAK, MAP4K5

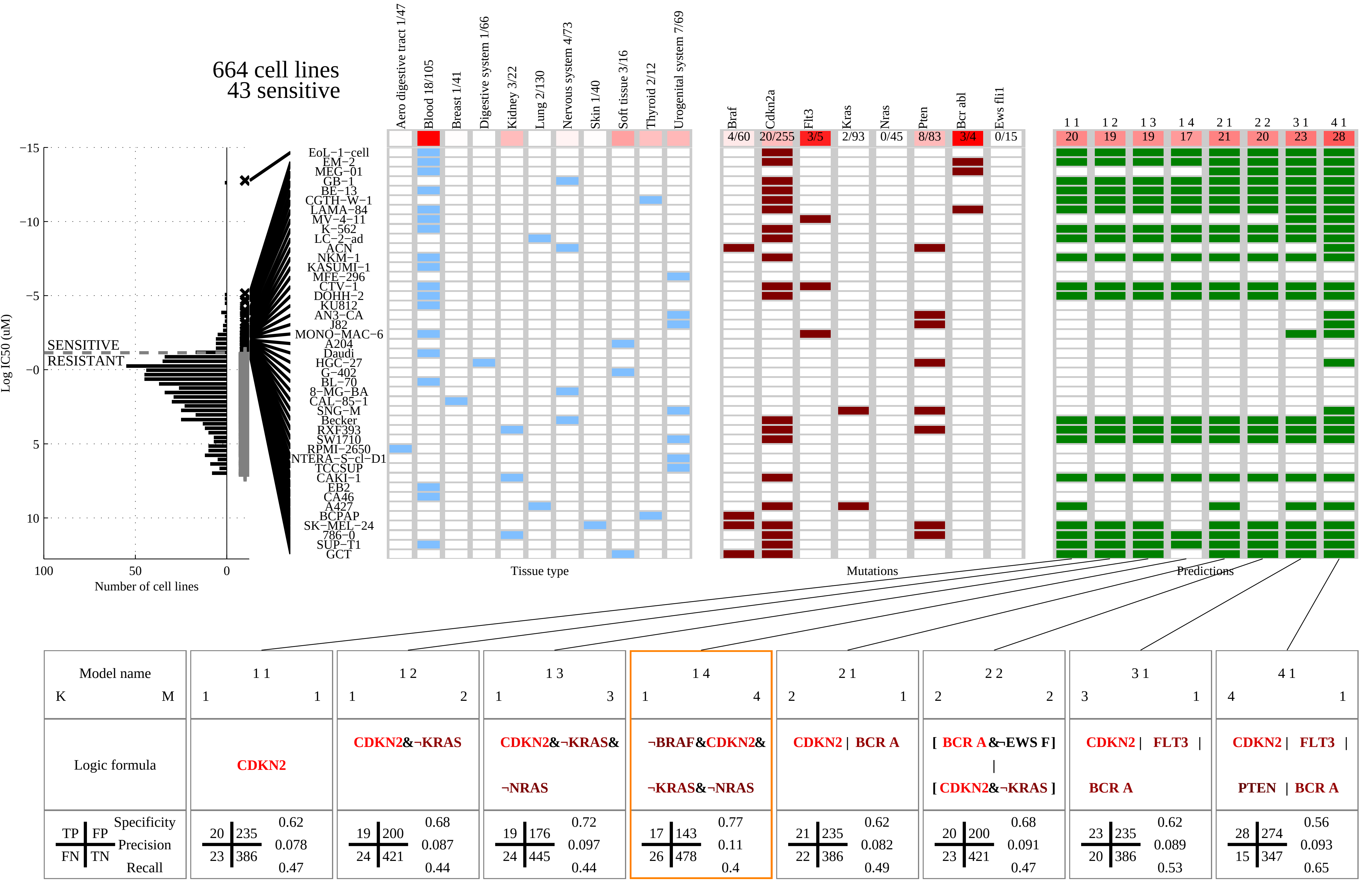

ID:163 JQ1 –> BRD4

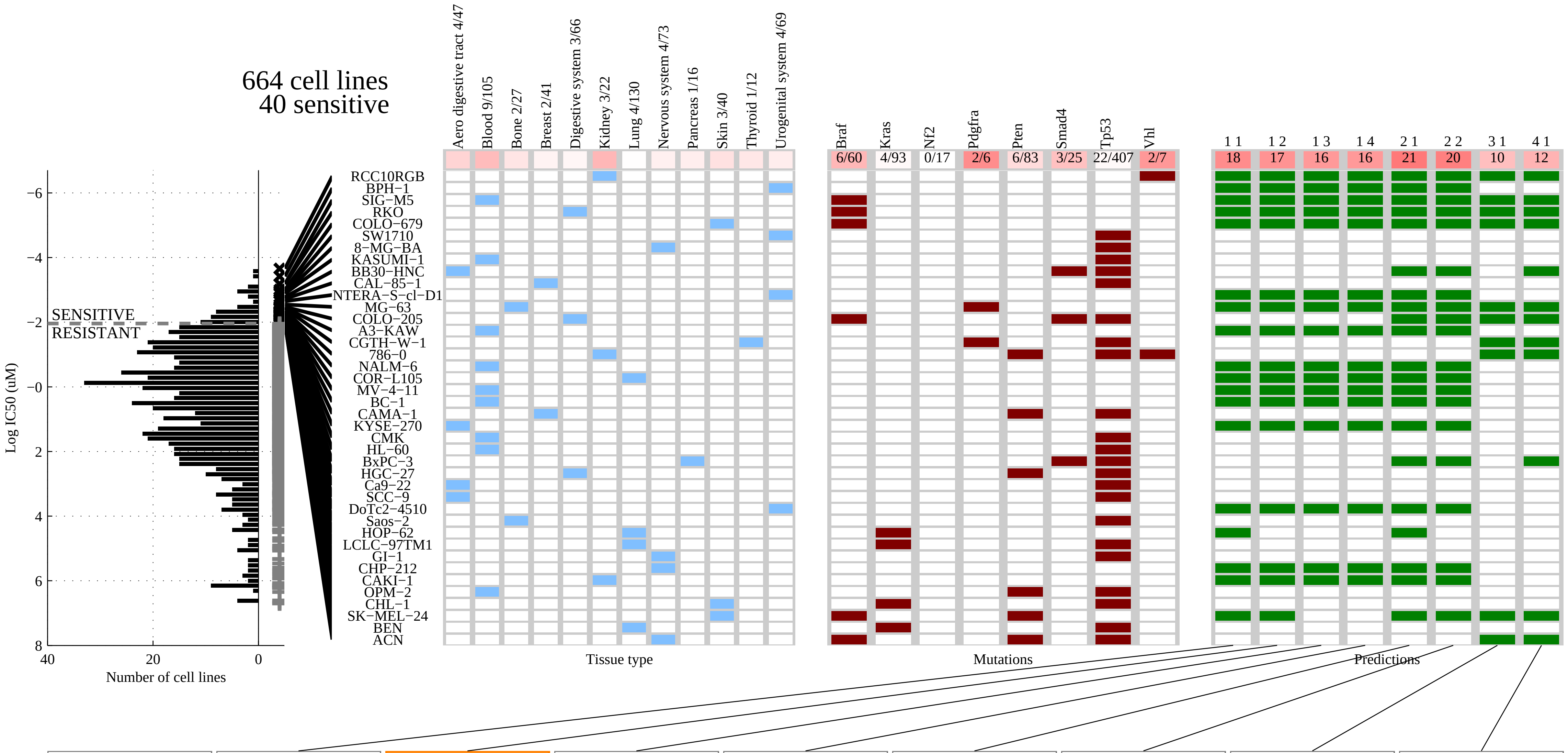

Model name	1 1	1 2	1 3	1 4	2 1	2 2	3 1	4 1
K / M	1 / 1	1 / 2	1 / 3	1 / 4	2 / 1	2 / 2	3 / 1	4 / 1
Logic formula	¬TP53	¬KRAS& ¬TP53	¬KRAS&¬PTEN& ¬TP53	¬KRAS& ¬NF2 & ¬PTEN& ¬TP53	SMAD4 | ¬TP53	[¬KRAS&SMAD4] | [¬KRAS& ¬TP53]	BRAF | PDGFR | VHL	BRAF | PDGFR | SMAD4 | VHL
TP FP / FN TN	18 239 / 22 385	17 209 / 23 415	16 187 / 24 437	16 179 / 24 445	21 254 / 19 370	20 218 / 20 406	10 63 / 30 561	12 83 / 28 541
Specificity	0.62	0.67	0.7	0.71	0.59	0.65	0.9	0.87
Precision	0.07	0.075	0.079	0.082	0.076	0.084	0.14	0.13
Recall	0.45	0.42	0.4	0.4	0.53	0.5	0.25	0.3

ID:164 JQ12 -> HDAC

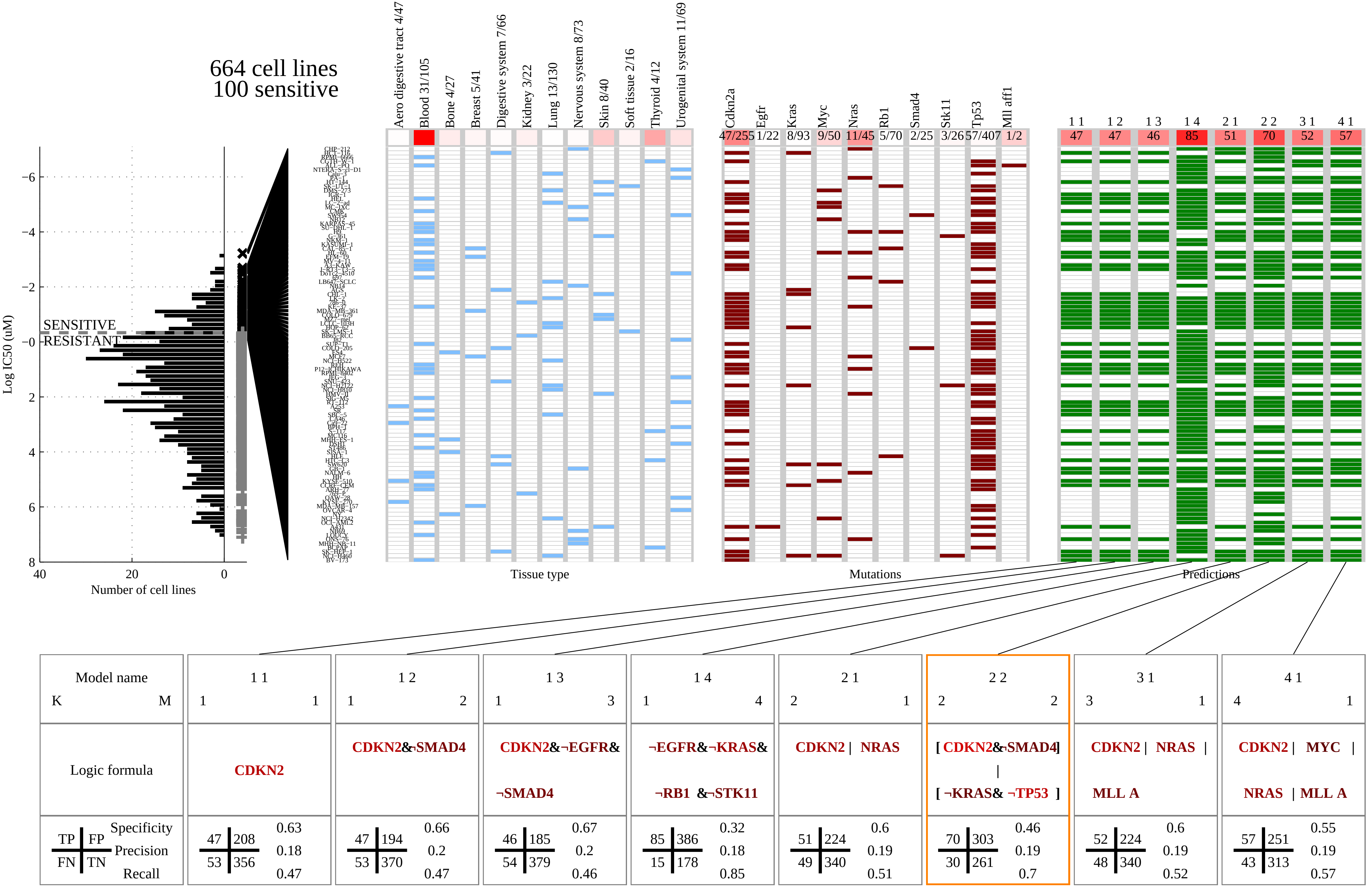

ID:165 DMOG −> Prolyl−4−Hydroxylase

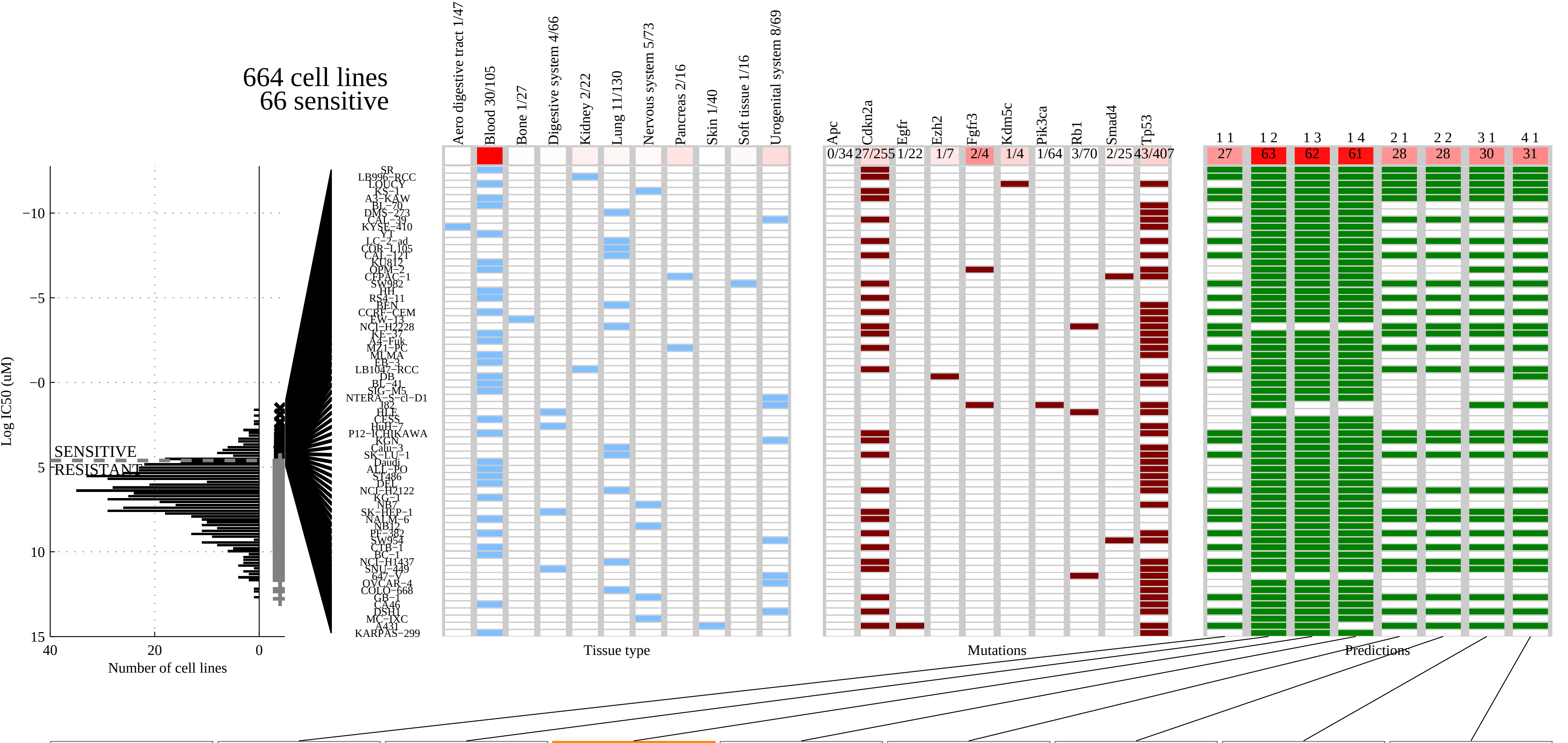

Model name	1 1	1 2	1 3	1 4	2 1	2 2	3 1	4 1
K / M	1 / 1	1 / 2	1 / 3	1 / 4	2 / 1	2 / 2	3 / 1	4 / 1
Logic formula	CDKN2	¬APC & ¬RB1	¬APC &¬PIK3C& ¬RB1	¬APC &¬EGFR& ¬PIK3C& ¬RB1	CDKN2 | KDM5C	[CDKN2&¬SMAD4] | [KDM5C& TP53]	CDKN2 | FGFR3 | KDM5C	CDKN2 | EZH2 | FGFR3 | KDM5C
TP FP	27 228	63 500	62 454	61 439	28 230	28 214	30 231	31 233
FN TN	39 370	3 98	4 144	5 159	38 368	38 384	36 367	35 365
Specificity	0.62	0.16	0.24	0.27	0.62	0.64	0.61	0.61
Precision	0.11	0.11	0.12	0.12	0.11	0.12	0.11	0.12
Recall	0.41	0.95	0.94	0.92	0.42	0.42	0.45	0.47

ID:166 FTI−277 −> Farnesyl transferase (FNTA)

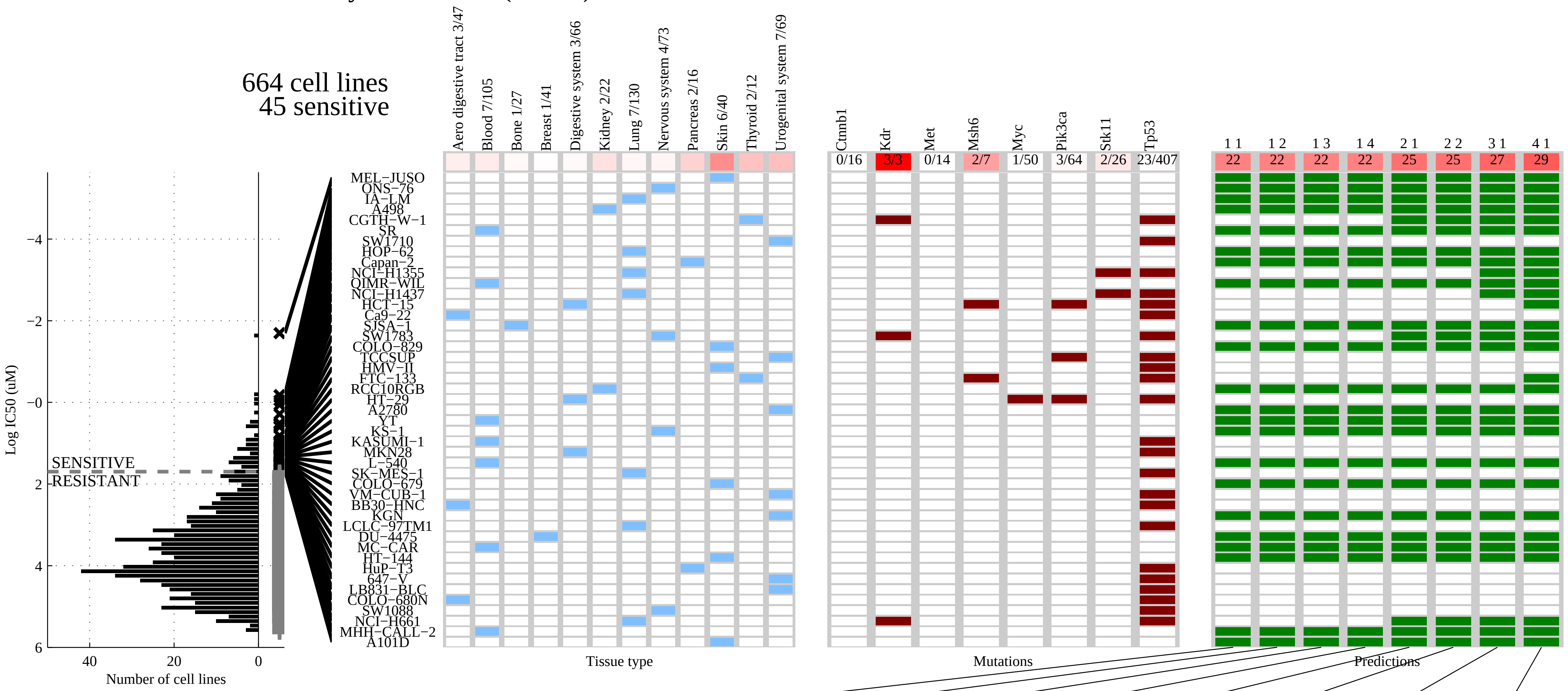

<table>
<tr><td>Model name
K M</td><td>1 1
1 1</td><td>1 2
1 2</td><td>1 3
1 3</td><td>1 4
1 4</td><td>2 1
2 1</td><td>2 2
2 2</td><td>3 1
3 1</td><td>4 1
4 1</td></tr>
<tr><td>Logic formula</td><td>¬TP53</td><td>¬PIK3C& ¬TP53</td><td>¬CTNNB& ¬PIK3C&
¬TP53</td><td>¬CTNNB& ¬MYC &
¬PIK3C& ¬TP53</td><td>KDR | ¬TP53</td><td>[¬PIK3C& ¬TP53]
|
[KDR & ¬MET]</td><td>KDR | STK11 |
¬TP53</td><td>KDR | MSH6 |
STK11 | ¬TP53</td></tr>
<tr><td>TP FP / FN TN
Specificity
Precision
Recall</td><td>22 235 / 23 384
0.62
0.086
0.49</td><td>22 211 / 23 408
0.66
0.094
0.49</td><td>22 201 / 23 418
0.68
0.099
0.49</td><td>22 190 / 23 429
0.69
0.1
0.49</td><td>25 235 / 20 384
0.62
0.096
0.56</td><td>25 211 / 20 408
0.66
0.11
0.56</td><td>27 252 / 18 367
0.59
0.097
0.6</td><td>29 254 / 16 365
0.59
0.1
0.64</td></tr>
</table>

ID:167 OSU−03012 −> PDPK1 (PDK1)

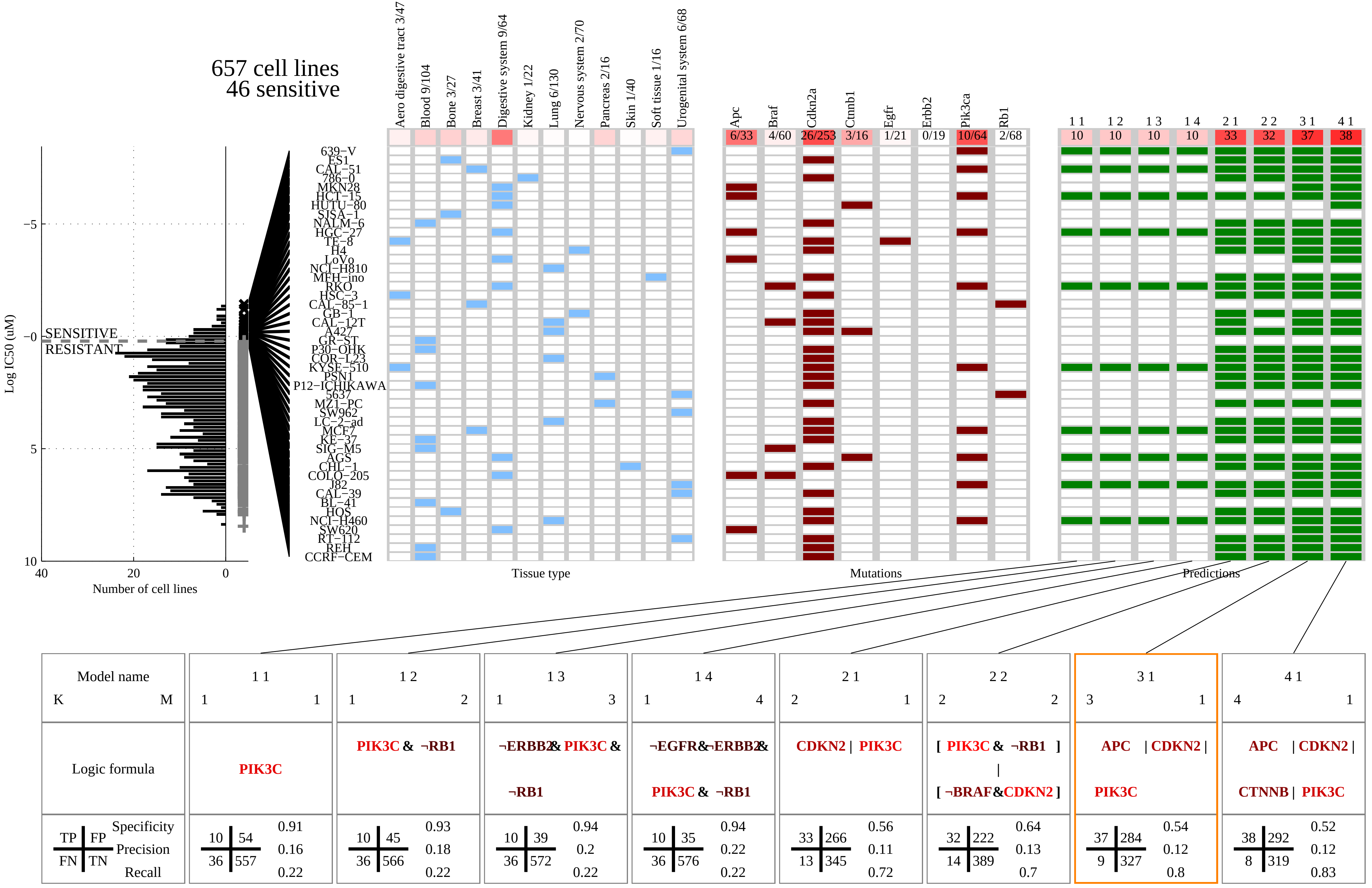

ID:168 PKM2_46 –> PKM2

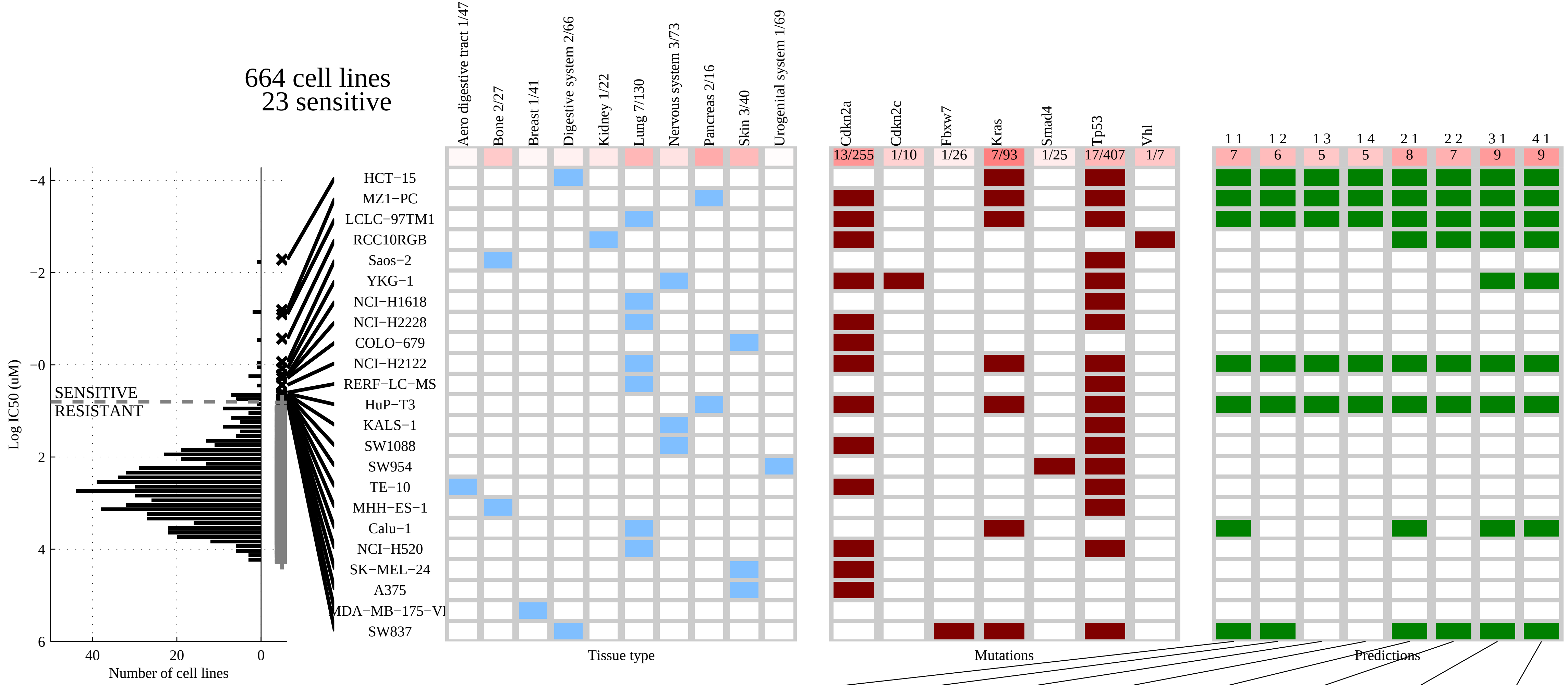

Model name	1 1	1 2	1 3	1 4	2 1	2 2	3 1	4 1
K / M	1 / 1	1 / 2	1 / 3	1 / 4	2 / 1	2 / 2	3 / 1	4 / 1
Logic formula	KRAS	KRAS & TP53	¬FBXW7 & KRAS & TP53	¬FBXW7 & KRAS & ¬SMAD4 & TP53	KRAS | VHL	[KRAS & TP53] | [CDKN2 & VHL]	CDKN2 | KRAS | VHL	CDKN2 | KRAS | VHL |
TP FP / FN TN	7 86 / 16 555	6 56 / 17 585	5 47 / 18 594	5 41 / 18 600	8 92 / 15 549	7 59 / 16 582	9 100 / 14 541	9 100 / 14 541
Specificity	0.87	0.91	0.93	0.94	0.86	0.91	0.84	0.84
Precision	0.075	0.097	0.096	0.11	0.08	0.11	0.083	0.083
Recall	0.3	0.26	0.22	0.22	0.35	0.3	0.39	0.39

ID:169 PKM2#58 –> PKM2

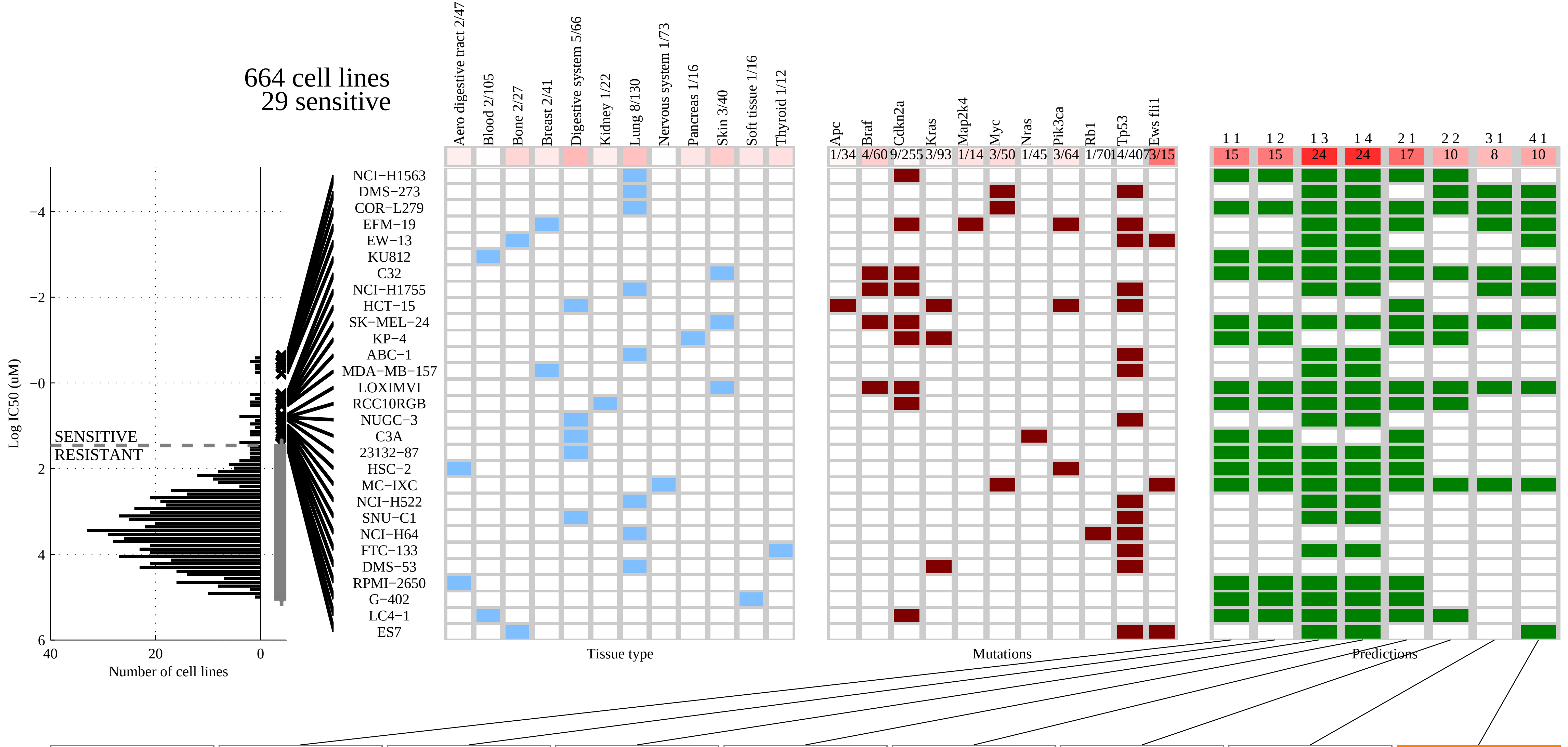

Model name	1 1	1 2	1 3	1 4	2 1	2 2	3 1	4 1
K / M	1 / 1	1 / 2	1 / 3	1 / 4	2 / 1	2 / 2	3 / 1	4 / 1
Logic formula	¬TP53	¬RB1 & ¬TP53	¬KRAS&¬NRAS& ¬RB1	¬APC &¬KRAS& ¬NRAS& ¬RB1	PIK3C | ¬TP53	[¬CDKN2& MYC] | [CDKN2& ¬TP53]	BRAF |MAP2K| MYC	BRAF |MAP2K| MYC | EWS F
TP / FP	15 / 242	15 / 227	24 / 440	24 / 426	17 / 280	10 / 131	8 / 107	10 / 117
FN / TN	14 / 393	14 / 408	5 / 195	5 / 209	12 / 355	19 / 504	21 / 528	19 / 518
Specificity	0.62	0.64	0.31	0.33	0.56	0.79	0.83	0.82
Precision	0.058	0.062	0.052	0.053	0.057	0.071	0.07	0.079
Recall	0.52	0.52	0.83	0.83	0.59	0.34	0.28	0.34

ID:17 Cyclopamine −> SMO

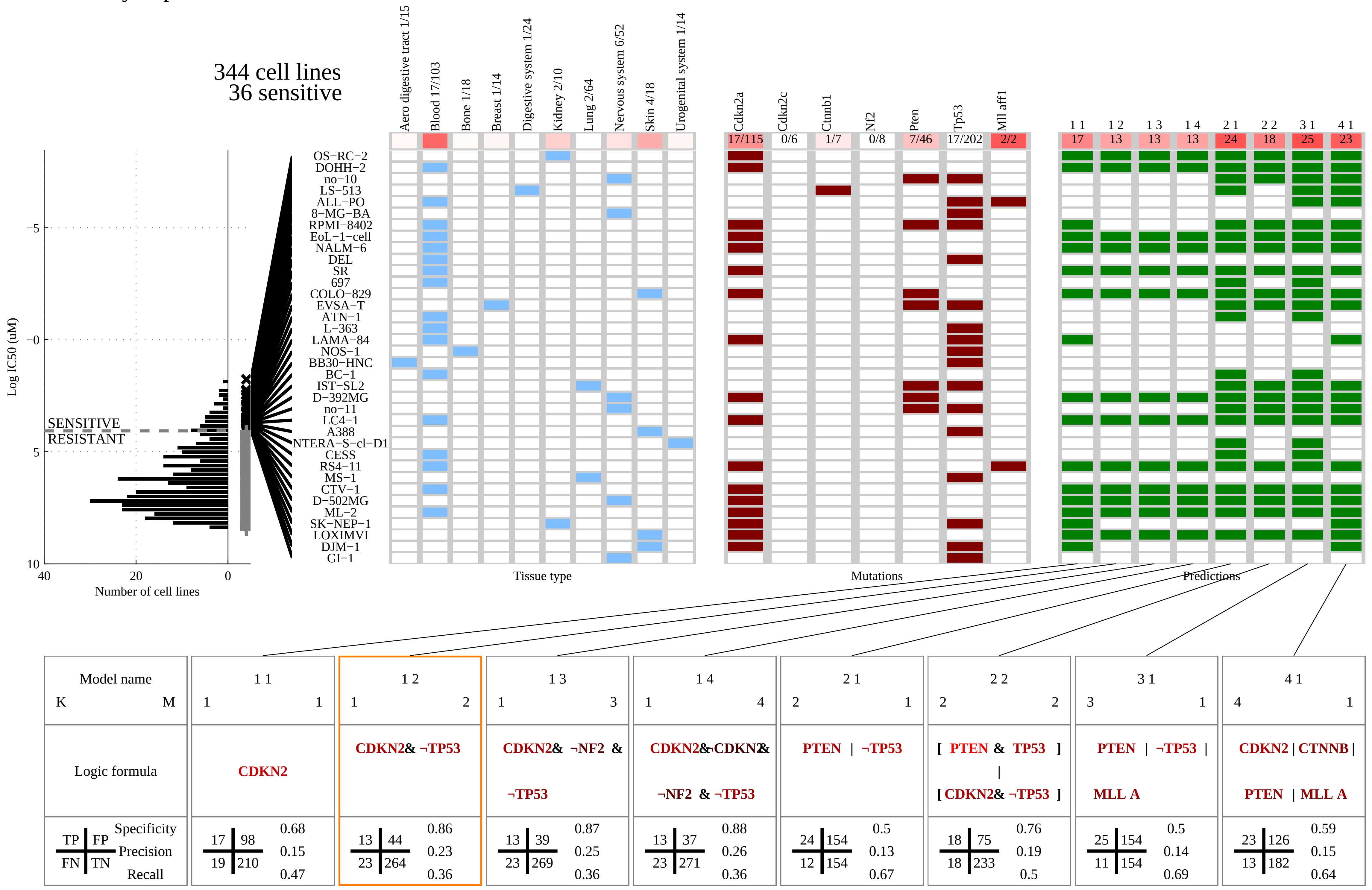

ID:170 Shikonin –> unknown

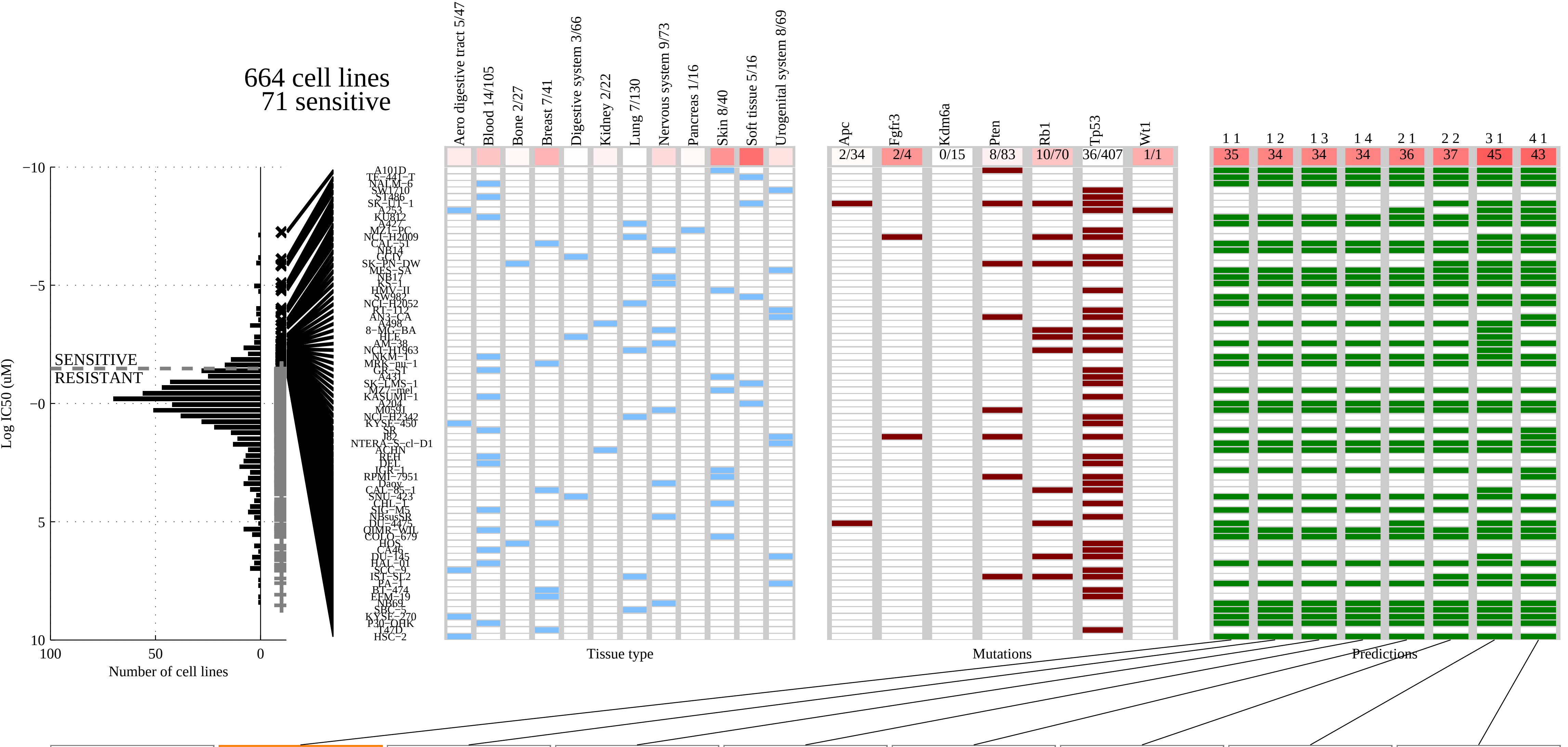

<table>
<tr><td>Model name
K M</td><td>1 1
1 1</td><td>1 2
1 2</td><td>1 3
1 3</td><td>1 4
1 4</td><td>2 1
2 1</td><td>2 2
2 2</td><td>3 1
3 1</td><td>4 1
4 1</td></tr>
<tr><td>Logic formula</td><td>¬TP53</td><td>¬RB1 & ¬TP53</td><td>¬APC & ¬RB1 & ¬TP53</td><td>¬APC & KDM6A & ¬RB1 & ¬TP53</td><td>¬TP53 | WT1</td><td>[PTEN & RB1] | [¬APC & ¬TP53]</td><td>RB1 | ¬TP53 | WT1</td><td>FGFR3 | PTEN | ¬TP53 | WT1</td></tr>
<tr><td>TP FP / FN TN</td><td>35 222 / 36 371</td><td>34 208 / 37 385</td><td>34 201 / 37 392</td><td>34 196 / 37 397</td><td>36 222 / 35 371</td><td>37 222 / 34 371</td><td>45 268 / 26 325</td><td>43 276 / 28 317</td></tr>
<tr><td>Specificity</td><td>0.63</td><td>0.65</td><td>0.66</td><td>0.67</td><td>0.63</td><td>0.63</td><td>0.55</td><td>0.53</td></tr>
<tr><td>Precision</td><td>0.14</td><td>0.14</td><td>0.14</td><td>0.15</td><td>0.14</td><td>0.14</td><td>0.14</td><td>0.13</td></tr>
<tr><td>Recall</td><td>0.49</td><td>0.48</td><td>0.48</td><td>0.48</td><td>0.51</td><td>0.52</td><td>0.63</td><td>0.61</td></tr>
</table>

ID:171 AKT inhibitor VIII –> AKT1, AKT2, AKT3

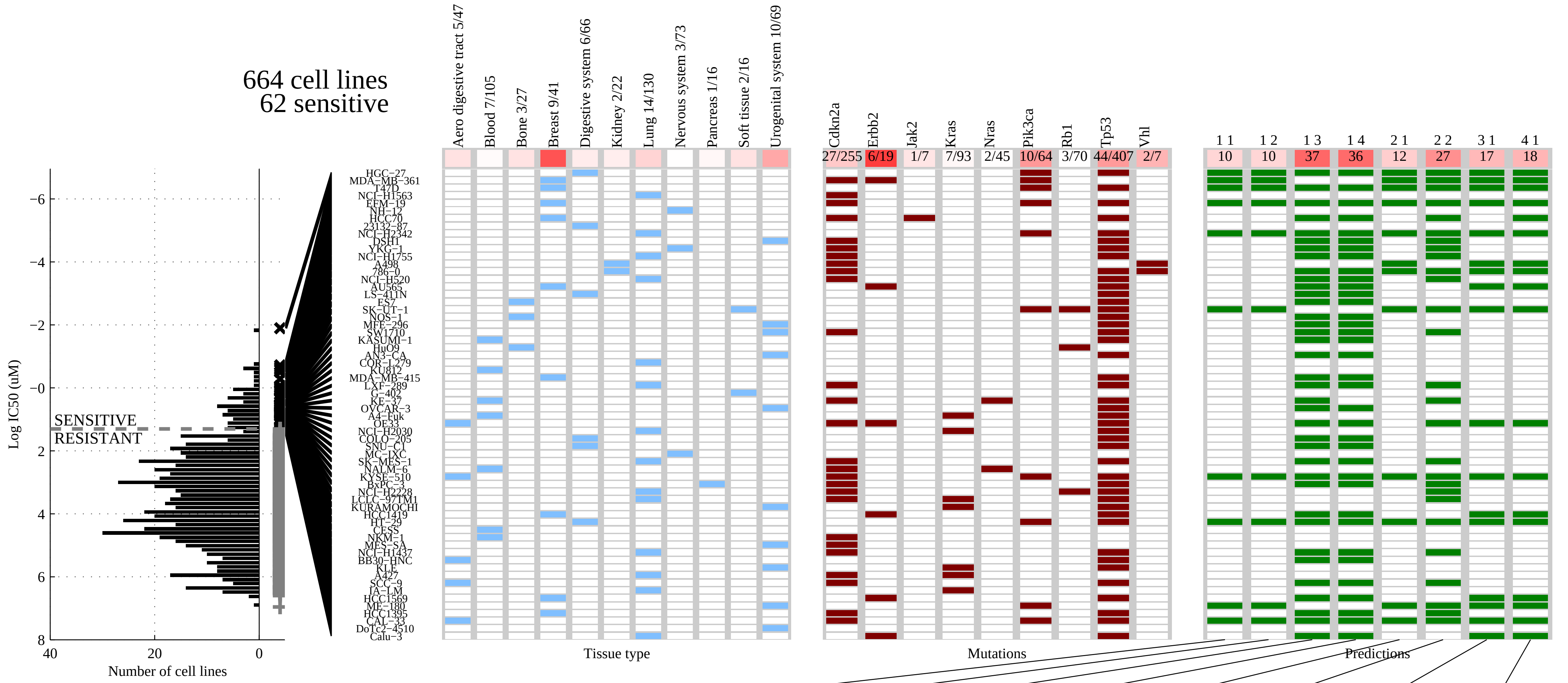

Model name (K, M)	1 1 (1, 1)	1 2 (1, 2)	1 3 (1, 3)	1 4 (1, 4)	2 1 (2, 1)	2 2 (2, 2)	3 1 (3, 1)	4 1 (4, 1)
Logic formula	PIK3C	¬KRAS & PIK3C	¬KRAS & ¬RB1 & TP53	¬KRAS & ¬NRAS & ¬RB1 & TP53	PIK3C | VHL	[CDKN2 & TP53] | [¬KRAS & PIK3C]	ERBB2 | PIK3C | VHL	ERBB2 | JAK2 | PIK3C | VHL
TP FP / FN TN	10 54 / 52 548	10 41 / 52 561	37 257 / 25 345	36 239 / 26 363	12 59 / 50 543	27 160 / 35 442	17 66 / 45 536	18 70 / 44 532
Specificity	0.91	0.93	0.57	0.6	0.9	0.73	0.89	0.88
Precision	0.16	0.2	0.13	0.13	0.17	0.14	0.2	0.2
Recall	0.16	0.16	0.6	0.58	0.19	0.44	0.27	0.29

ID:172 Embelin –> XIAP

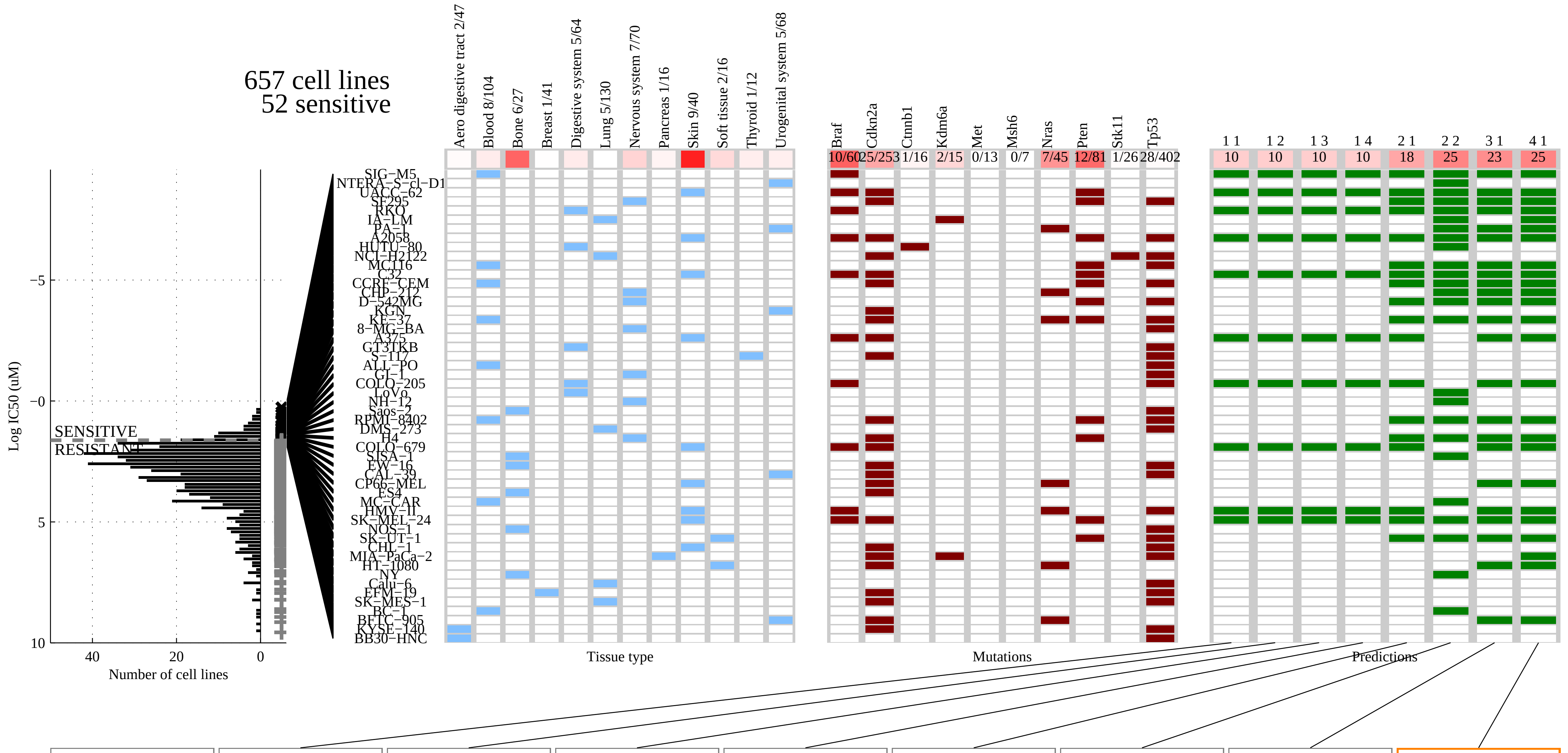

Model name (K, M)	1 1	1 2	1 3	1 4	2 1	2 2	3 1	4 1
K	1	1	1	1	2	2	3	4
M	1	2	3	4	1	2	1	1
Logic formula	BRAF	BRAF &¬STK11	BRAF &¬CTNNB& ¬STK11	BRAF &¬CTNNB& ¬MET &¬STK11	BRAF | PTEN	[¬MSH6& PTEN] | [¬CDKN2& ¬TP53]	BRAF | NRAS | PTEN	BRAF |KDM6A| NRAS | PTEN
TP	10	10	10	10	18	25	23	25
FP	50	47	45	41	109	191	142	152
FN	42	42	42	42	34	27	29	27
TN	555	558	560	564	496	414	463	453
Specificity	0.92	0.92	0.93	0.93	0.82	0.68	0.77	0.75
Precision	0.17	0.18	0.18	0.2	0.14	0.12	0.14	0.14
Recall	0.19	0.19	0.19	0.19	0.35	0.48	0.44	0.48

ID:173 FH535 –> unknown

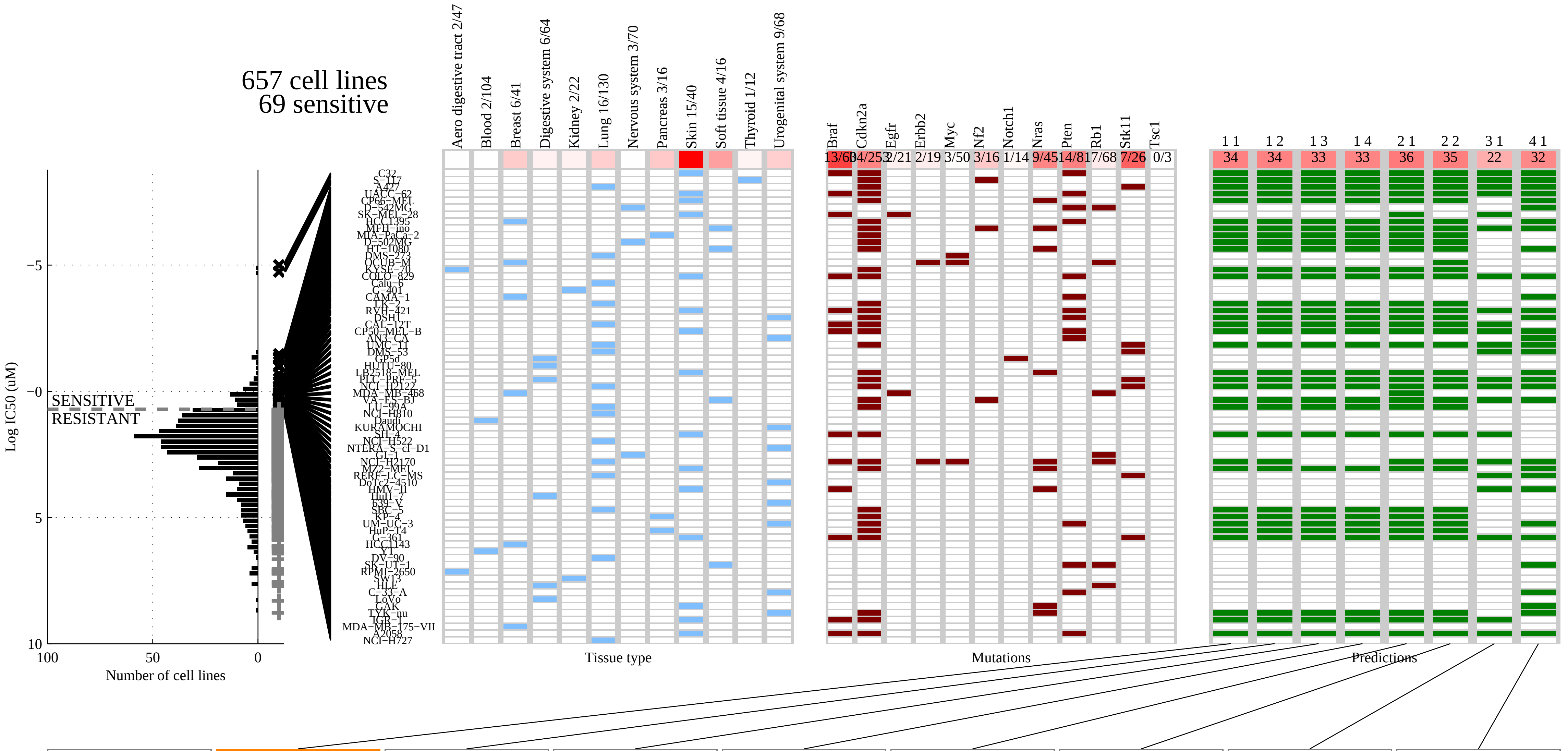

Model name (K, M)	1 1 (K=1, M=1)	1 2 (K=1, M=2)	1 3 (K=1, M=3)	1 4 (K=1, M=4)	2 1 (K=2, M=1)	2 2 (K=2, M=2)	3 1 (K=3, M=1)	4 1 (K=4, M=1)
Logic formula	CDKN2	CDKN2 & NOTCH	CDKN2 & ¬MYC & ¬NOTCH	CDKN2 & ¬MYC & ¬NOTCH & ¬RB1	CDKN2 | EGFR	[CDKN2 & ¬TSC1] | [ERBB2 & MYC]	BRAF | NF2 | STK11	NF2 | NRAS | PTEN | STK11
TP, FP / FN, TN	34, 219 / 35, 369	34, 207 / 35, 381	33, 191 / 36, 397	33, 185 / 36, 403	36, 229 / 33, 359	35, 219 / 34, 369	22, 72 / 47, 516	32, 129 / 37, 459
Specificity	0.63	0.65	0.68	0.69	0.61	0.63	0.88	0.78
Precision	0.13	0.14	0.15	0.15	0.14	0.14	0.23	0.2
Recall	0.49	0.49	0.48	0.48	0.52	0.51	0.32	0.46

ID:175 PAC−1 −> CASP3 agonist

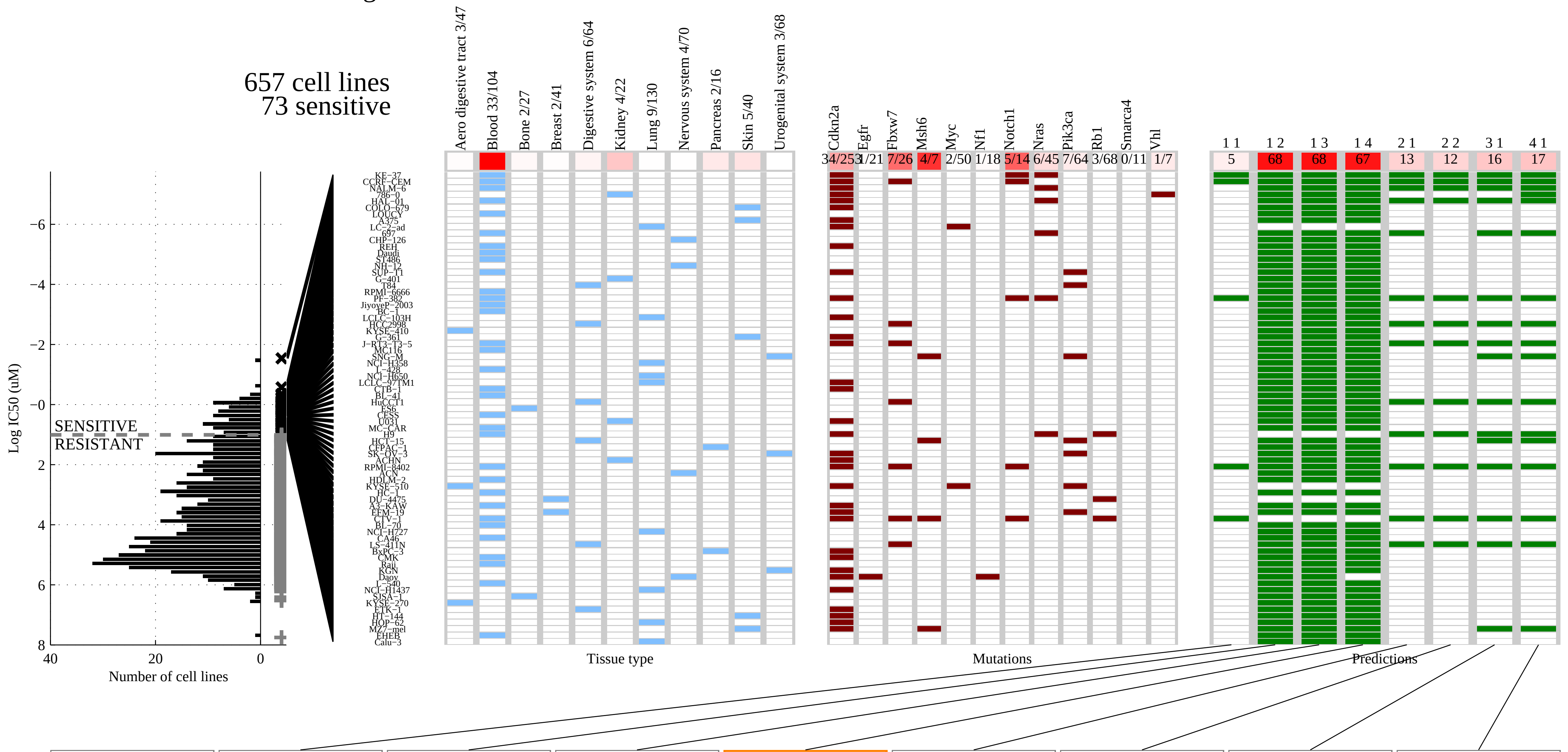

Model name (K, M)	1 1	1 2	1 3	1 4	2 1	2 2	3 1	4 1
K	1	1	1	1	2	2	3	4
M	1	2	3	4	1	2	1	1
Logic formula	NOTCH	¬MYC & ¬RB1	¬MYC & ¬RB1 & ¬SMARC	¬EGFR& ¬MYC & ¬NF1 & ¬RB1	FBXW7 | NRAS	[FBXW7&¬PIK3C] | [CDKN2& NRAS]	FBXW7 | MSH6 | NRAS	FBXW7 | MSH6 | NRAS | VHL
TP	5	68	68	67	13	12	16	17
FP	9	480	469	452	56	33	58	64
FN	68	5	5	6	60	61	57	56
TN	575	104	115	132	528	551	526	520
Specificity	0.98	0.18	0.2	0.23	0.9	0.94	0.9	0.89
Precision	0.36	0.12	0.13	0.13	0.19	0.27	0.22	0.21
Recall	0.068	0.93	0.93	0.92	0.18	0.16	0.22	0.23

ID:176 IPA−3 -> PAK1, PAK2, PAK3

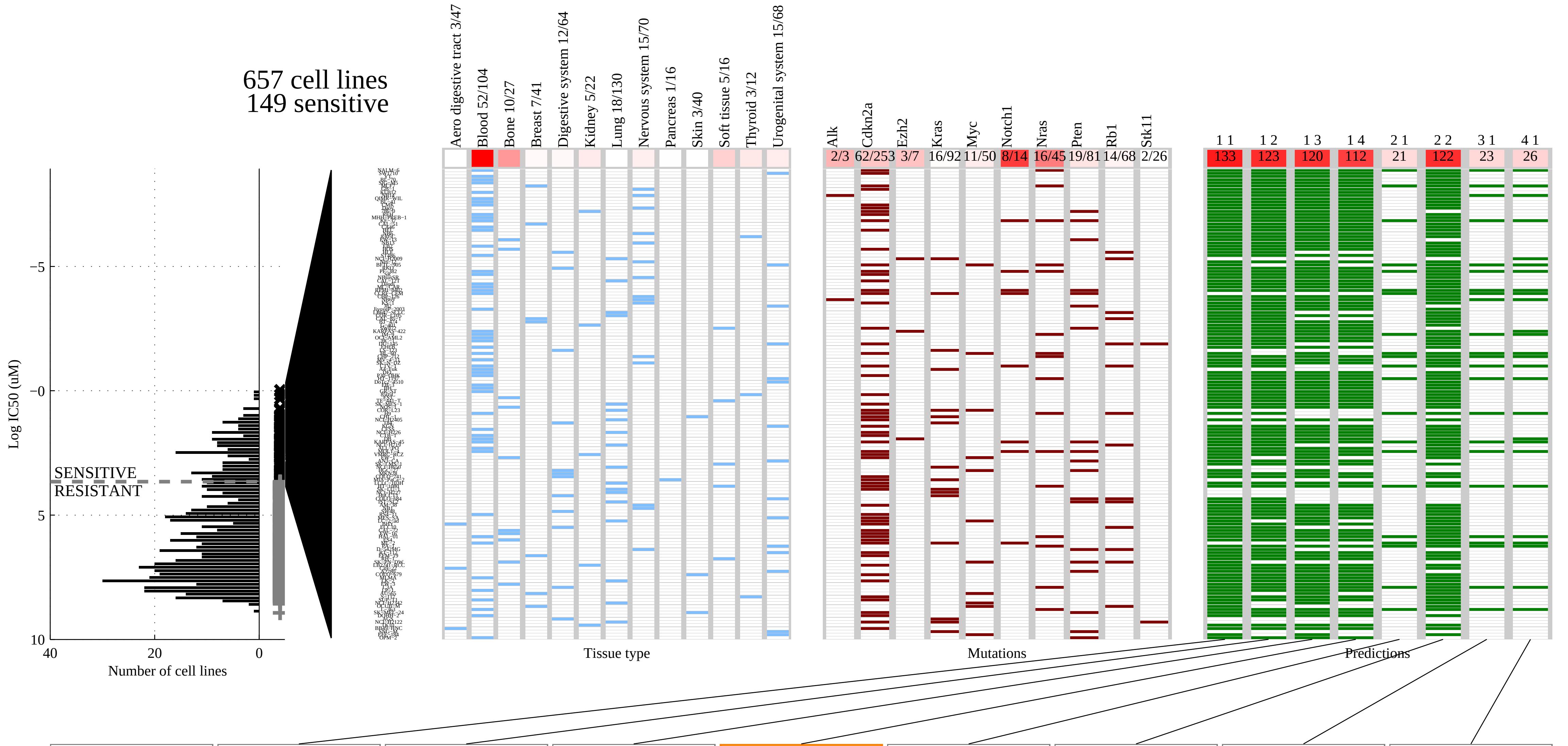

Model name (K, M)	1 1	1 2	1 3	1 4	2 1	2 2	3 1	4 1
K / M	1 / 1	1 / 2	1 / 3	1 / 4	2 / 1	2 / 2	3 / 1	4 / 1
Logic formula	¬KRAS	¬KRAS& ¬MYC	¬KRAS& ¬RB1 & ¬STK11	¬KRAS& ¬MYC & ¬RB1 &¬STK11	NOTCH| NRAS	[¬KRAS&¬PTEN] | [CDKN2&NOTCH]	ALK |NOTCH| NRAS	ALK | EZH2 | NOTCH| NRAS
TP FP / FN TN	133 432 / 16 76	123 399 / 26 109	120 365 / 29 143	112 337 / 37 171	21 34 / 128 474	122 373 / 27 135	23 35 / 126 473	26 38 / 123 470
Specificity	0.15	0.21	0.28	0.34	0.93	0.27	0.93	0.93
Precision	0.24	0.24	0.25	0.25	0.38	0.25	0.4	0.41
Recall	0.89	0.83	0.81	0.75	0.14	0.82	0.15	0.17

ID:177 GSK−650394 −> SGK3

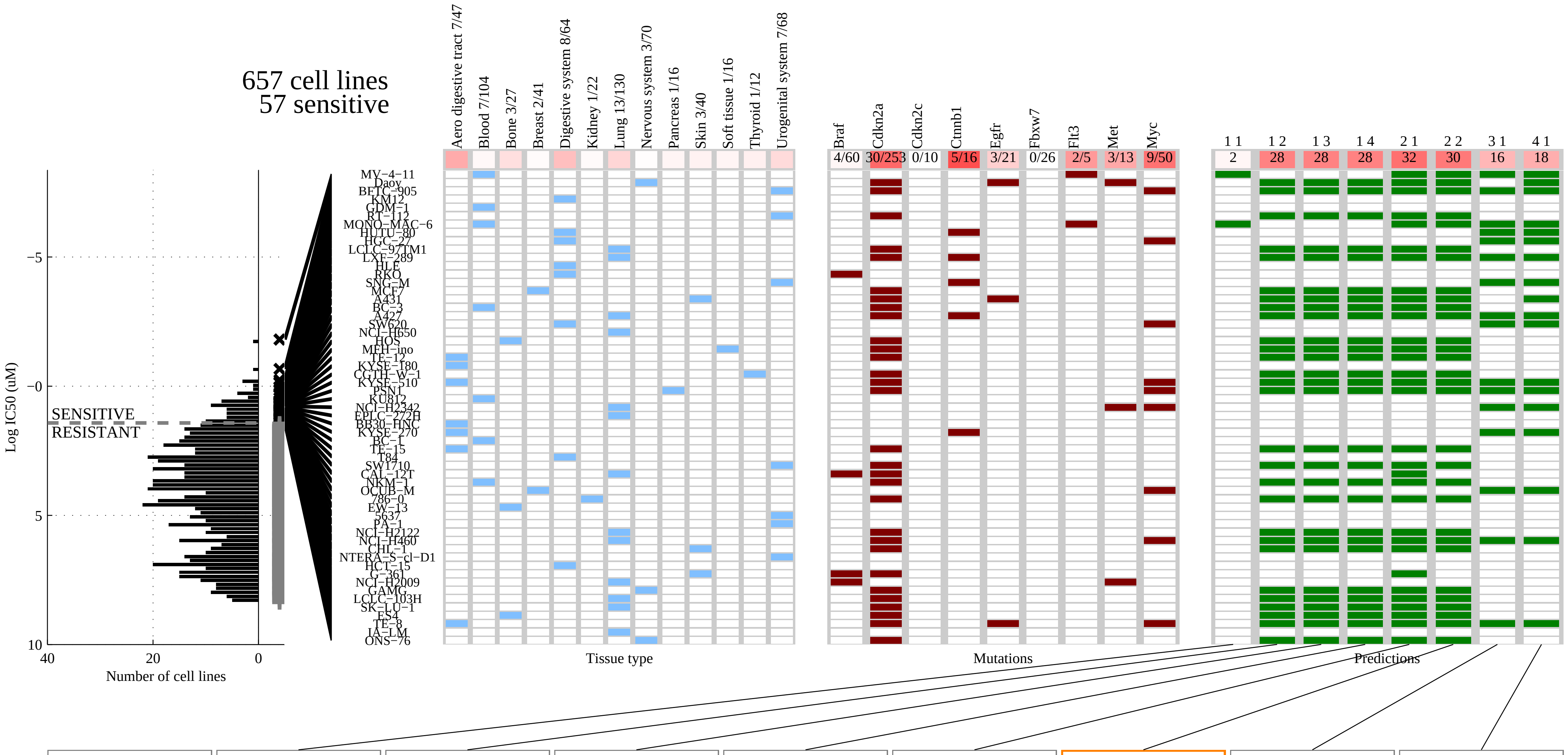

Model name K M	1 1 (K=1, M=1)	1 2 (K=1, M=2)	1 3 (K=1, M=3)	1 4 (K=1, M=4)	2 1 (K=2, M=1)	2 2 (K=2, M=2)	3 1 (K=3, M=1)	4 1 (K=4, M=1)
Logic formula	FLT3	¬BRAF&CDKN2	¬BRAF&CDKN2& ¬FBXW7	¬BRAF&CDKN2& ¬CDKN2&¬FBXW7	CDKN2 | FLT3	[¬BRAF&CDKN2] | [FLT3 & ¬MET]	CTNNB | FLT3 | MYC	CTNNB | EGFR | FLT3 | MYC
TP FP	2 3	28 188	28 178	28 172	32 223	30 188	16 54	18 67
FN TN	55 597	29 412	29 422	29 428	25 377	27 412	41 546	39 533
Specificity	1	0.69	0.7	0.71	0.63	0.69	0.91	0.89
Precision	0.4	0.13	0.14	0.14	0.13	0.14	0.23	0.21
Recall	0.035	0.49	0.49	0.49	0.56	0.53	0.28	0.32

ID:178 BAY 61−3606 −> SYK

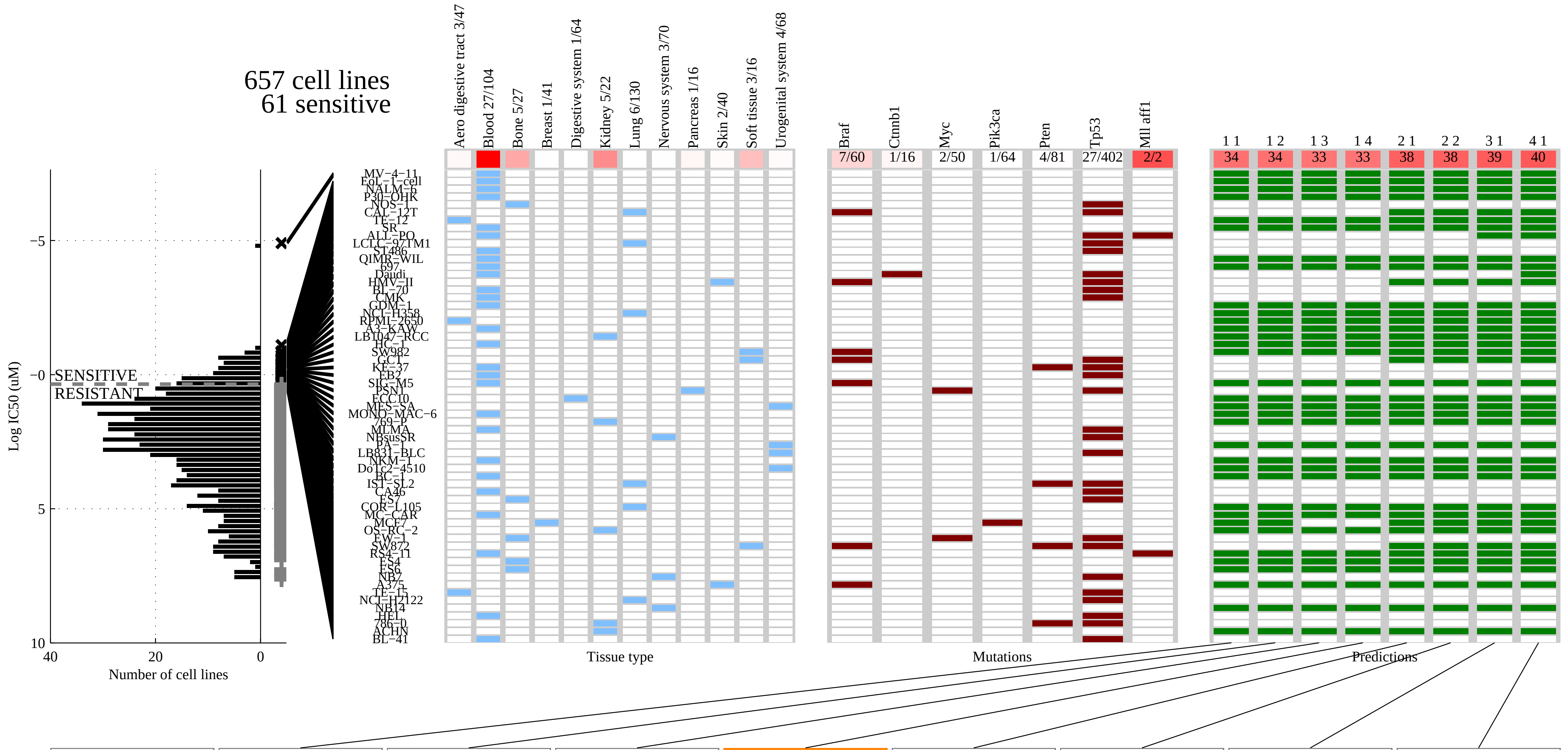

Model name	1 1	1 2	1 3	1 4	2 1	2 2	3 1	4 1
K / M	1 / 1	1 / 2	1 / 3	1 / 4	2 / 1	2 / 2	3 / 1	4 / 1
Logic formula	¬TP53	¬PTEN& ¬TP53	¬PIK3C&¬PTEN& ¬TP53	¬MYC &¬PIK3C& ¬PTEN& ¬TP53	BRAF | ¬TP53	[BRAF & TP53] | [¬PTEN& ¬TP53]	BRAF | ¬TP53 | MLL A	BRAF | CTNNB | ¬TP53 | MLL A
TP FP	34 221	34 197	33 175	33 165	38 247	38 223	39 247	40 248
FN TN	27 375	27 399	28 421	28 431	23 349	23 373	22 349	21 348
Specificity	0.63	0.67	0.71	0.72	0.59	0.63	0.59	0.58
Precision	0.13	0.15	0.16	0.17	0.13	0.15	0.14	0.14
Recall	0.56	0.56	0.54	0.54	0.62	0.62	0.64	0.66

ID:180 Thapsigargin −> sarco−endoplasmic reticulum Ca2+−ATPases

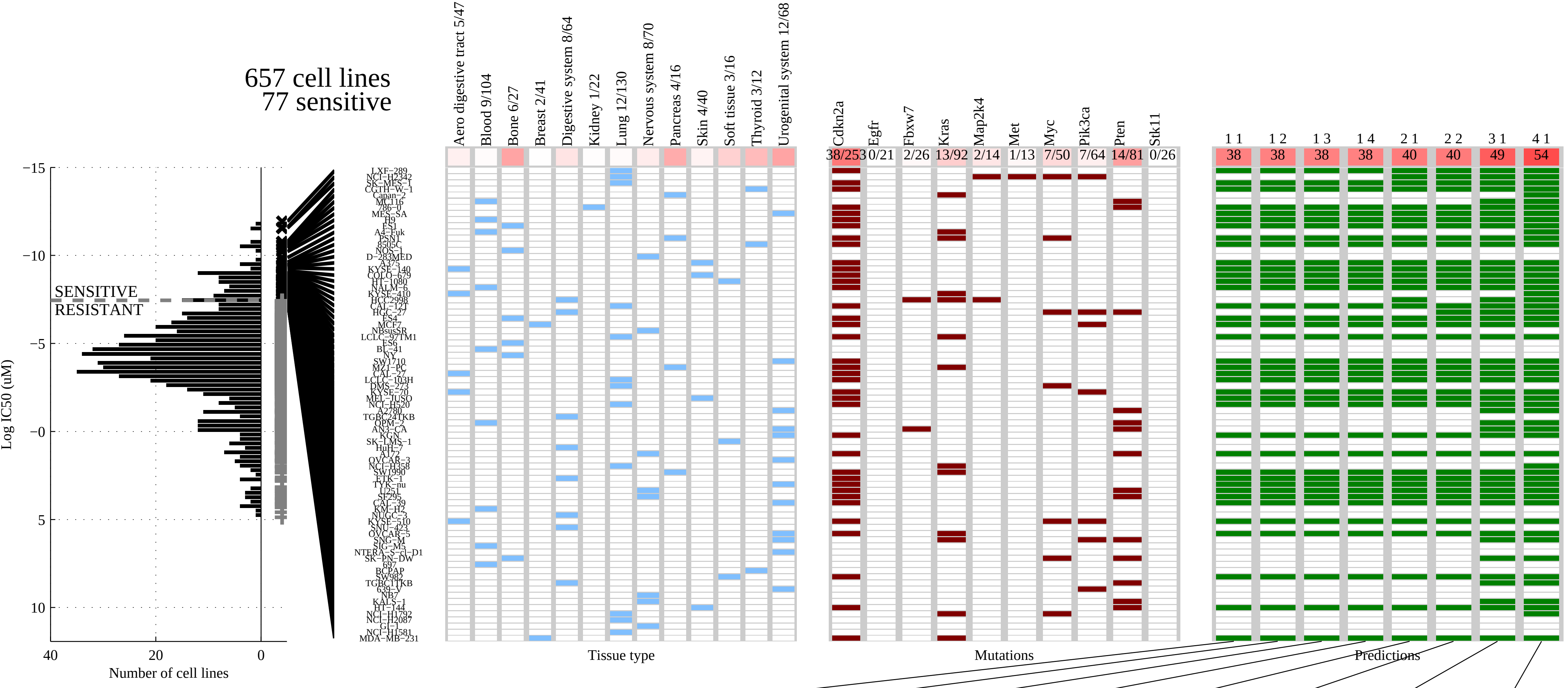

Model name (K, M)	1 1 (1, 1)	1 2 (1, 2)	1 3 (1, 3)	1 4 (1, 4)	2 1 (2, 1)	2 2 (2, 2)	3 1 (3, 1)	4 1 (4, 1)
Logic formula	CDKN2	CDKN2&¬STK11	CDKN2&¬FBXW7& ¬STK11	CDKN2&¬EGFR& ¬FBXW7&¬STK11	CDKN2 | MAP2K	[MYC & PIK3C] | [CDKN2&¬FBXW7]	CDKN2 | MAP2K | PTEN	CDKN2 | KRAS | MET | PTEN
TP	38	38	38	38	40	40	49	54
FP	215	201	191	182	222	211	255	299
FN	39	39	39	39	37	37	28	23
TN	365	379	389	398	358	369	325	281
Specificity	0.63	0.65	0.67	0.69	0.62	0.64	0.56	0.48
Precision	0.15	0.16	0.17	0.17	0.15	0.16	0.16	0.15
Recall	0.49	0.49	0.49	0.49	0.52	0.52	0.64	0.7

ID:182 Obatoclax Mesylate −> BCL2, BCL2L1, MCL1

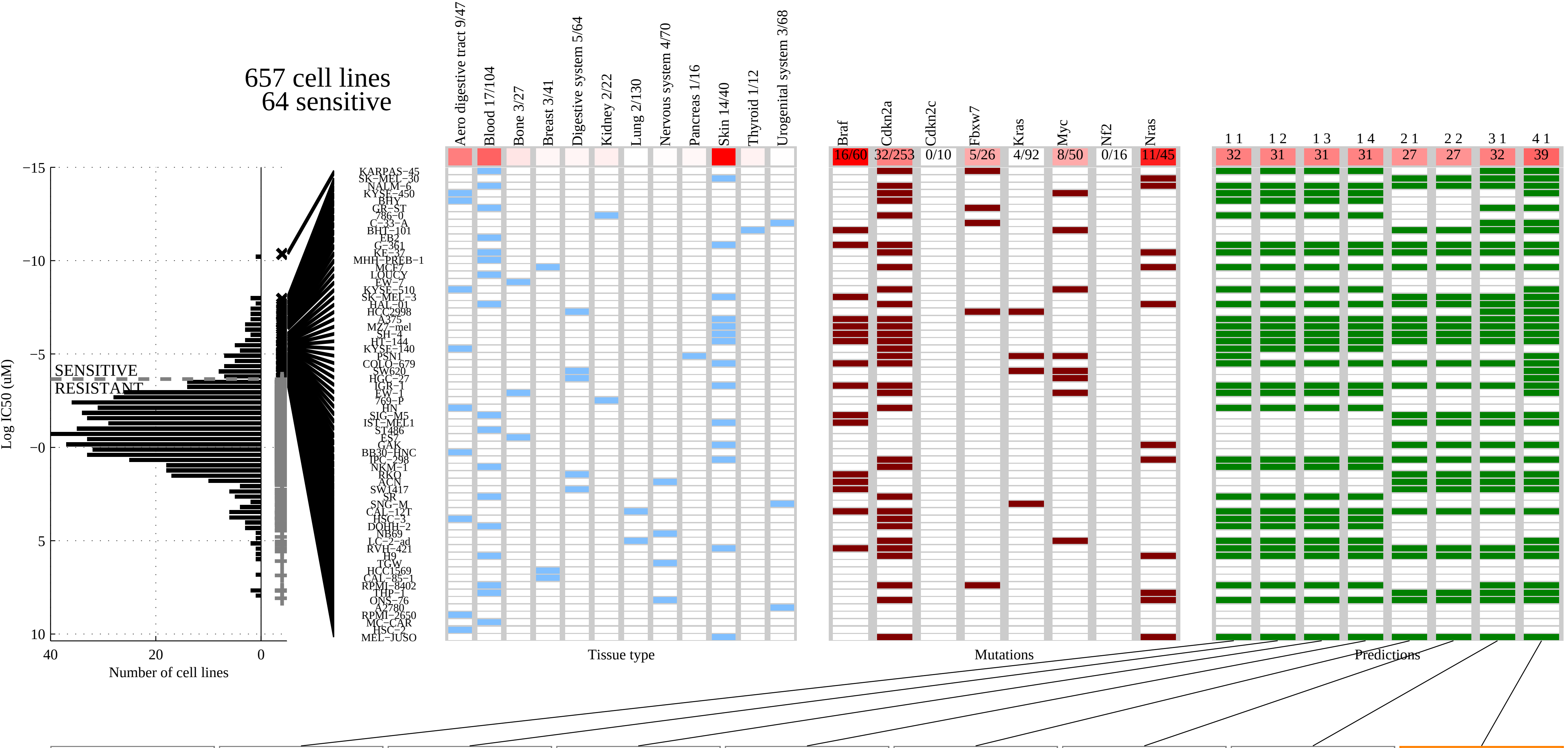

Model name	1 1	1 2	1 3	1 4	2 1	2 2	3 1	4 1
K / M	1 / 1	1 / 2	1 / 3	1 / 4	2 / 1	2 / 2	3 / 1	4 / 1
Logic formula	CDKN2	CDKN2&¬KRAS	CDKN2&¬KRAS&¬NF2	CDKN2&¬CDKN2&¬KRAS&¬NF2	BRAF | NRAS	[BRAF &¬KRAS] | [¬MYC & NRAS]	BRAF | FBXW7 | NRAS	BRAF | FBXW7 | MYC | NRAS
TP / FP	32 / 221	31 / 186	31 / 176	31 / 170	27 / 75	27 / 69	32 / 93	39 / 130
FN / TN	32 / 372	33 / 407	33 / 417	33 / 423	37 / 518	37 / 524	32 / 500	25 / 463
Specificity	0.63	0.69	0.7	0.71	0.87	0.88	0.84	0.78
Precision	0.13	0.14	0.15	0.15	0.26	0.28	0.26	0.23
Recall	0.5	0.48	0.48	0.48	0.42	0.42	0.5	0.61

ID:184 BMS−754807 -> IGF1R

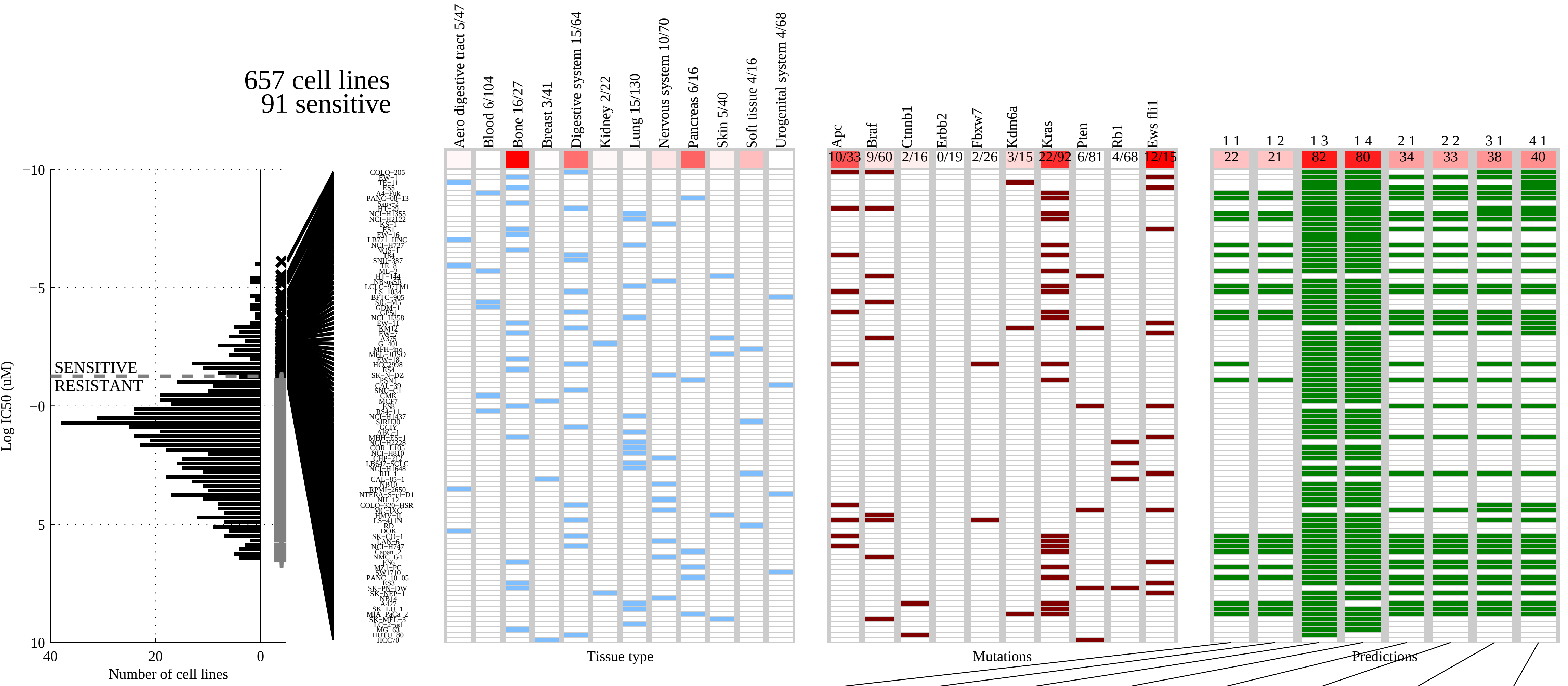

Model name (K, M)	1 1 (1, 1)	1 2 (1, 2)	1 3 (1, 3)	1 4 (1, 4)	2 1 (2, 1)	2 2 (2, 2)	3 1 (3, 1)	4 1 (4, 1)
Logic formula	KRAS	¬FBXW7 & KRAS	¬ERBB2 & ¬PTEN & ¬RB1	¬CTNNB & ¬ERBB2 & ¬PTEN & ¬RB1	KRAS | EWS F	[¬FBXW7 & KRAS] | [¬BRAF & EWS F]	APC | KRAS | EWS F	APC | KDM6A | KRAS | EWS F
TP, FP / FN, TN	22, 70 / 69, 496	21, 60 / 70, 506	82, 423 / 9, 143	80, 410 / 11, 156	34, 73 / 57, 493	33, 62 / 58, 504	38, 86 / 53, 480	40, 96 / 51, 470
Specificity	0.88	0.89	0.25	0.28	0.87	0.89	0.85	0.83
Precision	0.24	0.26	0.16	0.16	0.32	0.35	0.31	0.29
Recall	0.24	0.23	0.9	0.88	0.37	0.36	0.42	0.44

ID:185 OSI−906 -> IGF1R

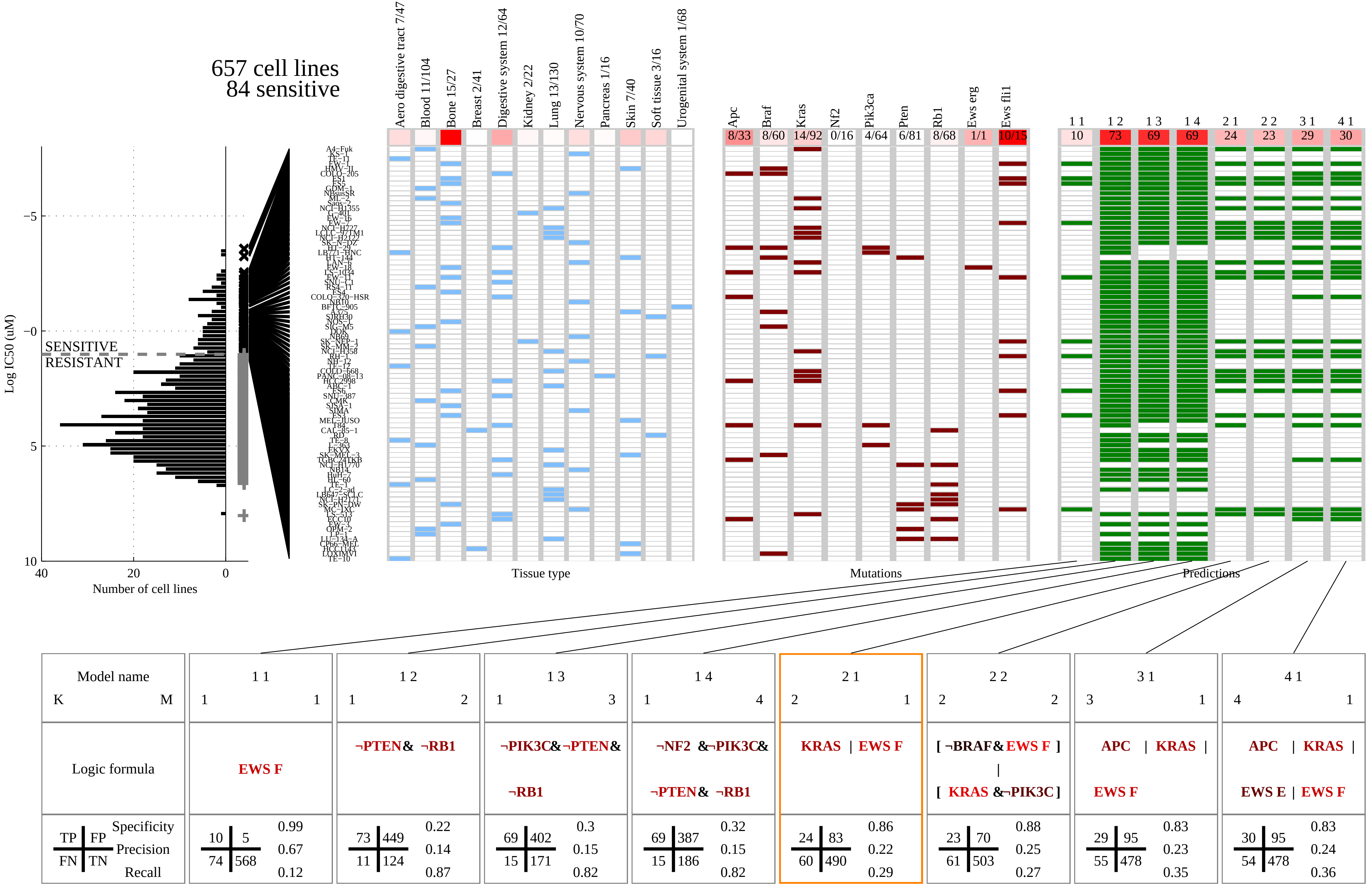

ID:186 Bexarotene –> Retinioic acid X family agonist

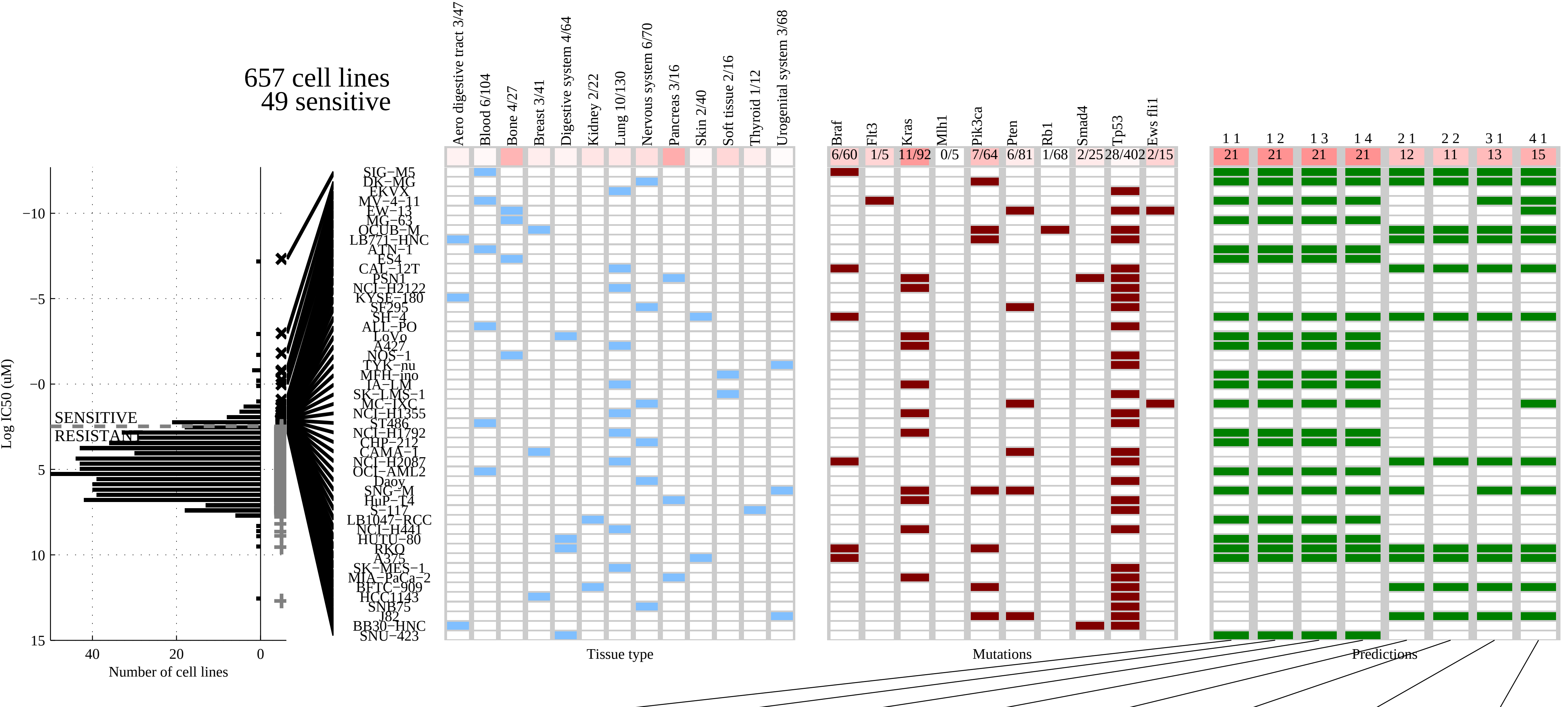

Model name (K, M)	1 1 (1, 1)	1 2 (1, 2)	1 3 (1, 3)	1 4 (1, 4)	2 1 (2, 1)	2 2 (2, 2)	3 1 (3, 1)	4 1 (4, 1)
Logic formula	¬TP53	¬RB1 & ¬TP53	¬RB1 & ¬SMAD4 & ¬TP53	¬MLH1 & ¬RB1 & ¬SMAD4 & ¬TP53	BRAF | PIK3C	[¬KRAS & PIK3C] | [BRAF & ¬PTEN]	BRAF | FLT3 | PIK3C	BRAF | FLT3 | PIK3C | EWS F
TP FP / FN TN	21 234 / 28 374	21 219 / 28 389	21 212 / 28 396	21 208 / 28 400	12 109 / 37 499	11 83 / 38 525	13 112 / 36 496	15 124 / 34 484
Specificity	0.62	0.64	0.65	0.66	0.82	0.86	0.82	0.8
Precision	0.082	0.087	0.09	0.092	0.099	0.12	0.1	0.11
Recall	0.43	0.43	0.43	0.43	0.24	0.22	0.27	0.31

ID:189 Pyrvinium Pamoate −> Hh/Wnt Antagonist

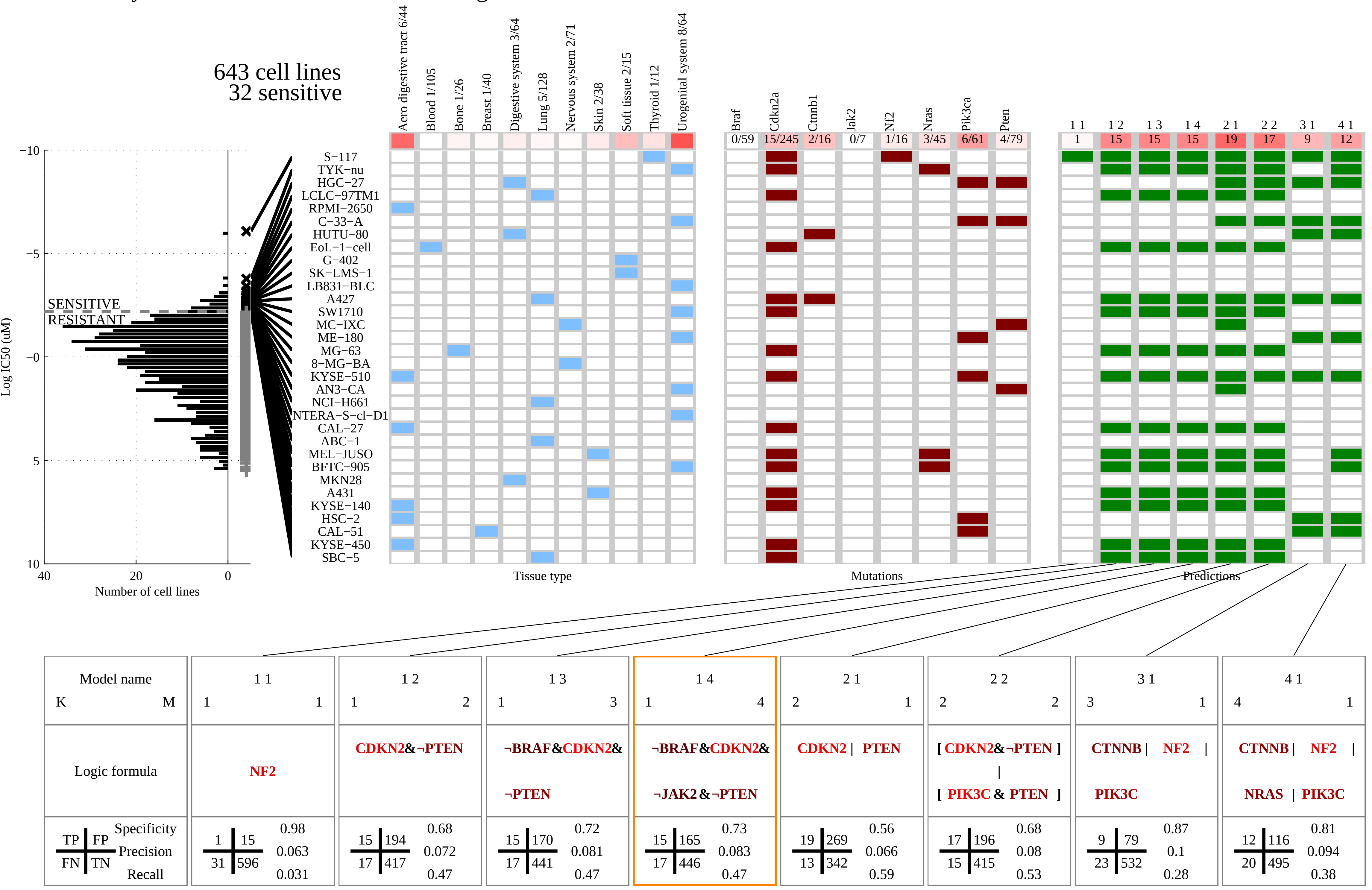

ID:190 Bleomycin −> DNA damage

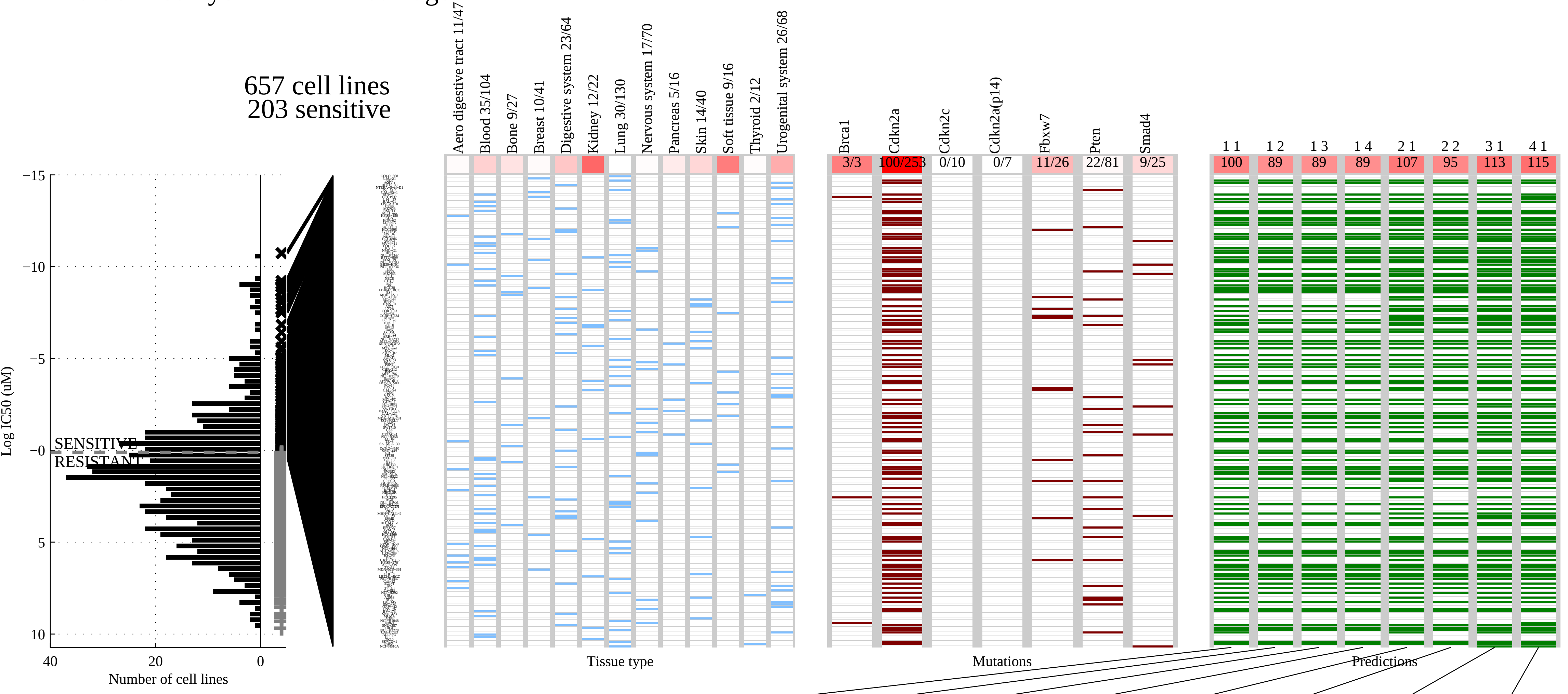

Model name	1 1	1 2	1 3	1 4	2 1	2 2	3 1	4 1
K / M	1 / 1	1 / 2	1 / 3	1 / 4	2 / 1	2 / 2	3 / 1	4 / 1
Logic formula	**CDKN2**	**CDKN2&¬PTEN**	**CDKN2&¬CDKN2& ¬PTEN**	**CDKN2&¬CDKN2& ¬CDKN2&¬PTEN**	**CDKN2 | FBXW7**	**[FBXW7&¬PTEN] | [CDKN2&¬PTEN]**	**CDKN2 | FBXW7 | SMAD4**	**BRCA1 | CDKN2 | FBXW7 | SMAD4**
TP FP / FN TN	100 153 / 103 301	89 126 / 114 328	89 121 / 114 333	89 116 / 114 338	107 162 / 96 292	95 133 / 108 321	113 167 / 90 287	115 167 / 88 287
Specificity	0.66	0.72	0.73	0.74	0.64	0.71	0.63	0.63
Precision	0.4	0.41	0.42	0.43	0.4	0.42	0.4	0.41
Recall	0.49	0.44	0.44	0.44	0.53	0.47	0.56	0.57

ID:192 LFM−A13 −> BTK

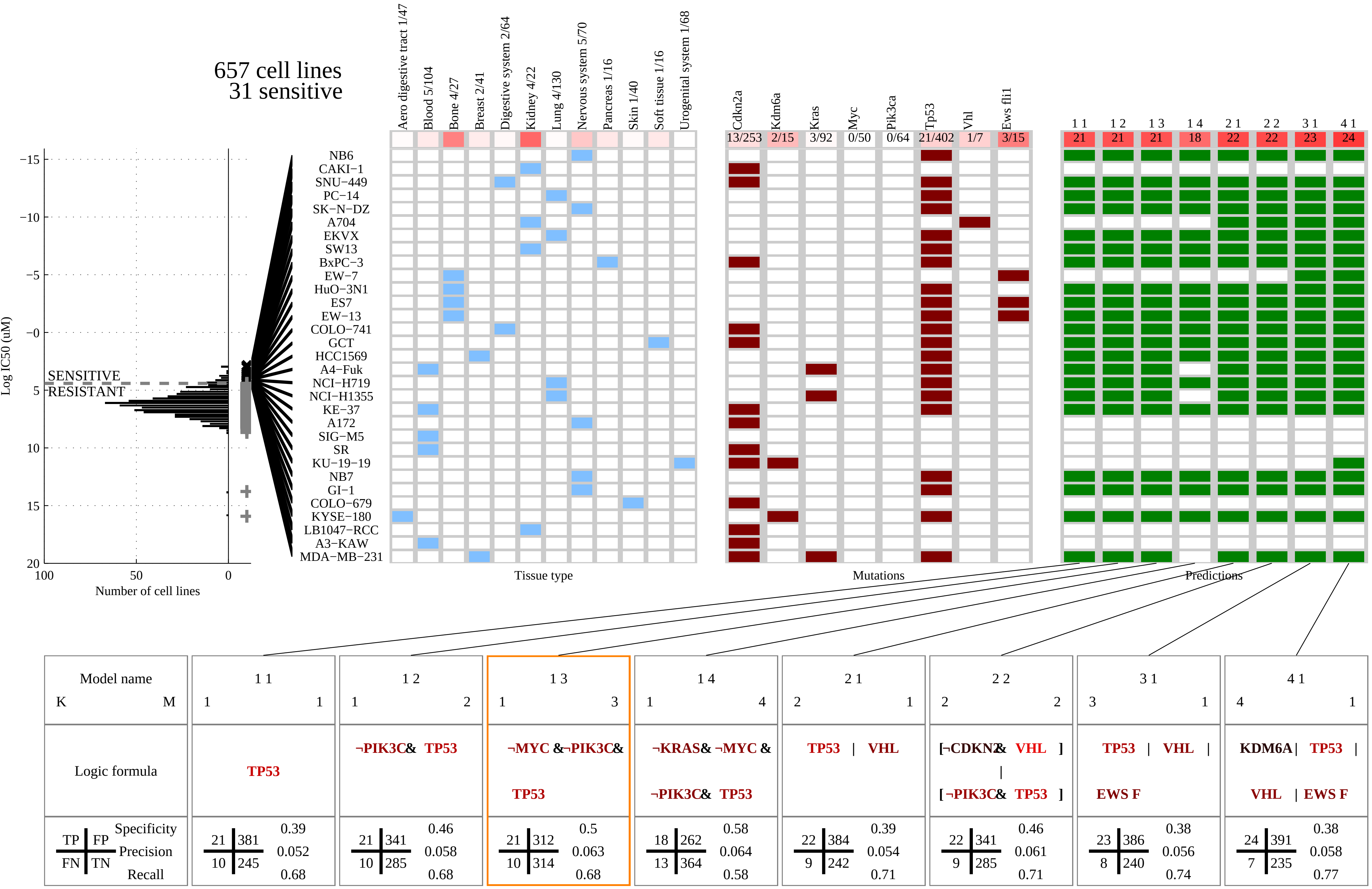

ID:194 AUY922 −> HSP90

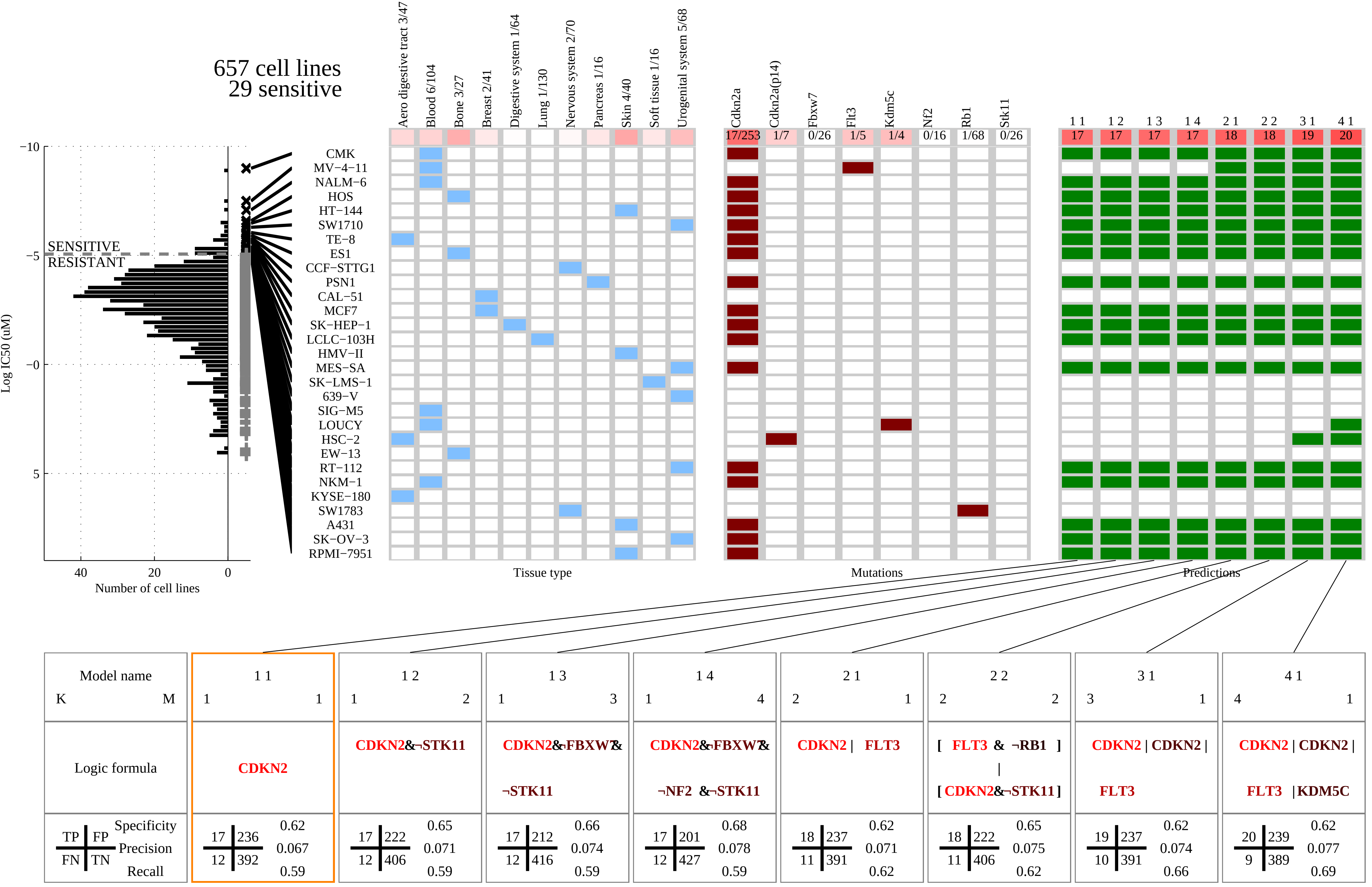

ID:195 Camptothecin -> Topisomerase 1

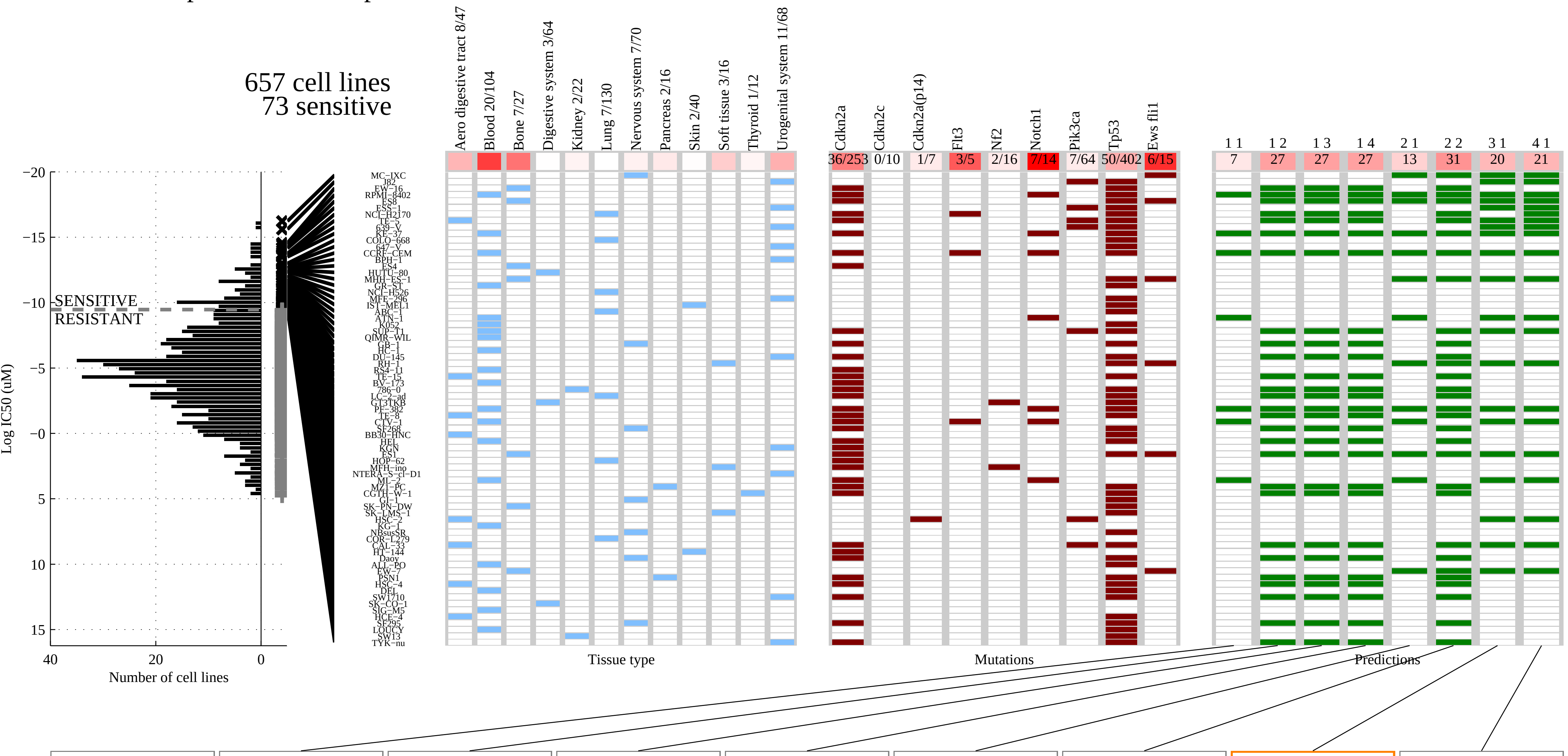

Model name	1 1	1 2	1 3	1 4	2 1	2 2	3 1	4 1
K / M	1 / 1	1 / 2	1 / 3	1 / 4	2 / 1	2 / 2	3 / 1	4 / 1
Logic formula	NOTCH	CDKN2 & TP53	CDKN2 & ¬CDKN2 & TP53	CDKN2 & ¬CDKN2 & ¬NF2 & TP53	NOTCH | EWS F	[¬CDKN2 & EWS F] | [CDKN2 & TP53]	NOTCH | PIK3C | EWS F	FLT3 | NOTCH | PIK3C | EWS F
TP FP	7 7	27 118	27 114	27 109	13 16	31 124	20 72	21 74
FN TN	66 577	46 466	46 470	46 475	60 568	42 460	53 512	52 510
Specificity	0.99	0.8	0.8	0.81	0.97	0.79	0.88	0.87
Precision	0.5	0.19	0.19	0.2	0.45	0.2	0.22	0.22
Recall	0.096	0.37	0.37	0.37	0.18	0.42	0.27	0.29

ID:197 Bryostatin 1 –> PRKC

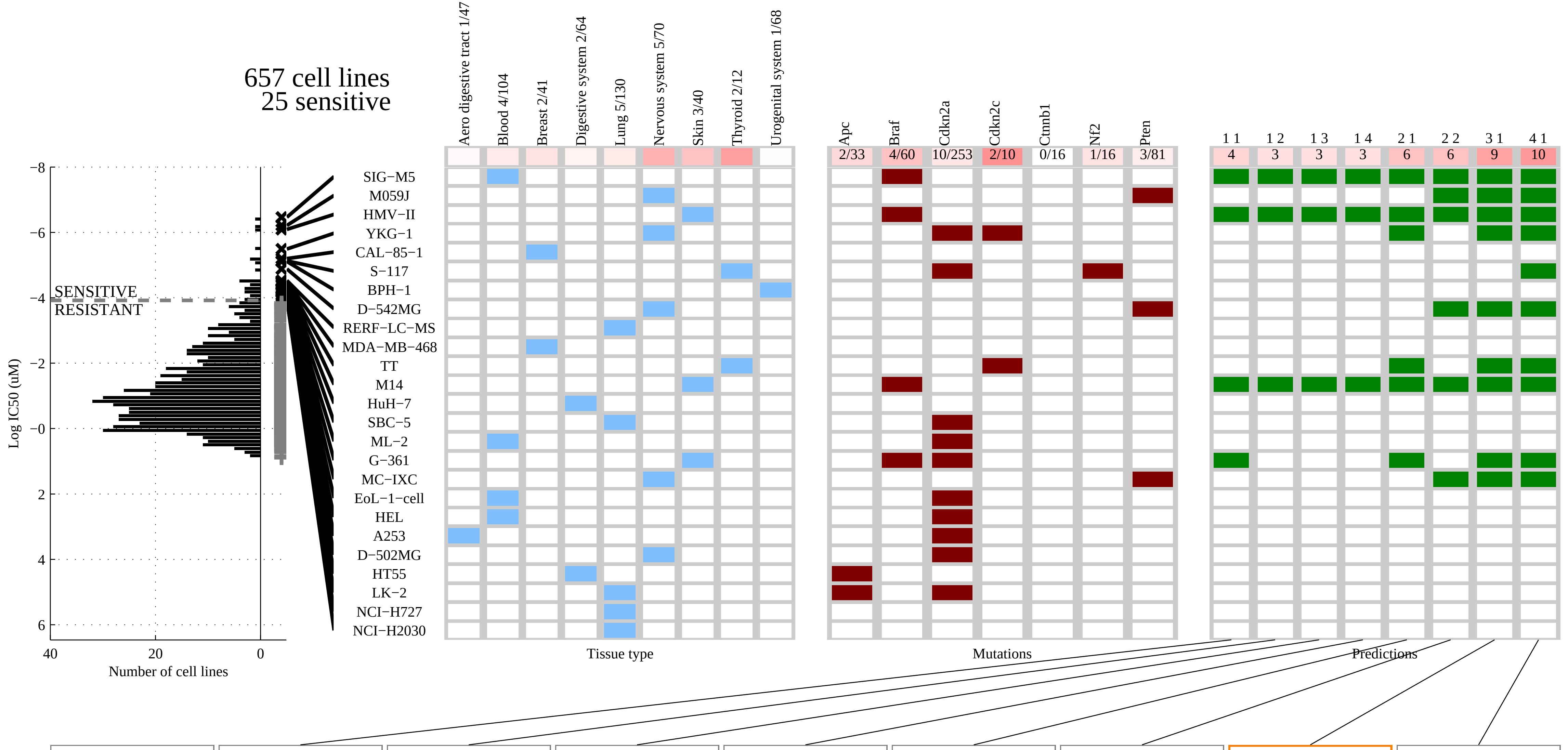

Model name	1 1	1 2	1 3	1 4	2 1	2 2	3 1	4 1
K / M	1 / 1	1 / 2	1 / 3	1 / 4	2 / 1	2 / 2	3 / 1	4 / 1
Logic formula	BRAF	BRAF &¬CDKN2	¬APC & BRAF & ¬CDKN2	¬APC & BRAF & ¬CDKN2&¬CTNNB	BRAF | CDKN2	[¬CDKN2& PTEN] | [BRAF &¬CDKN2]	BRAF | CDKN2 | PTEN	BRAF | CDKN2 | NF2 | PTEN
TP FP / FN TN	4 56 / 21 576	3 20 / 22 612	3 15 / 22 617	3 13 / 22 619	6 64 / 19 568	6 59 / 19 573	9 126 / 16 506	10 138 / 15 494
Specificity	0.91	0.97	0.98	0.98	0.9	0.91	0.8	0.78
Precision	0.067	0.13	0.17	0.19	0.086	0.092	0.067	0.068
Recall	0.16	0.12	0.12	0.12	0.24	0.24	0.36	0.4

ID:199 Pazopanib −> VEGFR, PDGFRA, PDGFRB, KIT

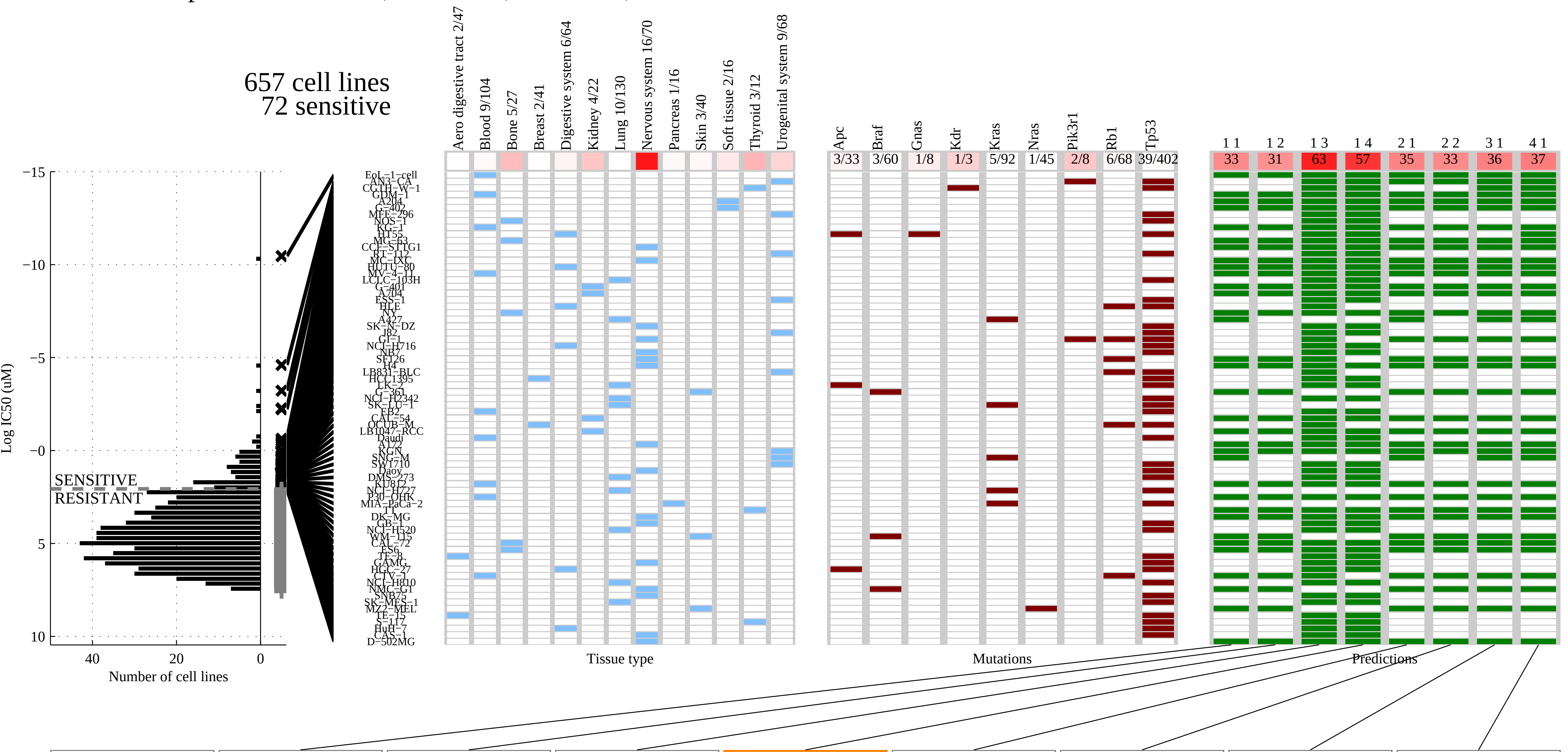

Model name (K, M)	1 1 (1, 1)	1 2 (1, 2)	1 3 (1, 3)	1 4 (1, 4)	2 1 (2, 1)	2 2 (2, 2)	3 1 (3, 1)	4 1 (4, 1)
Logic formula	¬TP53	¬KRAS& ¬TP53	¬BRAF&¬KRAS& ¬NRAS	¬BRAF&¬KRAS& ¬NRAS& ¬RB1	PIK3R | ¬TP53	[¬APC & PIK3R] | [¬KRAS& ¬TP53]	KDR | PIK3R | ¬TP53	GNAS | KDR | PIK3R | ¬TP53
TP FP	33 222	31 193	63 403	57 349	35 225	33 195	36 227	37 233
FN TN	39 363	41 392	9 182	15 236	37 360	39 390	36 358	35 352
Specificity	0.62	0.67	0.31	0.4	0.62	0.67	0.61	0.6
Precision	0.13	0.14	0.14	0.14	0.13	0.14	0.14	0.14
Recall	0.46	0.43	0.88	0.79	0.49	0.46	0.5	0.51

ID:20 Geldanamycin -> Hsp90

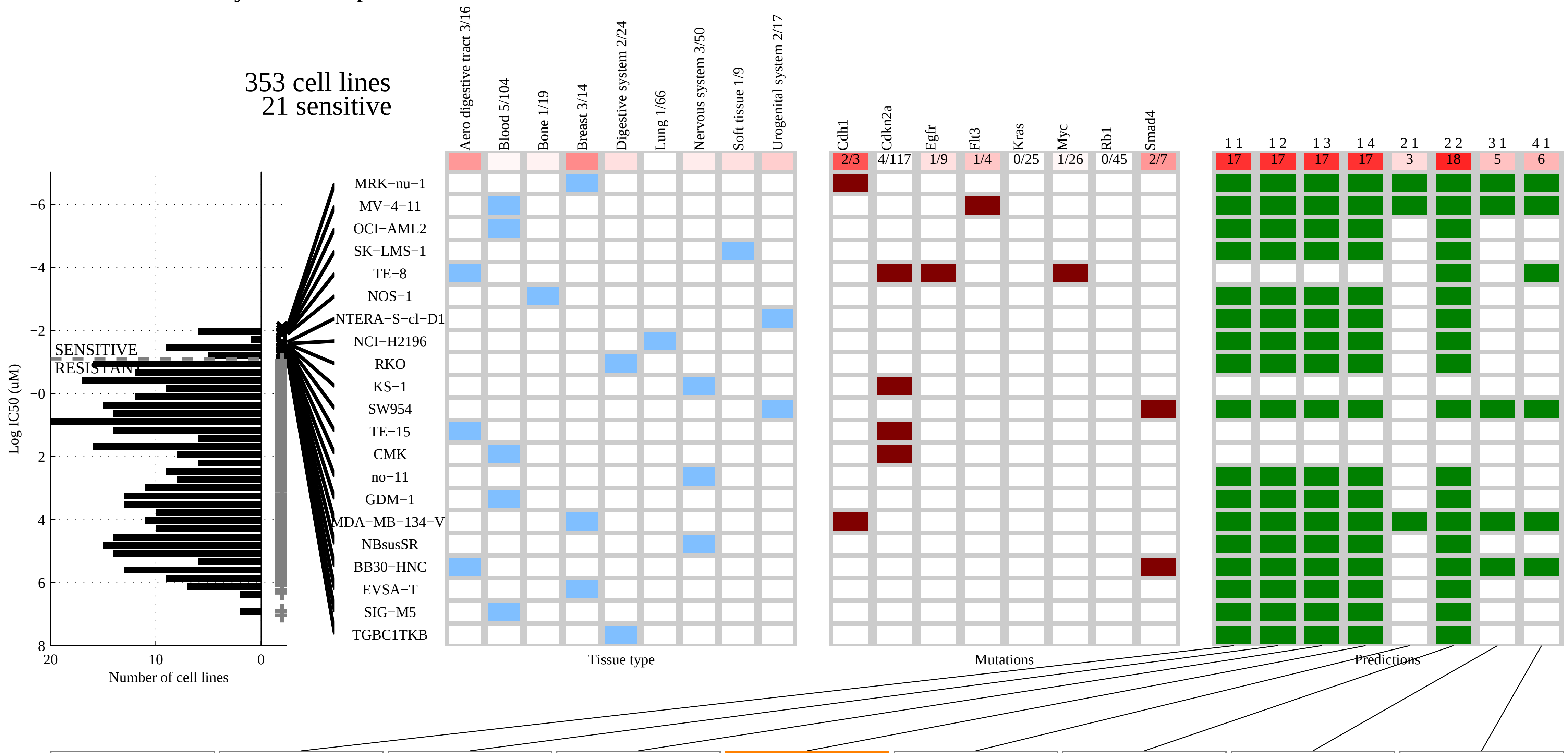

Model name	1 1	1 2	1 3	1 4	2 1	2 2	3 1	4 1
K / M	1 / 1	1 / 2	1 / 3	1 / 4	2 / 1	2 / 2	3 / 1	4 / 1
Logic formula	**¬CDKN2**	**¬CDKN2 & ¬RB1**	**¬CDKN2 & ¬KRAS & ¬RB1**	**¬CDKN2 & ¬KRAS & ¬MYC & ¬RB1**	**CDH1 | FLT3**	**[EGFR & MYC] | [¬CDKN2 & ¬RB1]**	**CDH1 | FLT3 | SMAD4**	**CDH1 | EGFR | FLT3 | SMAD4**
TP / FP	17 / 219	17 / 181	17 / 165	17 / 156	3 / 4	18 / 182	5 / 9	6 / 17
FN / TN	4 / 113	4 / 151	4 / 167	4 / 176	18 / 328	3 / 150	16 / 323	15 / 315
Specificity	0.34	0.45	0.5	0.53	0.99	0.45	0.97	0.95
Precision	0.072	0.086	0.093	0.098	0.43	0.09	0.36	0.26
Recall	0.81	0.81	0.81	0.81	0.14	0.86	0.24	0.29

ID:200 LAQ824 -> HDAC

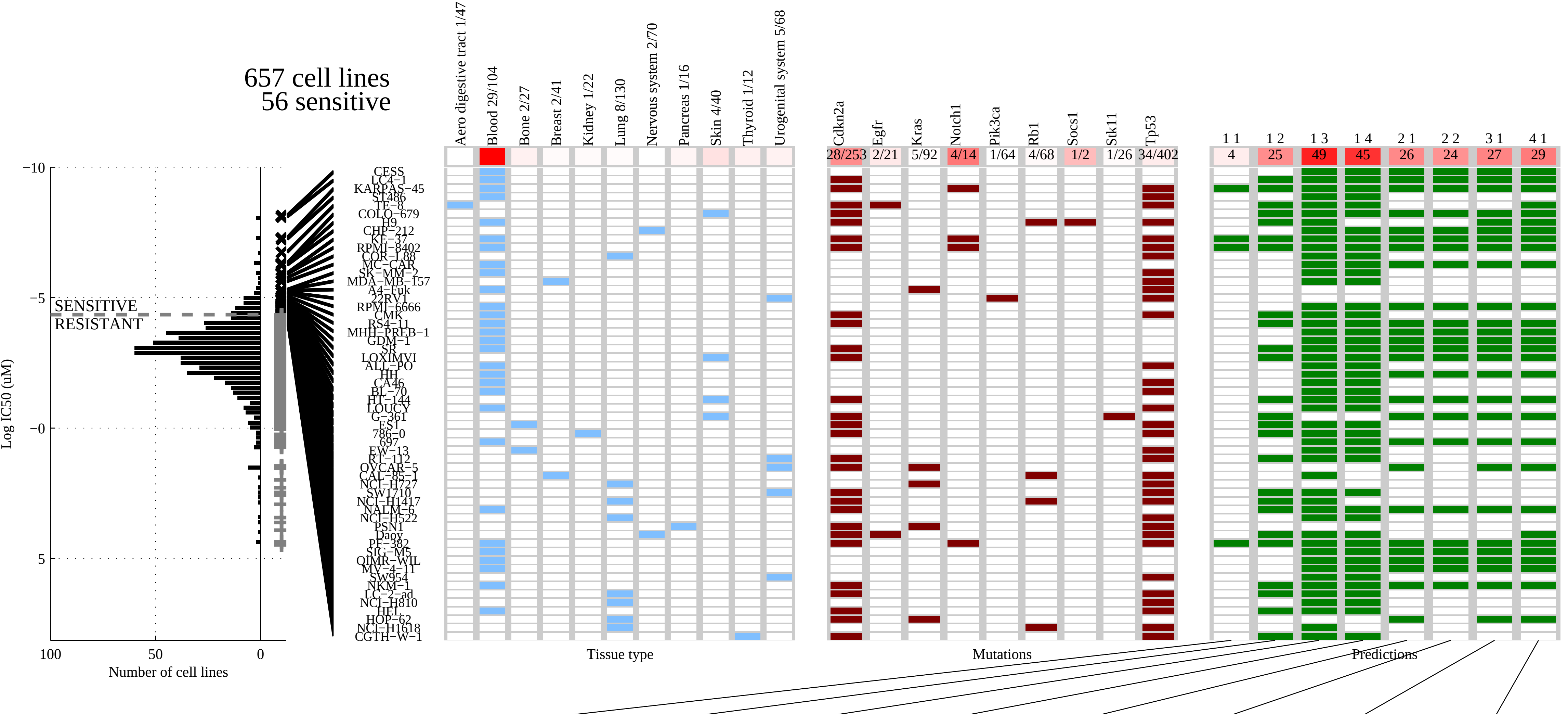

Model name (K, M)	1 1 (1, 1)	1 2 (1, 2)	1 3 (1, 3)	1 4 (1, 4)	2 1 (2, 1)	2 2 (2, 2)	3 1 (3, 1)	4 1 (4, 1)
Logic formula	NOTCH	CDKN2&¬KRAS	¬KRAS&¬PIK3C& ¬STK11	¬KRAS&¬PIK3C& ¬RB1 &¬STK11	NOTCH| ¬TP53	[¬KRAS&NOTCH] | [¬KRAS& ¬TP53]	NOTCH| SOCS1 | ¬TP53	EGFR |NOTCH| SOCS1 | ¬TP53
TP FP / FN TN	4 10 / 52 591	25 192 / 31 409	49 448 / 7 153	45 399 / 11 202	26 239 / 30 362	24 209 / 32 392	27 240 / 29 361	29 253 / 27 348
Specificity	0.98	0.68	0.25	0.34	0.6	0.65	0.6	0.58
Precision	0.29	0.12	0.099	0.1	0.098	0.1	0.1	0.1
Recall	0.071	0.45	0.88	0.8	0.46	0.43	0.48	0.52

ID:201 Epothilone B –> Microtubules

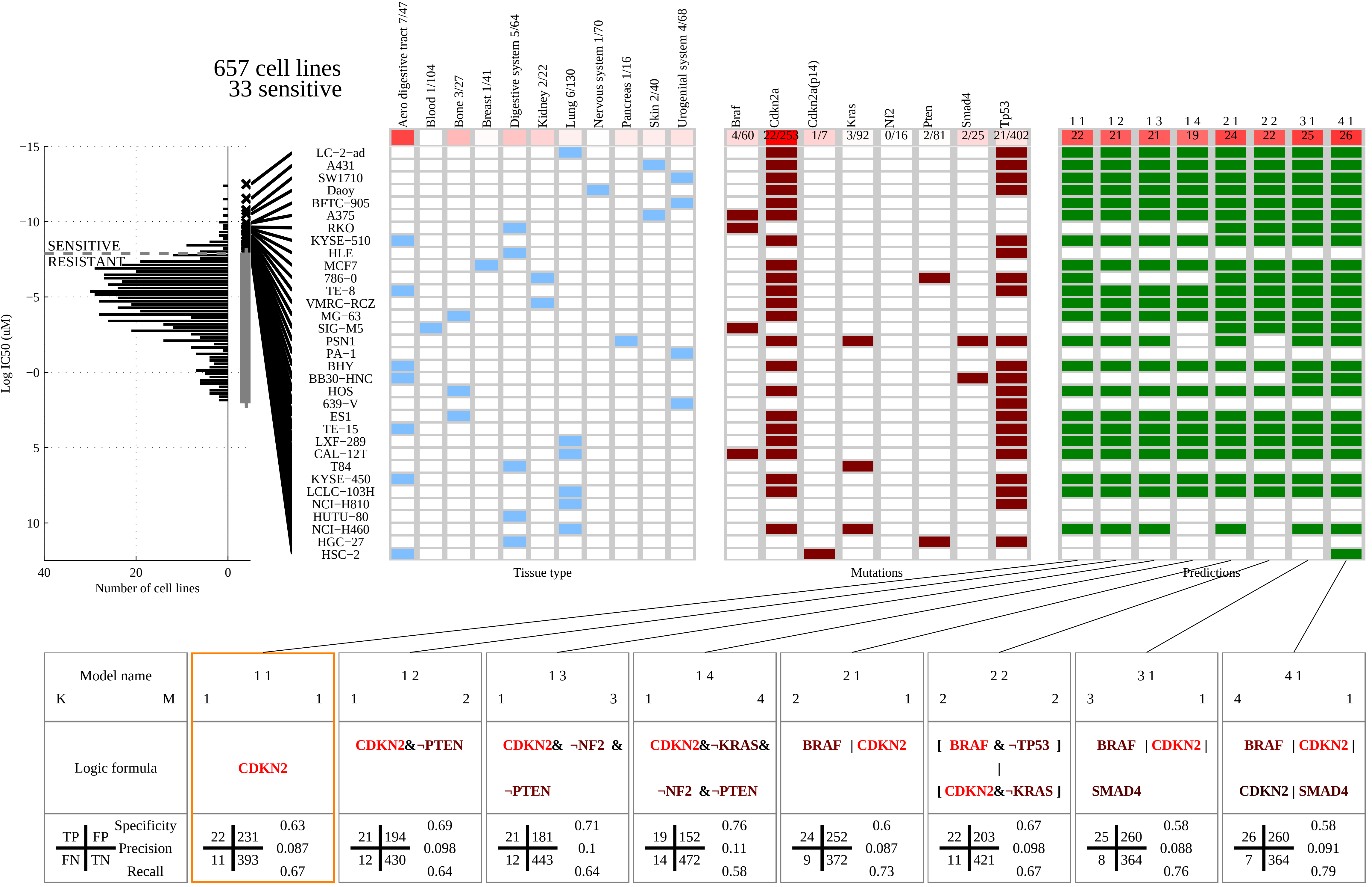

ID:202 GSK−1904529A −> IGF1R

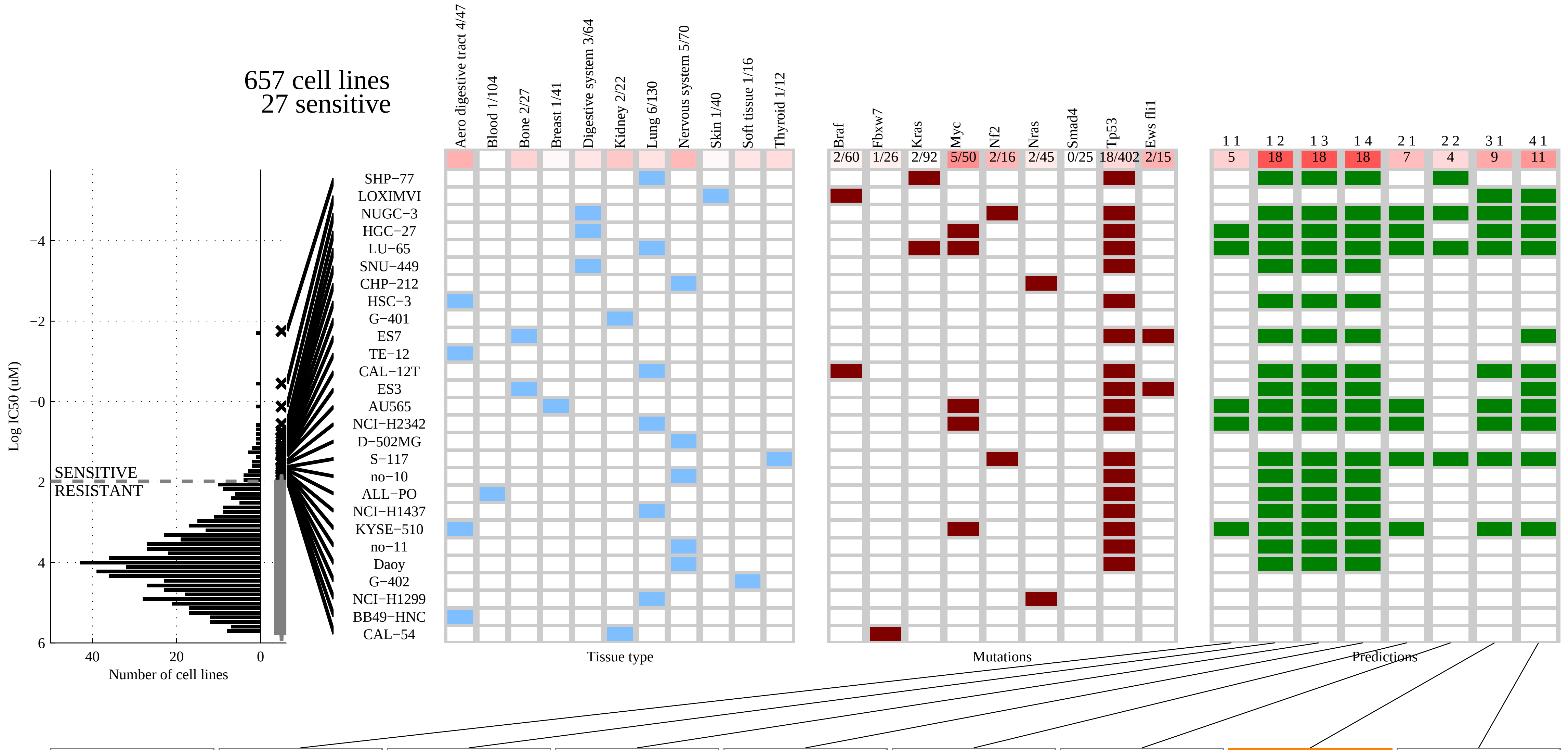

Model name	1 1	1 2	1 3	1 4	2 1	2 2	3 1	4 1
K / M	1 / 1	1 / 2	1 / 3	1 / 4	2 / 1	2 / 2	3 / 1	4 / 1
Logic formula	MYC	¬NRAS & TP53	¬FBXW7 & ¬NRAS & TP53	¬FBXW7 & ¬NRAS & ¬SMAD4 & TP53	MYC | NF2	[NF2 & TP53] | [KRAS & TP53]	BRAF | MYC | NF2	BRAF | MYC | NF2 | EWS F
TP / FP	5 / 45	18 / 362	18 / 342	18 / 324	7 / 58	4 / 62	9 / 110	11 / 120
FN / TN	22 / 585	9 / 268	9 / 288	9 / 306	20 / 572	23 / 568	18 / 520	16 / 510
Specificity	0.93	0.43	0.46	0.49	0.91	0.9	0.83	0.81
Precision	0.1	0.047	0.05	0.053	0.11	0.061	0.076	0.084
Recall	0.19	0.67	0.67	0.67	0.26	0.15	0.33	0.41

ID:203 BMS−345541 −> IKBKB

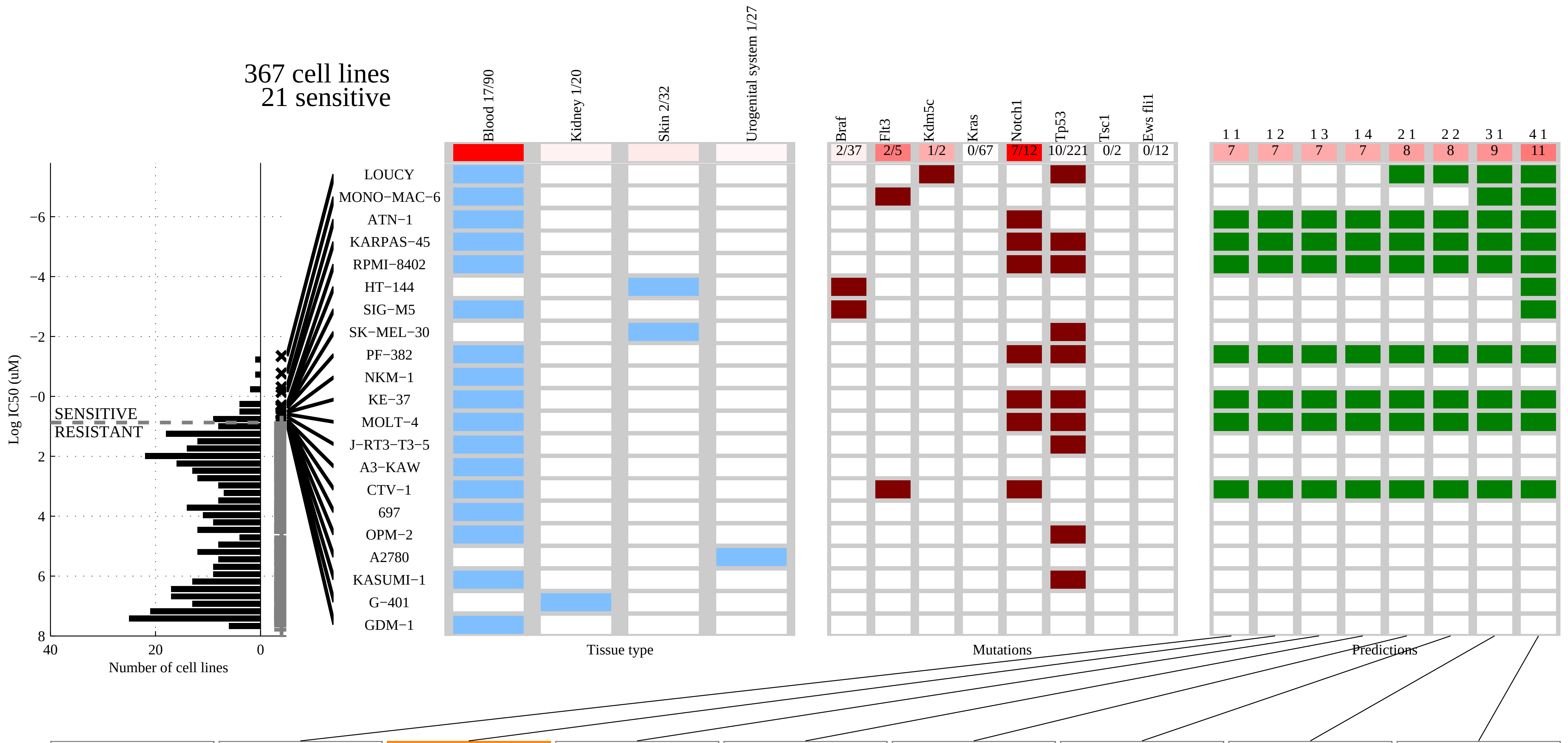

Model name<br>K M	1 1<br>1 1	1 2<br>1 2	1 3<br>1 3	1 4<br>1 4	2 1<br>2 1	2 2<br>2 2	3 1<br>3 1	4 1<br>4 1
Logic formula	NOTCH	¬KRAS&NOTCH	¬KRAS&NOTCH& ¬TSC1	¬KRAS&NOTCH& ¬TSC1 &¬EWS F	KDM5C | NOTCH	[¬KRAS&NOTCH] | [KDM5C& TP53]	FLT3 | KDM5C | NOTCH	BRAF | FLT3 | KDM5C | NOTCH
TP FP / FN TN	7 5 / 14 341	7 2 / 14 344	7 1 / 14 345	7 1 / 14 345	8 6 / 13 340	8 2 / 13 344	9 8 / 12 338	11 42 / 10 304
Specificity	0.99	0.99	1	1	0.98	0.99	0.98	0.88
Precision	0.58	0.78	0.88	0.88	0.57	0.8	0.53	0.21
Recall	0.33	0.33	0.33	0.33	0.38	0.38	0.43	0.52

ID:204 Tipifarnib –> Farnesyl–transferase (FNTA)

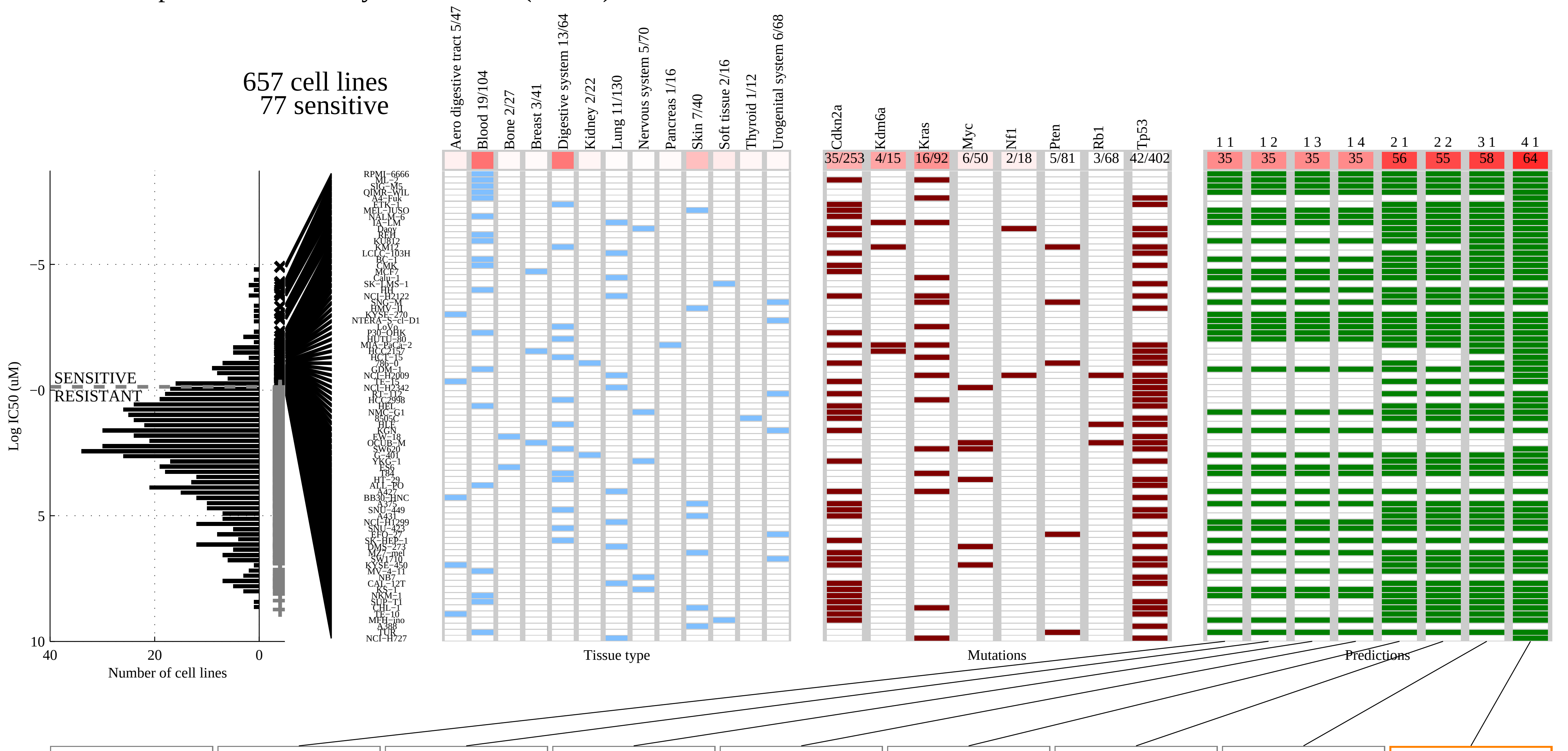

<table>
<tr><td>Model name
K M</td><td>1 1
1 1</td><td>1 2
1 2</td><td>1 3
1 3</td><td>1 4
1 4</td><td>2 1
2 1</td><td>2 2
2 2</td><td>3 1
3 1</td><td>4 1
4 1</td></tr>
<tr><td>Logic formula</td><td>¬TP53</td><td>¬RB1 & ¬TP53</td><td>¬MYC & ¬RB1 & ¬TP53</td><td>¬MYC & ¬NF1 & ¬RB1 & ¬TP53</td><td>CDKN2 | ¬TP53</td><td>[¬RB1 & ¬TP53] | [CDKN2&¬PTEN]</td><td>CDKN2 | KDM6A | ¬TP53</td><td>CDKN2 | KDM6A | KRAS | ¬TP53</td></tr>
<tr><td>TP FP / FN TN
Specificity
Precision
Recall</td><td>35 220 / 42 360
0.62
0.14
0.45</td><td>35 205 / 42 375
0.65
0.15
0.45</td><td>35 192 / 42 388
0.67
0.15
0.45</td><td>35 185 / 42 395
0.68
0.16
0.45</td><td>56 344 / 21 236
0.41
0.14
0.73</td><td>55 308 / 22 272
0.47
0.15
0.71</td><td>58 347 / 19 233
0.4
0.14
0.75</td><td>64 379 / 13 201
0.35
0.14
0.83</td></tr>
</table>

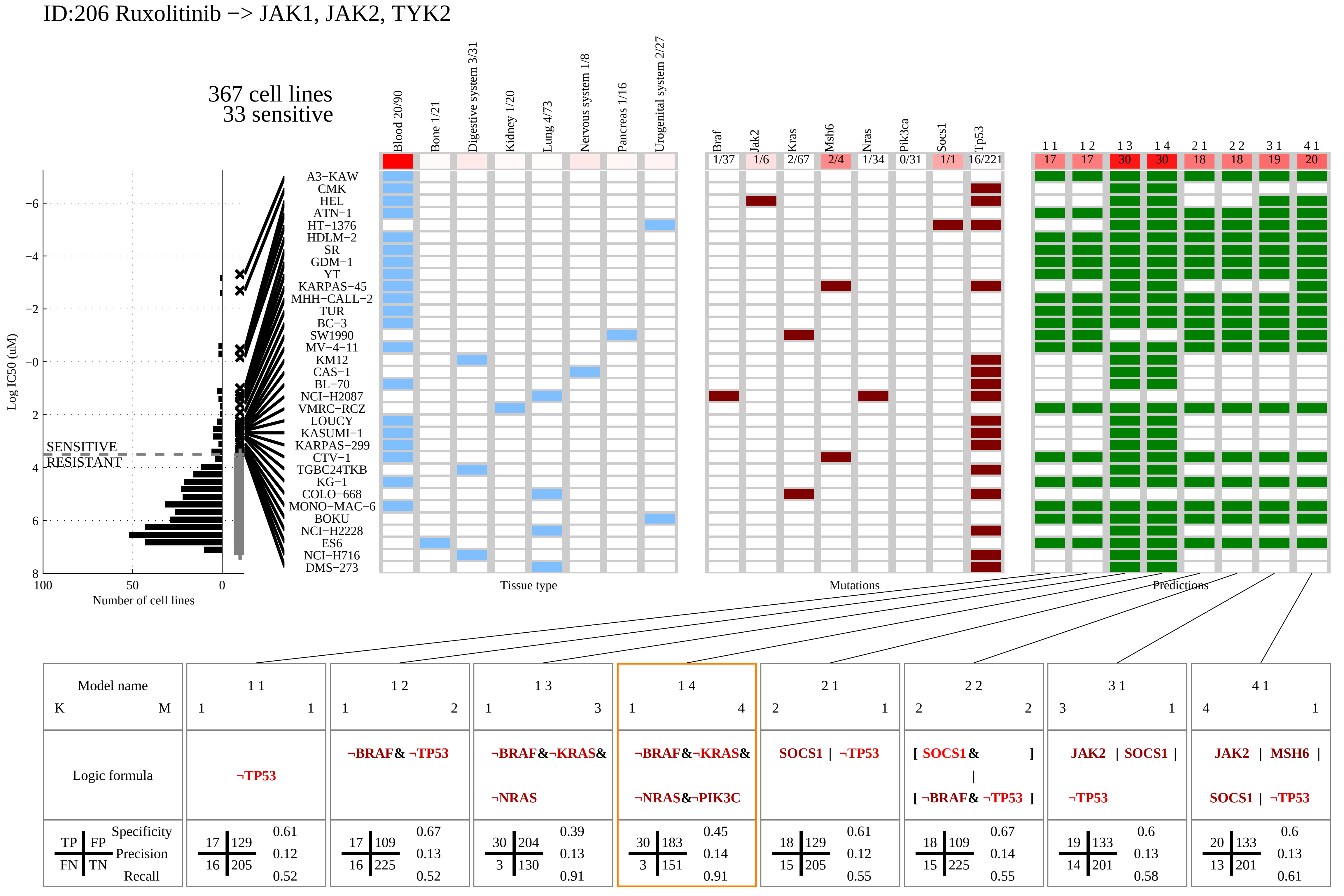
ID:206 Ruxolitinib −> JAK1, JAK2, TYK2
367 cell lines
33 sensitive
Log IC50 (uM)
Number of cell lines
SENSITIVE
RESISTANT
Blood 20/90
Bone 1/21
Digestive system 3/31
Kidney 1/20
Lung 4/73
Nervous system 1/8
Pancreas 1/16
Urogenital system 2/27
Braf 1/37
Jak2 1/6
Kras 2/67
Msh6 2/4
Nras 1/34
Pik3ca 0/31
Socs1 1/1
Tp53 16/221
1 1 17
1 2 17
1 3 30
1 4 30
2 1 18
2 2 18
3 1 19
4 1 20
A3−KAW
CMK
HEL
ATN−1
HT−1376
HDLM−2
SR
GDM−1
YT
KARPAS−45
MHH−CALL−2
TUR
BC−3
SW1990
MV−4−11
KM12
CAS−1
BL−70
NCI−H2087
VMRC−RCZ
LOUCY
KASUMI−1
KARPAS−299
CTV−1
TGBC24TKB
KG−1
COLO−668
MONO−MAC−6
BOKU
NCI−H2228
ES6
NCI−H716
DMS−273
Tissue type
Mutations
Predictions
Model name
K M
Logic formula
TP FP
FN TN
Specificity
Precision
Recall
1 1: K 1, M 1; ¬TP53; TP 17, FP 129, FN 16, TN 205; 0.61, 0.12, 0.52
1 2: K 1, M 2; ¬BRAF & ¬TP53; TP 17, FP 109, FN 16, TN 225; 0.67, 0.13, 0.52
1 3: K 1, M 3; ¬BRAF & ¬KRAS & ¬NRAS; TP 30, FP 204, FN 3, TN 130; 0.39, 0.13, 0.91
1 4: K 1, M 4; ¬BRAF & ¬KRAS & ¬NRAS & ¬PIK3C; TP 30, FP 183, FN 3, TN 151; 0.45, 0.14, 0.91
2 1: K 2, M 1; SOCS1 | ¬TP53; TP 18, FP 129, FN 15, TN 205; 0.61, 0.12, 0.55
2 2: K 2, M 2; [SOCS1 &] | [¬BRAF & ¬TP53]; TP 18, FP 109, FN 15, TN 225; 0.67, 0.14, 0.55
3 1: K 3, M 1; JAK2 | SOCS1 | ¬TP53; TP 19, FP 133, FN 14, TN 201; 0.6, 0.13, 0.58
4 1: K 4, M 1; JAK2 | MSH6 | SOCS1 | ¬TP53; TP 20, FP 133, FN 13, TN 201; 0.6, 0.13, 0.61

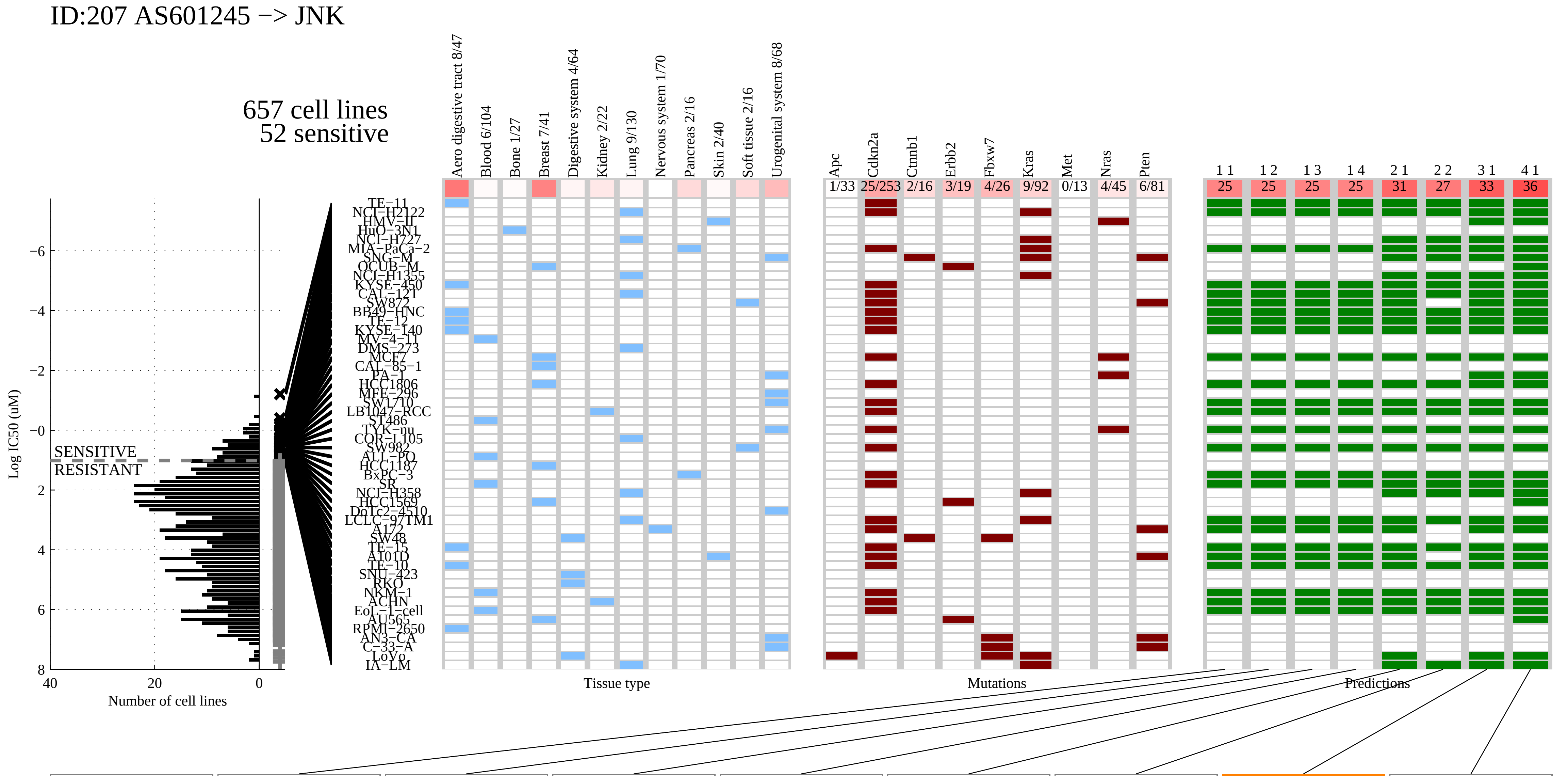

Model name (K, M)	1 1 (1, 1)	1 2 (1, 2)	1 3 (1, 3)	1 4 (1, 4)	2 1 (2, 1)	2 2 (2, 2)	3 1 (3, 1)	4 1 (4, 1)
Logic formula	CDKN2	CDKN2&¬FBXW7	CDKN2&¬FBXW7& ¬MET	CDKN2&¬CTNNB& ¬FBXW7& ¬MET	CDKN2 | KRAS	[CDKN2&¬PTEN] | [¬APC & KRAS]	CDKN2 | KRAS | NRAS	CDKN2 | ERBB2 | KRAS | NRAS
TP / FP	25 / 228	25 / 218	25 / 210	25 / 205	31 / 278	27 / 231	33 / 296	36 / 306
FN / TN	27 / 377	27 / 387	27 / 395	27 / 400	21 / 327	25 / 374	19 / 309	16 / 299
Specificity	0.62	0.64	0.65	0.66	0.54	0.62	0.51	0.49
Precision	0.099	0.1	0.11	0.11	0.1	0.1	0.1	0.11
Recall	0.48	0.48	0.48	0.48	0.6	0.52	0.63	0.69

ID:208 SB−715992 −> KIF11

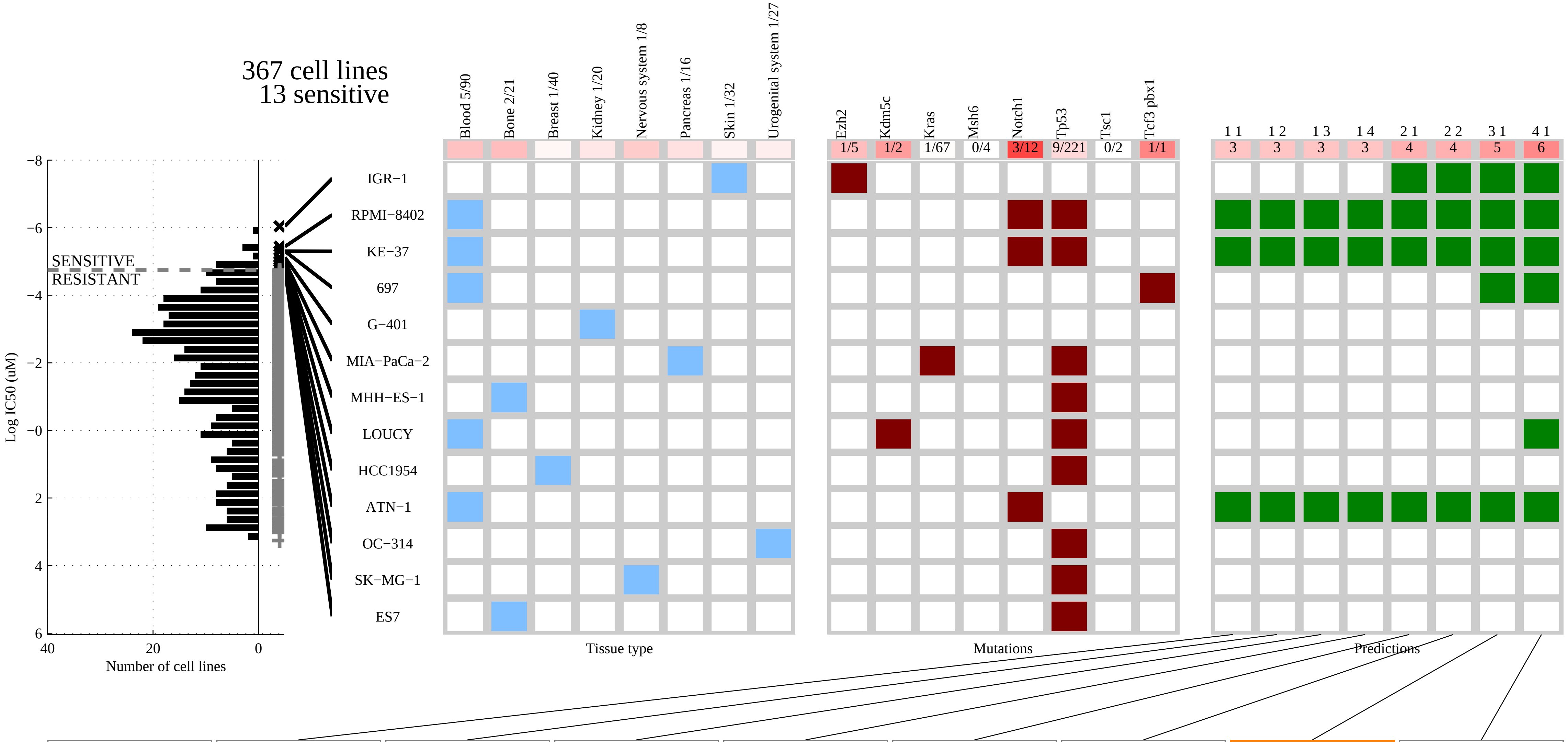

Model name (K, M)	1 1 (1, 1)	1 2 (1, 2)	1 3 (1, 3)	1 4 (1, 4)	2 1 (2, 1)	2 2 (2, 2)	3 1 (3, 1)	4 1 (4, 1)
Logic formula	NOTCH	¬KRAS&NOTCH	¬KRAS&¬MSH6& NOTCH	¬KRAS&¬MSH6& NOTCH& ¬TSC1	EZH2 |NOTCH	[¬KRAS&NOTCH] | [EZH2 & ¬TP53]	EZH2 |NOTCH| TCF3	EZH2 |KDM5C| NOTCH| TCF3
TP	3	3	3	3	4	4	5	6
FP	9	6	4	3	13	6	13	14
FN	10	10	10	10	9	9	8	7
TN	345	348	350	351	341	348	341	340
Specificity	0.97	0.98	0.99	0.99	0.96	0.98	0.96	0.96
Precision	0.25	0.33	0.43	0.5	0.24	0.4	0.28	0.3
Recall	0.23	0.23	0.23	0.23	0.31	0.31	0.38	0.46

ID:211 TL−2−105 -> CRAF

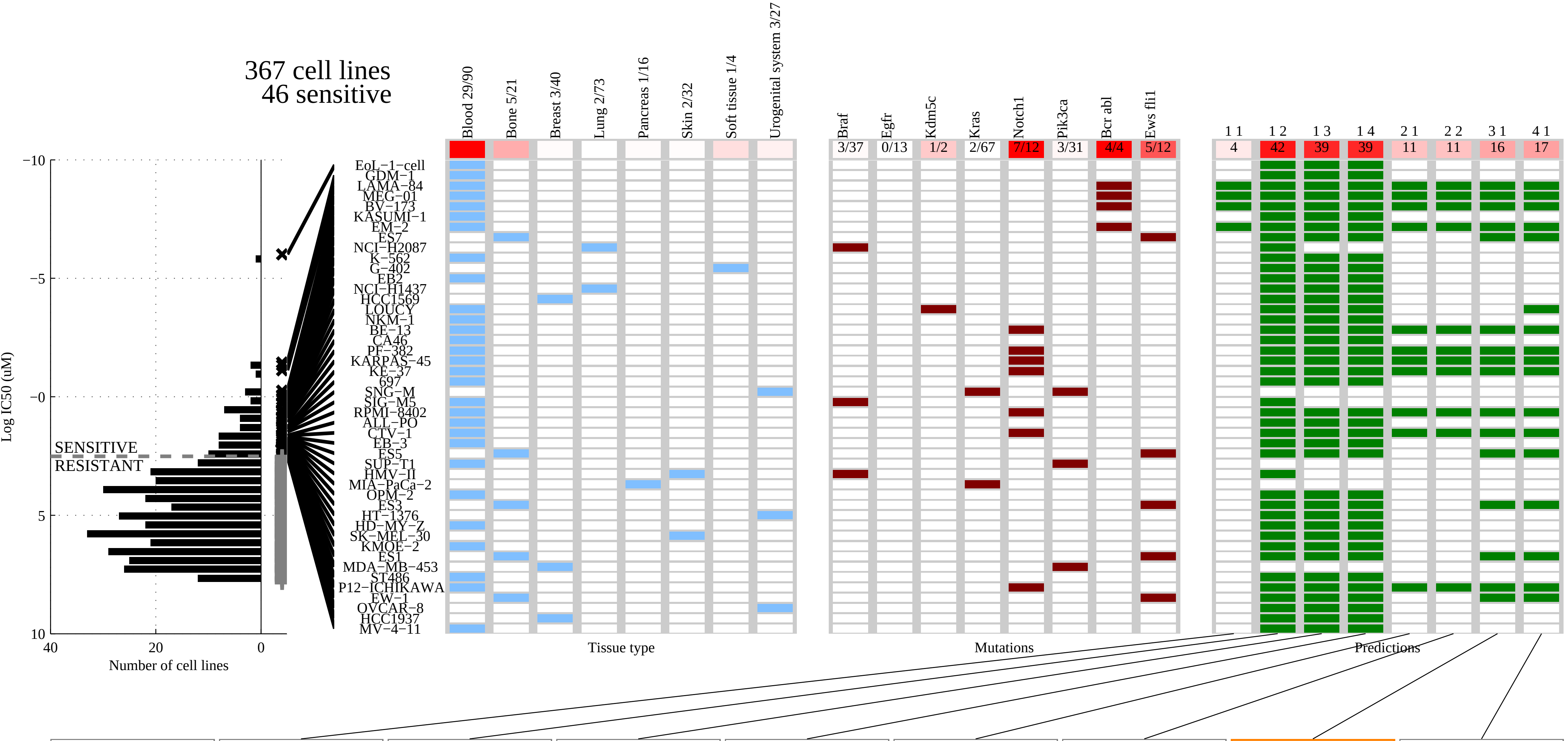

Model name	1 1	1 2	1 3	1 4	2 1	2 2	3 1	4 1
K / M	1 / 1	1 / 2	1 / 3	1 / 4	2 / 1	2 / 2	3 / 1	4 / 1
Logic formula	**BCR A**	**¬KRAS&¬PIK3C**	**¬BRAF&¬KRAS&¬PIK3C**	**¬BRAF&¬EGFR&¬KRAS&¬PIK3C**	**NOTCH | BCR A**	**[¬KRAS&NOTCH] | [BCR A&]**	**NOTCH | BCR A | EWS F**	**KDM5C | NOTCH | BCR A | EWS F**
TP	4	42	39	39	11	11	16	17
FP	0	234	203	196	5	2	12	13
FN	42	4	7	7	35	35	30	29
TN	321	87	118	125	316	319	309	308
Specificity	1	0.27	0.37	0.39	0.98	0.99	0.96	0.96
Precision	1	0.15	0.16	0.17	0.69	0.85	0.57	0.57
Recall	0.087	0.91	0.85	0.85	0.24	0.24	0.35	0.37

ID:219 AT−7519 −> CDK9

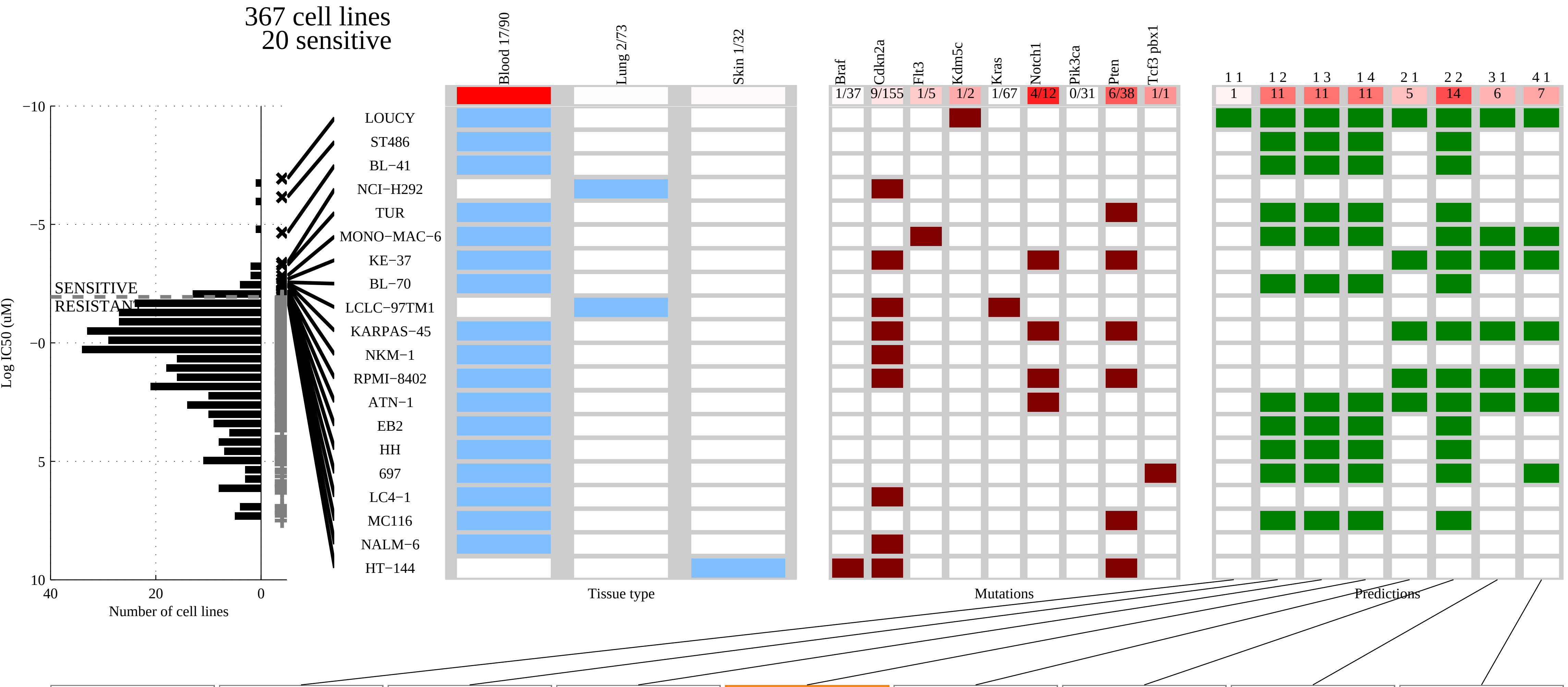

Model name<br>K M	1 1<br>1 1	1 2<br>1 2	1 3<br>1 3	1 4<br>1 4	2 1<br>2 1	2 2<br>2 2	3 1<br>3 1	4 1<br>4 1
Logic formula	**KDM5C**	**¬CDKN2&¬KRAS**	**¬CDKN2&¬KRAS& ¬PIK3C**	**¬BRAF&¬CDKN2& ¬KRAS&¬PIK3C**	**KDM5C|NOTCH**	**[NOTCH& PTEN] | [¬CDKN2&¬KRAS]**	**FLT3 |KDM5C| NOTCH**	**FLT3 |KDM5C| NOTCH| TCF3**
TP FP / FN TN	1 1 / 19 346	11 165 / 9 182	11 148 / 9 199	11 133 / 9 214	5 9 / 15 338	14 168 / 6 179	6 11 / 14 336	7 11 / 13 336
Specificity	1	0.52	0.57	0.62	0.97	0.52	0.97	0.97
Precision	0.5	0.063	0.069	0.076	0.36	0.077	0.35	0.39
Recall	0.05	0.55	0.55	0.55	0.25	0.7	0.3	0.35

ID:221 TAK−715 −> p38a

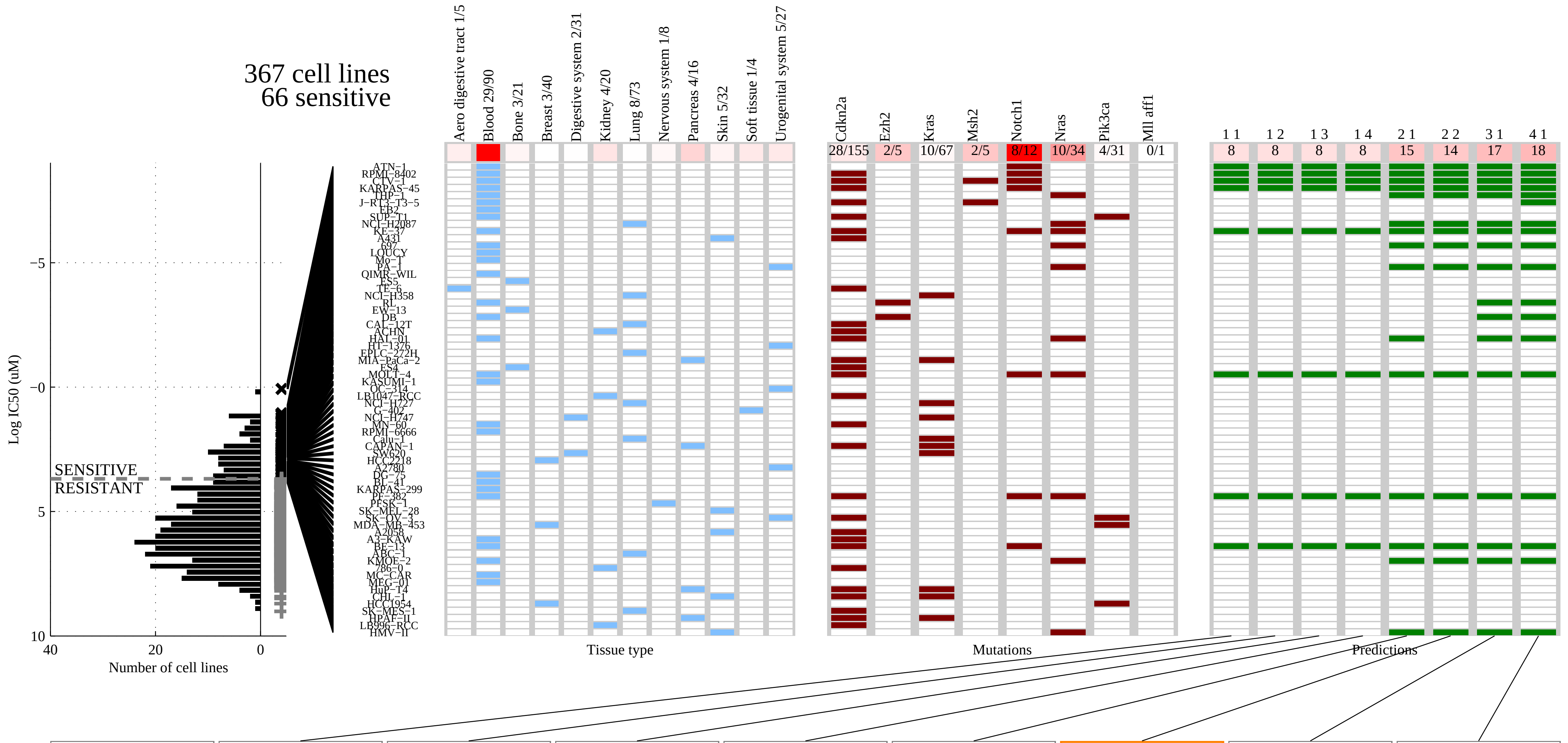

Model name (K, M)	1 1 (1, 1)	1 2 (1, 2)	1 3 (1, 3)	1 4 (1, 4)	2 1 (2, 1)	2 2 (2, 2)	3 1 (3, 1)	4 1 (4, 1)
Logic formula	NOTCH	¬KRAS&NOTCH	¬KRAS&NOTCH& ¬MLL A	¬KRAS&NOTCH& ¬PIK3C&	NOTCH | NRAS	[¬CDKN2& NRAS] | [¬KRAS&NOTCH]	EZH2 | NOTCH | NRAS	EZH2 | MSH2 | NOTCH | NRAS
TP	8	8	8	8	15	14	17	18
FP	4	1	1	1	27	9	29	31
FN	58	58	58	58	51	52	49	48
TN	297	300	300	300	274	292	272	270
Specificity	0.99	1	1	1	0.91	0.97	0.9	0.9
Precision	0.67	0.89	0.89	0.89	0.36	0.61	0.37	0.37
Recall	0.12	0.12	0.12	0.12	0.23	0.21	0.26	0.27

ID:222 BX−912 −> PDPK1 (PDK1)

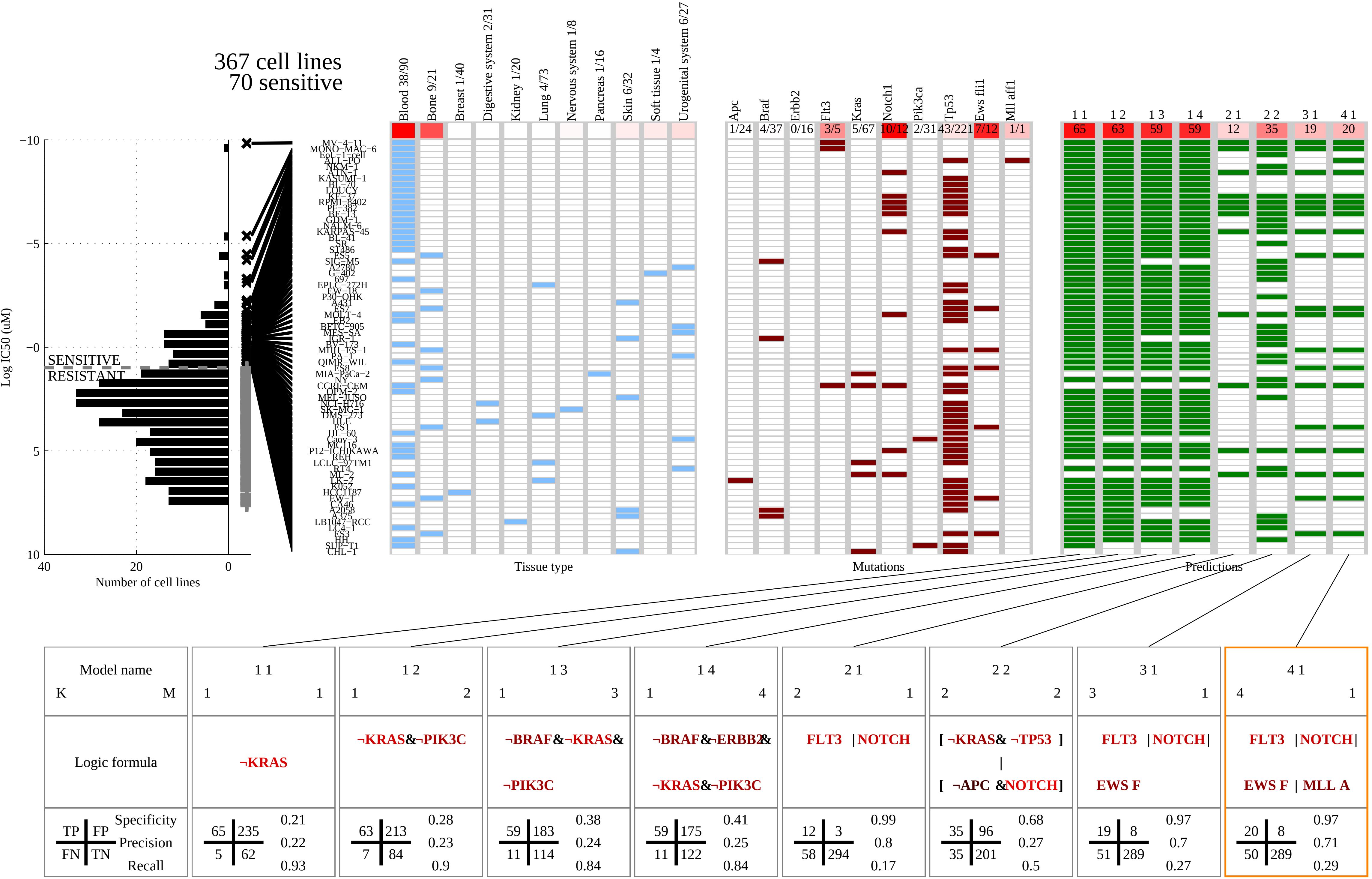

ID:223 ZSTK474 −> PI3K

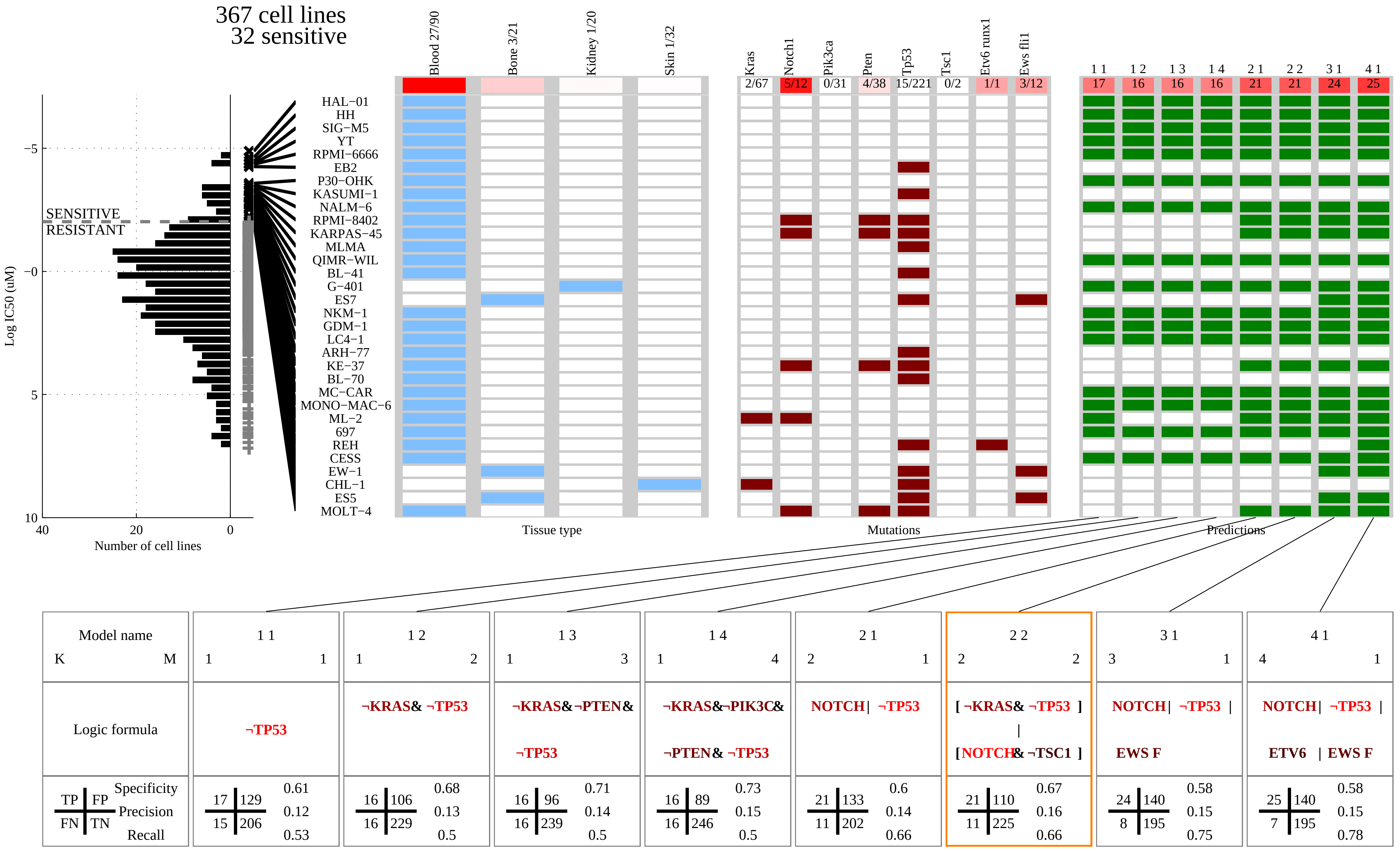

ID:224 AS605240 −> PI3Kgamma

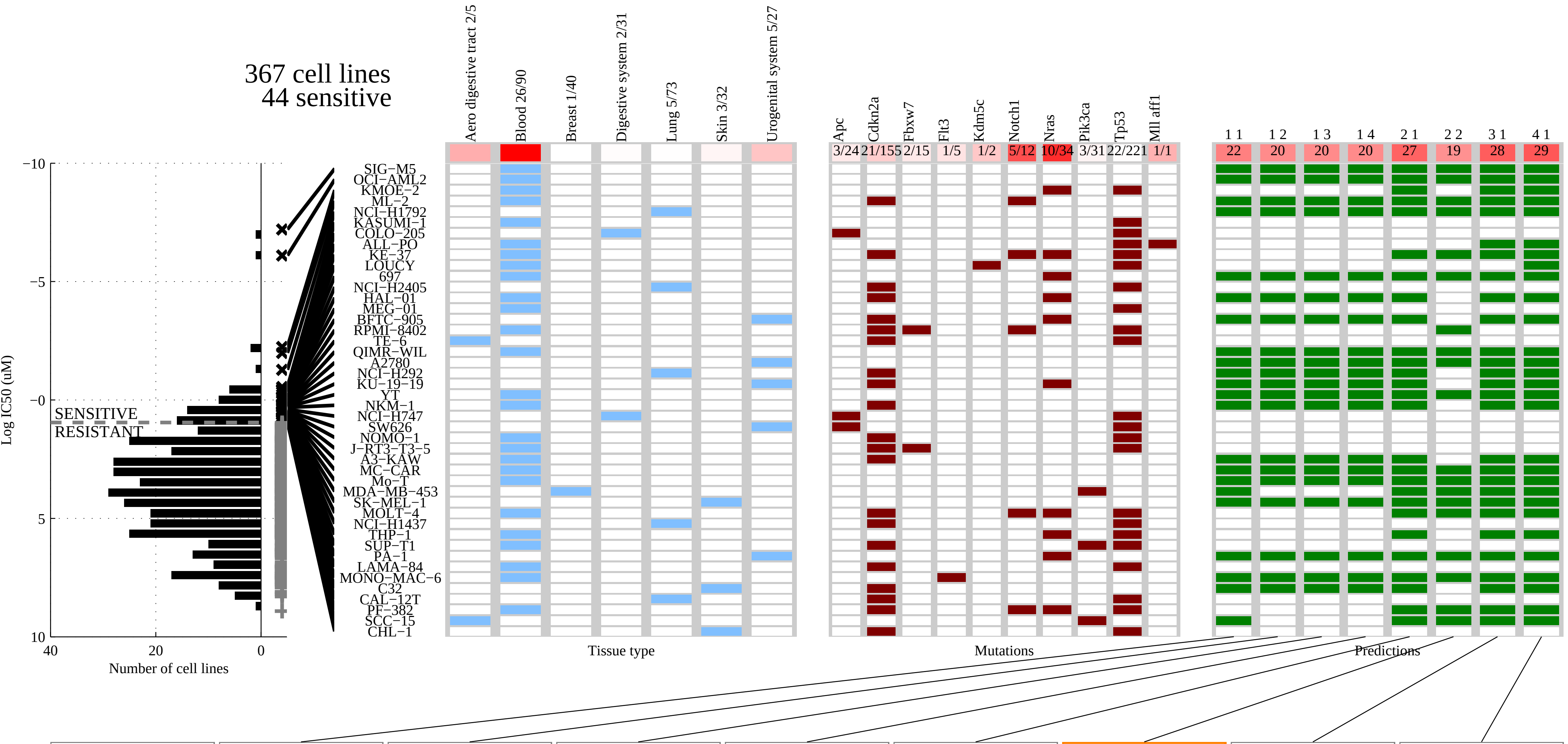

Model name	1 1	1 2	1 3	1 4	2 1	2 2	3 1	4 1
K	1	1	1	1	2	2	3	4
M	1	2	3	4	1	2	1	1
Logic formula	¬TP53	¬PIK3C& ¬TP53	¬APC &¬PIK3C& ¬TP53	¬APC &¬FBXW7& ¬PIK3C& ¬TP53	NRAS | ¬TP53	[¬CDKN2& ¬TP53] | [¬FLT3 &NOTCH]	NRAS | ¬TP53 | MLL A	KDM5C | NRAS | ¬TP53 | MLL A
TP	22	20	20	20	27	19	28	29
FP	124	112	109	105	138	65	138	138
FN	22	24	24	24	17	25	16	15
TN	199	211	214	218	185	258	185	185
Specificity	0.62	0.65	0.66	0.67	0.57	0.8	0.57	0.57
Precision	0.15	0.15	0.16	0.16	0.16	0.23	0.17	0.17
Recall	0.5	0.45	0.45	0.45	0.61	0.43	0.64	0.66

ID:225 Genentech Cpd 10 −> AURKA, AURKB

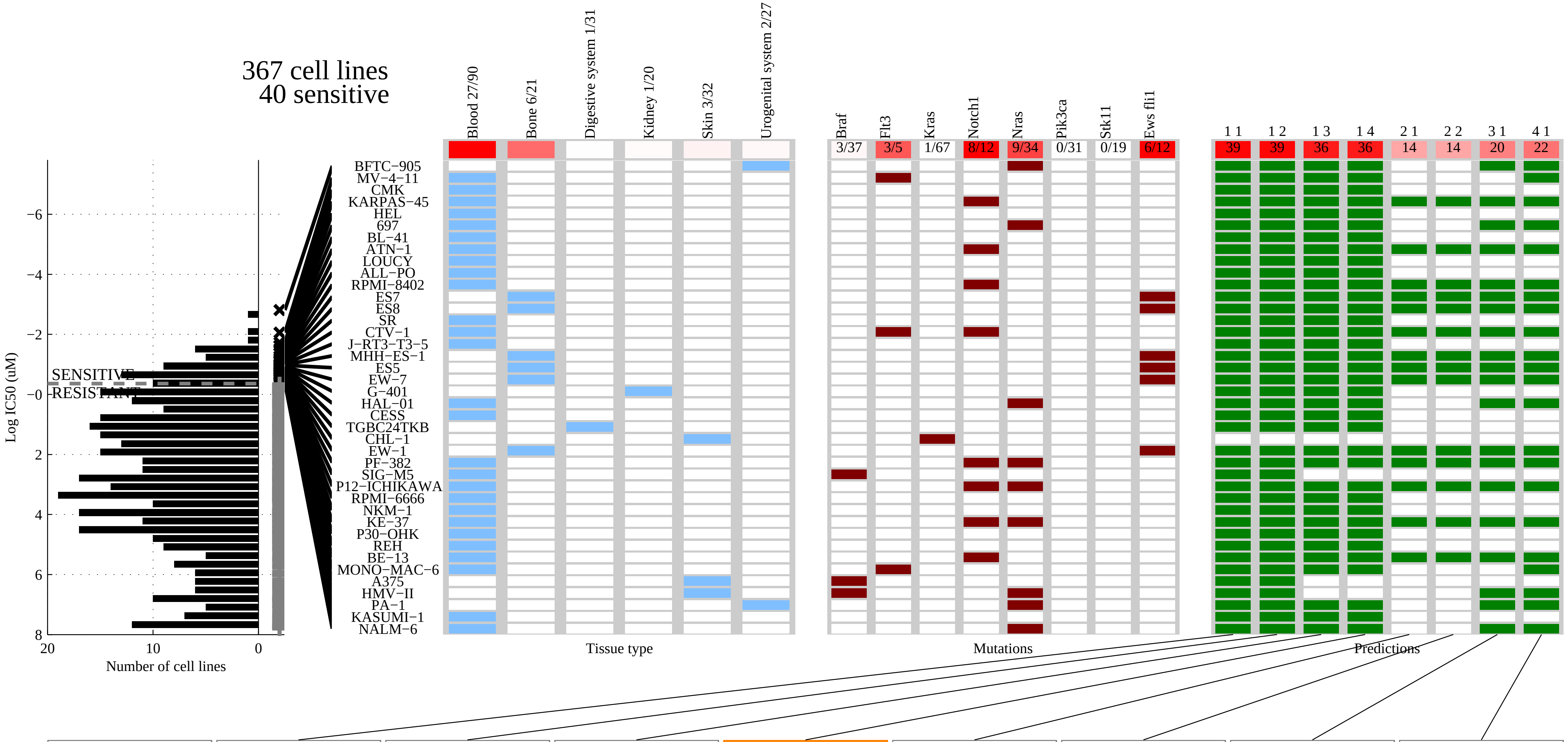

Model name (K, M)	1 1 (1, 1)	1 2 (1, 2)	1 3 (1, 3)	1 4 (1, 4)	2 1 (2, 1)	2 2 (2, 2)	3 1 (3, 1)	4 1 (4, 1)
Logic formula	¬KRAS	¬KRAS&¬PIK3C	¬BRAF&¬KRAS& ¬PIK3C	¬BRAF&¬KRAS& ¬PIK3C&¬STK11	NOTCH | EWS F	[¬BRAF& EWS F] | [¬KRAS&NOTCH]	NOTCH | NRAS | EWS F	FLT3 | NOTCH | NRAS | EWS F
TP / FP	39 / 261	39 / 237	36 / 206	36 / 198	14 / 10	14 / 6	20 / 34	22 / 34
FN / TN	1 / 66	1 / 90	4 / 121	4 / 129	26 / 317	26 / 321	20 / 293	18 / 293
Specificity	0.2	0.28	0.37	0.39	0.97	0.98	0.9	0.9
Precision	0.13	0.14	0.15	0.15	0.58	0.7	0.37	0.39
Recall	0.97	0.97	0.9	0.9	0.35	0.35	0.5	0.55

ID:226 GSK1070916 −> AURKB

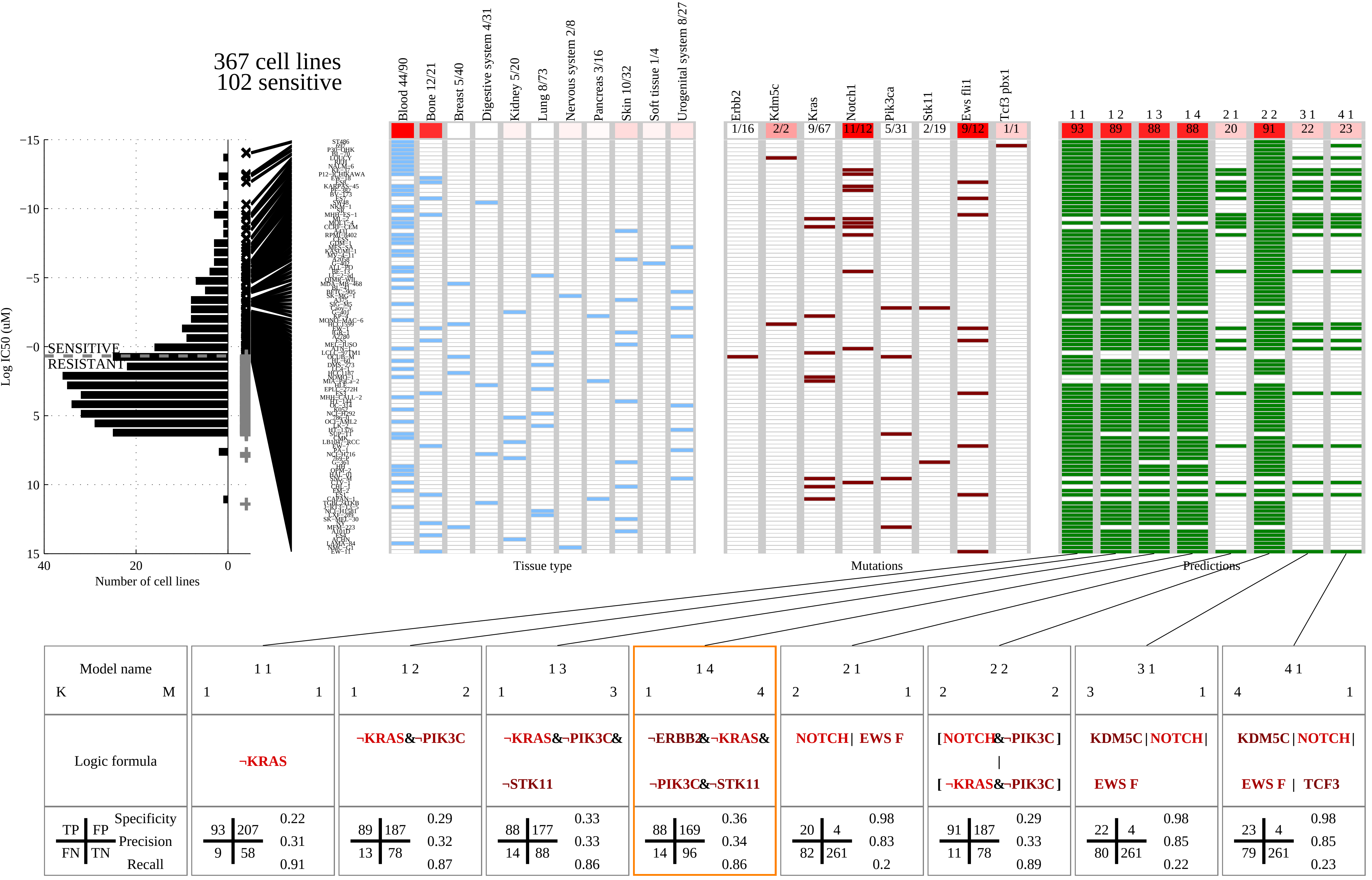

ID:227 THZ−2−98−1 -> IRAK1

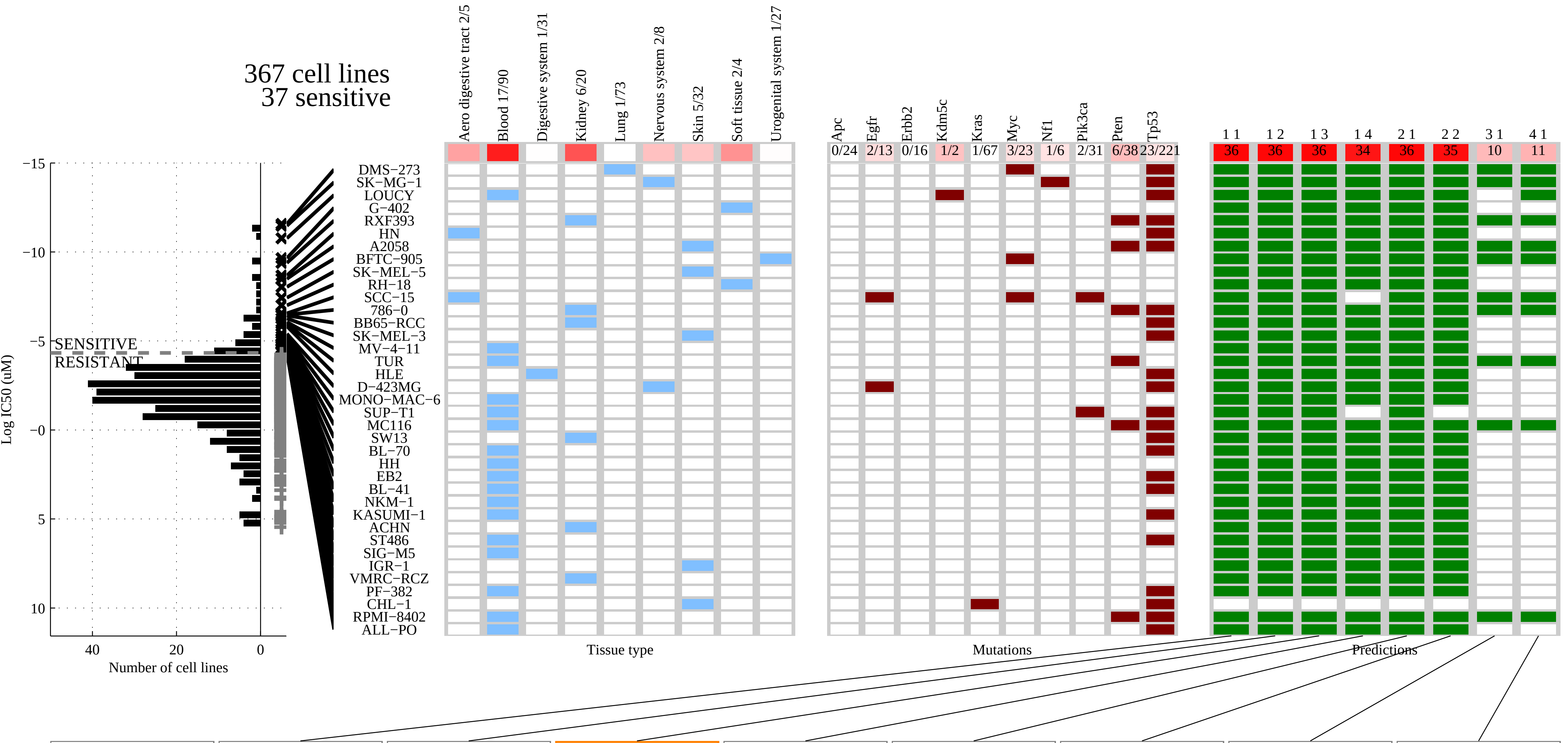

Model name (K, M)	1 1	1 2	1 3	1 4	2 1	2 2	3 1	4 1
K / M	1 / 1	1 / 2	1 / 3	1 / 4	2 / 1	2 / 2	3 / 1	4 / 1
Logic formula	¬KRAS	¬ERBB2&¬KRAS	¬APC &¬ERBB2& ¬KRAS	¬APC &¬ERBB2& ¬KRAS&¬PIK3C	¬KRAS |	[¬KRAS&¬PIK3C] | [EGFR & ¬TP53]	MYC | NF1 | PTEN	KDM5C | MYC | NF1 | PTEN
TP FP	36 264	36 249	36 239	34 224	36 264	35 242	10 56	11 57
FN TN	1 66	1 81	1 91	3 106	1 66	2 88	27 274	26 273
Specificity	0.2	0.25	0.28	0.32	0.2	0.27	0.83	0.83
Precision	0.12	0.13	0.13	0.13	0.12	0.13	0.15	0.16
Recall	0.97	0.97	0.97	0.92	0.97	0.95	0.27	0.3

ID:228 KIN001−102 −> AKT1

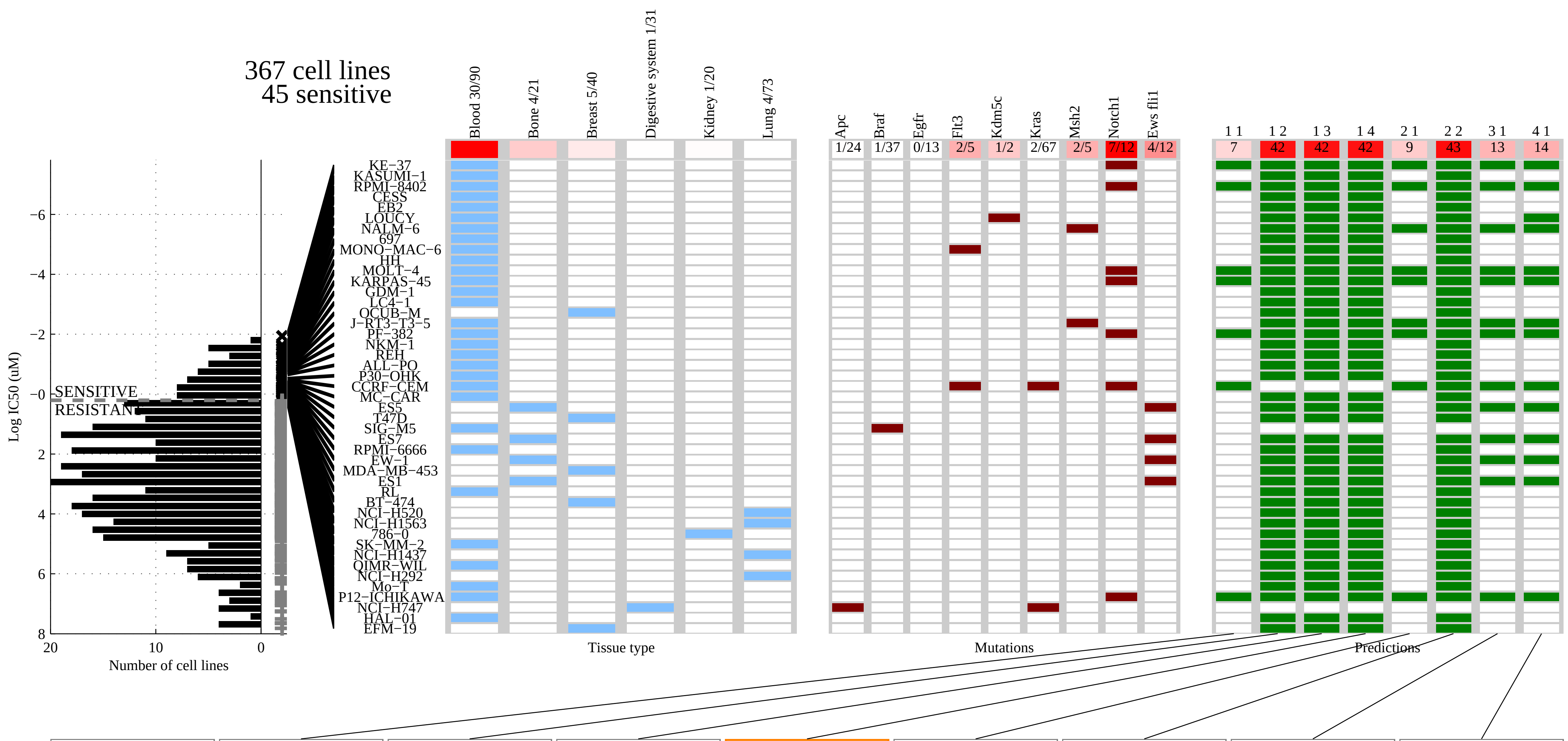

Model name<br>K M	1 1<br>1 1	1 2<br>1 2	1 3<br>1 3	1 4<br>1 4	2 1<br>2 1	2 2<br>2 2	3 1<br>3 1	4 1<br>4 1
Logic formula	NOTCH	¬BRAF&¬KRAS	¬BRAF&¬EGFR& ¬KRAS	¬APC &¬BRAF& ¬EGFR&¬KRAS	MSH2 |NOTCH	[¬BRAF&¬KRAS] | [¬BRAF& FLT3]	MSH2 |NOTCH| EWS F	KDM5C| MSH2 | NOTCH| EWS F
TP FP / FN TN	7 5 / 38 317	42 223 / 3 99	42 214 / 3 108	42 206 / 3 116	9 7 / 36 315	43 223 / 2 99	13 15 / 32 307	14 16 / 31 306
Specificity	0.98	0.31	0.34	0.36	0.98	0.31	0.95	0.95
Precision	0.58	0.16	0.16	0.17	0.56	0.16	0.46	0.47
Recall	0.16	0.93	0.93	0.93	0.2	0.96	0.29	0.31

ID:229 LY317615 –> PRKCB (PKCbeta)

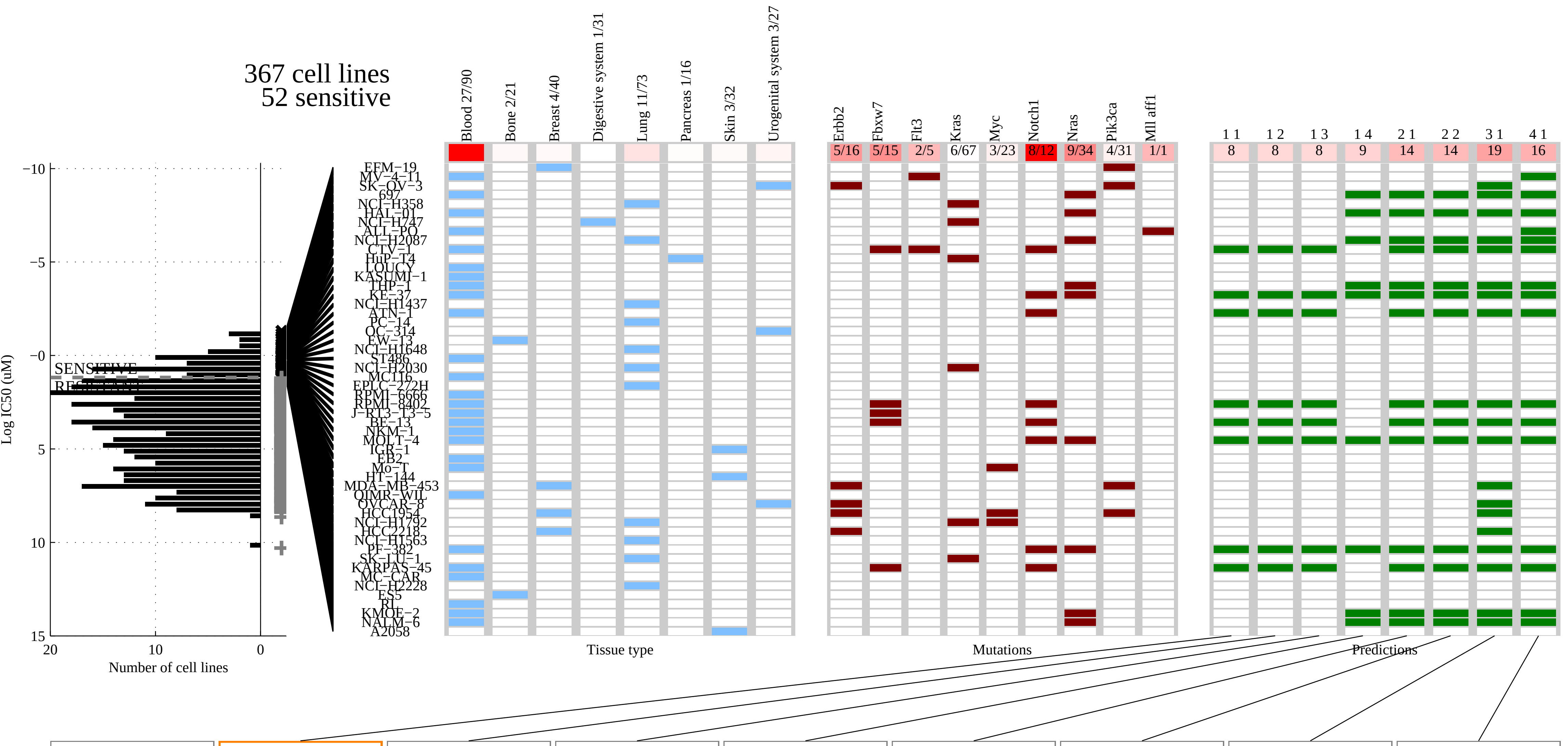

Model name (K, M)	1 1 (1, 1)	1 2 (1, 2)	1 3 (1, 3)	1 4 (1, 4)	2 1 (2, 1)	2 2 (2, 2)	3 1 (3, 1)	4 1 (4, 1)
Logic formula	NOTCH	¬KRAS&NOTCH	¬KRAS&NOTCH&	¬FBXW7& ¬MYC & NRAS &¬PIK3C	NOTCH | NRAS	[¬MYC & NRAS] | [¬KRAS&NOTCH]	ERBB2 | NOTCH | NRAS	FLT3 | NOTCH | NRAS | MLL A
TP	8	8	8	9	14	14	19	16
FP	4	1	1	17	28	21	38	29
FN	44	44	44	43	38	38	33	36
TN	311	314	314	298	287	294	277	286
Specificity	0.99	1	1	0.95	0.91	0.93	0.88	0.91
Precision	0.67	0.89	0.89	0.35	0.33	0.4	0.33	0.36
Recall	0.15	0.15	0.15	0.17	0.27	0.27	0.37	0.31

ID:230 GSK429286A –> ROCK2

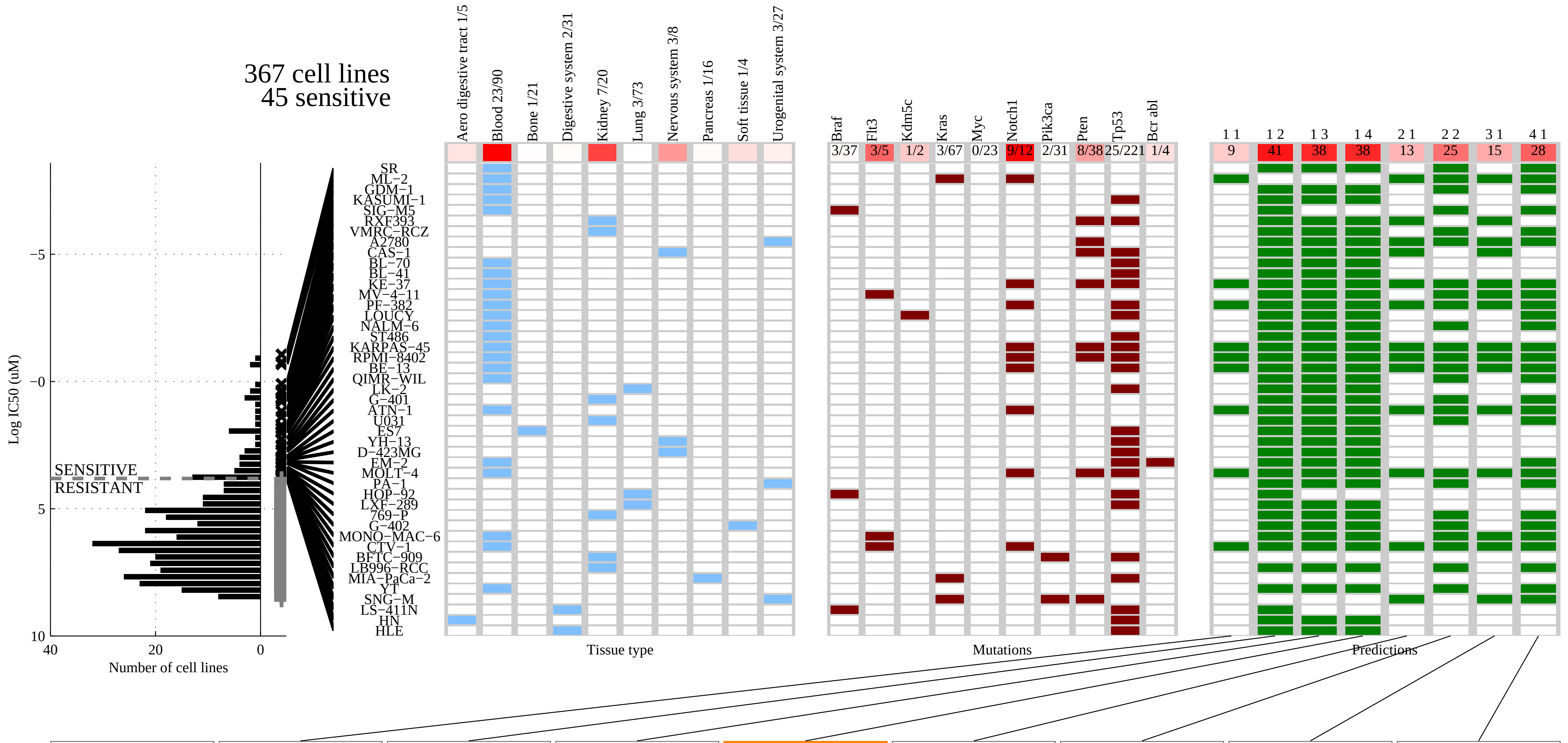

Model name (K / M)	1 1 (1 / 1)	1 2 (1 / 2)	1 3 (1 / 3)	1 4 (1 / 4)	2 1 (2 / 1)	2 2 (2 / 2)	3 1 (3 / 1)	4 1 (4 / 1)
Logic formula	NOTCH	¬KRAS&¬PIK3C	¬BRAF&¬KRAS& ¬PIK3C	¬BRAF&¬KRAS& ¬MYC &¬PIK3C	NOTCH| PTEN	[NOTCH&¬PIK3C] | [¬KRAS& ¬TP53]	FLT3 |NOTCH| PTEN	KDM5C|NOTCH| ¬TP53 | BCR A
TP FP / FN TN	9 3 / 36 319	41 235 / 4 87	38 204 / 7 118	38 192 / 7 130	13 31 / 32 291	25 106 / 20 216	15 32 / 30 290	28 130 / 17 192
Specificity	0.99	0.27	0.37	0.4	0.9	0.67	0.9	0.6
Precision	0.75	0.15	0.16	0.17	0.3	0.19	0.32	0.18
Recall	0.2	0.91	0.84	0.84	0.29	0.56	0.33	0.62

ID:231 FMK -> RSK

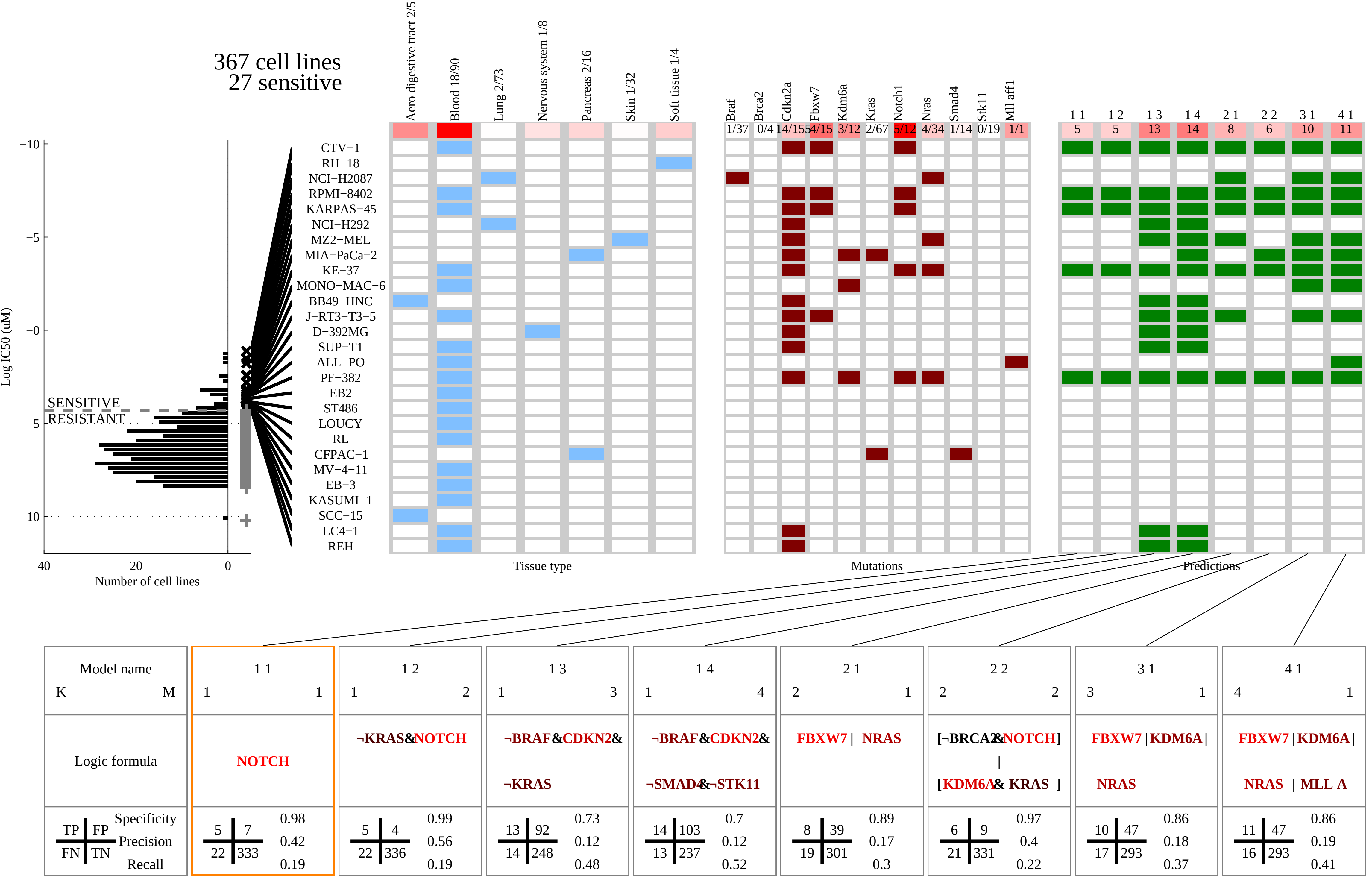

ID:235 QL−XII−47 −> BTK, BMX

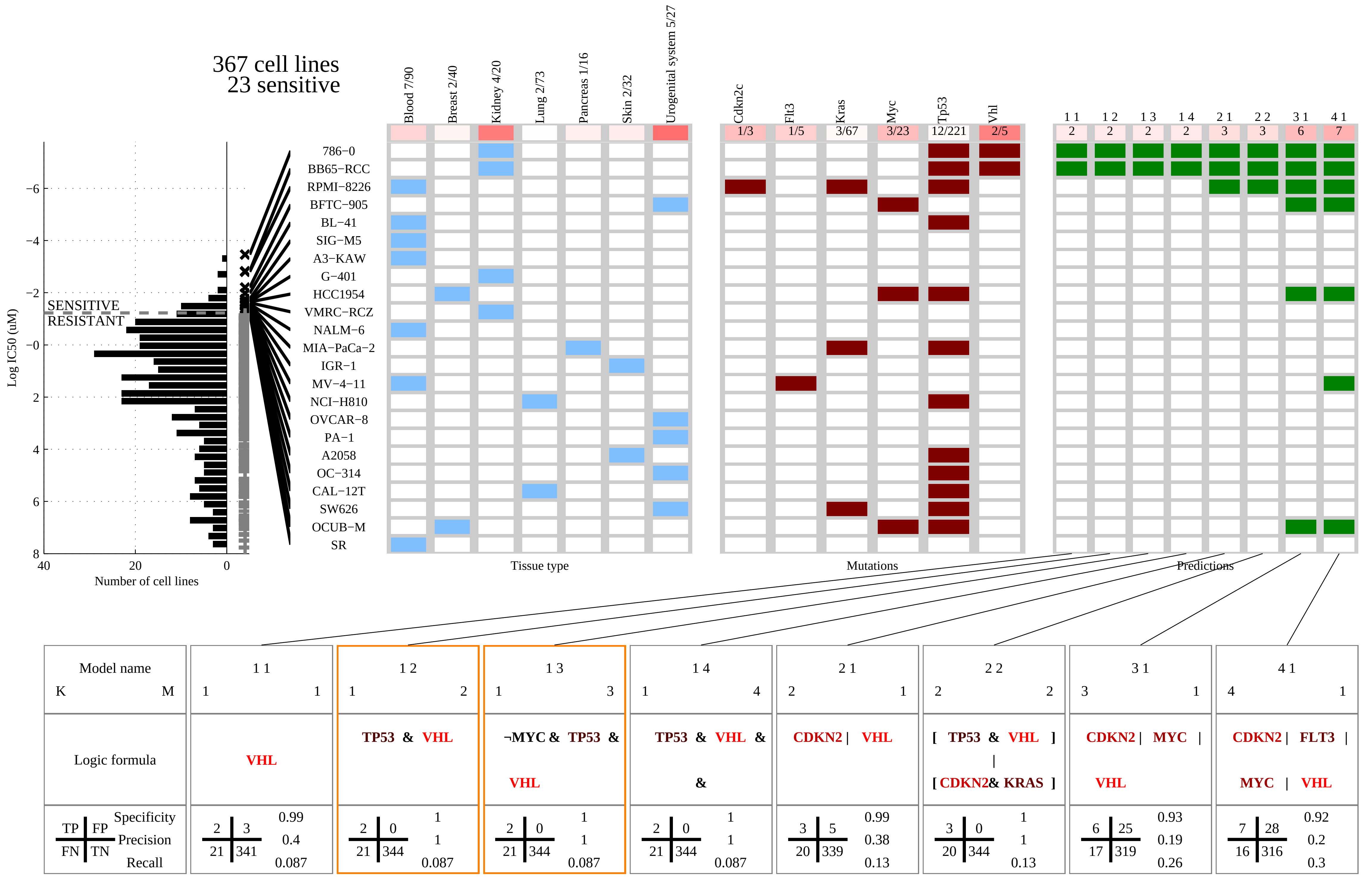

ID:238 CAL−101 −> PI3Kdelta

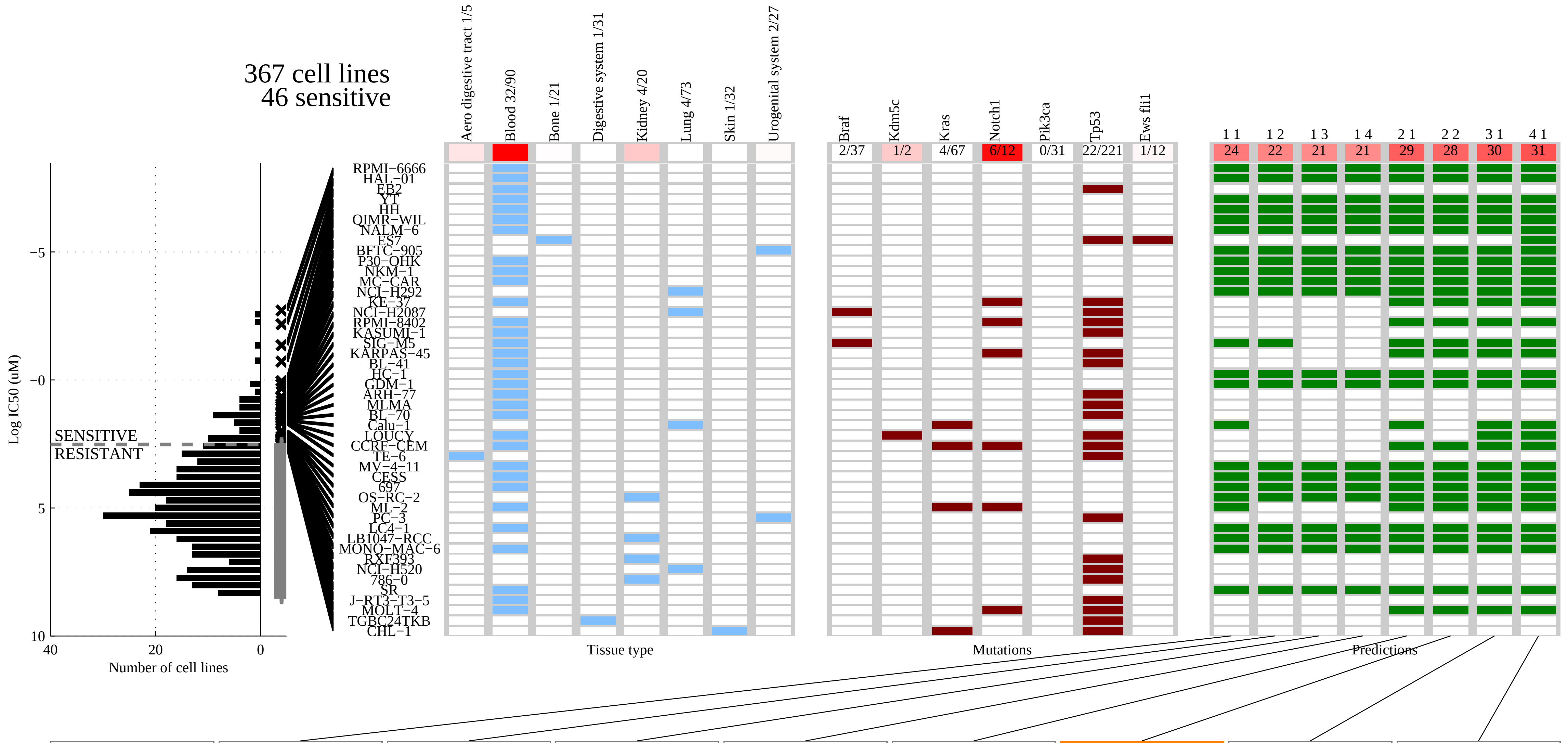

Model name (K M)	1 1	1 2	1 3	1 4	2 1	2 2	3 1	4 1
K / M	1 / 1	1 / 2	1 / 3	1 / 4	2 / 1	2 / 2	3 / 1	4 / 1
Logic formula	¬TP53	¬KRAS& ¬TP53	¬BRAF&¬KRAS& ¬TP53	¬BRAF&¬KRAS& ¬PIK3C& ¬TP53	NOTCH | ¬TP53	[¬KRAS& ¬TP53] | [NOTCH&¬PIK3C]	KDM5C | NOTCH | ¬TP53	KDM5C | NOTCH | ¬TP53 | EWS F
TP FP / FN TN	24 122 / 22 199	22 100 / 24 221	21 81 / 25 240	21 75 / 25 246	29 125 / 17 196	28 103 / 18 218	30 125 / 16 196	31 134 / 15 187
Specificity	0.62	0.69	0.75	0.77	0.61	0.68	0.61	0.58
Precision	0.16	0.18	0.21	0.22	0.19	0.21	0.19	0.19
Recall	0.52	0.48	0.46	0.46	0.63	0.61	0.65	0.67

ID:3 Rapamycin –> MTOR

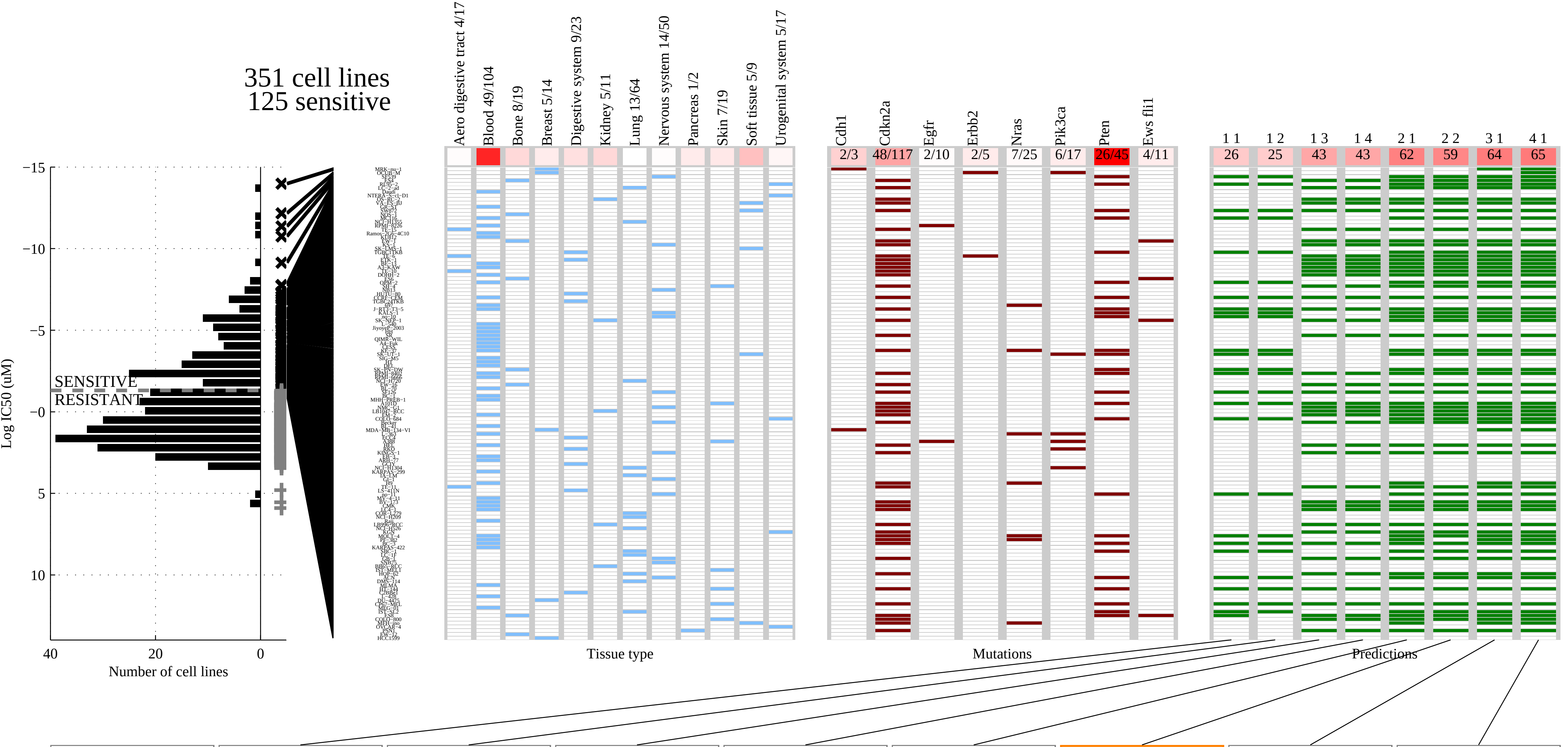

Model name	1 1	1 2	1 3	1 4	2 1	2 2	3 1	4 1
K	1	1	1	1	2	2	3	4
M	1	2	3	4	1	2	1	1
Logic formula	PTEN	PTEN &¬EWS F	CDKN2&¬EGFR& ¬NRAS	CDKN2&¬EGFR& ¬NRAS&¬PIK3C	CDKN2 | PTEN	[PTEN &¬EWS F] | [CDKN2&¬NRAS]	CDH1 | CDKN2 | PTEN	CDH1 | CDKN2 | ERBB2 | PTEN
TP	26	25	43	43	62	59	64	65
FP	19	18	53	49	81	70	82	84
FN	99	100	82	82	63	66	61	60
TN	207	208	173	177	145	156	144	142
Specificity	0.92	0.92	0.77	0.78	0.64	0.69	0.64	0.63
Precision	0.58	0.58	0.45	0.47	0.43	0.46	0.44	0.44
Recall	0.21	0.2	0.34	0.34	0.5	0.47	0.51	0.52

ID:30 Sorafenib −> PDGFRA, PDGFRB, KDR, KIT, FLT3

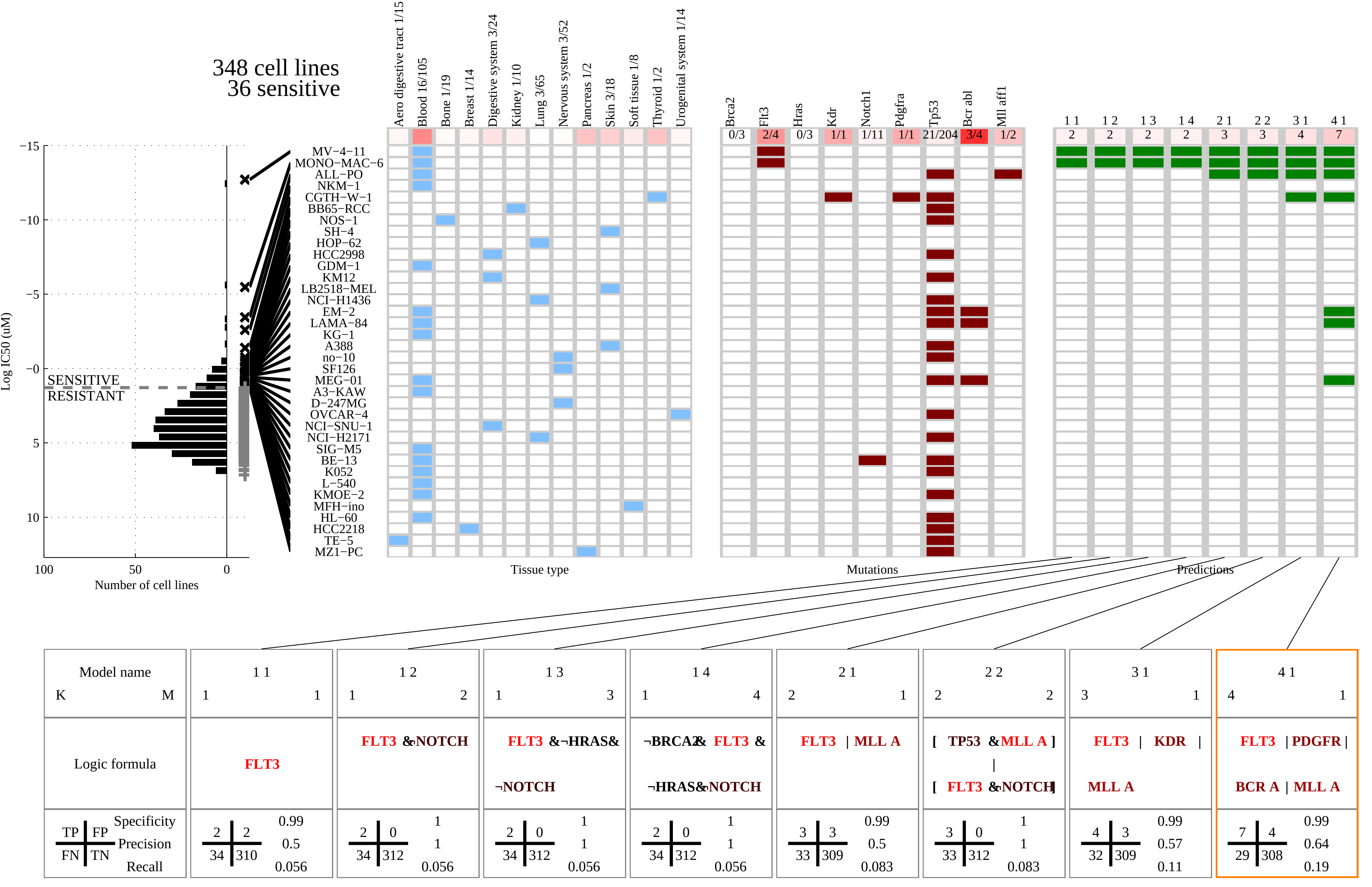

ID:40 TP−155 −> NFkappaB

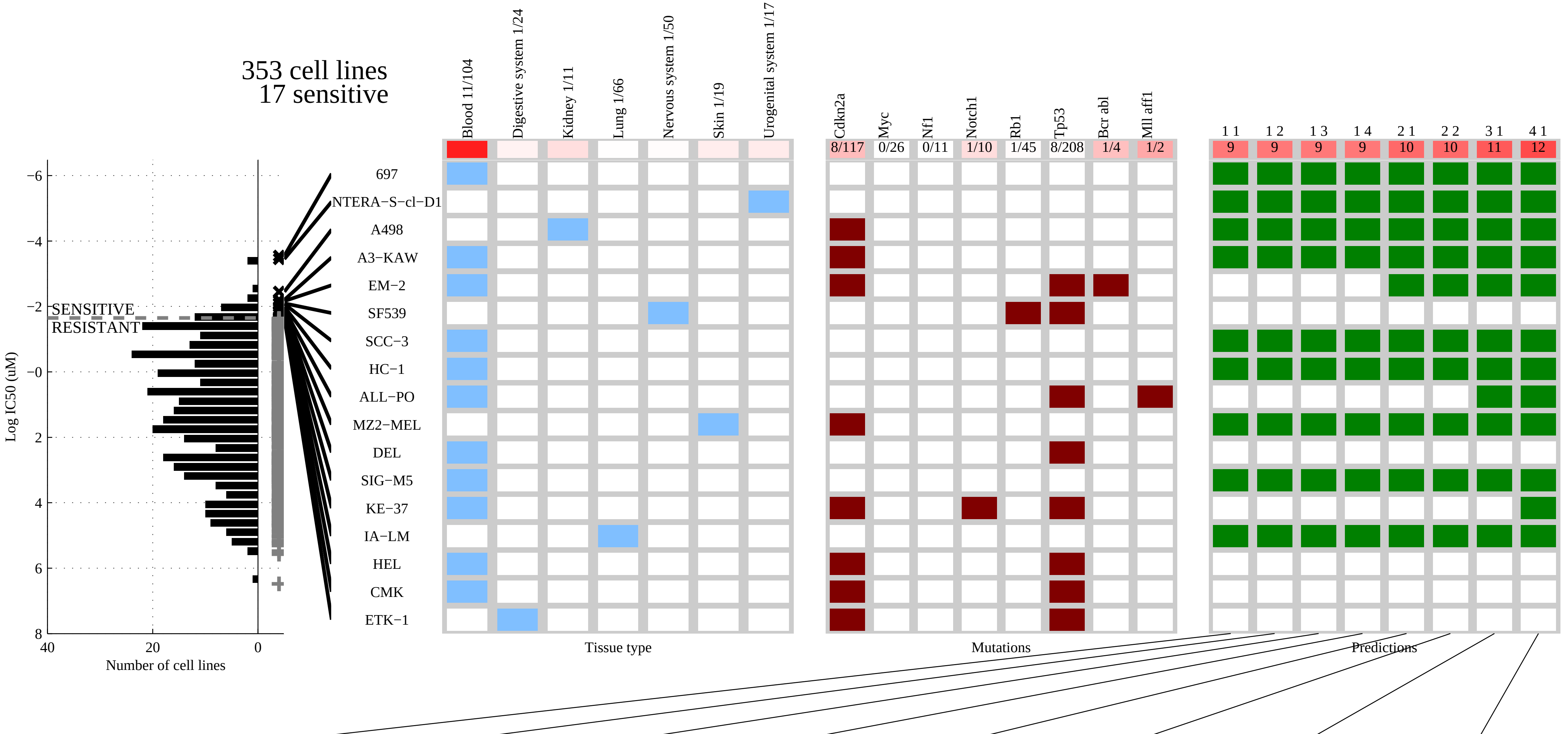

<table>
<tr><td>Model name
K M</td><td>1 1
1 1</td><td>1 2
1 2</td><td>1 3
1 3</td><td>1 4
1 4</td><td>2 1
2 1</td><td>2 2
2 2</td><td>3 1
3 1</td><td>4 1
4 1</td></tr>
<tr><td>Logic formula</td><td>¬TP53</td><td>¬RB1 & ¬TP53</td><td>¬NF1 & ¬RB1 & ¬TP53</td><td>¬MYC & ¬NF1 & ¬RB1 & ¬TP53</td><td>¬TP53 | BCR A</td><td>[CDKN2& BCR A] | [¬RB1 & ¬TP53]</td><td>¬TP53 | BCR A | MLL A</td><td>NOTCH | ¬TP53 | BCR A | MLL A</td></tr>
<tr><td>TP FP / FN TN
Specificity
Precision
Recall</td><td>9 136 / 8 200
0.6
0.062
0.53</td><td>9 125 / 8 211
0.63
0.067
0.53</td><td>9 118 / 8 218
0.65
0.071
0.53</td><td>9 114 / 8 222
0.66
0.073
0.53</td><td>10 138 / 7 198
0.59
0.068
0.59</td><td>10 126 / 7 210
0.63
0.074
0.59</td><td>11 138 / 6 198
0.59
0.074
0.65</td><td>12 144 / 5 192
0.57
0.077
0.71</td></tr>
</table>

ID:41 S−Trityl−L−cysteine −> KIF11

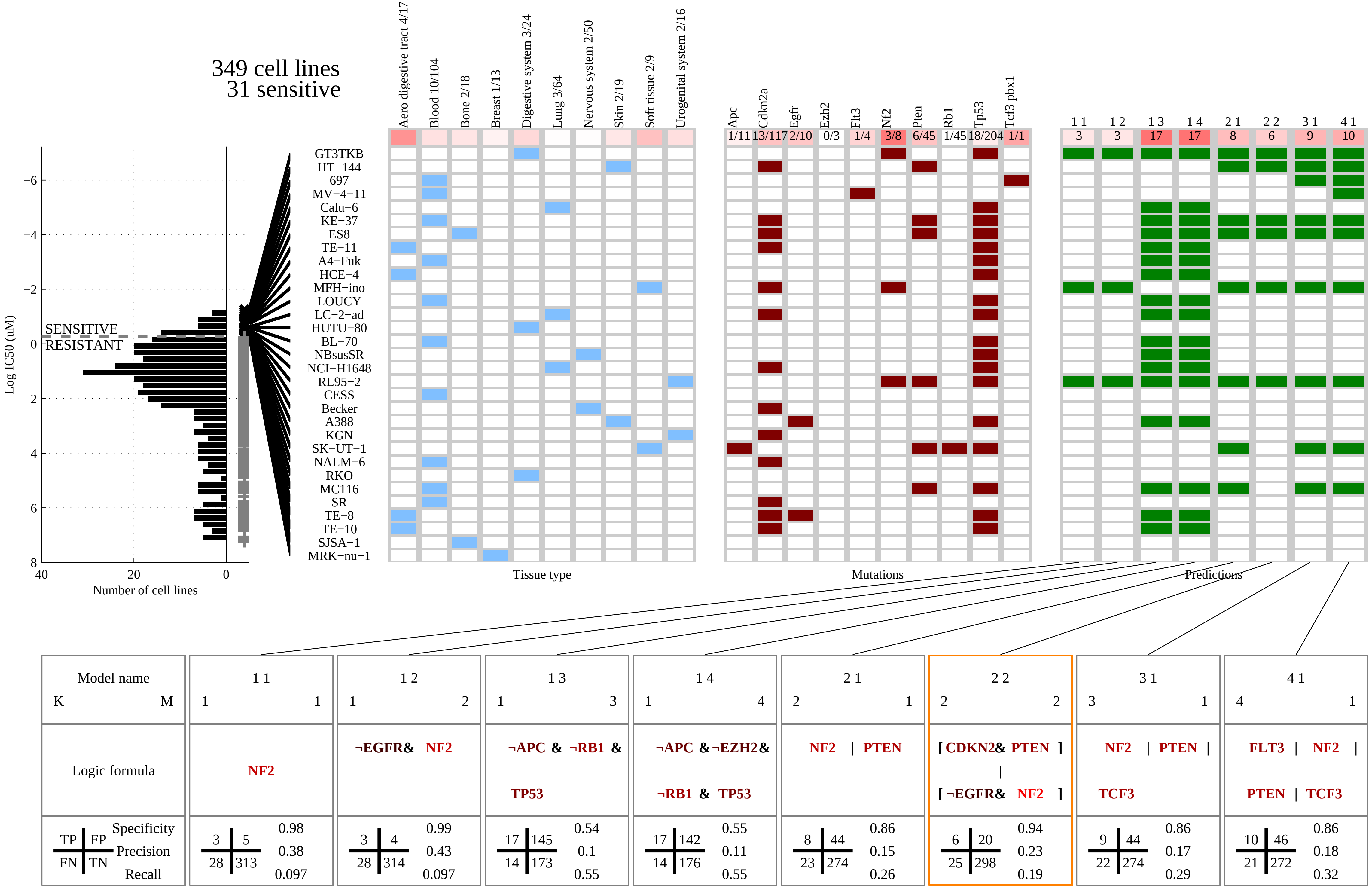

ID:45 Z−LLNle−CHO −> g−secretase

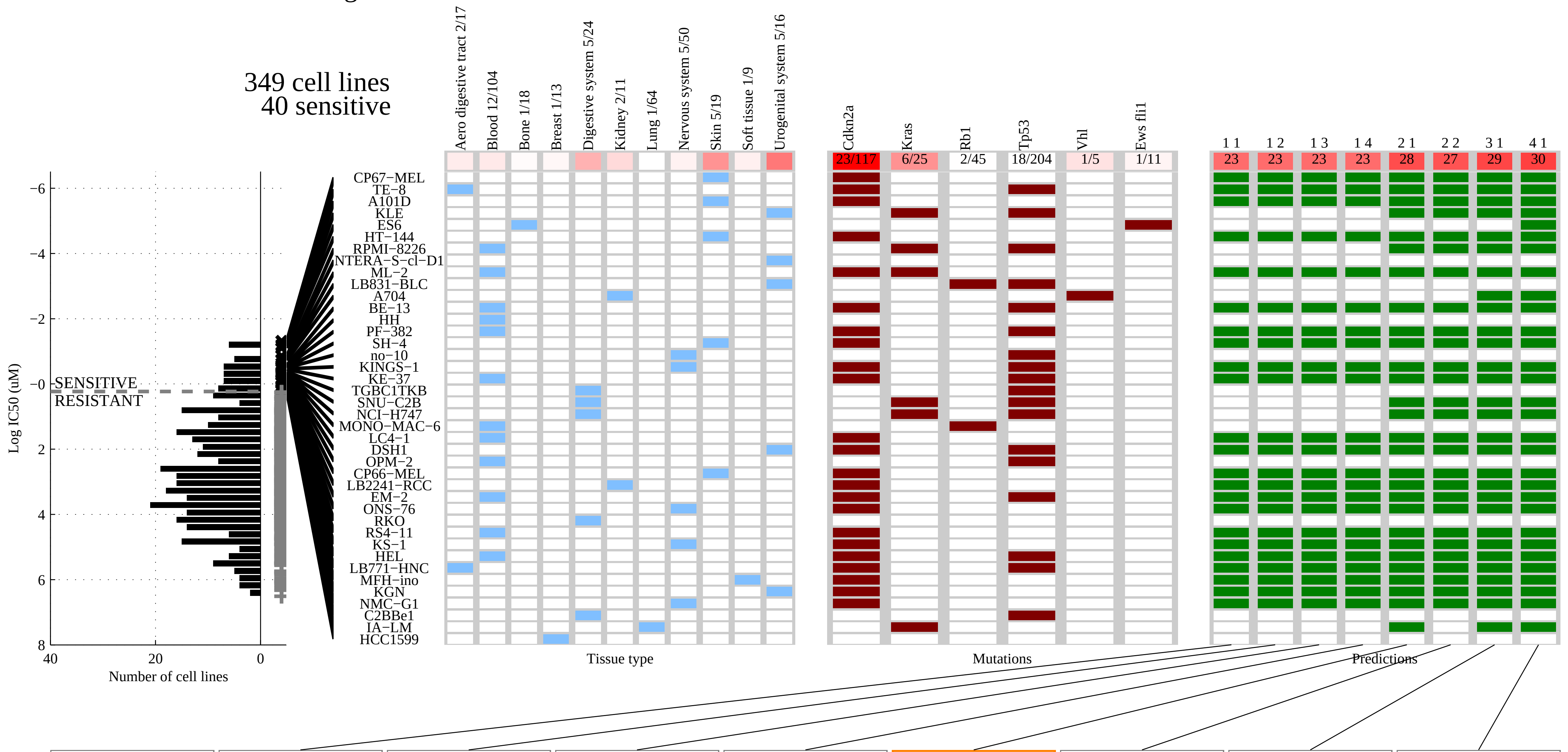

Model name	1 1	1 2	1 3	1 4	2 1	2 2	3 1	4 1
K	1	1	1	1	2	2	3	4
M	1	2	3	4	1	2	1	1
Logic formula	CDKN2	CDKN2& ¬RB1	CDKN2& ¬RB1 & ¬EWS F	CDKN2& ¬RB1 & ¬VHL &¬EWS F	CDKN2 | KRAS	[CDKN2& ¬RB1] | [KRAS & TP53]	CDKN2 | KRAS | VHL	CDKN2 | KRAS | VHL | EWS F
TP	23	23	23	23	28	27	29	30
FP	94	87	83	80	106	97	107	113
FN	17	17	17	17	12	13	11	10
TN	215	222	226	229	203	212	202	196
Specificity	0.7	0.72	0.73	0.74	0.66	0.69	0.65	0.63
Precision	0.2	0.21	0.22	0.22	0.21	0.22	0.21	0.21
Recall	0.57	0.57	0.57	0.57	0.7	0.68	0.72	0.75

ID:5 Sunitinib −> PDGFRA, PDGFRB, KDR, KIT, FLT3

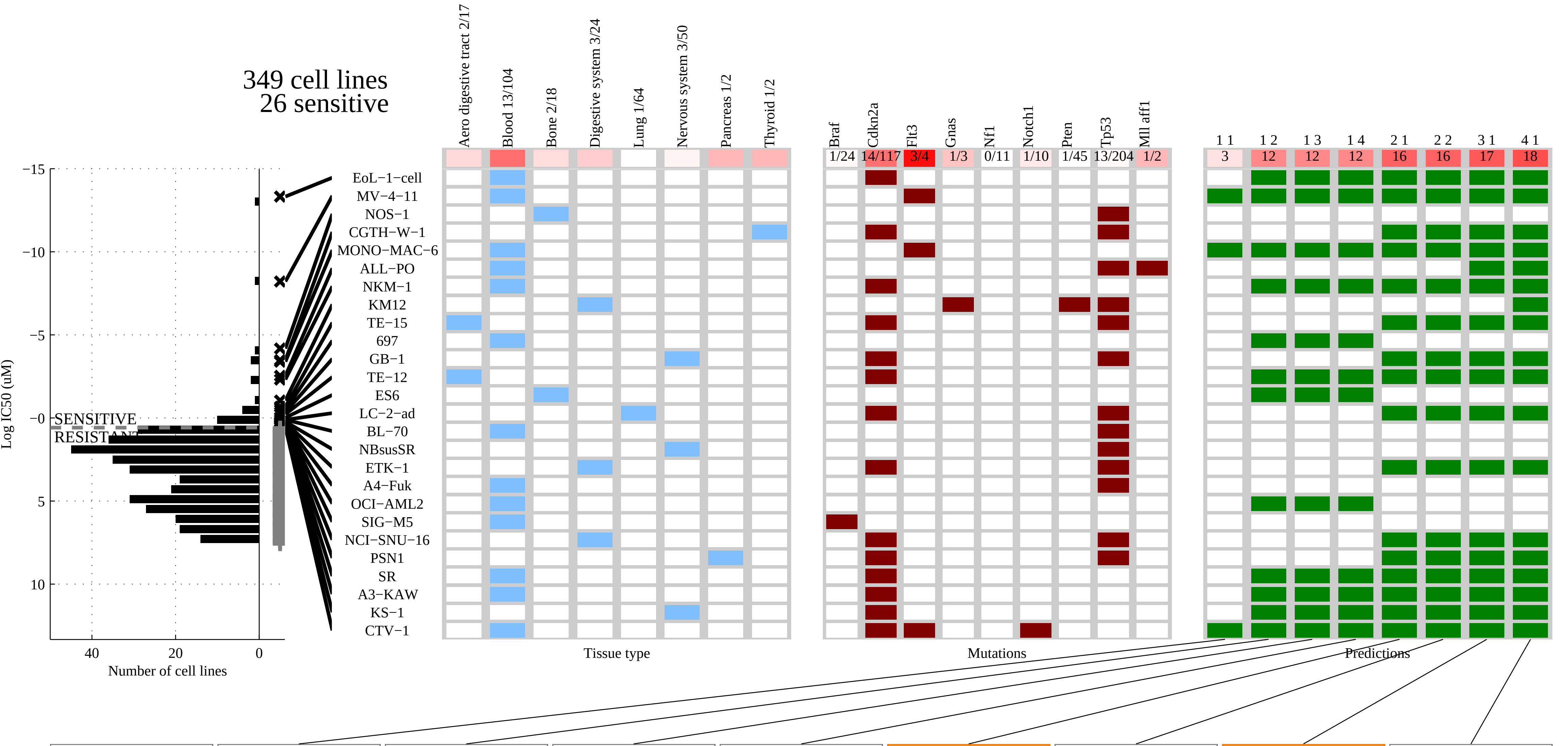

Model name	1 1	1 2	1 3	1 4	2 1	2 2	3 1	4 1
K / M	1 / 1	1 / 2	1 / 3	1 / 4	2 / 1	2 / 2	3 / 1	4 / 1
Logic formula	**FLT3**	**¬BRAF & ¬TP53**	**¬BRAF & ¬NF1 & ¬TP53**	**¬BRAF & ¬NF1 & ¬PTEN & ¬TP53**	**CDKN2 | FLT3**	**[FLT3 & NOTCH] | [CDKN2 & ¬PTEN]**	**CDKN2 | FLT3 | MLL A**	**CDKN2 | FLT3 | GNAS | MLL A**
TP / FP	3 / 1	12 / 114	12 / 108	12 / 102	16 / 103	16 / 84	17 / 103	18 / 105
FN / TN	23 / 322	14 / 209	14 / 215	14 / 221	10 / 220	10 / 239	9 / 220	8 / 218
Specificity	1	0.65	0.67	0.68	0.68	0.74	0.68	0.67
Precision	0.75	0.095	0.1	0.11	0.13	0.16	0.14	0.15
Recall	0.12	0.46	0.46	0.46	0.62	0.62	0.65	0.69

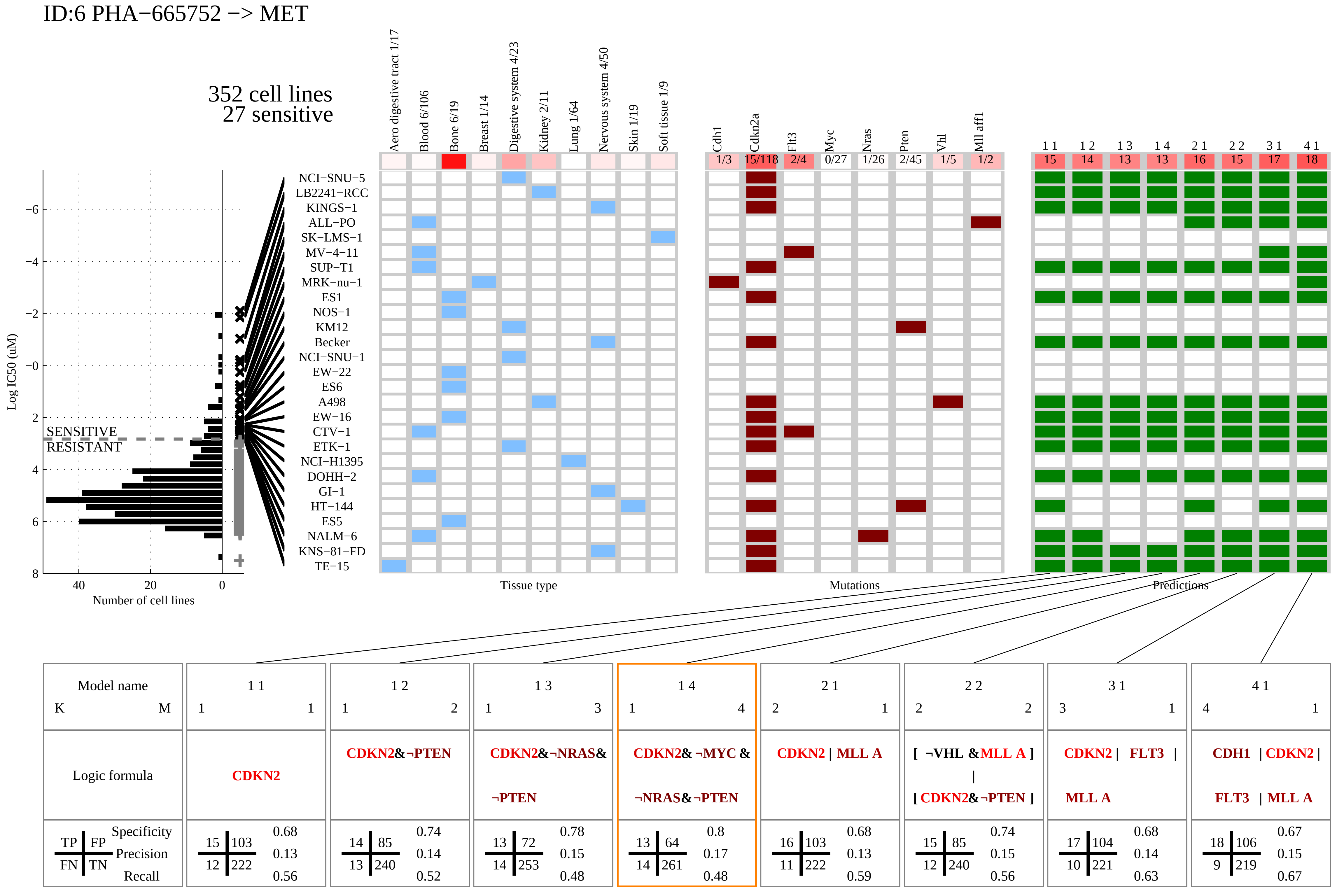

ID:6 PHA−665752 −> MET
352 cell lines
27 sensitive
Log IC50 (uM)
SENSITIVE
RESISTANT
Number of cell lines
Aero digestive tract 1/17
Blood 6/106
Bone 6/19
Breast 1/14
Digestive system 4/23
Kidney 2/11
Lung 1/64
Nervous system 4/50
Skin 1/19
Soft tissue 1/9
Cdh1 1/3
Cdkn2a 15/118
Flt3 2/4
Myc 0/27
Nras 1/26
Pten 2/45
Vhl 1/5
Mll aff1 1/2
1 1 15
1 2 14
1 3 13
1 4 13
2 1 16
2 2 15
3 1 17
4 1 18
NCI−SNU−5
LB2241−RCC
KINGS−1
ALL−PO
SK−LMS−1
MV−4−11
SUP−T1
MRK−nu−1
ES1
NOS−1
KM12
Becker
NCI−SNU−1
EW−22
ES6
A498
EW−16
CTV−1
ETK−1
NCI−H1395
DOHH−2
GI−1
HT−144
ES5
NALM−6
KNS−81−FD
TE−15
Tissue type
Mutations
Predictions
Model name
K M
Logic formula
TP FP FN TN
Specificity Precision Recall
1 1 — 1 1 — CDKN2 — 15 103 12 222 — 0.68 0.13 0.56
1 2 — 1 2 — CDKN2&¬PTEN — 14 85 13 240 — 0.74 0.14 0.52
1 3 — 1 3 — CDKN2&¬NRAS&¬PTEN — 13 72 14 253 — 0.78 0.15 0.48
1 4 — 1 4 — CDKN2&¬MYC&¬NRAS&¬PTEN — 13 64 14 261 — 0.8 0.17 0.48
2 1 — 2 1 — CDKN2 | MLL A — 16 103 11 222 — 0.68 0.13 0.59
2 2 — 2 2 — [¬VHL &MLL A] | [CDKN2&¬PTEN] — 15 85 12 240 — 0.74 0.15 0.56
3 1 — 3 1 — CDKN2 | FLT3 | MLL A — 17 104 10 221 — 0.68 0.14 0.63
4 1 — 4 1 — CDH1 | CDKN2 | FLT3 | MLL A — 18 106 9 219 — 0.67 0.15 0.67

ID:88 MS−275 −> HDAC

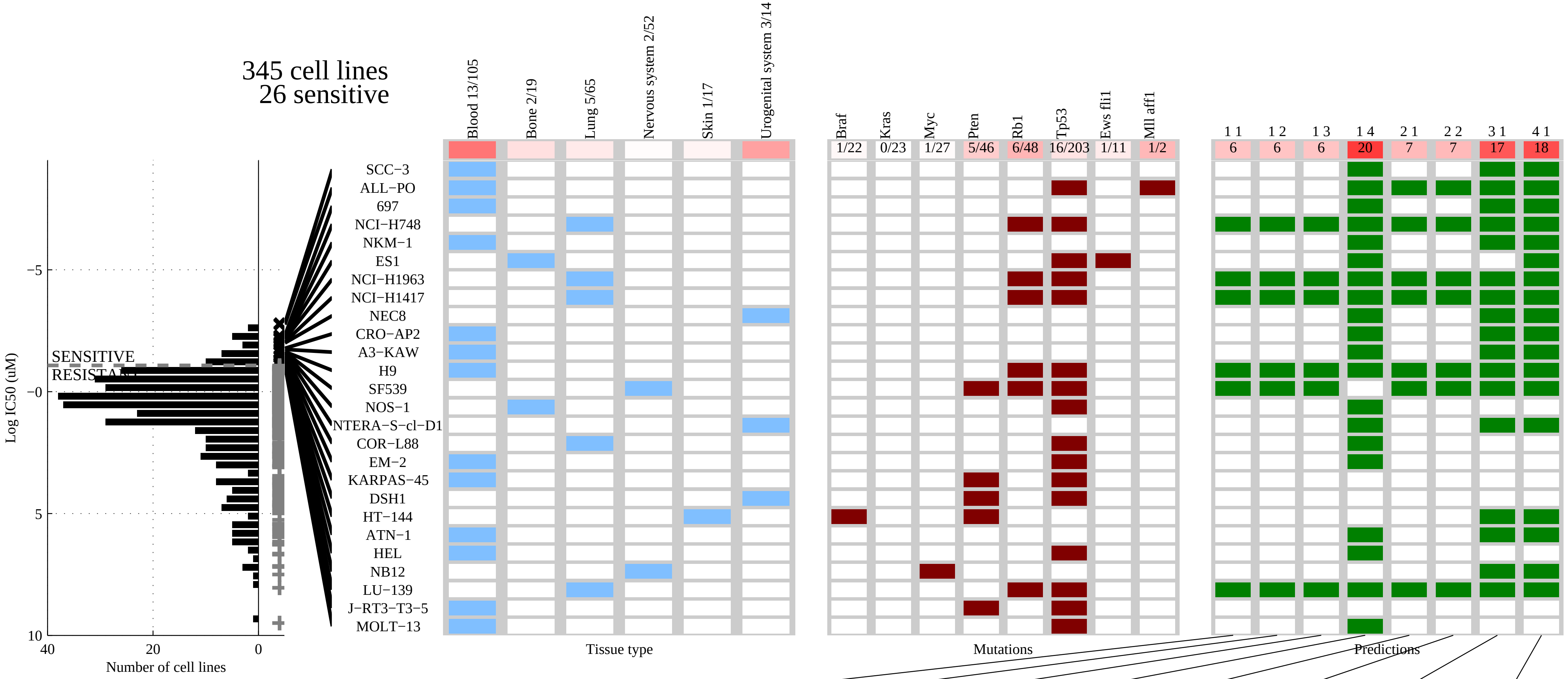

Model name (K, M)	1 1 (1, 1)	1 2 (1, 2)	1 3 (1, 3)	1 4 (1, 4)	2 1 (2, 1)	2 2 (2, 2)	3 1 (3, 1)	4 1 (4, 1)
Logic formula	RB1	RB1 & TP53	¬MYC & RB1 & TP53	¬BRAF&¬KRAS& ¬MYC &¬PTEN	RB1 | MLL A	[RB1 & TP53] | [TP53 & MLL A]	RB1 | ¬TP53 | MLL A	RB1 | ¬TP53 | EWS F | MLL A
TP	6	6	6	20	7	7	17	18
FP	42	30	22	217	43	30	162	171
FN	20	20	20	6	19	19	9	8
TN	277	289	297	102	276	289	157	148
Specificity	0.87	0.91	0.93	0.32	0.87	0.91	0.49	0.46
Precision	0.13	0.17	0.21	0.084	0.14	0.19	0.095	0.095
Recall	0.23	0.23	0.23	0.77	0.27	0.27	0.65	0.69

ID:9 MG−132 -> Proteasome

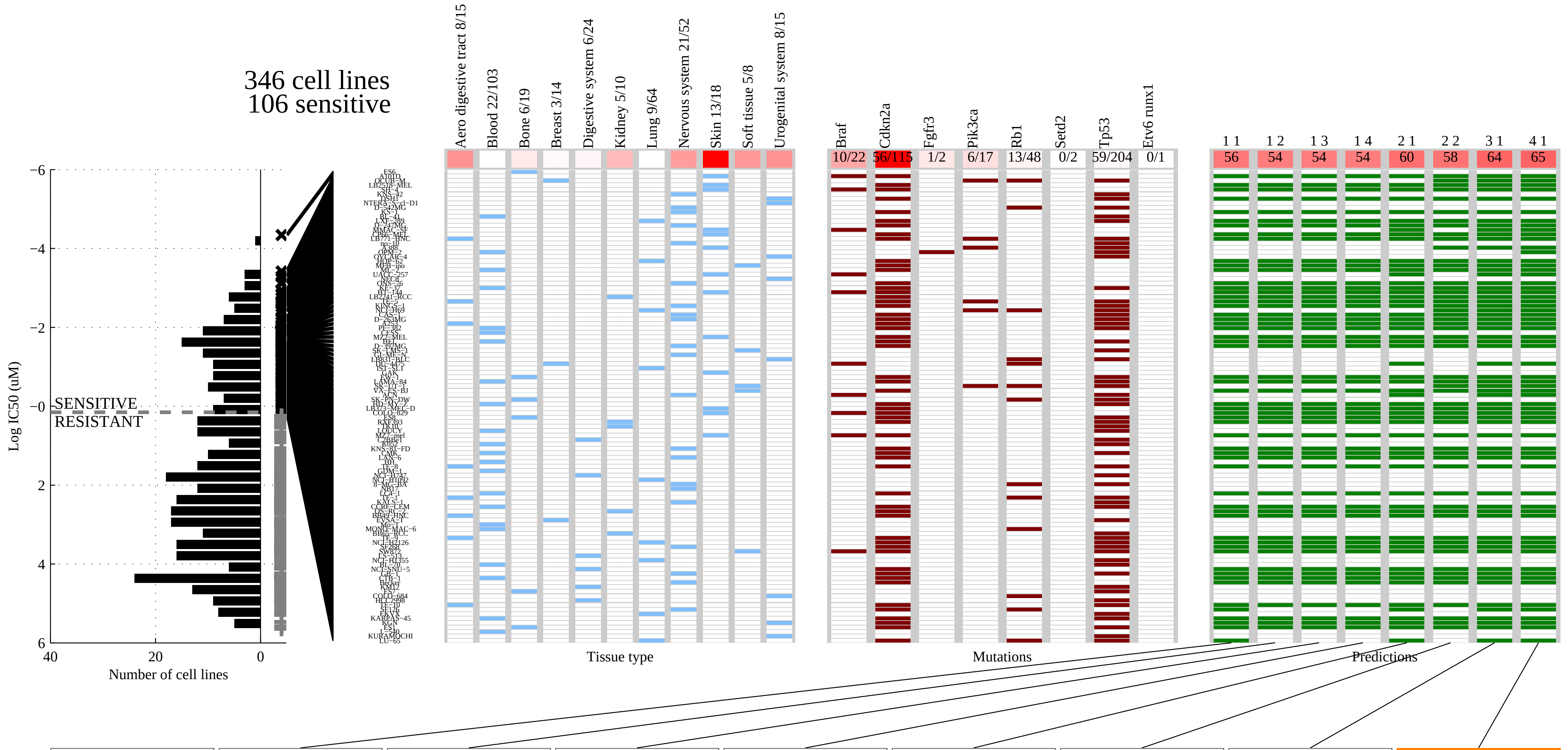

Model name (K, M)	1 1	1 2	1 3	1 4	2 1	2 2	3 1	4 1
K / M	1 / 1	1 / 2	1 / 3	1 / 4	2 / 1	2 / 2	3 / 1	4 / 1
Logic formula	**CDKN2**	**CDKN2& ¬RB1**	**CDKN2& ¬RB1 & ¬SETD2**	**CDKN2& ¬RB1 & ¬SETD2&¬ETV6**	**BRAF | CDKN2**	**[PIK3C & TP53] | [CDKN2& ¬RB1]**	**BRAF | CDKN2 | PIK3C**	**BRAF | CDKN2 | FGFR3 | PIK3C**
TP / FP	56 / 59	54 / 54	54 / 52	54 / 51	60 / 66	58 / 60	64 / 74	65 / 74
FN / TN	50 / 181	52 / 186	52 / 188	52 / 189	46 / 174	48 / 180	42 / 166	41 / 166
Specificity	0.75	0.78	0.78	0.79	0.72	0.75	0.69	0.69
Precision	0.49	0.5	0.51	0.51	0.48	0.49	0.46	0.47
Recall	0.53	0.51	0.51	0.51	0.57	0.55	0.6	0.61
