# Supplementary material for: Logic models to predict continuous outputs based on binary inputs with an application to personalized cancer therapy
Source: Sci Rep. 2016 Nov 23;6:36812. doi: 10.1038/srep36812 (PMC5120272; doi:10.1038/srep36812)
Supplement: Supplementary Dataset 3 [file srep36812-s4.zip › SD3.pdf]

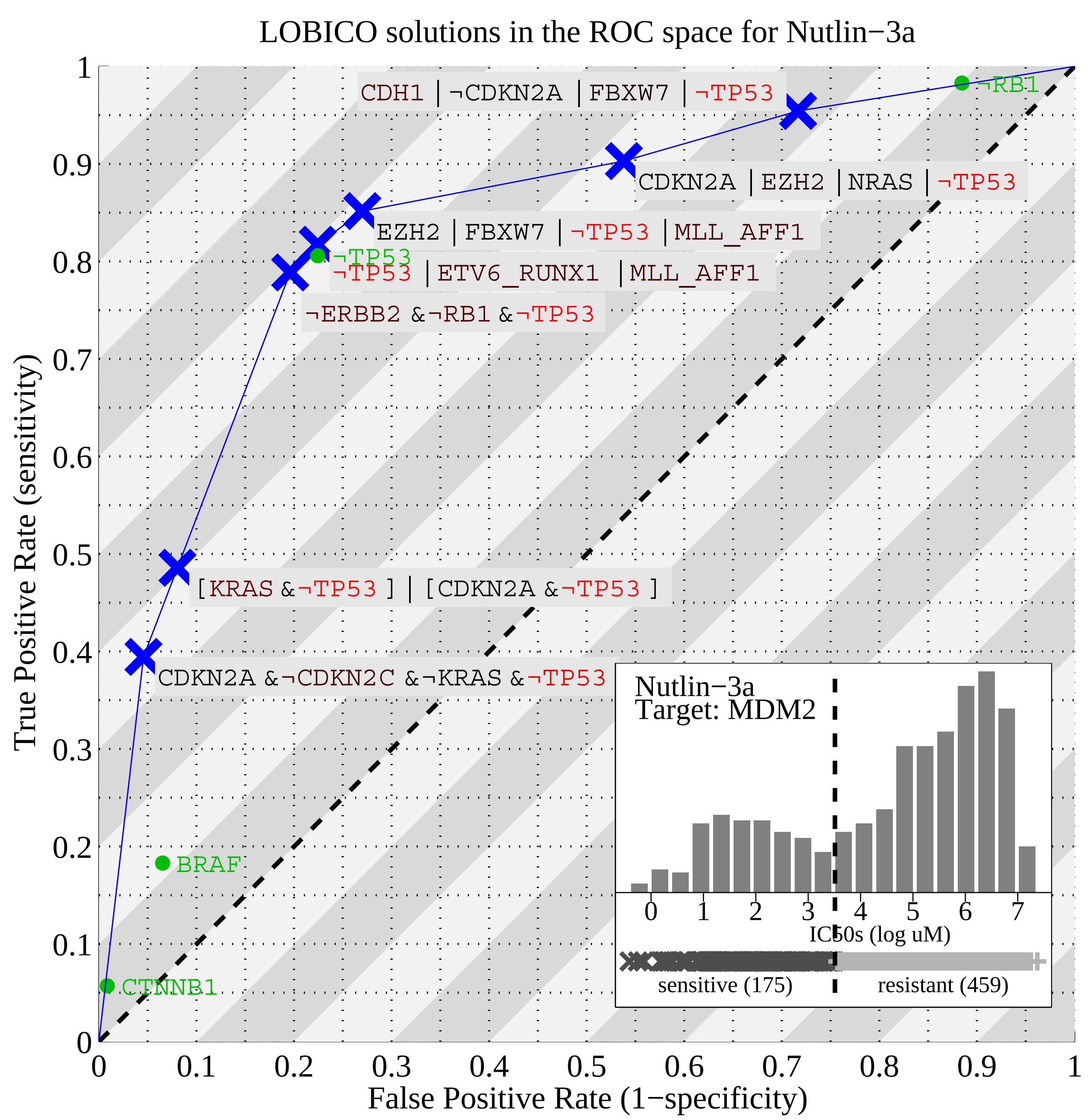
LOBICO solutions in the ROC space for Nutlin−3a
CDH1 | ¬CDKN2A | FBXW7 | ¬TP53
¬RB1
CDKN2A | EZH2 | NRAS | ¬TP53
EZH2 | FBXW7 | ¬TP53 | MLL_AFF1
¬TP53
¬TP53 | ETV6_RUNX1 | MLL_AFF1
¬ERBB2 & ¬RB1 & ¬TP53
[KRAS & ¬TP53] | [CDKN2A & ¬TP53]
CDKN2A & ¬CDKN2C & ¬KRAS & ¬TP53
BRAF
CTNNB1
Nutlin−3a
Target: MDM2
IC50s (log uM)
sensitive (175)
resistant (459)
True Positive Rate (sensitivity)
False Positive Rate (1−specificity)

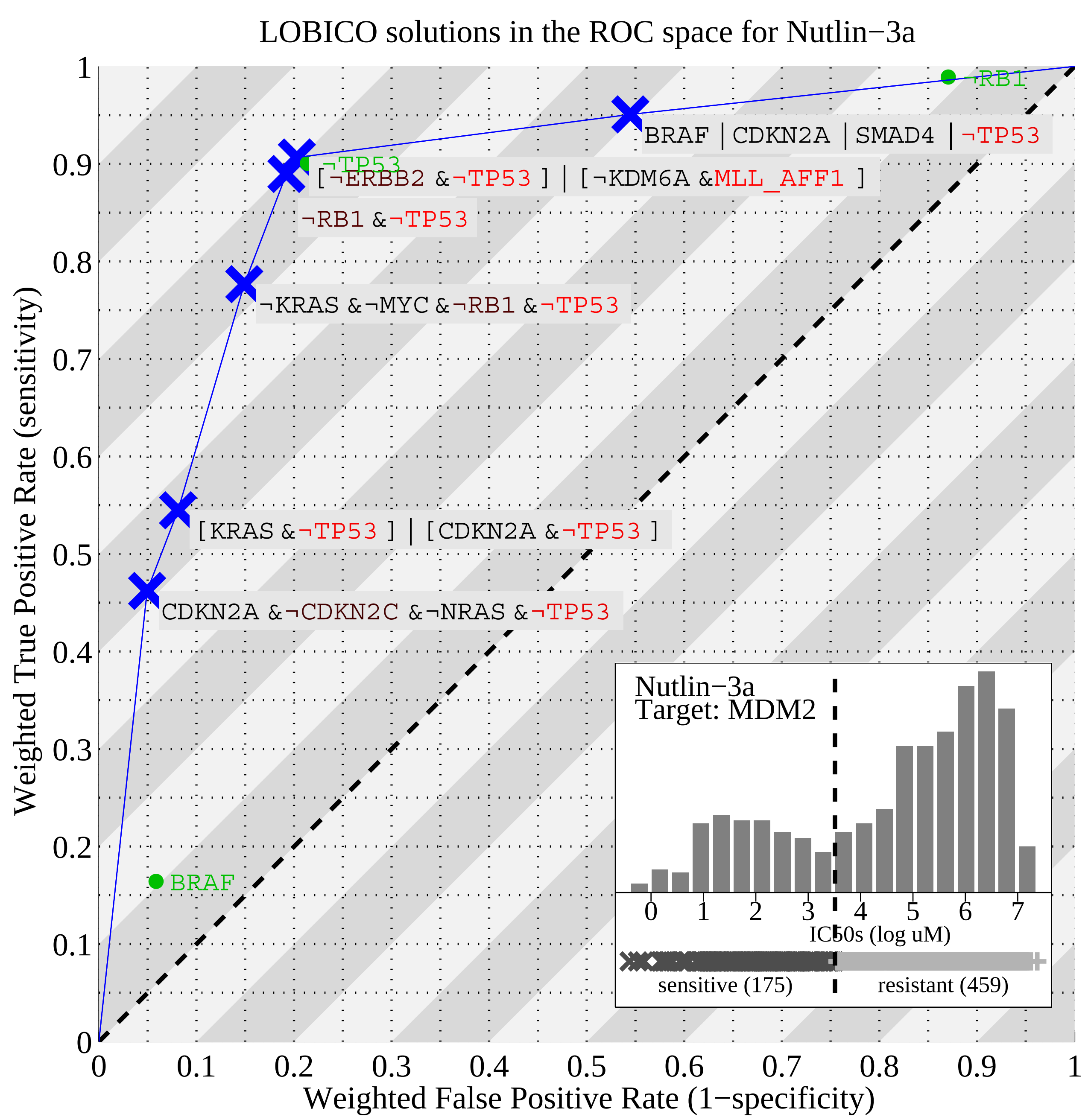

LOBICO solutions in the ROC space for Nutlin−3a
Weighted True Positive Rate (sensitivity)
Weighted False Positive Rate (1−specificity)
0
0.1
0.2
0.3
0.4
0.5
0.6
0.7
0.8
0.9
1
¬RB1
BRAF | CDKN2A | SMAD4 | ¬TP53
¬TP53
[¬ERBB2 & ¬TP53] | [¬KDM6A & MLL_AFF1]
¬RB1 & ¬TP53
¬KRAS & ¬MYC & ¬RB1 & ¬TP53
[KRAS & ¬TP53] | [CDKN2A & ¬TP53]
CDKN2A & ¬CDKN2C & ¬NRAS & ¬TP53
BRAF
Nutlin−3a
Target: MDM2
0
1
2
3
4
5
6
7
IC50s (log uM)
sensitive (175)
resistant (459)

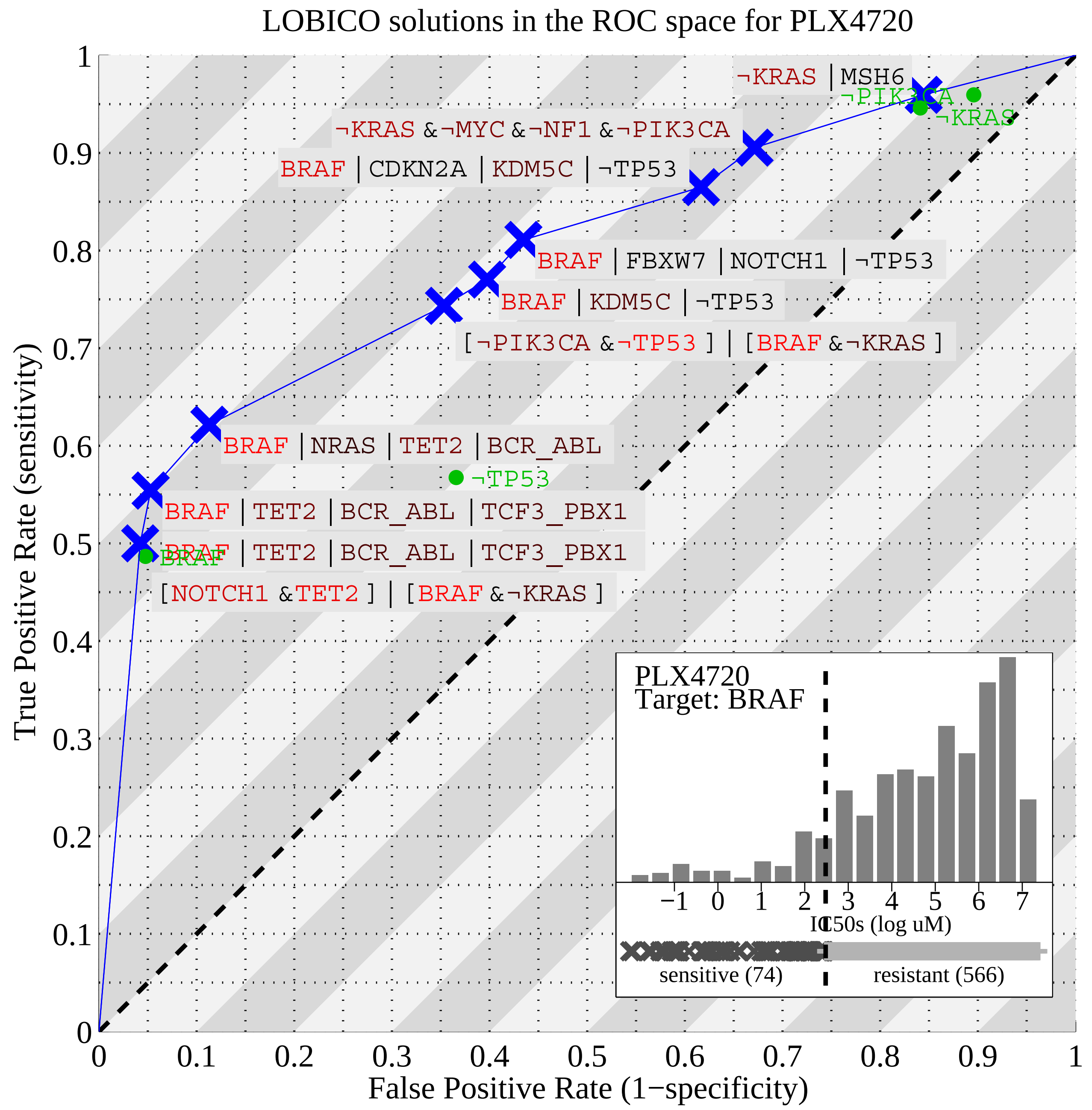

LOBICO solutions in the ROC space for PLX4720
True Positive Rate (sensitivity)
False Positive Rate (1−specificity)
0
0.1
0.2
0.3
0.4
0.5
0.6
0.7
0.8
0.9
1
¬KRAS | MSH6
¬PIK3CA
¬KRAS
¬KRAS &¬MYC &¬NF1 &¬PIK3CA
BRAF | CDKN2A | KDM5C | ¬TP53
BRAF | FBXW7 | NOTCH1 | ¬TP53
BRAF | KDM5C | ¬TP53
[¬PIK3CA &¬TP53] | [BRAF &¬KRAS]
BRAF | NRAS | TET2 | BCR_ABL
¬TP53
BRAF | TET2 | BCR_ABL | TCF3_PBX1
BRAF | TET2 | BCR_ABL | TCF3_PBX1
BRAF
[NOTCH1 &TET2] | [BRAF &¬KRAS]
PLX4720
Target: BRAF
−1
0
1
2
3
4
5
6
7
IC50s (log uM)
sensitive (74)
resistant (566)

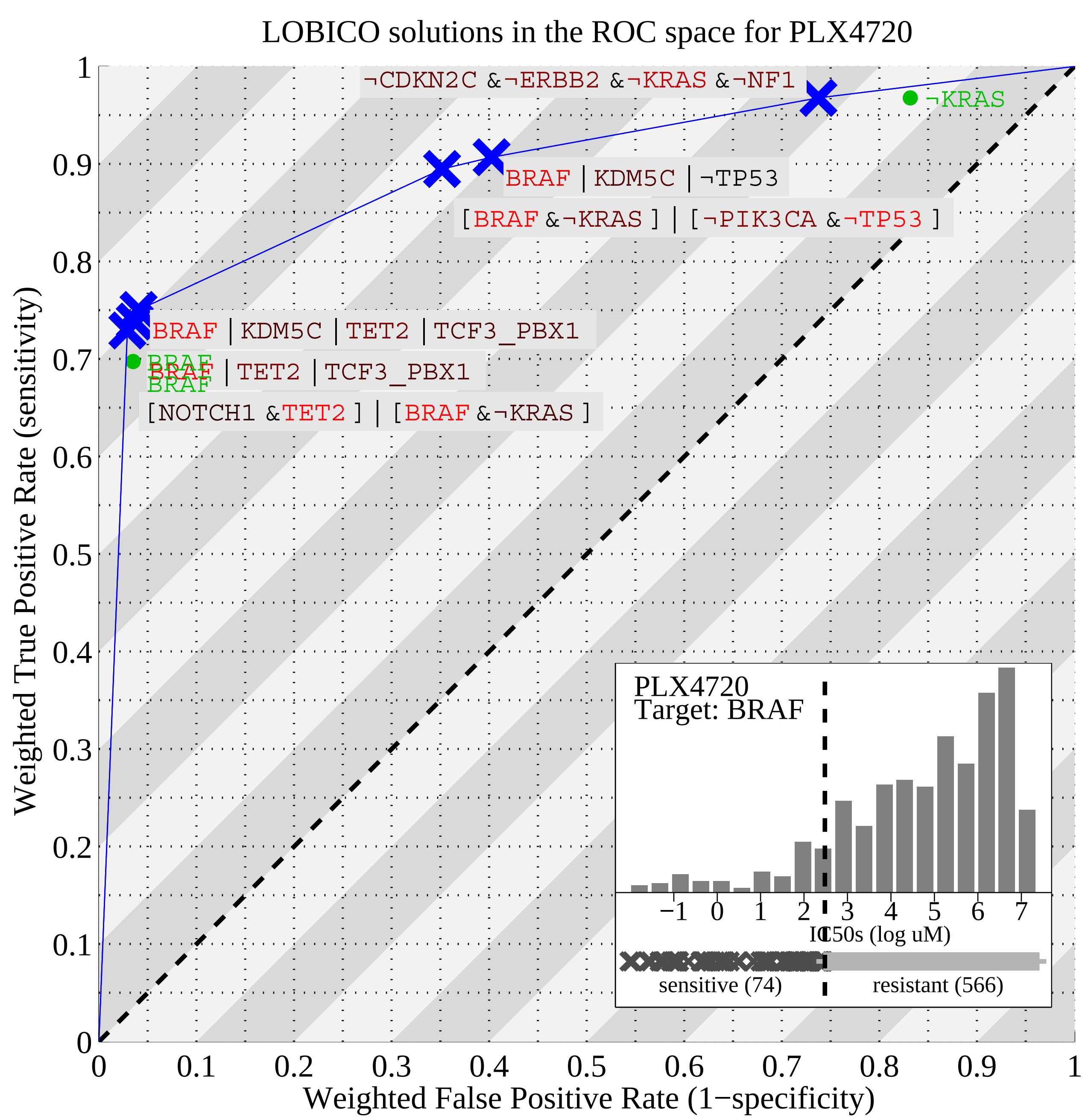

LOBICO solutions in the ROC space for PLX4720
¬CDKN2C &¬ERBB2 &¬KRAS &¬NF1
¬KRAS
BRAF |KDM5C |¬TP53
[BRAF &¬KRAS] | [¬PIK3CA &¬TP53]
BRAF |KDM5C |TET2 |TCF3_PBX1
BRAF
BRAF |TET2 |TCF3_PBX1
[NOTCH1 &TET2] | [BRAF &¬KRAS]
PLX4720
Target: BRAF
IC50s (log uM)
sensitive (74)
resistant (566)
Weighted True Positive Rate (sensitivity)
Weighted False Positive Rate (1−specificity)

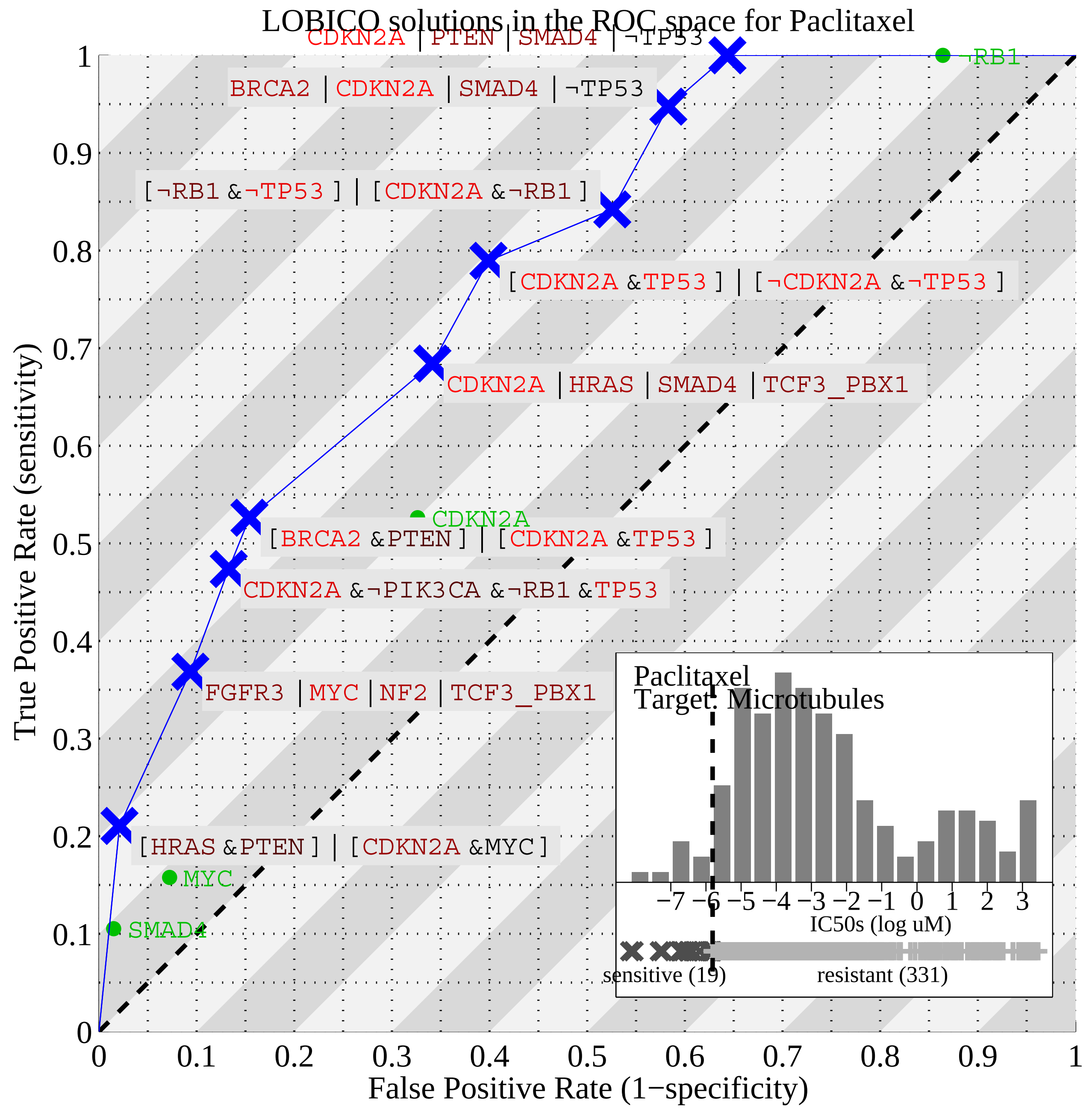

LOBICO solutions in the ROC space for Paclitaxel
CDKN2A | PTEN | SMAD4 | ¬TP53
BRCA2 | CDKN2A | SMAD4 | ¬TP53
[¬RB1 & ¬TP53] | [CDKN2A & ¬RB1]
[CDKN2A & TP53] | [¬CDKN2A & ¬TP53]
CDKN2A | HRAS | SMAD4 | TCF3_PBX1
[BRCA2 & PTEN] | [CDKN2A & TP53]
CDKN2A & ¬PIK3CA & ¬RB1 & TP53
FGFR3 | MYC | NF2 | TCF3_PBX1
[HRAS & PTEN] | [CDKN2A & MYC]
¬RB1
CDKN2A
MYC
SMAD4
Paclitaxel
Target: Microtubules
−7 −6 −5 −4 −3 −2 −1 0 1 2 3
IC50s (log uM)
sensitive (19)
resistant (331)
True Positive Rate (sensitivity)
False Positive Rate (1−specificity)
0 0.1 0.2 0.3 0.4 0.5 0.6 0.7 0.8 0.9 1

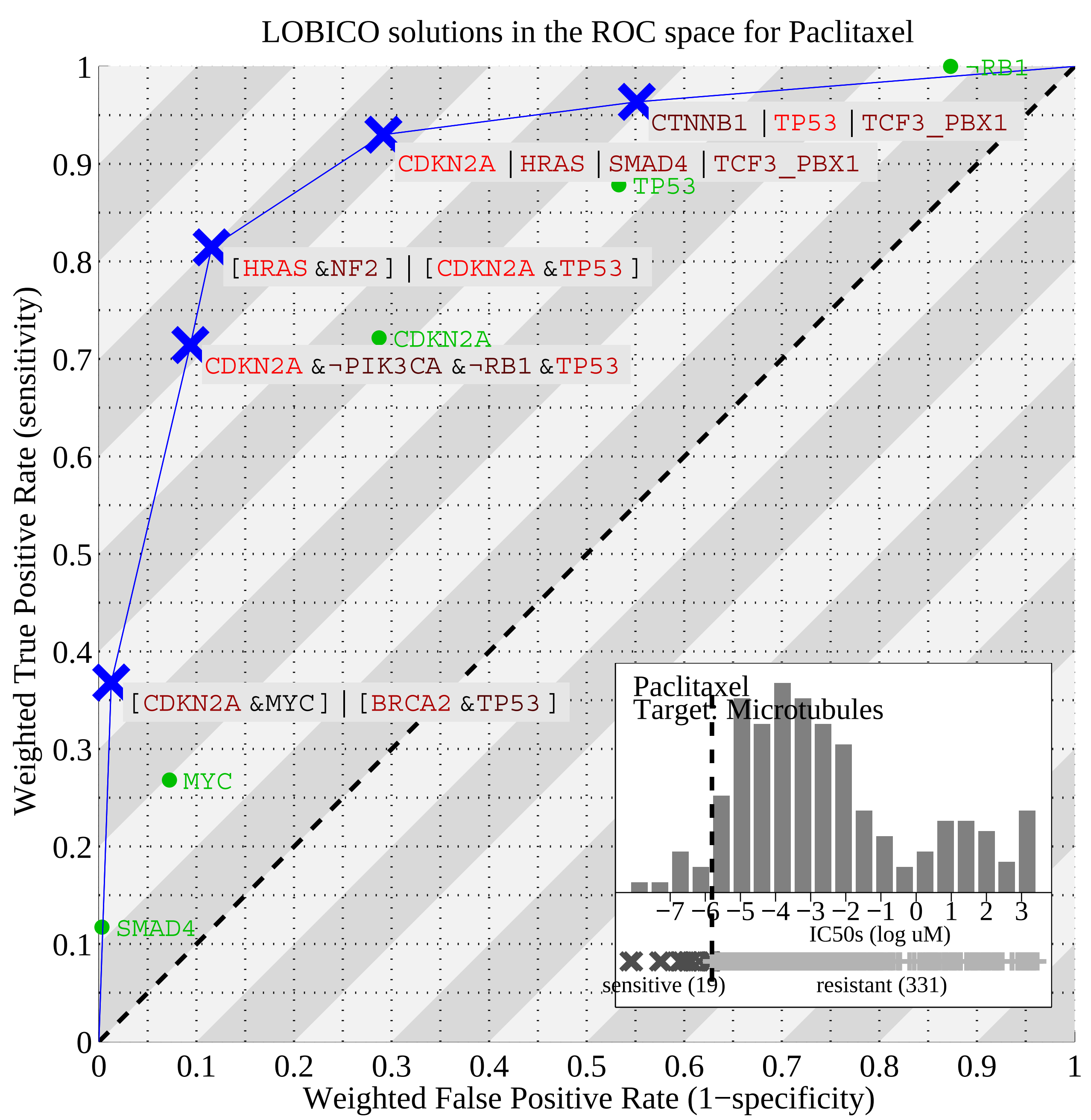

LOBICO solutions in the ROC space for Paclitaxel
Weighted True Positive Rate (sensitivity)
Weighted False Positive Rate (1−specificity)
0
0.1
0.2
0.3
0.4
0.5
0.6
0.7
0.8
0.9
1
¬RB1
CTNNB1 | TP53 | TCF3_PBX1
CDKN2A | HRAS | SMAD4 | TCF3_PBX1
TP53
[HRAS & NF2] | [CDKN2A & TP53]
CDKN2A
CDKN2A & ¬PIK3CA & ¬RB1 & TP53
[CDKN2A & MYC] | [BRCA2 & TP53]
MYC
SMAD4
Paclitaxel
Target: Microtubules
−7 −6 −5 −4 −3 −2 −1 0 1 2 3
IC50s (log uM)
sensitive (19)
resistant (331)

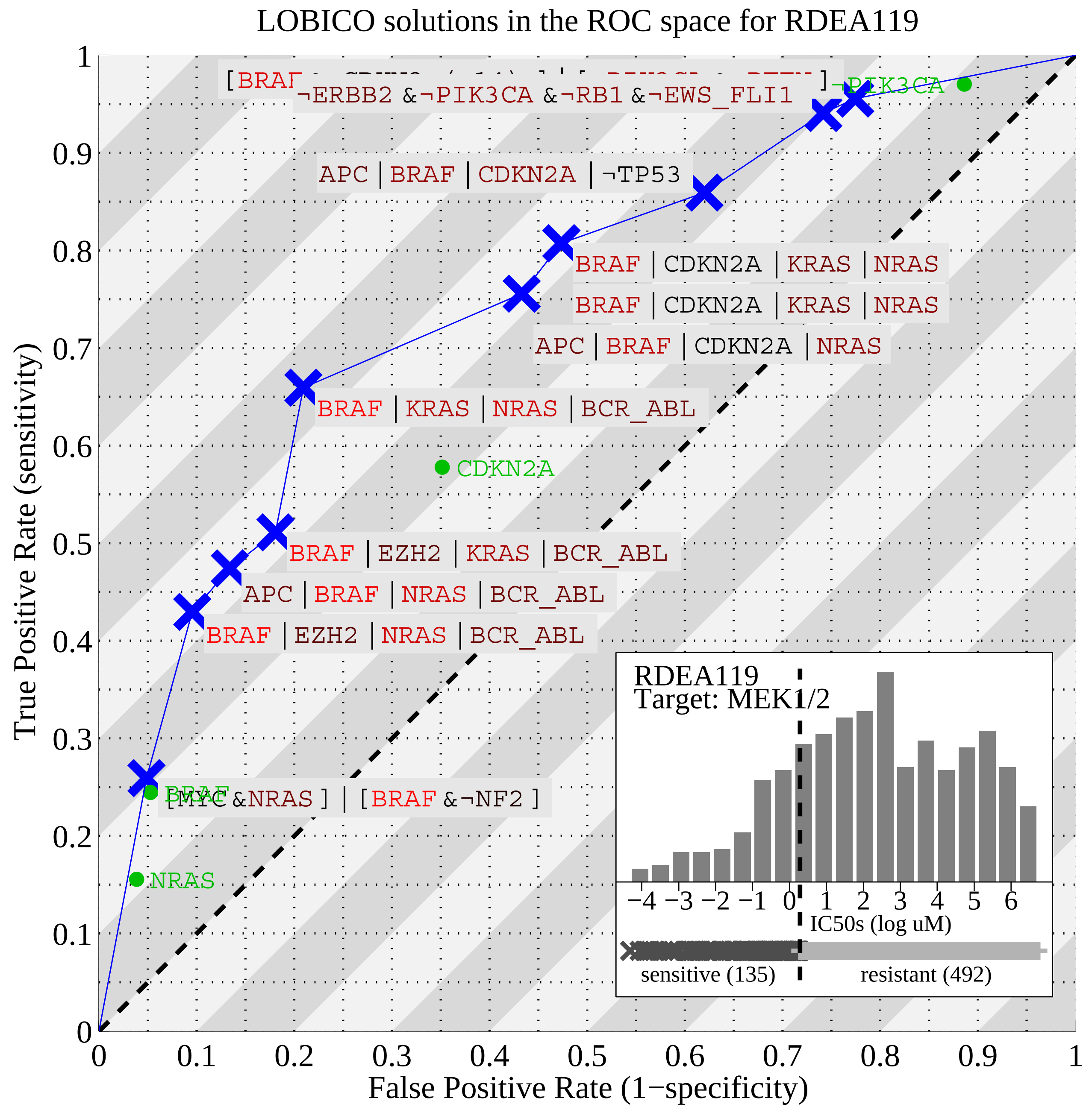

LOBICO solutions in the ROC space for RDEA119
True Positive Rate (sensitivity)
False Positive Rate (1−specificity)
¬ERBB2 &¬PIK3CA &¬RB1 &¬EWS_FLI1
¬PIK3CA
APC | BRAF | CDKN2A | ¬TP53
BRAF | CDKN2A | KRAS | NRAS
BRAF | CDKN2A | KRAS | NRAS
APC | BRAF | CDKN2A | NRAS
BRAF | KRAS | NRAS | BCR_ABL
CDKN2A
BRAF | EZH2 | KRAS | BCR_ABL
APC | BRAF | NRAS | BCR_ABL
BRAF | EZH2 | NRAS | BCR_ABL
BRAF
[MYC &NRAS] | [BRAF &¬NF2]
NRAS
RDEA119
Target: MEK1/2
IC50s (log uM)
sensitive (135)
resistant (492)

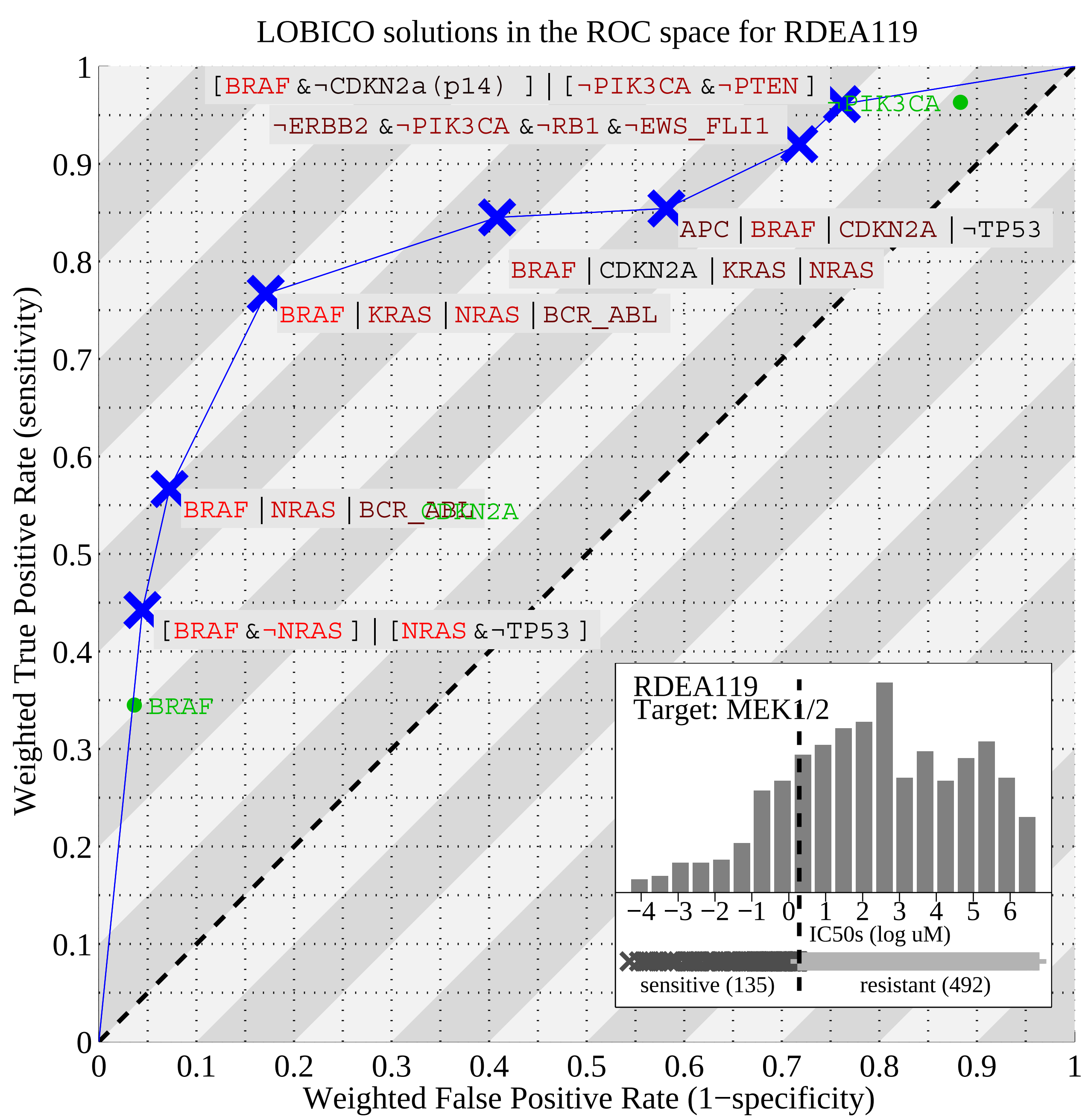

LOBICO solutions in the ROC space for RDEA119
[BRAF &¬CDKN2a (p14)] | [¬PIK3CA &¬PTEN]
¬ERBB2 &¬PIK3CA &¬RB1 &¬EWS_FLI1
¬PIK3CA
APC | BRAF | CDKN2A | ¬TP53
BRAF | CDKN2A | KRAS | NRAS
BRAF | KRAS | NRAS | BCR_ABL
BRAF | NRAS | BCR_ABL
CDKN2A
[BRAF &¬NRAS] | [NRAS &¬TP53]
BRAF
RDEA119
Target: MEK1/2
IC50s (log uM)
sensitive (135)
resistant (492)
Weighted True Positive Rate (sensitivity)
Weighted False Positive Rate (1−specificity)

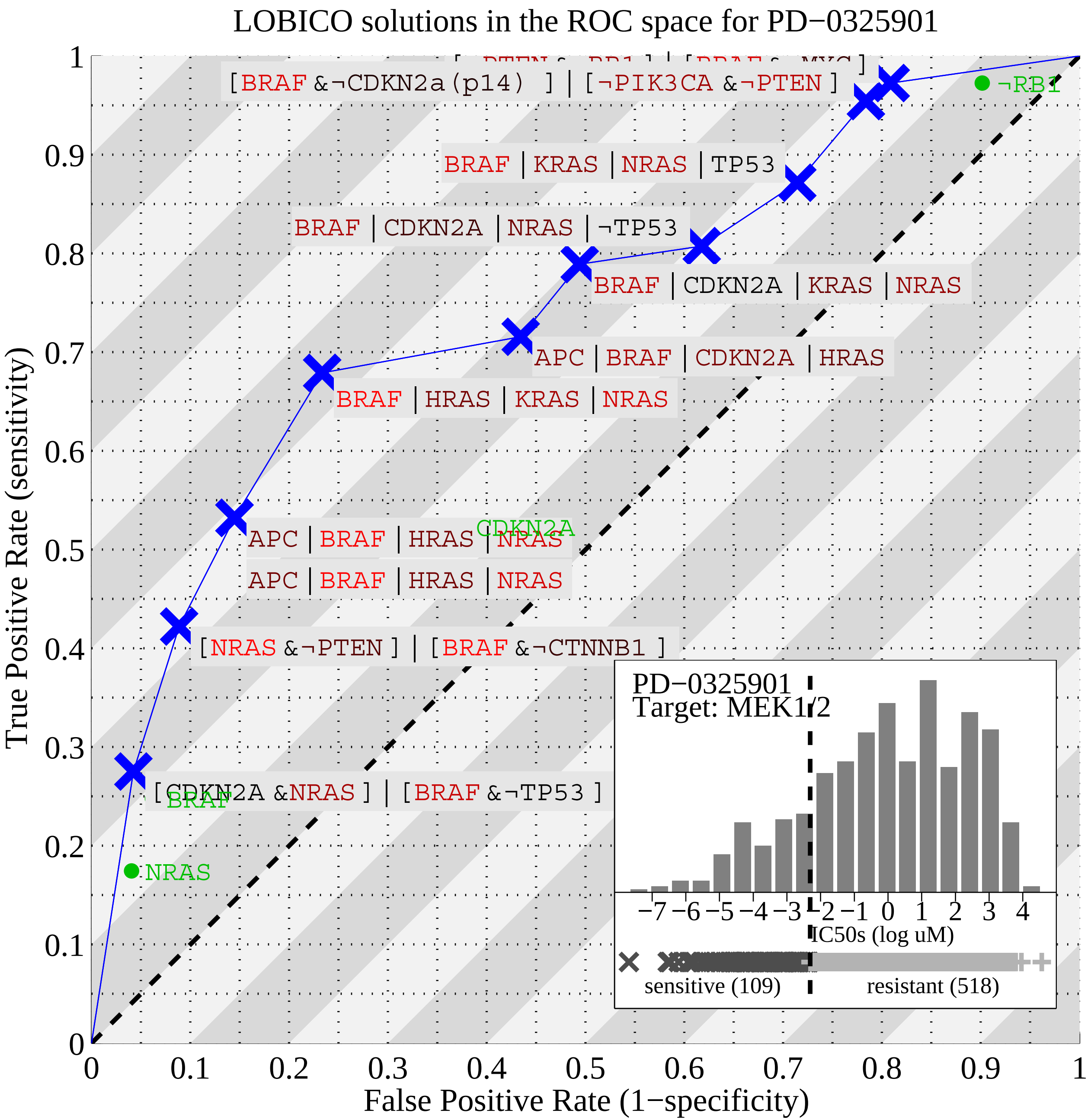
LOBICO solutions in the ROC space for PD−0325901
True Positive Rate (sensitivity)
False Positive Rate (1−specificity)
0
0.1
0.2
0.3
0.4
0.5
0.6
0.7
0.8
0.9
1
[BRAF &¬CDKN2a(p14)] | [¬PIK3CA &¬PTEN]
¬RB1
BRAF | KRAS | NRAS | TP53
BRAF | CDKN2A | NRAS | ¬TP53
BRAF | CDKN2A | KRAS | NRAS
APC | BRAF | CDKN2A | HRAS
BRAF | HRAS | KRAS | NRAS
APC | BRAF | HRAS | NRAS
CDKN2A
APC | BRAF | HRAS | NRAS
[NRAS &¬PTEN] | [BRAF &¬CTNNB1]
[CDKN2A &NRAS] | [BRAF &¬TP53]
BRAF
NRAS
PD−0325901
Target: MEK1/2
−7 −6 −5 −4 −3 −2 −1 0 1 2 3 4
IC50s (log uM)
sensitive (109)
resistant (518)

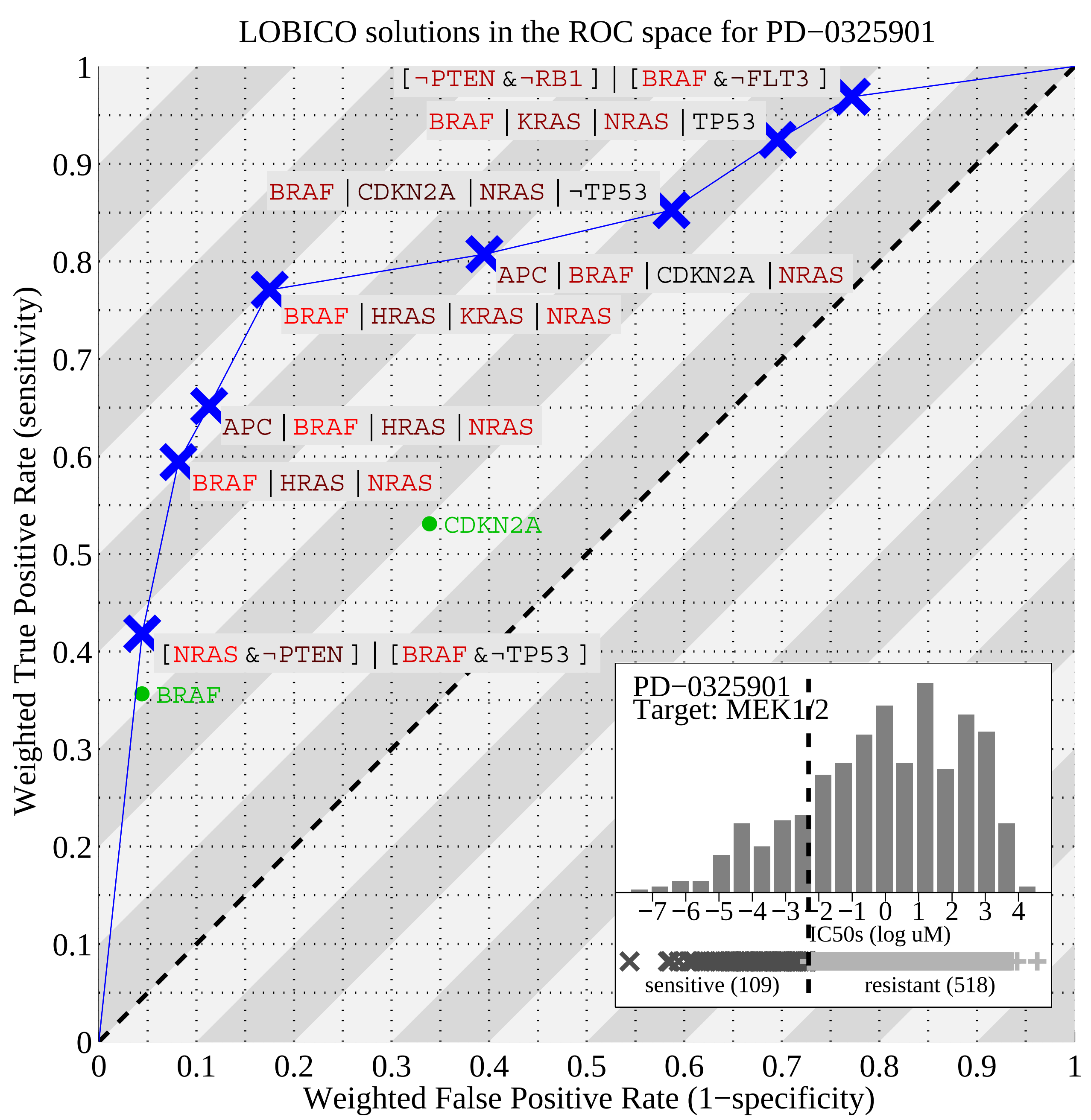
LOBICO solutions in the ROC space for PD−0325901
[¬PTEN &¬RB1] | [BRAF &¬FLT3]
BRAF | KRAS | NRAS | TP53
BRAF | CDKN2A | NRAS | ¬TP53
APC | BRAF | CDKN2A | NRAS
BRAF | HRAS | KRAS | NRAS
APC | BRAF | HRAS | NRAS
BRAF | HRAS | NRAS
CDKN2A
[NRAS &¬PTEN] | [BRAF &¬TP53]
BRAF
PD−0325901
Target: MEK1/2
−7 −6 −5 −4 −3 −2 −1 0 1 2 3 4
IC50s (log uM)
sensitive (109)
resistant (518)
Weighted True Positive Rate (sensitivity)
Weighted False Positive Rate (1−specificity)
0 0.1 0.2 0.3 0.4 0.5 0.6 0.7 0.8 0.9 1

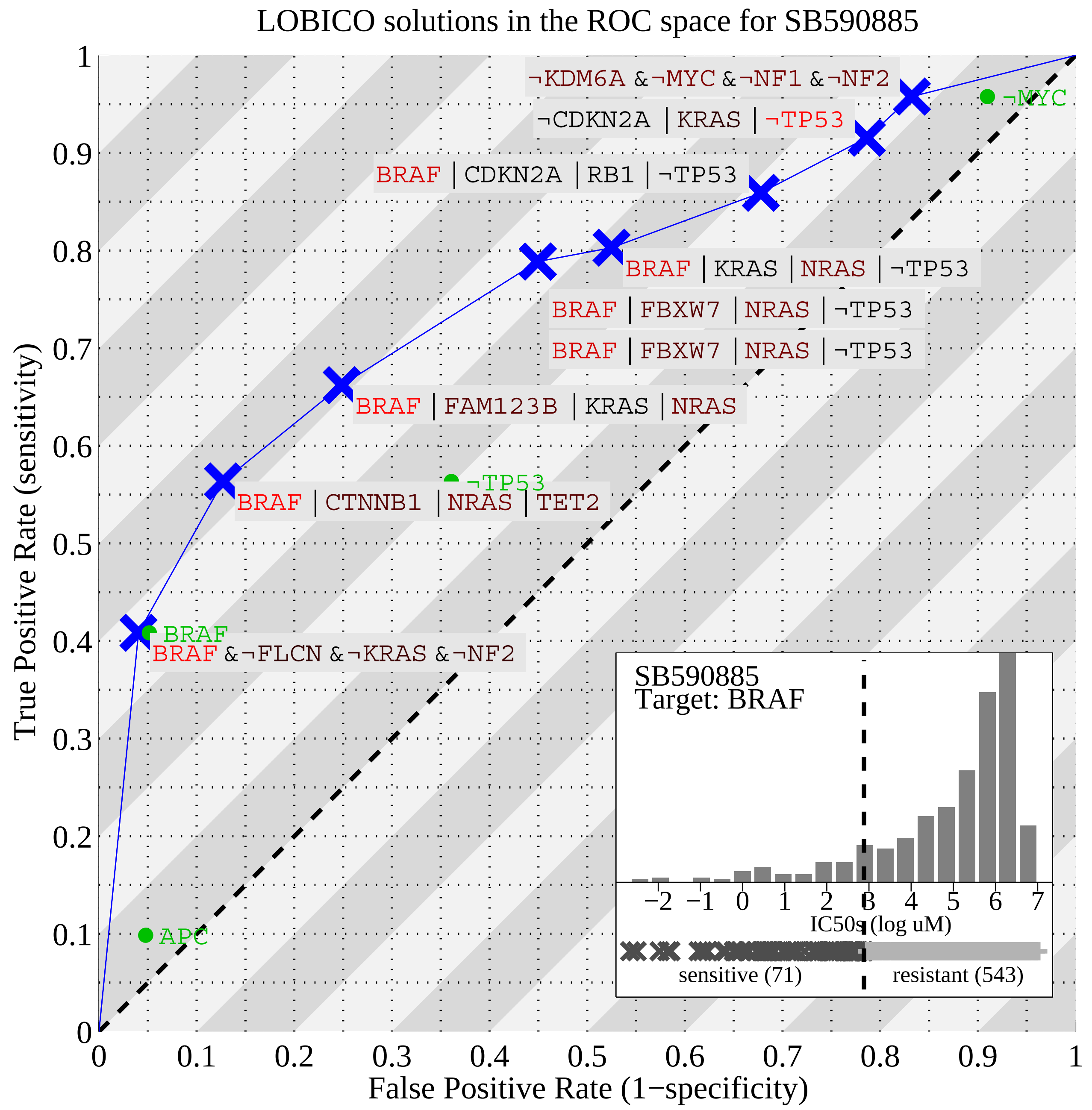
LOBICO solutions in the ROC space for SB590885
True Positive Rate (sensitivity)
False Positive Rate (1−specificity)
0
0.1
0.2
0.3
0.4
0.5
0.6
0.7
0.8
0.9
1
¬KDM6A & ¬MYC & ¬NF1 & ¬NF2
¬CDKN2A | KRAS | ¬TP53
BRAF | CDKN2A | RB1 | ¬TP53
BRAF | KRAS | NRAS | ¬TP53
BRAF | FBXW7 | NRAS | ¬TP53
BRAF | FBXW7 | NRAS | ¬TP53
BRAF | FAM123B | KRAS | NRAS
BRAF | CTNNB1 | NRAS | TET2
BRAF & ¬FLCN & ¬KRAS & ¬NF2
¬MYC
¬TP53
BRAF
APC
SB590885
Target: BRAF
−2
−1
0
1
2
3
4
5
6
7
IC50s (log uM)
sensitive (71)
resistant (543)

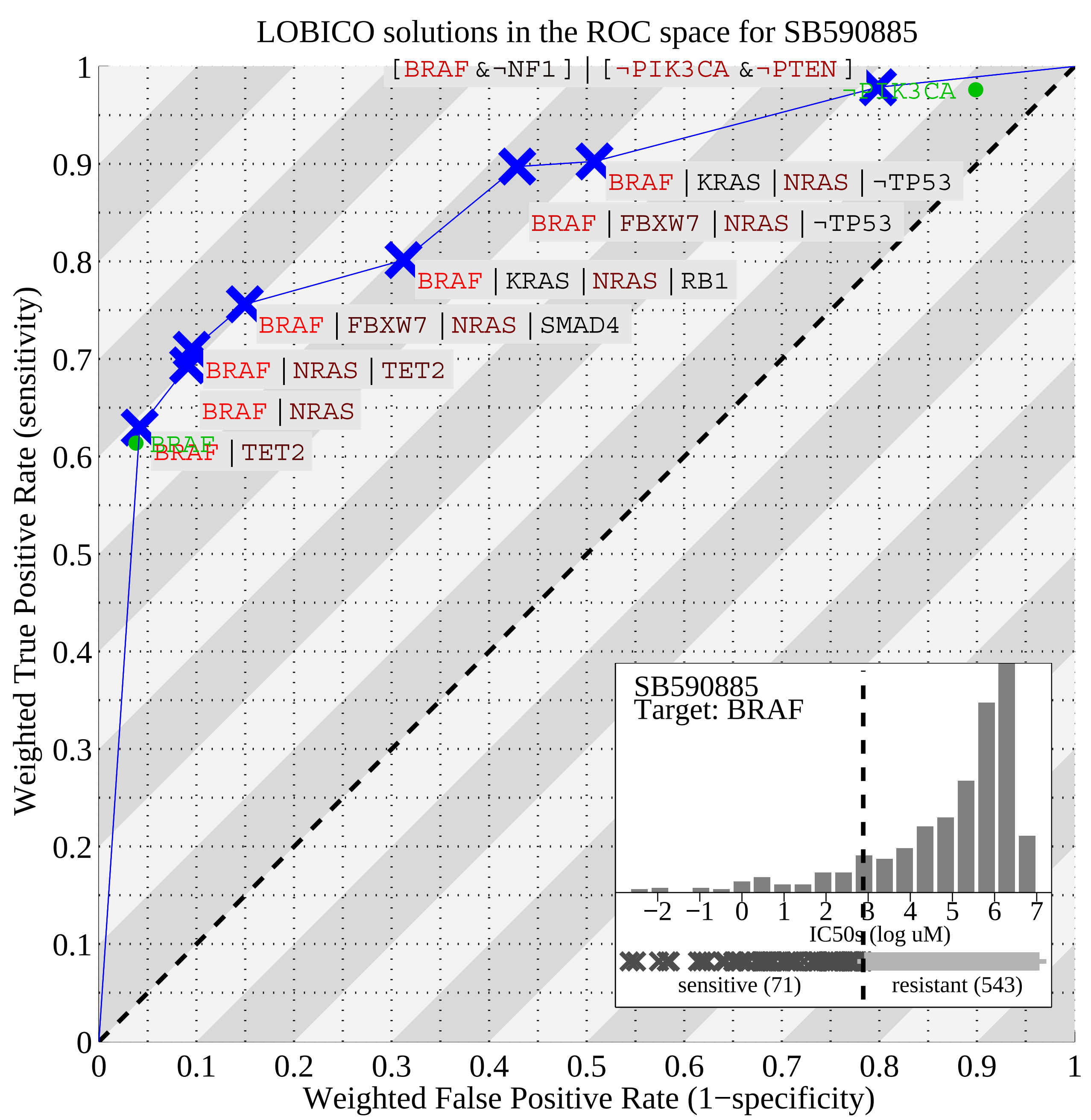
LOBICO solutions in the ROC space for SB590885
[BRAF &¬NF1] | [¬PIK3CA &¬PTEN]
¬PIK3CA
BRAF | KRAS | NRAS | ¬TP53
BRAF | FBXW7 | NRAS | ¬TP53
BRAF | KRAS | NRAS | RB1
BRAF | FBXW7 | NRAS | SMAD4
BRAF | NRAS | TET2
BRAF | NRAS
BRAF
BRAF | TET2
SB590885
Target: BRAF
IC50s (log uM)
sensitive (71)
resistant (543)
Weighted True Positive Rate (sensitivity)
Weighted False Positive Rate (1−specificity)

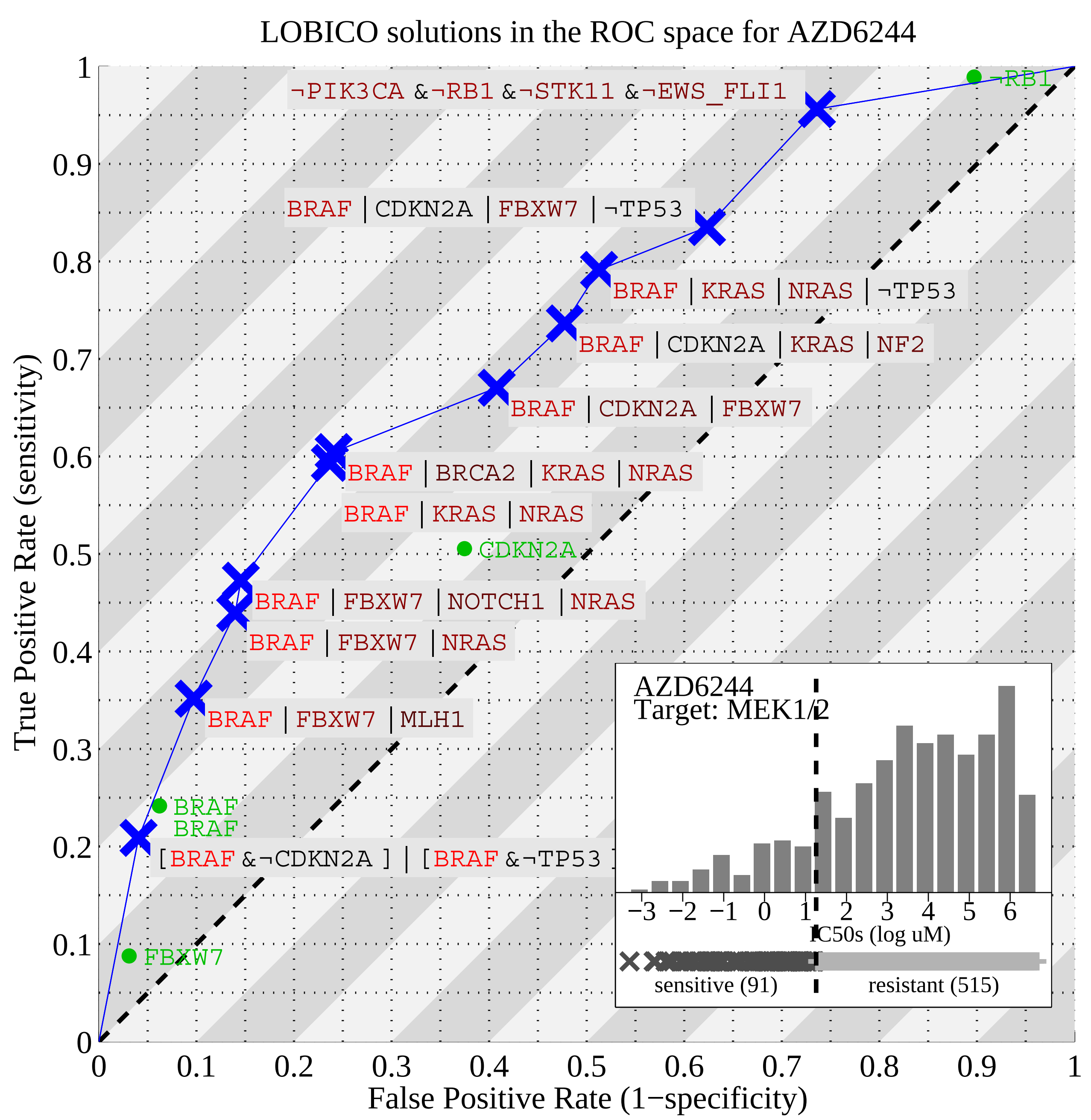

LOBICO solutions in the ROC space for AZD6244
True Positive Rate (sensitivity)
False Positive Rate (1−specificity)
¬PIK3CA &¬RB1 &¬STK11 &¬EWS_FLI1
¬RB1
BRAF | CDKN2A | FBXW7 | ¬TP53
BRAF | KRAS | NRAS | ¬TP53
BRAF | CDKN2A | KRAS | NF2
BRAF | CDKN2A | FBXW7
BRAF | BRCA2 | KRAS | NRAS
BRAF | KRAS | NRAS
CDKN2A
BRAF | FBXW7 | NOTCH1 | NRAS
BRAF | FBXW7 | NRAS
BRAF | FBXW7 | MLH1
BRAF
BRAF
[BRAF &¬CDKN2A] | [BRAF &¬TP53
FBXW7
AZD6244
Target: MEK1/2
IC50s (log uM)
sensitive (91)
resistant (515)

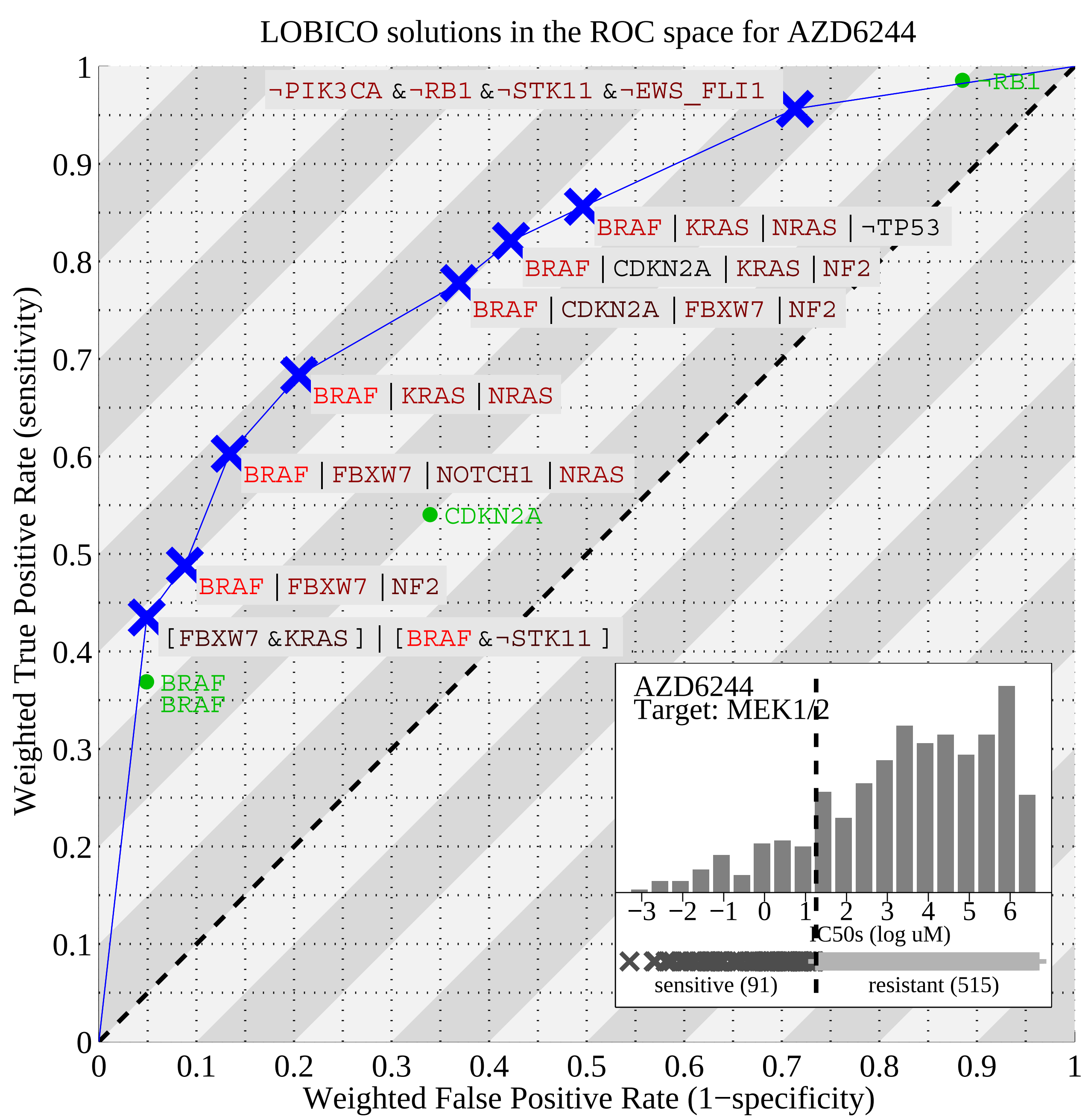

LOBICO solutions in the ROC space for AZD6244
¬PIK3CA &¬RB1 &¬STK11 &¬EWS_FLI1
¬RB1
BRAF | KRAS | NRAS | ¬TP53
BRAF | CDKN2A | KRAS | NF2
BRAF | CDKN2A | FBXW7 | NF2
BRAF | KRAS | NRAS
BRAF | FBXW7 | NOTCH1 | NRAS
CDKN2A
BRAF | FBXW7 | NF2
[FBXW7 &KRAS] | [BRAF &¬STK11]
BRAF
BRAF
AZD6244
Target: MEK1/2
IC50s (log uM)
sensitive (91)
resistant (515)
Weighted True Positive Rate (sensitivity)
Weighted False Positive Rate (1−specificity)

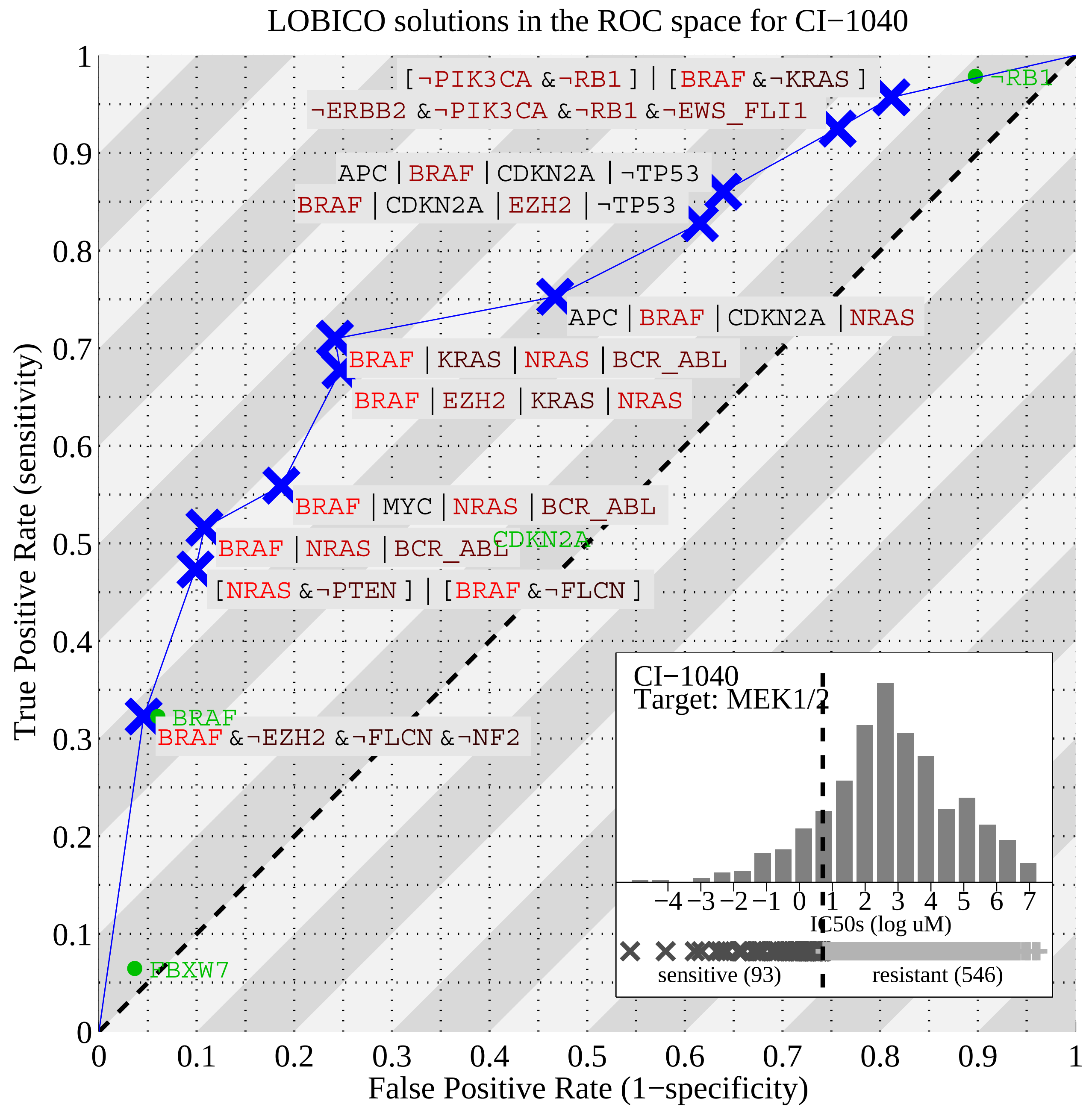

LOBICO solutions in the ROC space for CI−1040
True Positive Rate (sensitivity)
False Positive Rate (1−specificity)
[¬PIK3CA &¬RB1] | [BRAF &¬KRAS]
¬ERBB2 &¬PIK3CA &¬RB1 &¬EWS_FLI1
APC | BRAF | CDKN2A | ¬TP53
BRAF | CDKN2A | EZH2 | ¬TP53
APC | BRAF | CDKN2A | NRAS
BRAF | KRAS | NRAS | BCR_ABL
BRAF | EZH2 | KRAS | NRAS
BRAF | MYC | NRAS | BCR_ABL
BRAF | NRAS | BCR_ABL
CDKN2A
[NRAS &¬PTEN] | [BRAF &¬FLCN]
BRAF
BRAF &¬EZH2 &¬FLCN &¬NF2
FBXW7
¬RB1
CI−1040
Target: MEK1/2
IC50s (log uM)
sensitive (93)
resistant (546)

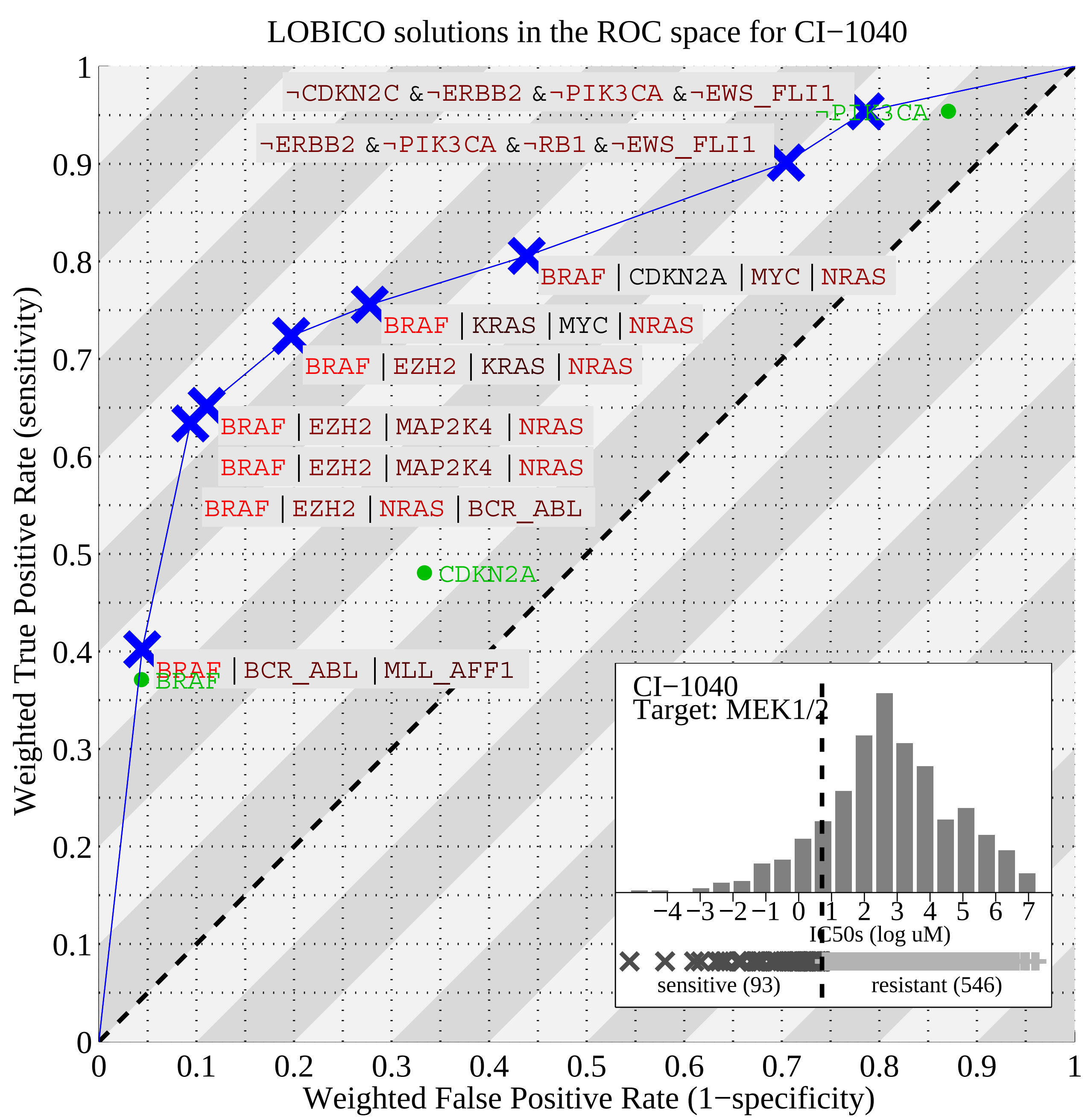

LOBICO solutions in the ROC space for CI−1040
Weighted True Positive Rate (sensitivity)
Weighted False Positive Rate (1−specificity)
¬CDKN2C &¬ERBB2 &¬PIK3CA &¬EWS_FLI1
¬PIK3CA
¬ERBB2 &¬PIK3CA &¬RB1 &¬EWS_FLI1
BRAF | CDKN2A | MYC | NRAS
BRAF | KRAS | MYC | NRAS
BRAF | EZH2 | KRAS | NRAS
BRAF | EZH2 | MAP2K4 | NRAS
BRAF | EZH2 | MAP2K4 | NRAS
BRAF | EZH2 | NRAS | BCR_ABL
CDKN2A
BRAF | BCR_ABL | MLL_AFF1
BRAF
CI−1040
Target: MEK1/2
IC50s (log uM)
sensitive (93)
resistant (546)

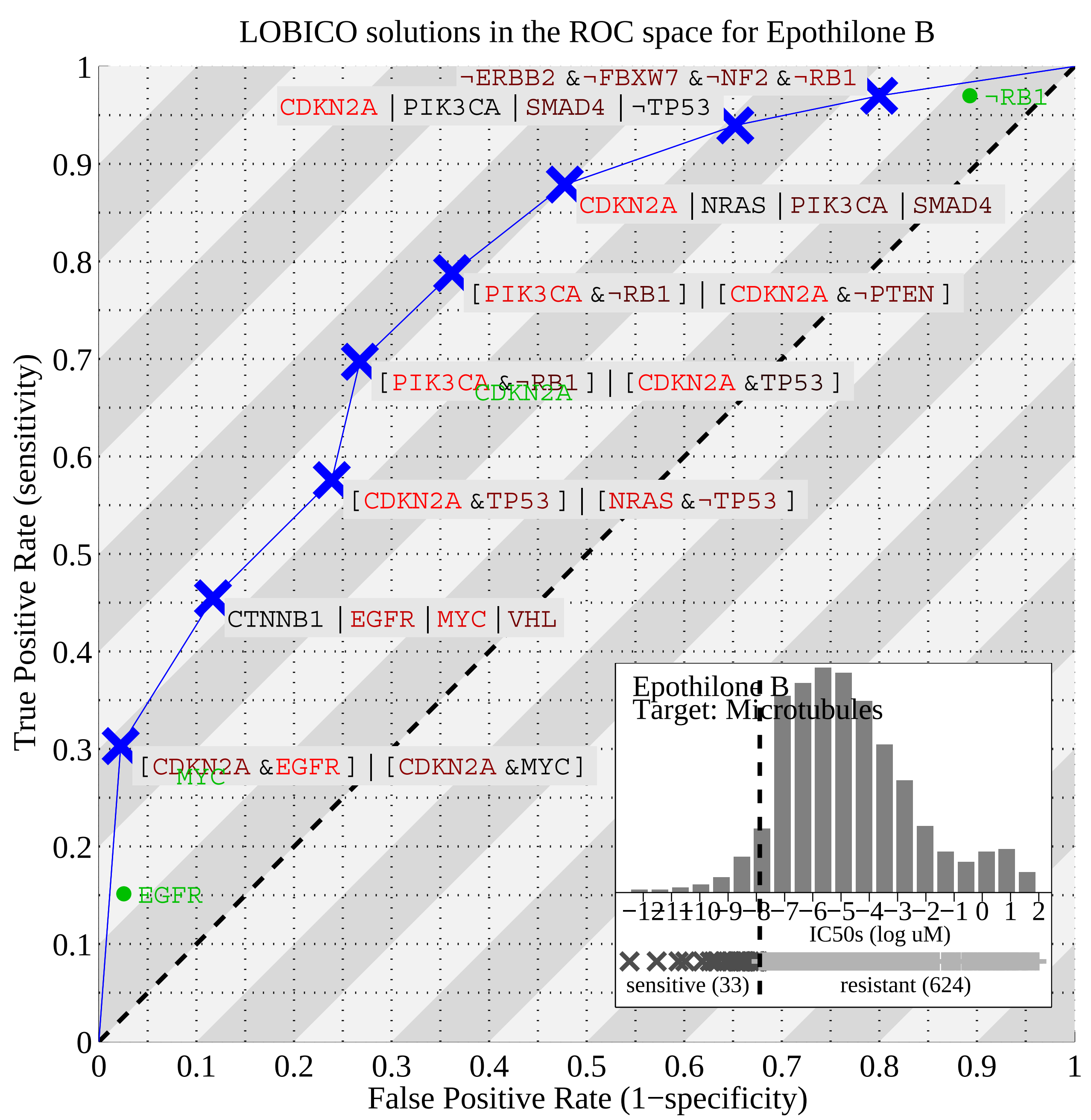
LOBICO solutions in the ROC space for Epothilone B
¬ERBB2 &¬FBXW7 &¬NF2 &¬RB1
CDKN2A |PIK3CA |SMAD4 |¬TP53
¬RB1
CDKN2A |NRAS |PIK3CA |SMAD4
[PIK3CA &¬RB1] | [CDKN2A &¬PTEN]
[PIK3CA &¬RB1] | [CDKN2A &TP53]
CDKN2A
[CDKN2A &TP53] | [NRAS &¬TP53]
CTNNB1 |EGFR |MYC |VHL
[CDKN2A &EGFR] | [CDKN2A &MYC]
MYC
EGFR
Epothilone B
Target: Microtubules
IC50s (log uM)
sensitive (33)
resistant (624)
True Positive Rate (sensitivity)
False Positive Rate (1−specificity)

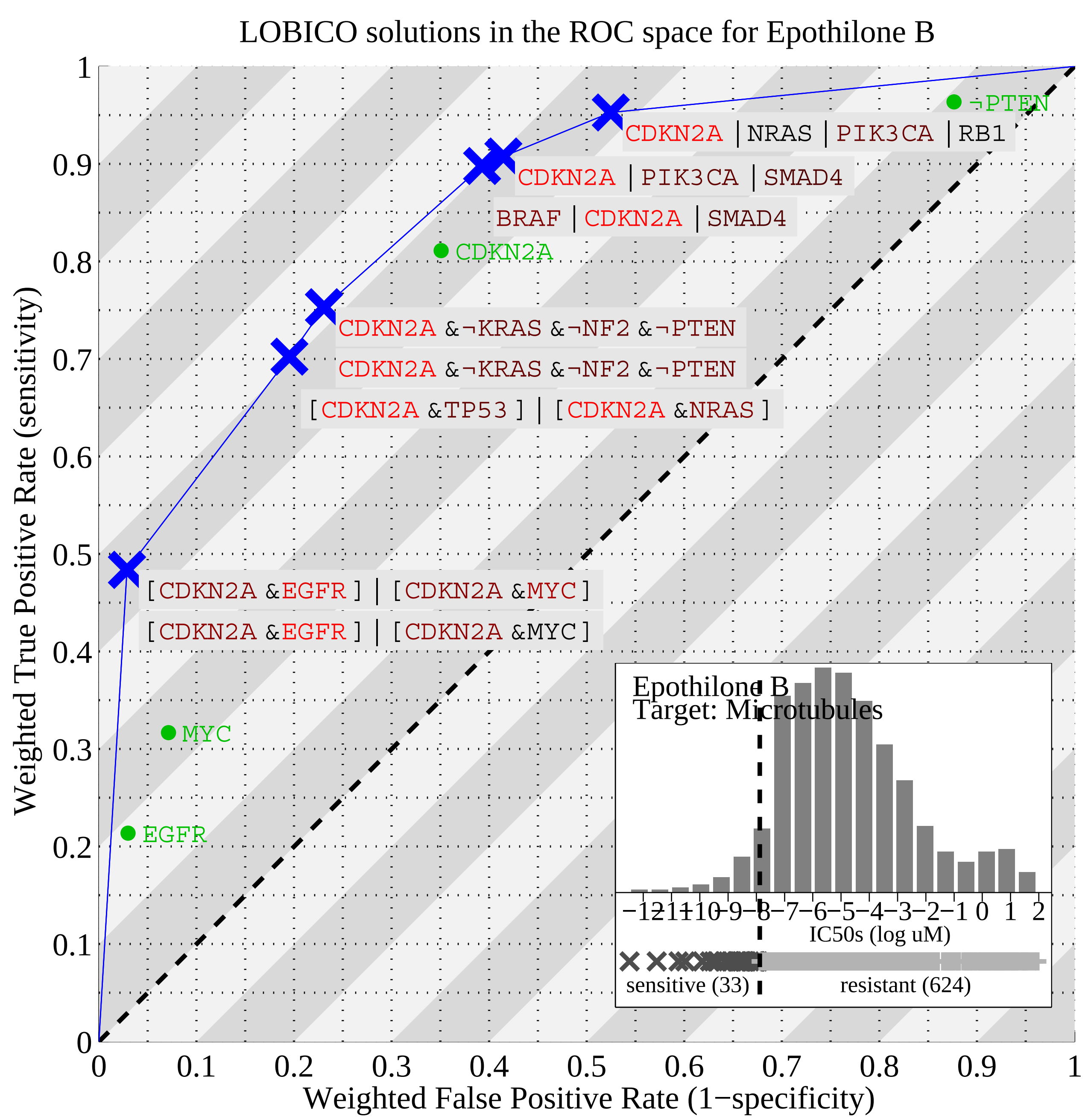

LOBICO solutions in the ROC space for Epothilone B
Weighted True Positive Rate (sensitivity)
Weighted False Positive Rate (1−specificity)
0
0.1
0.2
0.3
0.4
0.5
0.6
0.7
0.8
0.9
1
¬PTEN
CDKN2A | NRAS | PIK3CA | RB1
CDKN2A | PIK3CA | SMAD4
BRAF | CDKN2A | SMAD4
CDKN2A
CDKN2A &¬KRAS &¬NF2 &¬PTEN
CDKN2A &¬KRAS &¬NF2 &¬PTEN
[CDKN2A &TP53] | [CDKN2A &NRAS]
[CDKN2A &EGFR] | [CDKN2A &MYC]
[CDKN2A &EGFR] | [CDKN2A &MYC]
MYC
EGFR
Epothilone B
Target: Microtubules
−12 −11 −10 −9 −8 −7 −6 −5 −4 −3 −2 −1 0 1 2
IC50s (log uM)
sensitive (33)
resistant (624)

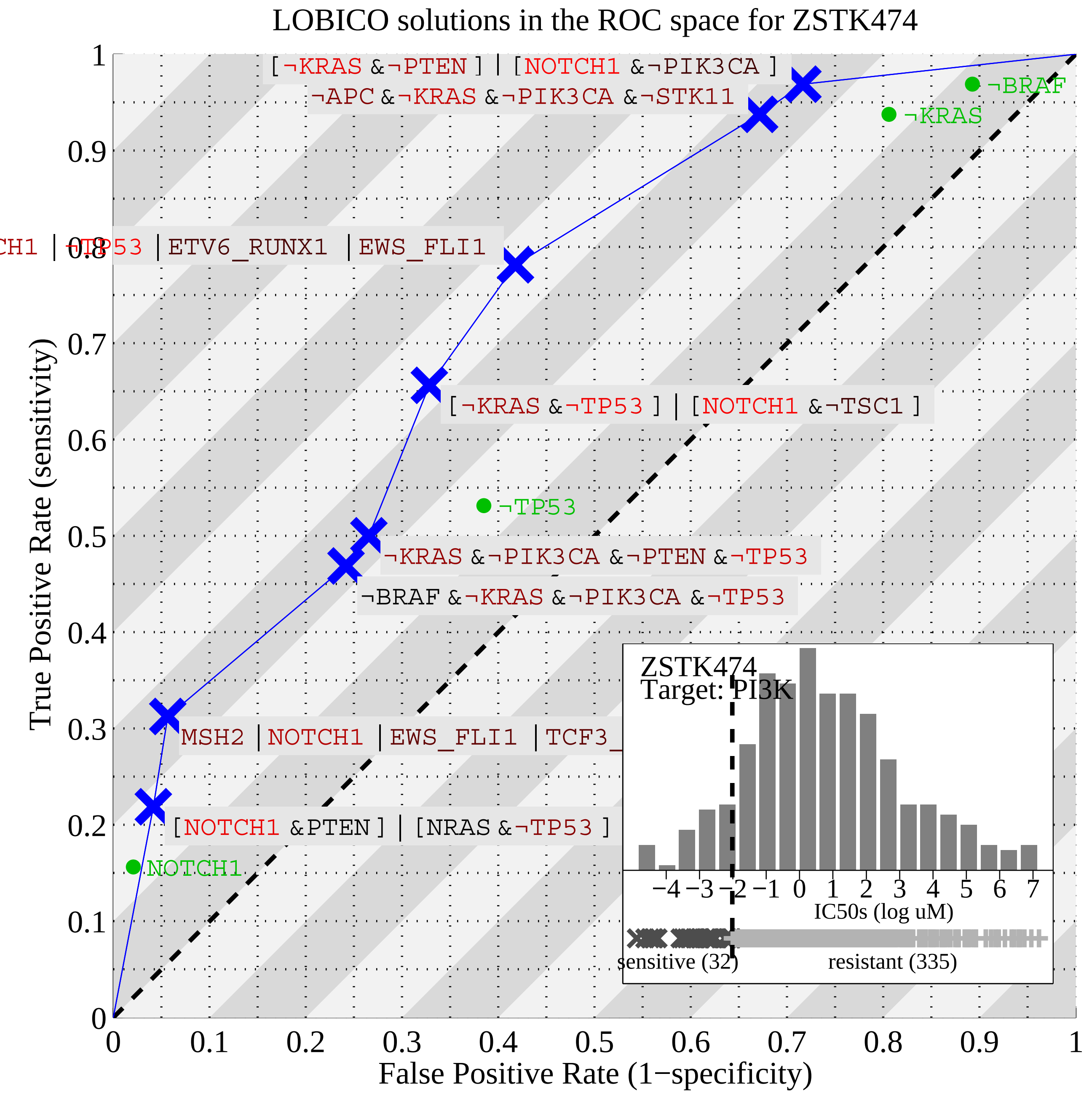

LOBICO solutions in the ROC space for ZSTK474
[¬KRAS &¬PTEN] | [NOTCH1 &¬PIK3CA]
¬APC &¬KRAS &¬PIK3CA &¬STK11
¬BRAF
¬KRAS
CH1 | ¬TP53 | ETV6_RUNX1 | EWS_FLI1
[¬KRAS &¬TP53] | [NOTCH1 &¬TSC1]
¬TP53
¬KRAS &¬PIK3CA &¬PTEN &¬TP53
¬BRAF &¬KRAS &¬PIK3CA &¬TP53
MSH2 | NOTCH1 | EWS_FLI1 | TCF3_
[NOTCH1 &PTEN] | [NRAS &¬TP53]
NOTCH1
ZSTK474
Target: PI3K
IC50s (log uM)
sensitive (32)
resistant (335)
True Positive Rate (sensitivity)
False Positive Rate (1−specificity)

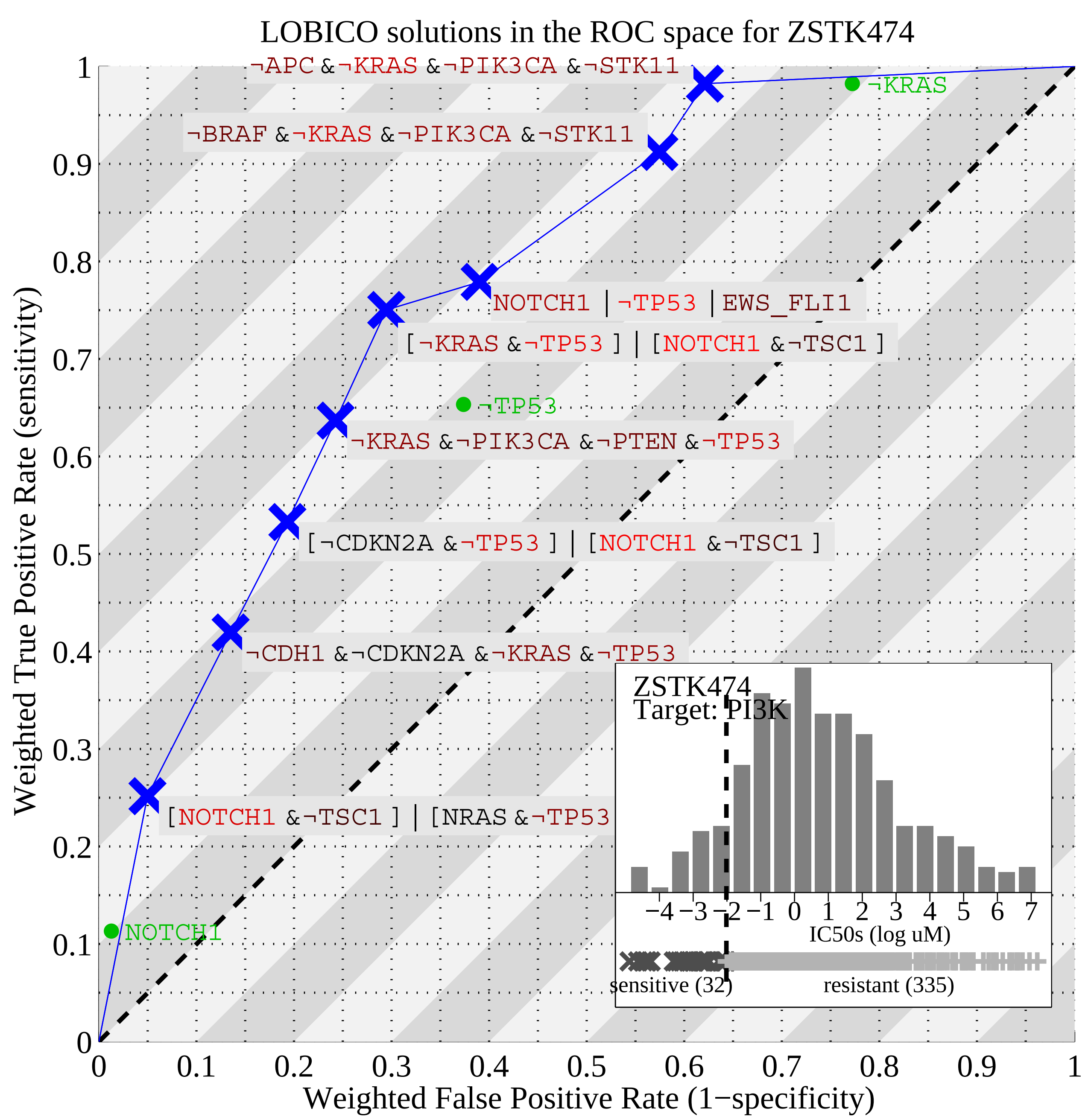

LOBICO solutions in the ROC space for ZSTK474
¬APC &¬KRAS &¬PIK3CA &¬STK11
¬KRAS
¬BRAF &¬KRAS &¬PIK3CA &¬STK11
NOTCH1 |¬TP53 |EWS_FLI1
[¬KRAS &¬TP53] | [NOTCH1 &¬TSC1]
¬TP53
¬KRAS &¬PIK3CA &¬PTEN &¬TP53
[¬CDKN2A &¬TP53] | [NOTCH1 &¬TSC1]
¬CDH1 &¬CDKN2A &¬KRAS &¬TP53
[NOTCH1 &¬TSC1] | [NRAS &¬TP53
NOTCH1
ZSTK474
Target: PI3K
IC50s (log uM)
sensitive (32)
resistant (335)
Weighted True Positive Rate (sensitivity)
Weighted False Positive Rate (1−specificity)

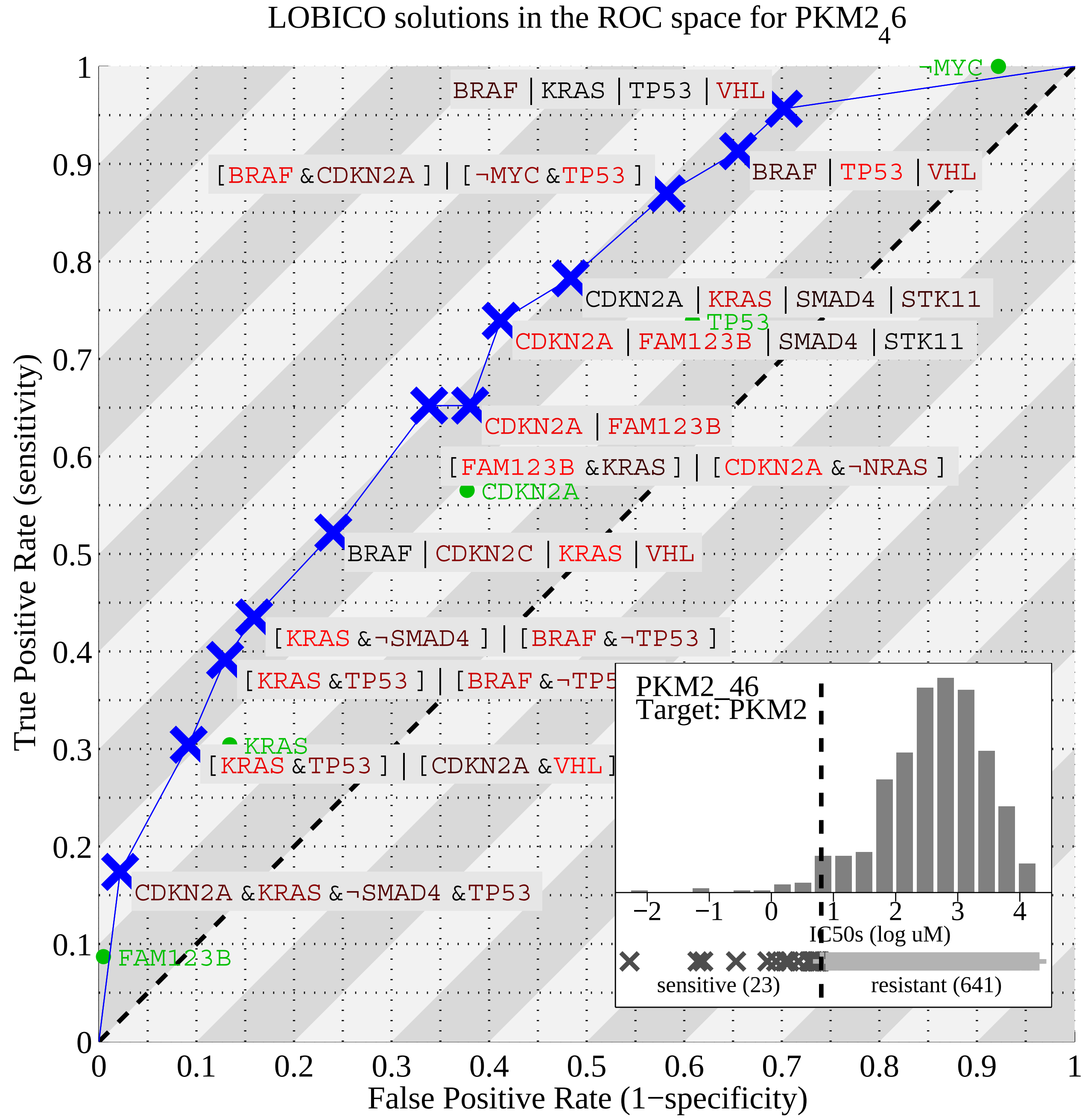

LOBICO solutions in the ROC space for PKM2_46
True Positive Rate (sensitivity)
False Positive Rate (1−specificity)
0
0.1
0.2
0.3
0.4
0.5
0.6
0.7
0.8
0.9
1
¬MYC
BRAF | KRAS | TP53 | VHL
BRAF | TP53 | VHL
[BRAF & CDKN2A] | [¬MYC & TP53]
CDKN2A | KRAS | SMAD4 | STK11
TP53
CDKN2A | FAM123B | SMAD4 | STK11
CDKN2A | FAM123B
[FAM123B & KRAS] | [CDKN2A & ¬NRAS]
CDKN2A
BRAF | CDKN2C | KRAS | VHL
[KRAS & ¬SMAD4] | [BRAF & ¬TP53]
[KRAS & TP53] | [BRAF & ¬TP5
KRAS
[KRAS & TP53] | [CDKN2A & VHL]
CDKN2A & KRAS & ¬SMAD4 & TP53
FAM123B
PKM2_46
Target: PKM2
−2
−1
0
1
2
3
4
IC50s (log uM)
sensitive (23)
resistant (641)

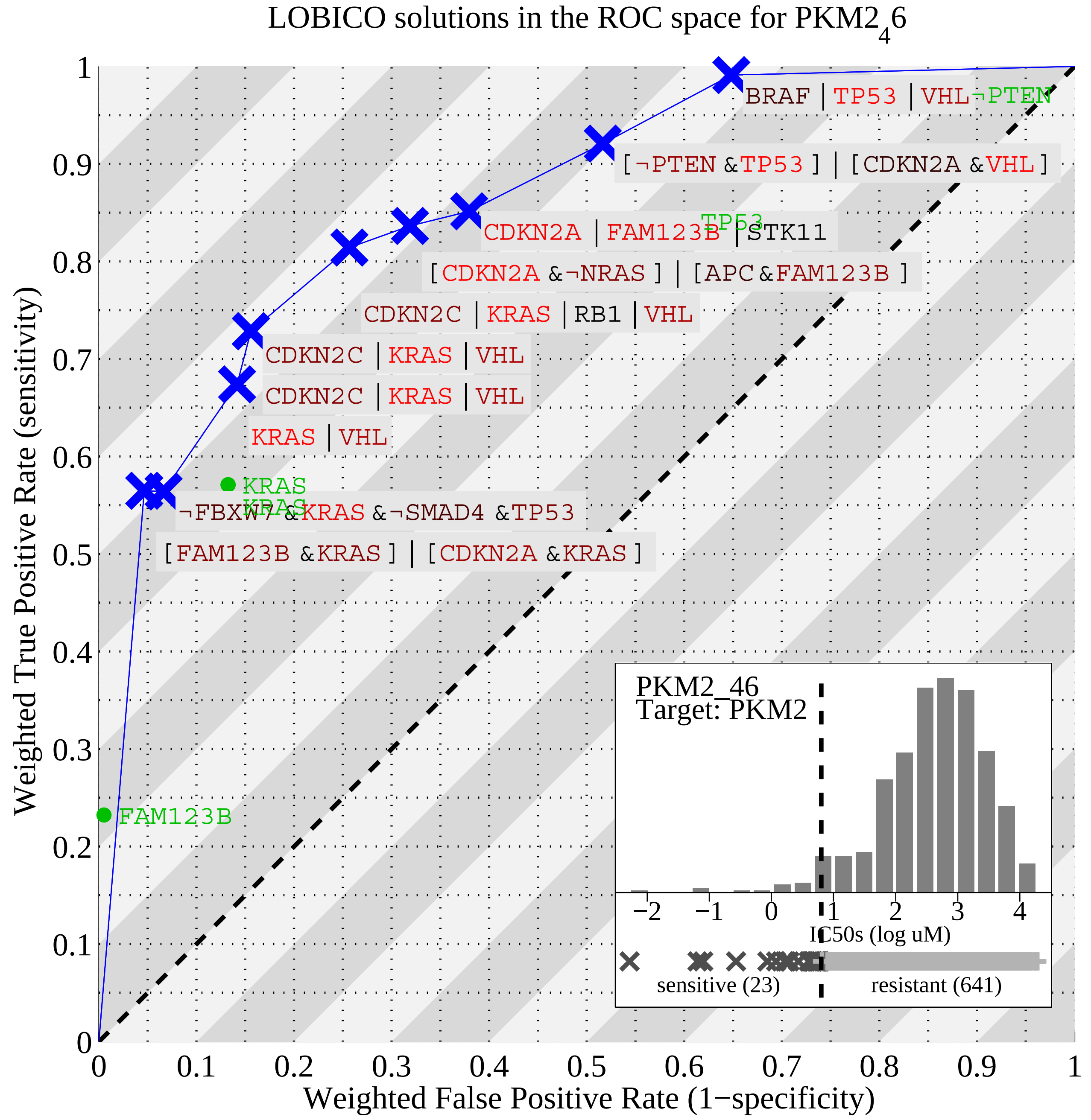
LOBICO solutions in the ROC space for PKM2_46
Weighted True Positive Rate (sensitivity)
Weighted False Positive Rate (1−specificity)
0
0.1
0.2
0.3
0.4
0.5
0.6
0.7
0.8
0.9
1
BRAF | TP53 | VHL ¬PTEN
[¬PTEN & TP53] | [CDKN2A & VHL]
CDKN2A | FAM123B | STK11
TP53
[CDKN2A & ¬NRAS] | [APC & FAM123B]
CDKN2C | KRAS | RB1 | VHL
CDKN2C | KRAS | VHL
CDKN2C | KRAS | VHL
KRAS | VHL
KRAS
¬FBXW7 & KRAS & ¬SMAD4 & TP53
[FAM123B & KRAS] | [CDKN2A & KRAS]
FAM123B
PKM2_46
Target: PKM2
−2
−1
0
1
2
3
4
IC50s (log uM)
sensitive (23)
resistant (641)

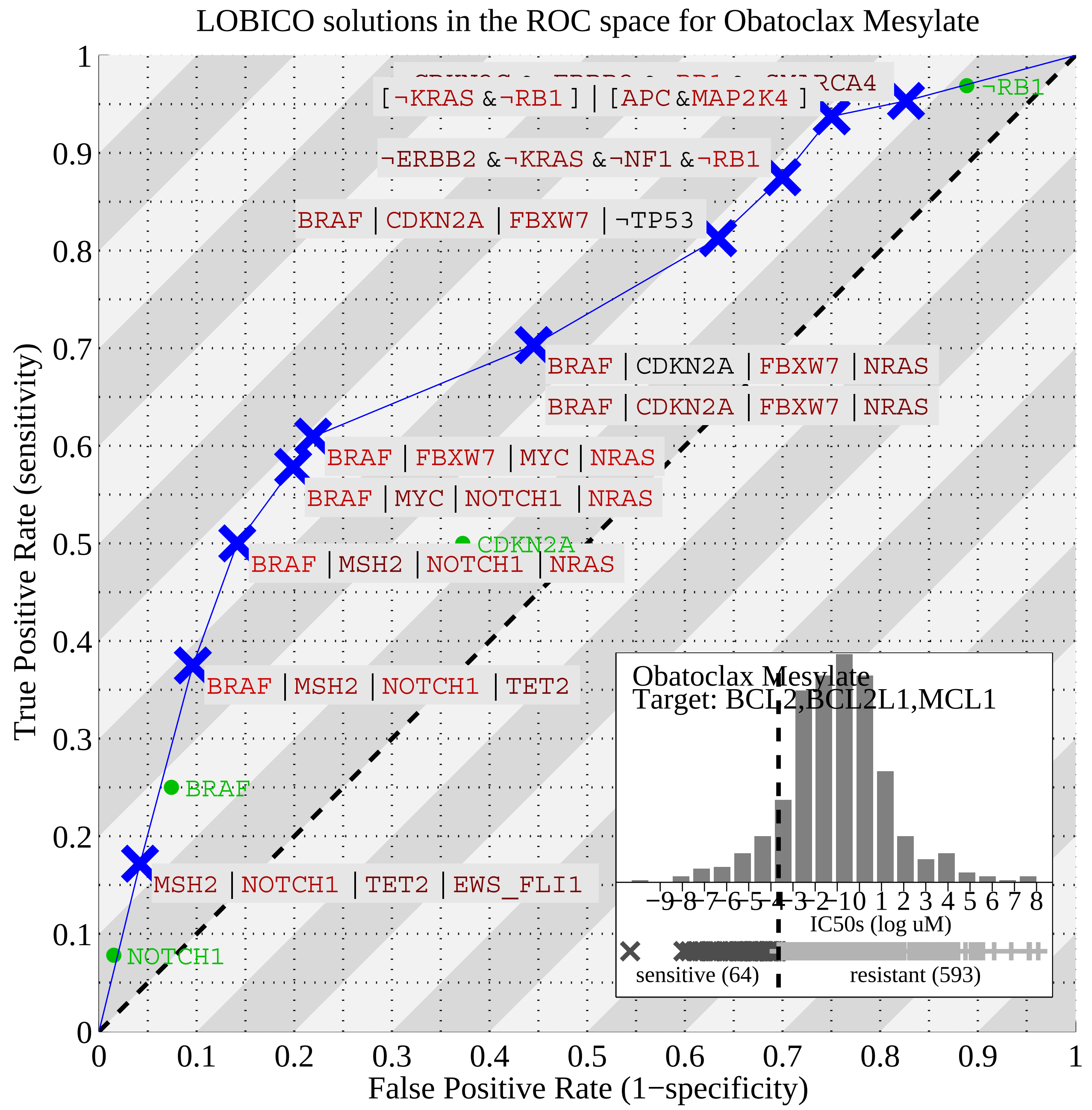

LOBICO solutions in the ROC space for Obatoclax Mesylate
[¬KRAS &¬RB1] | [APC &MAP2K4]
¬RB1
¬ERBB2 &¬KRAS &¬NF1 &¬RB1
BRAF | CDKN2A | FBXW7 | ¬TP53
BRAF | CDKN2A | FBXW7 | NRAS
BRAF | CDKN2A | FBXW7 | NRAS
BRAF | FBXW7 | MYC | NRAS
BRAF | MYC | NOTCH1 | NRAS
CDKN2A
BRAF | MSH2 | NOTCH1 | NRAS
BRAF | MSH2 | NOTCH1 | TET2
BRAF
MSH2 | NOTCH1 | TET2 | EWS_FLI1
NOTCH1
True Positive Rate (sensitivity)
False Positive Rate (1−specificity)
0
0.1
0.2
0.3
0.4
0.5
0.6
0.7
0.8
0.9
1
Obatoclax Mesylate
Target: BCL2,BCL2L1,MCL1
−9 −8 −7 −6 −5 −4 −3 −2 −1 0 1 2 3 4 5 6 7 8
IC50s (log uM)
sensitive (64)
resistant (593)

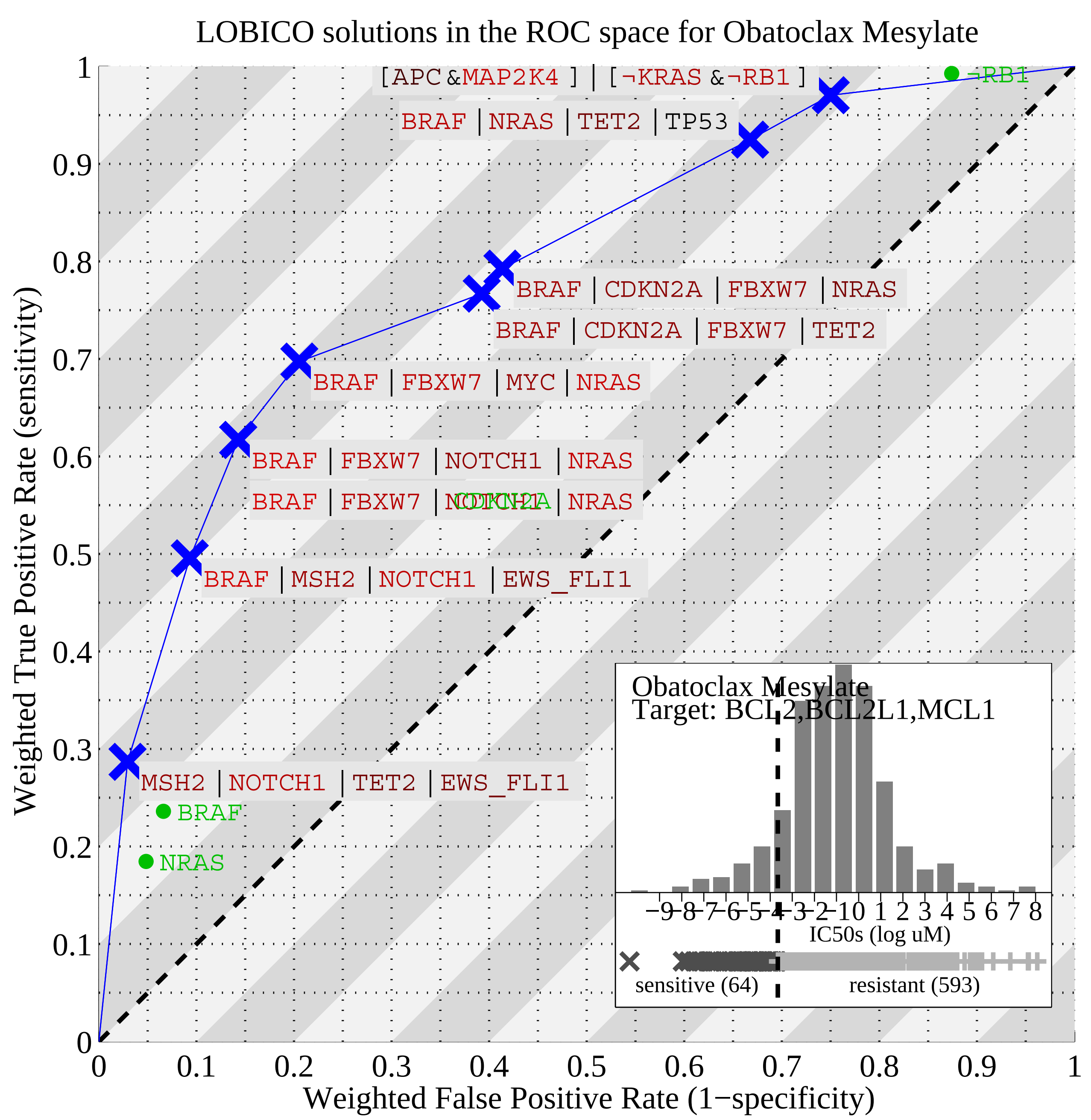

LOBICO solutions in the ROC space for Obatoclax Mesylate
[APC & MAP2K4] | [¬KRAS & ¬RB1]
¬RB1
BRAF | NRAS | TET2 | TP53
BRAF | CDKN2A | FBXW7 | NRAS
BRAF | CDKN2A | FBXW7 | TET2
BRAF | FBXW7 | MYC | NRAS
BRAF | FBXW7 | NOTCH1 | NRAS
BRAF | FBXW7 | NOTCH1 | NRAS
CDKN2A
BRAF | MSH2 | NOTCH1 | EWS_FLI1
MSH2 | NOTCH1 | TET2 | EWS_FLI1
BRAF
NRAS
Obatoclax Mesylate
Target: BCL2,BCL2L1,MCL1
−9 −8 −7 −6 −5 −4 −3 −2 −1 0 1 2 3 4 5 6 7 8
IC50s (log uM)
sensitive (64)
resistant (593)
Weighted True Positive Rate (sensitivity)
Weighted False Positive Rate (1−specificity)
0 0.1 0.2 0.3 0.4 0.5 0.6 0.7 0.8 0.9 1

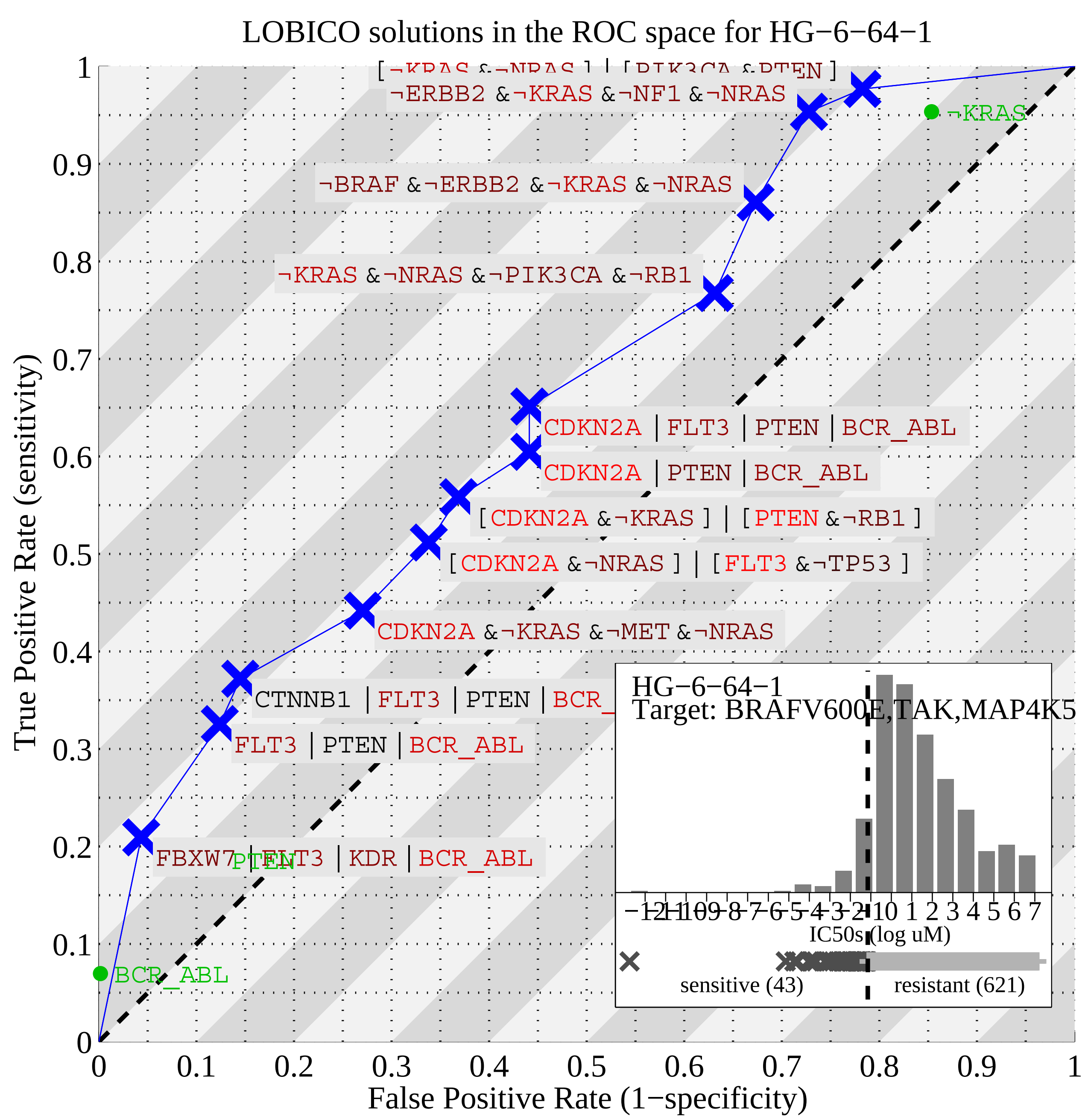
LOBICO solutions in the ROC space for HG−6−64−1
True Positive Rate (sensitivity)
False Positive Rate (1−specificity)
0
0.1
0.2
0.3
0.4
0.5
0.6
0.7
0.8
0.9
1
¬ERBB2 &¬KRAS &¬NF1 &¬NRAS
¬KRAS
¬BRAF &¬ERBB2 &¬KRAS &¬NRAS
¬KRAS &¬NRAS &¬PIK3CA &¬RB1
CDKN2A | FLT3 | PTEN | BCR_ABL
CDKN2A | PTEN | BCR_ABL
[CDKN2A &¬KRAS] | [PTEN &¬RB1]
[CDKN2A &¬NRAS] | [FLT3 &¬TP53]
CDKN2A &¬KRAS &¬MET &¬NRAS
FLT3 | PTEN | BCR_ABL
PTEN
BCR_ABL
HG−6−64−1
Target: BRAFV600E,TAK,MAP4K5
IC50s (log uM)
sensitive (43)
resistant (621)

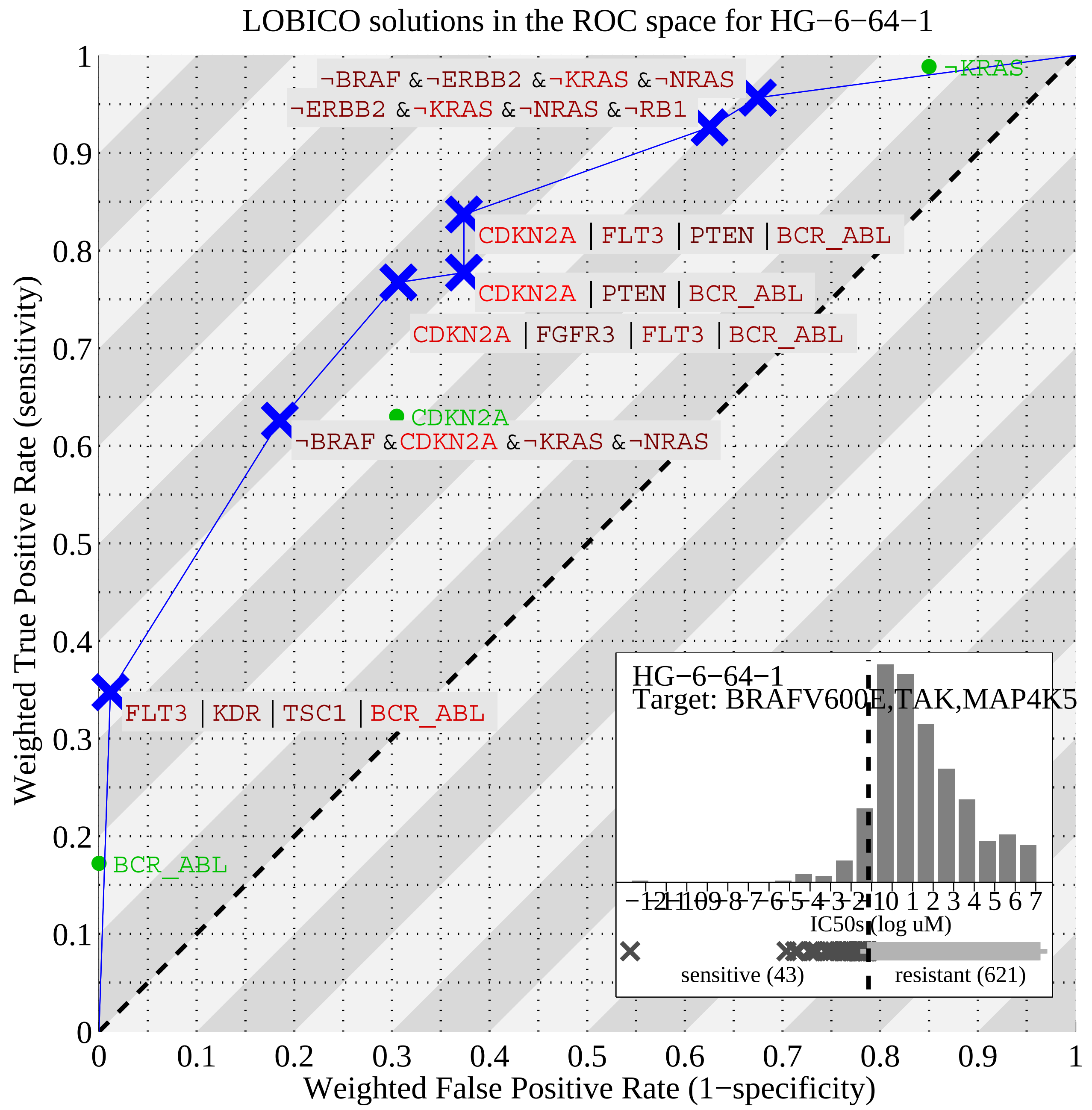
LOBICO solutions in the ROC space for HG−6−64−1
¬BRAF &¬ERBB2 &¬KRAS &¬NRAS
¬ERBB2 &¬KRAS &¬NRAS &¬RB1
¬KRAS
CDKN2A | FLT3 | PTEN | BCR_ABL
CDKN2A | PTEN | BCR_ABL
CDKN2A | FGFR3 | FLT3 | BCR_ABL
CDKN2A
¬BRAF &CDKN2A &¬KRAS &¬NRAS
FLT3 | KDR | TSC1 | BCR_ABL
BCR_ABL
HG−6−64−1
Target: BRAFV600E,TAK,MAP4K5
IC50s (log uM)
sensitive (43)
resistant (621)
Weighted True Positive Rate (sensitivity)
Weighted False Positive Rate (1−specificity)

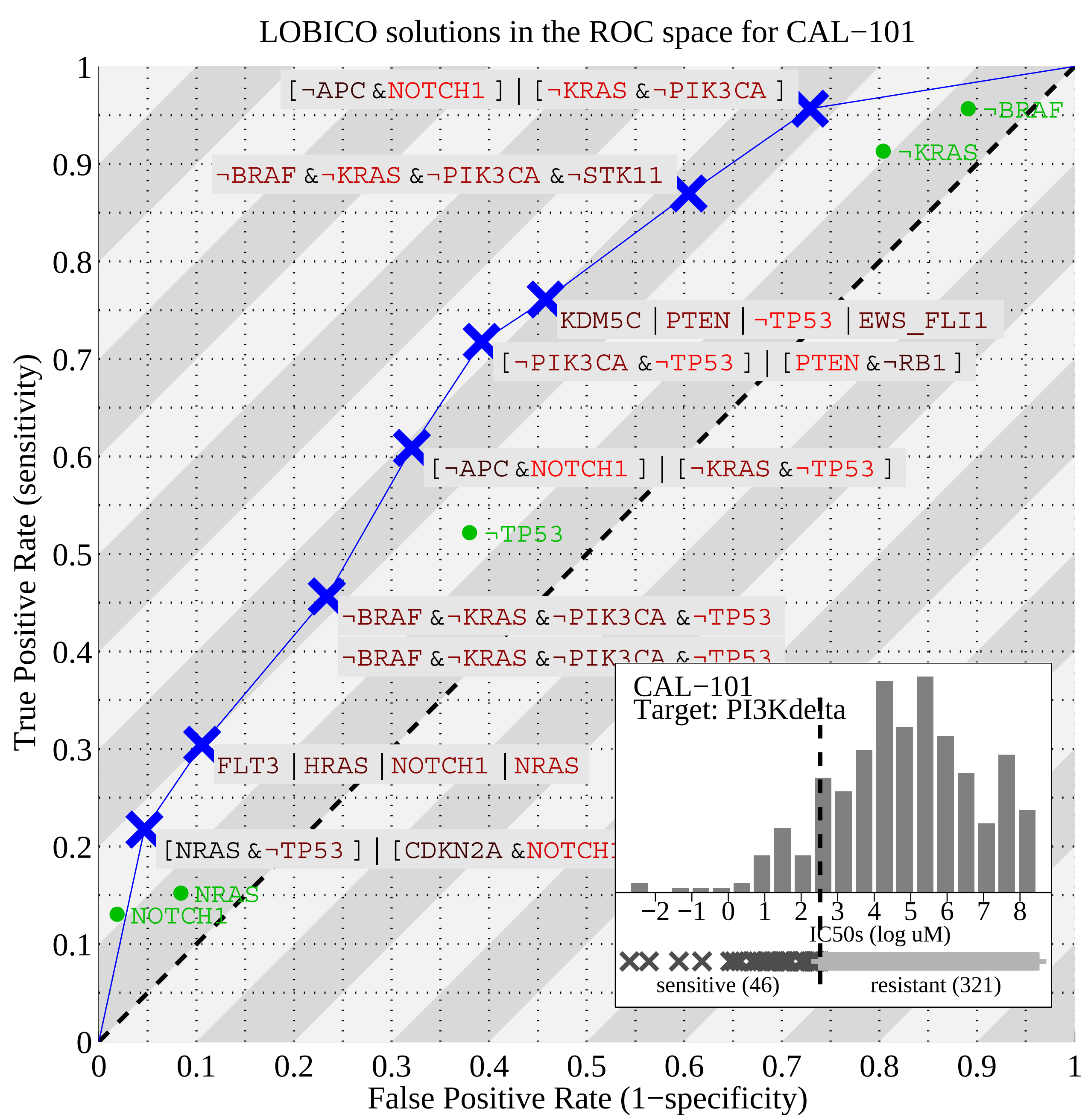

LOBICO solutions in the ROC space for CAL−101
True Positive Rate (sensitivity)
False Positive Rate (1−specificity)
[¬APC &NOTCH1] | [¬KRAS &¬PIK3CA]
¬BRAF &¬KRAS &¬PIK3CA &¬STK11
KDM5C | PTEN | ¬TP53 | EWS_FLI1
[¬PIK3CA &¬TP53] | [PTEN &¬RB1]
[¬APC &NOTCH1] | [¬KRAS &¬TP53]
¬BRAF &¬KRAS &¬PIK3CA &¬TP53
FLT3 | HRAS | NOTCH1 | NRAS
¬BRAF
¬KRAS
¬TP53
NRAS
NOTCH1
CAL−101
Target: PI3Kdelta
IC50s (log uM)
sensitive (46)
resistant (321)

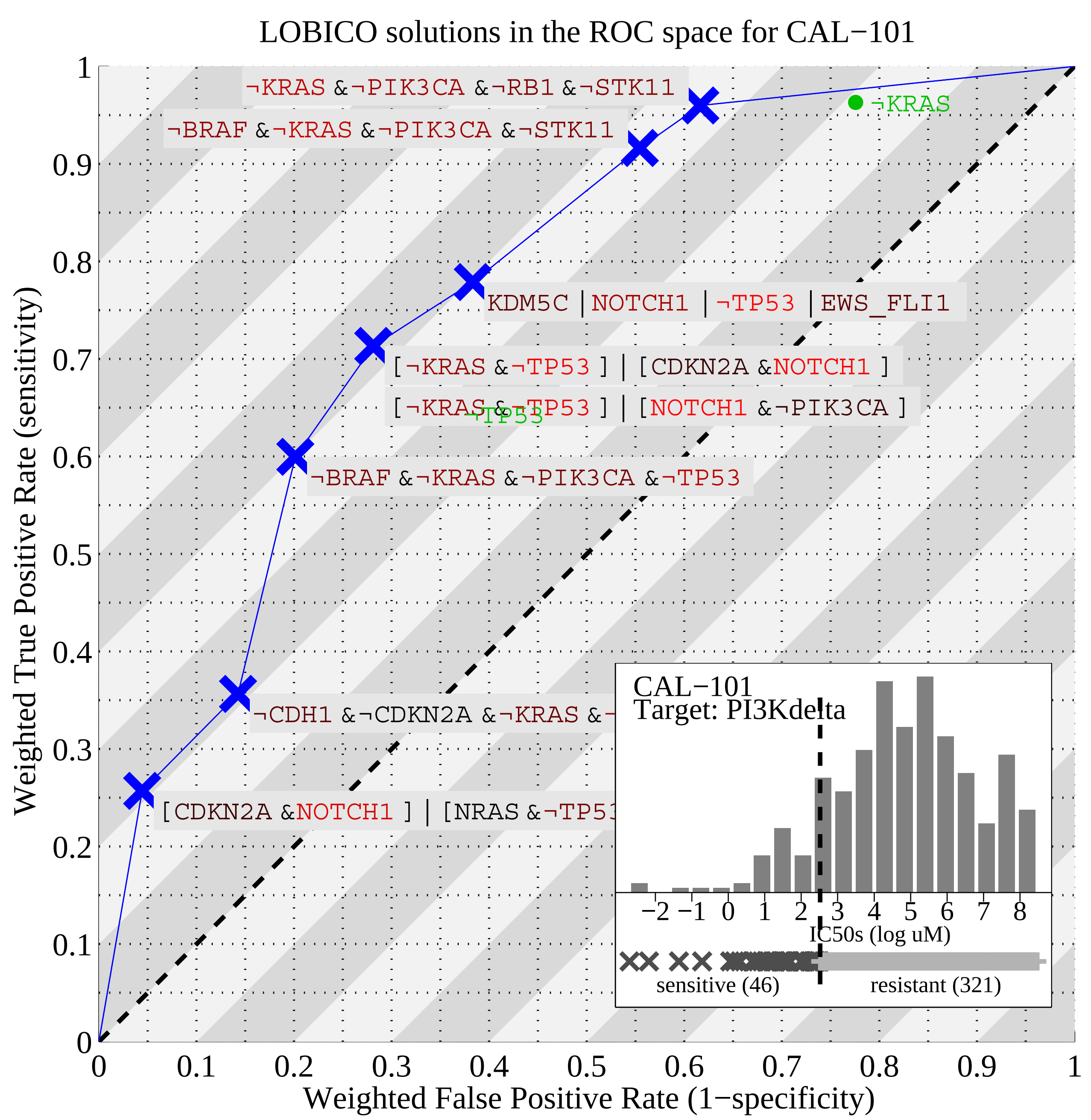

LOBICO solutions in the ROC space for CAL−101
Weighted True Positive Rate (sensitivity)
Weighted False Positive Rate (1−specificity)
¬KRAS &¬PIK3CA &¬RB1 &¬STK11
¬BRAF &¬KRAS &¬PIK3CA &¬STK11
¬KRAS
KDM5C | NOTCH1 | ¬TP53 | EWS_FLI1
[¬KRAS &¬TP53] | [CDKN2A &NOTCH1]
[¬KRAS &¬TP53] | [NOTCH1 &¬PIK3CA]
¬TP53
¬BRAF &¬KRAS &¬PIK3CA &¬TP53
¬CDH1 &¬CDKN2A &¬KRAS &
[CDKN2A &NOTCH1] | [NRAS &¬TP53
CAL−101
Target: PI3Kdelta
IC50s (log uM)
sensitive (46)
resistant (321)

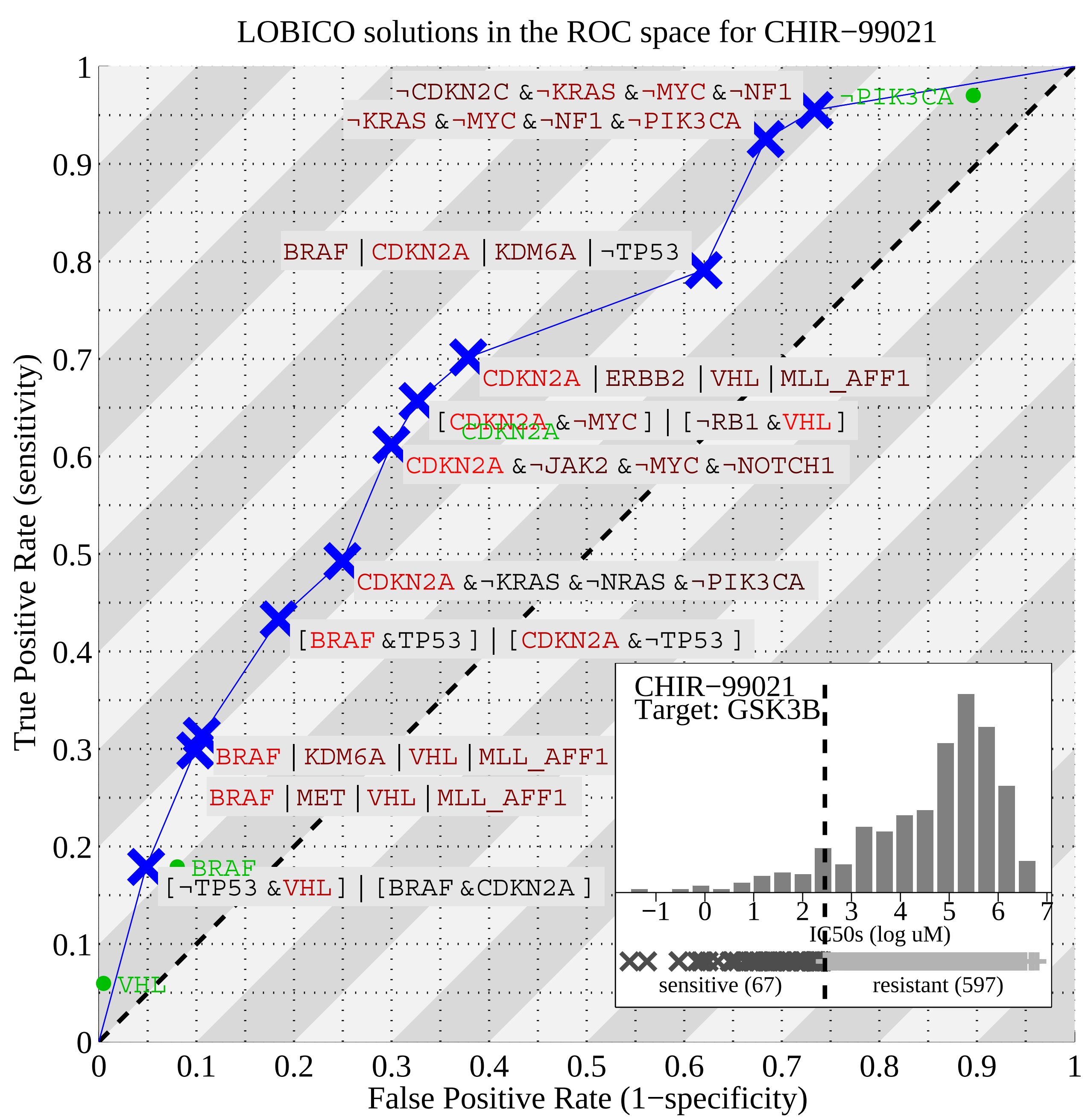

LOBICO solutions in the ROC space for CHIR−99021
True Positive Rate (sensitivity)
False Positive Rate (1−specificity)
¬CDKN2C &¬KRAS &¬MYC &¬NF1
¬KRAS &¬MYC &¬NF1 &¬PIK3CA
¬PIK3CA
BRAF | CDKN2A | KDM6A | ¬TP53
CDKN2A | ERBB2 | VHL | MLL_AFF1
[CDKN2A &¬MYC] | [¬RB1 &VHL]
CDKN2A
CDKN2A &¬JAK2 &¬MYC &¬NOTCH1
CDKN2A &¬KRAS &¬NRAS &¬PIK3CA
[BRAF &TP53] | [CDKN2A &¬TP53]
BRAF | KDM6A | VHL | MLL_AFF1
BRAF | MET | VHL | MLL_AFF1
BRAF
[¬TP53 &VHL] | [BRAF &CDKN2A]
VHL
CHIR−99021
Target: GSK3B
IC50s (log uM)
sensitive (67)
resistant (597)

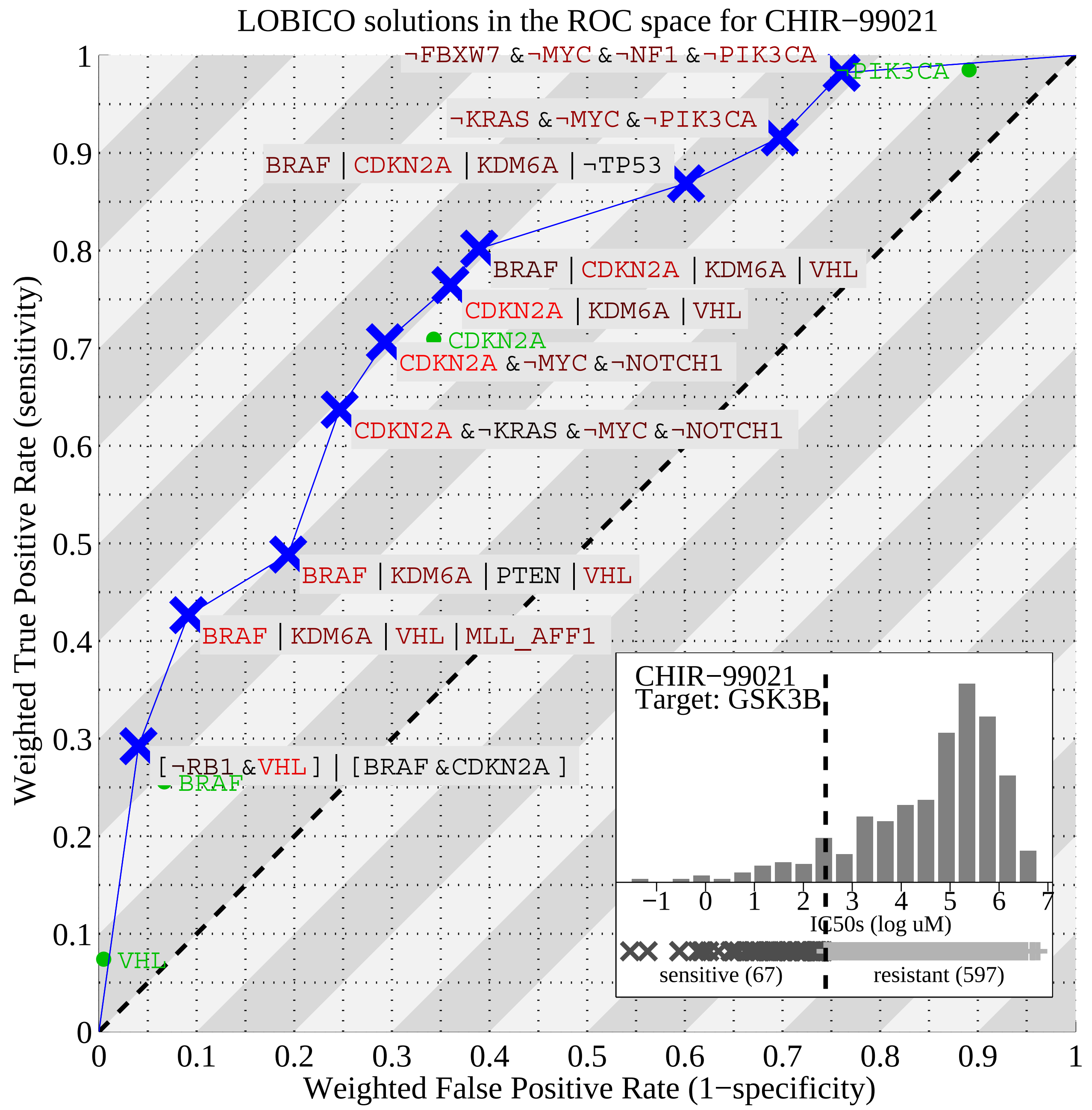

LOBICO solutions in the ROC space for CHIR−99021
¬FBXW7 &¬MYC &¬NF1 &¬PIK3CA
¬PIK3CA
¬KRAS &¬MYC &¬PIK3CA
BRAF | CDKN2A | KDM6A | ¬TP53
BRAF | CDKN2A | KDM6A | VHL
CDKN2A | KDM6A | VHL
CDKN2A
CDKN2A &¬MYC &¬NOTCH1
CDKN2A &¬KRAS &¬MYC &¬NOTCH1
BRAF | KDM6A | PTEN | VHL
BRAF | KDM6A | VHL | MLL_AFF1
[¬RB1 &VHL] | [BRAF &CDKN2A]
BRAF
VHL
CHIR−99021
Target: GSK3B
IC50s (log uM)
sensitive (67)
resistant (597)
Weighted True Positive Rate (sensitivity)
Weighted False Positive Rate (1−specificity)

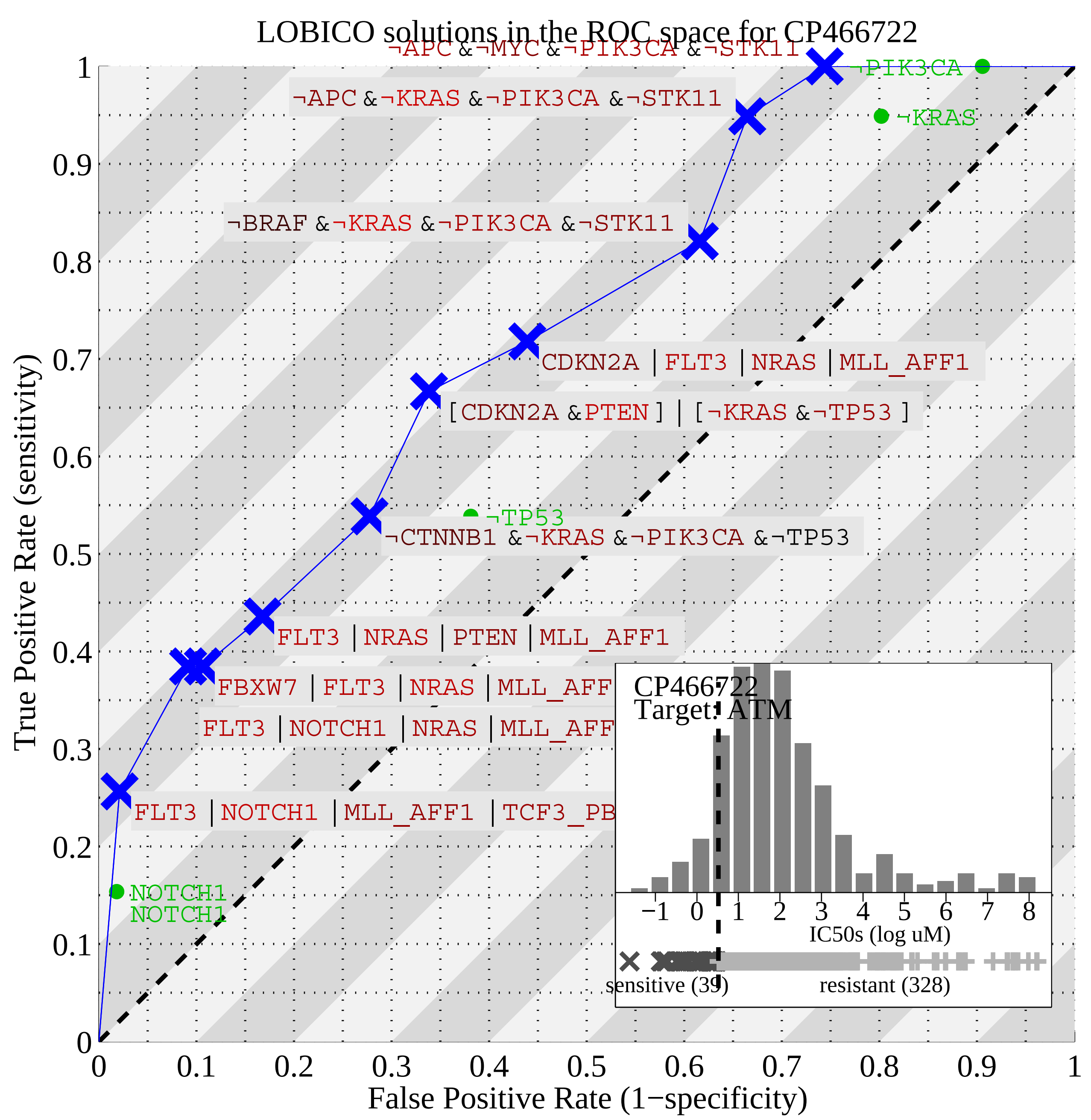

LOBICO solutions in the ROC space for CP466722
¬APC &¬MYC &¬PIK3CA &¬STK11
¬PIK3CA
¬APC &¬KRAS &¬PIK3CA &¬STK11
¬KRAS
¬BRAF &¬KRAS &¬PIK3CA &¬STK11
CDKN2A | FLT3 | NRAS | MLL_AFF1
[CDKN2A & PTEN] | [¬KRAS &¬TP53]
¬TP53
¬CTNNB1 &¬KRAS &¬PIK3CA &¬TP53
FLT3 | NRAS | PTEN | MLL_AFF1
FBXW7 | FLT3 | NRAS | MLL_AFF
FLT3 | NOTCH1 | NRAS | MLL_AFF
FLT3 | NOTCH1 | MLL_AFF1 | TCF3_PB
NOTCH1
NOTCH1
CP466722
Target: ATM
IC50s (log uM)
sensitive (39)
resistant (328)
True Positive Rate (sensitivity)
False Positive Rate (1−specificity)

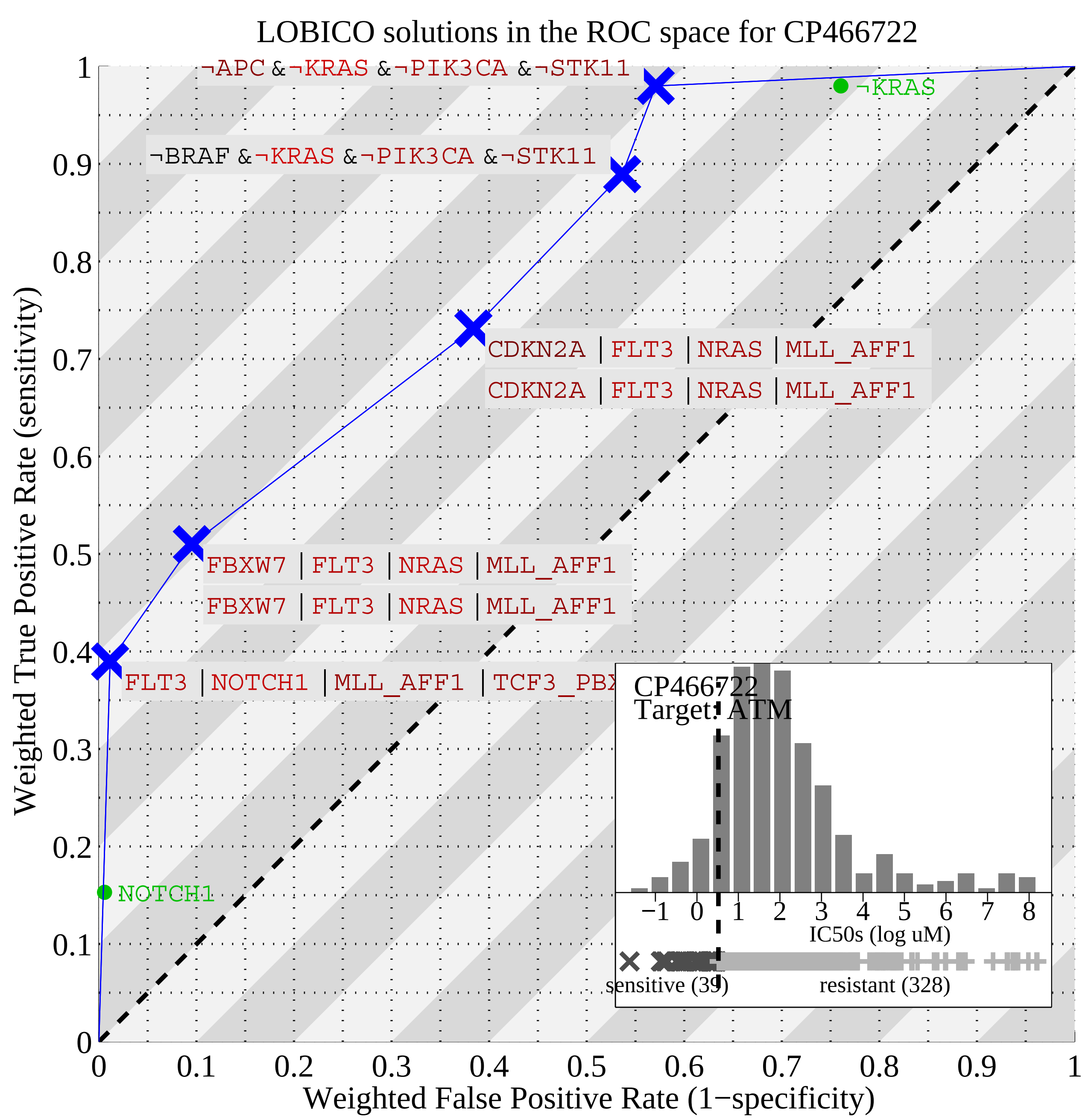

LOBICO solutions in the ROC space for CP466722
¬APC &¬KRAS &¬PIK3CA &¬STK11
¬BRAF &¬KRAS &¬PIK3CA &¬STK11
¬KRAS
CDKN2A | FLT3 | NRAS | MLL_AFF1
CDKN2A | FLT3 | NRAS | MLL_AFF1
FBXW7 | FLT3 | NRAS | MLL_AFF1
FBXW7 | FLT3 | NRAS | MLL_AFF1
FLT3 | NOTCH1 | MLL_AFF1 | TCF3_PBX
NOTCH1
CP466722
Target: ATM
IC50s (log uM)
sensitive (39)
resistant (328)
Weighted True Positive Rate (sensitivity)
Weighted False Positive Rate (1−specificity)

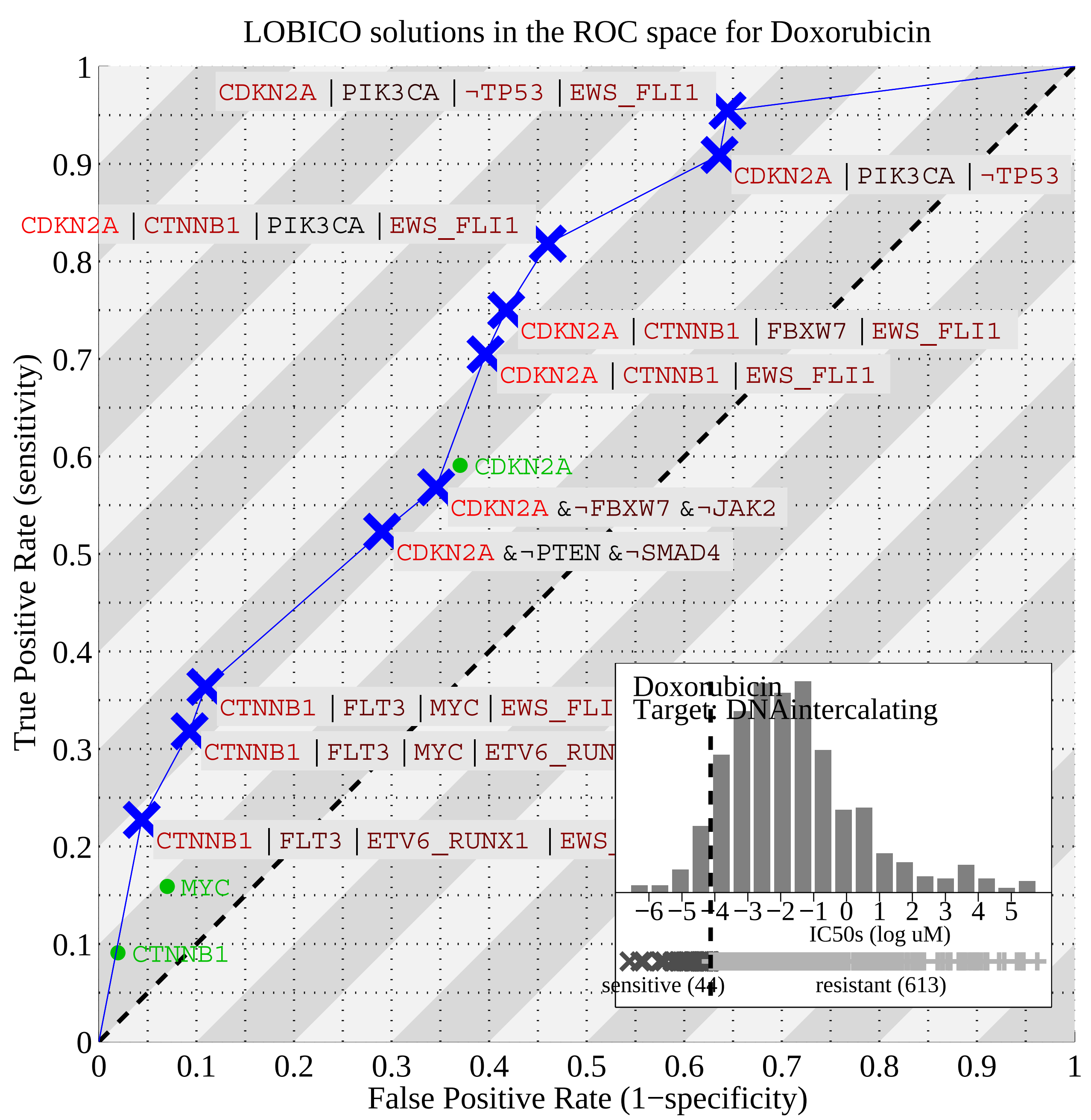
LOBICO solutions in the ROC space for Doxorubicin
CDKN2A |PIK3CA |¬TP53 |EWS_FLI1
CDKN2A |PIK3CA |¬TP53
CDKN2A |CTNNB1 |PIK3CA |EWS_FLI1
CDKN2A |CTNNB1 |FBXW7 |EWS_FLI1
CDKN2A |CTNNB1 |EWS_FLI1
CDKN2A
CDKN2A &¬FBXW7 &¬JAK2
CDKN2A &¬PTEN &¬SMAD4
CTNNB1 |FLT3 |MYC |EWS_FLI
CTNNB1 |FLT3 |MYC |ETV6_RUN
CTNNB1 |FLT3 |ETV6_RUNX1 |EWS_
MYC
CTNNB1
Doxorubicin
Target: DNAintercalating
IC50s (log uM)
sensitive (44)
resistant (613)
True Positive Rate (sensitivity)
False Positive Rate (1−specificity)

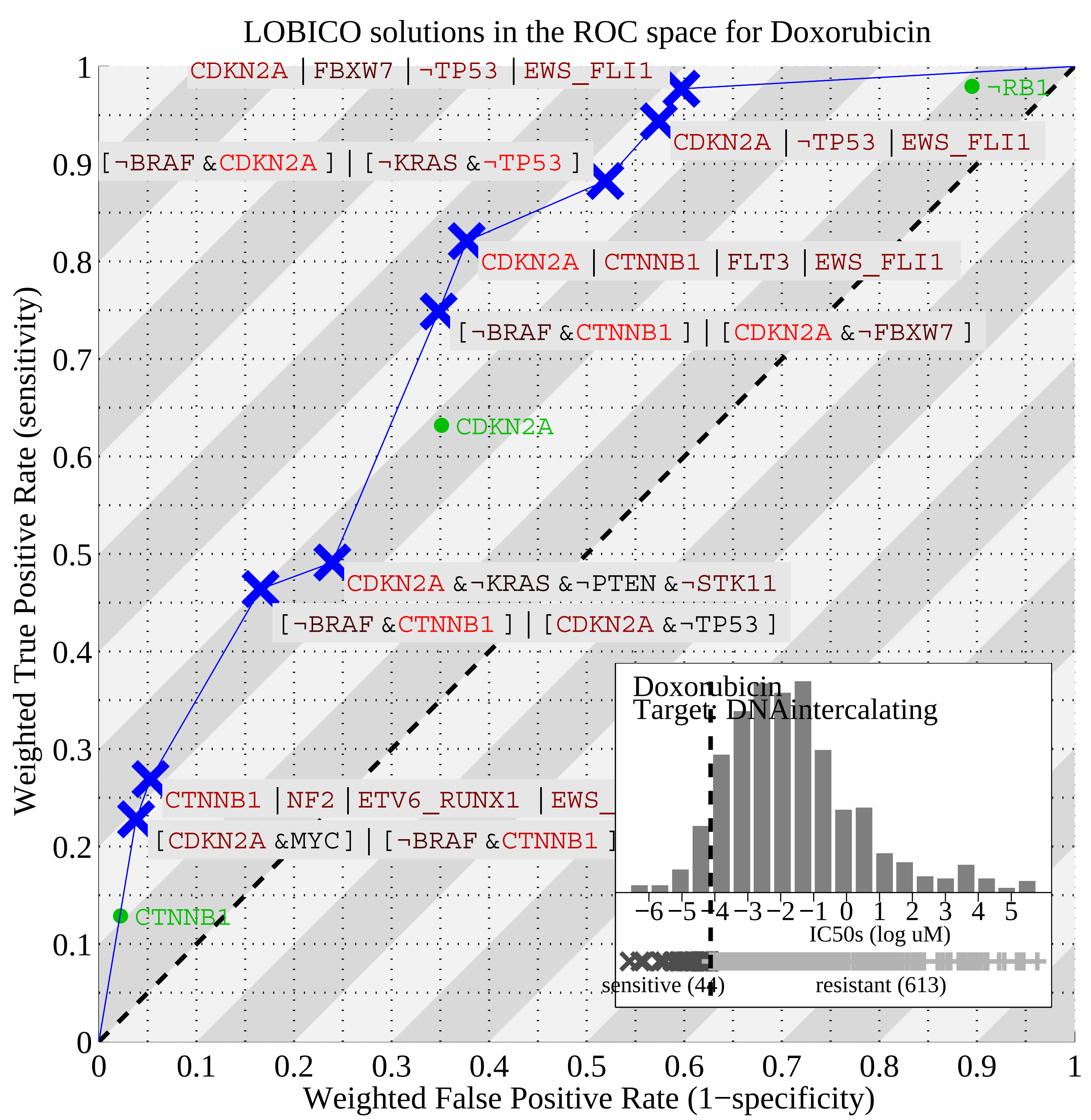

LOBICO solutions in the ROC space for Doxorubicin
CDKN2A |FBXW7 |¬TP53 |EWS_FLI1
¬RB1
CDKN2A |¬TP53 |EWS_FLI1
[¬BRAF &CDKN2A] | [¬KRAS &¬TP53]
CDKN2A |CTNNB1 |FLT3 |EWS_FLI1
[¬BRAF &CTNNB1] | [CDKN2A &¬FBXW7]
CDKN2A
CDKN2A &¬KRAS &¬PTEN &¬STK11
[¬BRAF &CTNNB1] | [CDKN2A &¬TP53]
CTNNB1 |NF2 |ETV6_RUNX1 |EWS_
[CDKN2A &MYC] | [¬BRAF &CTNNB1]
CTNNB1
Doxorubicin
Target: DNAintercalating
IC50s (log uM)
sensitive (44)
resistant (613)
Weighted True Positive Rate (sensitivity)
Weighted False Positive Rate (1−specificity)

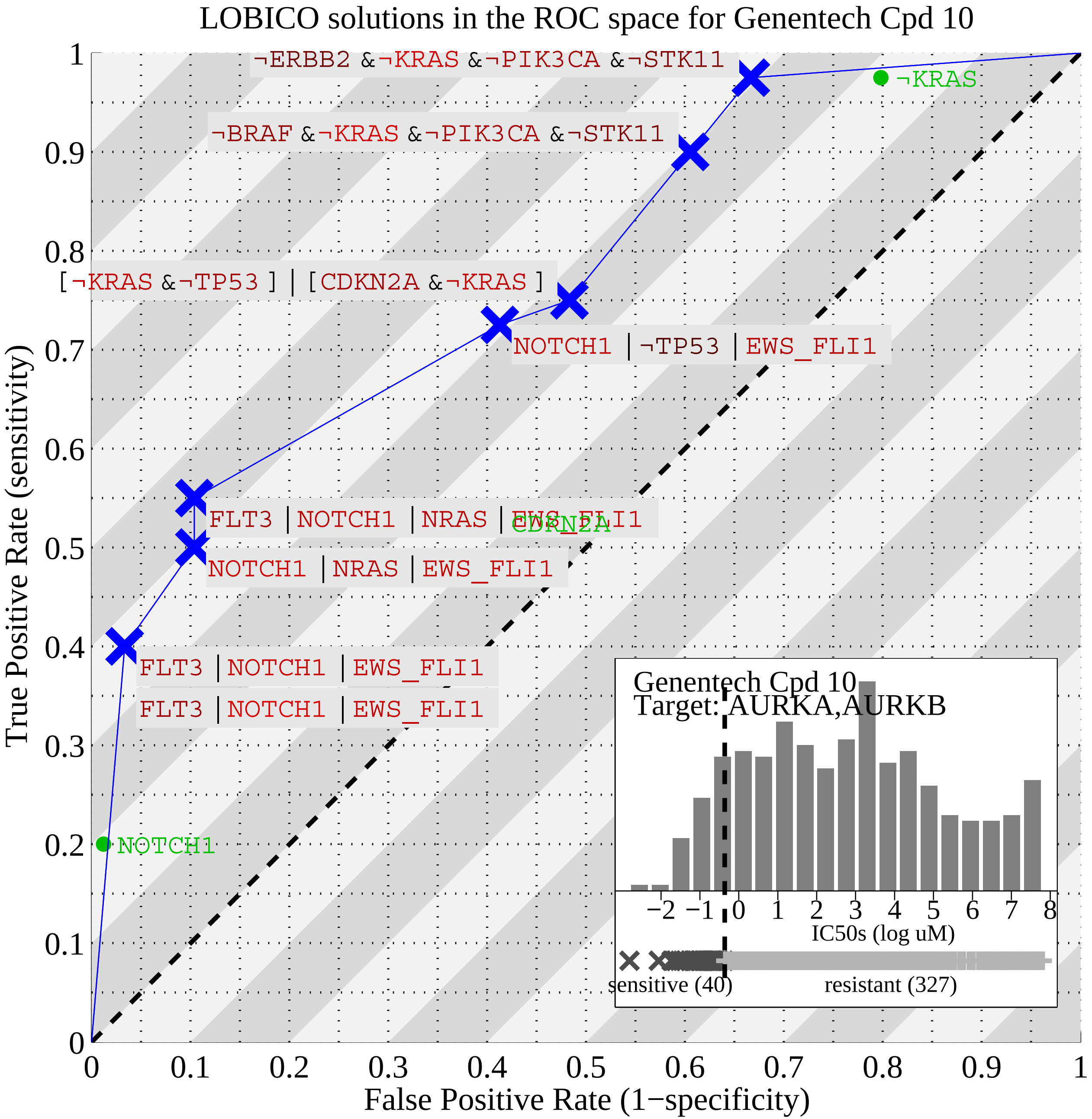

LOBICO solutions in the ROC space for Genentech Cpd 10
¬ERBB2 & ¬KRAS & ¬PIK3CA & ¬STK11
¬KRAS
¬BRAF & ¬KRAS & ¬PIK3CA & ¬STK11
[¬KRAS & ¬TP53] | [CDKN2A & ¬KRAS]
NOTCH1 | ¬TP53 | EWS_FLI1
FLT3 | NOTCH1 | NRAS | EWS_FLI1
CDKN2A
NOTCH1 | NRAS | EWS_FLI1
FLT3 | NOTCH1 | EWS_FLI1
FLT3 | NOTCH1 | EWS_FLI1
NOTCH1
Genentech Cpd 10
Target: AURKA,AURKB
IC50s (log uM)
sensitive (40)
resistant (327)
True Positive Rate (sensitivity)
False Positive Rate (1−specificity)

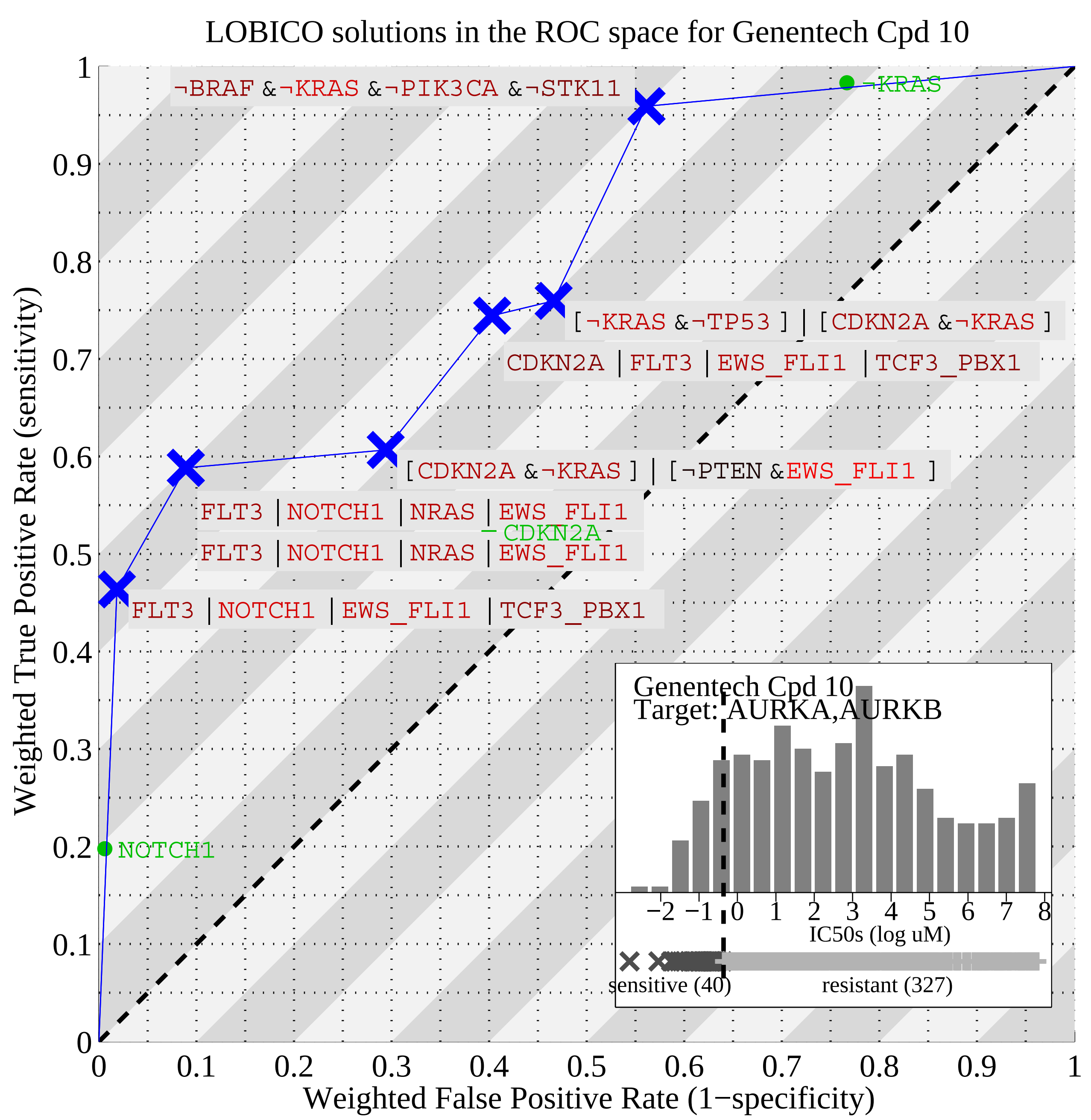
LOBICO solutions in the ROC space for Genentech Cpd 10
Weighted True Positive Rate (sensitivity)
Weighted False Positive Rate (1−specificity)
¬BRAF &¬KRAS &¬PIK3CA &¬STK11
¬KRAS
[¬KRAS &¬TP53] | [CDKN2A &¬KRAS]
CDKN2A | FLT3 | EWS_FLI1 | TCF3_PBX1
[CDKN2A &¬KRAS] | [¬PTEN &EWS_FLI1]
FLT3 | NOTCH1 | NRAS | EWS_FLI1
CDKN2A
FLT3 | NOTCH1 | NRAS | EWS_FLI1
FLT3 | NOTCH1 | EWS_FLI1 | TCF3_PBX1
NOTCH1
Genentech Cpd 10
Target: AURKA,AURKB
IC50s (log uM)
sensitive (40)
resistant (327)

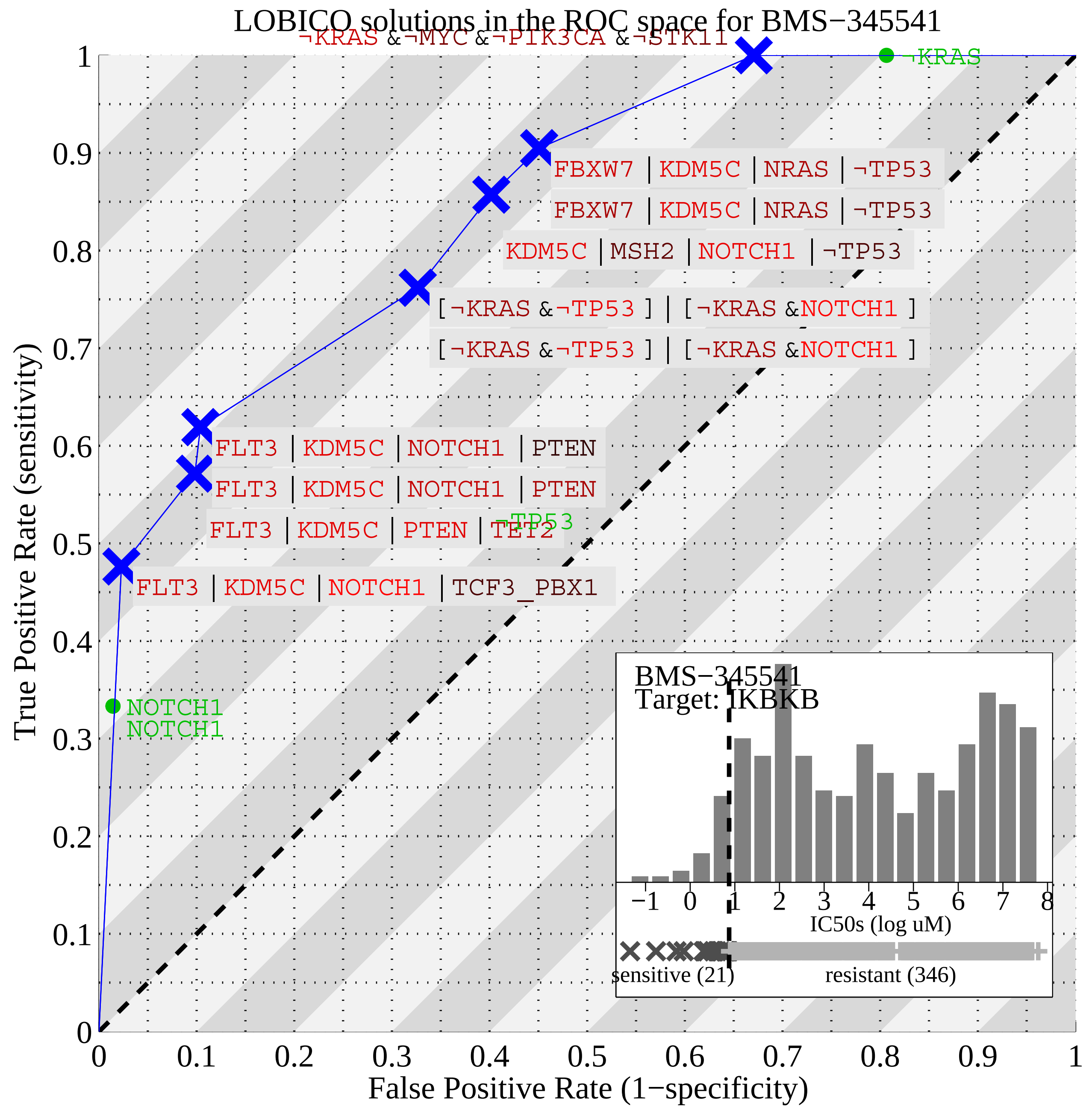
LOBICO solutions in the ROC space for BMS−345541
¬KRAS &¬MYC &¬PIK3CA &¬STK11
¬KRAS
FBXW7 | KDM5C | NRAS | ¬TP53
FBXW7 | KDM5C | NRAS | ¬TP53
KDM5C | MSH2 | NOTCH1 | ¬TP53
[¬KRAS &¬TP53] | [¬KRAS &NOTCH1]
[¬KRAS &¬TP53] | [¬KRAS &NOTCH1]
FLT3 | KDM5C | NOTCH1 | PTEN
FLT3 | KDM5C | NOTCH1 | PTEN
FLT3 | KDM5C | PTEN | TET2
¬TP53
FLT3 | KDM5C | NOTCH1 | TCF3_PBX1
NOTCH1
NOTCH1
True Positive Rate (sensitivity)
False Positive Rate (1−specificity)
BMS−345541
Target: IKBKB
IC50s (log uM)
sensitive (21)
resistant (346)

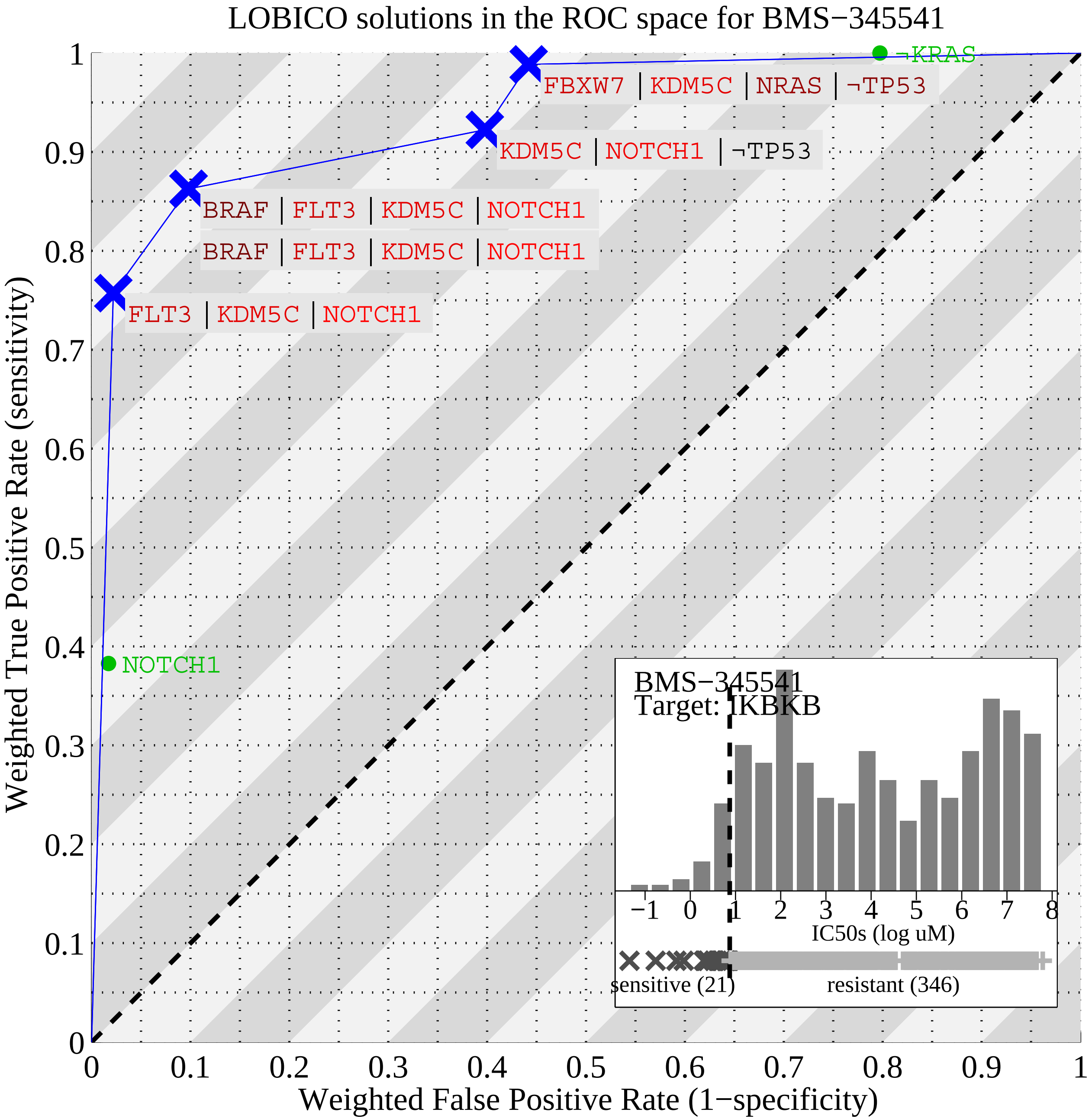

LOBICO solutions in the ROC space for BMS−345541
Weighted True Positive Rate (sensitivity)
Weighted False Positive Rate (1−specificity)
0
0.1
0.2
0.3
0.4
0.5
0.6
0.7
0.8
0.9
1
¬KRAS
FBXW7 | KDM5C | NRAS | ¬TP53
KDM5C | NOTCH1 | ¬TP53
BRAF | FLT3 | KDM5C | NOTCH1
BRAF | FLT3 | KDM5C | NOTCH1
FLT3 | KDM5C | NOTCH1
NOTCH1
BMS−345541
Target: IKBKB
−1 0 1 2 3 4 5 6 7 8
IC50s (log uM)
sensitive (21)
resistant (346)

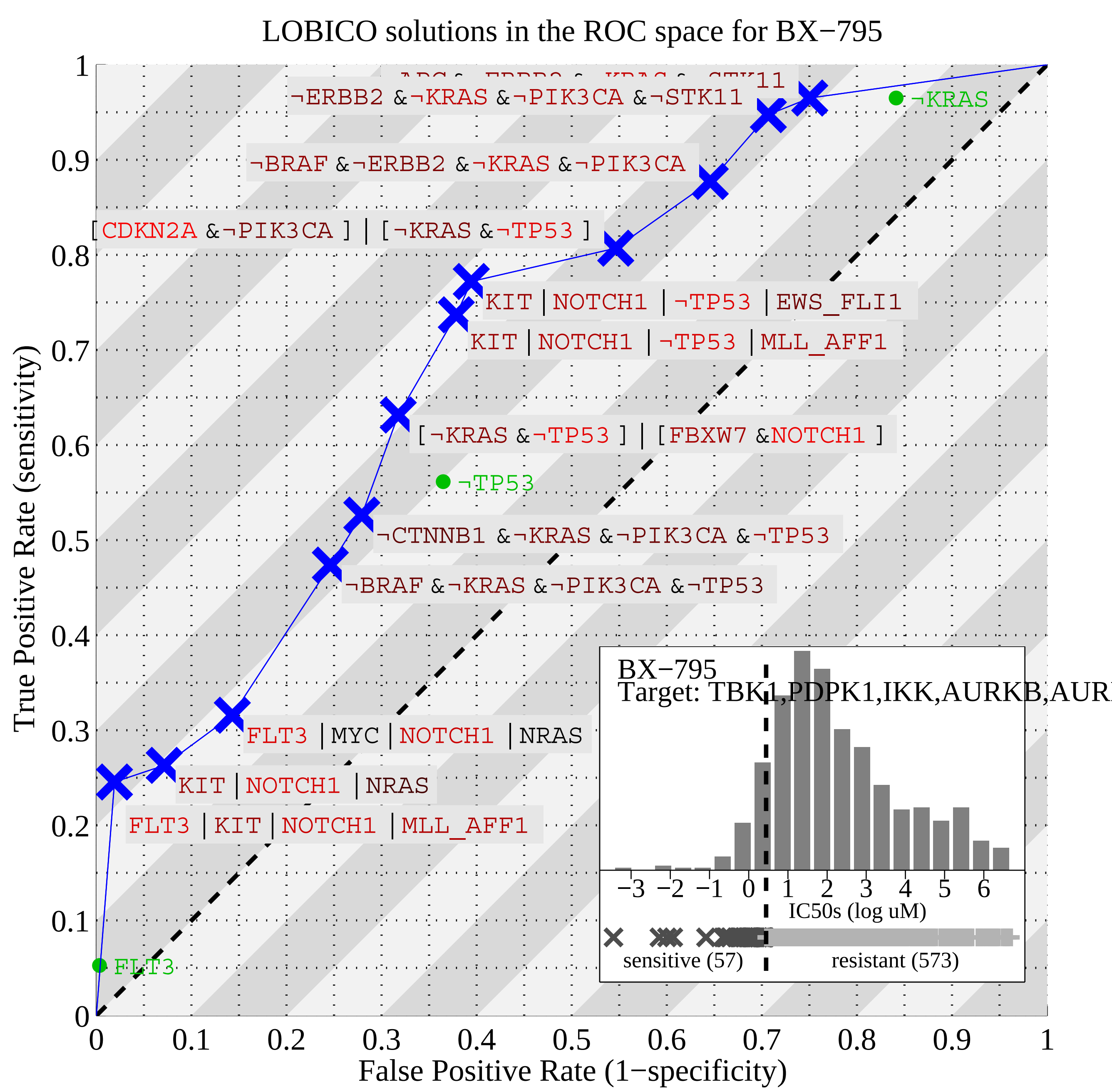

LOBICO solutions in the ROC space for BX−795
¬ERBB2 &¬KRAS &¬PIK3CA &¬STK11
¬KRAS
¬BRAF &¬ERBB2 &¬KRAS &¬PIK3CA
[CDKN2A &¬PIK3CA] | [¬KRAS &¬TP53]
KIT | NOTCH1 | ¬TP53 | EWS_FLI1
KIT | NOTCH1 | ¬TP53 | MLL_AFF1
[¬KRAS &¬TP53] | [FBXW7 &NOTCH1]
¬TP53
¬CTNNB1 &¬KRAS &¬PIK3CA &¬TP53
¬BRAF &¬KRAS &¬PIK3CA &¬TP53
FLT3 | MYC | NOTCH1 | NRAS
KIT | NOTCH1 | NRAS
FLT3 | KIT | NOTCH1 | MLL_AFF1
FLT3
BX−795
Target: TBK1,PDPK1,IKK,AURKB,AUR
−3 −2 −1 0 1 2 3 4 5 6
IC50s (log uM)
sensitive (57)
resistant (573)
True Positive Rate (sensitivity)
False Positive Rate (1−specificity)
0 0.1 0.2 0.3 0.4 0.5 0.6 0.7 0.8 0.9 1

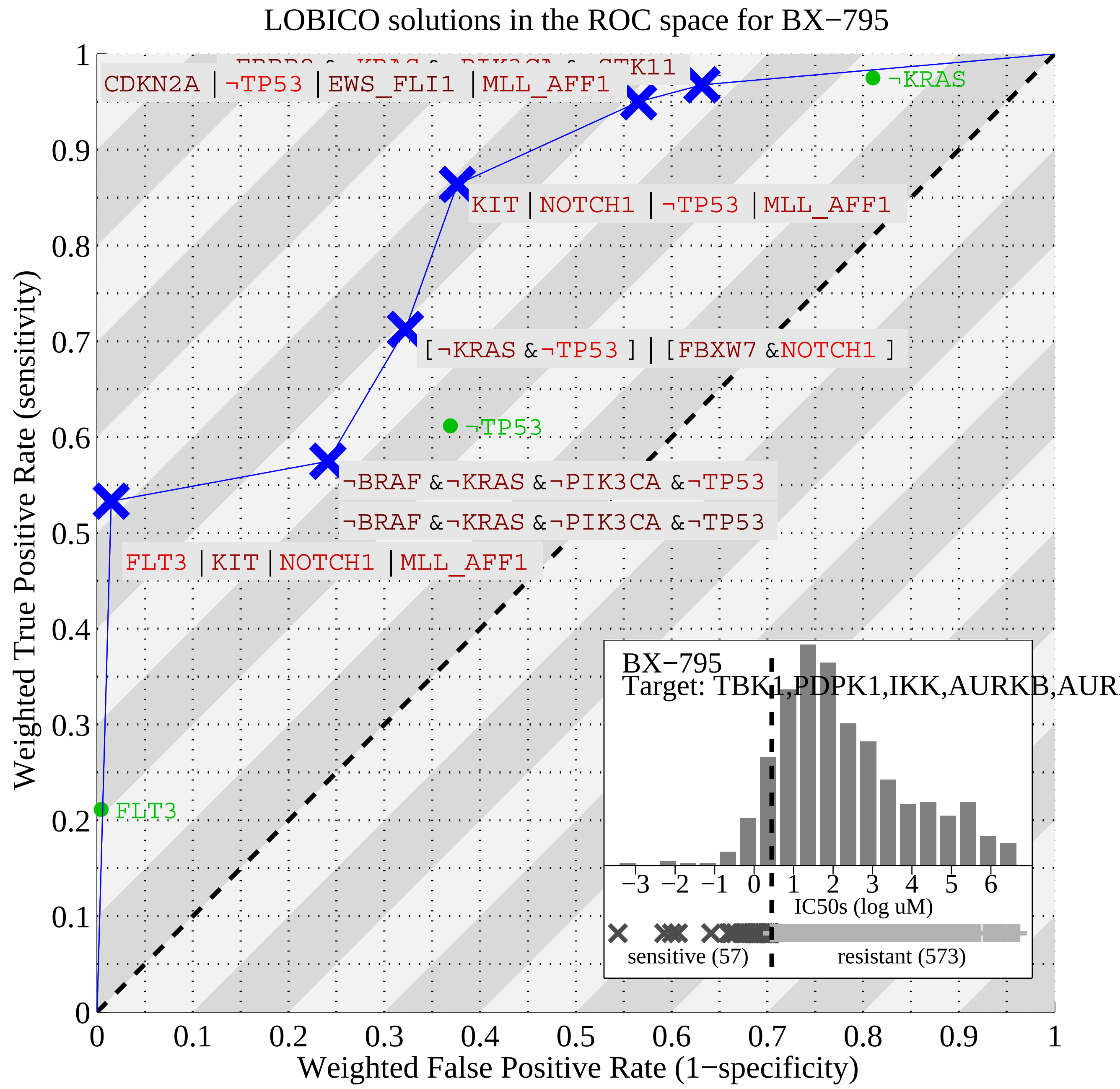

LOBICO solutions in the ROC space for BX−795
CDKN2A | ¬TP53 | EWS_FLI1 | MLL_AFF1
¬KRAS
KIT | NOTCH1 | ¬TP53 | MLL_AFF1
[¬KRAS &¬TP53] | [FBXW7 &NOTCH1]
¬TP53
¬BRAF &¬KRAS &¬PIK3CA &¬TP53
¬BRAF &¬KRAS &¬PIK3CA &¬TP53
FLT3 | KIT | NOTCH1 | MLL_AFF1
FLT3
BX−795
IC50s (log uM)
sensitive (57)
resistant (573)
Weighted True Positive Rate (sensitivity)
Weighted False Positive Rate (1−specificity)

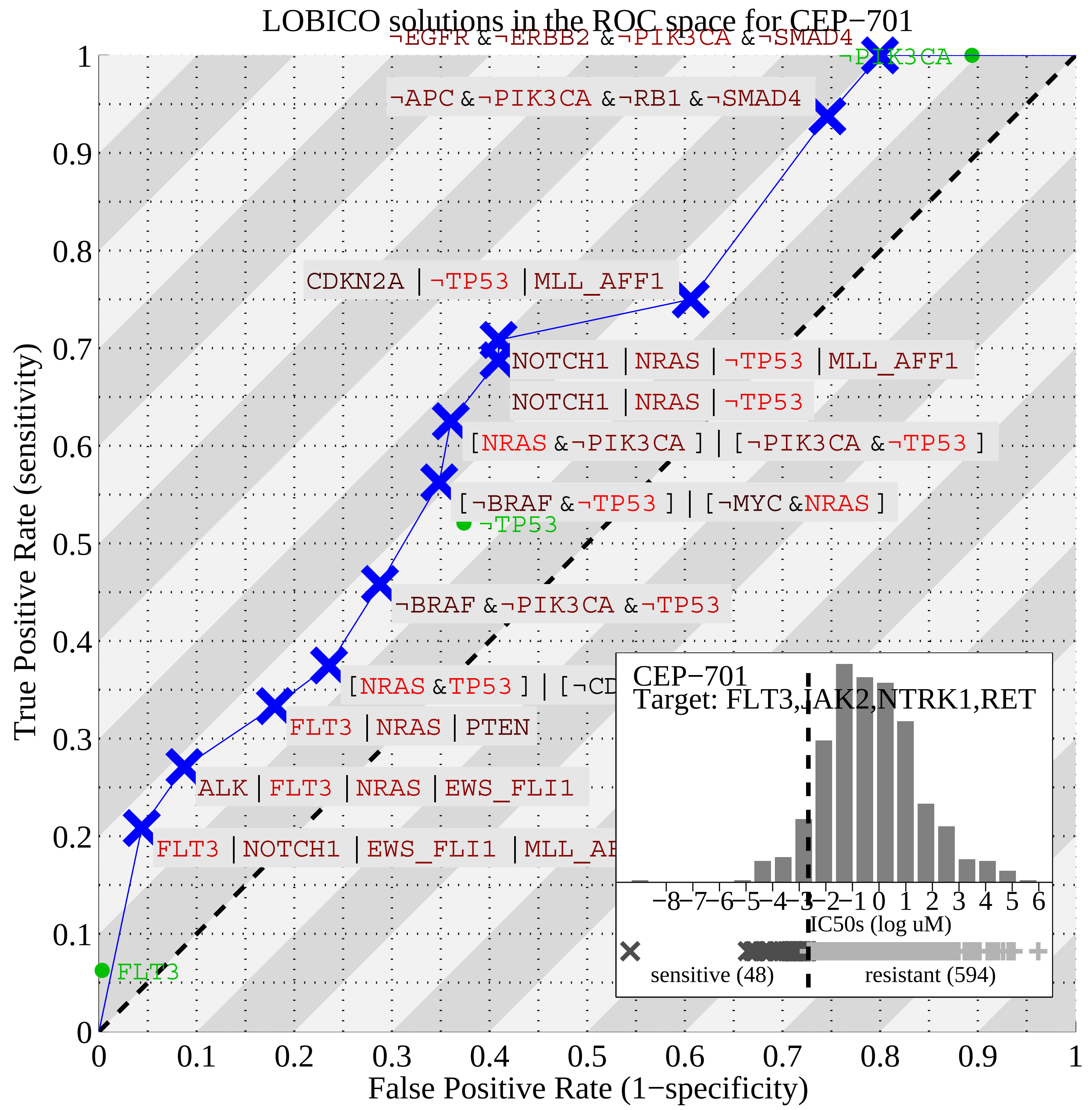

LOBICO solutions in the ROC space for CEP−701
¬EGFR &¬ERBB2 &¬PIK3CA &¬SMAD4
¬PIK3CA
¬APC &¬PIK3CA &¬RB1 &¬SMAD4
CDKN2A |¬TP53 |MLL_AFF1
NOTCH1 |NRAS |¬TP53 |MLL_AFF1
NOTCH1 |NRAS |¬TP53
[NRAS &¬PIK3CA] | [¬PIK3CA &¬TP53]
[¬BRAF &¬TP53] | [¬MYC &NRAS]
¬TP53
¬BRAF &¬PIK3CA &¬TP53
[NRAS &TP53] | [¬CD
FLT3 |NRAS |PTEN
ALK |FLT3 |NRAS |EWS_FLI1
FLT3 |NOTCH1 |EWS_FLI1 |MLL_AF
FLT3
True Positive Rate (sensitivity)
False Positive Rate (1−specificity)
CEP−701
Target: FLT3,JAK2,NTRK1,RET
IC50s (log uM)
sensitive (48)
resistant (594)

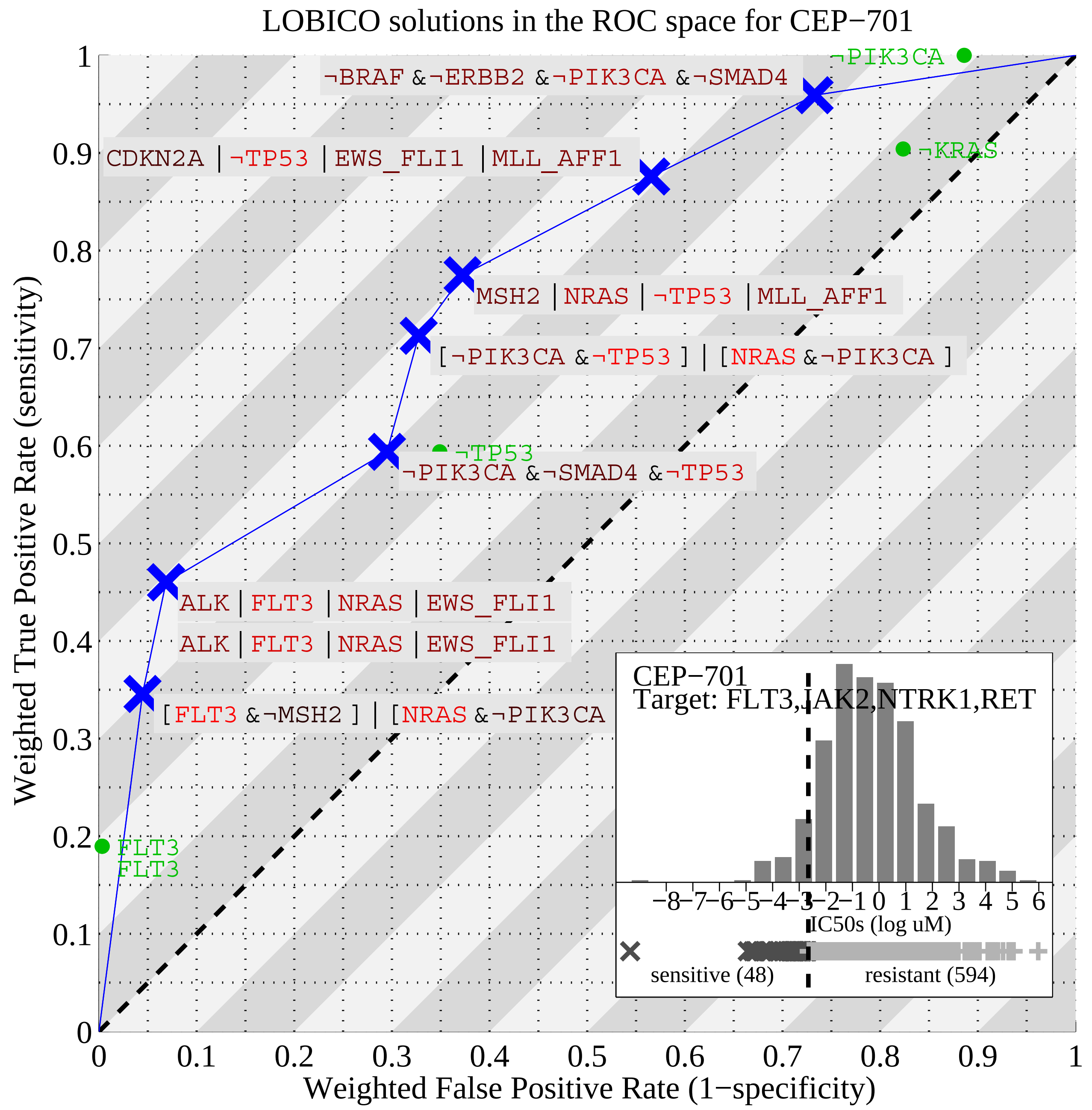

LOBICO solutions in the ROC space for CEP−701
¬BRAF &¬ERBB2 &¬PIK3CA &¬SMAD4
¬PIK3CA
CDKN2A |¬TP53 |EWS_FLI1 |MLL_AFF1
¬KRAS
MSH2 |NRAS |¬TP53 |MLL_AFF1
[¬PIK3CA &¬TP53] | [NRAS &¬PIK3CA]
¬TP53
¬PIK3CA &¬SMAD4 &¬TP53
ALK |FLT3 |NRAS |EWS_FLI1
ALK |FLT3 |NRAS |EWS_FLI1
[FLT3 &¬MSH2] | [NRAS &¬PIK3CA
FLT3
FLT3
CEP−701
Target: FLT3,JAK2,NTRK1,RET
−8 −7 −6 −5 −4 −3 −2 −1 0 1 2 3 4 5 6
IC50s (log uM)
sensitive (48)
resistant (594)
Weighted True Positive Rate (sensitivity)
Weighted False Positive Rate (1−specificity)
0 0.1 0.2 0.3 0.4 0.5 0.6 0.7 0.8 0.9 1

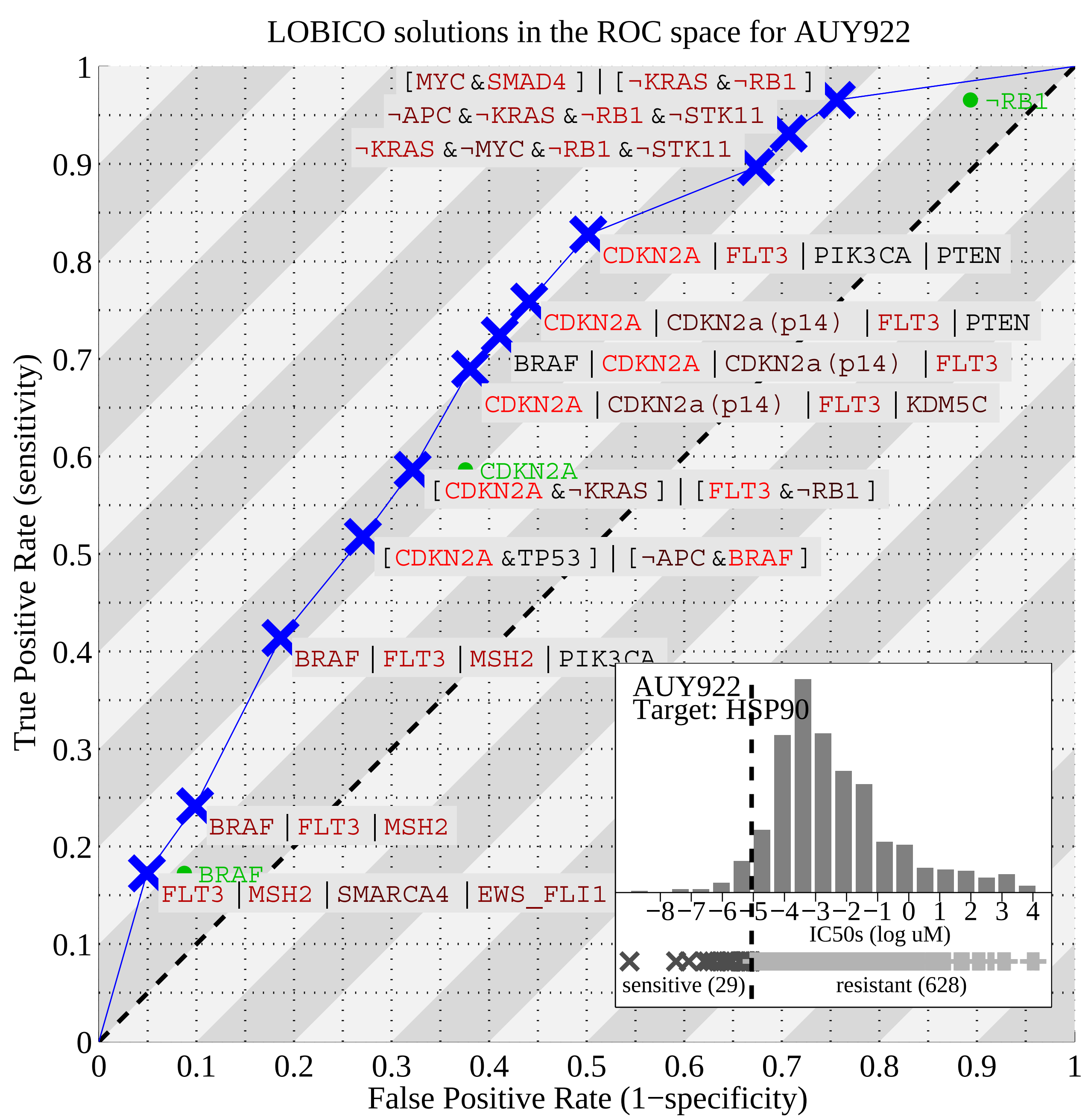
LOBICO solutions in the ROC space for AUY922
True Positive Rate (sensitivity)
False Positive Rate (1−specificity)
[MYC &SMAD4] | [¬KRAS &¬RB1]
¬APC &¬KRAS &¬RB1 &¬STK11
¬KRAS &¬MYC &¬RB1 &¬STK11
¬RB1
CDKN2A | FLT3 | PIK3CA | PTEN
CDKN2A | CDKN2a (p14) | FLT3 | PTEN
BRAF | CDKN2A | CDKN2a (p14) | FLT3
CDKN2A | CDKN2a (p14) | FLT3 | KDM5C
CDKN2A
[CDKN2A &¬KRAS] | [FLT3 &¬RB1]
[CDKN2A &TP53] | [¬APC &BRAF]
BRAF | FLT3 | MSH2 | PIK3CA
BRAF | FLT3 | MSH2
BRAF
FLT3 | MSH2 | SMARCA4 | EWS_FLI1
AUY922
Target: HSP90
IC50s (log uM)
sensitive (29)
resistant (628)

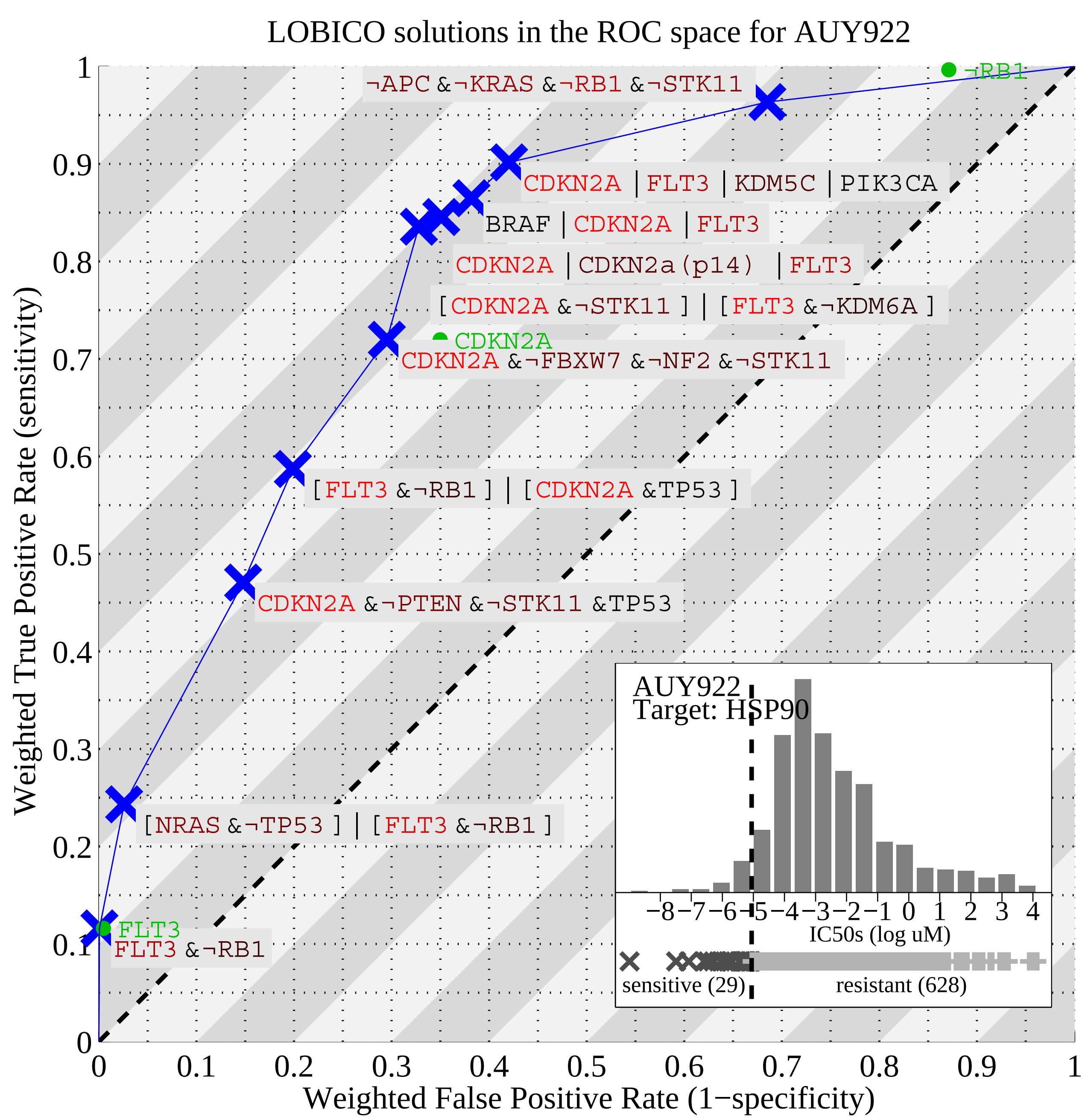
LOBICO solutions in the ROC space for AUY922
Weighted True Positive Rate (sensitivity)
Weighted False Positive Rate (1−specificity)
0
0.1
0.2
0.3
0.4
0.5
0.6
0.7
0.8
0.9
1
¬RB1
¬APC & ¬KRAS & ¬RB1 & ¬STK11
CDKN2A | FLT3 | KDM5C | PIK3CA
BRAF | CDKN2A | FLT3
CDKN2A | CDKN2a (p14) | FLT3
[CDKN2A & ¬STK11] | [FLT3 & ¬KDM6A]
CDKN2A
CDKN2A & ¬FBXW7 & ¬NF2 & ¬STK11
[FLT3 & ¬RB1] | [CDKN2A & TP53]
CDKN2A & ¬PTEN & ¬STK11 & TP53
[NRAS & ¬TP53] | [FLT3 & ¬RB1]
FLT3
FLT3 & ¬RB1
AUY922
Target: HSP90
−8 −7 −6 −5 −4 −3 −2 −1 0 1 2 3 4
IC50s (log uM)
sensitive (29)
resistant (628)

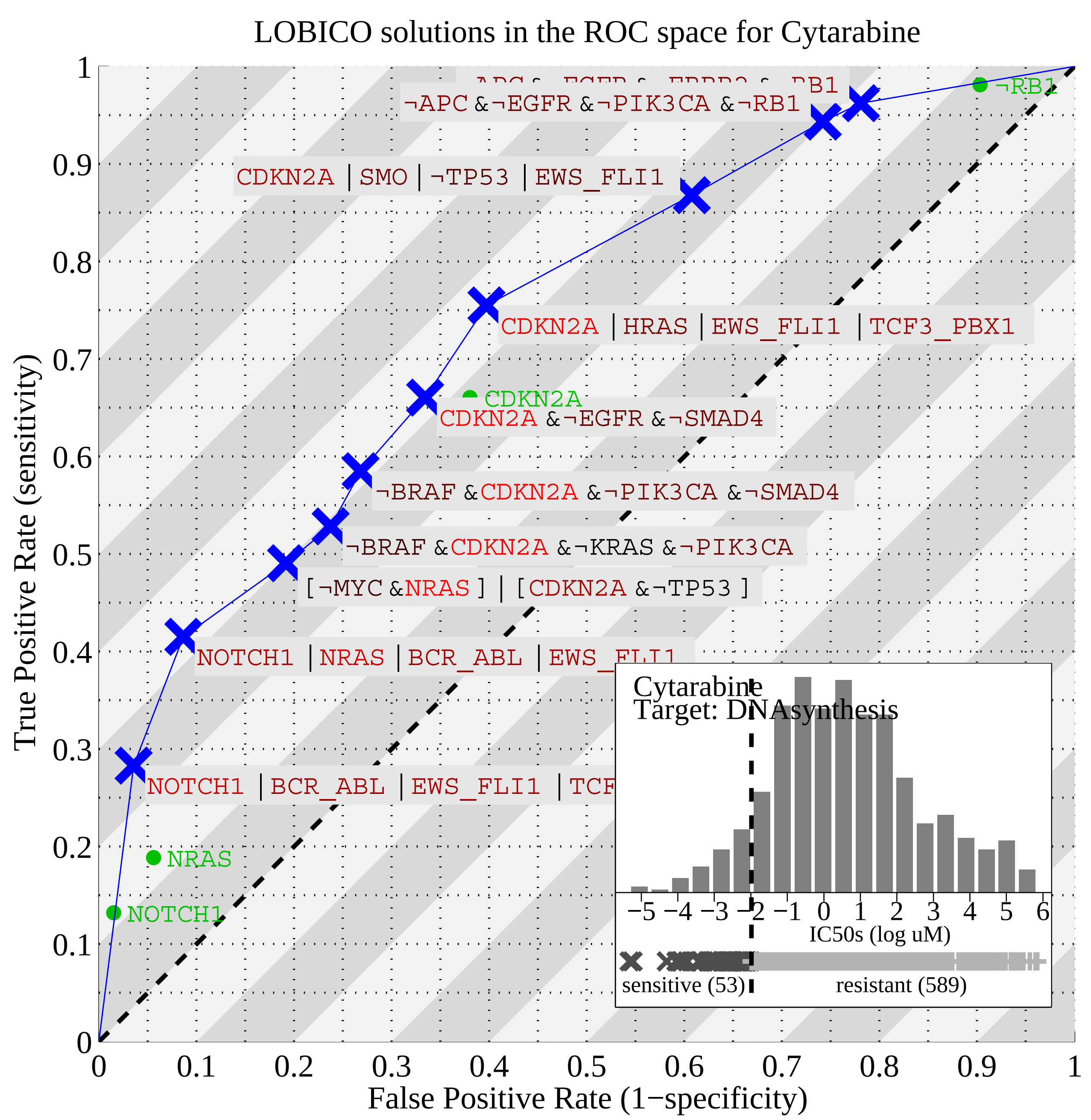

LOBICO solutions in the ROC space for Cytarabine
¬APC &¬EGFR &¬PIK3CA &¬RB1
¬RB1
CDKN2A | SMO | ¬TP53 | EWS_FLI1
CDKN2A | HRAS | EWS_FLI1 | TCF3_PBX1
CDKN2A
CDKN2A &¬EGFR &¬SMAD4
¬BRAF &CDKN2A &¬PIK3CA &¬SMAD4
¬BRAF &CDKN2A &¬KRAS &¬PIK3CA
[¬MYC &NRAS] | [CDKN2A &¬TP53]
NOTCH1 | NRAS | BCR_ABL | EWS_FLI1
NOTCH1 | BCR_ABL | EWS_FLI1 | TCF
NRAS
NOTCH1
True Positive Rate (sensitivity)
False Positive Rate (1−specificity)
0
0.1
0.2
0.3
0.4
0.5
0.6
0.7
0.8
0.9
1
Cytarabine
Target: DNAsynthesis
−5 −4 −3 −2 −1 0 1 2 3 4 5 6
IC50s (log uM)
sensitive (53)
resistant (589)

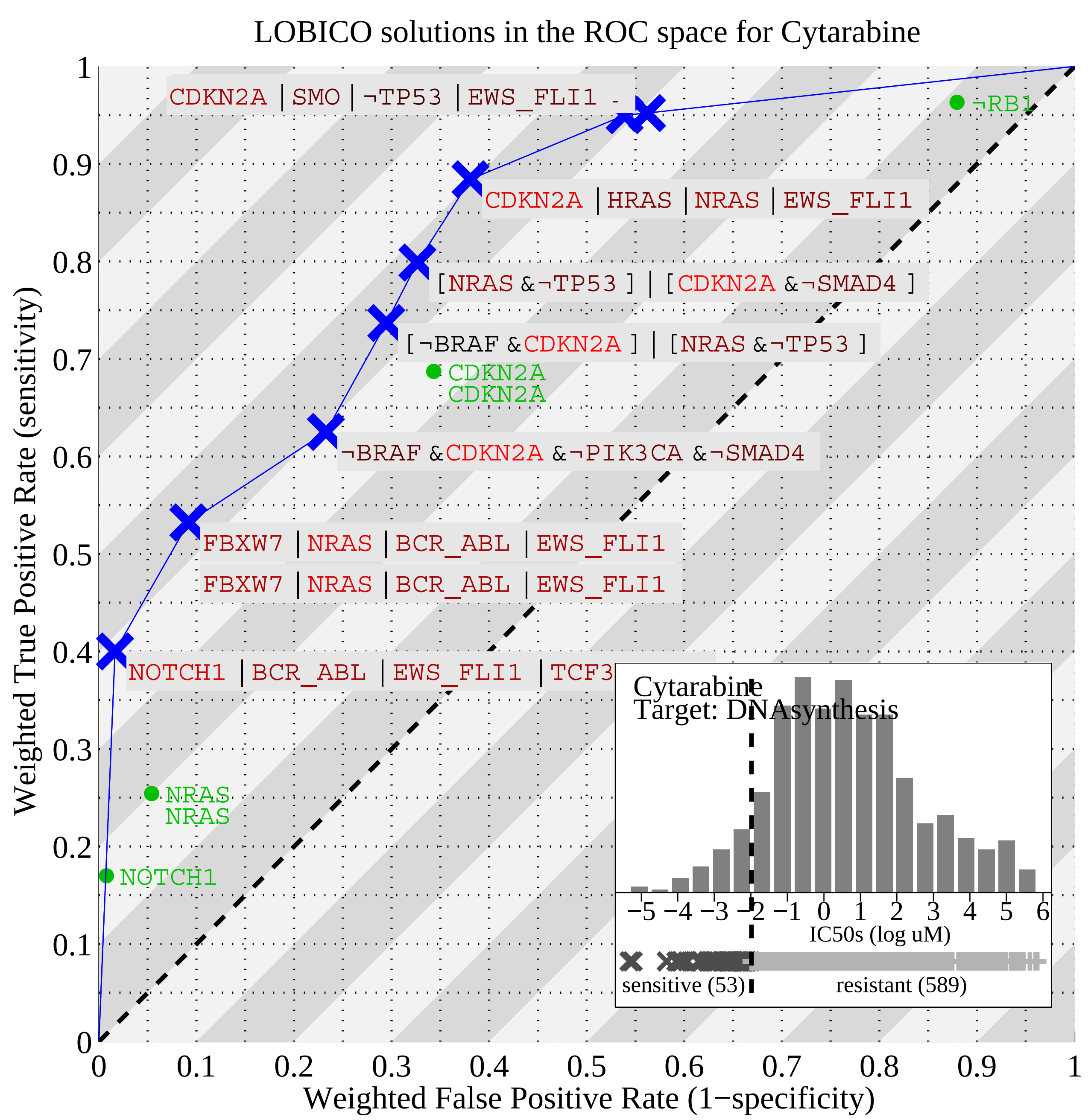

LOBICO solutions in the ROC space for Cytarabine
Weighted True Positive Rate (sensitivity)
Weighted False Positive Rate (1−specificity)
0
0.1
0.2
0.3
0.4
0.5
0.6
0.7
0.8
0.9
1
CDKN2A | SMO | ¬TP53 | EWS_FLI1
¬RB1
CDKN2A | HRAS | NRAS | EWS_FLI1
[NRAS & ¬TP53] | [CDKN2A & ¬SMAD4]
[¬BRAF & CDKN2A] | [NRAS & ¬TP53]
CDKN2A
CDKN2A
¬BRAF & CDKN2A & ¬PIK3CA & ¬SMAD4
FBXW7 | NRAS | BCR_ABL | EWS_FLI1
FBXW7 | NRAS | BCR_ABL | EWS_FLI1
NOTCH1 | BCR_ABL | EWS_FLI1 | TCF3
NRAS
NRAS
NOTCH1
Cytarabine
Target: DNAsynthesis
−5
−4
−3
−2
−1
0
1
2
3
4
5
6
IC50s (log uM)
sensitive (53)
resistant (589)

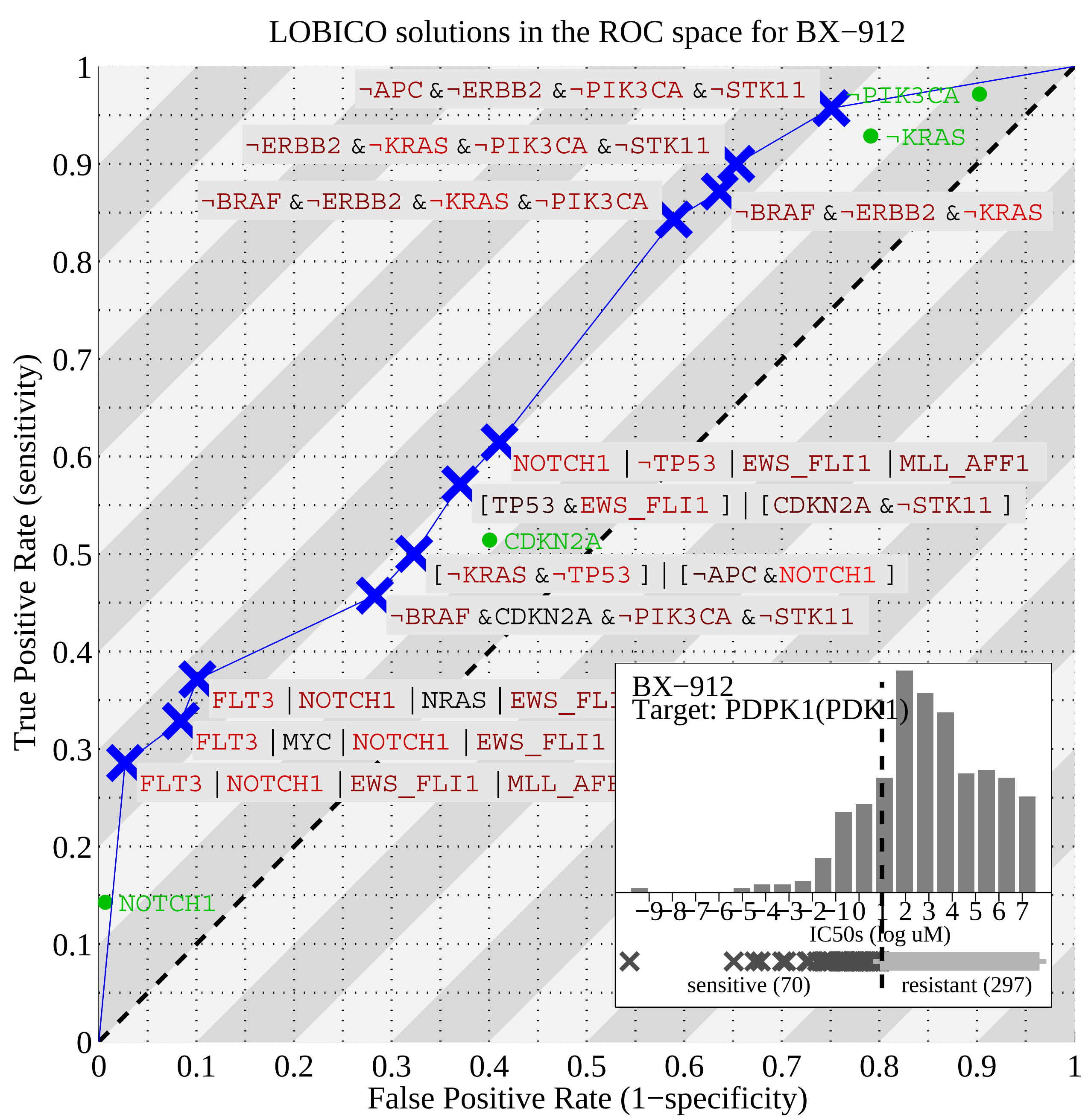

LOBICO solutions in the ROC space for BX−912
True Positive Rate (sensitivity)
False Positive Rate (1−specificity)
¬APC &¬ERBB2 &¬PIK3CA &¬STK11
¬ERBB2 &¬KRAS &¬PIK3CA &¬STK11
¬BRAF &¬ERBB2 &¬KRAS &¬PIK3CA
¬BRAF &¬ERBB2 &¬KRAS
¬PIK3CA
¬KRAS
NOTCH1 |¬TP53 |EWS_FLI1 |MLL_AFF1
[TP53 &EWS_FLI1] | [CDKN2A &¬STK11]
CDKN2A
[¬KRAS &¬TP53] | [¬APC &NOTCH1]
¬BRAF &CDKN2A &¬PIK3CA &¬STK11
FLT3 |NOTCH1 |NRAS |EWS_FLI
FLT3 |MYC |NOTCH1 |EWS_FLI1
FLT3 |NOTCH1 |EWS_FLI1 |MLL_AFF
NOTCH1
BX−912
Target: PDPK1(PDK1)
IC50s (log uM)
sensitive (70)
resistant (297)

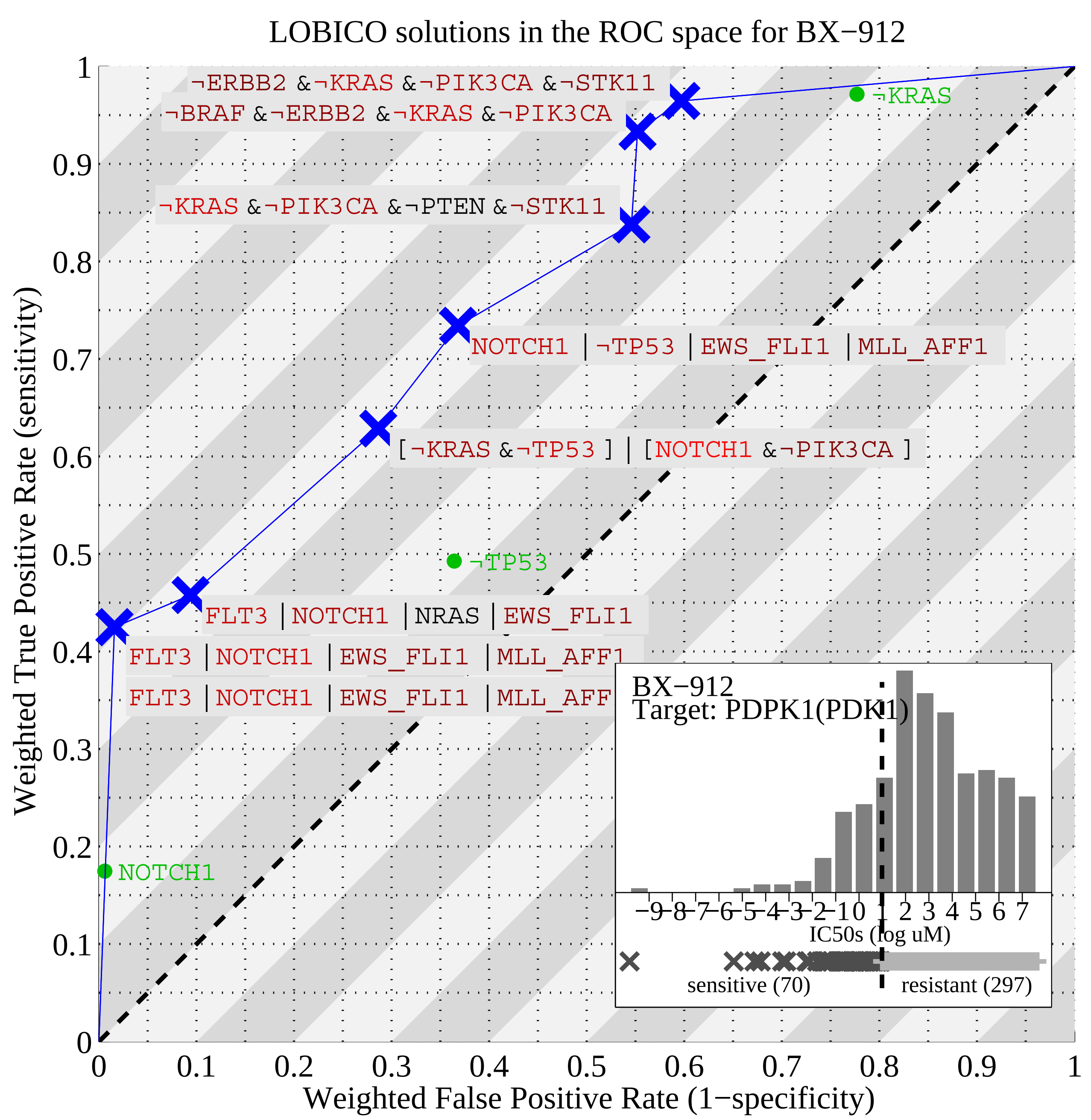

LOBICO solutions in the ROC space for BX−912
¬ERBB2 &¬KRAS &¬PIK3CA &¬STK11
¬BRAF &¬ERBB2 &¬KRAS &¬PIK3CA
¬KRAS &¬PIK3CA &¬PTEN &¬STK11
NOTCH1 |¬TP53 |EWS_FLI1 |MLL_AFF1
[¬KRAS &¬TP53] | [NOTCH1 &¬PIK3CA]
¬KRAS
¬TP53
FLT3 |NOTCH1 |NRAS |EWS_FLI1
FLT3 |NOTCH1 |EWS_FLI1 |MLL_AFF1
NOTCH1
BX−912
Target: PDPK1(PDK1)
IC50s (log uM)
sensitive (70)
resistant (297)
Weighted True Positive Rate (sensitivity)
Weighted False Positive Rate (1−specificity)

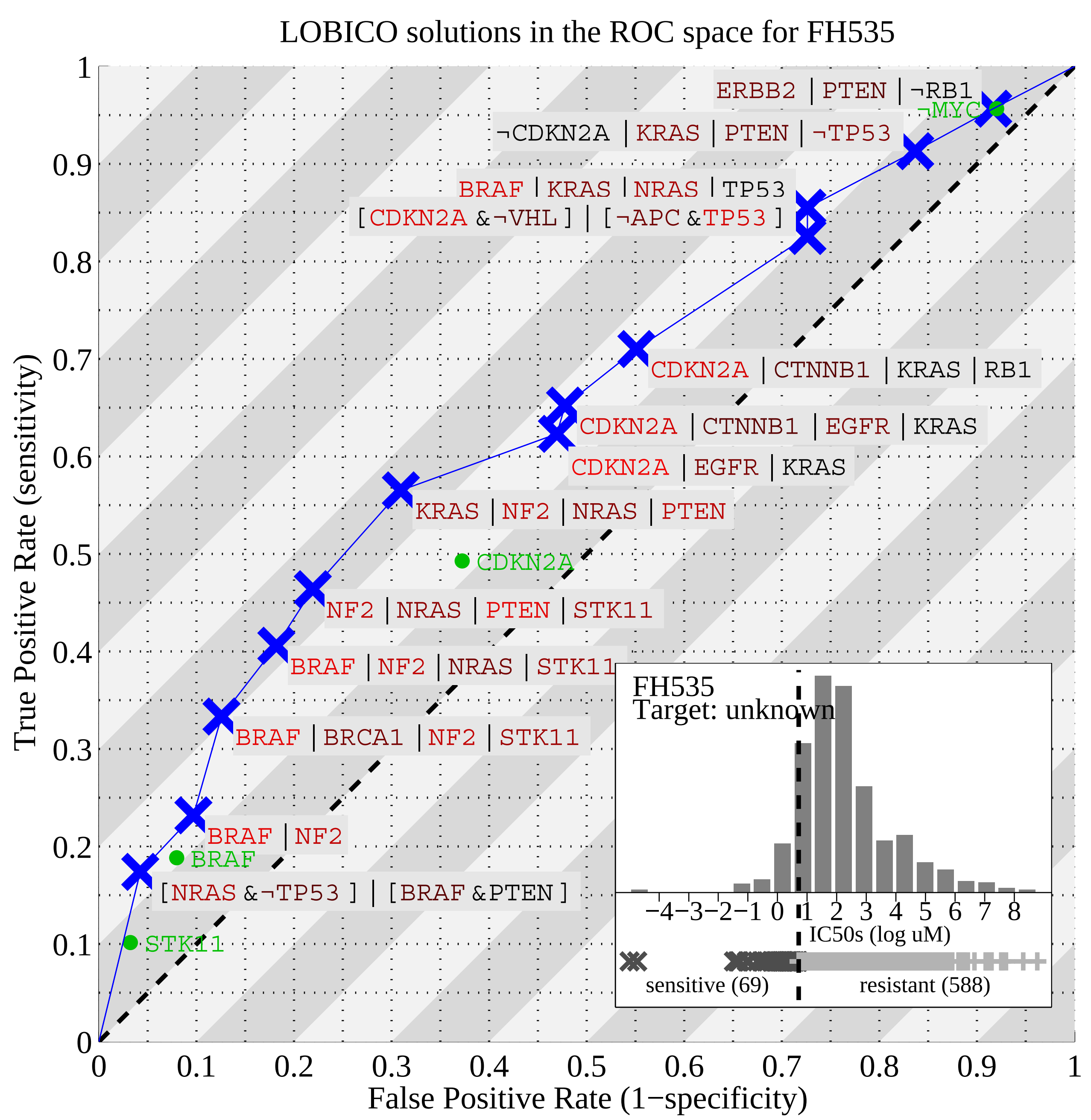
LOBICO solutions in the ROC space for FH535
True Positive Rate (sensitivity)
False Positive Rate (1−specificity)
0
0.1
0.2
0.3
0.4
0.5
0.6
0.7
0.8
0.9
1
ERBB2 | PTEN | ¬RB1
¬MYC
¬CDKN2A | KRAS | PTEN | ¬TP53
BRAF | KRAS | NRAS | TP53
[CDKN2A & ¬VHL] | [¬APC & TP53]
CDKN2A | CTNNB1 | KRAS | RB1
CDKN2A | CTNNB1 | EGFR | KRAS
CDKN2A | EGFR | KRAS
KRAS | NF2 | NRAS | PTEN
CDKN2A
NF2 | NRAS | PTEN | STK11
BRAF | NF2 | NRAS | STK11
BRAF | BRCA1 | NF2 | STK11
BRAF | NF2
BRAF
[NRAS & ¬TP53] | [BRAF & PTEN]
STK11
FH535
Target: unknown
−4 −3 −2 −1 0 1 2 3 4 5 6 7 8
IC50s (log uM)
sensitive (69)
resistant (588)

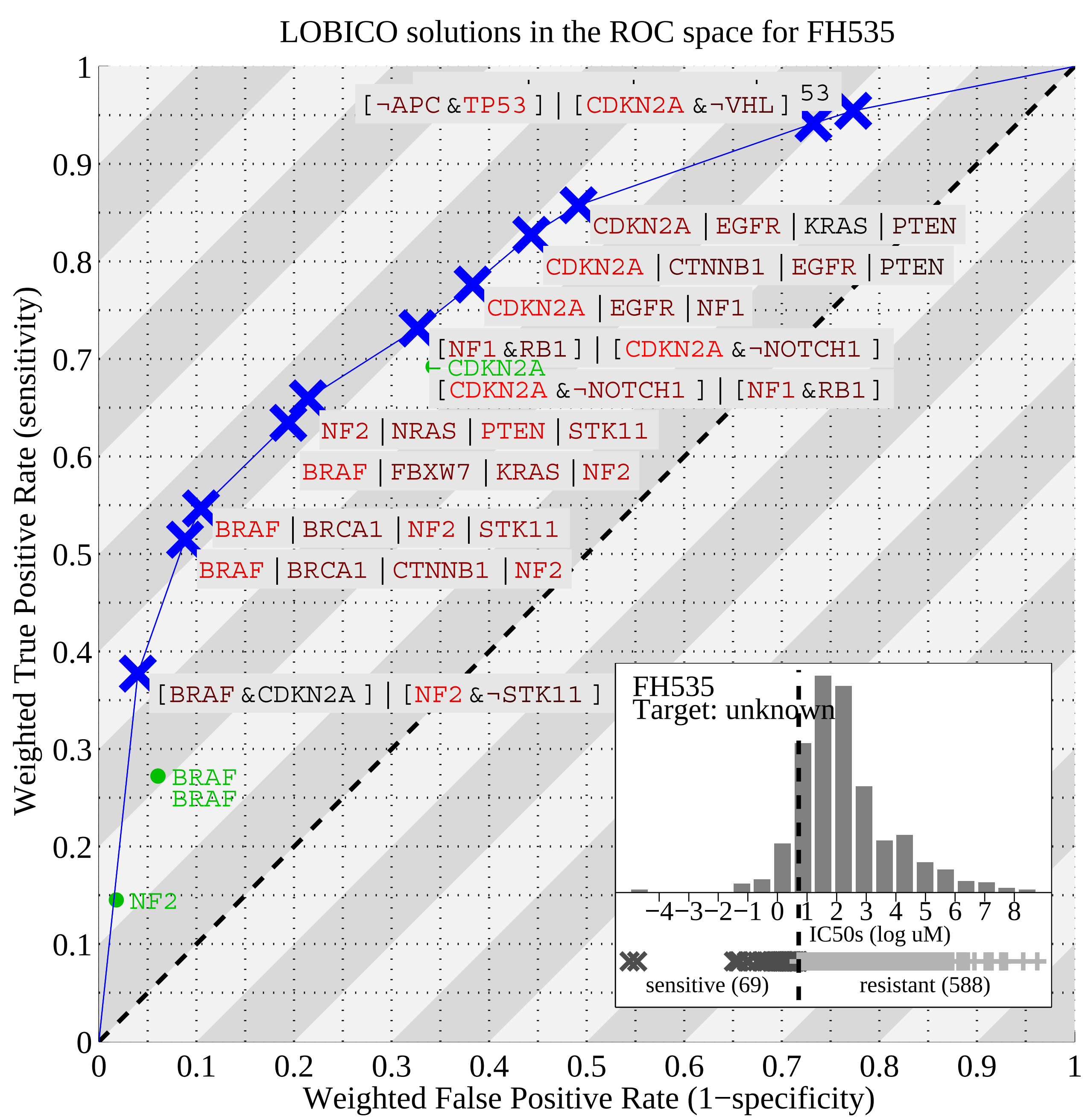

LOBICO solutions in the ROC space for FH535
Weighted True Positive Rate (sensitivity)
Weighted False Positive Rate (1−specificity)
0
0.1
0.2
0.3
0.4
0.5
0.6
0.7
0.8
0.9
1
[¬APC &TP53] | [CDKN2A &¬VHL]
53
CDKN2A | EGFR | KRAS | PTEN
CDKN2A | CTNNB1 | EGFR | PTEN
CDKN2A | EGFR | NF1
[NF1 &RB1] | [CDKN2A &¬NOTCH1]
CDKN2A
[CDKN2A &¬NOTCH1] | [NF1 &RB1]
NF2 | NRAS | PTEN | STK11
BRAF | FBXW7 | KRAS | NF2
BRAF | BRCA1 | NF2 | STK11
BRAF | BRCA1 | CTNNB1 | NF2
[BRAF &CDKN2A] | [NF2 &¬STK11]
BRAF
BRAF
NF2
FH535
Target: unknown
−4 −3 −2 −1 0 1 2 3 4 5 6 7 8
IC50s (log uM)
sensitive (69)
resistant (588)
